# Computational Study of Alkyne‐Acid Cycloisomerization in Gold‐Functionalized Resorcinarene Cavitand

**DOI:** 10.1002/chem.202404480

**Published:** 2025-03-10

**Authors:** Joannes Peters, Fahmi Himo

**Affiliations:** ^1^ Department of Chemistry Arrhenius Laboratory Stockholm University SE-10691 Stockholm Sweden

**Keywords:** supramolecular chemistry, cavitand, density functional theory, cycloisomerization, gold

## Abstract

Herein, we report density functional theory calculations to investigate the reaction mechanism and the selectivity of the cycloisomerization of alkynoic acids inside a gold‐functionalized resorcinarene‐based cavitand. This cavitand has experimentally been shown to catalyze the cycloisomerization of a number of substituted alkynoic acids to give the corresponding γ‐lactones. Three representative substrates, with different substitution patterns, are considered in the calculations, and for each of them, the geometries and energies of various binding modes are first characterized. Next, the cycloisomerization reaction mechanism is evaluated, which is shown to consist of an intramolecular addition of the carboxylic acid to the gold‐activated alkyne triple bond, followed by a protodeauration step. Both 5‐*exo*‐dig and 6‐*endo*‐dig cyclizations were considered, leading to γ‐ or δ‐lactones, respectively, and the involvement of the triflate counterion in the cyclization step is discussed. Finally, the influence of the cavitand walls on the reactivity is evaluated by using a model catalyst in which the walls were removed.

## Graphical abstract

The binding and cycloisomerization of acetylenic acids to give the corresponding γ‐lactones in a gold‐functionalized resorcinarene‐based cavitand are studied by means of density functional theory calculations. The reaction mechanism and selectivity are investigated for substrates with different substituents to assess the size‐reactivity relationships. The influence of the cavitand walls on the reactivity and selectivity is examined using a stripped‐down model.

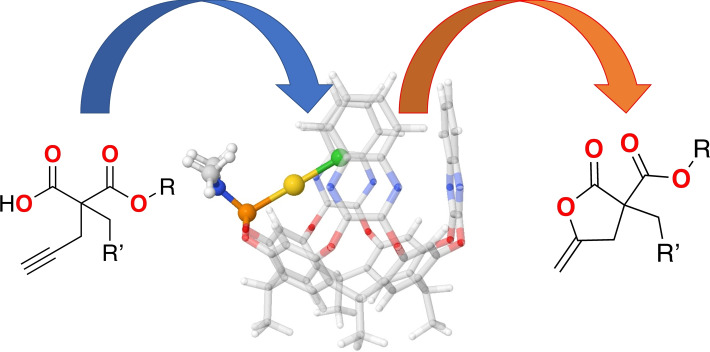

## 1 Introduction

Supramolecular catalysts accelerate chemical reactions through the use of non‐covalent interactions, such as hydrogen bonds, π‐π and cation‐π interactions.[^1^, ^2^, ^3^, ^4^, ^5^, ^6^, ^7^, ^8^, ^9^, ^10^] Many different types of supramolecular hosts have been synthesized over the years to achieve catalysis, such as cyclodextrins,[^11^, ^12^, ^13^] cucurbit[n]urils,[^14^, ^15^] metal‐organic cages,[^16^, ^17^, ^18^, ^19^, ^20^, ^21^, ^22^] capsules and cavitands.[^23^, ^24^, ^25^, ^26^, ^27^]

**Figure 1:**
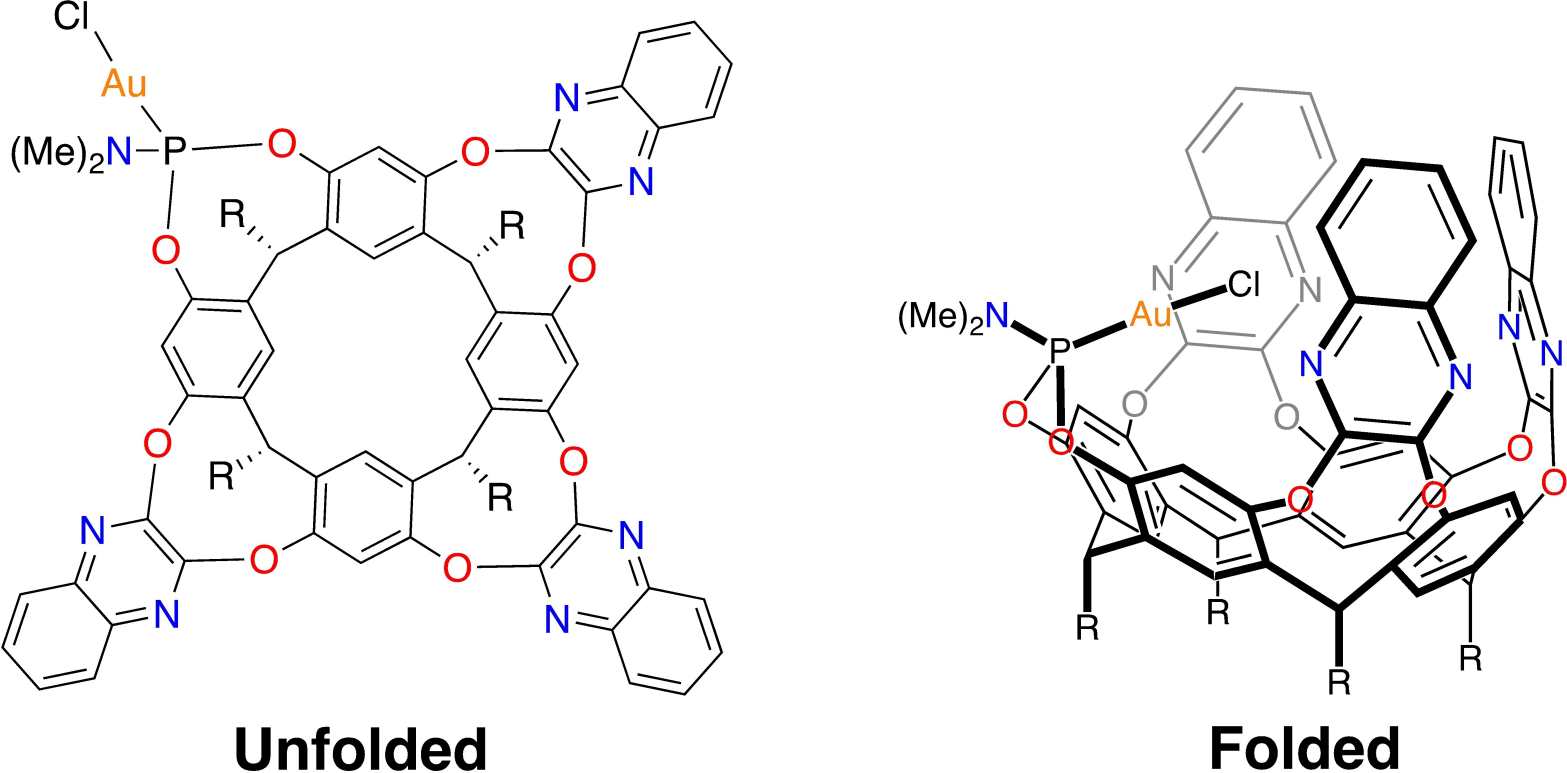
Schematic representation of gold‐functionalized cavitand **AuClCav** (R=C_11_H_23_).

One interesting development in this area is the functionalization of supramolecular hosts with a transition metal site.[^28^, ^29^, ^30^, ^31^] Such complexes allow for metal‐catalyzed reactions to occur in the confined space of the host, the effects of which can include increased rates and altered selectivities of the reactions. In particular, a number of supramolecular systems have been synthesized incorporating gold ion as a catalyst.[^32^, ^33^, ^34^, ^35^, ^36^, ^37^, ^38^, ^39^, ^40^, ^41^, ^42^] Among these complexes, an interesting catalyst is the resorcin[4]arene‐based cavitand synthesized by Iwasawa and co‐workers, where one cavitand wall was functionalized by a phosphine group with a coordinated gold(I)‐chloride molecule (Figure 1).^[40]^ This cavitand, which here will be referred to as **AuClCav**, has displayed catalytic activity for multiple well‐known gold‐catalyzed reactions, such as alkyne hydration,^[40]^ Conia‐ene reaction,^[40]^ cycloisomerization of acetylenic acids to lactones,^[41]^ and alkyne‐arene cycloisomerization reactions.^[42]^

**Scheme 1:**
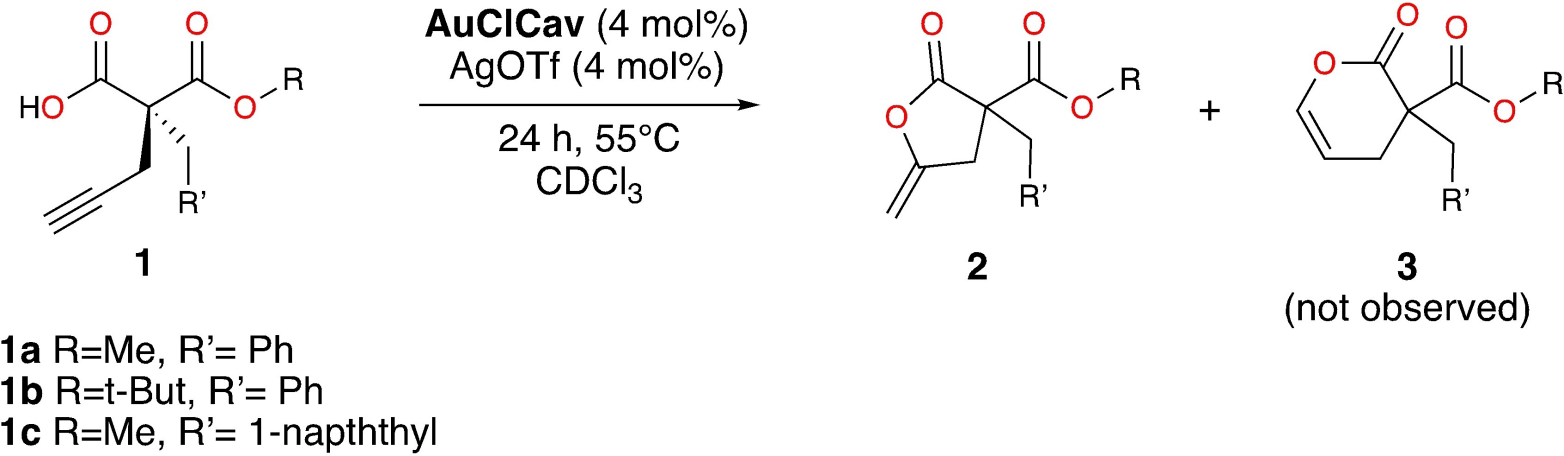
Cycloisomerization of acetylenic acids to γ‐lactones catalyzed by the cavitand considered in the current work.

The present study is focused on the cycloisomerization of alkyne acids to lactones by cavitand **AuClCav** reported by Ho and Schramm (Scheme 1).^[41]^ The gold‐catalyzed cycloisomerization of acetylenic acids to lactones is an important reaction with a perfect atom economy,[^43^, ^44^] and it has potential applications in, e. g., the synthesis of complex natural products and in polymer chemistry.[^45^, ^46^]

Ho and Schramm demonstrated that cavitand **AuClCav** is capable of cycloisomerizing a series of alkyne acids, with different substituents, which allowed for the exploration of the effects of the guest size on the reactivity in the host.^[41]^

In recent years, density functional theory (DFT) methods have become an important tool to investigate reactions in host‐guest complexes.[^47^, ^48^, ^49^, ^50^, ^51^, ^52^, ^53^, ^54^, ^55^, ^56^, ^57^, ^58^, ^59^, ^60^, ^61^, ^62^] In particular, reactions inside resorcinarene‐based capsules and cavitands have been successfully studied with this methodology,[^51^, ^63^, ^64^, ^65^, ^66^, ^67^] which is of relevance for the present work.

Herein, we present a computational study on the cycloisomerization of alkyne acids in **AuClCav** using DFT calculations (Scheme 1). We first focus on the binding modes of alkyne acid **1a** inside the cavitand, which is then followed by an investigation of the reaction mechanism for the cycloisomerization for this guest to its possible lactone products. Next, we investigate the effect of different substituents of the acetylenic acid by calculating the binding modes and reaction mechanisms of two other guest molecules for which the reaction kinetics have been measured, namely acetylenic acids **1b** and **1c** (Scheme 1). Finally, we investigate the effect of the cavitand itself on the energies of the reaction.

## Computational Details

All calculations were performed using the dispersion‐corrected B3LYP‐D3(BJ)[^68^, ^69^, ^70^, ^71^] functional as implemented in the Gaussian 16 software, revision C.01.^[72]^ Geometry optimizations were carried out in the gas phase using the 6‐31G(d,p) basis set for all atoms except the metal atoms, for which the SDD basis set was employed. The energies were refined using single‐point calculations with the 6‐311+G(2d,2p) basis set for all atoms except the metal atoms, for which the SDD basis set was maintained. Frequency calculations were carried out at the same level as the geometry optimization, and the thermal corrections to Gibbs free energies were evaluated with the quasi‐rigid rotor harmonic oscillator approximation with a cutoff value of 100 cm^−1^.^[73]^ Corrections for solvation were included as single‐point energy calculations using the SMD method, with chloroform as solvent.^[74]^ To assess the impact of performing the geometry optimizations in the gas phase, the geometries for one the reaction pathways with the model catalyst (Section 2.5. below) were re‐optimized in solvent and the energies re‐evaluated. These calculations showed that neither the geometries nor the energies were affected significantly (see results in the SI).

Standard state corrections were added to account for the change from a 1 atm gas to a 1 M solution state through the addition of a correction term of ‐RT ln (1/24.5)=+1.9 kcal/mol to the energies of all species, except for chloroform, for which a correction term of ‐RT ln (1/(24.5×12.5))=+3.4 kcal/mol was added.

As shown in Figure 1, the feet of the cavitand consist of C_11_H_23_ alkane chains. In the model, these moieties were truncated to methyl groups. A test optimization of the geometry of one host‐guest complex where the full feet were kept in the model showed that the structure of the cavitand is almost identical to the one with truncated feet (see SI), justifying this simplification of the cavitand feet.

Finally, a number of conformers were calculated for every stationary point to make sure that the lowest‐energy conformer is located, with special consideration given to guest orientation inside the cavitand.

## 2 Results and Discussion

As mentioned above, we have investigated the reactions of three different acetylenic acids: **1a**, **1b** and **1c** (Scheme 1). We begin the discussion by presenting the results concerning the geometries and energies of possible binding modes for **1a**, taking into account the potential participation of the chloroform solvent and triflate counterion molecules. Next, we focus on the reaction mechanism for the cycloisomerization of this guest molecule, considering pathways leading to the different products. Then, similar analyses are made for guests **1b** and **1c** to investigate the influence of the R and R’ substituents on the binding, reactivity, and selectivity of the reaction. Finally, the influence of the cavitand itself on the reactivity is discussed by calculating the cycloisomerization of **1a** using a model catalyst in which the walls of the cavitand have been removed.

Before discussing the results, however, it is important to note that the **AuClCav** cavitand is activated by reaction with AgOTf, creating the dechlorinated cationic cavitand **AuCav**.^[40]^ The calculations show that this activation is indeed energetically feasible, as the displacement equilibrium (1) is calculated to be exergonic by 6.9 kcal/mol (see SI).
(1)

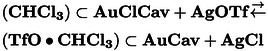




The calculations presented here therefore start from this activated form of the cavitand. Furthermore, the activation process creates catalytic amounts of TfO^−^ anion that can possibly influence the guest binding and the reactivity. We have therefore considered these possibilities in the calculations.

### 2.1 Binding of Acetylenic Acid 1a

**Figure 2:**
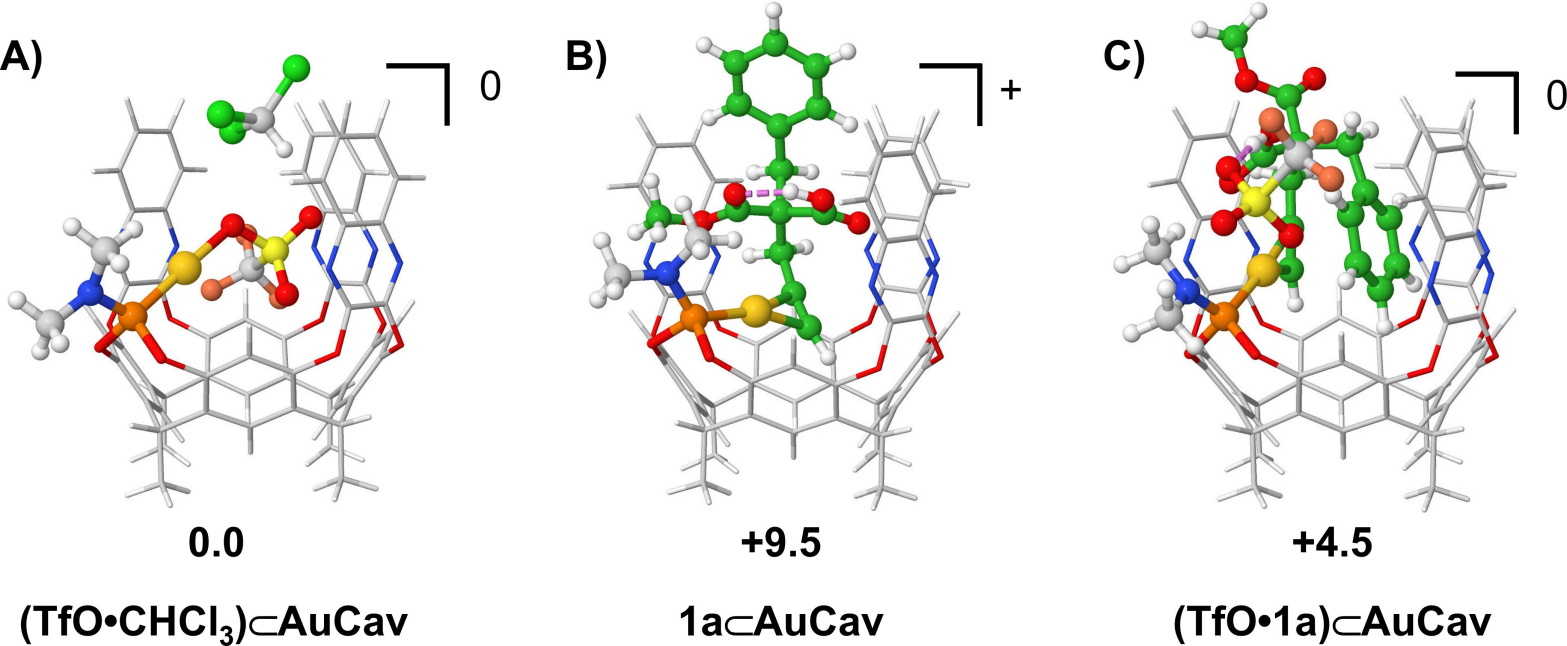
Optimized geometries of lowest‐energy conformers for possible guests in cavitand **AuCav**. Relative free energies are given in kcal/mol. A) triflate and chloroform; B) Acetylenic acid **1 a**; C) Acetylenic acid **1 a** and triflate.

There are a number of molecules present in the solution that could be possible guests in the cavitand, namely the acetylenic acid substrate **1a**, chloroform solvent molecules, and the triflate anion resulting from the activation reaction (see above). We have calculated the energies of possible combinations of these three potential guests that can fit inside the cavitand space (see SI). The lowest energy structure found is the one in which the triflate anion coordinates to the gold cation inside the cavitand, with one chloroform solvent molecule occupying the space on top of the triflate, as shown in Figure 2. This complex will be referred to as **(TfO**•**CHCl_3_)⊂AuCav** and will serve as the energy reference point for following free energy profiles.

An important note here concerns the free acetylenic acids in solution. In non‐polar solvents, such as chloroform, these acids form dimers that are more stable than the individual monomers (see SI), and the penalty for breaking these dimers has to be taken into account when calculating the energies for the binding and the reaction of the guests in the cavitand. In the case of **1a**, this dimerization energy amounts to 4.2 kcal/mol.

Replacement of the triflate and the chloroform solvent in **(TfO**•**CHCl_3_)⊂AuCav** by substrate **1a** to yield **1a⊂AuCav** leads to an energy increase of 9.5 kcal/mol (Figure 2). In the lowest‐energy structure of **1a⊂AuCav**, the alkyne group coordinates to the gold, with the acetylenic proton pointing toward to the bottom of the cavitand, while the benzyl group points out from the cavitand. The ester methyl group points to the area between the amine and one of the panels, while the carboxylic acid forms an intramolecular hydrogen bond with the ester oxygen.

We have considered many other binding modes, in which the various groups of the guest molecule point in different directions in the cavitand. All of these alternative orientations have higher energies, with seven of them within 7 kcal/mol from **1a⊂AuCav** (see SI).

Importantly, the calculations show that the triflate anion can form a favorable hydrogen bond with **1a⊂AuCav** (see Figure 2), resulting in a complex with lower energy, called **(TfO**•**1a)⊂AuCav**, which is 4.5 kcal/mol higher than **(TfO**•**CHCl_3_)⊂AuCav**. Interestingly, in complex **(TfO**•**1a)⊂AuCav**, the guest **1a** is oriented differently compared to in **1a⊂AuCav** without the triflate anion. Namely, the benzyl group points inward inside the cavitand, between the gold ion and one of the walls, while the ester group points outside the cavitand opening. The carboxylic acid forms a hydrogen bond with the triflate, which is located above the amine moiety. Many different binding modes were calculated for this complex as well, eight of which fall within 7 kcal/mol from **(TfO**•**1a)⊂AuCav** (see SI).

### 2.2 Cycloisomerization of 1a in Cavitand

In general, the gold‐catalyzed cycloisomerization of acetylenic acids is presumed to occur through an intramolecular addition of the carboxylic acid to the gold‐activated alkyne triple bond, followed by a protodeauration step to generate the product. The addition of the carboxylic acid to the alkyne in the case of pentynoic acid **1a** can take place through either a 5‐*exo*‐dig or a 6‐*endo*‐dig cyclization, leading to a γ‐ or a δ‐lactone, respectively. Moreover, in the case of internal alkynes, the *syn*‐addition to the Au−C bond in the 5‐*exo*‐dig cyclization will lead to an (*E*)‐*exo*‐alkylidene lactone, whereas the *anti*‐addition yields a (*Z*)‐*exo*‐alkylidene lactone. Experimentally, the cavitand‐catalyzed reaction is selective towards γ‐lactones, and all employed substates were terminal alkynes, showing thus no E/Z stereoselectivity.^[41]^ Nevertheless, for comparison, we have in the present work examined all three pathways for the cycloisomerization of substrate **1a** inside the **AuCav**.

Interestingly, although a large number of computational mechanistic studies have been previously reported on various gold‐catalyzed reactions,[^75^, ^76^, ^77^] only a couple have dealt with variations of the alkyne‐acid cycloisomerization.[^46^, ^78^]

Before discussing the mechanism, we first note here that the overall reaction energies for the cycloisomerizations of **1a** to yield γ‐lactone **2a** and δ‐lactone **3a** are calculated to be −22.7 kcal/mol and −17.2 kcal/mol, respectively.

**Scheme 2:**
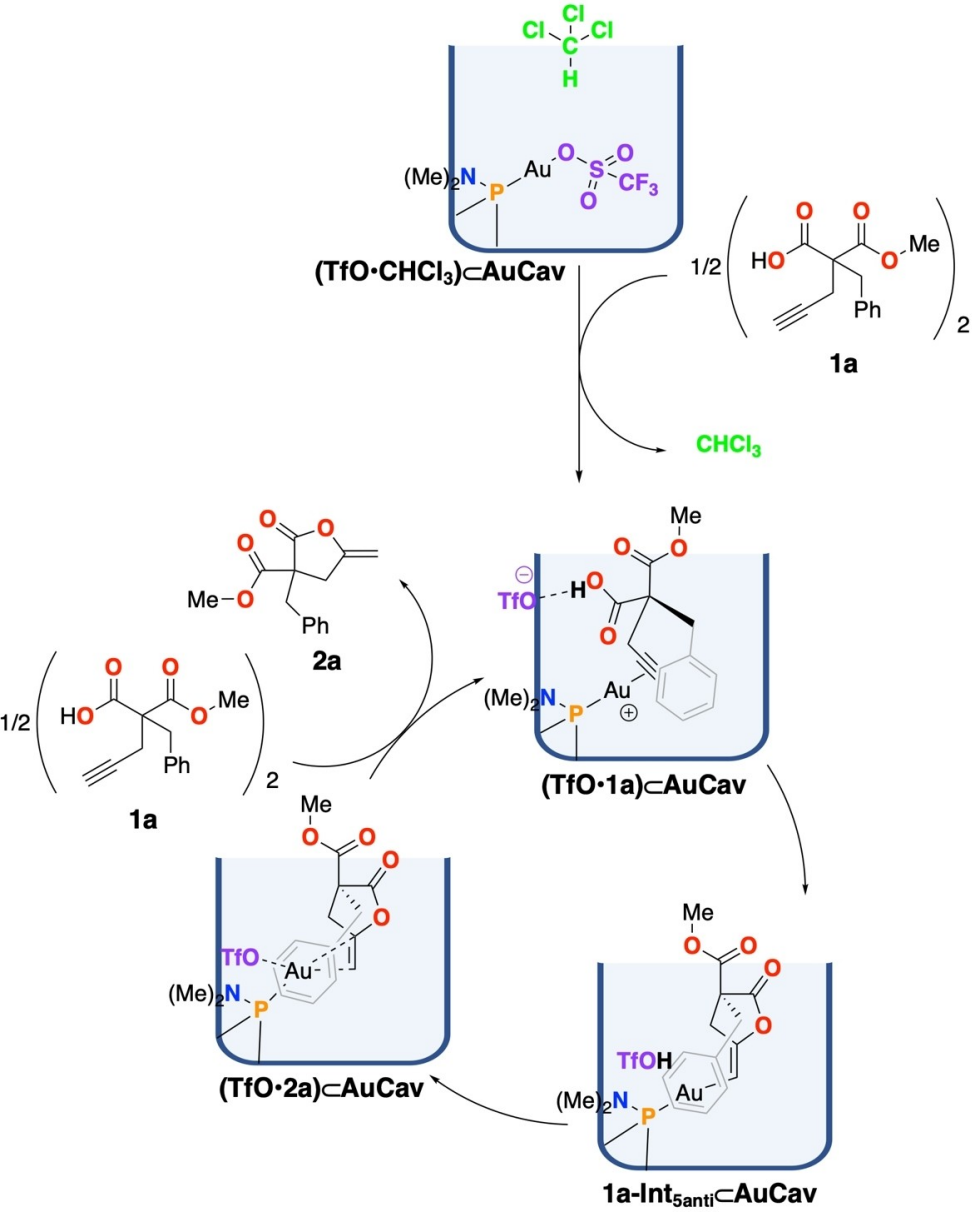
Catalytic cycle for the cycloisomerization of **1 a** in **AuCav** obtained on the basis of the present calculations.

The mechanism for the cycloisomerization of **1a** inside **AuCav** obtained on the basis of the present calculations is shown in Scheme 2. Starting from complex **(TfO**•**1a)⊂AuCav**, in which the alkyne triple bond is coordinated to the gold ion, the calculations show that the cyclization step takes place with a concomitant proton transfer from the carboxylic acid to the triflate counterion. Subsequently, the generated triflic acid protodeaurates the Au−C bond of the cyclized intermediate, generating the lactone product and regenerating the triflate anion. Finally, a guest exchange takes place, releasing the product from the cavitand and binding a new substrate molecule, completing thus the catalytic cycle.

**Figure 3:**
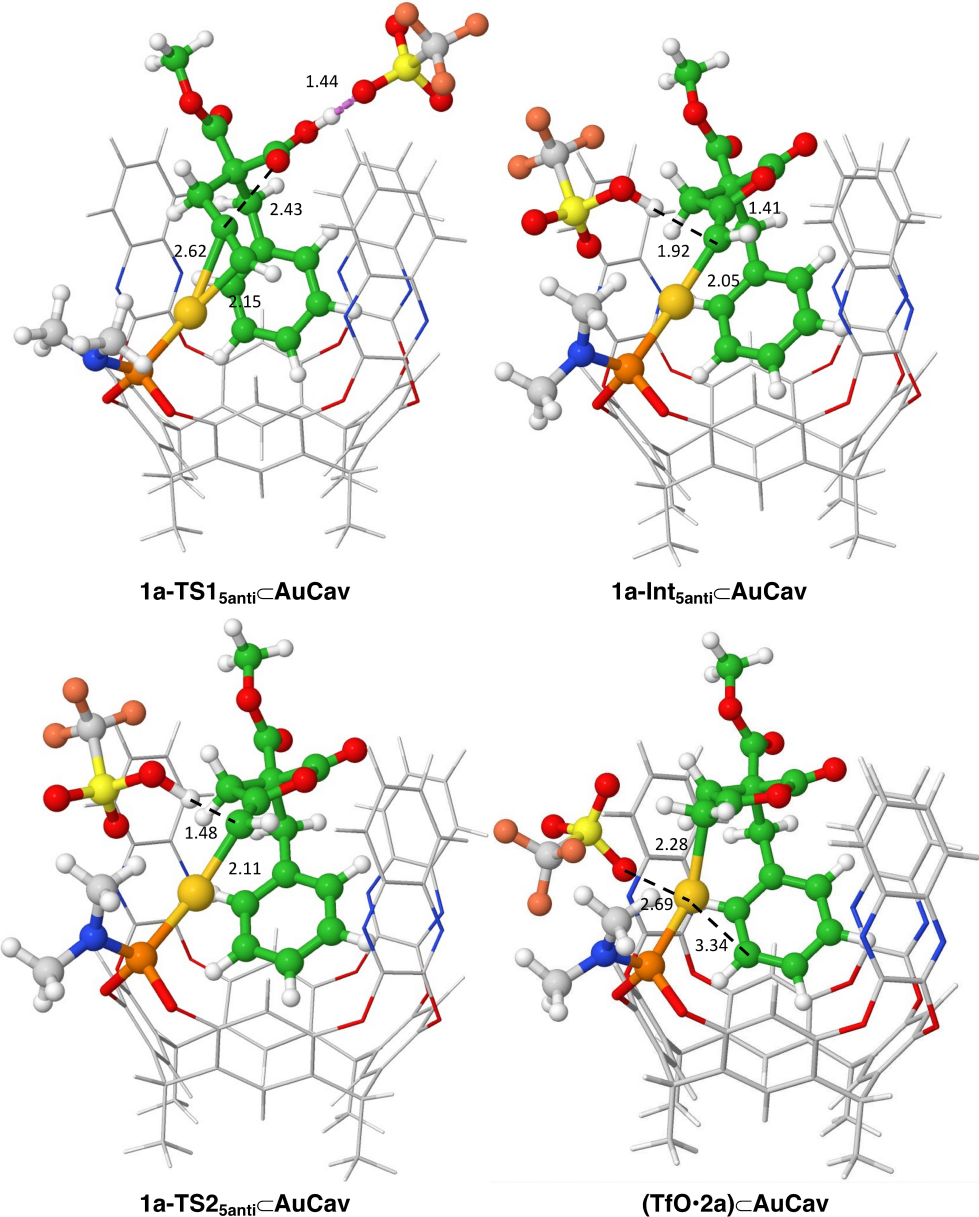
Optimized geometries of intermediates and transition states along the lowest‐energy reaction pathway for acetylenic acid **1 a**. Distances are in Å.

Interestingly, in all three cyclization pathways (5‐*anti*‐*exo*‐dig, 5‐*syn*‐*exo*‐dig and 6‐*endo*‐dig), the benzyl substituent of **1a** is found to point inside the cavitand throughout the entire reaction. The optimized geometries of the transition states (TSs) and intermediates for the 5‐*anti*‐*exo*‐dig cyclization pathway are displayed in Figure 3, while the geometries for the other pathways are given in the SI.

**Figure 4:**
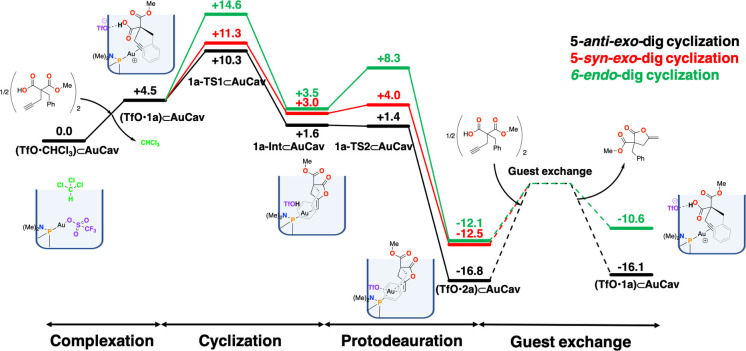
Calculated free energy profile (kcal/mol) of the cycloisomerization of **1 a** to its corresponding lactone inside the cavitand.

The calculations show that the cyclization/proton transfer step proceeds most favorably with a 5‐*anti*‐*exo*‐dig cyclization transition state **1a‐TS1_5anti_⊂AuCav**, with an overall barrier of 10.3 kcal/mol relative to **(TfO**•**CHCl_3_)⊂AuCav** (Figure 4). The 5‐*syn*‐addition via **1a‐TS1_5syn_⊂AuCav** has a slightly higher barrier of 11.3 kcal/mol, while the 6‐*endo*‐dig cyclization **1a‐TS1_6_⊂AuCav** has the highest barrier of 14.6 kcal/mol.

Cyclizations without the involvement of the triflate, i. e., starting from complex **1a⊂AuCav**, were calculated to have significantly higher barriers (see SI). For example, the barrier for the 5‐*anti*‐*exo*‐dig cyclization without triflate was found to be 17.6 kcal/mol, which is 7.3 kcal/mol higher than the concerted reaction involving the triflate.

The calculations further show that the 5‐*anti‐*cyclized intermediate **1a‐Int_5anti_⊂AuCav** is the lowest in energy among the cyclized intermediates, at +1.6 kcal/mol relative to **(TfO**•**CHCl_3_)⊂AuCav**, while the 5‐*syn*‐cyclized intermediate **1a‐Int_5syn_⊂AuCav** and the 6‐*endo*‐dig‐cyclized intermediate **1a‐Int_6_⊂AuCav** are at +3.0 and +3.5 kcal/mol, respectively (Figure 4).

Interestingly, in the lowest‐energy structure of **1a‐Int_5anti_⊂AuCav**, the OH group of the triflic acid interacts with the Au‐coordinated carbon rather than forming a hydrogen bond to any of the oxygen atoms of the intermediate (Figure 3). Removal of the triflic acid from **1a‐Int_5anti_⊂AuCav** leads to higher energy, by 4.2 kcal/mol (see SI).

From the cyclized intermediates, we have calculated the TSs for the protodeauration step by considering a proton transfer from the triflic acid to the carbon, generating the coordinated lactone products and regenerating the triflate counterion. The barrier for this process was found to be very low. In fact, in the case of 5‐*anti*‐*exo*‐dig, the calculated transition state (**1a‐TS2_5anti_⊂AuCav**) was found to be 0.2 kcal/mol lower than the preceding intermediate. This is of course a slight artifact of the computational procedure. In fact, at the level of theory of the geometry optimization, a barrier of 1.9 kcal/mol was obtained, which upon addition of various energy corrections (basis set, solvation, and Gibbs free energy) was lowered by 2.1 kcal/mol to become slightly negative. Nevertheless, the calculations show clearly that the protodeauration step for this pathway is very fast.

The protodeauration for the 5‐*syn*‐*exo*‐dig and 6‐*endo*‐dig pathways, on the other hand, are higher in energy (Figure 4), with **1a‐TS2_5syn_⊂AuCav** and **1a‐TS2_6_⊂AuCav** being 4.0 and 8.3 kcal/mol higher than the starting point, respectively.

The protodeauration steps lead to the lactone products bound to **AuCav**. In the case of the 5‐*anti*‐*exo*‐dig cyclization, **2a** coordinates to the gold ion through the terminal methylene carbon, as well as through a cation‐π interaction with the benzyl group. Moreover, the triflate interacts with the gold cation as well, with an Au−O distance of 2.69 Å (see Figure 3). The energy of this complex, called **(TfO**•**2a)⊂AuCav**, is calculated to be −16.8 kcal/mol relative to the starting point **(TfO**•**CHCl_3_)⊂AuCav**. The bound products of the 5‐*syn*‐cyclization, (**TfO**•**2a’)⊂AuCav**, and 6‐*endo*‐cyclization, (**TfO**•**3a)⊂AuCav**, are calculated to have higher energies, being −12.5 and −12.1 kcal/mol, relative to **(TfO**•**CHCl_3_)⊂AuCav**, respectively. Note that the 5‐*syn*‐*exo*‐dig and 5‐*anti*‐*exo*‐dig cyclization pathways lead to the same product, because **1a** is a terminal alkyne. Therefore, (**TfO**•**2a’)⊂AuCav** will interconvert to **(TfO**•**2a)⊂AuCav**, which has a lower energy. Different binding modes of product **2a**, and also product **3a**, to the cavitand were explored and the geometries and relative energies are provided in the SI.

The final step of the catalytic cycle is the exchange of the products with a new substrate molecule. This process is calculated to be slightly endergonic for both the γ‐ and δ‐lactones, by 0.7 and 1.5 kcal/mol, respectively, which is consistent with the fact that product inhibition was experimentally observed for the reaction of substrate **1a**. The barrier for the exchange step was not calculated explicitly in the current work as the employed computational methods are not suitable for this purpose.

The obtained energies for the overall reaction mechanism are summarized in Figure 4, from which a number of conclusions can be drawn. The selectivity‐determining step is found to be the concerted cyclization/proton transfer step (**1a‐TS1⊂AuCav**). The calculated difference in barriers between the formation of the γ‐ and δ‐lactones is 4.3 kcal/mol in favor of the former (10.3 vs. 14.6 kcal/mol), which is consistent with the exclusive formation of γ‐lactones observed by Ho and Schramm.^[41]^

Here, it should be noted that product **2a** is more stable than **3a** (Figure 4), showing that it is the preferred product both kinetically and thermodynamically. The energy difference between products **2a** and **3a** is 5.5 kcal/mol, which is very close to the difference between the rate‐determining TSs leading to them (i. e., 4.3 kcal/mol), indicating that the selectivity is inherent in the product structures. One explanation is the fact that lactone **2a** has a better stabilizing resonance with the *exo*‐methylene leading to a primary carbanion, in contrast to the corresponding resonance in **3a** which gives a less stable secondary carbanion.

Notably, however, the overall barrier for the conversion of reactant to product is calculated to be only 10.3 kcal/mol, which is too low compared to the overall barrier estimated from kinetics measurements. Namely, the rate constant was measured to be 5.59 ⋅ 10^−5^ s^−1^ at 55 °C,^[41]^ which can be converted to a free energy barrier of 25.6 kcal/mol.

One might envisage that the reason for this discrepancy is that the rate‐determining step (RDS) is not the cyclization/proton transfer step, but rather the aforementioned final guest exchange step that was not considered explicitly in the current study. However, this scenario can be ruled out, because if the exchange step were rate‐determining, adding product to the reaction mixture would not result in product inhibition, which is what is observed experimentally.^[41]^

Two other explanations for the underestimation of the barrier for the cyclization/proton transfer step can be envisioned. One is that the triflate might not be involved in the cyclization/proton transfer step. As discussed above, the barrier for this scenario is higher by ca 7 kcal/mol compared to when the triflate is directly involved in this step (17.6 vs. 10.3 kcal/mol), see SI. Another possibility is that there exists a lower‐energy reference point other than **(TfO**•**CHCl_3_)⊂AuCav** which we have not identified despite examining many possible host‐guest complexes. This scenario cannot be ruled out.

### 2.3 Influence of Ester Group Size

Next, we focus on substrate **1b**, which has a *tert*‐butyl substituent on the ester functionality (Scheme 1) and for which a slightly slower conversion was observed experimentally as compared to **1a**.^[41]^

**Figure 5:**
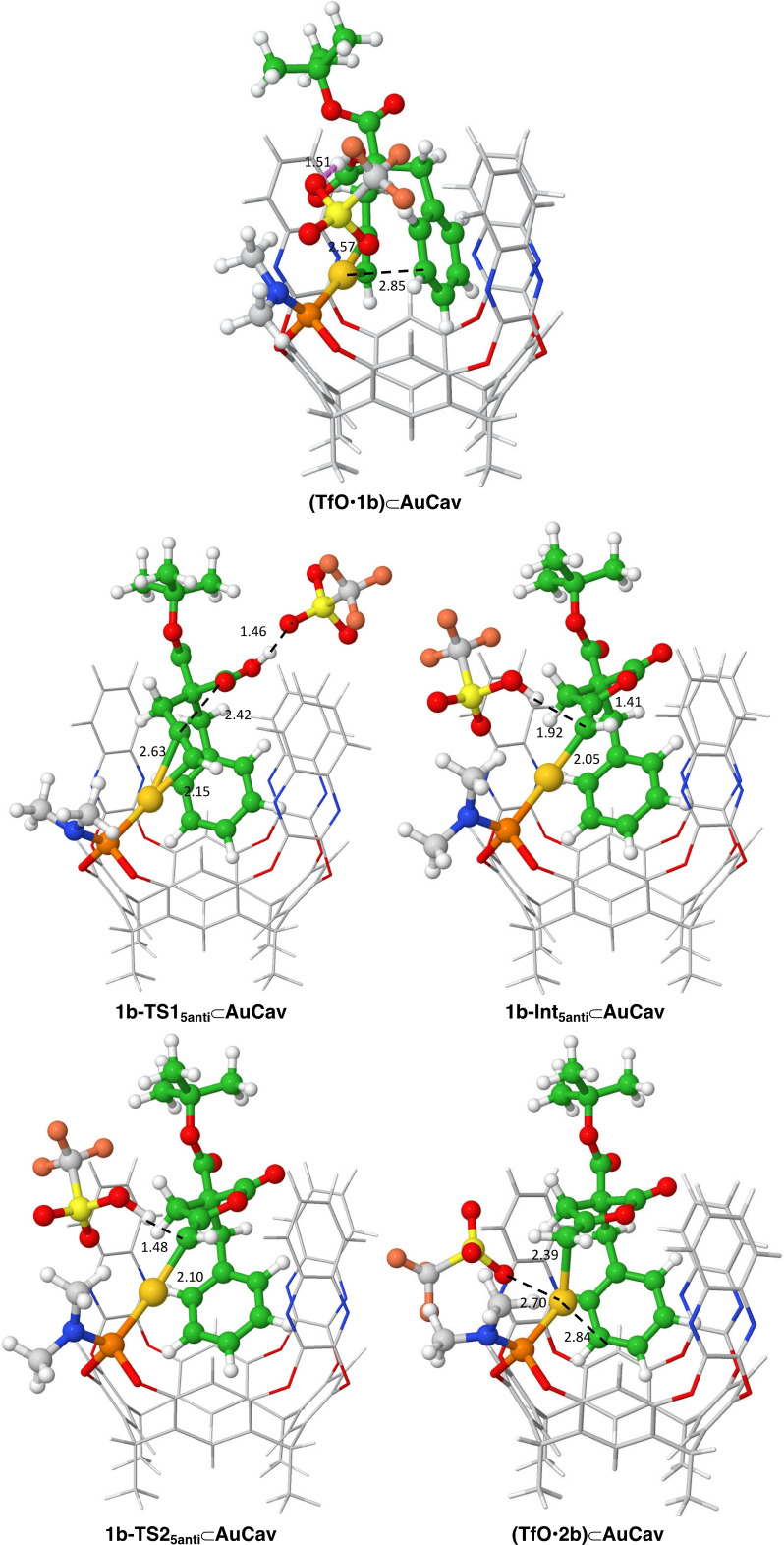
Optimized geometries for the lowest‐energy pathway for the reaction of **1 b**. Distances are given in Å.

**Figure 6:**
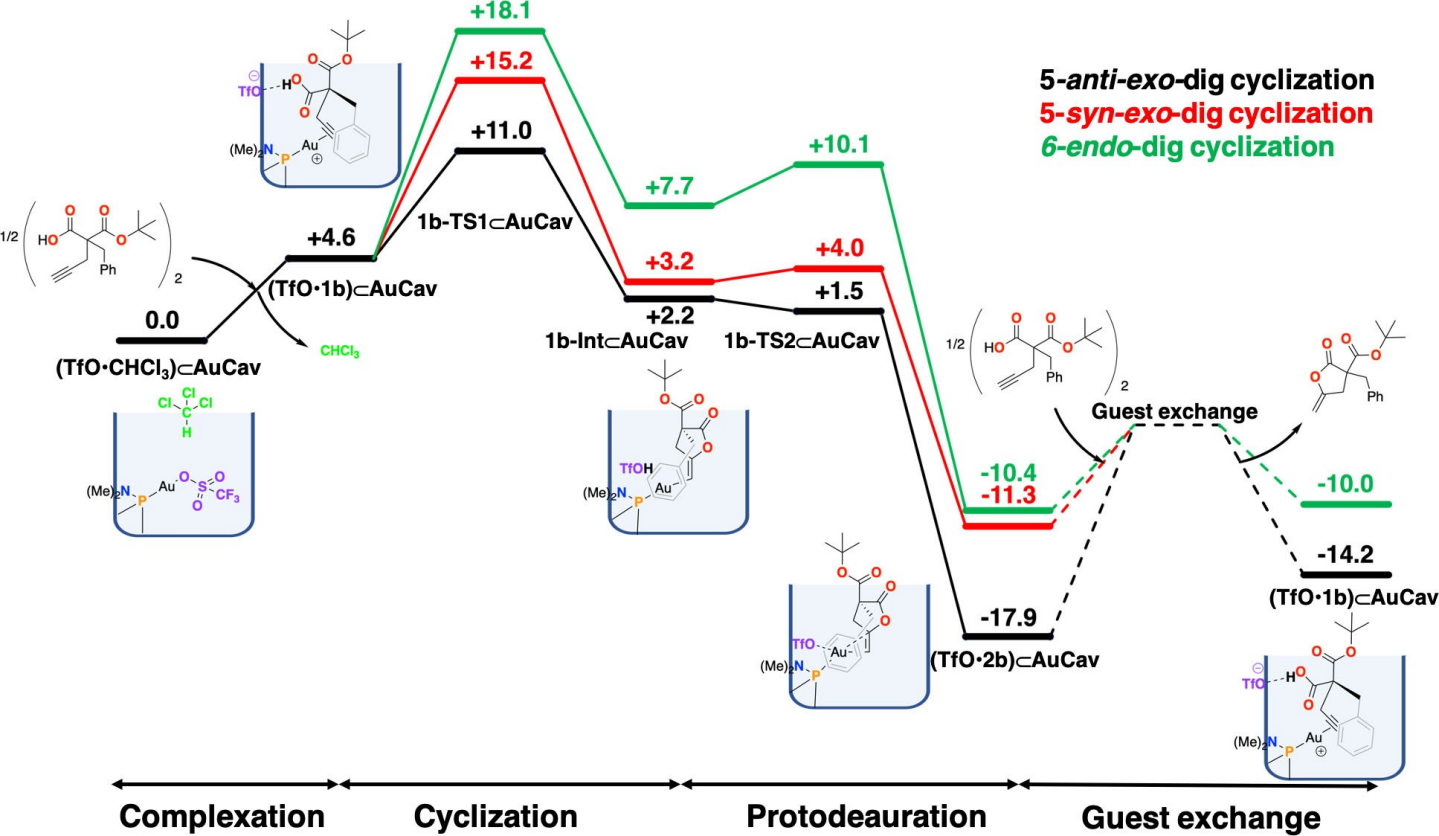
Calculated free energy profile of the cycloisomerization of **1 b**.

We performed a similar study as above, and the calculations show that the lowest‐energy binding mode, called (**TfO**•**1b)⊂AuCav**, is the same as for **1a**. Since the ester group points outside the cavitand (Figure 5), the *t*‐Bu substituent has no significant steric influence on the binding. Other binding modes with higher energies are given in the SI. The binding of **1b** to the cavitand was found to result in an energetic penalty of 4.6 kcal/mol (Figure 6), which is almost identical to substrate **1a**. The calculations further show that the reaction proceeds in an identical fashion as for substrate **1a**, with very similar geometries and energies (see SI for optimized geometries of the different TSs and intermediates along the reaction paths).

The lowest cyclization/proton transfer step was found for the 5*‐anti*‐*exo*‐dig pathway (**1b‐TS1_5anti_⊂AuCav**) with a barrier of 11.0 kcal/mol. The other pathways, **1b‐TS1_5syn_⊂AuCav** and **1b‐TS1_6_⊂AuCav**, were calculated to have significantly higher barriers, by 4.2 and 7.1 kcal/mol, respectively. Again, these results are consistent with the exclusive formation of γ‐lactone observed for substrate **1b**. The energy differences are larger than for **1a**, because in the TSs for the 5‐*syn*‐*exo*‐dig and the 6‐*endo*‐dig pathways, the bulky *t*‐Bu substituent is closer to the rim of the cavitand (see SI). However, as in the case for **1a**, the overall barrier calculated for the process is too low compared to the reaction time of the experiments. The same potential reasons as above could be invoked here. We have also for the reaction of guest **1b** considered the scenario in which the triflate does not participate in the cyclization/proton transfer step, and the barrier was calculated to be also ca 7 kcal/mol higher than when the triflate is directly involved (18.4 vs. 11.0 kcal/mol), see SI.

### 2.4 Influence of Side Chain Group Size

To investigate the influence of the size of the side chain on the binding and reactivity, we now consider substrate **1c**, with a 1‐naphthyl substituent. Experimentally, this substrate was found to react somewhat faster than **1a**, with the same selectivity towards γ‐lactones.^[41]^

**Figure 7:**
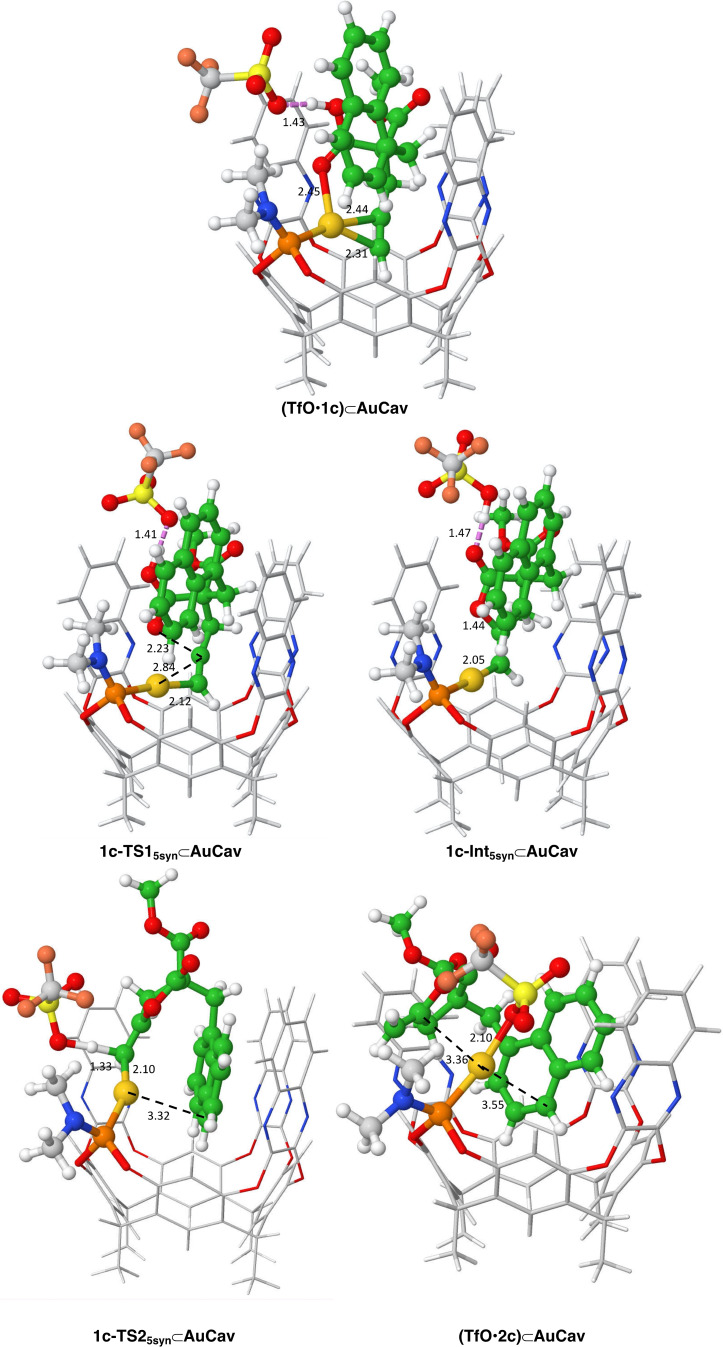
Optimized geometries for the lowest‐energy pathway for the reaction of **1 c**.

**Figure 8:**
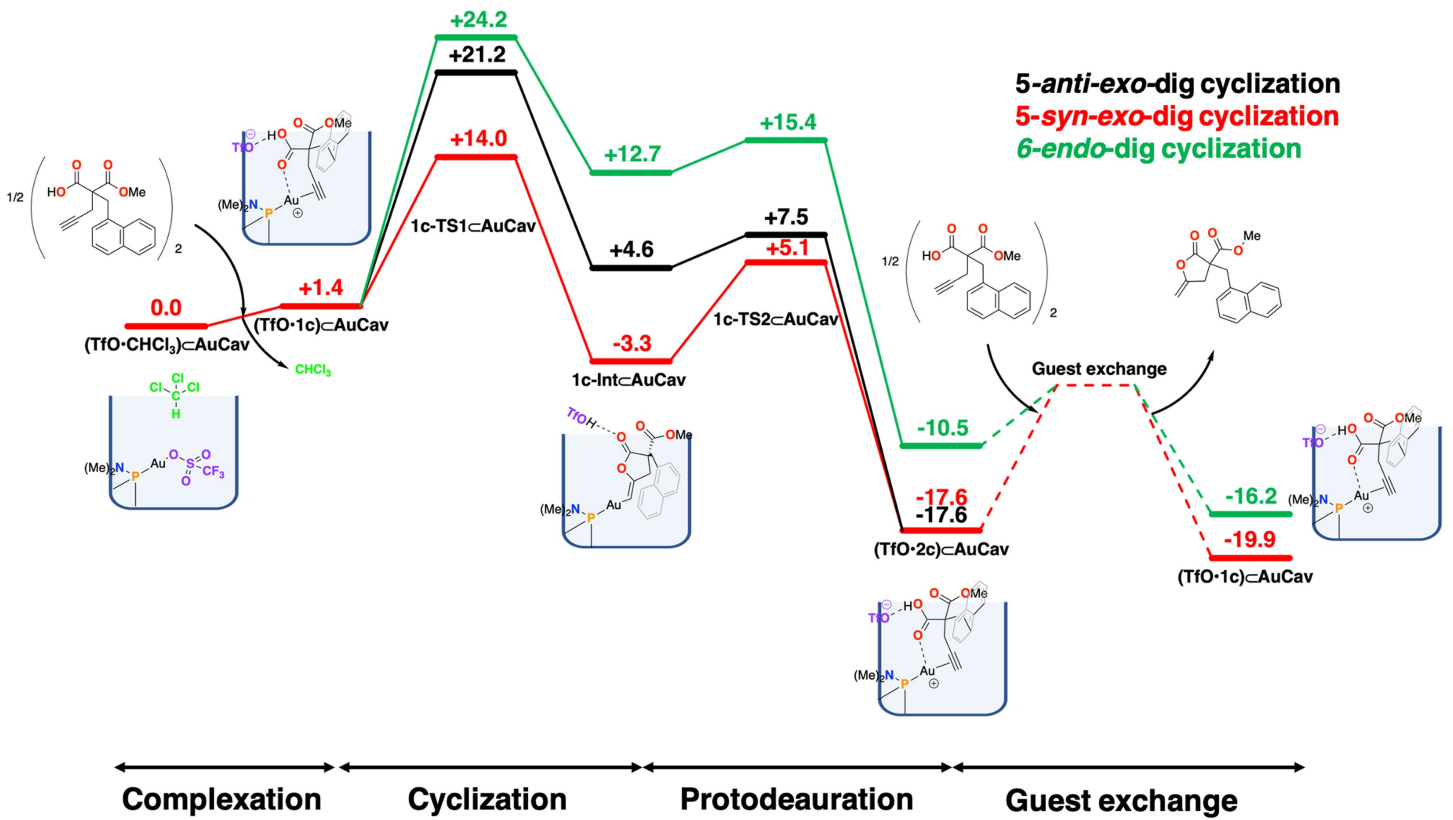
Calculated free energy profile of the cycloisomerization of **1 c**.

We considered different binding modes for complex (**TfO**•**1c)⊂AuCav** and found that, although the naphthyl group in analogy to the benzyl in **1a** and **1b** can fit inside the cavitand, this conformation is associated with higher energy (see SI). Instead, in the lowest‐energy binding mode, the naphthyl is located in the area between the gold atom and one of the cavitand walls, pointing upward and protruding out from the cavitand rim (Figure 7). The carbonyl oxygen of the carboxylic acid coordinates to the gold with a distance of 2.45 Å. The ester group points upward, just as in the case for **1a** and **1b**, but is still fully located inside the cavitand. For substrate **1c**, the combined monomerization and binding was calculated to be associated with an energetic penalty of 1.4 kcal/mol (Figure 8).

Unlike **1a** and **1b**, the lowest‐energy pathway proceeds through the 5‐*syn*‐*exo*‐dig cyclization, with an overall barrier of 14.0 kcal/mol for **1c‐TS1_5syn_⊂AuCav**, compared to 21.2 and 24.2 kcal/mol for **1c‐TS1_5anti_⊂AuCav** and **1c‐TS1_6_⊂AuCav**, respectively (see Figure 8).

The reason for the 5‐*anti*‐*exo*‐dig and the 6‐*endo*‐dig having significantly higher barriers is that the naphthyl group points inside the cavitand, requiring a distortion of the cavitand to accommodate this. The optimized geometries of the different transition states and intermediates for the different reaction pathways are presented in the SI. The preference of the 5‐*syn*‐*exo*‐dig pathway is maintained for the rest of the reaction (Figure 8). Importantly, since the alkyne in **1c** is terminal, there is no difference between the products obtained from the 5‐*syn*‐*exo*‐dig and 5‐*anti*‐*exo*‐dig cyclizations, and the calculations are thus consistent with the results of the experiments.

Finally, unlike substrate **1a** and **1b**, the product exchange step for substrate **1c** is calculated to be exergonic, by 2.3 kcal/mol (Figure 8). Here, it should be noted again that the overall barrier for the process is greatly underestimated, for the same possible reasons discussed above for substrates **1a** and **1b**. For the reaction of guest **1c**, we have also considered the scenario in which the triflate does not participate in the cyclization/proton transfer step, and the barrier was calculated to be ca 3 kcal/mol higher than when the triflate is directly involved (16.7 vs. 14.0 kcal/mol), see SI.

Very interestingly, comparison of the rate‐determining barriers for guests **1c** and **1a** shows that the barrier for **1c** is higher than for **1a** when triflate is involved (14.0 vs. 10.3 kcal/mol), whereas the opposite is true when the triflate is not involved in that step (16.7 vs. 17.6 kcal/mol). The latter is more consistent with the experimental kinetics, which showed that **1c** reacts slightly faster than **1a**. These results, in conjunction with the fact that the obtained absolute barriers are greatly underestimated for all guests when triflate is involved in the RDS, speak in favor of the scenario in which triflate is in fact not involved in this step.

### 2.5 Influence of the Cavitand Walls

**Figure 9:**
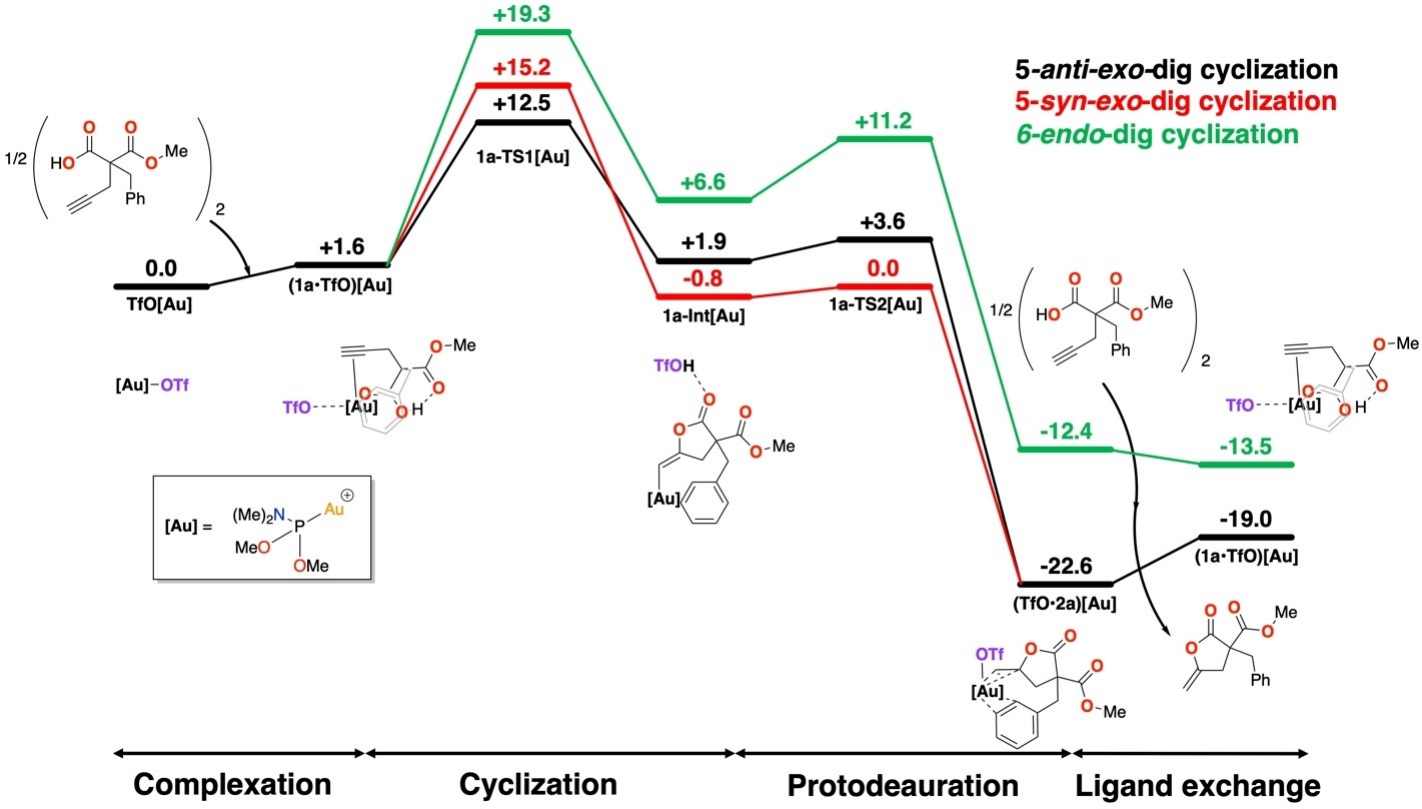
Calculated free energy profile showing the cycloisomerization of **1 a** with catalyst **[Au]**.

It is interesting to compare the energies obtained above for the reactions in the cavitand to the same reaction in the absence of **AuCav** to evaluate its influence on the reactivity and selectivity. To this end, we focused on substrate **1a** and removed the cavitand to keep only the dimethyl dimethylphosphoramidite ligand, (OCH_3_)_2_PN(CH_3_)_2_. In what follows, this catalyst will be referred to simply as **[Au]**, and the calculated energies obtained for this model are displayed in Figure 9, while the optimized geometries are given in the SI.

The calculations show that, although the penalty for binding substrate **1a** is 2.9 kcal/mol lower without the cavitand (1.6 vs. 4.5 kcal/mol), the overall barrier of the cyclization/proton transfer is 2.2 kcal/mol higher than inside the cavitand (12.5 vs 10.3 kcal/mol). The cavitand walls provide thus a lowering of the overall energy barrier, but the effect is found to be rather small.

It is informative here to compare the calculated barriers for the chemical step of the reactions, i. e., starting from the bound complexes, with and without the cavitand. Namely, the energy of transition state **1a‐TS1_5anti_⊂AuCav** relative to **(TfO**•**1a)⊂AuCav** is 5.8 kcal/mol, to be compared to 10.9 kcal/mol, which is the energy of transition state **1a‐TS1_5anti_[Au]** relative to **(TfO**•**1a)[Au]**. Thus, whereas the cavitand walls cause a greater penalty for binding the substate, they provide a significant stabilization of the transition state. The same stabilization trend is true for the energies of the cyclized intermediates **1a‐Int_5anti_⊂AuCav** and **1a‐Int_5anti_[Au]** relative to their bound complexes.

Regarding the selectivity of the reaction, both the catalysts, with and without the walls, display preference towards γ‐lactones. The energy difference without the walls, between **1a‐TS1_5anti_[Au]** and **1a‐TS1_6_[Au]**, is 6.8 kcal/mol, to be compared to the cavitand reaction where the difference is somewhat smaller, 4.3 kcal/mol. Similarly, the energy difference between the *anti‐* and *syn‐*cyclization transition states **1a‐TS1_5anti_[Au]** and **1a‐TS1_5syn_[Au]** is calculated to be 2.7 kcal/mol, which is somewhat larger than the 1.0 kcal/mol difference obtained for the cavitand counterparts.

Finally, we also examined how the involvement of the triflate in the cyclization/proton transfer step affects the barrier. It was found that without the triflate the barrier increases only by 1.7 kcal/mol, from 12.5 to 14.2 kcal/mol (see SI), to be compared to a difference of 7.3 kcal/mol in the presence of the cavitand (see above).

## 3 Conclusions

We have in the present paper used density functional theory methodology to investigate the cycloisomerization of acetylenic acids taking place in a gold‐functionalized resorcinarene‐based cavitand to yield corresponding lactones. Three substrates with different substitution patterns (**1a–c**, Scheme 1) were considered by the calculations, and for each of them, a comprehensive binding analysis was first conducted to identify the lowest‐energy binding mode.

The calculations suggest the reaction mechanism shown in Scheme 2, involving the following steps: 1) binding of guest acetylenic acid by coordination of the triple bond to the gold ion; 2) a cyclization step occurring concertedly with a proton transfer from the carboxylic acid to the triflate counterion, 3) a protodeauration of the Au−C bond by proton transfer from triflic acid; and finally, 4) a guest exchange step, releasing the lactone product and binding a new substrate.

The cyclization/proton transfer step was found to be both rate‐ and selectivity‐determining for the reaction, and the selectivity was well reproduced by the calculations, namely that γ‐lactones are formed exclusively for all three substates. However, for all three substrates, we found the overall barrier to be too low to match the experimentally measured rate constants. We have speculated that the reason for the underestimation could be that the concerted cyclization/proton transfer step is in fact stepwise, and the triflate is not involved in the cyclization step. This scenario leads to higher barriers, and the energies would also be in line with the fact that substrate **1c** with a 1‐naphthyl substituent is slightly faster that substrate **1a**, with a benzyl substituent. Another possible explanation for the low barriers could be that there exists a low‐energy reference point which was not identified by the calculations.

Finally, the influence of the cavitand on the energies of the reaction was analyzed by calculating the cycloisomerization of substrate **1a** using a stripped‐down model that consists only of the gold ion and a dimethyl dimethylphosphoramidite ligand. The calculations show that the energetic penalty for binding the substrate is higher in the cavitand compared to without it, but the overall barrier of the cyclization/proton transfer somewhat lower in the cavitand.

## Conflict of Interests

The authors declare no conflict of interest.

4

## Supporting information

As a service to our authors and readers, this journal provides supporting information supplied by the authors. Such materials are peer reviewed and may be re‐organized for online delivery, but are not copy‐edited or typeset. Technical support issues arising from supporting information (other than missing files) should be addressed to the authors.

Supporting Information

## Data Availability

The data that support the findings of this study are available in the supplementary material of this article.
