# Supplementary material for: Computational Study of Alkyne‐Acid Cycloisomerization in Gold‐Functionalized Resorcinarene Cavitand
Source: Chemistry. 2025 Mar 10;31(20):e202404480. doi: 10.1002/chem.202404480 (PMC11973874; doi:10.1002/chem.202404480)
Supplement: Supplementary file 1 — Supporting Information [file CHEM-31-e202404480-s001.pdf]

Chemistry–A European Journal

Supporting Information

Computational Study of Alkyne-Acid Cycloisomerization in Gold-Functionalized Resorcinarene Cavitand

Joannes Peters and Fahmi Himo*

Supporting Information

Computational Study of Alkyne-Acid Cycloisomerization in Gold-Functionalized Resorcinarene Cavitand

Joannes Peters and Fahmi Himo

Department of Chemistry
Arrhenius Laboratory
Stockholm University
SE-10691 Stockholm
Sweden.

Table of Contents

I. Effect of geometry optimizations in solution S3
II. Effect of truncation of cavitand feet S4
III. Activation of **AuClCav** S5
IV. Cavitand-guest structures and energies S6
V. Substrate dimerization energies S7
VI. Additional results for reaction of **1a** in **AuCav** S8
VII. Additional results for reaction of **1b** in **AuCav** S17
VIII. Additional results for reaction of **1c** in **AuCav** S24
IX. Cycloisomerization of **1a** by **[Au]** S31
X. Absolute energies and energy corrections S36
XI. Cartesian coordinates S42

I. Effect of geometry optimizations in solution

To examine the influence of optimizing the geometries in the gas phase, the stationary points for the 5-*anti*-*exo*-dig pathway of the reaction of **1a** with model catalyst **[Au]** (see Section 2.5. in the main text) were re-optimized in solution and the energy profile was recalculated on the basis of these geometries. As seen from Figure S1, the effect of this procedure on the energies is small, less that 2 kcal/mol, and does not impact the conclusions.

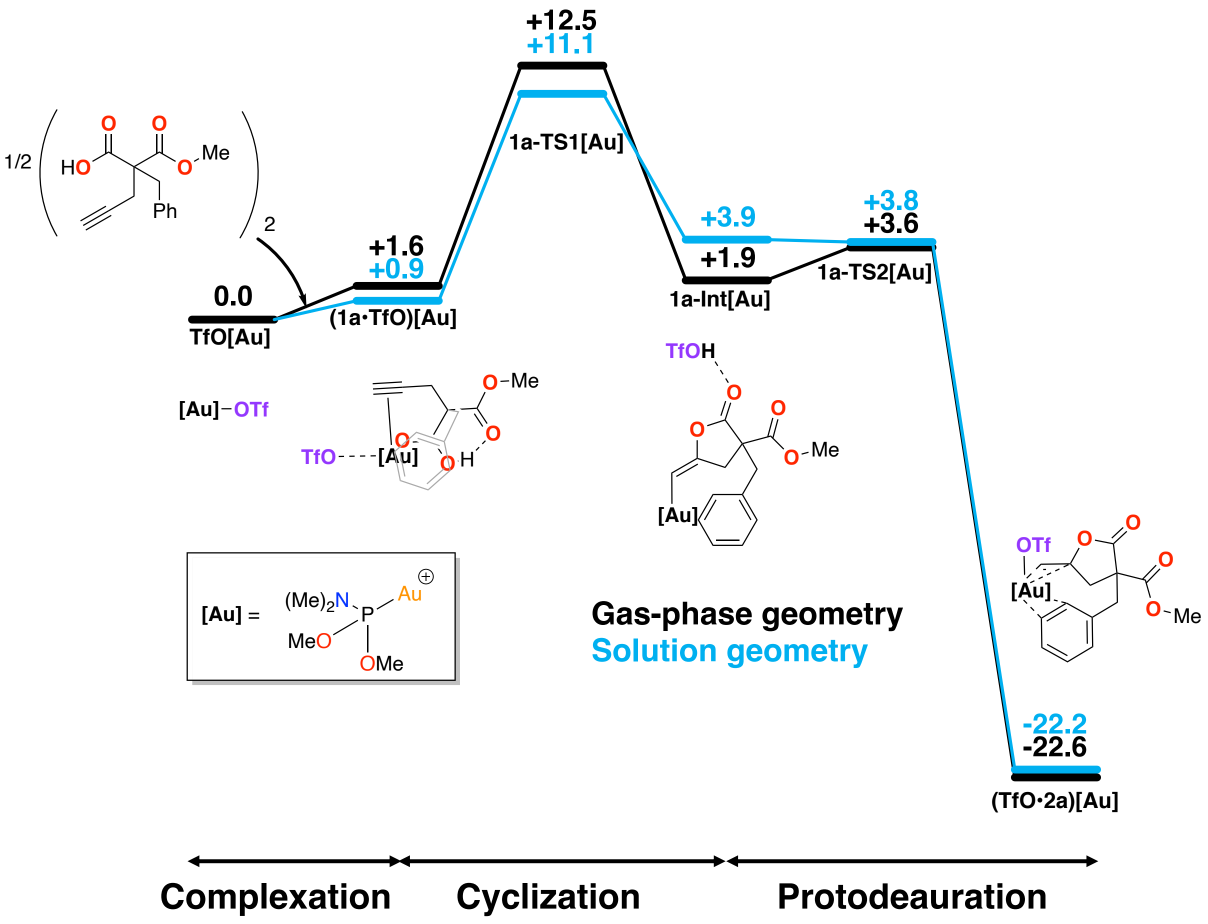

Figure S1. *Comparison of calculated free energy profiles for the cyclization of* ***1a*** *by* ***[Au]*** *by optimizing the geometries in solution (blue) and in gas phase (black). Energies are in kcal/mol.*

II. Effect of truncation of cavitand feet

As discussed in the main text, the influence of the cavitand feet on the geometry of the cavitand was examined. A geometry optimization of complex **(TfO•CHCl$_3$)⊂AuCav** with the full feet ($C_{11}H_{23}$) included was performed, and a superposition of the resulting geometry (red) and the geometry of the same complex with truncated feet (blue) is shown in Figure S2.

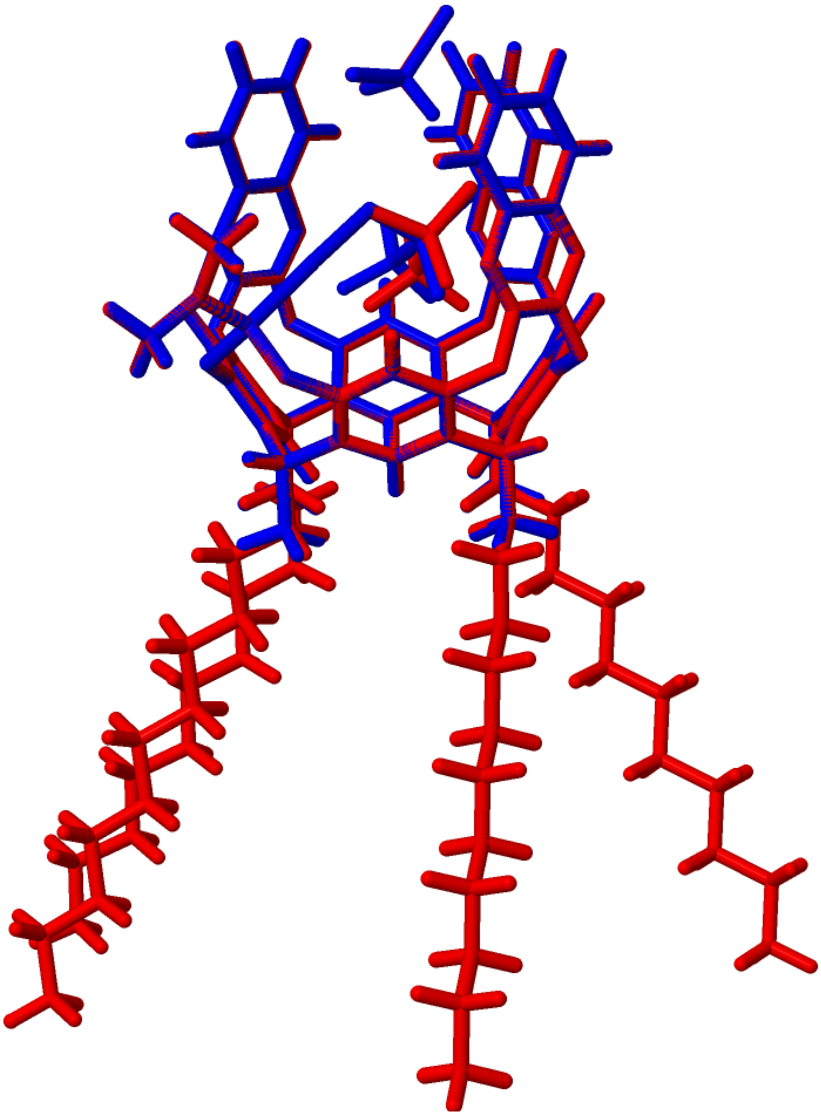

Figure S2. *Superposition of geometries of complex* ***(TfO•CHCl$_3$)⊂AuCav*** *with the feet fully modelled ($C_{11}H_{23}$, red) and with the feet truncated (Me, blue).*

III. Activation of AuClCav

The equilibrium for the activation of the catalyst:

(CHCl$_3$)⊂AuClCav + AgOTf ⇌ **(TfO·CHCl$_3$)⊂AuCav** + AgCl

was calculated to be exergonic by 6.9 kcal/mol. The optimized structures are given in Figure S3.

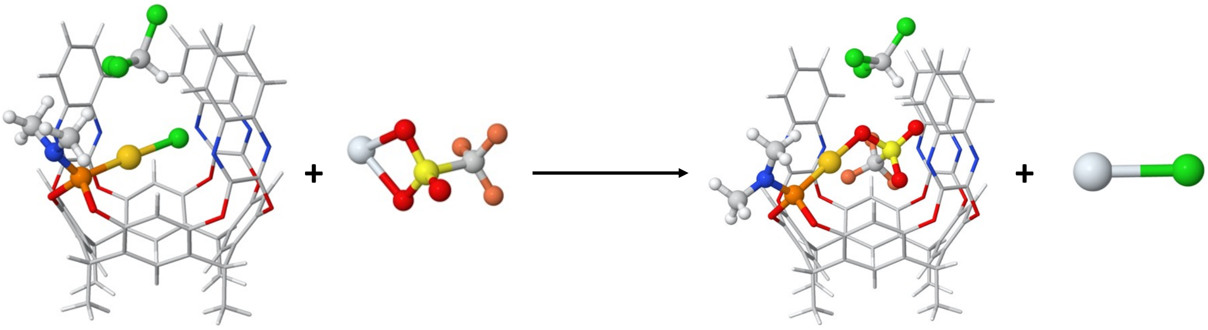

(CHCl$_3$)⊂AuClCav **AgOTf** **(TfO·CHCl$_3$)⊂AuCav** **AgCl**

Figure S3. *Geometries for the activation of* ***(CHCl$_3$)⊂AuClCav*** *by AgOTf.*

IV. Cavitand-guest structures and energies

As mentioned in the main text, the cavitand **AuCav** can bind different guests in the absence of the acetylenic acid substrate. We have considered a number of complexes with the chloroform solvent and the triflate counterion. The optimized geometries and relative free energies are given in Figure S4.

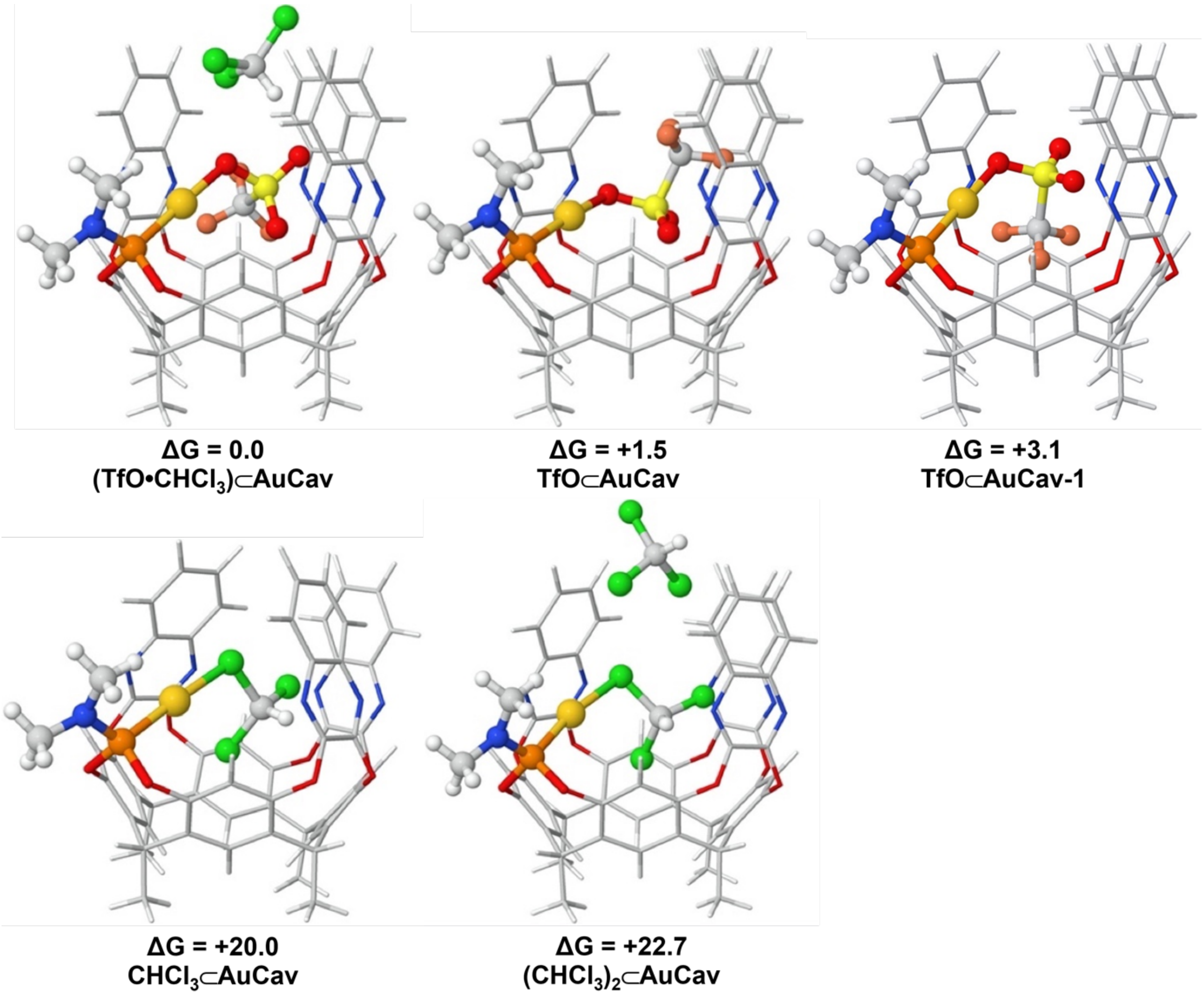

Figure S4. *Optimized geometries and relative free energies of possible guests for* ***AuCav****. Relative energies are in kcal/mol.*

V. Substrate dimerization energies

The acetylenic acid substrates **1a-c** can form dimers through complementary hydrogen bonds between the carboxylic moieties. The energy gains are given in Figure S5, and the optimized geometries of the monomers and dimers are given in Figure S6.

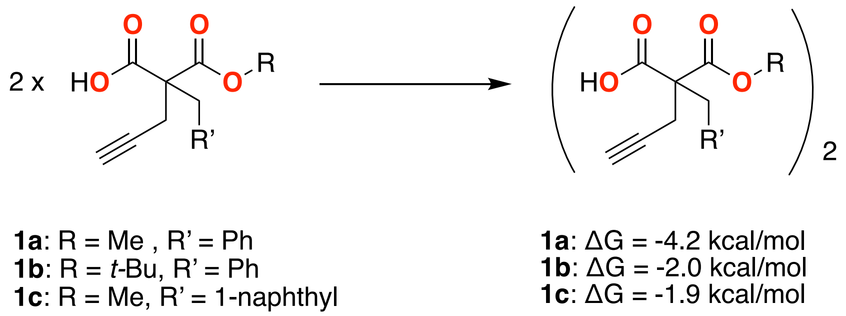

Figure S5. *Dimerization energies of acetylenic acid substrates* ***1a-c****.*

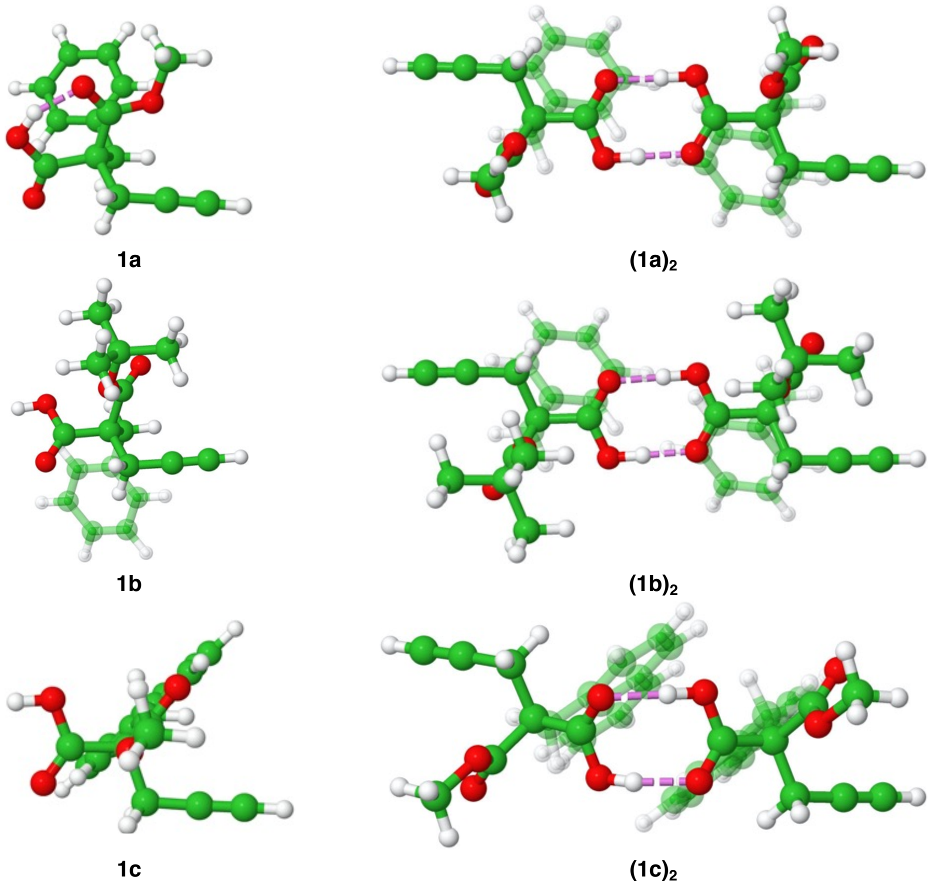

Figure S6. *Optimized geometries of acetylenic acid substrates* ***1a-c*** *and their dimers.*

VI. Additional results for reaction of 1a in AuCav

VI.A. Binding modes of 1a to AuCav

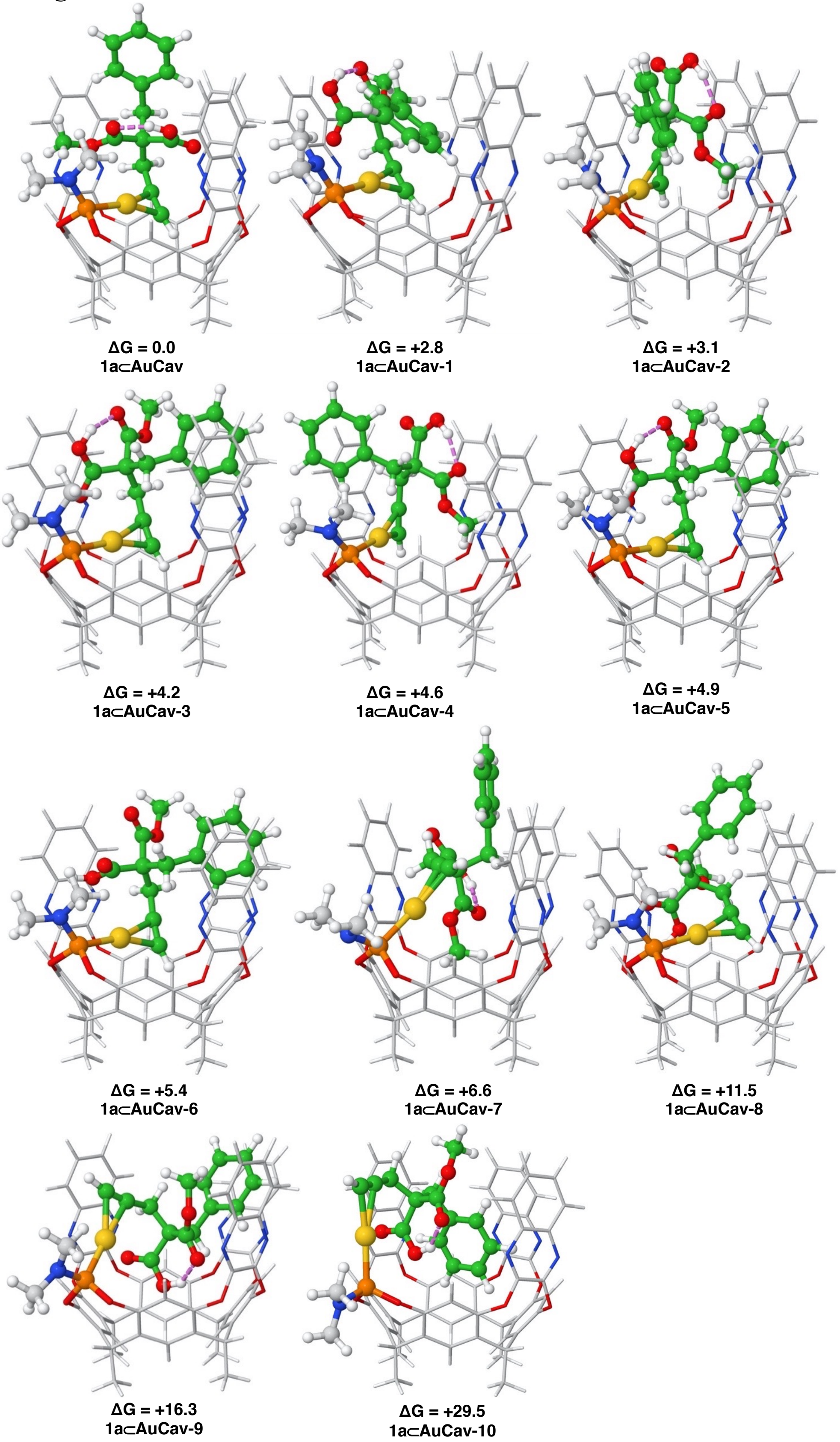

Figure S7. *Alternative binding modes for* ***1a⊂AuCav****. Relative energies are in kcal/mol.*

VI.B. Alternative binding modes for (TfO•1a)⊂AuCav

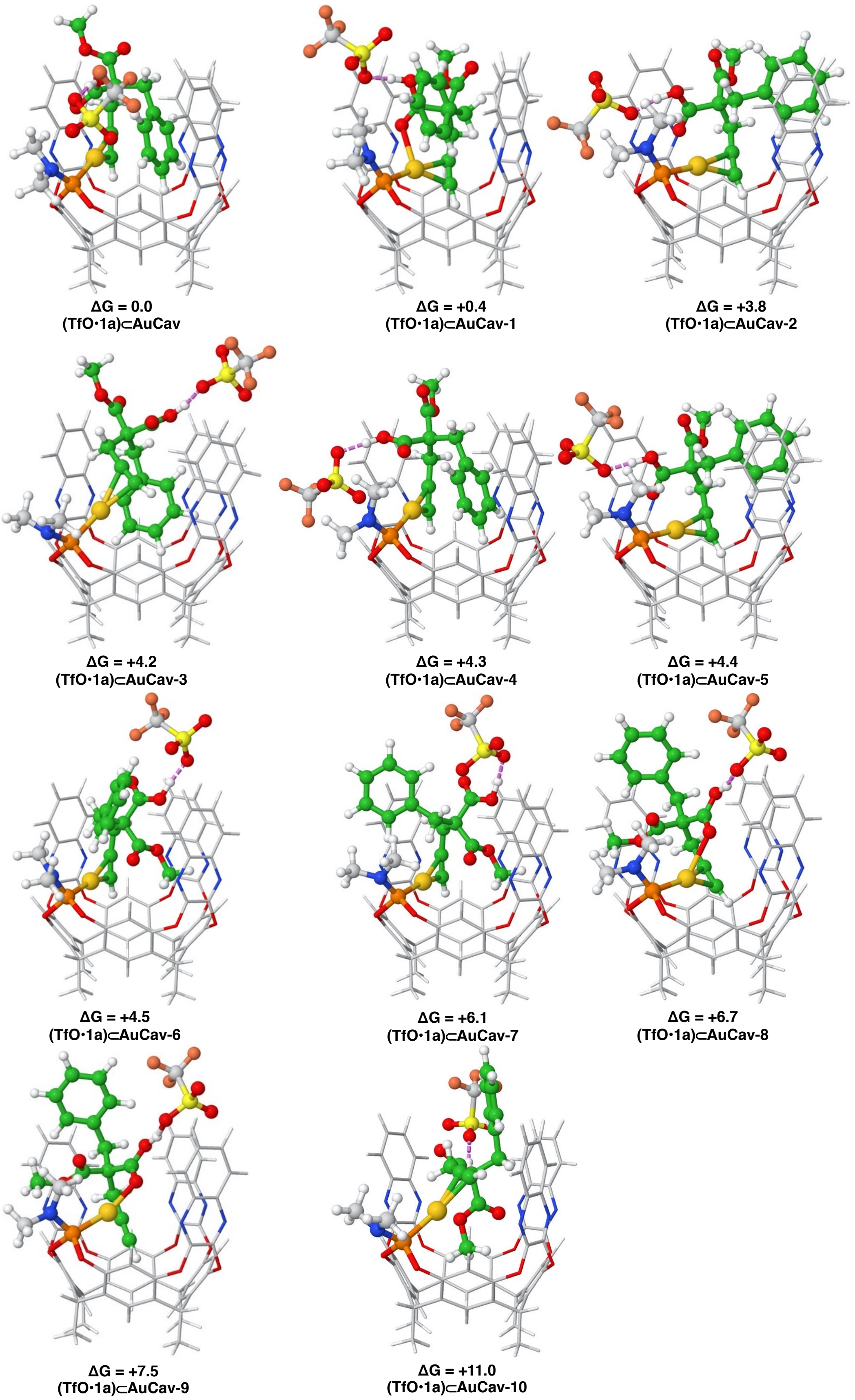

Figure S8. *Alternative binding modes for **(TfO•1a)⊂AuCav**. Relative energies are in kcal/mol.*

VI.C. 5-*syn*-*exo*-dig cyclization pathway

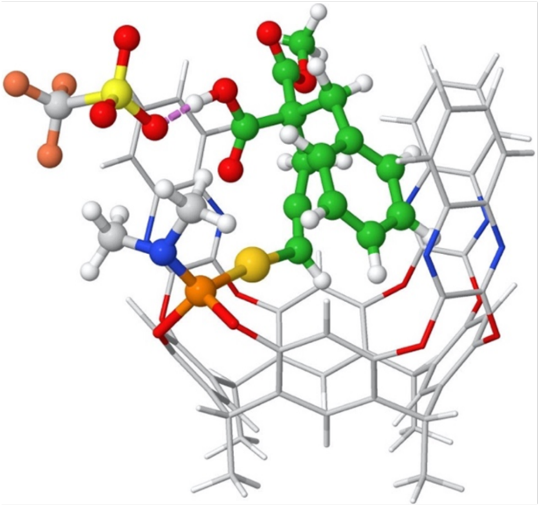

$\Delta G^{\ddagger}$ = +11.3
1a-TS1$_{5syn}$$\subset$AuCav

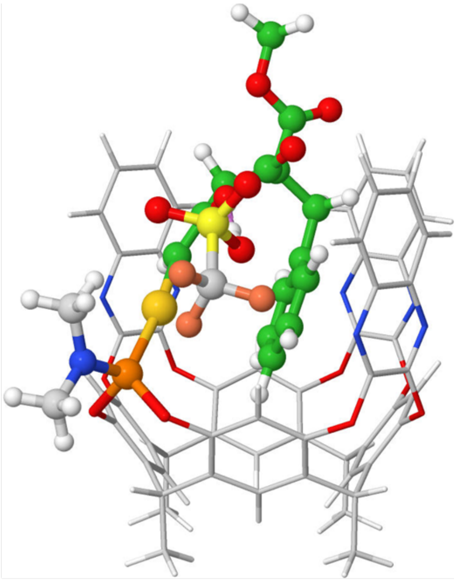

ΔG = +3.0
1a-Int$_{5syn}$$\subset$AuCav

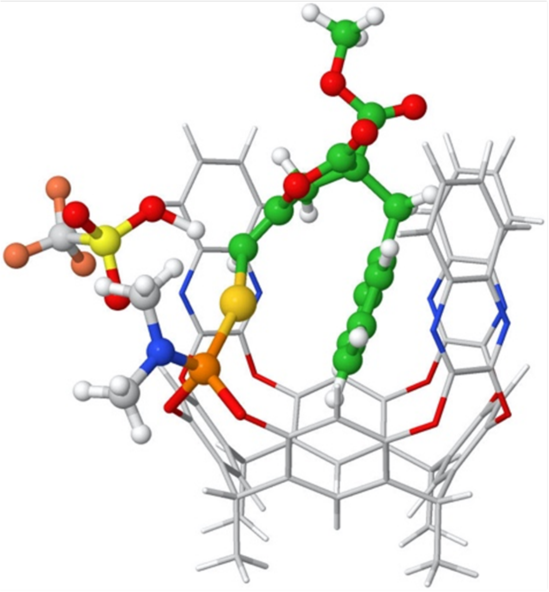

$\Delta G^{\ddagger}$ = +4.0
1a-TS2$_{5syn}$$\subset$AuCav

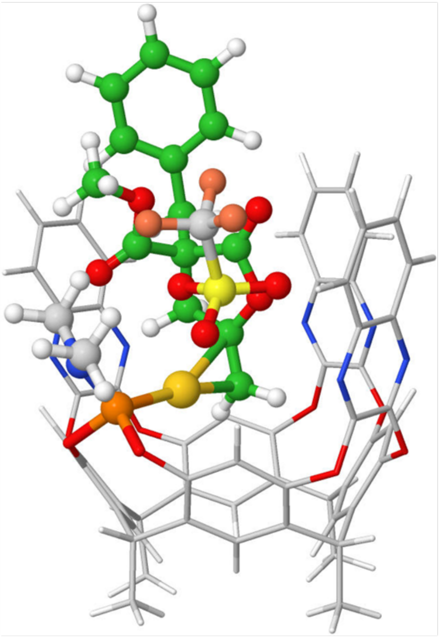

ΔG = -12.5
(TfO•2a')$\subset$AuCav

Figure S9. *Optimized geometries for the cycloisomerization of* ***1a*** *through 5-syn-exo-dig cyclization. Energies are in kcal/mol relative to* ***(TfO•CHCl$_3$)$\subset$AuCav***.

VI.D. 6-*endo*-dig cyclization pathway

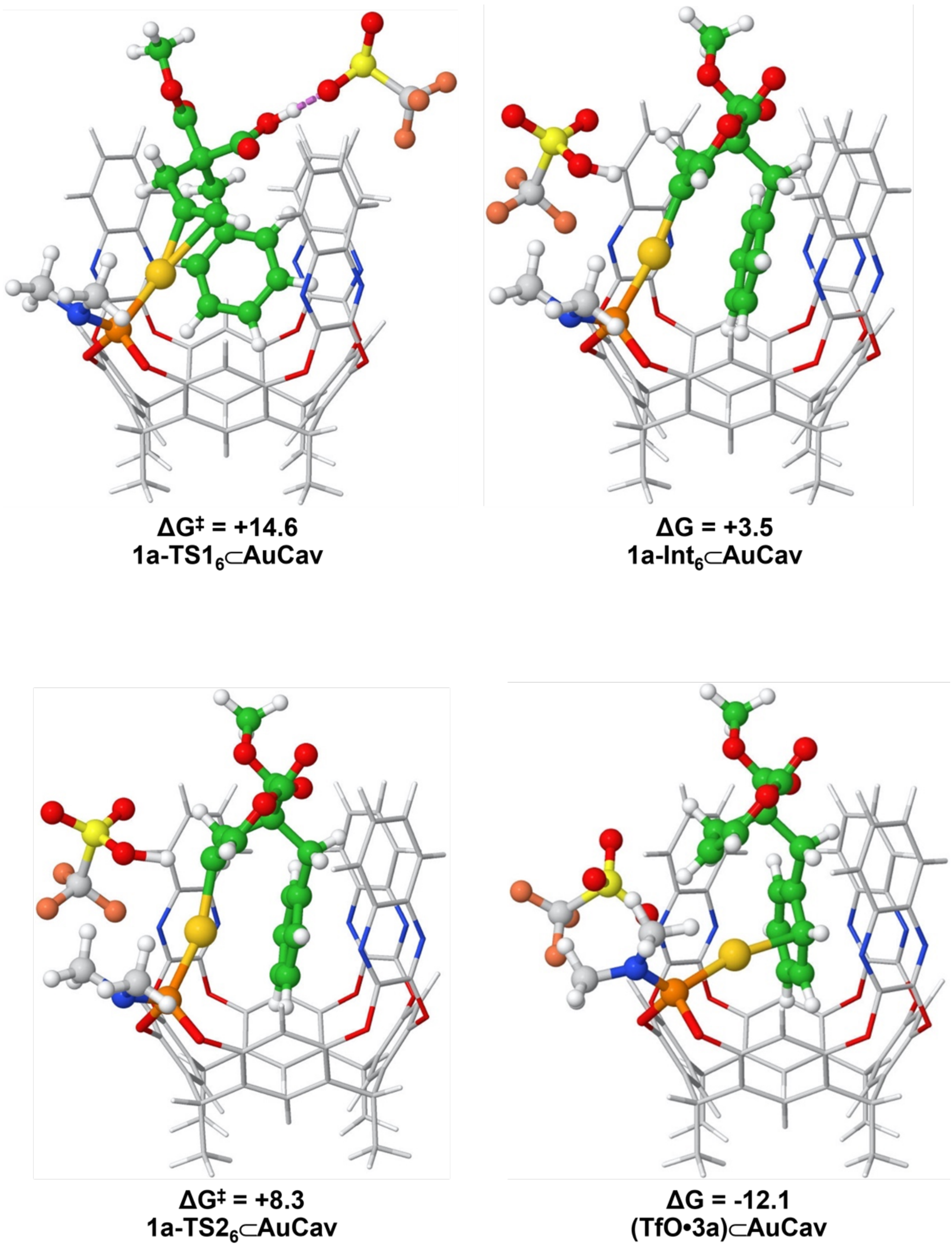

Figure S10. *Optimized geometries for the cycloisomerization of* ***1a*** *through 6-endo-dig cyclization. Energies are in kcal/mol relative to* ***(TfO•CHCl$_3$)⊂AuCav****.*

VI.E. Cyclization of 1a in AuCav without triflate

As discussed in the main text, we have also considered the cyclization process taking place without the participation of triflate as a proton acceptor. The calculated energies for the three pathways are given in Figure S11 and the optimized geometries are given in Figure S12.

To proceed from **1a-IntH$^+$⊂AuCav**, a proton transfer is required to the triflate counterion. Despite many attempts, no transition state could be located for this step, as the proton transfer occurred spontaneously in the geometry optimization, leading to structure **1a-Int⊂AuCav**.

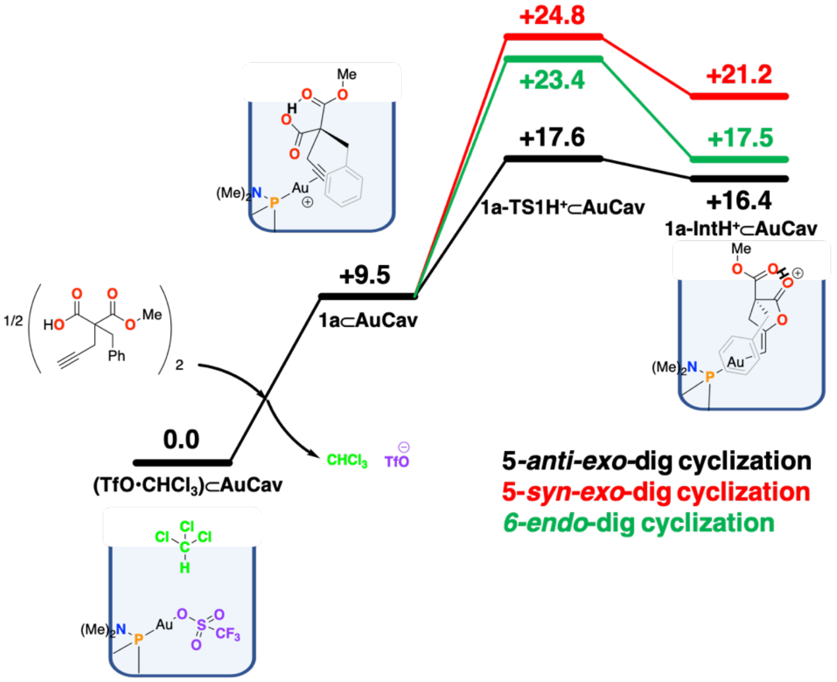

Figure S11. *Calculated free energy profile for the cycloisomerization of* ***1a*** *in the absence of triflate during the cyclization. Energies are in kcal/mol.*

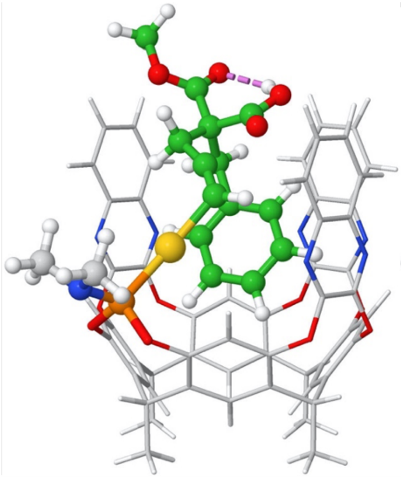

$\Delta G^{\ddagger}$ = +17.6
1a-$TS1_{5anti}H^{+}\subset$AuCav

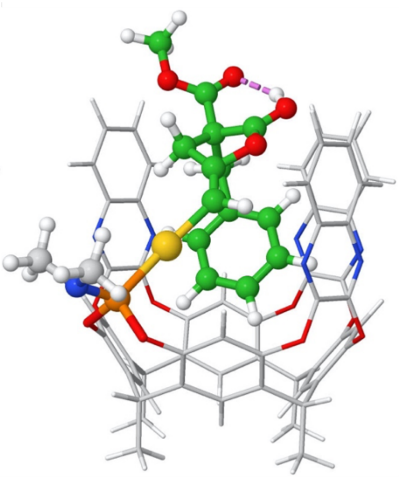

ΔG = +16.4
1a-$Int_{5anti}H^{+}\subset$AuCav

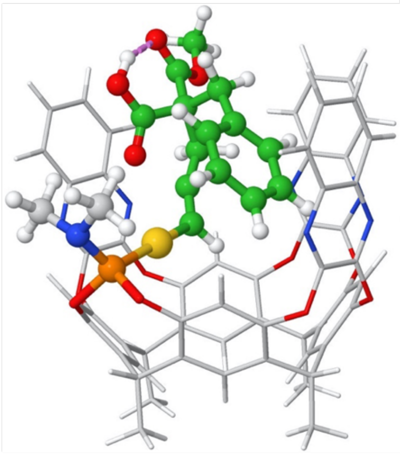

$\Delta G^{\ddagger}$ = +24.8
1a-$TS1_{5syn}H^{+}\subset$AuCav

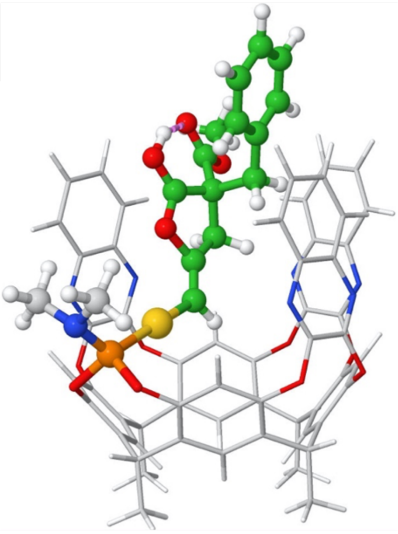

ΔG = +21.2
1a-$Int_{5syn}H^{+}\subset$AuCav

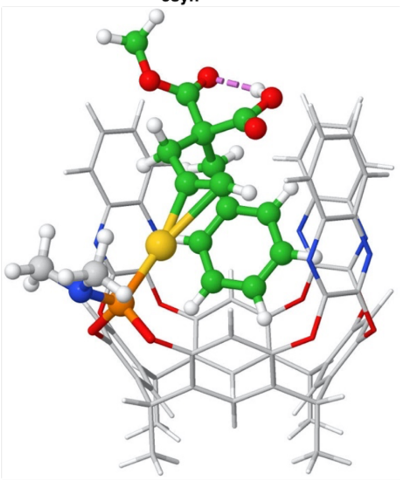

$\Delta G^{\ddagger}$ = +23.4
1a-$TS1_{6}H^{+}\subset$AuCav

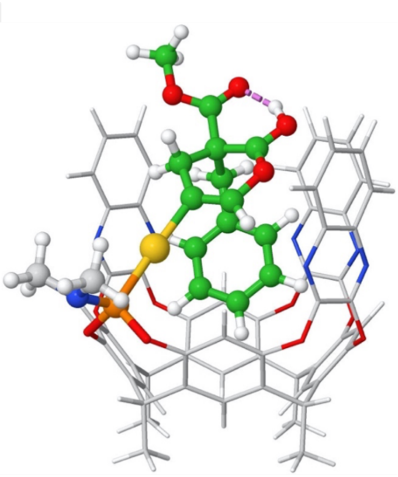

ΔG = +17.5
1a-$Int_{6}H^{+}\subset$AuCav

Figure S12. *Optimized geometries of the cyclization transition states and following intermediates for the cyclization of **1a** without triflate. Energies are in kcal/mol relative to **(TfO•CHCl$_3$)$\subset$AuCav**.*

VI.F. Energy of 1a-Int$_{5anti}$⊂AuCav without triflic acid

As discussed in the main text, we have also considered the energy of the cyclized intermediate **1a-Int$_{5anti}$⊂AuCav** without triflic acid bound (**1a-Int$_{5anti}$'⊂AuCav**). Geometries and energies are given in Figure S13.

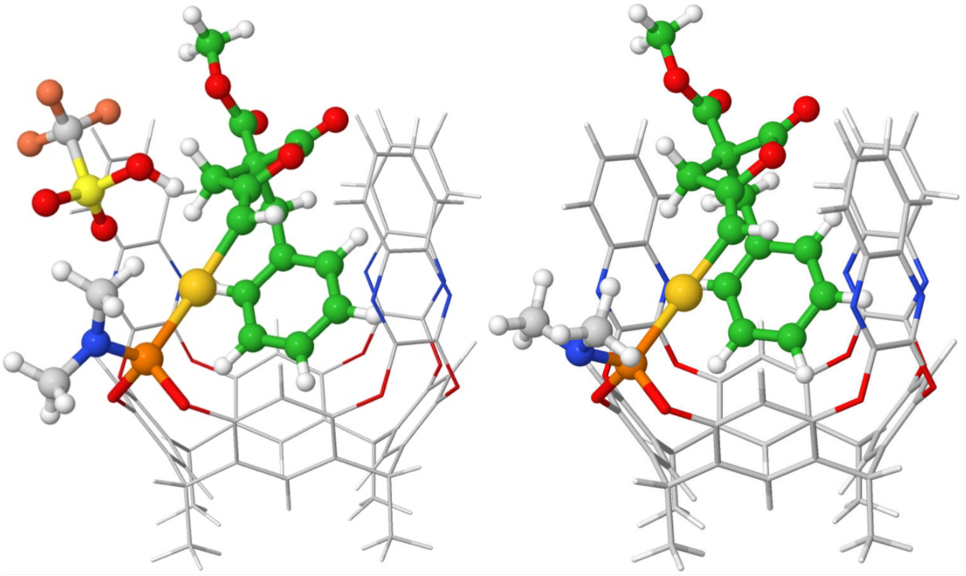

ΔG = +1.6
1a-Int$_{5anti}$⊂AuCav

ΔG = +5.8
1a-Int$_{5anti}$'⊂AuCav

Figure S13. *Optimized geometries for **1a-Int$_{5anti}$⊂AuCav** with and without triflic acid bound. Energies are in kcal/mol relative to **(TfO•CHCl$_3$)⊂AuCav**.*

VI.G. Binding modes of 2a to AuCav

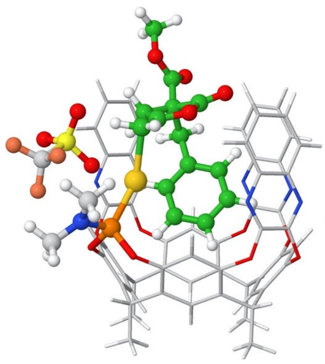

ΔG = 0.0
(TfO•2a)⊂AuCav

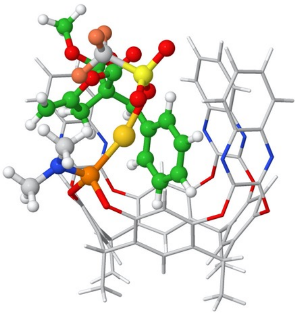

ΔG = +2.7
(TfO•2a)⊂AuCav-1

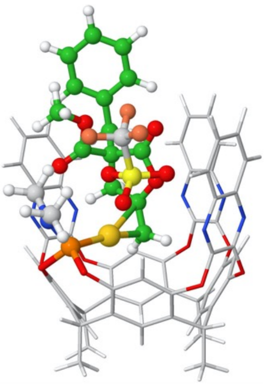

ΔG = +4.2
(TfO•2a')⊂AuCav

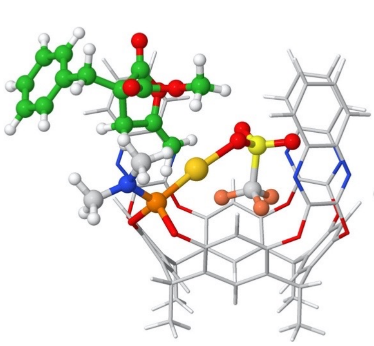

ΔG = +4.9
(TfO•2a)⊂AuCav-2

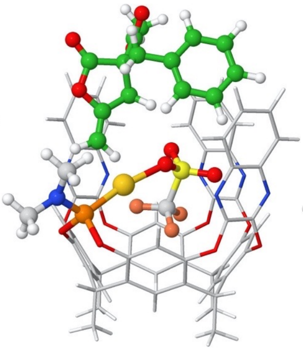

ΔG = +5.5
(TfO•2a)⊂AuCav-3

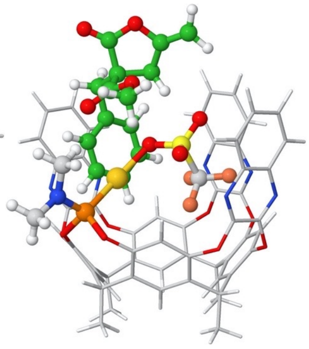

ΔG = +6.0
(TfO•2a)⊂AuCav-4

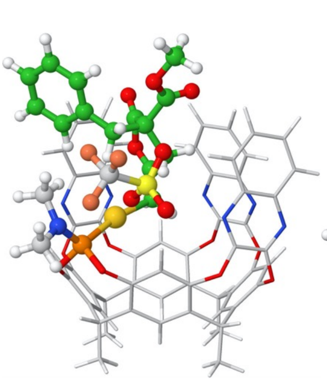

ΔG = +6.8
(TfO•2a)⊂AuCav-5

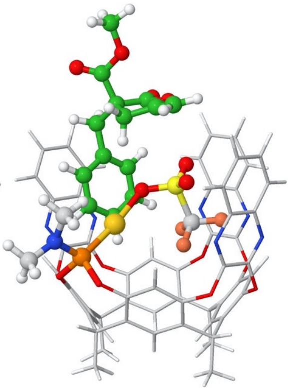

ΔG = +6.9
(TfO•2a)⊂AuCav-6

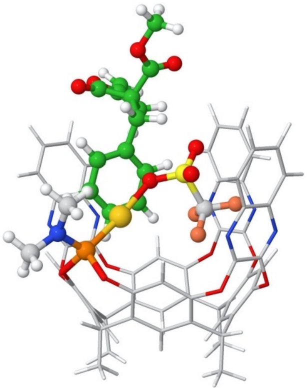

ΔG = +9.5
(TfO•2a)⊂AuCav-7

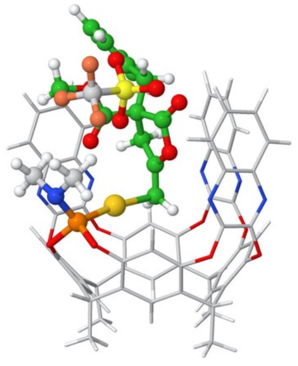

ΔG = +14.5
(TfO•2a)⊂AuCav-8

Figure S14. *Alternative binding modes for* ***(TfO•2a)⊂AuCav****. Relative energies are in kcal/mol.*

VI.H. Binding modes of 3a to AuCav

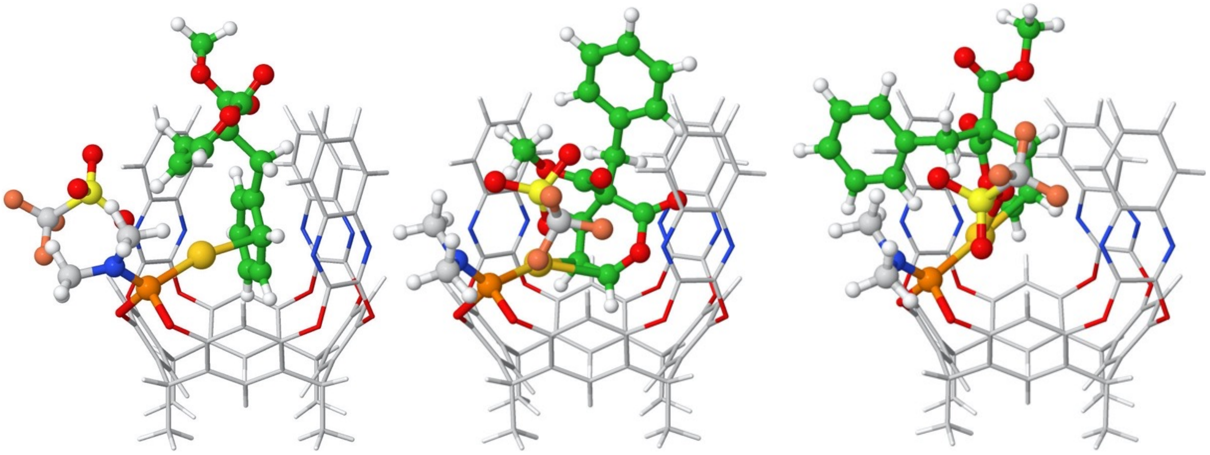

ΔG = 0.0 (TfO·3a)⊂AuCav | ΔG = +8.8 (TfO·3a)⊂AuCav-1 | ΔG = +9.6 (TfO·3a)⊂AuCav-2

Figure S15. *Alternative binding modes for* ***(TfO•3a)⊂AuCav****. Relative energies are in kcal/mol.*

VII. Additional results for reaction of 1b in AuCav

VII.A. Binding modes of 1b to AuCav

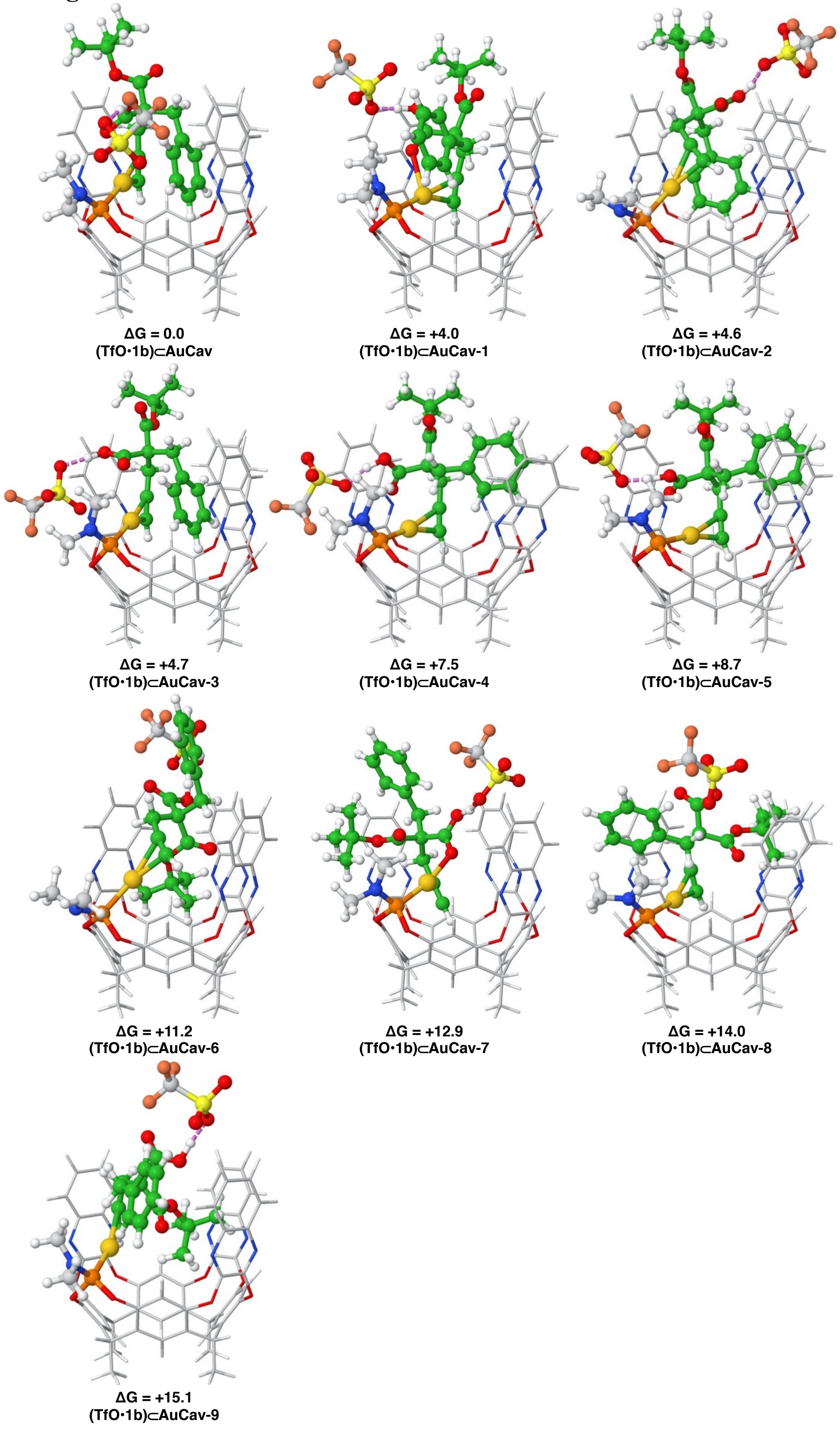

Figure S16. *Alternative binding modes for **(TfO•1b)⊂AuCav**. Relative energies are in kcal/mol.*

VII.B. 5-*syn*-*exo*-dig cyclization pathway

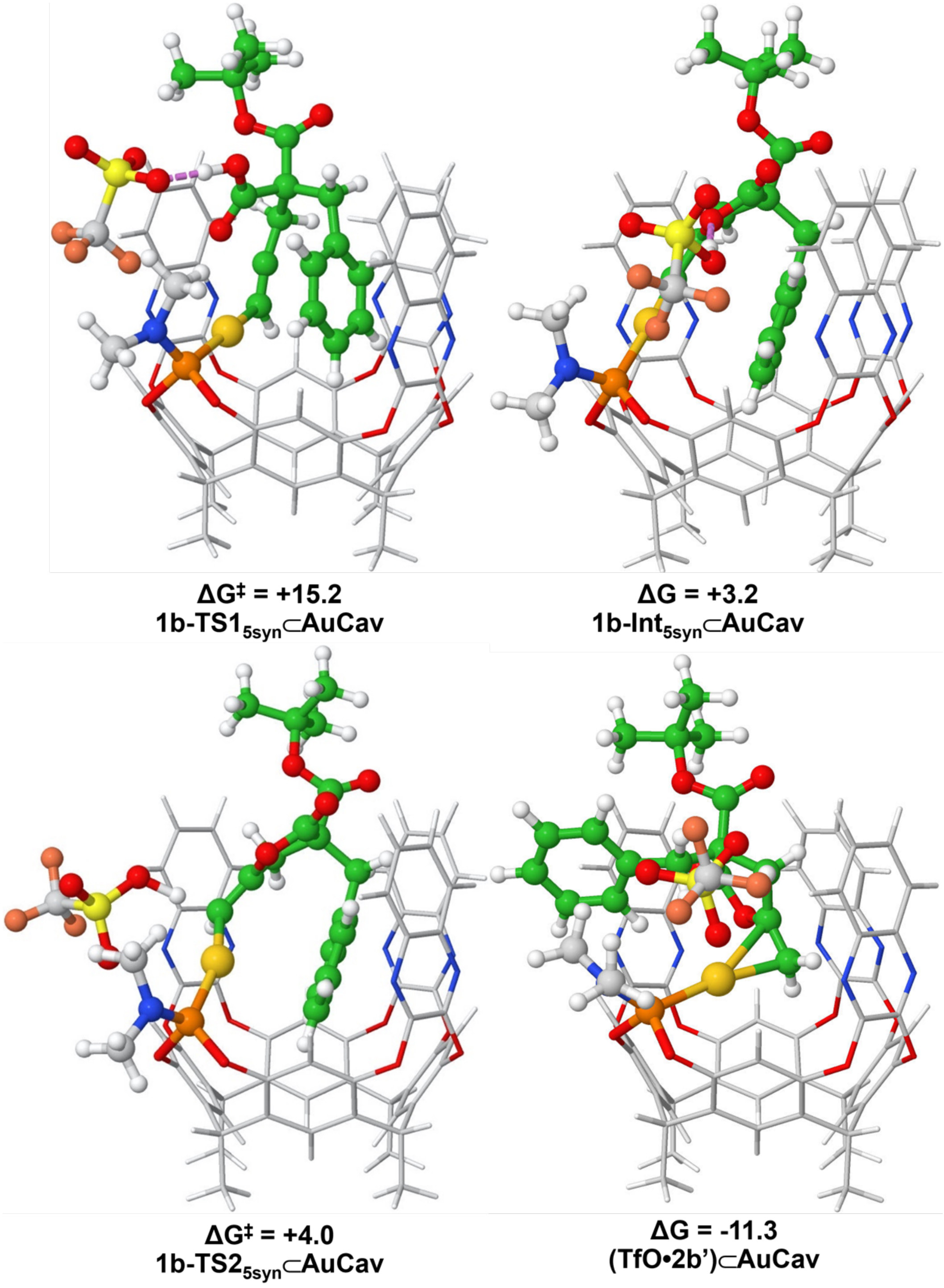

Figure S17. *Optimized geometries for the cycloisomerization of* ***1b*** *through 5-syn-exo-dig cyclization. Energies are in kcal/mol relative to* ***(TfO•CHCl$_3$)⊂AuCav***.

VII.C. 6-*endo*-dig cyclization pathway

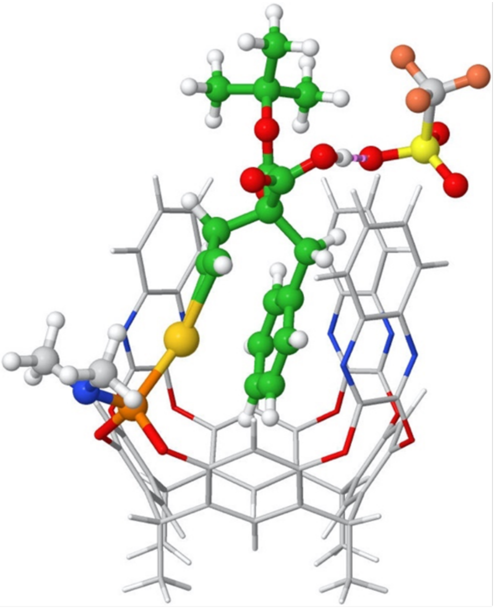

ΔG‡ = +18.1
1b-TS1$_6$⊂AuCav

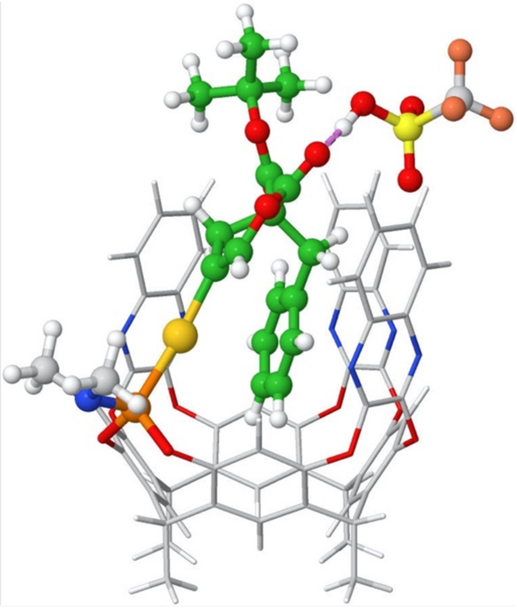

ΔG = +7.7
1b-Int$_6$⊂AuCav

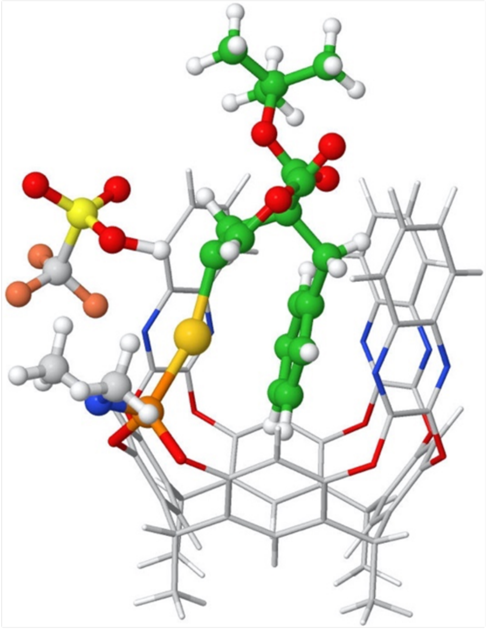

ΔG‡ = +10.1
1b-TS2$_6$⊂AuCav

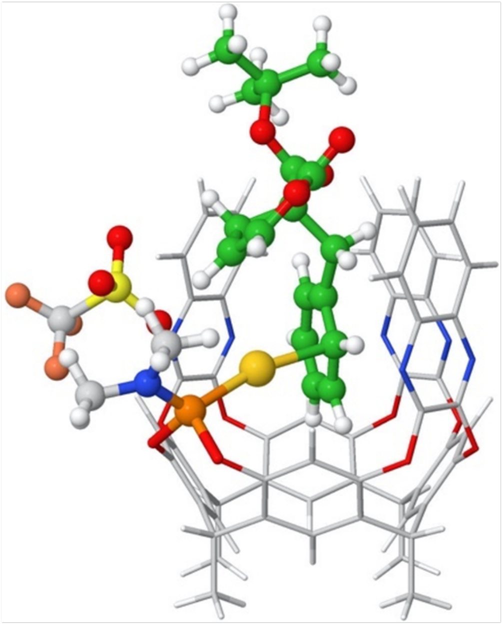

ΔG = -10.4
(TfO•3b)⊂AuCav

Figure S18. *Optimized geometries for the cycloisomerization of* ***1b*** *through 6-endo-dig cyclization. Energies are in kcal/mol relative to* ***(TfO•CHCl$_3$)⊂AuCav****.*

VII.D. Binding modes of 2b to AuCav

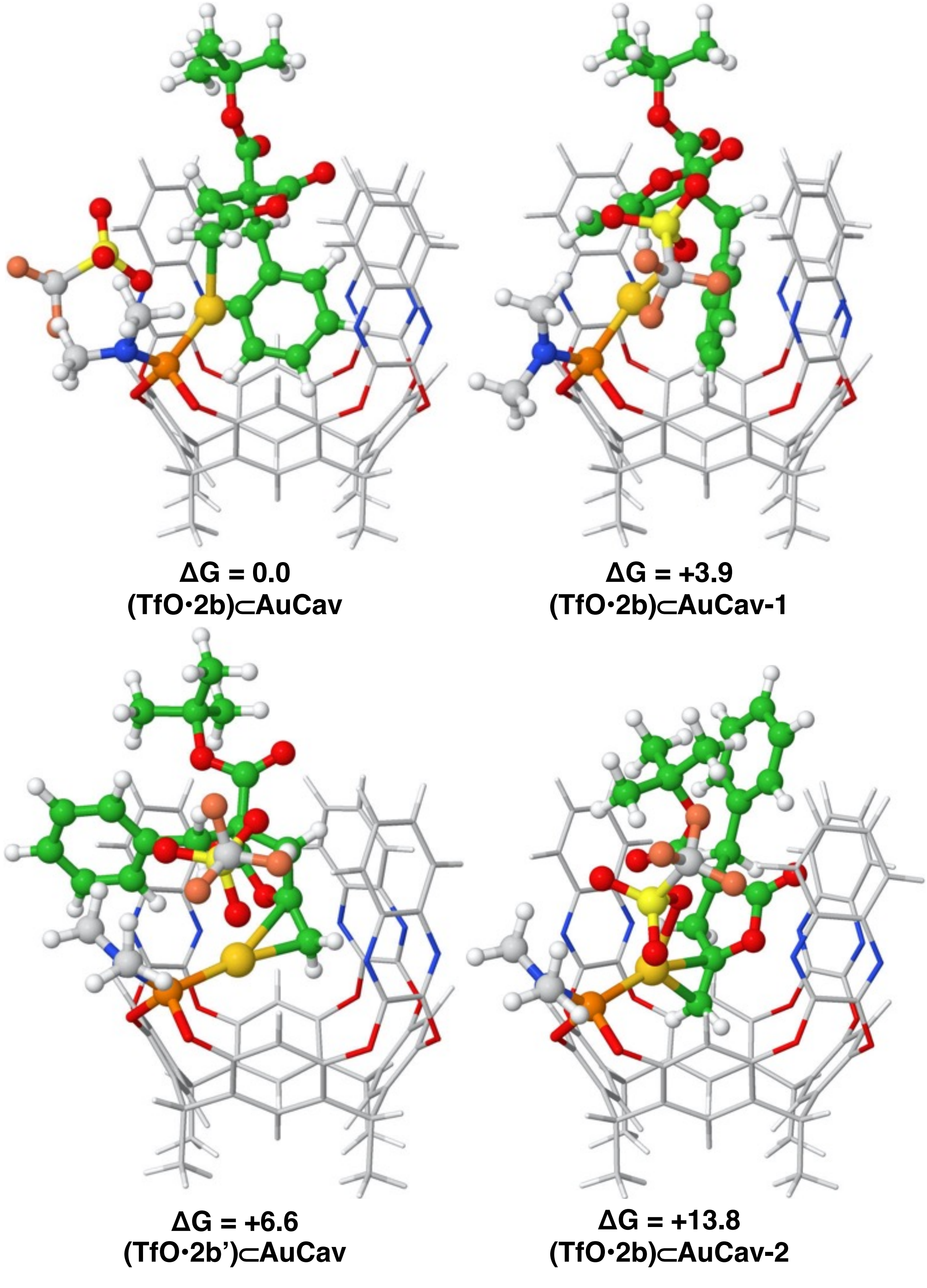

Figure S19. *Alternative binding modes for* ***(TfO•2b)$\subset$AuCav****. Relative energies are in kcal/mol.*

VII.E. Binding modes of 3b to AuCav

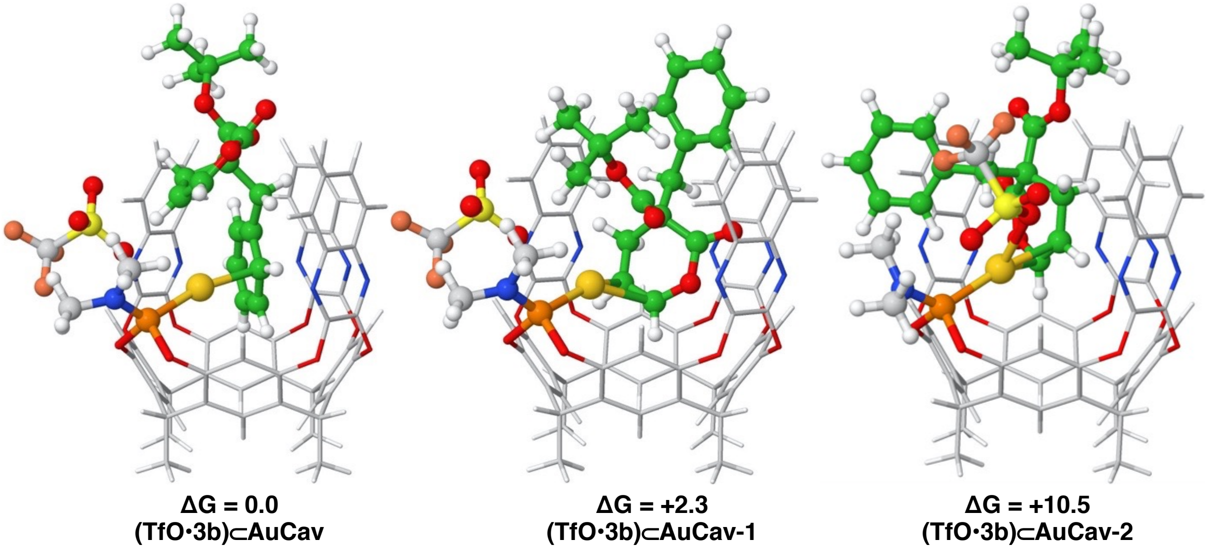

Figure S20. *Alternative binding modes for* ***(TfO•3b)⊂AuCav****. Relative energies are in kcal/mol.*

VII.F. Cyclization of 1b in AuCav without triflate

We have also considered the cyclization process taking place without the participation of triflate as a proton acceptor. The calculated energies for the three pathways are given in Figure S21 and the optimized geometries are given in Figure S22.

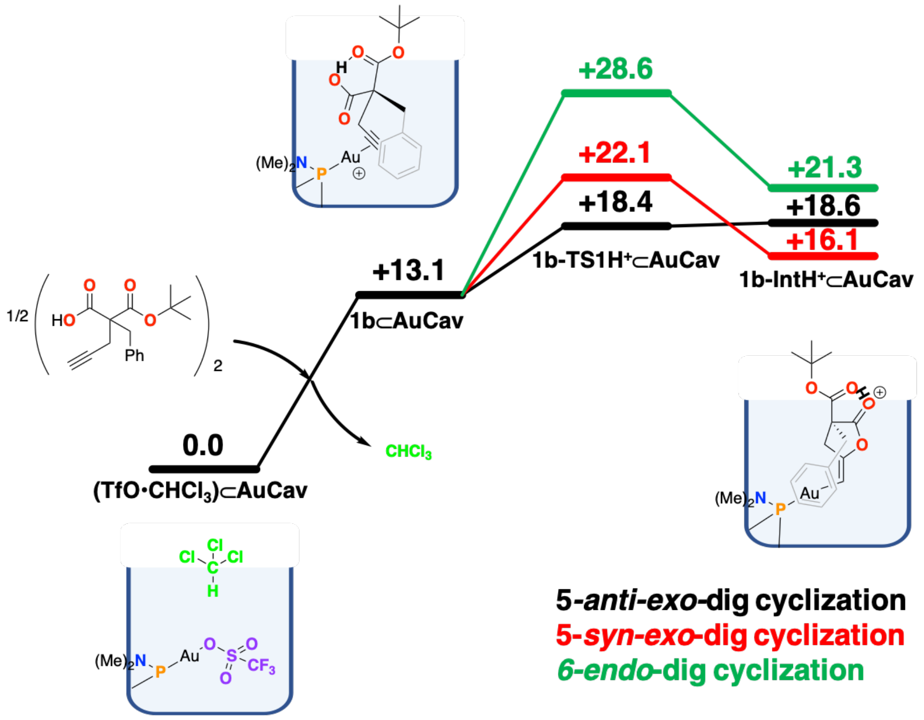

Figure S21. *Calculated free energy profile for the cycloisomerization of **1b** in the absence of triflate during the cyclization. Energies are in kcal/mol.*

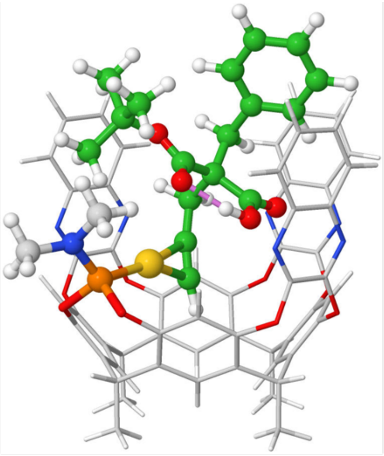

ΔG = +13.1
1b⊂AuCav

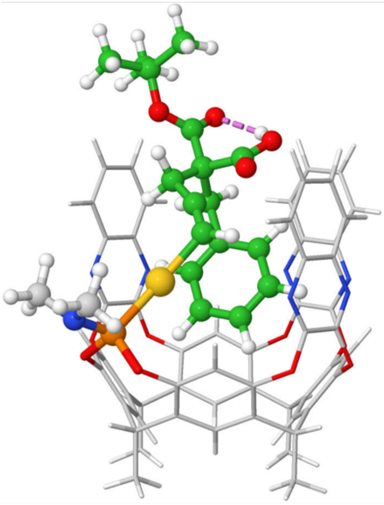

ΔG‡ = +18.4
1b-TS1$_{5anti}$H^{+}⊂AuCav

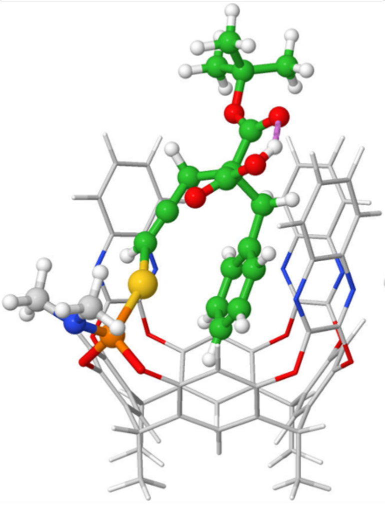

ΔG‡ = +22.1
1b-TS1$_{5syn}$H^{+}⊂AuCav

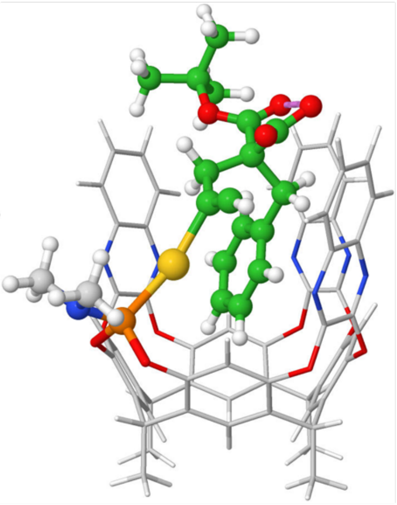

ΔG‡ = +28.6
1b-TS1$_{6}$H^{+}⊂AuCav

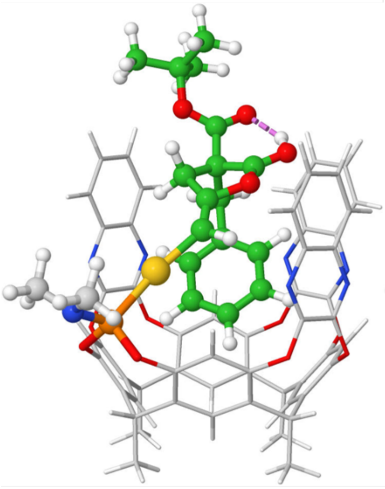

ΔG = +18.6
1b-Int$_{5anti}$H^{+}⊂AuCav

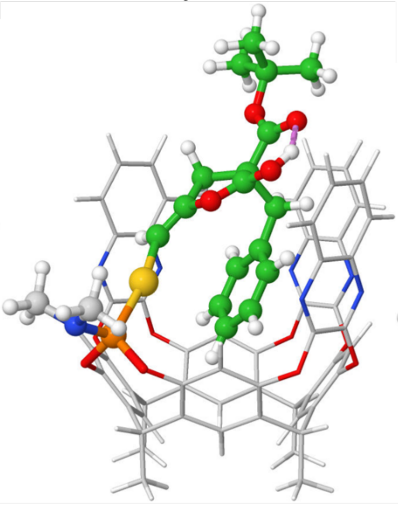

ΔG = +16.1
1b-Int$_{5syn}$H^{+}⊂AuCav

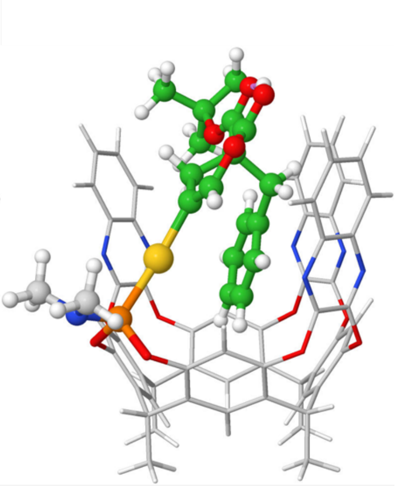

ΔG = +21.3
1b-Int$_{6}$H^{+}⊂AuCav

Figure S22. *Optimized geometries of* ***1b⊂AuCav****, the cyclization transition states and following intermediates for the cyclization of* ***1b*** *without triflate. Energies are in kcal/mol relative to* ***(TfO•CHCl$_3$)⊂AuCav****.*

VIII. Additional results for reaction of 1c in AuCav

VIII.A. Binding modes of 1c to AuCav

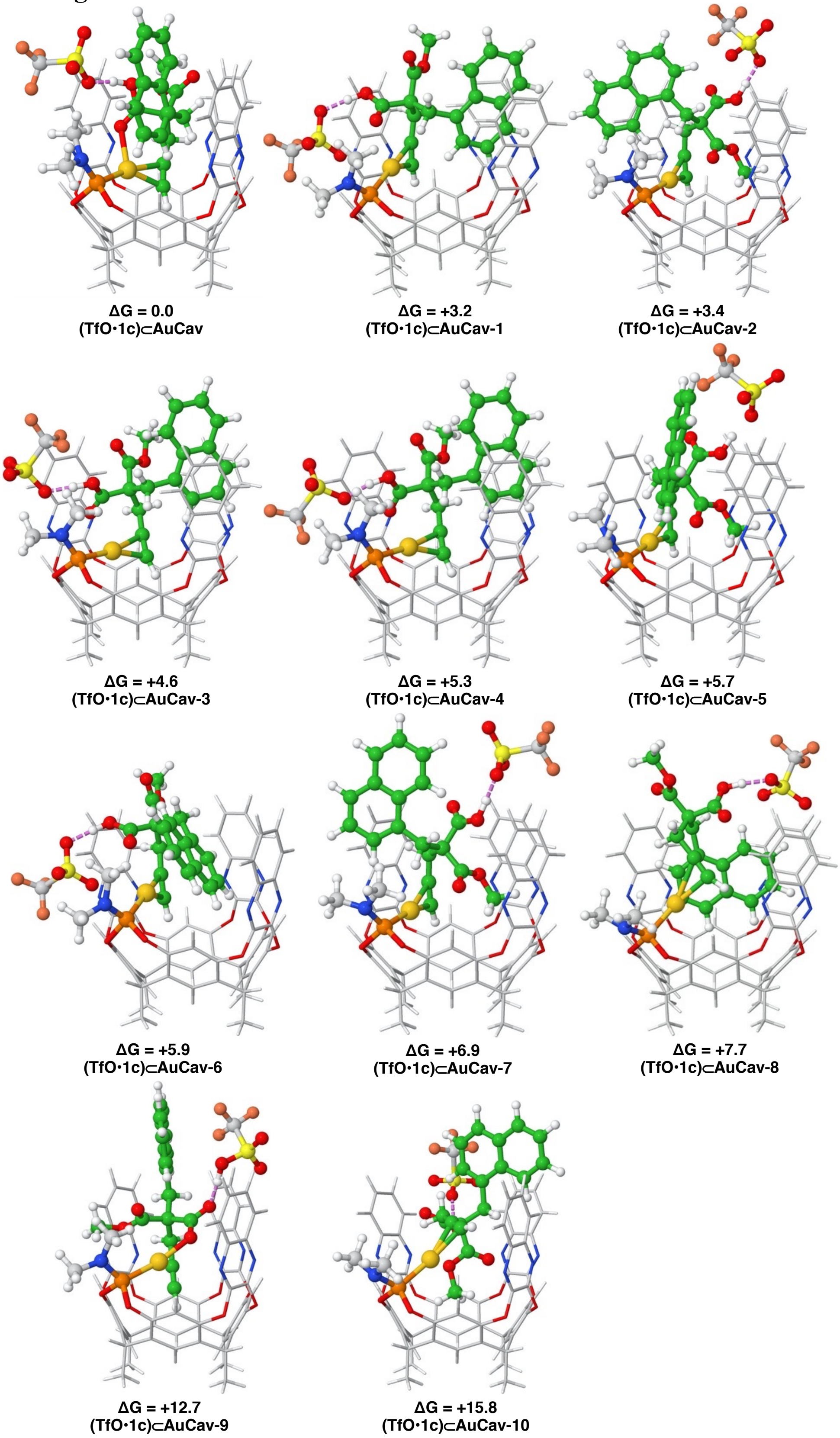

Figure S23. *Alternative binding modes for* ***(TfO•1c)⊂AuCav****. Energies are in kcal/mol.*

VIII.B. 5-*anti*-*exo*-dig cyclization pathway

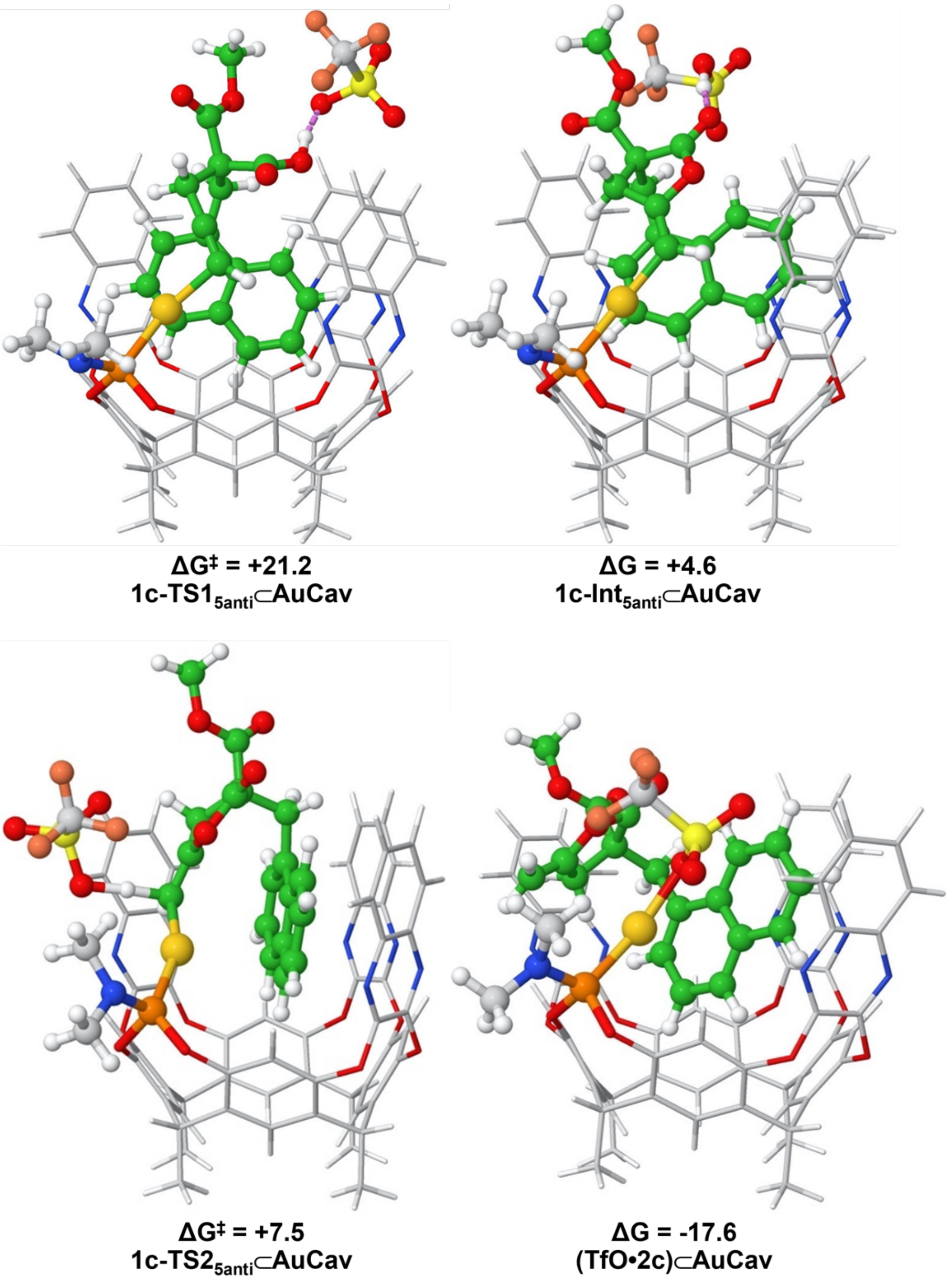

Figure S24. *Optimized geometries for the cycloisomerization of* ***1c*** *through 5-anti-exo-dig cyclization. Energies are in kcal/mol relative to* ***(TfO•CHCl₃)⊂AuCav****.*

VIII.C. 6-*endo*-dig cyclization pathway

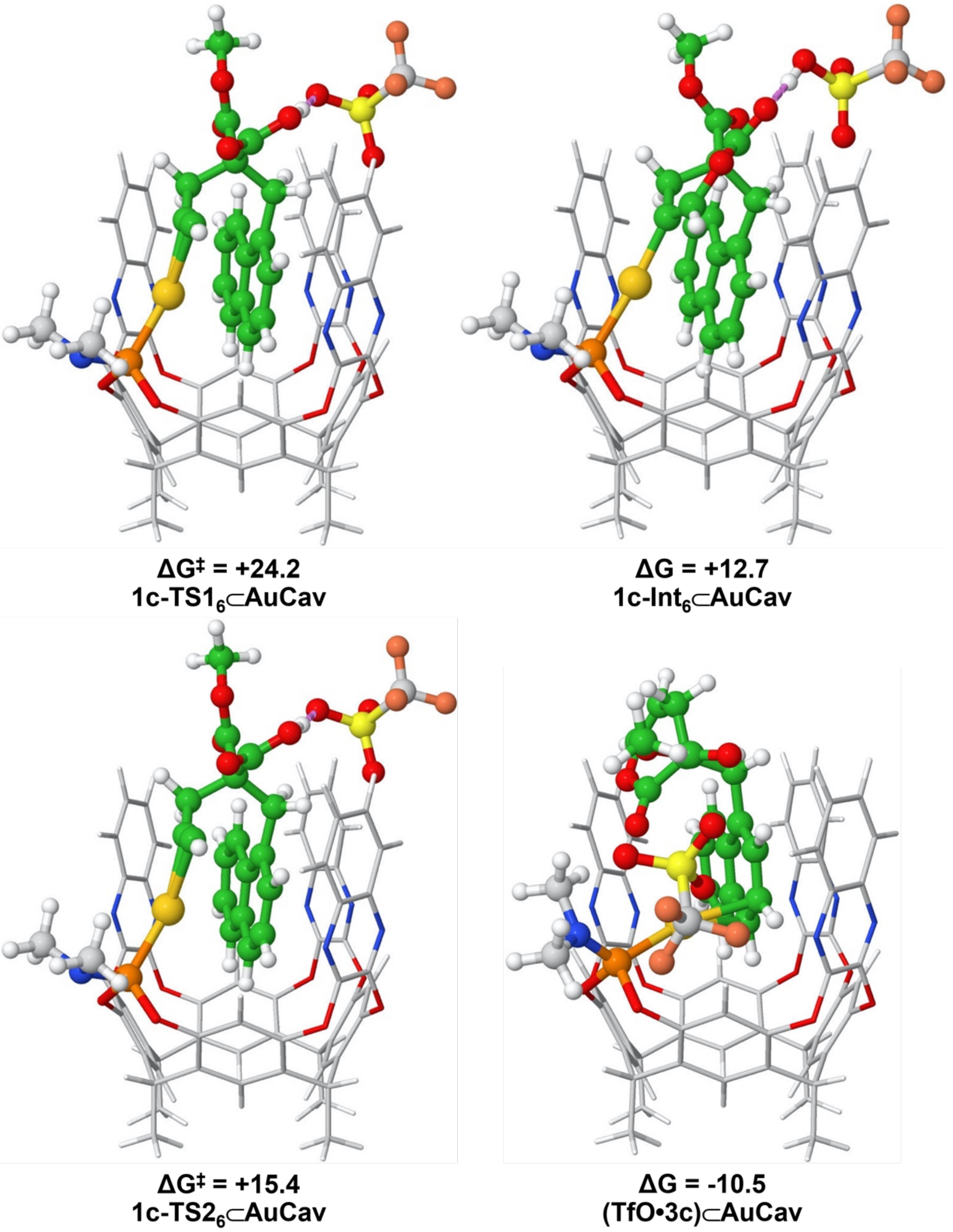

Figure S25. *Optimized geometries for the cycloisomerization of* ***1c*** *through 6-endo-dig cyclization. Energies are in kcal/mol relative to* ***(TfO•CHCl$_3$)⊂AuCav****.*

VIII.D. Binding modes of 2c to AuCav

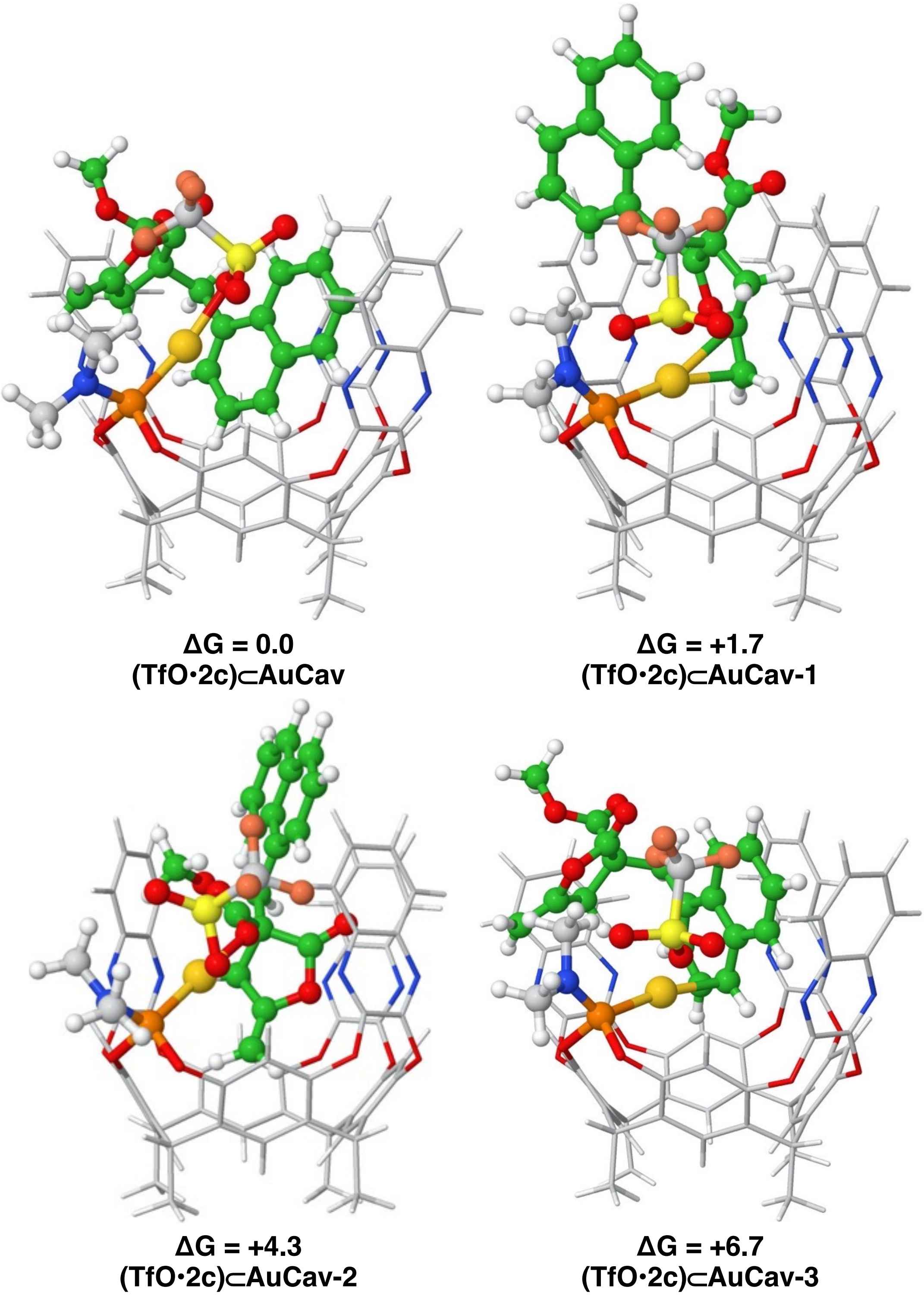

Figure S26. *Alternative binding modes for* ***(TfO•2c)⊂AuCav****. Relative energies are in kcal/mol.*

VIII.E. Binding modes of 3c to AuCav

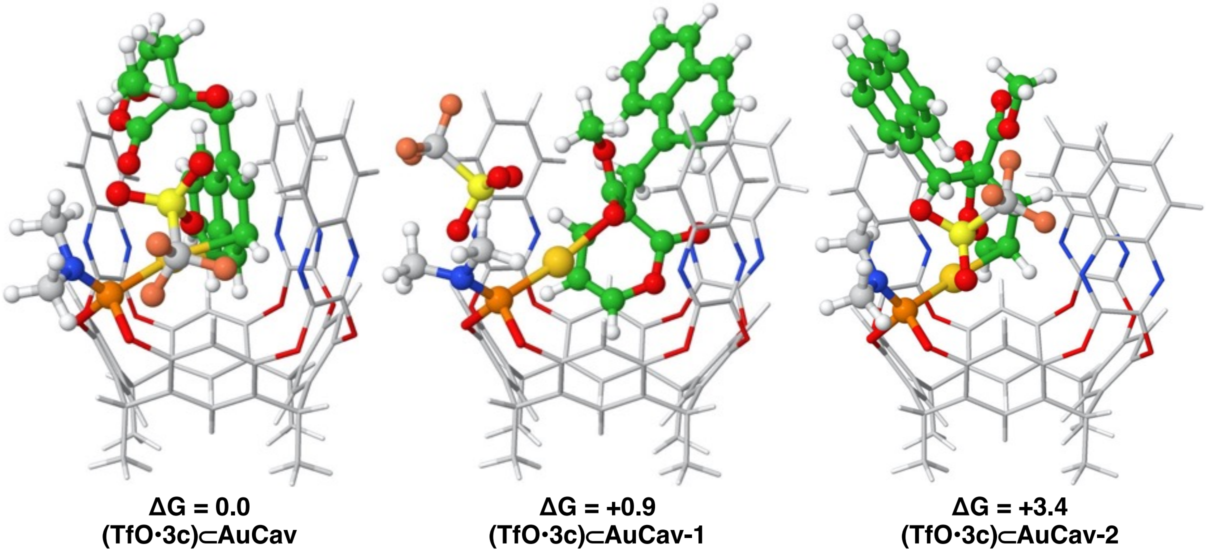

Figure S27. *Alternative binding modes for* ***(TfO•3c)⊂AuCav****. Relative energies are in kcal/mol.*

VIII.F. Cyclization of 1c in AuCav without triflate

We have also considered the cyclization process taking place without the participation of triflate as a proton acceptor. The calculated energies for the three pathways are given in Figure S28 and the optimized geometries are given in Figure S29.

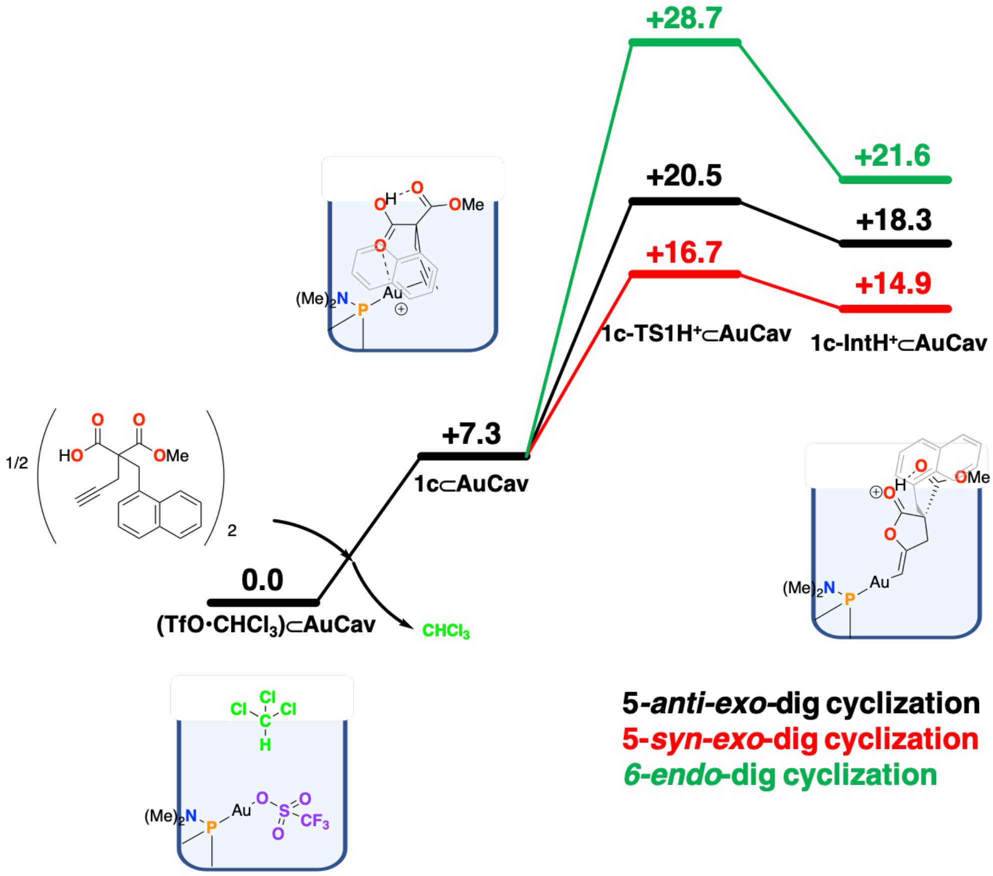

Figure S28. *Calculated free energy profile for the cycloisomerization of* ***1c*** *in the absence of triflate during the cyclization. Energies are in kcal/mol.*

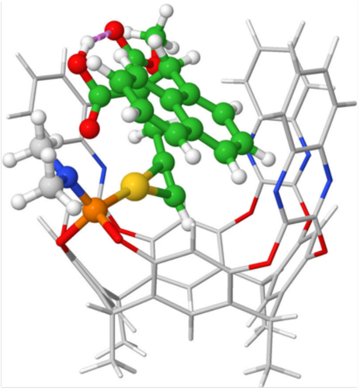

ΔG = +7.3
1c⊂AuCav

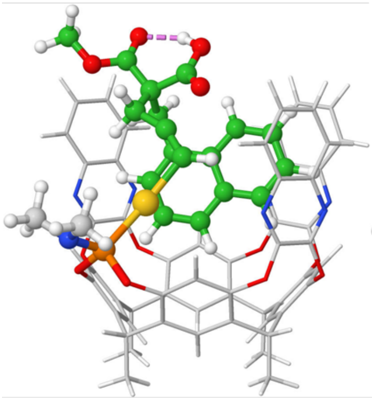

ΔG‡ = +20.5
1c-TS1$_{5anti}$H^{+}⊂AuCav

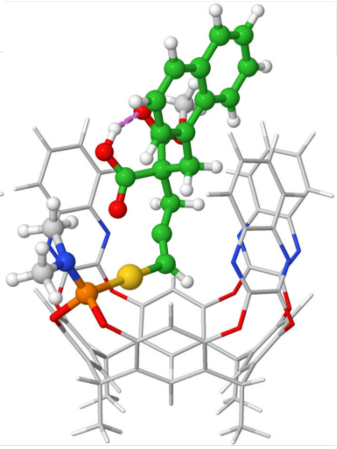

ΔG‡ = +16.7
1c-TS1$_{5syn}$H^{+}⊂AuCav

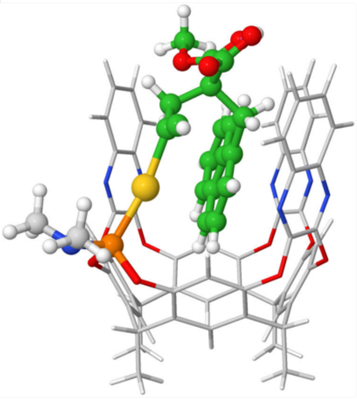

ΔG‡ = +28.7
1c-TS1$_{6}$H^{+}⊂AuCav

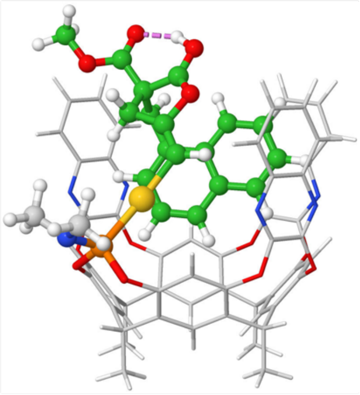

ΔG = +18.3
1c-Int$_{5anti}$H^{+}⊂AuCav

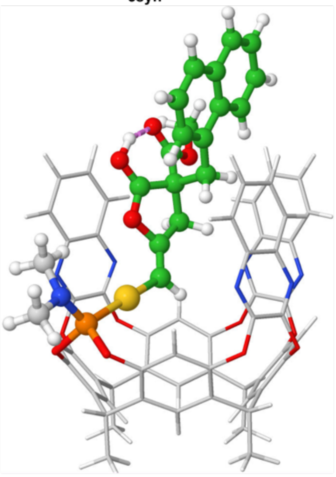

ΔG = +14.9
1c-Int$_{5syn}$H^{+}⊂AuCav

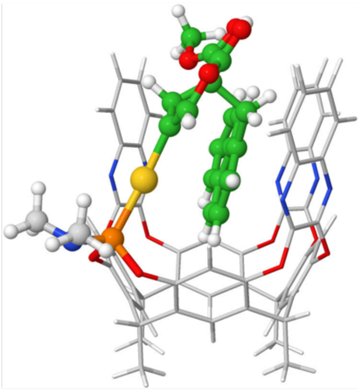

ΔG = +21.6
1c-Int$_{6}$H^{+}⊂AuCav

Figure S29. *Optimized geometries of **1c⊂AuCav**, the cyclization transition states and following intermediates for the cyclization of **1c** without triflate. Energies are in kcal/mol relative to **(TfO•CHCl$_3$)⊂AuCav**.*

IX. Cycloisomerization of 1a by [Au]

IX.A. 5-*anti*-*exo*-dig cyclization pathway

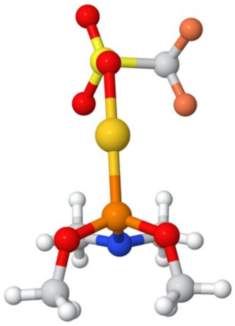

$\Delta G = 0.0$
TfO[Au]

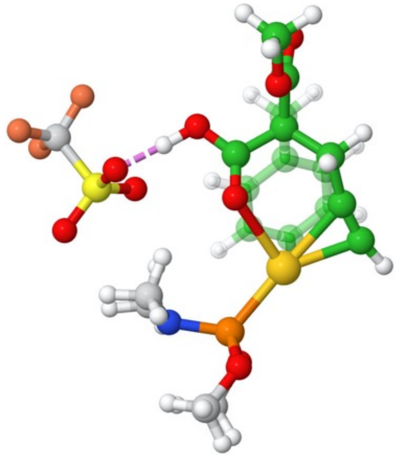

$\Delta G = +1.6$
(TfO·1a)[Au]

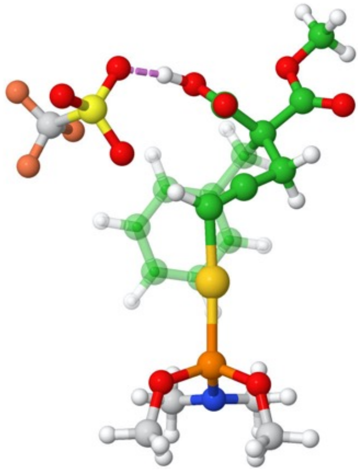

$\Delta G^{\ddagger} = +12.5$
1a-TS1$_{5anti}$[Au]

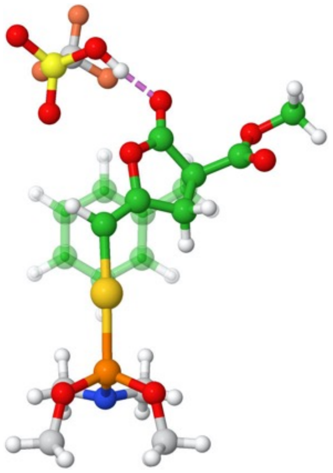

$\Delta G = +1.9$
1a-Int$_{5anti}$ [Au]

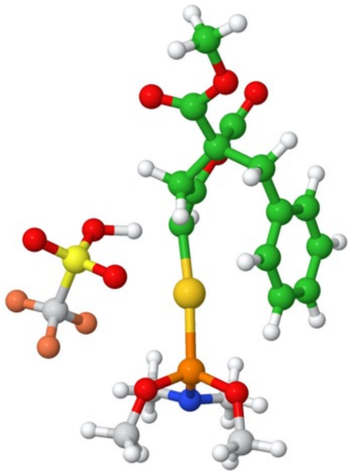

$\Delta G^{\ddagger} = +3.6$
1a-TS2$_{5anti}$ [Au]

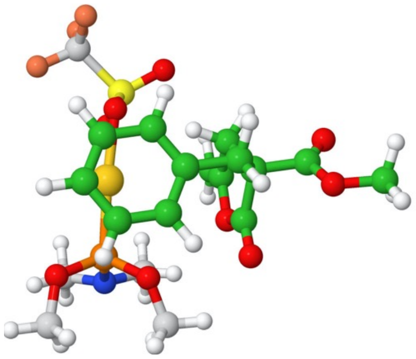

$\Delta G = -22.6$
(TfO·2a)[Au]

Figure S30. *Optimized geometries for the cycloisomerization of **1a** through 5-anti-exo-dig cyclization by **[Au]**. Relative energies are in kcal/mol.*

IX.B. 5-*syn*-*exo*-dig cyclization pathway

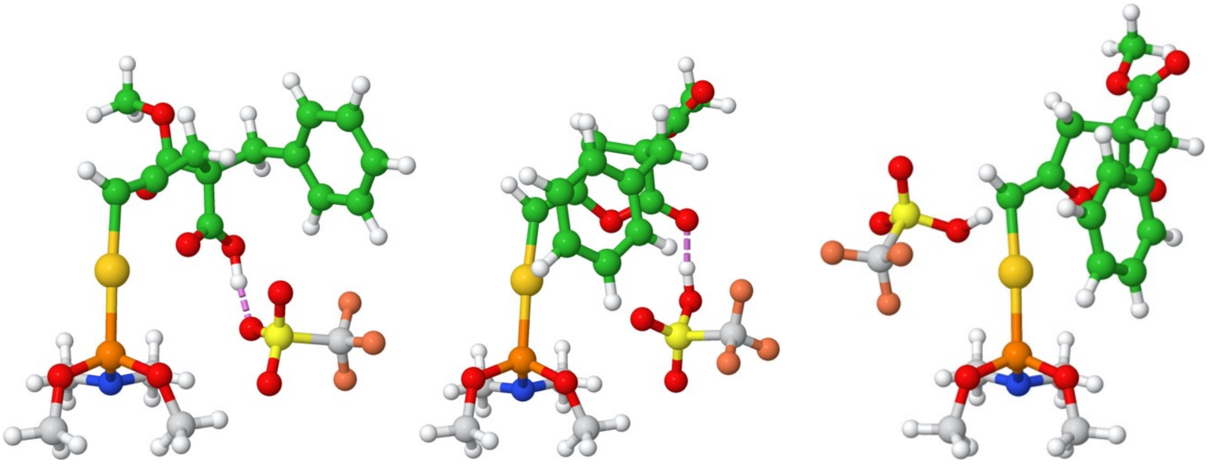

$\Delta G^{\ddagger}$ = +15.2 **1a-TS1$_{5syn}$[Au]** | ΔG = -0.8 **1a-Int$_{5syn}$ [Au]** | $\Delta G^{\ddagger}$ = 0.0 **1a-TS2$_{5syn}$ [Au]**

Figure S31. *Optimized geometries for the cycloisomerization of* ***1a*** *through 5-syn-exo-dig cyclization by* ***[Au]****. Energies are in kcal/mol relative to* ***TfO[Au]****.*

IX.C. 6-*endo*-dig cyclization pathway

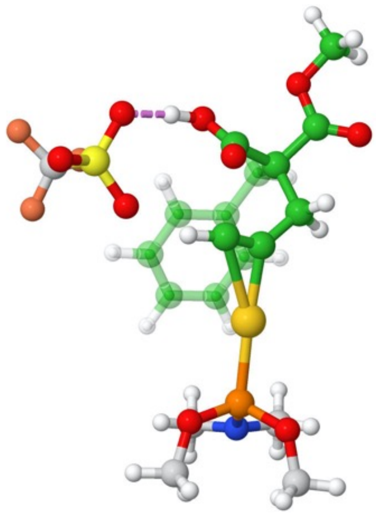

$\Delta G^{‡}$ = +19.3
1a-TS1$_6$[Au]

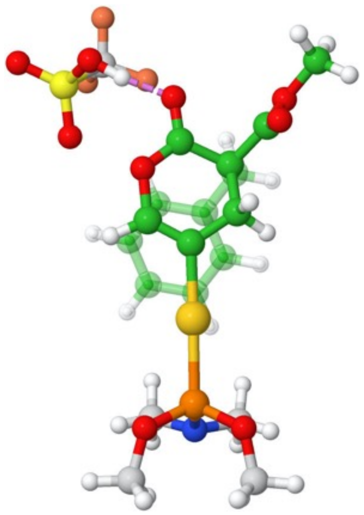

ΔG = +6.6
1a-Int$_6$[Au]

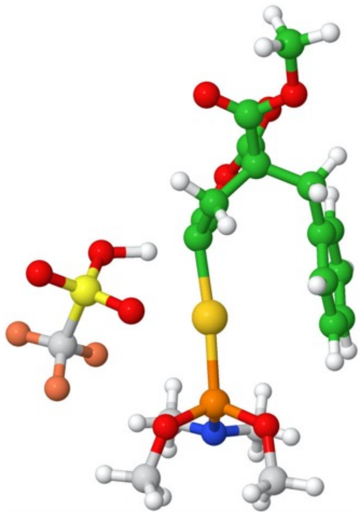

$\Delta G^{‡}$ = +11.2
1a-TS2$_6$[Au]

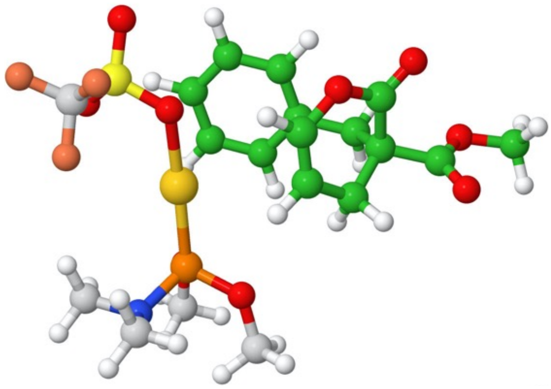

ΔG = -12.4
(TfO·3a)[Au]

Figure S32. *Optimized geometries for the cycloisomerization of* ***1a*** *through 6-endo-dig cyclization by* ***[Au]****. Energies are in kcal/mol relative to* ***TfO[Au]****.*

IX.D. Cyclization without triflate

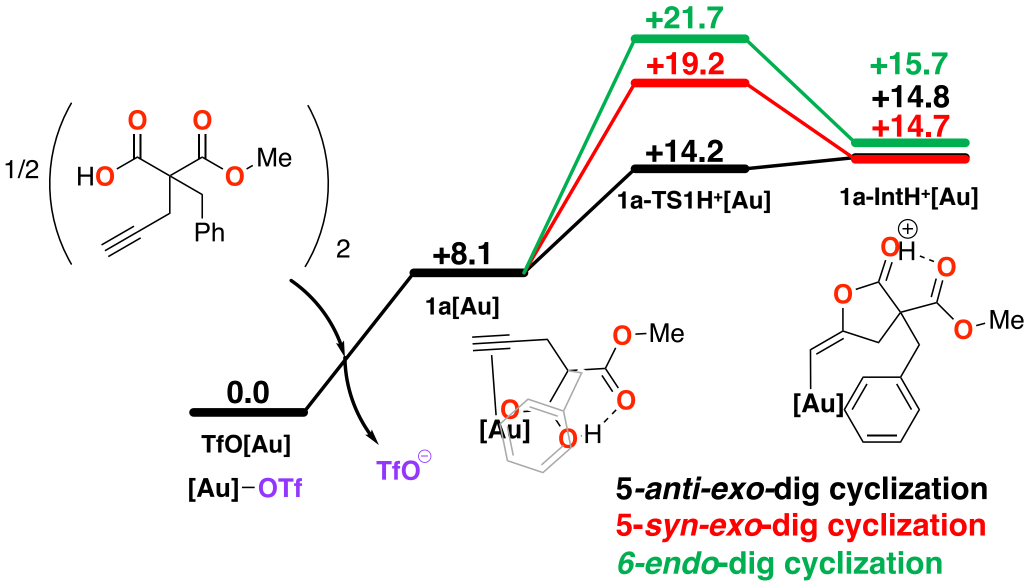

Figure S33. *Calculated free energy profile for the cyclization of **1a** without triflate by **[Au]**. Energies are in kcal/mol.*

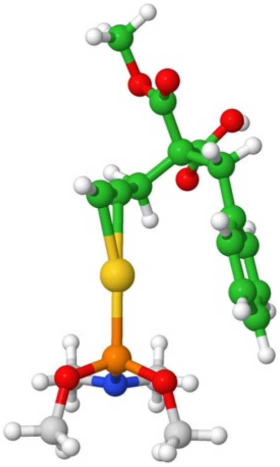

ΔG = +8.1
1a[Au]

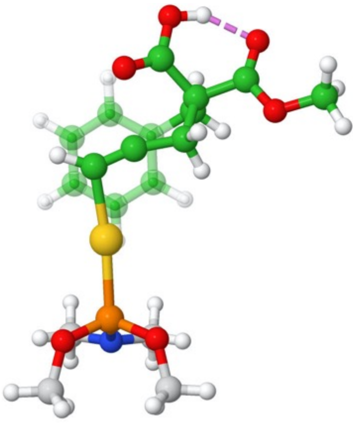

ΔG‡ = +14.2
1a-TS1$_{5anti}$H^{+}[Au]

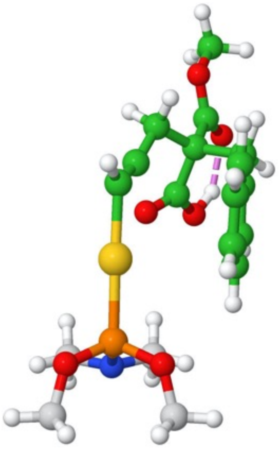

ΔG‡ = +19.2
1a-TS1$_{5syn}$H^{+}[Au]

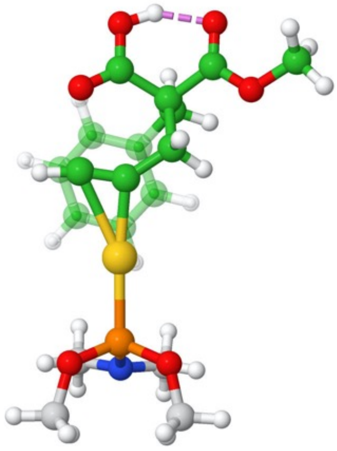

ΔG‡ = +21.7
1a-TS1$_{6}$H^{+}[Au]

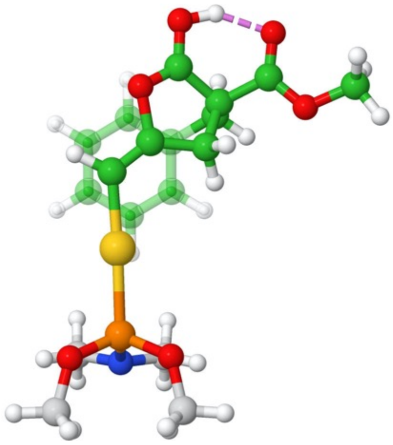

ΔG = +14.8
1a-Int$_{5anti}$H^{+}[Au]

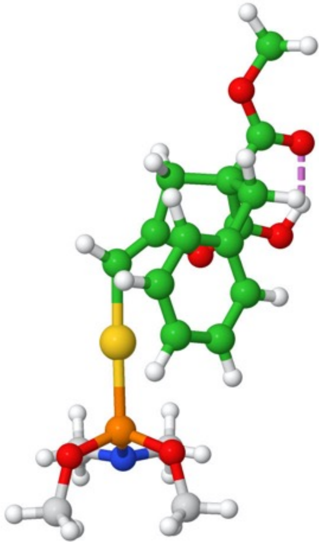

ΔG = +14.7
1a-Int$_{5syn}$H^{+}[Au]

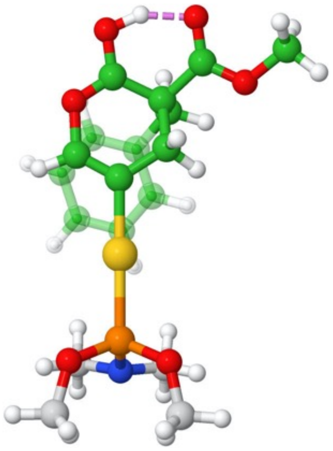

ΔG = +15.7
1a-Int$_{6}$H^{+}[Au]

Figure S34. *Optimized geometries of **1a[Au]**, the cyclization transition state and subsequent intermediate of **1a** by **[Au]** without triflate. Energies are in kcal/mol relative to **TfO[Au]**.*

X. Absolute energies and energy corrections

- **E_{opt}** is the electronic energy obtained at the theory level of the optimization in the gas phase.
- **G_{qRRHO}** is the correction to the Gibbs free energy using the quasi-rigid rotor harmonic oscillator approximation.
- **E_{bb}** is the electronic energy obtained from single-point calculations with the larger basis set
- **ΔE_{SMD}** is the solvation energy obtained from the difference between **E_{opt}** and the energy of a single-point energy calculation using the SMD method.
- **E_{SS}** is the correction for a 1M solution standard state, which is +1.9 kcal/mol for all species except chloroform, for which the correction is +3.7 kcal/mol.
- **$G_{total} = E_{bb} + G_{qRRHO} + \Delta E_{SMD} + E_{SS}$**

Table S1. *Calculated energies (a.u.) of all structures without **AuCav** or **[Au]**.*

Species	E_{opt}	G_{qRRHO}	E_{bb}	ΔE_{SMD}	E_{SS}	G_{total}
**$CHCl_3$**	-1419.279715	-0.008284	-1419.397355	-0.007741	0.005418	-1419.408351
**1a**	-842.839691	0.211005	-843.104700	-0.018353	0.003028	-842.909021
**1b**	-960.817060	0.290647	-961.111015	-0.020826	0.003028	-960.838166
**1c**	-996.494472	0.254546	-996.799941	-0.022527	0.003028	-996.564894
**$(1a)_2$**	-1685.723648	0.445912	-1686.245908	-0.027735	0.003028	-1685.824703
**$(1b)_2$**	-1921.677900	0.607238	-1922.256992	-0.032725	0.003028	-1921.679451
**$(1c)_2$**	-1993.039496	0.536250	-1993.639305	-0.032799	0.003028	-1993.132827
**2a**	-842.887028	0.213789	-843.145232	-0.016836	0.003028	-842.945251
**3a**	-842.879075	0.214872	-843.136794	-0.017520	0.003028	-842.936414
**2b**	-960.863722	0.294837	-961.149470	-0.018088	0.003028	-960.869693
**3b**	-960.876373	0.296188	-961.143150	-0.019098	0.003028	-960.863032
**2c**	-996.544330	0.258495	-996.841401	-0.021281	0.003028	-996.601159
**3c**	-996.540544	0.259755	-996.836167	-0.021088	0.003028	-996.594472
**TfO^-**	-961.499627	-0.003993	-961.788874	-0.065143	0.003028	-961.854981
**TfOH**	-962.008415	0.006837	-962.278940	-0.005519	0.003028	-962.274595
**AgOTf**	-1108.442416	-0.007391	-1108.705372	-0.023651	0.003028	-1108.733387
**AgCl**	-607.234754	-0.023536	-607.269448	-0.024566	0.003028	-607.314522

Table S2. *Calculated energies (a.u.) of all host-guest complexes without substrates.*

Species	E_{opt}	G_{qRRHO}	E_{bb}	ΔE_{SMD}	E_{SS}	G_{total}
**(TfO•CHCl$_3$)⊂AuCav**	-6078.872886	0.856820	-6080.176085	-0.058600	0.003028	-6079.374837
**TfO⊂AuCav**	-4659.566137	0.842528	-4660.753400	-0.056313	0.003028	-4659.964156
**TfO⊂AuCav-1**	-4659.561852	0.842055	-4660.749247	-0.057434	0.003028	-4659.961598
**CHCl$_3$⊂AuCav**	-5117.189658	0.836635	-5118.234996	-0.092666	0.003028	-5117.488000
**(CHCl$_3$)$_2$⊂AuCav**	-6536.482072	0.849458	-6537.646082	-0.098487	0.003028	-6536.892084
**(CHCl$_3$)⊂AuClCav**	-5577.638996	0.834744	-5578.723764	-0.058959	0.003028	-5577.944951

Table S3. *Calculated energies (a.u.) of structures for the cycloisomerization of* ***1a***.

Species	E_{opt}	G_{qRRHO}	E_{bb}	ΔE_{SMD}	E_{SS}	G_{total}
**(TfO•1a)⊂AuCav**	-5502.455549	1.083361	-5503.895828	-0.062148	0.003028	-5502.871588
**1a-TS1$_{5anti}$⊂AuCav**	-5502.426290	1.080295	-5503.873149	-0.072555	0.003028	-5502.862381
**1a-Int$_{5anti}$⊂AuCav**	-5502.470770	1.083166	-5503.906686	-0.055846	0.003028	-5502.876338
**1a-TS2$_{5anti}$⊂AuCav**	-5502.467792	1.080098	-5503.902636	-0.057052	0.003028	-5502.876562
**(TfO•2a)⊂AuCav**	-5502.495368	1.084995	-5503.930638	-0.062954	0.003028	-5502.905569
**1a-TS1$_{5syn}$⊂AuCav**	-5502.429081	1.080123	-5503.870382	-0.073670	0.003028	-5502.860901
**1a-Int$_{5syn}$⊂AuCav**	-5502.468137	1.084321	-5503.902788	-0.058680	0.003028	-5502.874119
**1a-TS2$_{5syn}$⊂AuCav**	-5502.457007	1.079342	-5503.894378	-0.060527	0.003028	-5502.872535
**(TfO•2a')⊂AuCav**	-5502.480943	1.083237	-5503.919605	-0.065469	0.003028	-5502.898810
**1a-TS1$_6$⊂AuCav**	-5502.420168	1.080797	-5503.866310	-0.073044	0.003028	-5502.855529
**1a-Int$_6$⊂AuCav**	-5502.471312	1.086698	-5503.908356	-0.054632	0.003028	-5502.873262
**1a-TS2$_6$⊂AuCav**	-5502.454085	1.081391	-5503.888655	-0.061337	0.003028	-5502.865573
**(TfO•3a)⊂AuCav**	-5502.488426	1.087441	-5503.925997	-0.062576	0.003028	-5502.898104

Table S4. *Calculated energies (a.u.) of additional structures for the cycloisomerization of **1a**.*

Species	E_{opt}	G_{qRRHO}	E_{bb}	ΔE_{SMD}	E_{SS}	G_{total}
**1a⊂AuCav**	-4540.798214	1.061068	-4541.969630	-0.103159	0.003028	-4541.008694
**1a⊂AuCav-1**	-4540.800807	1.062375	-4541.970901	-0.098681	0.003028	-4541.004179
**1a⊂AuCav-2**	-4540.793575	1.061986	-4541.965848	-0.102959	0.003028	-4541.003794
**1a⊂AuCav-3**	-4540.794871	1.060010	-4541.965698	-0.099396	0.003028	-4541.002055
**1a⊂AuCav-4**	-4540.790393	1.061964	-4541.963068	-0.103318	0.003028	-4541.001394
**1a⊂AuCav-5**	-4540.794683	1.060180	-4541.965733	-0.098434	0.003028	-4541.000960
**1a⊂AuCav-6**	-4540.787349	1.058433	-4541.960148	-0.101466	0.003028	-4541.000153
**1a⊂AuCav-7**	-4540.782564	1.060227	-4541.958596	-0.102855	0.003028	-4540.998196
**1a⊂AuCav-8**	-4540.781017	1.060091	-4541.951443	-0.101964	0.003028	-4540.990288
**1a⊂AuCav-9**	-4540.775134	1.061254	-4541.946877	-0.100171	0.003028	-4540.982766
**1a⊂AuCav-10**	-4540.752882	1.062220	-4541.923514	-0.103456	0.003028	-4540.961723
**(TfO•1a)⊂AuCav-1**	-5502.444478	1.080058	-5503.888040	-0.066001	0.003028	-5502.870955
**(TfO•1a)⊂AuCav-2**	-5502.438447	1.080330	-5503.880171	-0.068795	0.003028	-5502.865608
**(TfO•1a)⊂AuCav-3**	-5502.426799	1.080538	-5503.874433	-0.074106	0.003028	-5502.864973
**(TfO•1a)⊂AuCav-4**	-5502.444431	1.081188	-5503.885358	-0.063610	0.003028	-5502.864752
**(TfO•1a)⊂AuCav-5**	-5502.433914	1.079747	-5503.877804	-0.069542	0.003028	-5502.864571
**(TfO•1a)⊂AuCav-6**	-5502.429056	1.082398	-5503.873094	-0.076815	0.003028	-5502.864483
**(TfO•1a)⊂AuCav-7**	-5502.425902	1.082031	-5503.871641	-0.075250	0.003028	-5502.861832
**(TfO•1a)⊂AuCav-8**	-5502.427943	1.080081	-5503.870900	-0.073091	0.003028	-5502.860881
**(TfO•1a)⊂AuCav-9**	-5502.429730	1.079120	-5503.871569	-0.070242	0.003028	-5502.859664
**(TfO•1a)⊂AuCav-10**	-5502.408237	1.079925	-5503.856165	-0.080768	0.003028	-5502.853980
**1a-TS1$_{5anti}$H^{+}⊂AuCav**	-4540.791803	1.062466	-4541.962832	-0.098520	0.003028	-4540.995858
**1a-Int$_{5anti}$H^{+}⊂AuCav**	-4540.798568	1.064860	-4541.966879	-0.098722	0.003028	-4540.997713
**1a-TS1$_{5syn}$H^{+}⊂AuCav**	-4540.779960	1.061719	-4541.949159	-0.100002	0.003028	-4540.984414
**1a-Int$_{5syn}$H^{+}⊂AuCav**	-4540.785440	1.063427	-4541.954485	-0.102012	0.003028	-4540.990042
**1a-TS1$_{6}$H^{+}⊂AuCav**	-4540.782499	1.062753	-4541.954048	-0.098280	0.003028	-4540.986547
**1a-Int$_{6}$H^{+}⊂AuCav**	-4540.801298	1.066389	-4541.968050	-0.097303	0.003028	-4540.995937
**1a-Int$_{5anti}$'⊂AuCav**	-4540.415760	1.053246	-4541.592294	-0.059088	0.003028	-4540.595109
**(TfO•2a)⊂AuCav-1**	-5502.489265	1.083765	-5503.924192	-0.063907	0.003028	-5502.901306
**(TfO•2a)⊂AuCav-2**	-5502.478955	1.082686	-5503.915056	-0.068397	0.003028	-5502.897740
**(TfO•2a)⊂AuCav-3**	-5502.481710	1.084245	-5503.917939	-0.066130	0.003028	-5502.896796
**(TfO•2a)⊂AuCav-4**	-5502.484279	1.084398	-5503.919021	-0.064371	0.003028	-5502.895966
**(TfO•2a)⊂AuCav-5**	-5502.476213	1.084769	-5503.915668	-0.066931	0.003028	-5502.894802
**(TfO•2a)⊂AuCav-6**	-5502.476274	1.083394	-5503.913440	-0.067499	0.003028	-5502.894516
**(TfO•2a)⊂AuCav-7**	-5502.471561	1.083139	-5503.909250	-0.067411	0.003028	-5502.890495
**(TfO•2a)⊂AuCav-8**	-5502.461128	1.086384	-5503.901136	-0.070733	0.003028	-5502.882458
**(TfO•3a)⊂AuCav-1**	-5502.465389	1.084500	-5503.903529	-0.068056	0.003028	-5502.884058
**(TfO•3a)⊂AuCav-2**	-5502.462893	1.084029	-5503.904023	-0.065793	0.003028	-5502.882759

Table S5. *Calculated energies (a.u.) of structures for the cycloisomerization of* ***1b***.

Species	E_{opt}	G_{qRRHO}	E_{bb}	ΔE_{SMD}	E_{SS}	G_{total}
**(TfO•1b)⊂AuCav**	-5620.433263	1.164352	-5621.902199	-0.064098	0.003028	-5620.798918
**1b-TS1$_{5anti}$⊂AuCav**	-5620.406925	1.161565	-5621.880641	-0.072693	0.003028	-5620.788741
**1b-Int$_{5anti}$⊂AuCav**	-5620.449462	1.165053	-5621.912932	-0.057860	0.003028	-5620.802712
**1b-TS2$_{5anti}$⊂AuCav**	-5620.446588	1.161474	-5621.909237	-0.059156	0.003028	-5620.803891
**(TfO•2b)⊂AuCav**	-5620.479818	1.166066	-5621.943090	-0.060705	0.003028	-5620.834701
**1b-TS1$_{5syn}$⊂AuCav**	-5620.416986	1.163980	-5621.881770	-0.067306	0.003028	-5620.782068
**1b-Int$_{5syn}$⊂AuCav**	-5620.445779	1.165215	-5621.908524	-0.060817	0.003028	-5620.801099
**1b-TS2$_{5syn}$⊂AuCav**	-5620.435490	1.160230	-5621.900858	-0.062251	0.003028	-5620.799851
**(TfO•2b')⊂AuCav**	-5620.461945	1.166762	-5621.930136	-0.063864	0.003028	-5620.824210
**1b-TS1$_6$⊂AuCav**	-5620.399684	1.163746	-5621.872468	-0.071694	0.003028	-5620.777388
**1b-Int$_6$⊂AuCav**	-5620.442758	1.167301	-5621.908065	-0.056190	0.003028	-5620.793927
**1b-TS2$_6$⊂AuCav**	-5620.431442	1.163035	-5621.893798	-0.062302	0.003028	-5620.790037
**(TfO•3b)⊂AuCav**	-5620.465537	1.168977	-5621.930905	-0.063847	0.003028	-5620.822746

Table S6. *Calculated energies (a.u.) of additional structures for the cycloisomerization of* ***1b***.

Species	E_{opt}	G_{qRRHO}	E_{bb}	ΔE_{SMD}	E_{SS}	G_{total}
**(TfO•1b)⊂AuCav-1**	-5620.415289	1.160965	-5621.888144	-0.068428	0.003028	-5620.792579
**(TfO•1b)⊂AuCav-2**	-5620.407288	1.161694	-5621.881726	-0.074541	0.003028	-5620.791545
**(TfO•1b)⊂AuCav-3**	-5620.422921	1.163126	-5621.892030	-0.065547	0.003028	-5620.791423
**(TfO•1b)⊂AuCav-4**	-5620.410437	1.161971	-5621.881106	-0.070877	0.003028	-5620.786984
**(TfO•1b)⊂AuCav-5**	-5620.405673	1.161392	-5621.876948	-0.072601	0.003028	-5620.785129
**(TfO•1b)⊂AuCav-6**	-5620.386537	1.161956	-5621.862910	-0.083077	0.003028	-5620.781003
**(TfO•1b)⊂AuCav-7**	-5620.398924	1.160464	-5621.870308	-0.071542	0.003028	-5620.778359
**(TfO•1b)⊂AuCav-8**	-5620.382092	1.160227	-5621.860503	-0.079326	0.003028	-5620.776574
**(TfO•1b)⊂AuCav-9**	-5620.370377	1.159391	-5621.848638	-0.088621	0.003028	-5620.774840
**(TfO•2b)⊂AuCav-1**	-5620.472817	1.166451	-5621.934557	-0.063508	0.003028	-5620.828587
**(TfO•2b)⊂AuCav-2**	-5620.453187	1.165358	-5621.917783	-0.063351	0.003028	-5620.812748
**(TfO•3b)⊂AuCav-1**	-5620.458379	1.166849	-5621.923166	-0.065923	0.003028	-5620.819212
**(TfO•3b)⊂AuCav-2**	-5620.440591	1.165859	-5621.906608	-0.068348	0.003028	-5620.806069
**1b⊂AuCav**	-4658.770009	1.141280	-4659.970177	-0.104508	0.003028	-4658.930377
**1b-TS1$_{5anti}$H$^+$⊂AuCav**	-4658.769587	1.143812	-4659.969115	-0.099703	0.003028	-4658.921978
**1b-Int$_{5anti}$H$^+$⊂AuCav**	-4658.775917	1.146090	-4659.972201	-0.098467	0.003028	-4658.921551
**1b-TS1$_{5syn}$H$^+$⊂AuCav**	-4658.761063	1.142038	-4659.960839	-0.100203	0.003028	-4658.915976
**1b-Int$_{5syn}$H$^+$⊂AuCav**	-4658.776337	1.145012	-4659.973945	-0.099670	0.003028	-4658.925575
**1b-TS1$_6$H$^+$⊂AuCav**	-4658.748636	1.142060	-4659.949890	-0.100895	0.003028	-4658.905698
**1b-Int$_6$H$^+$⊂AuCav**	-4658.769605	1.145731	-4659.966410	-0.099584	0.003028	-4658.917235

Table S7. *Calculated energies (a.u.) of structures for the cycloisomerization of* ***1c****.*

Species	E_{opt}	G_{qRRHO}	E_{bb}	ΔE_{SMD}	E_{SS}	G_{total}
**(TfO•1c)⊂AuCav**	-5656.108278	1.125023	-5657.589760	-0.068913	0.003028	-5656.530622
**1c-TS1$_{5anti}$⊂AuCav**	-5656.075530	1.126407	-5657.556981	-0.071563	0.003028	-5656.499109
**1c-Int$_{5anti}$⊂AuCav**	-5656.117855	1.129181	-5657.595473	-0.062259	0.003028	-5656.525523
**1c-TS2$_{5anti}$⊂AuCav**	-5656.109830	1.123790	-5657.586040	-0.061770	0.003028	-5656.520992
**(TfO•2c)⊂AuCav**	-5656.150520	1.129056	-5657.626239	-0.066834	0.003028	-5656.560988
**1c-TS1$_{5syn}$⊂AuCav**	-5656.090856	1.125989	-5657.571554	-0.067982	0.003028	-5656.510519
**1c-Int$_{5syn}$⊂AuCav**	-5656.128115	1.128252	-5657.605791	-0.063615	0.003028	-5656.538126
**1c-TS2$_{5syn}$⊂AuCav**	-5656.106252	1.123882	-5657.584847	-0.066812	0.003028	-5656.524749
**1c-TS1$_6$⊂AuCav**	-5656.064561	1.127240	-5657.551223	-0.073438	0.003028	-5656.494393
**1c-Int$_6$⊂AuCav**	-5656.111080	1.131385	-5657.588641	-0.058391	0.003028	-5656.512619
**1c-TS2$_6$⊂AuCav**	-5656.100196	1.127220	-5657.573190	-0.065343	0.003028	-5656.508285
**(TfO•3c)⊂AuCav**	-5656.137424	1.131628	-5657.618591	-0.065622	0.003028	-5656.549557

Table S8. *Calculated energies (a.u.) of additional structures for the cycloisomerization of* ***1c****.*

Species	E_{opt}	G_{qRRHO}	E_{bb}	ΔE_{SMD}	E_{SS}	G_{total}
**(TfO•1c)⊂AuCav-1**	-5656.109911	1.126785	-5657.590138	-0.065207	0.003028	-5656.525532
**(TfO•1c)⊂AuCav-2**	-5656.096246	1.127647	-5657.578012	-0.077904	0.003028	-5656.525241
**(TfO•1c)⊂AuCav-3**	-5656.098162	1.125109	-5657.580225	-0.071165	0.003028	-5656.523253
**(TfO•1c)⊂AuCav-4**	-5656.101895	1.126446	-5657.581814	-0.069828	0.003028	-5656.522168
**(TfO•1c)⊂AuCav-5**	-5656.088739	1.127348	-5657.571606	-0.080344	0.003028	-5656.521574
**(TfO•1c)⊂AuCav-6**	-5656.102814	1.126311	-5657.583060	-0.067559	0.003028	-5656.521281
**(TfO•1c)⊂AuCav-7**	-5656.089779	1.127398	-5657.571939	-0.078164	0.003028	-5656.519678
**(TfO•1c)⊂AuCav-8**	-5656.092335	1.126805	-5657.578088	-0.070111	0.003028	-5656.518366
**(TfO•1c)⊂AuCav-9**	-5656.091475	1.124552	-5657.571632	-0.066302	0.003028	-5656.510354
**(TfO•1c)⊂AuCav-10**	-5656.063646	1.124542	-5657.550408	-0.082585	0.003028	-5656.505422
**(TfO•2c)⊂AuCav-1**	-5656.140336	1.129983	-5657.618591	-0.072750	0.003028	-5656.558330
**(TfO•2c)⊂AuCav-2**	-5656.142922	1.130190	-5657.619787	-0.067703	0.003028	-5656.554272
**(TfO•2c)⊂AuCav-3**	-5656.130476	1.129860	-5657.609677	-0.073633	0.003028	-5656.550422
**(TfO•3c)⊂AuCav-1**	-5656.147379	1.131873	-5657.621408	-0.061695	0.003028	-5656.548201
**(TfO•3c)⊂AuCav-2**	-5656.129529	1.131202	-5657.607051	-0.071290	0.003028	-5656.544111
**1c⊂AuCav**	-4694.465672	1.106511	-4695.673829	-0.101968	0.003028	-4694.666258
**1c-TS1$_{5anti}$H$^+$⊂AuCav**	-4694.442360	1.108059	-4695.653790	-0.102611	0.003028	-4694.645314
**1c-Int$_{5anti}$H$^+$⊂AuCav**	-4694.449600	1.110014	-4695.658704	-0.103073	0.003028	-4694.648735
**1c-TS1$_{5syn}$H$^+$⊂AuCav**	-4694.440368	1.106387	-4695.648697	-0.104181	0.003028	-4694.643464
**1c-Int$_{5syn}$H$^+$⊂AuCav**	-4694.453502	1.108445	-4695.660659	-0.104966	0.003028	-4694.654152
**1c-TS1$_6$H$^+$⊂AuCav**	-4694.427313	1.107821	-4695.639934	-0.103160	0.003028	-4694.632245
**1c-Int$_6$H$^+$⊂AuCav**	-4694.449872	1.111336	-4695.657308	-0.100535	0.003028	-4694.643479

Table S9. *Calculated energies (a.u.) of structures for the cycloisomerization of* ***1a*** *by* ***[Au]****.*

Species	E_{opt}	G_{qRRHO}	E_{bb}	ΔE_{SMD}	E_{SS}	G_{total}
**TfO•[Au]**	-1803.541789	0.151353	-1803.956330	-0.020543	0.003028	-1803.822492
**(TfO•1a)[Au]**	-2646.415739	0.386929	-2647.089990	-0.032220	0.003028	-2646.732253
**1a-TS1$_{5anti}$[Au]**	-2646.422844	0.385592	-2647.087851	-0.029953	0.003028	-2646.729184
**1a-Int$_{5anti}$[Au]**	-2646.425102	0.388869	-2647.095726	-0.027933	0.003028	-2646.731762
**1a-TS2$_{5anti}$[Au]**	-2646.422844	0.385592	-2647.087851	-0.029953	0.003028	-2646.729184
**(TfO•2a)[Au]**	-2646.469667	0.390229	-2647.135526	-0.028532	0.003028	-2646.770801
**1a-TS1$_{5syn}$[Au]**	-2646.396763	0.386716	-2647.068821	-0.031507	0.003028	-2646.710585
**1a-Int$_{5syn}$[Au]**	-2646.439219	0.390027	-2647.106874	-0.022298	0.003028	-2646.736117
**1a-TS2$_{5syn}$[Au]**	-2646.419614	0.383779	-2647.086093	-0.035615	0.003028	-2646.734900
**1a-TS1$_6$[Au]**	-2646.379686	0.386000	-2647.053801	-0.039326	0.003028	-2646.704099
**1a-Int$_6$[Au]**	-2646.420291	0.389858	-2647.089778	-0.027489	0.003028	-2646.724381
**1a-TS2$_6$[Au]**	-2646.409053	0.385837	-2647.073683	-0.032244	0.003028	-2646.717062
**(TfO•3a)[Au]**	-2646.445865	0.389297	-2647.112617	-0.034272	0.003028	-2646.754564

Table S10. *Calculated energies (a.u.) of additional structures for the cycloisomerization of* ***1a*** *by* ***[Au]****.*

Species	E_{opt}	G_{qRRHO}	E_{bb}	ΔE_{SMD}	E_{SS}	G_{total}
**1a[Au]**	-1684.758376	0.366053	-1685.163399	-0.072653	0.003028	-1684.866972
**1a-TS1$_{5anti}$H$^+$[Au]**	-1684.755283	0.367862	-1685.157333	-0.070848	0.003028	-1684.857291
**1a-Int$_{5anti}$H$^+$[Au]**	-1684.765690	0.370703	-1685.162830	-0.067118	0.003028	-1684.856217
**1a-TS1$_{5syn}$H$^+$[Au]**	-1684.754499	0.368126	-1685.153336	-0.067123	0.003028	-1684.849305
**1a-Int$_{5syn}$H$^+$[Au]**	-1684.767545	0.370668	-1685.163999	-0.066064	0.003028	-1684.856367
**1a-TS1$_6$H$^+$[Au]**	-1684.743250	0.367710	-1685.145463	-0.070479	0.003028	-1684.845204
**1a-Int$_6$H$^+$[Au]**	-1684.767401	0.371436	-1685.163378	-0.065991	0.003028	-1684.854906

Table S11. *Calculated energies (a.u.) of structures for the cycloisomerization of* ***1a*** *by* ***[Au]*** *using geometry optimization in solution.*

Species	$E_{opt, SMD}$	G_{qRRHO}	$E_{bb, SMD}$	E_{SS}	G_{total}
**SolvOpt-TfO•[Au]**	-1803.565124	0.149789	-1803.981199	0.003028	-1803.828383
**SolvOpt-(TfO•1a)[Au]**	-2646.450127	0.386069	-2647.128160	0.003028	-2646.739063
**SolvOpt-1a-TS1$_{5anti}$[Au]**	-2646.430941	0.385185	-2647.110988	0.003028	-2646.722775
**SolvOpt-1a-Int$_{5anti}$[Au]**	-2646.454111	0.387797	-2647.125090	0.003028	-2646.734265
**SolvOpt-1a-TS2$_{5anti}$[Au]**	-2646.454683	0.384392	-2647.121925	0.003028	-2646.734506
**SolvOpt-(TfO•2a)[Au]**	-2646.499610	0.389420	-2647.168268	0.003028	-2646.775820
**SolvOpt-(1a)$_2$**	-1685.751797	0.448928	-1686.276194	0.003028	-1685.824238

XI. Cartesian coordinates

$CHCl_3$

C	0.00000000	0.00000000	0.45481700
H	0.00000000	0.00000000	1.54034300
Cl	0.00000000	1.70269300	-0.08371100
Cl	-1.47457500	-0.85134600	-0.08371100
Cl	1.47457500	-0.85134600	-0.08371100

1a

C	-0.50124400	2.03301900	0.49070800
O	-0.46616800	2.97467500	-0.27267200
O	-0.15898000	2.14613500	1.76809800
H	-0.28025000	1.26618400	2.20801300
C	-0.98339400	0.65044900	-0.04568500
C	-2.47411200	0.88608300	-0.47227100
H	-3.06395800	1.12563500	0.42158200
H	-2.46299600	1.78939900	-1.09067900
C	-3.10229300	-0.20760300	-1.20232600
C	-3.64032100	-1.09417600	-1.81965100
H	-4.11583500	-1.87582900	-2.36492600
C	-0.91625200	-0.47299800	0.98489000
O	-0.69705400	-0.32608000	2.18280700
O	-1.14578100	-1.66671300	0.44968900
C	-1.07985400	-2.78940100	1.35092400
H	-1.78570300	-2.65882500	2.17287600
H	-1.34148100	-3.65681400	0.74742800
H	-0.06815400	-2.88284300	1.75109900
C	-0.11489100	0.28869100	-1.28412700
H	-0.58249800	-0.55777100	-1.78966300
H	-0.15884200	1.15269700	-1.95130300
C	1.31631300	-0.03445700	-0.91780000
C	1.73691700	-1.36553500	-0.79796000
C	2.23806200	0.98858600	-0.65350300
C	3.04265300	-1.67054100	-0.41279800
H	1.03368800	-2.16405500	-1.01421500
C	3.54274600	0.68480500	-0.26619100
H	1.92956000	2.02422500	-0.75480400
C	3.94863300	-0.64485800	-0.14123000
H	3.35312800	-2.70815800	-0.33032000
H	4.24401300	1.48914900	-0.06533200
H	4.96540900	-0.87952300	0.15899000

1b

C	0.24702200	-1.44215700	0.62882000
O	0.54515500	-1.66039800	1.77929500
O	0.04876300	-2.43169500	-0.27644900
H	0.18708100	-3.26632800	0.20175600
C	0.06023700	-0.06484700	-0.00305500
C	0.17360400	0.99292500	1.12594500
H	-0.52989300	0.73005600	1.91934600
H	1.17695700	0.92893400	1.55654000
C	-0.09034300	2.34421400	0.64358800
C	-0.32009900	3.44979000	0.21522800
H	-0.52272500	4.42852300	-0.15269500
C	-1.35198300	0.00159900	-0.61484700
O	-1.58167600	0.25336400	-1.77606000
O	-2.26132600	-0.23828100	0.34114200
C	1.09989800	0.16005600	-1.13321900
H	0.91100900	-0.57958500	-1.91323600
H	0.88647900	1.13984600	-1.56571700
C	2.53064700	0.08642800	-0.65897700
C	3.21028300	-1.13787100	-0.62126000
C	3.19949100	1.23893500	-0.22547600
C	4.52255400	-1.21163900	-0.15534400
H	2.70385500	-2.03601400	-0.96337900
C	4.51191000	1.16804600	0.24165200
H	2.68227700	2.19410000	-0.25522200
C	5.17708400	-0.05825500	0.27922900
H	5.03536400	-2.16881600	-0.13513500
H	5.01586000	2.07129200	0.57291300
H	6.19952300	-0.11416500	0.64055000
C	-3.70885700	-0.13703100	0.06958300
C	-4.04083800	1.28573300	-0.38246000
C	-4.11098200	-1.19313600	-0.95984500
C	-4.32260500	-0.43648900	1.43554800
H	-3.63469100	2.01059200	0.32903800
H	-3.62579400	1.49313500	-1.36852600
H	-5.12738300	1.40993000	-0.42167700
H	-3.79238500	-2.18534700	-0.62672200
H	-5.20008300	-1.20088600	-1.06698400
H	-3.66218200	-0.98454300	-1.93081500
H	-5.41371400	-0.40238100	1.37015100
H	-4.02453700	-1.43000300	1.78152600
H	-3.99448700	0.29995000	2.17421000

1c

C	1.83387400	1.59243000	-0.60332000
O	2.12925300	2.61124800	-0.01966900
O	1.72789400	1.52201900	-1.94770100
H	1.92624000	2.41110500	-2.28646700
C	1.54867000	0.23652200	0.04405700
C	1.71524400	0.36434300	1.58481700
H	2.66815200	0.85738100	1.79163300
H	0.92822300	1.01421200	1.97471100
C	1.65889300	-0.93514200	2.24582000
C	1.61380400	-2.02622400	2.76026100
H	1.57152900	-2.98328100	3.22563700
C	2.63155400	-0.73304600	-0.47571400
O	2.42794700	-1.78372000	-1.03521700
O	3.85832300	-0.24746900	-0.20370900
C	4.95370800	-1.08991500	-0.60152800
H	4.92556400	-1.26747000	-1.67894200
H	5.85635400	-0.54848300	-0.32149200
H	4.89985900	-2.04831400	-0.08030800
C	0.18224100	-0.34710300	-0.36704700
H	0.21074400	-0.52575400	-1.44604300
H	0.14351600	-1.33835900	0.09171700
C	-1.08153500	0.41169100	-0.01722100
C	-2.33448700	-0.27031900	-0.19921400
C	-1.09907600	1.71621600	0.43523600
C	-2.41958000	-1.60373500	-0.68614800
C	-3.55815100	0.41029000	0.10911800
C	-2.31027200	2.38547800	0.73644500
H	-0.17766200	2.26686300	0.57272800
C	-3.63614200	-2.22927000	-0.85029700
H	-1.51460900	-2.14218900	-0.94068000
C	-4.79458300	-0.26362100	-0.06768100
C	-3.51449900	1.74831000	0.58207400
H	-2.27127200	3.41008200	1.09354900
C	-4.83827000	-1.55613500	-0.53652700
H	-3.67166300	-3.24794800	-1.22462100
H	-5.71158600	0.26700900	0.17426000
H	-4.44790500	2.25359500	0.81405900
H	-5.79116600	-2.05969500	-0.66816000

$(1a)_2$

C	1.87149300	-0.84440100	0.15524600
O	1.32994000	-1.00388900	-0.93832600
O	1.23170000	-0.76531000	1.30152500
H	0.23670700	-0.88106000	1.16431700
C	3.37839000	-0.65973900	0.30158100
C	4.06124200	-1.02256400	-1.04339400
H	3.78515500	-2.04582300	-1.31203400
H	3.65177300	-0.36931200	-1.81875500
C	5.51285500	-0.89072100	-0.98194500
C	6.71204800	-0.77240900	-0.90576100
H	7.77120600	-0.67827300	-0.84391300
C	3.88229100	-1.60314300	1.40126300
O	4.50985900	-1.27347800	2.37899200
O	3.54230400	-2.87611500	1.11328300
C	3.97362900	-3.85745900	2.07182600
H	3.54261900	-3.64426100	3.05259200
H	3.61706700	-4.81445800	1.69296700

H	5.06295700	-3.85458600	2.15247100
C	3.66540600	0.80647400	0.73338900
H	3.19321200	0.96398300	1.70507100
H	4.74390300	0.88443500	0.88367800
C	3.18830600	1.82872900	-0.26949900
C	1.87075000	2.30574100	-0.23612200
C	4.04692500	2.29807500	-1.27171900
C	1.42175800	3.22625400	-1.18322900
H	1.19664300	1.95399000	0.53993900
C	3.59961300	3.21746900	-2.22112800
H	5.06961700	1.93333700	-1.30605000
C	2.28548800	3.68365100	-2.17987400
H	0.40050800	3.59182900	-1.14252500
H	4.27932500	3.57121300	-2.99072500
H	1.93632100	4.40122300	-2.91606200
C	-1.87197900	-0.84420500	-0.15573100
O	-1.33038300	-1.00428900	0.93773100
O	-1.23230500	-0.76487100	-1.30206600
H	-0.23729500	-0.88075600	-1.16501100
C	-3.37878800	-0.65896600	-0.30176700
C	-4.06152600	-1.02166400	1.04335000
H	-3.78570200	-2.04503000	1.31187100
H	-3.65164900	-0.36859200	1.81865100
C	-5.51309800	-0.88934000	0.98228100
C	-6.71227500	-0.77064800	0.90646100
H	-7.77141000	-0.67601900	0.84499200
C	-3.88343700	-1.60197400	-1.40139300
O	-4.51121400	-1.27189000	-2.37885500
O	-3.54391200	-2.87514200	-1.11373400
C	-3.97610000	-3.85616300	-2.07222200
H	-3.54545300	-3.64301100	-3.05315600
H	-3.61977300	-4.81336800	-1.69366100
H	-5.06546400	-3.85280600	-2.15236000
C	-3.66539500	0.80746400	-0.73316000
H	-3.19369800	0.96502500	-1.70508000
H	-4.74394800	0.88583800	-0.88281600
C	-3.18738500	1.82922800	0.26976100
C	-1.86970800	2.30577700	0.23583000
C	-4.04530900	2.29861500	1.27257500
C	-1.41988400	3.22587300	1.18293900
H	-1.19609000	1.95410400	-0.54069500
C	-3.59716000	3.21756600	2.22201900
H	-5.06809800	1.93419100	1.30737600
C	-2.28287900	3.68331300	2.18018500
H	-0.39856000	3.59113600	1.14151400
H	-4.27634600	3.57134100	2.99206700
H	-1.93307400	4.40057800	2.91636900

(1b)$_2$

C	-1.87710900	-0.13778900	0.05721500
O	-1.21520500	-0.30245200	1.08198100
O	-1.36962600	-0.05940200	-1.15406800
H	-0.36578300	-0.17751500	-1.12895200
C	-3.38924200	0.04859000	0.08120200
C	-3.91707300	-0.31222600	1.49420700
H	-3.58656900	-1.32440100	1.74111200
H	-3.44993500	0.36138500	2.21785400
C	-5.37089000	-0.22354700	1.57853700
C	-6.57536100	-0.14686300	1.61773000
H	-7.63784700	-0.08514600	1.65902700
C	-4.01095400	-0.90835100	-0.95068900
O	-4.73895800	-0.56345500	-1.85321600
O	-3.63234800	-2.16355400	-0.66692100
C	-3.72728300	1.51073200	-0.32153800
H	-3.36816600	1.66528400	-1.34088600
H	-4.81615700	1.58523800	-0.35040200
C	-3.14403800	2.53820300	0.61755100
C	-1.83793400	3.01460100	0.43914700
C	-3.88828100	3.01174300	1.70557500
C	-1.28851100	3.93840400	1.32832800
H	-1.25216500	2.65946800	-0.40421300
C	-3.34050700	3.93467900	2.59717000
H	-4.90126800	2.64728300	1.85226600
C	-2.03863100	4.40009900	2.41129700
H	-0.27757200	4.30295700	1.17538600
H	-3.93237900	4.29164700	3.43486100
H	-1.61136300	5.12024100	3.10242600
C	-4.10550200	-3.31043800	-1.46688800
C	-5.62839300	-3.40398500	-1.36721100
C	-3.61378000	-3.16353300	-2.90688600
C	-3.43457100	-4.49475900	-0.77435100
H	-5.93771600	-3.40777600	-0.31787600
H	-6.10738800	-2.56481900	-1.87114600
H	-5.96653300	-4.33606400	-1.83034500
H	-2.53030400	-3.01346600	-2.92113100
H	-3.84251800	-4.07697000	-3.46448900
H	-4.09335000	-2.31901900	-3.40136000
H	-3.70472300	-5.42553800	-1.28088400
H	-2.34660300	-4.38760500	-0.79859900
H	-3.75353200	-4.56106500	0.26954400
C	1.87707300	-0.13774300	-0.05717300
O	1.21514400	-0.30229500	-1.08193800
O	1.36959700	-0.05916800	1.15410600
H	0.36577300	-0.17731100	1.12902000
C	3.38923500	0.04839200	-0.08116900
C	3.91700000	-0.31250200	-1.49417800
H	3.58636900	-1.32463500	-1.74107400
H	3.44994300	0.36116800	-2.21782200
C	5.37082700	-0.22399500	-1.57850700
C	6.57530900	-0.14745100	-1.61769300
H	7.63780200	-0.08586200	-1.65899400
C	4.01078600	-0.90865900	0.95072700
O	4.73878800	-0.56388200	1.85329700
O	3.63199800	-2.16380100	0.66692400
C	3.72753100	1.51047200	0.32157000
H	3.36843600	1.66509600	1.34091400
H	4.81641800	1.58479400	0.35044700
C	3.14446700	2.53802400	-0.61753900
C	1.83841800	3.01458300	-0.43917600
C	3.88879400	3.01145200	-1.70555400
C	1.28912000	3.93843300	-1.32838400
H	1.25259300	2.65952200	0.40417300
C	3.34115100	3.93444300	-2.59717400
H	4.90174100	2.64686600	-1.85221500
C	2.03932400	4.40002000	-2.41134100
H	0.27821400	4.30309400	-1.17547400
H	3.93308600	4.29132800	-3.43485500
H	1.61215800	5.12019900	-3.10249600
C	4.10500100	-3.31077000	1.46683200
C	5.62788700	-3.40447700	1.36719100
C	3.61326500	-3.16390400	2.90683300
C	3.43396500	-4.49498800	0.77421500
H	5.93723800	-3.40825500	0.31786400
H	6.10695900	-2.56538400	1.87117400
H	5.96591300	-4.33660900	1.83029800
H	2.52980600	-3.01370600	2.92106300
H	3.84187800	-4.07740600	3.46437900
H	4.09292500	-2.31948200	3.40137500
H	3.70403200	-5.42582300	1.28068900
H	2.34600700	-4.38773900	0.79846400
H	3.75292700	-4.56125200	-0.26968200

(1c)$_2$

C	-2.59467300	-1.68418700	-0.06717200
O	-2.27176500	-2.48807000	0.80494000
O	-2.06625200	-1.65673100	-1.27733000
H	-1.29427100	-2.29121800	-1.32483600
C	-3.62594000	-0.57903600	0.14087800
C	-4.30187100	-0.73533300	1.53206200
H	-4.63262400	-1.77075600	1.64632000
H	-3.56222500	-0.54761400	2.31353100
C	-5.42986200	0.17701200	1.68955700
C	-6.36062100	0.93950300	1.78552100
H	-7.18163200	1.61118600	1.88152200
C	-4.70824500	-0.76108200	-0.94259100
O	-5.04237300	0.06714000	-1.75488900
O	-5.24845400	-1.99144600	-0.84829000
C	-6.30043300	-2.27381100	-1.78768800
H	-5.93210600	-2.17480500	-2.81112400
H	-6.60773900	-3.29868100	-1.58408400

H	-7.13317500	-1.58293400	-1.63841500
C	-2.98744900	0.81128400	-0.07277100
H	-2.73117000	0.88189800	-1.13311000
H	-3.79432700	1.53438100	0.08202100
C	-1.77778800	1.21855400	0.74678700
C	-1.24377600	2.53292800	0.51278300
C	-1.14914100	0.40853300	1.67136900
C	-1.82208600	3.44148100	-0.41440700
C	-0.07388800	2.95461300	1.22431600
C	0.00360500	0.83141700	2.37668700
H	-1.51776300	-0.58558800	1.87748700
C	-1.28016300	4.69139900	-0.62266400
H	-2.70440800	3.15244800	-0.97277600
C	0.46152500	4.24512600	0.98465400
C	0.53901000	2.07286600	2.15243500
H	0.46067900	0.15265100	3.09012900
C	-0.12657800	5.10043800	0.08217700
H	-1.74333500	5.36670500	-1.33604800
H	1.36315300	4.53647000	1.51475500
H	1.43772700	2.39794900	2.66770400
H	0.29707800	6.08431400	-0.09529600
C	0.89073300	-2.62355300	-0.18739400
O	0.26604200	-2.85047800	-1.22193700
O	0.35498800	-2.66305000	1.01993800
H	-0.63006800	-2.80832300	0.94841300
C	2.36318100	-2.22888300	-0.16256600
C	2.96277300	-2.37152800	-1.58779500
H	2.78990600	-3.39146800	-1.94164400
H	2.41979100	-1.70284800	-2.25990100
C	4.38684300	-2.05625000	-1.61018600
C	5.56380300	-1.78964500	-1.59550400
H	6.60132300	-1.54937800	-1.59439100
C	3.07706200	-3.22393600	0.77342000
O	3.77976400	-2.93609100	1.71229100
O	2.82088100	-4.48775400	0.38121800
C	3.45007700	-5.51510800	1.16675800
H	3.12386500	-5.44989500	2.20715200
H	3.13601800	-6.45818000	0.72143800
H	4.53646800	-5.41003200	1.12561200
C	2.56439400	-0.80895900	0.42757600
H	2.04382200	-0.76674700	1.38915000
H	3.62689500	-0.76294100	0.65814800
C	2.17375300	0.38509000	-0.41912100
C	2.91614000	1.60754800	-0.27965000
C	1.09437000	0.36552900	-1.28085000
C	3.98529400	1.76760800	0.64575100
C	2.55266200	2.74244800	-1.07824700
C	0.73040200	1.49141400	-2.05398800
H	0.49494900	-0.52656200	-1.38992700
C	4.65813300	2.96314100	0.76717800
H	4.27222100	0.94239900	1.28585700
C	3.27755900	3.95509400	-0.94148500
C	1.45280000	2.65299200	-1.96834600
H	-0.13138500	1.42499500	-2.71047900
C	4.30781900	4.07093200	-0.03882700
H	5.46562700	3.05521800	1.48744700
H	2.98379900	4.80103100	-1.55672300
H	1.17489600	3.52720600	-2.54849200
H	4.84883600	5.00709900	0.06108700

2a

C	-0.77927700	0.63816100	1.10622700
O	-0.12054600	1.78642200	0.77243200
O	-1.12640000	0.39087500	2.22910800
C	-0.92090500	-0.25971000	-0.13558300
C	-0.53917700	0.70011100	-1.28379900
H	0.06817800	0.22452700	-2.05460000
H	-1.44086200	1.10814700	-1.74977700
C	0.21318000	1.80198600	-0.57957100
C	1.06801400	2.69692300	-1.05577400
H	1.32165200	2.69559900	-2.10839200
C	-2.36321800	-0.74912800	-0.23159900
O	-2.70464200	-1.90680700	-0.30878200
O	-3.21900600	0.29160800	-0.22852400
C	-4.61411900	-0.05609400	-0.27711500
H	-4.87918500	-0.67204700	0.58482000
H	-5.15245600	0.89040400	-0.25486200
H	-4.83798300	-0.60827200	-1.19278800
C	0.02857700	-1.47861300	0.01546800
H	-0.25665500	-2.00073400	0.93279400
H	-0.17972900	-2.15791600	-0.81480800
C	1.48833100	-1.09545300	0.04185600
C	2.08224400	-0.61610000	1.21735500
C	2.26493600	-1.16759100	-1.12090200
C	3.41108800	-0.19577300	1.22100400
H	1.49705300	-0.56618200	2.13085500
C	3.59546400	-0.74960300	-1.12004400
H	1.82294800	-1.56072600	-2.03311200
C	4.17024500	-0.25579100	0.05117300
H	3.85440600	0.17731300	2.13928900
H	4.18257900	-0.81263400	-2.03147500
H	5.20538800	0.07190000	0.05499800
H	1.52711800	3.43388200	-0.40898500

3a

C	-0.77785000	1.09249000	0.65081300
O	0.00757200	2.07882700	0.13567300
O	-1.29953600	1.26652700	1.72416200
C	-0.89794900	-0.23502100	-0.11290900
C	-0.64706500	-0.09110500	-1.63671300
H	-0.35693500	-1.06783200	-2.03806100
H	-1.58332900	0.18592100	-2.13534400
C	0.39921600	0.93920700	-1.93402100
C	0.65556400	1.92158800	-1.08042000
C	-2.32998900	-0.73765000	0.11801700
O	-2.62762100	-1.80237000	0.60815700
O	-3.22676800	0.15813400	-0.33660500
C	-4.60525100	-0.19081400	-0.12539100
H	-4.80611800	-0.29891600	0.94272100
H	-5.18330700	0.63195800	-0.54415700
H	-4.84440400	-1.12950800	-0.63057400
C	0.07610300	-1.25047700	0.55881500
H	-0.21046700	-1.32515400	1.61060800
H	-0.12598200	-2.22534700	0.10746300
C	1.53588700	-0.89512800	0.42892200
C	2.12951100	0.01025400	1.31770400
C	2.31628700	-1.43494600	-0.60010100
C	3.46017900	0.39410400	1.15808100
H	1.54138800	0.42347700	2.13215800
C	3.64921200	-1.05820000	-0.75961500
H	1.87317100	-2.15593100	-1.28222300
C	4.22281700	-0.13465500	0.11515400
H	3.90336400	1.10181900	1.85234100
H	4.23983600	-1.48669400	-1.56408000
H	5.26027200	0.16173600	-0.00706000
H	1.39062700	2.70293200	-1.22029600
H	0.95266100	0.89471700	-2.86460600

2b

C	3.72719200	0.15070000	-0.07915200
C	4.97632700	0.54346800	-0.29827000
H	5.43570700	0.37141800	-1.26373400
C	2.74163500	-0.53710200	-0.98188700
C	1.38619800	-0.14636000	-0.37044500
H	2.85980300	-1.62440900	-0.93666600
H	2.84989700	-0.23765800	-2.02556700
C	1.76668200	0.12781200	1.09666100
O	3.12339300	0.35531200	1.15852100
O	1.07830400	0.19481500	2.07600600
C	0.88042100	1.18771500	-1.03180600
H	1.65350700	1.94268300	-0.84735000
H	0.87612200	1.00190000	-2.11051300
C	0.35277000	-1.26445000	-0.52056200
O	0.51856900	-2.21049500	-1.26777600
O	-0.70602900	-1.02727600	0.24062100
C	-1.86260900	-1.94097500	0.30806600
H	5.55823100	1.02442800	0.47829300
C	-0.47371700	1.70608400	-0.61495800
C	-1.56814600	1.54467600	-1.47232600

C	-0.66931500	2.35475400	0.60971600
C	-2.83521700	2.00235300	-1.11221700
H	-1.42535700	1.04964900	-2.42953100
C	-1.93535000	2.80831200	0.97622200
H	0.16497000	2.49122700	1.28861200
C	-3.02323400	2.63212200	0.11907800
H	-3.67173000	1.86969700	-1.79200900
H	-2.07170300	3.30232300	1.93341100
H	-4.00762400	2.99022300	0.40503800
C	-2.78078600	-1.21941800	1.29180600
H	-3.05281600	-0.23425300	0.90525300
H	-3.69148300	-1.80441200	1.45041500
H	-2.27438300	-1.08354100	2.25076200
C	-1.40097200	-3.29007000	0.85821200
H	-2.27203000	-3.92941900	1.03127700
H	-0.73142800	-3.79142600	0.15837200
H	-0.88337300	-3.14992300	1.81157300
C	-2.51204800	-2.06165100	-1.07050100
H	-1.84003700	-2.54294200	-1.78144300
H	-3.42425200	-2.66051000	-0.98662700
H	-2.78490500	-1.07171600	-1.44437600

3b

C	3.38443800	0.63005100	0.83928900
C	3.78804500	0.07433600	-0.29997100
H	4.59788500	0.42770800	-0.92487700
C	2.21837300	0.03782200	1.57538500
C	1.24802300	-0.63029100	0.57203600
H	1.66461700	0.80147100	2.12581300
H	2.55050800	-0.71113600	2.30862300
C	2.04401100	-1.56111700	-0.35394200
O	3.24038600	-1.08457800	-0.81704100
O	1.69716200	-2.66445400	-0.68878800
H	3.88850800	1.51457500	1.21002900
C	0.13985500	-1.41416900	1.33542200
H	0.50740200	-2.43471900	1.47828700
H	0.04208900	-0.96051500	2.32513900
C	0.63424300	0.45424700	-0.33035200
O	0.75486400	0.46977100	-1.53704000
O	-0.03761400	1.33646700	0.41319100
C	-0.80591800	2.43919400	-0.20771600
C	-1.22969800	-1.44365200	0.69044400
C	-1.43380800	-1.98134400	-0.58700800
C	-2.32740700	-0.91341200	1.37857300
C	-2.70563600	-1.97883400	-1.15883500
H	-0.59254000	-2.39919800	-1.12730900
C	-3.60230300	-0.92068200	0.81303200
H	-2.17878400	-0.48733800	2.36715700
C	-3.79421500	-1.45112800	-0.46313000
H	-2.84578700	-2.39454300	-2.15203800
H	-4.44118300	-0.50775500	1.36546000
H	-4.78322300	-1.45370300	-0.91109000
C	-1.48046400	3.08408400	1.00021800
H	-2.08076100	3.94001300	0.67975500
H	-2.13592000	2.36345000	1.49615700
H	-0.73457200	3.43258200	1.72019300
C	0.17133000	3.39991300	-0.88331000
H	-0.37146300	4.27813100	-1.24574000
H	0.92936400	3.73616000	-0.16950800
H	0.66631100	2.91970500	-1.72763900
C	-1.84672200	1.87906600	-1.17697200
H	-2.48671400	2.69878700	-1.51849500
H	-1.37430100	1.41790000	-2.04294200
H	-2.47270800	1.13628400	-0.67853800

2c

C	-1.65521100	-2.54915800	-0.25049200
C	-1.55705700	-3.81700400	-0.63351100
H	-2.07075600	-4.15513600	-1.52484000
C	-2.39131200	-1.39391100	-0.87890700
C	-1.54128200	-0.18703900	-0.41748200
H	-3.40127900	-1.32715000	-0.46068400
H	-2.46505300	-1.47869000	-1.96450400
C	-0.96455700	-0.71271800	0.90787400
O	-1.01226200	-2.09121000	0.89237500
O	-0.50685600	-0.09640100	1.82480900
C	-0.40877300	0.11080600	-1.44561200
H	-0.11547800	-0.83820400	-1.90028300
H	-0.87180900	0.71325100	-2.23053500
C	-2.39719300	1.05070700	-0.17272800
O	-2.36373300	2.06997500	-0.82640300
O	-3.24915500	0.84327500	0.84608600
C	-4.10453100	1.95200900	1.17487100
H	-0.98414100	-4.53332800	-0.05763400
C	0.80228000	0.83383000	-0.90276400
C	1.92656500	0.10784900	-0.39036900
C	0.82243400	2.21456300	-0.89375400
C	1.99646000	-1.31267200	-0.36808700
C	3.04510500	0.84277200	0.12758200
C	1.92577100	2.93289200	-0.38153400
H	-0.03808500	2.75250700	-1.27607700
C	3.09737800	-1.96660400	0.13767800
H	1.17037000	-1.90388800	-0.74461400
C	4.16548700	0.13711400	0.63929900
C	3.01396100	2.26124300	0.12016200
H	1.90527500	4.01843800	-0.38795100
C	4.19554400	-1.23762700	0.64806000
H	3.12089100	-3.05210800	0.14684500
H	5.00316700	0.70982000	1.02822500
H	3.86726800	2.80356600	0.51821700
H	5.05767400	-1.76493600	1.04487500
H	-4.70571100	1.61676100	2.01877300
H	-3.50372600	2.82141200	1.44976100
H	-4.73895900	2.21025300	0.32377100

3c

C	3.61258500	-1.52856500	-0.37598200
C	4.16028000	-0.61166400	0.41884400
H	4.98389800	-0.77705800	1.10155400
C	2.44252500	-1.15303200	-1.23984300
C	1.63772600	-0.03056300	-0.54855600
H	1.77988000	-2.00604500	-1.39992300
H	2.77102200	-0.80822300	-2.23076000
C	2.60424800	1.10275900	-0.17088100
O	3.77176200	0.71493200	0.43230000
O	2.42406100	2.27509100	-0.37359400
H	3.99978300	-2.54057900	-0.36967000
C	0.49906700	0.50178600	-1.47581300
H	0.92258000	1.31969700	-2.06261500
H	0.24475900	-0.29464900	-2.17649500
C	1.04157000	-0.54495500	0.76916900
O	1.09404400	0.04433000	1.82430100
O	0.40534900	-1.71423300	0.58538100
C	-0.37241300	-2.17201300	1.70649900
C	-0.74098700	0.99540900	-0.75544200
C	-1.89895400	0.16432000	-0.59402200
C	-0.73789600	2.26441900	-0.20787900
C	-2.00703200	-1.14991800	-1.13012800
C	-3.01680500	0.67274600	0.15166200
C	-1.84300700	2.76100300	0.51636500
H	0.14863700	2.87755900	-0.32172000
C	-3.13409600	-1.91555500	-0.93650800
H	-1.17857300	-1.57102700	-1.68495100
C	-4.16488300	-0.14459200	0.33480800
C	-2.95896000	1.98038300	0.69726600
H	-1.79905400	3.76195000	0.93433300
C	-4.22739300	-1.41198000	-0.19316200
H	-3.18508100	-2.91517400	-1.35799200
H	-4.99818600	0.25761200	0.90469600
H	-3.81227600	2.34974800	1.25944100
H	-5.11087300	-2.02559600	-0.04555200
H	0.27151300	-2.33966400	2.57213300
H	-1.13433700	-1.43026600	1.95424200
H	-0.83887300	-3.09917500	1.37778200

TfO^-

S	-0.92307900	-0.00000800	-0.00001200
O	-1.23890200	0.72832600	1.24943800

O	-1.23894400	0.71788100	-1.25545400
O	-1.23894300	-1.44619600	0.00603300
C	0.93722800	0.00002000	0.00003400
F	1.43999300	1.25459300	-0.00558900
F	1.43999300	-0.62246800	1.08929200
F	1.44003700	-0.63213400	-1.08371800

TfOH

S	-0.85056400	-0.14658800	0.07716700
O	-1.25950500	0.19782400	1.43083000
O	-1.24260900	1.08466800	-0.91416700
O	-1.21387200	-1.37952700	-0.58712400
C	1.00358500	0.00798500	-0.00100300
F	1.35457400	1.22880200	0.40824100
F	1.53527100	-0.90662800	0.80477500
F	1.42100000	-0.18595400	-1.24688900
H	-1.48220900	1.84779700	-0.36009800

AgOTf

S	0.71203400	0.84552400	-0.00000400
O	-0.13608300	0.58528100	1.22839600
O	1.52500400	2.05354100	0.00010300
O	-0.13606500	0.58550300	-1.22847200
C	1.88223700	-0.59870900	-0.00001600
F	2.64952800	-0.57129200	1.08832100
F	1.16852500	-1.74147300	-0.00016200
F	2.64978900	-0.57119600	-1.08817500
Ag	-1.93445500	-0.20798000	0.00000200

AgCl

Ag	0.00000000	0.00000000	0.61711600
Cl	0.00000000	0.00000000	-1.70614500

$(TfO\bullet CHCl_3)\subset AuCav$

C	0.36517500	4.98758100	2.04066500
C	0.26829100	4.73288400	0.53934900
C	0.38083400	3.67623900	2.82506100
H	-0.55574200	5.49884500	2.32288800
C	1.39488700	4.43355800	-0.23372700
C	-0.96886000	4.75553100	-0.11775700
C	1.56004100	2.98743400	3.12498900
C	-0.81845500	3.11795700	3.28811400
C	1.33098400	4.15549700	-1.60231600
H	2.36223700	4.40625200	0.25532600
C	-1.08563400	4.47924600	-1.47581100
O	-2.12498700	5.03910700	0.61628200
C	1.58002200	1.80566000	3.87354600
H	2.50104700	3.39258100	2.77031800
C	-0.85185700	1.94800500	4.03632500
O	-2.00587000	3.80454100	3.02464500
C	2.57731600	3.86993500	-2.43761900
C	0.06193200	4.17051800	-2.19469100
H	-2.05607100	4.46996100	-1.95348500
C	-2.94426100	3.95409000	0.82612700
C	2.87280000	1.06478700	4.20608100
C	0.35048800	1.31331200	4.32463900
H	-1.79129200	1.53406500	4.37630300
C	-2.84408700	3.26978000	2.08379400
H	2.29378600	4.05117500	-3.47503600
C	2.98436600	2.40175100	-2.36235500
O	-0.04488700	3.89409300	-3.56327600
C	3.25403200	0.09129900	3.09409800
H	2.65531300	0.45299300	5.08222800
O	0.34828200	0.17059900	5.13126800
C	3.85681300	1.91303200	-1.38660500
C	2.49513500	1.48230900	-3.30051000
C	-0.56871800	2.67138700	-3.88668400
C	-4.46947000	2.42015100	0.11870300
C	4.02313100	0.48210100	1.99533200
C	2.83714200	-1.24629600	3.13916600
C	-0.16584900	-0.96488500	4.56690800
C	-4.33594600	1.71425600	1.35236600
C	4.25632400	0.57402500	-1.32045900
H	4.24835300	2.60456200	-0.65072600
C	2.84978900	0.13756400	-3.26885700
O	1.67686000	1.95119700	-4.32165100
C	0.34430000	1.63252800	-4.27354700
C	4.39149500	-0.39516800	0.97077500
H	4.34632900	1.51428700	1.93301900
C	3.18011000	-2.15713300	2.14558200
O	2.09705300	-1.67919900	4.23176300
C	0.75983900	-1.92869700	4.04244200
C	5.19963100	0.05955700	-0.23908600
C	3.72299300	-0.29338300	-2.27876400
H	2.45158300	-0.54952800	-4.00413200
C	-2.31093800	1.24251800	-4.20863900
C	3.94912400	-1.71698400	1.07669900
H	2.83535400	-3.18104400	2.20743700
C	-1.89714000	-2.27670600	3.90478300
H	5.68832300	-0.82987300	-0.64456300
O	4.09855600	-1.65138100	-2.29317700
C	-1.40119500	0.20829600	-4.58774700
O	4.28173400	-2.65463600	0.07720600
C	-0.97805100	-3.20608000	3.32934700
C	-5.04396900	0.50594300	1.54359300
C	-5.32711400	1.91387800	-0.88471700
C	-1.90292800	-1.05695800	-4.96797500
C	-3.70004700	0.98272500	-4.21187100
C	-1.47547600	-4.33731300	2.64389800
C	-3.28552500	-2.51742200	3.81048700
C	6.31022300	1.05702500	0.11813800
H	6.98800400	0.61286700	0.85190700
H	5.92753900	1.98656900	0.54664000
C	3.73455100	4.82280400	-2.10124900
H	4.59081700	4.62381700	-2.75208600
H	4.07631500	4.73182200	-1.06641800
C	1.53815600	5.91031500	2.40417000
H	1.44446500	6.86237700	1.87431900
H	2.51222600	5.48551300	2.14478000
C	4.02155900	2.01420500	4.57797200
H	3.72736400	2.64028300	5.42458800
H	4.30607800	2.68338900	3.76091500
H	4.90991600	1.44174600	4.85994500
H	1.54181800	6.10828200	3.47963300
H	3.41738600	5.85837800	-2.24938900
H	6.88367200	1.31760900	-0.77556600
C	-3.74484000	-3.63677300	3.15133300
C	-2.83633600	-4.54577000	2.55864800
H	-3.21887500	-5.40806900	2.02352600
H	-4.81027800	-3.81987900	3.06707700
H	-0.76330400	-5.02239700	2.19687900
H	-3.95742200	-1.79373500	4.25865500
C	-6.00929200	0.73606500	-0.67127600
H	-5.41327200	2.47355400	-1.80945400
C	-5.86316800	0.02693900	0.54544900
H	-4.89283600	-0.03020600	2.47292300
C	-4.16501400	-0.25636200	-4.59806900
H	-4.36777600	1.78127900	-3.91054600
C	-3.26316900	-1.27870900	-4.97998500
H	-1.19318600	-1.82677100	-5.25062300
H	-3.65066500	-2.25025300	-5.26784300
H	-5.23135400	-0.45773200	-4.60262000
H	-6.38770700	-0.91283400	0.67993300
H	-6.65462200	0.33457000	-1.44614800
P	3.36688400	-2.70748400	-1.28842700
N	3.74115700	-4.14406600	-2.01314800
C	5.10850200	-4.40071400	-2.48092900
C	3.00508600	-5.33695500	-1.58393800
H	5.55418000	-3.47624300	-2.84705100
H	5.06483100	-5.11627800	-3.30704400
H	5.73582300	-4.81628200	-1.68214800
H	1.98472900	-5.06628000	-1.30172400
H	3.48938100	-5.83203000	-0.73202900
H	2.95492500	-6.03935500	-2.42084000
Au	1.22793900	-2.24881500	-0.82868300
N	0.37402900	-3.01015600	3.43748800
N	-1.45058100	-1.14256600	4.52253200
N	-3.51349100	2.18562700	2.33831800

N	-3.74334200	3.55568000	-0.11771600
N	-1.85378300	2.48094300	-3.85198800
N	-0.05188400	0.44004700	-4.60424100
S	-0.96753500	-0.66069300	0.67633400
O	-2.31161400	-0.75057600	1.24539900
O	-0.75765000	-1.78930700	-0.35084500
O	0.18714500	-0.46468100	1.55918500
C	-0.95807600	0.84559100	-0.41540900
F	-2.06890500	0.92779000	-1.14437100
F	-0.85389800	1.92227800	0.36744100
F	0.10185600	0.81957800	-1.24500100
C	-3.62563500	-3.09826700	-0.49794200
H	-3.16400600	-2.62062900	0.36197800
Cl	-2.59615300	-4.47505400	-1.00301000
Cl	-3.76673000	-1.89631800	-1.79432800
Cl	-5.25126600	-3.68905400	-0.00305600

TfO⊂AuCav

C	2.17026900	-2.63778100	3.67587900
C	1.58533800	-3.28996300	2.42705100
C	2.05763000	-1.11717300	3.59026800
H	3.23668800	-2.86564300	3.66987200
C	0.22469500	-3.58568400	2.29566300
C	2.41062600	-3.58760600	1.33763900
C	0.92624500	-0.43112400	4.03690000
C	3.09783100	-0.34620600	3.05474200
C	-0.32525600	-4.13017400	1.12960600
H	-0.43240200	-3.38177400	3.13381800
C	1.91292500	-4.11103100	0.15304800
O	3.78135400	-3.34904200	1.45597400
C	0.80714900	0.96059600	3.98976900
H	0.10411700	-1.00578100	4.44782500
C	3.02247900	1.03895500	2.97052500
O	4.27295500	-0.99291500	2.66385200
C	-1.79708000	-4.52681500	1.01955100
C	0.55089700	-4.35252700	0.05933200
H	2.56620400	-4.28127300	-0.69099000
C	4.29268200	-2.34193200	0.68430200
C	-0.43226900	1.67866200	4.51757300
C	1.87815900	1.67358400	3.43921700
H	3.83759900	1.61054400	2.54659300
C	4.54840300	-1.08168800	1.32789100
H	-1.84849000	-5.25885600	0.21235400
C	-2.69897600	-3.37274900	0.59146700
O	0.03135700	-4.83686800	-1.15072800
C	-1.51547600	1.74705000	3.44646500
H	-0.12962100	2.70836300	4.71118000
O	1.82099500	3.07391800	3.41984800
C	-3.21225000	-2.44080900	1.50065200
C	-3.05408300	-3.21198600	-0.75587400
C	-0.12824500	-3.86813400	-2.10616400
C	5.04525700	-1.48860400	-1.28116300
C	-2.47175400	0.74422600	3.26678100
C	-1.56952300	2.84415600	2.57894700
C	1.64589700	3.67558300	2.20522700
C	5.31361100	-0.24282600	-0.64116400
C	-4.03159500	-1.37067800	1.11951300
H	-2.93517300	-2.53270100	2.54440600
C	-3.89928900	-2.19335300	-1.17543000
O	-2.49621200	-4.05066200	-1.71915700
C	-1.46426400	-3.45314200	-2.41316500
C	-3.45373700	0.79442000	2.26997800
H	-2.45290700	-0.11128500	3.93107300
C	-2.51319000	2.93597000	1.56513200
O	-0.66885600	3.88205000	2.77506800
C	0.31325400	4.07222200	1.84062900
C	-4.50413300	-0.30304800	2.10235800
C	-4.35967100	-1.28350900	-0.23596200
H	-4.12570600	-2.07108500	-2.22558900
C	0.67764300	-2.32556000	-3.57417400
C	-3.43340500	1.90792100	1.42105700
H	-2.51300800	3.78615800	0.89583500
C	2.41270900	4.51003000	0.23298700
H	-5.37688700	0.17280800	1.64745800
O	-5.06940100	-0.16683900	-0.72760700
C	-0.65472500	-1.90988700	-3.87726900
O	-4.36331400	2.03524300	0.37070600
C	1.08164800	4.85205300	-0.14924300
C	5.83381800	0.82981700	-1.40050700
C	5.30147900	-1.63164600	-2.66400300
C	-0.86519600	-0.83531800	-4.77061100
C	1.76805800	-1.65548200	-4.17147900
C	0.84773200	5.43181000	-1.41665100
C	3.47883400	4.76637800	-0.65874900
C	-4.96438800	-0.90058400	3.44007500
H	-5.33129700	-0.10980200	4.09977300
H	-4.16430600	-1.42593300	3.96760700
C	-2.31043700	-5.21081500	2.29729300
H	-3.34098200	-5.54988400	2.15748300
H	-2.29520700	-4.54959000	3.16827000
C	1.58305700	-3.20697100	4.97545800
H	1.74902800	-4.28707800	5.01996500
H	0.50655300	-3.03726900	5.06941600
C	-0.94081900	1.08920100	5.84132400
H	-0.14705200	1.12035700	6.59241900
H	-1.26300400	0.04796900	5.75096800
H	-1.79288300	1.66756300	6.21037100
H	2.06465000	-2.74400500	5.84131900
H	-1.68658300	-6.07706400	2.53281100
H	-5.77147100	-1.61829900	3.27031500
C	3.22546800	5.33100000	-1.89039100
C	1.90412300	5.66286200	-2.27228700
H	1.72478800	6.10525700	-3.24730900
H	4.04379900	5.52058400	-2.57803200
H	-0.17328300	5.68473900	-1.68244600
H	4.47960600	4.48953100	-0.34596800
C	5.79859100	-0.56605100	-3.38537000
H	5.09778800	-2.59416100	-3.12154000
C	6.06689000	0.66925100	-2.74993100
H	6.02180900	1.76853200	-0.89113800
C	1.53763700	-0.60525300	-5.03328900
H	2.77013400	-1.96544600	-3.89996400
C	0.21770200	-0.19436100	-5.33574600
H	-1.88650500	-0.53704400	-4.98361600
H	0.05986800	0.63632300	-6.01680400
H	2.37678200	-0.07584300	-5.47324900
H	6.45922300	1.49621600	-3.33380200
H	5.99213600	-0.67445700	-4.44824600
P	-4.12954700	1.14960600	-0.99496900
N	-4.96926500	1.92954600	-2.18661500
C	-6.42685100	2.06986600	-2.09196900
C	-4.28613800	3.02376300	-2.88231700
H	-6.85344600	1.18120400	-1.62712100
H	-6.83405800	2.16337400	-3.10294400
H	-6.71021300	2.95624700	-1.51021200
H	-3.21508700	2.81472100	-2.94429100
H	-4.43010900	3.98625400	-2.37373500
H	-4.68269900	3.09735400	-3.89912100
Au	-1.97331700	0.58170700	-1.27933000
N	0.03266000	4.63050900	0.70280600
N	2.66584700	3.90545200	1.43489700
N	5.05322100	-0.06825700	0.69233800
N	4.53748100	-2.54433600	-0.57398000
N	0.90770300	-3.33051000	-2.67929800
N	-1.72253900	-2.51291500	-3.27316100
S	0.99595600	0.26481400	-0.34341700
O	1.79298500	-0.86200500	0.13540400
O	0.41806300	1.20968000	0.62238000
O	-0.02500100	-0.17778200	-1.41132900
C	2.14216300	1.27920100	-1.39074000
F	3.08980100	1.80210800	-0.60774800
F	2.72033500	0.52873800	-2.33087000
F	1.46736500	2.27480800	-1.98547500

TfO⊂AuCav-1

C	3.02804200	-2.10342900	3.55894600
C	2.43853500	-2.89516300	2.39177000
C	2.67537300	-0.61816400	3.45601000
H	4.11153800	-2.16541900	3.45059800
C	1.12861900	-3.38986100	2.41025200
C	3.18910900	-3.12907600	1.23163200

C	1.50072300	-0.08014800	3.99102500
C	3.53275600	0.26946200	2.79104900
C	0.55161700	-4.06554300	1.32948500
H	0.53193500	-3.23449700	3.30227400
C	2.65477200	-3.77993600	0.12568900
O	4.51252500	-2.68308700	1.17468300
C	1.17230400	1.27720300	3.89474600
H	0.81440300	-0.74371200	4.50524000
C	3.24683000	1.62211400	2.65806100
O	4.72345400	-0.23107700	2.26522000
C	-0.86135900	-4.64591300	1.37598800
C	1.34020200	-4.21939200	0.18138800
H	3.24062000	-3.89993800	-0.77495100
C	4.73785400	-1.62767100	0.32449400
C	-0.10231500	1.86130400	4.49926600
C	2.06906200	2.10771200	3.21180800
H	3.91850000	2.27936500	2.12293200
C	4.80732900	-0.31700200	0.90054300
H	-0.89054700	-5.42164400	0.60976000
C	-1.93114100	-3.63085800	0.98139800
O	0.77665300	-4.83468300	-0.94225900
C	-1.26608900	1.74064200	3.52174300
H	0.08133500	2.92906000	4.62361700
O	1.79182000	3.47438300	3.14339300
C	-2.47662500	-2.72539400	1.89781800
C	-2.41828600	-3.58088400	-0.33261900
C	0.39104300	-3.97515400	-1.94063600
C	5.02914100	-0.72776400	-1.74312800
C	-2.07780700	0.60465700	3.47318300
C	-1.55399500	2.77415200	2.61918500
C	1.44632600	3.98283300	1.91872900
C	5.04478500	0.58139900	-1.17392900
C	-3.46411100	-1.79371700	1.55800800
H	-2.10761000	-2.73735200	2.91668200
C	-3.40397000	-2.67741600	-0.71590800
O	-1.88344500	-4.43493900	-1.29527400
C	-1.01310000	-3.79731100	-2.15639000
C	-3.14659800	0.46541100	2.58369600
H	-1.87074200	-0.20507800	4.16200600
C	-2.59452800	2.67354000	1.70030900
O	-0.82355300	3.95261700	2.69454900
C	0.05555500	4.23465200	1.67538900
C	-4.02062900	-0.78396600	2.55590400
C	-3.90330700	-1.79680000	0.23156800
H	-3.74146100	-2.64552800	-1.74298000
C	0.82219800	-2.45179300	-3.57943700
C	-3.37292200	1.52370600	1.69614600
H	-2.78881900	3.48636500	1.01223300
C	1.94340300	4.78126200	-0.14827500
H	-4.99733700	-0.47385600	2.17554600
O	-4.86955800	-0.86321300	-0.19779800
C	-0.57821600	-2.31517700	-3.82911700
O	-4.45064300	1.47709100	0.79192400
C	0.55819000	5.03333700	-0.38905700
C	5.15084300	1.70799000	-2.01956200
C	5.15853600	-0.87918700	-3.14227100
C	-1.02681900	-1.41374800	-4.82035400
C	1.73737200	-1.65282900	-4.29941300
C	0.15388100	5.55585900	-1.63738600
C	2.88787800	5.05732900	-1.16176200
C	-4.25421700	-1.39048400	3.94666400
H	-4.68884400	-0.64242000	4.61501500
H	-3.33522700	-1.75461400	4.41259100
C	-1.17103500	-5.31492500	2.72479000
H	-2.15963900	-5.78199000	2.69684300
H	-1.16212100	-4.61055100	3.56142000
C	2.66264600	-2.70404800	4.92420900
H	2.99127100	-3.74575100	4.97635900
H	1.58717600	-2.68934300	5.12350800
C	-0.42269900	1.28062700	5.88414700
H	0.41552600	1.45622700	6.56366300
H	-0.60631600	0.20257300	5.86547200
H	-1.31389700	1.76031700	6.29893000
H	3.15211000	-2.14302400	5.72513800
H	-0.42642300	-6.08633800	2.93833900
H	-4.94202700	-2.23714200	3.87365000
C	2.46673200	5.55698400	-2.37524200
C	1.09434800	5.80776600	-2.61371100
H	0.78332300	6.20113800	-3.57656800
H	3.19038500	5.75998900	-3.15856300
H	-0.90350000	5.73795000	-1.79753300
H	3.93240300	4.85474500	-0.95177600
C	5.26503700	0.23629900	-3.94551700
H	5.16556200	-1.88564400	-3.54730600
C	5.25286500	1.53465100	-3.38230500
H	5.13185100	2.69069200	-1.56221600
C	1.27367000	-0.76804000	-5.24729800
H	2.78880200	-1.73249400	-4.05597800
C	-0.11049000	-0.65560200	-5.51705400
H	-2.09433900	-1.33288500	-4.99654700
H	-0.45212100	0.04785800	-6.27007000
H	1.97650900	-0.13716900	-5.78181500
H	5.32338800	2.39970800	-4.03426700
H	5.35713700	0.12142200	-5.02131500
P	-4.34376300	0.62762000	-0.60922700
N	-5.60265500	1.16810100	-1.53428400
C	-6.98542500	0.96470900	-1.08572700
C	-5.38856200	2.38290800	-2.32630900
H	-7.07005800	0.00871700	-0.56977000
H	-7.63608000	0.94425000	-1.96471200
H	-7.31491300	1.77009500	-0.41701600
H	-4.34793000	2.43726200	-2.65545600
H	-5.62714600	3.29040100	-1.75610000
H	-6.02796200	2.34265900	-3.21279800
Au	-2.28060200	0.64706600	-1.48480100
N	-0.37926700	4.75304100	0.56696800
N	2.36397000	4.25773700	1.04161700
N	4.94937100	0.75420400	0.17871600
N	4.86483400	-1.83192400	-0.95278500
N	1.27883300	-3.32115100	-2.62870700
N	-1.48439100	-3.01159200	-3.07853300
S	0.85134600	0.89068000	-1.38348000
O	2.09658000	0.53732100	-2.05577500
O	-0.35754800	0.55348000	-2.28250100
O	0.72024900	2.18512800	-0.71402200
C	0.65448000	-0.35171200	-0.00178900
F	0.13727500	-1.50272100	-0.45827000
F	1.85075000	-0.59280700	0.53373600
F	-0.16360700	0.11738900	0.95077700

$CHCl_3 \subset AuCav$

C	3.18629300	4.02322700	-0.66768200
C	2.70435500	3.50716500	0.68833100
C	2.74430500	3.03923800	-1.75219900
H	4.27602200	3.99199700	-0.64483000
C	1.44682500	3.82654200	1.21452000
C	3.50484500	2.63340100	1.43676100
C	1.49043300	3.12489300	-2.36828100
C	3.57451900	1.97936700	-2.14463800
C	0.95759200	3.27677500	2.40557400
H	0.82382100	4.52925600	0.67313300
C	3.05390200	2.03778800	2.60890500
O	4.80455800	2.38480000	0.99575400
C	1.03866300	2.19654000	-3.31216900
H	0.84438800	3.95540500	-2.10840300
C	3.15372300	1.00402500	-3.04366800
O	4.87294900	1.91939400	-1.63821300
C	-0.38800200	3.67562700	3.01168300
C	1.77808000	2.34709600	3.05738500
H	3.67741300	1.33731900	3.14677000
C	5.10178000	1.11183100	0.60161400
C	-0.28035300	2.35583100	-4.06454600
C	1.88457200	1.11651400	-3.59441200
H	3.80323400	0.17906300	-3.30249300
C	5.15266700	0.86511000	-0.81304100
H	-0.32055400	3.43276000	4.07290000
C	-1.55768300	2.85352500	2.47283600
O	1.29011500	1.71927200	4.21011800
C	-1.46189900	1.76464600	-3.30470000
H	-0.18123200	1.76189200	-4.97378100

O	1.43371300	0.14119500	-4.49647700
C	-2.21879900	3.18346900	1.28232800
C	-2.03584300	1.73864800	3.17769300
C	0.84385400	0.44226600	4.02559600
C	5.56933100	-1.08040200	0.99433600
C	-2.15714800	2.49265400	-2.33255700
C	-1.91667200	0.46723200	-3.57812500
C	0.98428300	-1.01306000	-3.92966800
C	5.63100900	-1.32233900	-0.41318400
C	-3.31949400	2.47024400	0.79057400
H	-1.85696200	4.03083900	0.71241500
C	-3.14610000	1.01976300	2.74953500
O	-1.37466700	1.31599100	4.32802800
C	-0.57090700	0.21888100	4.12241200
C	-3.27317700	1.99588200	-1.65220600
H	-1.81256900	3.49226900	-2.09794300
C	-3.03535900	-0.06487200	-2.94473800
O	-1.24132500	-0.31314500	-4.51808700
C	-0.42918800	-1.27735100	-3.97707600
C	-3.97501700	2.78879300	-0.55116300
C	-3.76623000	1.39867800	1.56845700
H	-3.49966400	0.17204700	3.32106800
C	1.14587600	-1.76472500	3.54002100
C	-3.69699300	0.70777400	-1.99852400
H	-3.37746900	-1.06144100	-3.19101500
C	1.29543500	-2.95748500	-2.76858700
H	-5.00141400	2.41713500	-0.49877700
O	-4.87527200	0.62748800	1.15471500
C	-0.25724900	-1.99315000	3.67671100
O	-4.81857500	0.12888300	-1.37312600
C	-0.09926200	-3.25085600	-2.87725900
C	5.86599500	-2.63465300	-0.88518700
C	5.74269100	-2.15694900	1.89551600
C	-0.77886600	-3.28959000	3.45740100
C	1.99228000	-2.83403800	3.16227300
C	-0.60870300	-4.43606400	-2.29834500
C	2.13928200	-3.82690400	-2.03847500
C	-4.05490400	4.29305600	-0.84422600
H	-4.57046700	4.46258500	-1.79265400
H	-3.07326200	4.76824500	-0.91309800
C	-0.65124000	5.18628000	2.89896500
H	-1.58251900	5.44620600	3.40910500
H	-0.73344200	5.52960500	1.86391400
C	2.76619000	5.47036300	-0.95476200
H	3.14641200	6.13239900	-0.17258200
H	1.68138400	5.60388200	-0.99920800
C	-0.53423400	3.81092300	-4.49116500
H	0.29966800	4.16970900	-5.09932700
H	-0.63972800	4.49353000	-3.64351500
H	-1.44989900	3.87498300	-5.08492800
H	3.17629500	5.79653300	-1.91397500
H	0.16652300	5.74133800	3.36494900
H	-4.61037100	4.79900300	-0.05077500
C	1.61209300	-4.96520700	-1.46571000
C	0.23795600	-5.27733700	-1.60642200
H	-0.14989500	-6.18829800	-1.16203400
H	2.25634400	-5.63455100	-0.90498300
H	-1.66584300	-4.65150100	-2.41225000
H	3.18729200	-3.56522900	-1.94092800
C	5.96067500	-3.42948400	1.40880100
H	5.70238500	-1.94455300	2.95862000
C	6.02294300	-3.66886900	0.01464000
H	5.91844500	-2.78993800	-1.95758700
C	1.45781600	-4.08606800	2.94192900
H	3.05148200	-2.63373600	3.04491500
C	0.06961600	-4.31711100	3.09559000
H	-1.84536400	-3.44367600	3.58536400
H	-0.32760900	-5.31381400	2.93183200
H	2.10643400	-4.90572000	2.65025200
H	6.20631600	-4.67600800	-0.34597000
H	6.09700600	-4.25604800	2.09883000
P	-4.61546300	-0.57230400	0.09453400
N	-5.89327500	-1.56740800	0.35112500
C	-7.23367600	-1.02921900	0.64212300
C	-5.87923800	-2.88467600	-0.29850000
H	-7.14738300	-0.09192600	1.19002100
H	-7.76386100	-1.75193500	1.26738400
H	-7.80238700	-0.86487500	-0.28010900
H	-4.85077700	-3.23854500	-0.41065100
H	-6.35353700	-2.85318500	-1.28643500
H	-6.41811600	-3.59317300	0.33562900
Au	-2.49708700	-1.40025000	0.23334200
N	-0.94956000	-2.35947600	-3.47431400
N	1.81049600	-1.81613000	-3.32070400
N	5.41750300	-0.30684100	-1.30708200
N	5.30648400	0.17453800	1.47814600
N	1.67137800	-0.51681200	3.73031300
N	-1.10428300	-0.95494600	3.95566600
C	0.80636400	-0.70477700	-0.27542300
H	0.74002100	-0.74732300	-1.35639000
Cl	2.45751000	-0.93424700	0.23004800
Cl	-0.16560300	-2.18491500	0.33003100
Cl	0.07821200	0.78840400	0.30442400

$(CHCl_3)_2 \subset$AuCav

C	-0.17565900	5.37593700	-0.85844200
C	0.17993700	4.30647400	-1.88823500
C	-0.50736300	4.72454200	0.48268800
H	-1.09534100	5.84616100	-1.20783500
C	1.48804200	3.85553500	-2.09677100
C	-0.82897600	3.72003700	-2.66385200
C	0.49224600	4.35401300	1.38664300
C	-1.83130700	4.45203700	0.85476400
C	1.80985100	2.85955600	-3.02818800
H	2.28904400	4.31038600	-1.52566700
C	-0.56533400	2.72028400	-3.59190000
O	-2.12457700	4.21086100	-2.52244600
C	0.22859200	3.73860800	2.61474700
H	1.52495600	4.54743500	1.11909800
C	-2.14507200	3.84090600	2.06343500
O	-2.87135800	4.80814500	-0.00879900
C	3.24789400	2.41670900	-3.29612300
C	0.74900700	2.30020500	-3.75293700
H	-1.36412400	2.27847700	-4.17124100
C	-3.06395000	3.40458000	-1.94880600
C	1.35094200	3.37677400	3.58342800
C	-1.11303100	3.48184800	2.92196200
H	-3.17481800	3.63023300	2.32060100
C	-3.49231600	3.75089300	-0.62110600
H	3.25021300	1.99993100	-4.30402300
C	3.68986900	1.28233400	-2.37337600
O	1.03686000	1.30883900	-4.69807000
C	2.01432200	2.06140400	3.18828600
H	0.87634700	3.19868100	4.54927500
O	-1.43869400	2.85540600	4.13557100
C	4.19393600	1.51428700	-1.08862300
C	3.61464700	-0.05322000	-2.79591400
C	0.70474900	0.03628900	-4.33300200
C	-4.49542000	1.63622800	-1.94946600
C	3.16519200	1.99465300	2.39929700
C	1.48374500	0.84778900	3.64819400
C	-1.84644900	1.55703600	4.00021900
C	-4.93739500	1.99103700	-0.63729300
C	4.61868000	0.48833500	-0.23496900
H	4.23946500	2.53584200	-0.72993000
C	4.06056800	-1.10336900	-2.00178300
O	3.05534300	-0.34692700	-4.03455500
C	1.77672200	-0.84605100	-3.96916700
C	3.81096400	0.79280100	2.08791600
H	3.59279900	2.91921500	2.03285400
C	2.08633700	-0.37601900	3.36837400
O	0.39491700	0.91108100	4.50326100
C	-0.84909200	0.52431000	4.09914300
C	5.05430200	0.74744000	1.20387700
C	4.54896300	-0.81215400	-0.73670100
H	3.99933600	-2.12225300	-2.36002300
C	-0.79619000	-1.61474400	-3.87469000
C	3.24820200	-0.38150900	2.60205800
H	1.68459300	-1.29147100	3.78357800
C	-3.42867500	-0.03255600	3.59792100

H	5.63934200	-0.11880800	1.52262100
O	4.91747900	-1.91034600	0.07560800
C	0.27009200	-2.48635800	-3.49280500
O	3.89441400	-1.62092900	2.42926300
C	-2.42692100	-1.05170000	3.63167400
C	-5.89439300	1.18320700	0.01929400
C	-5.02969700	0.48794700	-2.57639600
C	-0.02692500	-3.78288400	-3.01158900
C	-2.13157600	-2.06699800	-3.78124900
C	-2.80247800	-2.40378400	3.47043200
C	-4.77904300	-0.38907900	3.38265100
C	5.95921800	1.97850900	1.34479800
H	6.25342600	2.11330900	2.38867000
H	5.47959100	2.90251900	1.01270000
C	4.24006600	3.59050800	-3.27856900
H	5.23941200	3.24237600	-3.55264900
H	4.31538400	4.07318300	-2.30013400
C	0.89006000	6.47400400	-0.73363100
H	1.06553700	6.93993500	-1.70683500
H	1.85092300	6.10081500	-0.36755500
C	2.35497300	4.52476900	3.76541500
H	1.83568000	5.41168100	4.13605500
H	2.85659900	4.80717500	2.83561600
H	3.12487900	4.24597800	4.49011700
H	0.55177500	7.24455900	-0.03630200
H	3.92864800	4.35193600	-3.99782600
H	6.86183300	1.84491000	0.74368800
C	-5.12367400	-1.71691200	3.24361800
C	-4.13340600	-2.72532400	3.30160300
H	-4.42996600	-3.76822100	3.23909800
H	-6.16286100	-1.99490000	3.10188200
H	-2.03231000	-3.16487500	3.52253000
H	-5.51744400	0.40423600	3.35635200
C	-5.96199900	-0.28425500	-1.91461900
H	-4.69053100	0.24981200	-3.57855000
C	-6.39265700	0.06228600	-0.61091200
H	-6.20983900	1.47358700	1.01517100
C	-2.39520500	-3.33893100	-3.31960300
H	-2.92247200	-1.38479100	-4.07055400
C	-1.34136700	-4.19805200	-2.92831200
H	0.79796100	-4.43089600	-2.73285800
H	-1.57467700	-5.19146800	-2.56152500
H	-3.41855500	-3.68720600	-3.24444100
H	-7.12412100	-0.56186300	-0.10831000
H	-6.37329700	-1.16627000	-2.39432300
P	3.76240700	-2.49581400	1.05328000
N	4.29759500	-4.00893600	1.38698200
C	5.72890900	-4.27331300	1.61674600
C	3.37824700	-4.93866700	2.05566600
H	6.33384300	-3.58959900	1.02263700
H	5.94328200	-5.29611000	1.29699400
H	5.98564500	-4.16539300	2.67662900
H	2.34560200	-4.71571900	1.77414200
H	3.46919600	-4.88226700	3.14682200
H	3.61017300	-5.95542100	1.72873700
Au	1.67794200	-2.21554000	0.19173300
N	-1.11497400	-0.73070000	3.87483700
N	-3.09333000	1.28414500	3.75710100
N	-4.40292900	3.07320500	0.01149400
N	-3.54354100	2.38113500	-2.59085900
N	-0.53989300	-0.33669100	-4.28582600
N	1.56898200	-2.05816400	-3.54647800
C	-3.80800700	-4.29294800	0.20108900
H	-4.38565600	-4.46388600	1.10277500
Cl	-4.37471700	-5.44660200	-1.03470500
Cl	-2.09042000	-4.59877800	0.62406400
Cl	-4.08384700	-2.60661300	-0.29840500
C	-0.63316500	0.06283300	0.43834600
H	-0.34158800	-0.17200000	1.45902000
Cl	-0.61367700	-1.58460300	-0.44406300
Cl	0.54384100	1.14599700	-0.29436700
Cl	-2.27147700	0.67120000	0.40193900

($CHCl_3$)⊂AuClCav

C	-1.97847000	3.50652700	3.29589000
C	-2.87037000	2.66252400	2.38737600
C	-0.48676600	3.25825700	3.08408000
H	-2.19185200	3.17162800	4.31205500
C	-3.24551300	3.06180100	1.10076100
C	-3.38517800	1.44254900	2.84554100
C	0.25397000	3.94102400	2.11320500
C	0.21365600	2.35878800	3.89964300
C	-4.11121900	2.31502700	0.29084600
H	-2.83865200	3.98677200	0.70758700
C	-4.27861500	0.68814300	2.10055900
O	-2.98223000	0.97052600	4.09452900
C	1.63312000	3.77428700	1.94404500
H	-0.26603600	4.63598400	1.46352100
C	1.58328200	2.16837500	3.77967700
O	-0.47952000	1.62847400	4.87248600
C	-4.43187800	2.72364300	-1.14575400
C	-4.63307900	1.13559700	0.83507200
H	-4.66112000	-0.24800600	2.48216000
C	-2.02436000	-0.01251700	4.04189700
C	2.41718600	4.52295000	0.86779400
C	2.27514400	2.87814600	2.80793300
H	2.09581800	1.45572900	4.41065500
C	-0.71217700	0.32352800	4.50792100
H	-5.34331200	2.19333200	-1.42463900
C	-3.32845100	2.20826200	-2.06668500
O	-5.53775300	0.36991100	0.08422500
C	2.36487300	3.76978100	-0.45904100
H	3.46053100	4.51045600	1.18562800
O	3.66053900	2.69316000	2.69835900
C	-2.18656800	2.95609000	-2.36961000
C	-3.40837800	0.91905300	-2.61057400
C	-5.05609900	-0.84786300	-0.31163900
C	-1.30790700	-2.11044700	3.53436600
C	1.32017900	3.92589900	-1.37624100
C	3.36806900	2.84643900	-0.78460400
C	4.02571100	1.48035200	2.17533800
C	-0.01949600	-1.79676600	4.06416300
C	-1.13718400	2.46818600	-3.15760600
H	-2.11210300	3.96386100	-1.97968900
C	-2.38036000	0.37592800	-3.36927000
O	-4.57171300	0.18384700	-2.40750500
C	-4.49610400	-0.93932300	-1.63091100
C	1.22969200	3.18596500	-2.56166400
H	0.52906300	4.62956600	-1.14436300
C	3.33379200	2.10567200	-1.95750400
O	4.40873900	2.63548700	0.11364100
C	4.37094800	1.43655100	0.78488000
C	0.06665100	3.32969700	-3.54085700
C	-1.25685100	1.15014000	-3.61718300
H	-2.45932100	-0.63336900	-3.75125500
C	-4.55920900	-3.03962400	0.03910300
C	2.25670300	2.27126200	-2.81299000
H	4.09568000	1.36422400	-2.15463300
C	4.32440000	-0.76935400	2.30976300
H	0.42355800	2.94047100	-4.49804900
O	-0.24442600	0.56208700	-4.38978700
C	-3.97624500	-3.12142500	-1.26205500
O	2.16492200	1.38880700	-3.90495800
C	4.59767300	-0.82146500	0.90943600
C	0.99847400	-2.77512900	4.03847800
C	-1.53473300	-3.38026600	2.95750000
C	-3.40722700	-4.34122600	-1.69371500
C	-4.56175200	-4.18045700	0.87437100
C	4.82172300	-2.07078300	0.28986100
C	4.32382200	-1.96541700	3.06209300
C	-0.31187000	4.80087800	-3.77241400
H	0.55670400	5.35440500	-4.13873100
H	-0.65708900	5.29839600	-2.86246900
C	-4.69966000	4.22673900	-1.29826500
H	-4.95139600	4.46377900	-2.33593600
H	-3.84286100	4.84511300	-1.01626200
C	-2.33194100	5.00085700	3.21956500
H	-3.38911500	5.14976200	3.45649000
H	-2.15335700	5.43010700	2.22947900
C	1.98907000	5.99148800	0.73167900

H	2.09779900	6.50288400	1.69182300
H	0.94799500	6.10544300	0.41641800
H	2.61470500	6.50046500	-0.00713200
H	-1.72992100	5.56619800	3.93626400
H	-5.53755100	4.52165400	-0.66103800
H	-1.11193300	4.87322200	-4.51396300
C	4.54263300	-3.17296700	2.43422600
C	4.78490400	-3.22653500	1.04096400
H	4.93954400	-4.18738000	0.56228600
H	4.52599300	-4.09416300	3.00795400
H	5.00920400	-2.08845100	-0.77754400
H	4.13820100	-1.89317500	4.12774700
C	-0.51703100	-4.30913700	2.92566200
H	-2.50983200	-3.58419900	2.53115800
C	0.75181900	-4.00861700	3.47651600
H	1.96635100	-2.51166200	4.44461200
C	-3.99316900	-5.35705300	0.43260200
H	-5.01378500	-4.09088400	1.85632000
C	-3.41413600	-5.43755600	-0.85573600
H	-2.97172600	-4.38096700	-2.68615600
H	-2.96815100	-6.37062600	-1.18418300
H	-3.98854800	-6.23114400	1.07597500
H	1.54013000	-4.75237300	3.43651000
H	-0.68253300	-5.27641100	2.46275300
P	1.14893700	0.12350300	-3.65141500
N	1.73606200	-0.96080900	-4.78086300
C	0.88479500	-2.12379200	-5.06783600
C	3.16910100	-1.27176000	-4.72245900
H	-0.15294400	-1.80555300	-5.16940600
H	0.95535300	-2.89433900	-4.28896400
H	1.20408300	-2.55450500	-6.02118000
H	3.74166200	-0.35550600	-4.58179400
H	3.46135500	-1.71759500	-5.67750200
H	3.41333600	-1.97811900	-3.91736000
Au	0.95929700	-0.31191100	-1.42651500
Cl	0.92108700	-0.68692300	0.88485600
N	4.63884800	0.32616300	0.16587000
N	4.03150400	0.41692200	2.92214100
N	0.25223600	-0.54667000	4.54669500
N	-2.31388500	-1.18516500	3.56240400
N	-5.09778500	-1.86680600	0.49403100
N	-3.96674200	-2.03270000	-2.09351900
C	1.42974600	-3.92270500	-0.31977700
H	1.58054800	-3.08013600	0.35497400
Cl	2.39888300	-3.62075900	-1.80383400
Cl	1.99259300	-5.42305800	0.47982300
Cl	-0.30244300	-4.01694700	-0.70966400

(TfO•1a)⊂AuCav

C	5.85026500	-1.18286200	0.81281000
C	5.29858800	0.04335900	0.08935400
C	4.72786400	-2.11418300	1.26354100
H	6.32107600	-0.81159700	1.72345000
C	5.09507500	0.08143900	-1.29265900
C	4.99636900	1.20643300	0.80989000
C	4.18333200	-3.07760000	0.40630200
C	4.20968800	-2.04985200	2.56453800
C	4.63867200	1.22237100	-1.96114900
H	5.32159100	-0.80383200	-1.87490100
C	4.52581200	2.36390200	0.19702100
O	5.27274800	1.20183500	2.17696400
C	3.17452200	-3.96260900	0.79483500
H	4.56969200	-3.14470800	-0.60425400
C	3.19324800	-2.89936500	2.99502100
O	4.76534800	-1.13382100	3.47218800
C	4.42848900	1.25696100	-3.47131200
C	4.35964700	2.35548800	-1.18504100
H	4.31703600	3.25336500	0.77645200
C	4.22736500	1.17869600	3.05243800
C	2.59953600	-5.02098900	-0.14603000
C	2.68436300	-3.83805800	2.10225200
H	2.79320500	-2.82019000	3.99713400
C	3.99794200	-0.04207200	3.78075800
H	4.49487200	2.30295300	-3.77099100
C	3.01056300	0.79918400	-3.79194100
O	4.00770200	3.53031300	-1.85306700
C	1.47490800	-4.42174000	-0.98376900
H	2.14185500	-5.78012300	0.48817000
O	1.68959100	-4.72509000	2.50856200
C	2.68147600	-0.55587900	-3.89795800
C	1.97226600	1.72596900	-3.95257200
C	2.74349800	4.02947800	-1.73086800
C	2.49820200	2.16516600	4.15387800
C	1.73021500	-3.72926500	-2.16968100
C	0.13680900	-4.52111900	-0.57442700
C	0.41833600	-4.23022700	2.65833700
C	2.30960300	0.97617200	4.92365200
C	1.38277600	-1.00034200	-4.14998300
H	3.46818200	-1.29136100	-3.77655900
C	0.66070500	1.32836800	-4.19509000
O	2.27950800	3.08790700	-3.89656900
C	1.82582000	3.79752000	-2.81830000
C	0.73097700	-3.10910600	-2.92473000
H	2.75108400	-3.68045200	-2.52879500
C	-0.89068600	-3.89012800	-1.27700000
O	-0.15414000	-5.28662000	0.55774700
C	-0.56239500	-4.58685300	1.67124600
C	1.02774300	-2.47348000	-4.27851300
C	0.38009100	-0.03219800	-4.28124600
H	-0.12539000	2.06581600	-4.29642000
C	1.16186300	5.27589100	-0.67061600
C	-0.57327000	-3.14708800	-2.41212400
H	-1.91751700	-3.95539900	-0.93628300
C	-1.15706800	-3.03606400	3.78583700
H	0.09400000	-2.51415100	-4.84443200
O	-0.93453000	-0.43237200	-4.48137100
C	0.24582000	5.03036600	-1.73956400
O	-1.62119400	-2.51174000	-3.08444100
C	-2.15091200	-3.46094700	2.85106000
C	1.24936200	0.91578600	5.85739400
C	1.60533000	3.25049000	4.31289700
C	-1.05701900	5.57656500	-1.68031800
C	0.75913800	6.07942400	0.42115900
C	-3.47592400	-2.98857100	2.97113500
C	-1.50964900	-2.12858900	4.80950800
C	2.07248400	-3.26088000	-5.08456500
H	1.75671100	-4.30160200	-5.19498100
H	3.06059500	-3.26366800	-4.61692600
C	5.49938000	0.48133400	-4.25097200
H	5.32958700	0.58884500	-5.32569200
H	5.50496100	-0.58868000	-4.02569400
C	6.93031100	-1.91411500	0.00129800
H	7.74131800	-1.22554200	-0.25149900
H	6.55230600	-2.33596500	-0.93415100
C	3.67874600	-5.71012700	-0.99341200
H	4.42804400	-6.16983600	-0.34327700
H	4.20371300	-5.02261300	-1.66281000
H	3.22789500	-6.49133400	-1.61153900
H	7.34334500	-2.73862500	0.58846900
H	6.49268800	0.87260300	-4.01479800
H	2.18180100	-2.82089000	-6.07943000
C	-2.80667300	-1.66822600	4.89618800
C	-3.79251800	-2.10111700	3.97728500
H	-4.80194300	-1.71096100	4.04565200
H	-3.07699500	-0.95879300	5.67243100
H	-4.20052200	-3.30356400	2.23209500
H	-0.73299200	-1.80107300	5.49171000
C	0.55611600	3.15260300	5.20402300
H	1.75859900	4.13201100	3.69933200
C	0.38582700	1.98406400	5.98616400
H	1.12438300	0.00187400	6.42774700
C	-0.51344500	6.61255600	0.44952000
H	1.47452000	6.25447300	1.21749100
C	-1.42562400	6.35626600	-0.60239700
H	-1.74181100	5.35794700	-2.49168200
H	-2.42646600	6.77211000	-0.55712900
H	-0.82268500	7.22574800	1.28995100
H	-0.44483200	1.92673400	6.68215300
H	-0.15753300	3.96533900	5.28994400
P	-1.84129500	-0.88638000	-3.14189400

N	-3.39075500	-0.79041500	-3.65172000
C	-4.01396800	0.53889100	-3.68166200
C	-4.02696600	-1.80152000	-4.50259000
H	-3.59851600	1.17480100	-2.89683000
H	-5.07756300	0.42226600	-3.47071400
H	-3.87294700	1.01682100	-4.66021800
H	-3.52368300	-2.75907500	-4.38161400
H	-3.99043100	-1.49979300	-5.55730900
H	-5.06020500	-1.91189600	-4.16945400
Au	-1.12248100	0.41904400	-1.39383400
N	-1.81087500	-4.24958200	1.78690700
N	0.13224100	-3.47771000	3.67837200
N	3.08521800	-0.12992500	4.70343600
N	3.49830600	2.24137400	3.22498600
N	2.42235900	4.74260600	-0.69255400
N	0.61724800	4.27398200	-2.81810100
C	-0.11363100	2.17929300	0.15427000
C	0.68881400	1.55844600	-0.53435400
H	1.57089800	1.13233200	-0.96349000
C	-0.87448400	3.12889400	0.96864900
C	-2.24321400	2.71544400	1.58146000
H	-1.05594000	3.99987500	0.34100500
H	-0.22069000	3.43934800	1.79055600
C	-3.17386800	2.21561000	0.46381700
O	-2.87418700	2.33044700	-0.72216500
O	-4.26522400	1.64691000	0.91242100
H	-4.73960600	1.13542100	0.15717600
C	-2.10741700	1.67588600	2.73262200
H	-1.61327800	2.17549500	3.56769000
H	-3.11323300	1.38887600	3.04689000
C	-2.77507700	4.02339900	2.18819500
O	-2.36027700	4.50256500	3.22231000
O	-3.70031800	4.60527600	1.41167700
C	-4.21788300	5.85818000	1.89218300
H	-4.68355200	5.72570200	2.87083300
H	-3.41317500	6.59223100	1.97701900
H	-4.95407800	6.17161900	1.15369100
C	-1.31336200	0.48820200	2.27399300
C	0.05033500	0.40554900	2.55982900
C	-1.88462100	-0.46071000	1.42334000
C	0.85608200	-0.55103500	1.94772900
H	0.48106400	1.13679700	3.22464900
C	-1.07800700	-1.41012200	0.79748300
H	-2.94638900	-0.47004800	1.22330800
C	0.30027200	-1.44096100	1.03256900
H	1.91615900	-0.58571100	2.15668900
H	-1.55491100	-2.12715000	0.15120200
H	0.92661000	-2.15956700	0.51815400
S	-5.20950700	-1.26145600	-0.87386500
O	-5.36594400	0.23491100	-0.88654800
O	-3.84169300	-1.72190300	-0.54776900
O	-5.89753400	-1.93182000	-1.98349200
C	-6.18565700	-1.71414100	0.63823200
F	-5.70332400	-1.04396900	1.70752100
F	-7.47535900	-1.39975200	0.49452100
F	-6.09423400	-3.03010200	0.89572600

1a-TS1$_{5anti}$⊂AuCav

C	-2.93566300	3.89300900	3.24094100
C	-3.01612700	2.38296500	3.44045100
C	-2.24028200	4.24733300	1.93140800
H	-2.29163000	4.26790500	4.03694200
C	-4.10571200	1.63126300	2.99760200
C	-1.99969200	1.69846200	4.12398900
C	-2.91284800	4.26689800	0.70577900
C	-0.87490500	4.56315900	1.91925700
C	-4.23731200	0.26260700	3.24814100
H	-4.90071100	2.13867400	2.46396100
C	-2.07518400	0.33228900	4.38039000
O	-0.95227900	2.44188500	4.65364100
C	-2.27754600	4.58781300	-0.49840500
H	-3.96536700	4.00458200	0.68630600
C	-0.20851900	4.91423200	0.75098400
O	-0.17261000	4.53844900	3.12135500
C	-5.45268200	-0.52731300	2.77500600
C	-3.20151400	-0.36310100	3.95225800
H	-1.28741300	-0.16756000	4.92917100
C	0.28533600	2.37053500	4.06402700
C	-2.99092400	4.55493800	-1.84283700
C	-0.91921000	4.91316600	-0.44406700
H	0.84814400	5.14565800	0.76870100
C	0.70540400	3.48914000	3.27290000
H	-5.51379500	-1.41302300	3.40772600
C	-5.21427100	-1.02539300	1.35461600
O	-3.36471400	-1.70604200	4.31843200
C	-3.08309800	3.12661400	-2.36623700
H	-2.35217400	5.09355800	-2.54338900
O	-0.26732300	5.22361300	-1.64073300
C	-5.49953700	-0.24881600	0.22793800
C	-4.65453200	-2.29129600	1.13745500
C	-2.57218100	-2.64188400	3.72282100
C	2.29486800	1.36504700	3.70636700
C	-4.19439100	2.32438400	-2.09453200
C	-2.06621000	2.57814800	-3.16811300
C	0.65671300	4.27468200	-2.00720000
C	2.71185900	2.47811800	2.91019700
C	-5.26872300	-0.68884500	-1.07968800
H	-5.91911800	0.73889700	0.37518300
C	-4.39830600	-2.77253600	-0.14177700
O	-4.40107600	-3.09653500	2.23877000
C	-3.09919300	-3.34483600	2.58398600
C	-4.36711000	1.04680300	-2.63167600
H	-4.97292400	2.72568400	-1.45815900
C	-2.20782300	1.31659600	-3.74575500
O	-0.97575400	3.36250800	-3.50917800
C	0.25732000	3.23202600	-2.90595600
C	-5.57688900	0.17855500	-2.29867900
C	-4.71235300	-1.96427700	-1.22513300
H	-3.97124800	-3.75760800	-0.27878500
C	-0.66595100	-3.88023700	3.58340600
C	-3.36153900	0.58420900	-3.48517800
H	-1.44251300	0.93860900	-4.41207100
C	2.71998600	3.33031900	-1.80867600
H	-5.72225800	-0.50075500	-3.14188300
O	-4.48523600	-2.48573700	-2.51088000
C	-1.15791100	-4.51732400	2.40213400
O	-3.53568800	-0.65310400	-4.13953700
C	2.32318200	2.27191000	-2.68858900
C	3.98804700	2.46225000	2.30356500
C	3.17944700	0.27744000	3.89070900
C	-0.35120600	-5.46950200	1.73657800
C	0.59937700	-4.24676200	4.09614000
C	3.25452100	1.24967200	-2.98285500
C	4.01148000	3.32941400	-1.23839800
C	-6.87601900	0.98203500	-2.14661900
H	-7.07448600	1.54764100	-3.06066200
H	-6.84520300	1.69440400	-1.31849100
C	-6.77255600	0.24225500	2.92426700
H	-7.61295500	-0.38931900	2.62286700
H	-6.81088700	1.15252000	2.31953800
C	-4.29877700	4.58735200	3.38151100
H	-4.74945000	4.34399900	4.34765700
H	-5.00980800	4.29746500	2.60228200
C	-4.34870800	5.27182800	-1.80397300
H	-4.20111600	6.31906900	-1.52801800
H	-5.04043800	4.83972900	-1.07516000
H	-4.83046400	5.23552300	-2.78524900
H	-4.17162700	5.67146100	3.31953500
H	-6.91519100	0.53648300	3.96723300
H	-7.71459500	0.30459700	-1.96616000
C	4.88689800	2.30360500	-1.51800400
C	4.50460500	1.26298800	-2.39883100
H	5.20331600	0.45688800	-2.58030200
H	5.86573700	2.27805100	-1.05412900
H	2.97109200	0.45517400	-3.66387500
H	4.27362100	4.14817800	-0.57755900
C	4.41707800	0.28720600	3.28119600
H	2.84651400	-0.55304300	4.50424400
C	4.82518800	1.38028700	2.47756500
H	4.27779200	3.31106600	1.69612200

C	1.35554800	-5.19710500	3.44487200
H	0.95084800	-3.74939700	4.99305500
C	0.88370700	-5.80028300	2.25473000
H	-0.74344100	-5.93788600	0.83973100
H	1.50306800	-6.53382700	1.74828800
H	2.33735700	-5.45778200	3.81963600
H	5.79531200	1.36904100	1.99015200
H	5.08405100	-0.56036700	3.39659400
P	-3.15740900	-2.01648100	-3.32840600
N	-3.18707500	-3.12493700	-4.57841200
C	-3.21595100	-4.54324400	-4.19657400
C	-2.29469600	-2.84191300	-5.71224200
H	-3.94357100	-4.70123300	-3.40114100
H	-2.23020900	-4.90674300	-3.86985700
H	-3.52748600	-5.12490800	-5.06805100
H	-2.36761900	-1.79100100	-5.99063500
H	-2.62239600	-3.44604600	-6.56219300
H	-1.24657000	-3.08799600	-5.48863500
Au	-1.19256400	-1.61903200	-2.23236600
N	1.05855200	2.25379200	-3.22678400
N	1.84455400	4.32302200	-1.48322000
N	1.87586100	3.54179800	2.71106400
N	1.05128100	1.34149100	4.27633700
N	-1.39855900	-2.91175200	4.21309000
N	-2.40870300	-4.22776800	1.92463300
C	1.32433400	-1.98571800	-1.62324800
C	0.87639200	-1.02549300	-2.26357600
H	1.02531900	-0.09810600	-2.78919100
C	1.69067200	-3.11817200	-0.77663300
C	2.64289300	-2.67012800	0.36202900
H	2.17885200	-3.87742500	-1.39007500
H	0.78555000	-3.55724500	-0.34319400
C	3.74662400	-1.80565900	-0.29927300
O	3.71897100	-1.59076300	-1.50343300
O	4.66054900	-1.41137700	0.54285900
H	5.46134900	-0.95946800	0.04294000
C	1.90442400	-1.94452000	1.51662200
H	2.66611100	-1.52299700	2.17113700
H	1.38102700	-2.70683900	2.09477100
C	3.40540400	-3.85967800	0.96289800
O	3.67794600	-3.98331000	2.13425600
O	3.79540900	-4.72870000	0.00941400
C	4.65774600	-5.78891500	0.46167700
H	4.90825100	-6.36242800	-0.42945200
H	5.55630500	-5.37142100	0.91999000
H	4.14122400	-6.41534000	1.19314100
C	0.92809400	-0.87658900	1.08925300
C	-0.44706600	-1.14301500	1.04707800
C	1.37423500	0.39844300	0.71916800
C	-1.35963100	-0.17013700	0.62424200
H	-0.80742200	-2.12065400	1.34599700
C	0.46916400	1.37573500	0.30796800
H	2.43434800	0.62828100	0.75365800
C	-0.89936000	1.09638700	0.25090100
H	-2.42334200	-0.38546100	0.62296200
H	0.83247700	2.36294600	0.05313700
H	-1.60111500	1.86368900	-0.05568100
S	7.75790100	0.25032800	-0.07771900
O	8.63601800	-0.62115800	0.70783700
O	6.57992000	-0.45302500	-0.71490600
O	7.39525000	1.55812100	0.51228900
C	8.71010500	0.70161800	-1.60208800
F	9.10890400	-0.39444600	-2.25883500
F	7.94109600	1.43425000	-2.43003000
F	9.78893800	1.42433000	-1.27933900

1a-Int$_{5anti}$⊂AuCav

C	-5.39544300	0.92632300	2.27149600
C	-4.02951500	0.86871600	2.94941400
C	-5.29049300	0.62477700	0.78057000
H	-5.72729500	1.96200100	2.34948800
C	-3.53573100	-0.30786400	3.51377900
C	-3.23772600	2.01914800	3.07726200
C	-5.30534600	-0.67583700	0.27028200
C	-5.17435200	1.67194200	-0.14135000
C	-2.33512100	-0.36980800	4.22510500
H	-4.12970600	-1.20973300	3.42716300
C	-2.01416500	2.00122000	3.74309100
O	-3.77173600	3.23274700	2.64385100
C	-5.20535000	-0.95647100	-1.09667100
H	-5.38783100	-1.50445000	0.96476800
C	-5.07948600	1.44707100	-1.50953600
O	-5.19876100	2.97836500	0.34372200
C	-1.86423400	-1.66849000	4.87198200
C	-1.58217900	0.80779500	4.31798300
H	-1.43197200	2.90817500	3.84760000
C	-3.28884200	3.84587800	1.52116800
C	-5.21260800	-2.38324300	-1.63768100
C	-5.07694600	0.13302200	-1.96613500
H	-4.96771700	2.27384800	-2.19785800
C	-4.03627400	3.70073700	0.30092500
H	-1.15858000	-1.38897000	5.65414400
C	-1.08422900	-2.48132200	3.84766400
O	-0.43045500	0.80357700	5.11649600
C	-3.82409000	-3.00373200	-1.53324900
H	-5.43571600	-2.30779200	-2.70210500
O	-4.93418800	-0.12077800	-3.33696800
C	-1.70117900	-3.34229100	2.93737000
C	0.30331500	-2.32731100	3.74745400
C	0.80292100	0.87273600	4.53308900
C	-1.83962800	5.24221300	0.45605500
C	-3.39365600	-3.67311600	-0.38354200
C	-2.91548600	-2.90713800	-2.59673900
C	-3.66894000	0.08994400	-3.82440100
C	-2.55193400	5.05435800	-0.76737100
C	-0.99265800	-4.03910200	1.95362600
H	-2.77488400	-3.47488100	2.99512800
C	1.05117900	-2.97516600	2.76991900
O	0.92761500	-1.52290600	4.69507900
C	1.50796400	-0.35778200	4.27806500
C	-2.12739200	-4.25267900	-0.26950300
H	-4.07863600	-3.75711600	0.45151200
C	-1.63913600	-3.45887700	-2.52395200
O	-3.33835500	-2.29256400	-3.77631900
C	-2.82916000	-1.05063800	-4.05999900
C	-1.67837900	-4.99481000	0.98361100
C	0.38876500	-3.81686600	1.88216600
H	2.12152300	-2.82566600	2.70132400
C	2.59324500	2.03537500	3.72958000
C	-1.26154900	-4.12940100	-1.36389100
H	-0.95977100	-3.37295500	-3.36213000
C	-1.97018200	1.46603400	-4.47137400
H	-0.91611200	-5.70866900	0.66170900
O	1.16392800	-4.51128000	0.95377500
C	3.25777100	0.81114600	3.41071500
O	-0.01365600	-4.75231100	-1.33333900
C	-1.16005700	0.32857500	-4.77221100
C	-2.08505700	5.67680100	-1.94822300
C	-0.69304600	6.07089100	0.47240200
C	4.52805800	0.83846000	2.79205900
C	3.23682700	3.26271600	3.45294800
C	0.14645600	0.52354800	-5.27687700
C	-1.42662600	2.76524700	-4.59687800
C	-2.80483800	-5.80582200	1.63935500
H	-3.22150100	-6.51596000	0.92009500
H	-3.62762800	-5.18305600	1.99982900
C	-2.99995900	-2.46174600	5.53307500
H	-2.59671000	-3.34813600	6.03084600
H	-3.75881100	-2.80090600	4.82246400
C	-6.44622800	0.05241000	2.97277500
H	-6.52296100	0.32857200	4.02801900
H	-6.21567200	-1.01579900	2.92752900
C	-6.30562900	-3.24866700	-0.99299300
H	-7.28637100	-2.79665000	-1.16351900
H	-6.18131400	-3.35950100	0.08790200
H	-6.30260100	-4.25059200	-1.43145500
H	-7.42305700	0.19561000	2.50272400
H	-3.50395500	-1.84224200	6.27969700
H	-2.41577700	-6.36296700	2.49579100
C	-0.13125800	2.92536400	-5.03713400

C	0.64748200	1.80131100	-5.40209700
H	1.66288400	1.95090500	-5.75319300
H	0.30781600	3.91577100	-5.08284800
H	0.73571800	-0.35204200	-5.52804400
H	-2.04801800	3.60751200	-4.31482600
C	-0.24872400	6.65093600	-0.69601300
H	-0.15914800	6.19303800	1.40812200
C	-0.94502700	6.45041100	-1.91102300
H	-2.64600400	5.51897200	-2.86305000
C	4.48763400	3.26228200	2.87161700
H	2.71840300	4.18439500	3.68986100
C	5.12974300	2.05033000	2.52666800
H	4.99139000	-0.10479400	2.53403900
H	6.09951300	2.07383100	2.04174500
H	4.97551700	4.20782300	2.66432600
H	-0.56743000	6.90402500	-2.82106800
H	0.65913000	7.24350900	-0.69063000
P	1.30001200	-4.02446200	-0.61276900
N	2.57320900	-4.97604000	-1.06487900
C	2.67922800	-6.37400500	-0.64480600
C	3.31859100	-4.65221700	-2.28350700
H	2.17631000	-6.51848800	0.31041700
H	3.73767800	-6.62291000	-0.52047400
H	2.24202700	-7.04969700	-1.39169600
H	3.18149300	-3.60098400	-2.54289700
H	2.98486900	-5.27037500	-3.12768300
H	4.38455400	-4.81598600	-2.10656200
Au	1.50691500	-1.81035000	-1.20373100
N	-1.62496200	-0.93661700	-4.54023400
N	-3.25014100	1.30607500	-4.02126800
N	-3.67473200	4.27445600	-0.80796300
N	-2.23219400	4.60008800	1.59914500
N	1.33695400	2.03122600	4.27755000
N	2.67867200	-0.39000900	3.71626000
C	2.18416100	1.17852300	-1.73106100
C	1.82574500	-0.02856700	-2.17222200
H	1.38631400	0.00300000	-3.16682600
C	2.84844700	1.69132500	-0.48038800
C	2.24413700	3.10134000	-0.33731000
H	3.92509600	1.76240500	-0.64499300
H	2.67597500	1.04282700	0.37535000
C	1.87454100	3.43394900	-1.80131300
O	1.92125600	2.30210700	-2.54538200
O	1.58191600	4.50623900	-2.26617600
H	3.64689600	-0.61865900	-2.01701200
C	0.98381900	3.11984700	0.56588700
H	0.48183200	4.07042100	0.40813300
H	1.32775100	3.10969900	1.60044500
C	3.19564900	4.21185100	0.09199200
O	2.90020200	5.14785400	0.80032700
O	4.40482400	4.06728100	-0.48995900
C	5.32012500	5.15566100	-0.29543700
H	6.19775000	4.91149300	-0.89280900
H	4.87066800	6.09304400	-0.63019100
H	5.59040500	5.24939500	0.75873700
C	0.04522700	1.96724400	0.32130500
C	0.11988400	0.80669200	1.10034300
C	-0.89604400	2.01674200	-0.71214300
C	-0.71618600	-0.28198300	0.85718500
H	0.85083000	0.75173300	1.89595200
H	-1.73665000	0.93322300	-0.95488700
C	-0.96843300	2.90293200	-1.33471800
C	-1.65278200	-0.22027900	-0.17509300
H	-0.64709000	-1.16688200	1.47580500
H	-2.46740300	1.00397000	-1.74371700
H	-2.31384400	-1.05765000	-0.36203200
S	5.12770000	-1.49875100	-0.65492000
O	5.72456500	-2.80775600	-0.86429300
O	4.15773100	-1.26571000	0.41073400
O	4.62603600	-0.90322600	-2.04335300
C	6.53919600	-0.32019900	-0.34534600
F	7.00896600	-0.55086000	0.88582000
F	6.11630400	0.94709400	-0.41319800
F	7.50599200	-0.51059800	-1.23649400

1a-TS2$_{5anti}$⊂AuCav

C	-5.38997700	0.76010400	2.32351100
C	-4.01427700	0.72322200	2.98253000
C	-5.29767300	0.48527900	0.82660700
H	-5.74463300	1.78638400	2.42275400
C	-3.48937700	-0.44778800	3.53004300
C	-3.24474300	1.88844300	3.11106700
C	-5.28510300	-0.80726000	0.29631300
C	-5.22389500	1.54886900	-0.08077600
C	-2.27867200	-0.49093500	4.22538700
H	-4.06660300	-1.36051900	3.44467600
C	-2.01196600	1.89028900	3.75969300
O	-3.81339500	3.09440200	2.70083900
C	-5.20052900	-1.06546600	-1.07620100
H	-5.33379600	-1.64796100	0.97935200
C	-5.14456700	1.34669800	-1.45336500
O	-5.27493500	2.84758800	0.42313500
C	-1.77409400	-1.78473100	4.85652600
C	-1.54786900	0.70087500	4.31790500
H	-1.44809900	2.80840100	3.86708500
C	-3.36378200	3.73743700	1.58232600
C	-5.18223500	-2.48563300	-1.63512100
C	-5.11506300	0.04019900	-1.93068500
H	-5.06545700	2.18614100	-2.13084400
C	-4.13168400	3.59917200	0.37358800
H	-1.06843800	-1.49704000	5.63566300
C	-0.98481800	-2.56841100	3.81689400
O	-0.38790500	0.71386100	5.10276900
C	-3.77889800	-3.07511900	-1.55241300
H	-5.41857100	-2.40202300	-2.69610700
O	-4.98243300	-0.18490900	-3.30797100
C	-1.59171200	-3.42585000	2.89638800
C	0.39879100	-2.38512800	3.71075000
C	0.83947300	0.81051200	4.51005600
C	-1.96857300	5.18927600	0.51859600
C	-3.31886300	-3.75206600	-0.41840200
C	-2.88260900	-2.93243300	-2.62080500
C	-3.72500700	0.06654200	-3.79763600
C	-2.70238800	5.01209300	-0.69376000
C	-0.87724500	-4.08889600	1.89376500
H	-2.66219500	-3.58057300	2.95952500
C	1.15173000	-2.99777100	2.71439800
O	1.01335700	-1.58346400	4.66698200
C	1.57094000	-0.40430600	4.25629600
C	-2.03267100	-4.29067400	-0.32325900
H	-3.99437400	-3.87333100	0.41975300
C	-1.58881400	-3.44283700	-2.56764000
O	-3.33383800	-2.30528000	-3.78267800
C	-2.85366100	-1.04763400	-4.04401400
C	-1.54976200	-5.04223400	0.91177200
C	0.49811900	-3.83401500	1.81484700
H	2.21777300	-2.82258200	2.63672400
C	2.59938000	2.01097300	3.69376600
C	-1.17986700	-4.11836900	-1.42161300
H	-0.91945400	-3.32110400	-3.40923100
C	-2.06201500	1.49583000	-4.42184900
H	-0.77291300	-5.72981800	0.56800400
O	1.27803400	-4.48552500	0.86097600
C	3.29292700	0.80092600	3.38263900
O	0.08725000	-4.69908700	-1.41600400
C	-1.21467800	0.38455200	-4.71901800
C	-2.28183800	5.68039000	-1.86686900
C	-0.84421600	6.04785100	0.53205900
C	4.56047100	0.85236500	2.76041400
C	3.21142100	3.25146600	3.40258700
C	0.09110100	0.61984400	-5.20720500
C	-1.55865300	2.81145300	-4.54435400
C	-2.64682500	-5.89452700	1.56507400
H	-3.05503500	-6.60080700	0.83722500
H	-3.47994300	-5.29992000	1.94868000
C	-2.88598600	-2.61025400	5.51845900
H	-2.45777500	-3.49025600	6.00654700
H	-3.64081300	-2.96276500	4.81003100
C	-6.41160100	-0.14920300	3.02284100
H	-6.48038100	0.10683800	4.08367400

H	-6.15816900	-1.21104200	2.95562400
C	-6.24889700	-3.38238300	-0.98920100
H	-7.24110500	-2.94999900	-1.14332000
H	-6.10994600	-3.50340100	0.08881600
H	-6.22900700	-4.37859900	-1.44003200
H	-7.39750000	-0.01969800	2.56806000
H	-3.40008600	-2.00927000	6.27329200
H	-2.23240200	-6.45821800	2.40510400
C	-0.26659000	3.01189900	-4.97799600
C	0.55183900	1.91258300	-5.33158900
H	1.56421900	2.09381500	-5.67673000
H	0.13839400	4.01644500	-5.02878700
H	0.70822800	-0.23726300	-5.45497000
H	-2.20864800	3.63420900	-4.26913700
C	-0.44735200	6.67559700	-0.62861900
H	-0.29073800	6.15927000	1.45757500
C	-1.16743000	6.49056700	-1.83207700
H	-2.85942400	5.53160900	-2.77283000
C	4.45937200	3.27487500	2.81469100
H	2.67221700	4.16284600	3.63277700
C	5.13118000	2.07580200	2.48097600
H	5.04191900	-0.08105100	2.50189700
H	6.09751300	2.11685100	1.99120100
H	4.92668600	4.22928900	2.59906100
H	-0.82878300	6.98527200	-2.73618400
H	0.44009400	7.29855000	-0.62553900
P	1.39085100	-3.95870800	-0.69383000
N	2.67952800	-4.86367100	-1.18135600
C	2.82902800	-6.26785000	-0.79690200
C	3.46753600	-4.46743700	-2.35142000
H	2.28985600	-6.46586400	0.12845600
H	3.89069300	-6.47443200	-0.62988700
H	2.45693600	-6.93607000	-1.58431500
H	3.30785600	-3.41287600	-2.58047200
H	3.19320600	-5.06695700	-3.22937200
H	4.52933700	-4.59701000	-2.12914300
Au	1.60134200	-1.73852900	-1.21045400
N	-1.64728600	-0.89539000	-4.50725400
N	-3.33933700	1.29592100	-3.97963700
N	-3.80537400	4.20368600	-0.72983200
N	-2.32179600	4.51234000	1.65471100
N	1.34474200	1.98006700	4.24575400
N	2.74142700	-0.41158400	3.69412600
C	2.21171700	1.26887400	-1.72655300
C	2.13937400	0.02408000	-2.23898400
H	1.73896200	0.01526000	-3.25169700
C	2.81620800	1.80868400	-0.45763700
C	2.11179500	3.16671500	-0.28575200
H	3.88647000	1.94968400	-0.62492800
H	2.70018700	1.12667800	0.38106600
C	1.68346600	3.48366600	-1.73040400
O	1.76961700	2.34338700	-2.48270900
O	1.31006500	4.52587600	-2.19578300
H	3.50753300	-0.50442600	-2.07977700
C	0.86585200	3.07930600	0.63413400
H	0.31022300	4.00608600	0.52159600
H	1.22581900	3.04619700	1.66205500
C	2.98751400	4.33897900	0.14683000
O	2.63907900	5.23144900	0.88606600
O	4.18548600	4.29850900	-0.46883500
C	5.02970200	5.44178100	-0.25921100
H	5.92563700	5.25677500	-0.85012600
H	4.52405700	6.35037900	-0.59346900
H	5.28182000	5.54534300	0.79824900
C	-0.01161200	1.88872300	0.35168500
C	0.13562000	0.70259600	1.08093300
C	-0.97753500	1.93587300	-0.65964000
C	-0.66174500	-0.40968300	0.81566700
H	0.88604000	0.64828200	1.85847300
C	-1.77628700	0.82615500	-0.92649600
H	-1.10796100	2.84342400	-1.24105000
C	-1.62591600	-0.34903700	-0.19139300
H	-0.54415100	-1.31094700	1.40098900
H	-2.52966800	0.89443000	-1.69417100
H	-2.25808300	-1.20465000	-0.39123800
S	5.15344300	-1.37895200	-0.70530200
O	5.80423500	-2.68080100	-0.81295600
O	4.17773200	-1.14585800	0.36800600
O	4.64103400	-0.86806100	-2.07529200
C	6.52449500	-0.15940700	-0.38959900
F	6.99468000	-0.33735200	0.85379100
F	6.06819200	1.10087800	-0.48935700
F	7.51615900	-0.32698000	-1.26129800

(TfO•2a)⊂AuCav

C	-5.43257500	-0.73552700	2.57609700
C	-4.08591000	-0.38380100	3.20250300
C	-5.32338800	-0.93384100	1.06770700
H	-6.06909100	0.13822400	2.72001400
C	-3.19775200	-1.35645800	3.67060000
C	-3.70857600	0.95623700	3.36487800
C	-4.95072300	-2.15522900	0.49961200
C	-5.60856100	0.11681400	0.18578200
C	-1.99684200	-1.03963600	4.31204600
H	-3.46081500	-2.40090000	3.54828000
C	-2.52098900	1.32325600	3.98699500
O	-4.60510600	1.94653500	2.95784400
C	-4.86127600	-2.36185900	-0.88163200
H	-4.70994100	-2.97962900	1.16137100
C	-5.54803100	-0.04106300	-1.19319100
O	-5.98717900	1.35421200	0.71107500
C	-1.03055900	-2.10382800	4.82214400
C	-1.68795400	0.31683800	4.46294200
H	-2.26000500	2.36570600	4.11316300
C	-4.29894000	2.65124800	1.82740800
C	-4.42557700	-3.70139800	-1.46849600
C	-5.16710400	-1.27486200	-1.70996200
H	-5.76155000	0.79018600	-1.85163100
C	-5.04264700	2.34612600	0.63693300
H	-0.41143600	-1.62387900	5.57996900
C	-0.09299800	-2.51784400	3.69424000
O	-0.54466400	0.64897900	5.18878400
C	-2.90424000	-3.81792300	-1.46232700
H	-4.72811900	-3.69104200	-2.51610200
O	-5.07951800	-1.42616900	-3.10111700
C	-0.43625500	-3.52746900	2.79452200
C	1.13756500	-1.87107000	3.50232500
C	0.49710400	1.23174800	4.52371000
C	-3.14872600	4.25417200	0.69415600
C	-2.19037100	-4.34894500	-0.38266200
C	-2.16076300	-3.36682900	-2.56126500
C	-4.00785300	-0.76843300	-3.65258400
C	-3.88963300	3.95187700	-0.48927800
C	0.39330900	-3.92662300	1.74471500
H	-1.38589600	-4.03183900	2.92291600
C	1.98856100	-2.20523300	2.44887000
O	1.53388500	-0.93738700	4.45386700
C	1.59757300	0.39326900	4.12910900
C	-0.79712500	-4.46435400	-0.38054300
H	-2.74178500	-4.69924500	0.48163300
C	-0.77455500	-3.45743600	-2.60409200
O	-2.85363400	-2.86297600	-3.65789200
C	-2.80183300	-1.51263100	-3.87827400
C	-0.01376100	-5.04450700	0.79114700
C	1.59893700	-3.22965100	1.58928600
H	2.92222100	-1.67590500	2.29127300
C	1.59569000	3.04969300	3.70886300
C	-0.11377500	-4.01098600	-1.51510400
H	-0.22965500	-3.11251400	-3.47257000
C	-2.96264400	1.12447200	-4.36571000
H	0.90896600	-5.45612100	0.37506000
O	2.50338100	-3.63021700	0.59709700
C	2.68981100	2.21731100	3.31622000
O	1.26046200	-4.20977100	-1.61186500
C	-1.74599400	0.39376400	-4.53334900
C	-3.59530300	4.63873400	-1.68890400
C	-2.13720400	5.24075700	0.64689300
C	3.81337100	2.79097100	2.67619500
C	1.64899500	4.43896500	3.45361100
C	-0.57892000	1.07510000	-4.94369600

C	-2.98478500	2.51289500	-4.62701600
C	-0.74111700	-6.19978900	1.49242700
H	-0.95451500	-6.99660700	0.77486200
H	-1.69125400	-5.89931500	1.94128300
C	-1.74038700	-3.29223600	5.48543900
H	-1.00467400	-4.00671900	5.86511200
H	-2.40397200	-3.83266800	4.80441400
C	-6.11572900	-1.92313100	3.27146400
H	-6.22793700	-1.71882400	4.33973900
H	-5.55547500	-2.85720100	3.17215800
C	-5.12110300	-4.89281000	-0.79395600
H	-6.20546400	-4.79448800	-0.89186200
H	-4.89580800	-4.97115400	0.27333000
H	-4.81198200	-5.82892200	-1.26744500
H	-7.10742600	-2.08860200	2.84155100
H	-2.34879200	-2.94111200	6.32322500
H	-0.11532200	-6.60666900	2.29106300
C	-1.83022600	3.15390400	-5.02000200
C	-0.62416700	2.43217000	-5.17746600
H	0.27760100	2.96169400	-5.46246700
H	-1.83551700	4.22402900	-5.19715100
H	0.33708000	0.50449500	-5.04906300
H	-3.92366500	3.03892100	-4.49709400
C	-1.85255900	5.87982300	-0.54191400
H	-1.57970100	5.44835500	1.55373000
C	-2.58449500	5.57628000	-1.71377600
H	-4.16747900	4.38447700	-2.57304200
C	2.74906200	4.97545600	2.81799200
H	0.80407500	5.04786200	3.75573800
C	3.82991900	4.14927400	2.42724000
H	4.64040500	2.14069800	2.41207000
H	4.69020000	4.58962700	1.93223500
H	2.77855600	6.03818100	2.60346500
H	-2.33284400	6.07746100	-2.64217800
H	-1.03726700	6.59254100	-0.58445900
P	2.40533600	-3.20078700	-0.97273600
N	3.80879000	-3.86214000	-1.53053600
C	4.47177900	-5.00563000	-0.90592600
C	4.18460300	-3.61909200	-2.92088700
H	4.16853800	-5.09353600	0.13497300
H	5.55274500	-4.83851500	-0.93639100
H	4.23658100	-5.93856900	-1.43445000
H	3.72070500	-2.69747600	-3.28101500
H	3.87260900	-4.44764200	-3.56983700
H	5.26897500	-3.49695100	-2.98245900
Au	2.26309100	-0.96440100	-1.46496200
N	-1.70456500	-0.95260700	-4.29355400
N	-4.09172300	0.50380700	-3.90727600
N	-4.84866600	2.97609000	-0.48351400
N	-3.38471600	3.57533000	1.85802600
N	0.49310600	2.51489500	4.31842700
N	2.65641300	0.86792600	3.54916700
C	2.52594000	1.78720400	-2.16206000
C	3.17013200	0.71203900	-2.71356400
H	2.93512700	0.45227100	-3.74098200
C	2.82866200	2.52781200	-0.89326100
C	1.68774100	3.54835800	-0.77384700
H	3.81427700	2.98667800	-0.98389700
H	2.89051600	1.84251700	-0.04858600
C	1.03967300	3.50967400	-2.15869400
O	1.51298800	2.38435300	-2.83970800
O	0.25935000	4.25663600	-2.66933800
H	4.16712900	0.51255200	-2.32338800
C	0.63667700	3.14104700	0.29136700
H	-0.07866700	3.95663000	0.37077800
H	1.17049600	3.08265400	1.23795900
C	2.12584800	4.99465400	-0.51861700
O	1.45805300	5.82314200	0.05790000
O	3.33022500	5.24695700	-1.05755200
C	3.81224500	6.59297100	-0.89224800
H	4.76626800	6.62744000	-1.41576400
H	3.10392600	7.30511900	-1.32045300
H	3.94687400	6.81587100	0.16855000
C	-0.09206500	1.84771500	0.02238100
C	0.31908500	0.64230400	0.61116500
C	-1.24712700	1.85131800	-0.77122300
C	-0.43964900	-0.52102500	0.44057300
H	1.21273300	0.61403700	1.22688700
C	-2.00215500	0.69136700	-0.93925700
H	-1.57201100	2.77276600	-1.24050600
C	-1.60682400	-0.49669000	-0.32447100
H	-0.13685600	-1.43398800	0.93363800
H	-2.91291400	0.73008700	-1.52294100
H	-2.20676000	-1.39257300	-0.42521400
S	5.23196800	0.20629300	0.45435200
O	5.50466500	1.11718300	-0.68280800
O	3.78115200	-0.14539500	0.59840700
C	5.99575100	-1.39583400	-0.10141100
O	5.91664500	0.50844800	1.71933500
F	7.32375100	-1.36817000	0.02882100
F	5.51757700	-2.43285100	0.60727000
F	5.71182900	-1.61594700	-1.40573400

1a-TS1$_{5syn}$⊂AuCav

C	5.54522100	0.93695300	-1.77102800
C	4.29541800	0.80160300	-2.63344700
C	5.18187200	0.69434800	-0.31016600
H	5.86122700	1.97783400	-1.84445300
C	3.87896000	-0.44237300	-3.11813300
C	3.51494000	1.91501500	-2.97339000
C	5.22286000	-0.56891200	0.28696100
C	4.76937100	1.76812000	0.48850000
C	2.75669400	-0.61091400	-3.93156100
H	4.47646900	-1.31338200	-2.87647700
C	2.35860000	1.79210400	-3.73909600
O	3.97038800	3.19505100	-2.63410100
C	4.89593400	-0.77952500	1.63032600
H	5.53881100	-1.41598900	-0.31057100
C	4.41503300	1.61241100	1.82292400
O	4.83486200	3.03750200	-0.07872800
C	2.39135100	-1.96399300	-4.53767500
C	1.99388800	0.53265000	-4.20836800
H	1.77491500	2.66450700	-4.00460800
C	3.29720600	3.90821100	-1.68395700
C	4.95880800	-2.15756700	2.28601100
C	4.48773500	0.33642600	2.37367100
H	4.11755400	2.46032000	2.42566000
C	3.71308600	3.76970500	-0.31075900
H	1.78786700	-1.74598200	-5.41936300
C	1.49955900	-2.76531200	-3.59719700
O	0.90368400	0.41108800	-5.06354100
C	3.61608400	-2.86038200	2.11852500
H	5.08757900	-1.98563800	3.35507000
O	4.24174300	0.15573800	3.73028400
C	2.01216000	-3.55124100	-2.55985300
C	0.10597100	-2.70284600	-3.73225000
C	-0.35602600	0.46571600	-4.52147600
C	1.75081100	5.45419700	-1.04176600
C	3.30345300	-3.59406500	0.96847500
C	2.62564000	-2.75726300	3.10509100
C	2.96261500	0.27573800	4.19721400
C	2.10340300	5.24806600	0.32864700
C	1.19612400	-4.23221600	-1.64956300
H	3.08779300	-3.60951800	-2.43630700
C	-0.74392900	-3.37706900	-2.86494100
O	-0.43432400	-1.92526000	-4.75107200
C	-1.06808200	-0.76757800	-4.36024200
C	2.05707100	-4.19043000	0.75327500
H	4.06542100	-3.70466500	0.20604600
C	1.35895200	-3.30131400	2.92107400
O	2.92473700	-2.12442900	4.31106000
C	2.28508600	-0.93490800	4.56227000
C	1.75589800	-5.04345700	-0.48147600
C	-0.18878800	-4.11298500	-1.82511400
H	-1.81689100	-3.26878700	-2.95772600
C	-2.16796600	1.60505800	-3.74742600
C	1.08582900	-3.98717000	1.74449100
H	0.59874300	-3.17990400	3.68248200
C	1.18041500	1.49041900	4.92210300
H	0.96035000	-5.73083000	-0.18276900

O	-1.06458600	-4.60501700	-0.85191100
C	-2.87136900	0.37268500	-3.56352700
O	-0.22111600	-4.45466700	1.56885600
C	0.53129500	0.28861900	5.34259100
C	1.42054400	5.95835700	1.34164100
C	0.75809700	6.40570600	-1.36602400
C	-4.18230200	0.38949200	-3.03818100
C	-2.80753000	2.82408900	-3.43315400
C	-0.74940800	0.35991100	5.93588300
C	0.52856900	2.73284400	5.09644800
C	2.95887400	-5.90264900	-0.90174600
H	3.30067200	-6.51263400	-0.06131700
H	3.80795500	-5.30480300	-1.24371000
C	3.62267900	-2.75630800	-5.00212900
H	3.30702300	-3.67779200	-5.49895600
H	4.28631700	-3.03838900	-4.17974500
C	6.71115400	0.06527300	-2.25723700
H	6.95797700	0.31484700	-3.29260700
H	6.49314400	-1.00565700	-2.22147500
C	6.14647900	-3.00140700	1.80337900
H	7.08393000	-2.46979900	1.98766400
H	6.10463800	-3.23216900	0.73516900
H	6.17391700	-3.95179600	2.34327800
H	7.59402200	0.24149000	-1.63665700
H	4.20856500	-2.16272700	-5.70911600
H	2.67345400	-6.56556400	-1.72271800
C	-0.72702300	2.77164100	5.66230100
C	-1.36784400	1.58137500	6.08309300
H	-2.36352900	1.63426700	6.50969100
H	-1.23754200	3.72184500	5.78172900
H	-1.23050900	-0.56550700	6.22858000
H	1.04148200	3.62993800	4.76698000
C	0.13815700	7.11736000	-0.36037800
H	0.51499700	6.55398900	-2.41237900
C	0.45350400	6.87883500	0.99784000
H	1.69232600	5.76577600	2.37363600
C	-4.09527100	2.81169600	-2.94204900
H	-2.25428200	3.74390700	-3.58373800
C	-4.78110700	1.59322000	-2.73411400
H	-4.68703900	-0.55145600	-2.85836400
H	-5.77555700	1.60233400	-2.30536900
H	-4.58972400	3.74504400	-2.69374600
H	-0.07346500	7.42552700	1.77215200
H	-0.60820700	7.86438400	-0.60944500
P	-1.15951500	-3.62070400	0.49618000
N	-2.65167400	-3.81193300	1.14469100
C	-3.72726300	-2.97219300	0.59199800
C	-3.08484400	-5.06520400	1.76608900
H	-4.24920900	-3.48185200	-0.22569100
H	-3.33038000	-2.02496800	0.22530800
H	-4.44134900	-2.72879200	1.38011200
H	-2.23492800	-5.57109700	2.22422900
H	-3.55465000	-5.73280300	1.03288900
H	-3.81661400	-4.82774500	2.54358500
Au	-0.21080800	-1.58096800	-0.06063600
N	1.11548600	-0.93077000	5.12960000
N	2.42344500	1.44922800	4.35194500
N	3.11420400	4.38652800	0.66550900
N	2.35424800	4.73166200	-2.03758400
N	-0.88363100	1.61522500	-4.21838100
N	-2.27664000	-0.81611700	-3.88462600
C	0.41023500	1.16505100	-0.65857700
C	1.02393400	0.06762900	-0.55872600
H	2.09409500	-0.11007400	-0.57934000
C	0.04309900	2.57663900	-0.59793900
C	-1.12081800	2.75060200	0.40602400
H	-0.22800500	2.95969500	-1.58445900
H	0.92833100	3.10770600	-0.24005700
C	-2.16053600	1.73687400	-0.10043700
O	-1.72288600	0.74795900	-0.70360600
O	-3.39154000	1.99046100	0.16952600
H	-4.00721200	1.12729700	0.05139700
C	-0.68112000	2.38914000	1.87342300
H	0.36389400	2.07126900	1.84907200
H	-0.73470400	3.31154800	2.45299100
C	-1.76606800	4.13805000	0.36564700
O	-2.08267200	4.78585900	1.33741500
O	-2.00966500	4.51417900	-0.90129600
C	-2.88175600	5.64638700	-1.05272700
H	-2.86477500	5.89289000	-2.11367100
H	-3.89063300	5.37034500	-0.73708500
H	-2.53056100	6.48480900	-0.45255300
C	-1.51627600	1.31232800	2.52929000
C	-2.82079500	1.58852200	2.96174000
C	-1.03447700	0.00653800	2.64631700
C	-3.64344300	0.56916300	3.43402500
H	-3.20225800	2.60079100	2.87646800
C	-1.85052500	-1.01594700	3.13681000
H	-0.01124400	-0.21378600	2.35708800
C	-3.16374600	-0.74049900	3.50935000
H	-4.67087600	0.78332500	3.70717300
H	-1.46203400	-2.02505300	3.21503400
H	-3.82080000	-1.53559900	3.84440300
S	-6.04777200	-0.14401200	0.72451100
O	-6.67871600	1.17182000	0.87287400
O	-4.69753300	-0.08344200	0.03222600
O	-6.07662900	-1.06655000	1.86970400
C	-7.02495300	-1.02629000	-0.58426300
F	-6.41532300	-2.17407100	-0.93371000
F	-8.25372900	-1.31267100	-0.14611300

1a-Int$_{5syn}$⊂AuCav

C	-4.77039200	-2.89906800	-2.06565400
C	-4.54708300	-2.63614900	-0.58035300
C	-3.59762100	-2.36948700	-2.88531300
H	-5.63866900	-2.30635600	-2.35455800
C	-3.82950200	-3.52408900	0.22556800
C	-5.05303000	-1.48118800	0.03182800
C	-2.46315800	-3.13770200	-3.16323200
C	-3.63155000	-1.06705300	-3.39915400
C	-3.61988500	-3.31222000	1.59011600
H	-3.42684500	-4.42246500	-0.22737800
C	-4.85735500	-1.21547300	1.38467900
O	-5.83767900	-0.61486900	-0.72837100
C	-1.40316100	-2.66332600	-3.94088500
H	-2.41016700	-4.14714400	-2.77145800
C	-2.59559800	-0.54174900	-4.16253000
O	-4.79716300	-0.32343000	-3.20913900
C	-2.86473700	-4.30415500	2.46662600
C	-4.13649400	-2.13379300	2.14130700
H	-5.25909900	-0.31718200	1.83523800
C	-5.32663800	0.60929500	-1.06592100
C	-0.16661800	-3.50029100	-4.25574500
C	-1.49805800	-1.35390500	-4.42530500
H	-2.64650000	0.46644100	-4.55172800
C	-4.76983400	0.76253800	-2.38352900
H	-3.17298500	-4.10524100	3.49313800
C	-1.36625900	-4.03607700	2.40772300
O	-3.97341100	-1.93443500	3.51310500
C	0.85627300	-3.34403500	-3.13734200
H	0.28234600	-3.06578400	-5.14890900
O	-0.47516200	-0.90106600	-5.25713600
C	-0.55902900	-4.58688200	1.40946300
C	-0.74202000	-3.22249500	3.36331200
C	-3.10958400	-0.96508000	3.93413800
C	-4.95646100	2.81767700	-0.65959300
C	0.82058300	-4.15359400	-1.99980700
C	1.86020500	-2.36726700	-3.19674000
C	0.34216800	0.08546200	-4.77987500
C	-4.40508100	2.96938700	-1.96837500
C	0.82082200	-4.38150300	1.35230200
H	-1.02276200	-5.20875100	0.65297500
C	0.63019600	-2.98609000	3.34325200
O	-1.51644200	-2.71161600	4.40890700
C	-1.81812500	-1.37711800	4.42506900
C	1.72446600	-4.03121000	-0.94315500
H	0.06307200	-4.92550300	-1.94298700
C	2.76230400	-2.18351900	-2.14915400
O	1.99844900	-1.61918600	-4.36707600
C	1.65892400	-0.28740600	-4.34430400

C	1.68971300	-4.97333200	0.25299500
C	1.39577100	-3.57239700	2.33741500
H	1.08985100	-2.36385800	4.10059800
C	-2.62608100	1.19300500	4.47680000
C	2.67535300	-3.00389100	-1.02505100
H	3.52755300	-1.42209400	-2.22014300
C	0.80190400	2.26360800	-4.31608200
H	2.70780300	-5.01186600	0.64732200
O	2.77806100	-3.37844400	2.34115500
C	-1.35873300	0.78321000	4.99080700
O	3.64576100	-2.86053100	-0.03488900
C	2.13381600	1.90425800	-3.94124200
C	-3.96040600	4.24271700	-2.39325700
C	-5.03505700	3.94098100	0.19657500
C	-0.48812400	1.74880900	5.54700700
C	-2.99937000	2.55563400	4.54230600
C	3.02086100	2.90618300	-3.48825500
C	0.37820900	3.60685600	-4.18816400
C	1.29591200	-6.41018700	-0.11650000
H	1.97051900	-6.80017100	-0.88342500
H	0.27679900	-6.49006400	-0.50358900
C	-3.22115200	-5.76478900	2.15213800
H	-2.69573100	-6.43521300	2.83784700
H	-2.95605700	-6.06171600	1.13368500
C	-5.09395600	-4.36956800	-2.36701000
H	-5.97331000	-4.68500000	-1.79863200
H	-4.27676600	-5.05033900	-2.11235600
C	-0.50022400	-4.96564200	-4.56906100
H	-1.18618700	-5.01732800	-5.41890100
H	-0.97629200	-5.48688000	-3.73366600
H	0.41117000	-5.51396500	-4.82339400
H	-5.30419600	-4.49470600	-3.43268300
H	-4.29713400	-5.92002800	2.26908500
H	1.36140600	-7.05204900	0.76617900
C	1.25166900	4.55495700	-3.69927500
C	2.57934300	4.20410500	-3.35518200
H	3.24745900	4.95608100	-2.95210400
H	0.92164700	5.57992800	-3.56566900
H	4.02763800	2.62503200	-3.21439700
H	-0.64389900	3.84627700	-4.46131000
C	-4.59146500	5.17080200	-0.24240300
H	-5.45214800	3.79792200	1.18786100
C	-4.05480500	5.32284900	-1.54291400
H	-3.54441200	4.32883400	-3.39126300
C	-2.13907200	3.47456900	5.10563400
H	-3.97004100	2.83799700	4.14900300
C	-0.87790000	3.07043700	5.60523500
H	0.47669100	1.41708300	5.91371600
H	-0.21097100	3.81108900	6.03476200
H	-2.42534500	4.52017700	5.16186900
H	-3.69027200	6.29512900	-1.85374900
H	-4.63238700	6.03171500	0.41580500
P	3.42932700	-2.13820200	1.44402500
N	4.97251100	-2.11996600	2.01470700
C	5.62980700	-0.87301900	2.40008700
C	5.83105100	-3.29937600	1.94656500
H	4.91857700	-0.04663200	2.40032800
H	6.43696400	-0.62564500	1.70122700
H	6.05143200	-0.97458200	3.40644000
H	5.23827700	-4.18909300	1.73373000
H	6.33446100	-3.44174800	2.90912800
H	6.59009700	-3.18556900	1.16255900
Au	2.09836700	-0.27413700	1.73131400
N	2.53435100	0.59394100	-3.95968000
N	-0.07461300	1.31627200	-4.76173600
N	-4.31913600	1.90139100	-2.81881300
N	-5.41179300	1.60018300	-0.22840400
N	-3.49140900	0.27773000	3.94380900
N	-0.97456700	-0.53045100	4.93724300
C	0.66747400	2.43415000	1.89648000
C	0.78733800	1.17664600	2.28376200
H	-0.03203900	0.85204400	2.91502300
C	-0.42078400	3.45319600	2.08529300
C	-0.39557400	4.22535000	0.74815400
H	-0.18803000	4.12868500	2.91348500
H	-1.38211200	2.98042100	2.28410600
C	1.07823400	4.10619400	0.35055200
O	1.63019400	3.04348800	1.02172100
O	1.70554500	4.76110600	-0.43693900
H	2.69992600	2.20097100	0.11073200
C	-1.28627700	3.56172500	-0.34365500
H	-1.10443700	4.08513400	-1.28565900
H	-2.31804000	3.76315400	-0.05879200
C	-0.75243600	5.70043700	0.88340900
O	-1.60997600	6.28094400	0.25523000
O	0.02467000	6.29544800	1.80928000
C	-0.18549400	7.70722300	1.97761400
H	0.52683400	8.02081400	2.73949000
H	-0.00068100	8.23153600	1.03752900
H	-1.21046800	7.90537400	2.30029500
C	-1.06412200	2.07642800	-0.50739700
C	-1.93861000	1.13985300	0.05977300
C	0.06567900	1.60826000	-1.18698800
C	-1.67874600	-0.22895400	-0.03514100
H	-2.82120300	1.48013500	0.59256500
C	0.34335500	0.24653600	-1.25648500
H	0.75042600	2.31686400	-1.63702000
C	-0.52995900	-0.68126600	-0.68494100
H	-2.36194800	-0.94025200	0.41140700
H	1.25264200	-0.08667400	-1.73441100
H	-0.31807200	-1.74324900	-0.74938400
S	4.82859800	2.22062300	-0.25921800
O	5.10536700	2.13793700	1.17014400
O	5.24364100	3.37514500	-1.04159000
O	3.32345700	1.80440900	-0.57025000
C	5.60676300	0.73752500	-1.07256900
F	6.91629300	0.77023400	-0.81692000
F	5.40427600	0.76018600	-2.38777300
F	5.09851900	-0.39639200	-0.57508600

1a-TS2$_{5syn}$⊂AuCav

C	-4.82174700	-1.66783100	3.14041800
C	-3.47736200	-1.14585100	3.63793600
C	-4.83280300	-1.75637400	1.61702900
H	-5.56154500	-0.91310800	3.40857700
C	-2.39247500	-1.99754100	3.86759800
C	-3.27914700	0.22127800	3.87612900
C	-4.41408400	-2.89601000	0.92443100
C	-5.28144200	-0.67256200	0.85182600
C	-1.15538200	-1.54218600	4.33011200
H	-2.51940600	-3.05818500	3.68436000
C	-2.06223600	0.72643500	4.32558800
O	-4.36109900	1.08882000	3.71979900
C	-4.44809500	-2.99014500	-0.47011700
H	-4.06326000	-3.74920000	1.49348300
C	-5.31198000	-0.70547000	-0.53725500
O	-5.79696600	0.43411600	1.52818200
C	0.02256600	-2.47130400	4.59347700
C	-1.01751300	-0.16606500	4.54066200
H	-1.93489600	1.78590000	4.50580900
C	-4.34288500	1.95954200	2.66520900
C	-4.01656000	-4.24825400	-1.21900700
C	-4.89569000	-1.86750800	-1.17697600
H	-5.66193500	0.14605600	-1.10540600
C	-5.10525100	1.60927600	1.49696900
H	0.69252400	-1.93773300	5.26747200
C	0.80588500	-2.71210100	3.30898900
O	0.19812500	0.28634300	5.05009400
C	-2.51507500	-4.21277700	-1.47646300
H	-4.49880100	-4.20607600	-2.19584000
O	-4.98548400	-1.96270800	-2.56781400
C	0.44842100	-3.73312000	2.42577400
C	1.92149400	-1.92918200	2.97483100
C	1.01188000	1.02494700	4.24108500
C	-3.77529300	3.93752600	1.69293500
C	-1.59205300	-4.66569600	-0.52939000
C	-2.00062200	-3.69425300	-2.67140200
C	-4.08873100	-1.23828500	-3.30168400
C	-4.53735500	3.59116200	0.53553400
C	1.17211900	-4.01999700	1.26840300

H	-0.41651400	-4.34135400	2.66140000
C	2.67529100	-2.17431900	1.82661800
O	2.34473100	-0.95886100	3.89122100
C	2.15525900	0.37337500	3.64857300
C	-0.20916400	-4.61231900	-0.72136600
H	-1.96807000	-5.08294700	0.39688500
C	-0.63033800	-3.60340300	-2.89977200
O	-2.89247700	-3.29442900	-3.66958500
C	-2.99368000	-1.94656500	-3.90350900
C	0.77250200	-5.13921800	0.31722800
C	2.28826400	-3.22303500	0.99029300
H	3.55420100	-1.58453400	1.59189400
C	1.68772600	3.01863800	3.37002300
C	0.25218100	-4.04149100	-1.91602000
H	-0.25946800	-3.19812500	-3.83287500
C	-3.32209300	0.70623500	-4.19899700
H	1.67694700	-5.42233600	-0.22614300
O	3.06169700	-3.52152700	-0.13414500
C	2.84897800	2.39001900	2.82777500
O	1.61009000	-3.98765700	-2.20542700
C	-2.24507200	-0.00023500	-4.81751100
C	-4.61946900	4.50260700	-0.54261400
C	-3.10107400	5.18043700	1.73595000
C	3.79440500	3.16766300	2.11786700
C	1.49365100	4.40829400	3.19171100
C	-1.26928800	0.71713000	-5.54752700
C	-3.39314400	2.11361200	-4.32080000
C	0.26990200	-6.39545700	1.04190000
H	0.03832200	-7.18057300	0.31705300
H	-0.63190700	-6.21813200	1.63377700
C	-0.39517300	-3.77314300	5.29351100
H	0.48459200	-4.39026900	5.49523500
H	-1.09165900	-4.37472500	4.70290700
C	-5.23549900	-2.98181200	3.81873000
H	-5.26962900	-2.84932400	4.90357300
H	-4.54912700	-3.80739900	3.61062200
C	-4.47617100	-5.54079100	-0.52938200
H	-5.56393700	-5.53881800	-0.41913700
H	-4.04639200	-5.67188300	0.46767600
H	-4.18830700	-6.40912100	-1.12849000
H	-6.22755900	-3.28495500	3.47311000
H	-0.88534600	-3.54407600	6.24372300
H	1.04084000	-6.76506000	1.72321400
C	-2.41637200	2.79106600	-5.01923900
C	-1.35168200	2.09043700	-5.63656300
H	-0.58162600	2.64799900	-6.15646600
H	-2.44575600	3.87343600	-5.08544200
H	-0.45428700	0.16080900	-5.99760500
H	-4.21153500	2.62638500	-3.82698100
C	-3.19693000	6.04963300	0.66978900
H	-2.52362200	5.41651600	2.62357600
C	-3.96066000	5.71073500	-0.47251300
H	-5.20499500	4.21407000	-1.40888700
C	2.43384100	5.14650600	2.50383700
H	0.60374200	4.85803000	3.61903500
C	3.58570000	4.52314900	1.96641800
H	4.66458600	2.67815100	1.70279300
H	4.31346300	5.11936500	1.42515800
H	2.29270000	6.21470900	2.37092900
H	-3.99905800	6.40212500	-1.30631700
H	-2.66919500	6.99672900	0.68944000
P	2.66657700	-2.85762000	-1.60094800
N	3.97005000	-3.17786900	-2.54594000
C	4.78077900	-2.10699100	-3.12894300
C	4.58297300	-4.50558600	-2.55629800
H	4.24474000	-1.15806900	-3.07717900
H	4.97251200	-2.33996700	-4.18219600
H	5.72851200	-1.98827100	-2.59854300
H	3.87803800	-5.25219300	-2.18959600
H	5.48180100	-4.52299900	-1.92844300
H	4.85925700	-4.76851700	-3.58284500
Au	2.15503600	-0.66253500	-1.18221500
N	-2.10968300	-1.35164600	-4.64925900
N	-4.25123800	0.04332500	-3.44851800
N	-5.19601800	2.39430300	0.46479700
N	-3.69154700	3.08125000	2.75850800
N	0.77464500	2.29348800	4.08524900
N	3.04279400	1.04064400	2.97251900
C	1.66015600	2.34169800	-0.93309100
C	2.24411900	1.27259200	-0.35612900
H	2.15731200	1.29221300	0.72413400
C	1.03448800	3.55618100	-0.30960400
C	0.04281500	4.01921800	-1.38767400
H	1.81705200	4.30372500	-0.15379600
H	0.57698000	3.33455000	0.65263600
C	0.69862700	3.46031500	-2.66041400
O	1.60141500	2.48137900	-2.30521700
O	0.48497400	3.75373800	-3.80295700
H	3.60179400	1.21603200	-0.61500900
C	-1.36797300	3.39605600	-1.18159900
H	-1.95772400	3.57831500	-2.08267700
H	-1.83440500	3.95652600	-0.37276400
C	-0.06375500	5.53199800	-1.54973600
O	-1.09510000	6.15568900	-1.65874200
O	1.15901200	6.09555000	-1.58476600
C	1.17931600	7.51551300	-1.81340200
C	-1.33284000	1.92359800	-0.84595000
C	-1.45948500	1.47621800	0.47738400
C	-1.12234500	0.97592200	-1.85263800
C	-1.37380800	0.11684800	0.78583500
H	-1.62250600	2.19432400	1.27522500
C	-1.02419300	-0.37939100	-1.54793500
H	-1.00260300	1.30318800	-2.87772900
C	-1.15269300	-0.81732000	-0.22661900
H	-1.47329900	-0.21175200	1.81244300
H	-0.84159800	-1.09271300	-2.33887900
H	-1.08111100	-1.87353600	0.00821400
S	5.77911600	0.18922900	-0.49790500
O	6.77712100	0.12677000	-1.56440500
O	5.05941400	-1.04439800	-0.14110300
O	4.84260000	1.40317000	-0.64855000
C	6.71306300	0.64976300	1.04165800
F	7.73436200	-0.19275600	1.21195100
F	5.90629800	0.57788500	2.10709800
F	7.18458300	1.89999700	0.94571400
H	2.23192600	7.79410800	-1.83077600
H	0.70074500	7.75111200	-2.76626000
H	0.65321300	8.03771500	-1.01080000

(TfO•2a')⊂AuCav

C	3.15402600	3.18428500	3.43159400
C	2.06302400	3.73211100	2.51876300
C	3.44774800	1.71861300	3.13324800
H	2.74551200	3.21343600	4.44212400
C	2.35497500	4.23018200	1.24578100
C	0.72510500	3.78437800	2.93242700
C	4.41377500	1.30849700	2.20944400
C	2.74983500	0.71659700	3.81827600
C	1.39794600	4.81572700	0.41296300
H	3.37879300	4.17458400	0.89554000
C	-0.26823900	4.35070400	2.13792700
O	0.39101200	3.33929100	4.21256100
C	4.72335500	-0.03645800	1.98790600
H	4.95872400	2.06500100	1.65697200
C	3.01518700	-0.63536100	3.63459500
O	1.83612600	1.12419300	4.79087500
C	1.75448700	5.34721500	-0.97322200
C	0.08349700	4.87265800	0.89661600
H	-1.28746500	4.41875500	2.49733100
C	-0.27274100	2.14520600	4.31372700
C	5.75836500	-0.48581200	0.95706800
C	4.01293100	-0.99138300	2.73116100
H	2.48158900	-1.39266600	4.19381900
C	0.50168500	0.95959900	4.57327600
H	0.97858100	6.06473600	-1.24161300
C	1.65903500	4.19864500	-1.97123300
O	-0.87459900	5.56202100	0.15149000
C	5.04121900	-0.70728000	-0.37123600
H	6.12227400	-1.46024700	1.28394200
O	4.40598700	-2.31487100	2.62034000

C	2.73053800	3.34419900	-2.24425000
C	0.44021200	3.92228400	-2.60410300
C	-1.89829600	4.86229700	-0.41924900
C	-2.17894200	0.90262500	4.39018200
C	4.78764700	0.34640500	-1.25521900
C	4.52980400	-1.97043200	-0.70518300
C	3.55471600	-3.24662900	2.08250000
C	-1.40462200	-0.28349100	4.57650700
C	2.61796800	2.22986500	-3.08206600
H	3.68856200	3.54808300	-1.78060200
C	0.26799200	2.81070900	-3.41792000
O	-0.60283000	4.82939600	-2.42997200
C	-1.74280800	4.44485800	-1.78829400
C	4.02029600	0.19718000	-2.41392500
H	5.19528500	1.32435100	-1.02626100
C	3.70930000	-2.15546500	-1.81367800
O	4.86985600	-3.06510800	0.08243900
C	3.84329100	-3.69073900	0.75303900
C	3.80823100	1.33176600	-3.41552900
C	1.35400300	1.96765600	-3.63065300
H	-0.69538600	2.59575200	-3.86313900
C	-4.02190500	4.04260600	-0.41959300
C	3.45716000	-1.06682500	-2.63514800
H	3.23621400	-3.11074200	-2.00253300
C	1.87356500	-4.76446700	2.22628500
H	3.56308300	0.85179900	-4.36623300
O	1.13273600	0.81354200	-4.37644700
C	-3.85214400	3.59046100	-1.76383100
O	2.57377700	-1.25398200	-3.70983600
C	2.20035000	-5.24742400	0.92083900
C	-2.05795100	-1.52899300	4.71007000
C	-3.58986800	0.82156300	4.40490400
C	-4.93086400	2.96675500	-2.43093000
C	-5.27411000	3.88012600	0.21856100
C	1.45764500	-6.31216200	0.36683400
C	0.80071400	-5.34706500	2.93552000
C	5.09443300	2.14236100	-3.64443100
H	5.90199200	1.47871300	-3.96429900
H	5.43388900	2.66219300	-2.74458300
C	3.10144400	6.08069200	-1.01129700
H	3.28308500	6.47691700	-2.01410800
H	3.94820800	5.43861600	-0.75359700
C	4.41692000	4.05900800	3.42092800
H	4.16563200	5.08413500	3.70661800
H	4.90185700	4.10058400	2.44170500
C	6.96473000	0.45528000	0.85093400
H	7.45352000	0.55280800	1.82428500
H	6.69766700	1.46164400	0.51551600
H	7.68943000	0.05451800	0.13692500
H	5.14803400	3.66748800	4.13341200
H	3.09506100	6.91426100	-0.30386500
H	4.93138300	2.89576800	-4.41987100
C	0.08478100	-6.37731000	2.36673700
C	0.41612300	-6.86290300	1.07999600
H	-0.17021400	-7.66619900	0.64584800
H	-0.74680200	-6.82059700	2.90571000
H	1.71222400	-6.64577000	-0.63240600
H	0.56656900	-4.95555500	3.91951300
C	-4.20412000	-0.40143700	4.58362200
H	-4.15659800	1.73952700	4.29001300
C	-3.43561800	-1.58149200	4.71773400
H	-1.44683400	-2.41871100	4.80818900
C	-6.32216800	3.30315000	-0.46723100
H	-5.37804800	4.23529700	1.23801600
C	-6.14829100	2.84537500	-1.79596200
H	-4.77006100	2.61590600	-3.44344900
H	-6.98626000	2.39315600	-2.31687700
H	-7.28671700	3.18494000	0.01548800
H	-3.93791300	-2.53731500	4.82285500
H	-5.28723000	-0.46344900	4.60204900
P	1.08050000	-0.61564800	-3.51106700
N	0.16145200	-1.55728300	-4.49507100
C	-1.28127900	-1.29937000	-4.48740500
C	0.55553900	-2.87960200	-4.98298100
H	-1.51571400	-0.37160400	-3.96244900
H	-1.79900800	-2.10705100	-3.96565600
H	-1.64469100	-1.22118800	-5.51823000
H	1.63468800	-2.99656300	-4.91775400
H	0.25034400	-2.96824200	-6.03188400
H	0.07913100	-3.66094300	-4.38646700
Au	0.64903100	-0.02777000	-1.29258400
N	3.20411600	-4.67263400	0.19314600
N	2.59604100	-3.75440100	2.79784400
N	-0.04116800	-0.21744800	4.66660000
N	-1.56924300	2.11828200	4.21914900
N	-2.99633200	4.65870800	0.24744100
N	-2.67570000	3.80552500	-2.42996600
C	-0.82867800	0.35640800	0.84471200
C	0.47628600	0.79862700	0.79202400
H	0.64842400	1.85260300	0.59407800
C	-2.08013600	1.05280900	0.38240400
C	-3.12084900	-0.07700800	0.29474900
H	-1.95274500	1.56974400	-0.56762400
H	-2.35933500	1.78603400	1.14894100
C	-2.46979200	-1.20664200	1.10073900
O	-1.13485100	-0.82308800	1.40281400
O	-2.91296800	-2.21771000	1.53846100
C	-4.48262600	0.36645700	0.92971200
H	-4.28580000	0.55518200	1.98707600
H	-4.71881100	1.32904700	0.47213100
C	-3.36991100	-0.47846900	-1.16882400
O	-3.11773900	0.25219700	-2.10729600
O	-4.00054300	-1.64190700	-1.22644300
C	-4.41845800	-2.14114100	-2.50506700
H	-5.46919500	-2.41594700	-2.39940300
H	-4.29206900	-1.37584700	-3.27077300
H	-3.81435900	-3.01833800	-2.73150700
H	1.23078300	0.26339000	1.36227900
C	-5.67013100	-0.55506300	0.77849500
C	-6.57266900	-0.35767000	-0.27378200
C	-5.90466700	-1.60522700	1.67250500
C	-7.67993000	-1.18882600	-0.43590700
H	-6.39048600	0.44550900	-0.97918700
C	-7.01189800	-2.43814700	1.51493300
H	-5.20099500	-1.78874500	2.47343800
C	-7.90412300	-2.23408600	0.46173700
H	-8.36806500	-1.01902200	-1.25931000
H	-7.17417300	-3.25317100	2.21411000
H	-8.76591700	-2.88367000	0.34114000
S	-0.36984600	-3.21628800	-1.05090400
O	-0.10064800	-3.46765000	0.36941300
O	-1.22467600	-2.01900400	-1.32099100
O	0.78149800	-3.33688400	-1.97599000
C	-1.50854600	-4.58322400	-1.59036500
F	-0.93236300	-5.78707700	-1.48803400
F	-1.85552500	-4.40927700	-2.89297600
F	-2.63382000	-4.57836500	-0.86752900

1a-TS1$_6$⊂AuCav

C	2.53809800	-3.13895800	4.13192400
C	2.67802700	-1.62056800	4.05113800
C	1.91624600	-3.70652300	2.86004500
H	1.82278200	-3.33362900	4.93157500
C	3.81375400	-0.99670400	3.52954000
C	1.65827700	-0.78977800	4.53733900
C	2.68408500	-4.03851800	1.73977400
C	0.53357500	-3.91995800	2.77547300
C	3.97577500	0.39210400	3.51371200
H	4.61265000	-1.61706400	3.13969600
C	1.76576700	0.59737000	4.52974600
O	0.55294000	-1.38980200	5.12615200
C	2.13095100	-4.55837000	0.56579000
H	3.75671400	-3.88833400	1.78584800
C	-0.06417900	-4.43036100	1.62670800
O	-0.25255900	-3.65871600	3.89320200
C	5.22291900	1.05885700	2.94255400
C	2.92977400	1.16719700	4.02754900
H	0.96795800	1.21288000	4.92440600
C	-0.64213800	-1.35388900	4.45251500
C	2.98416400	-4.92597200	-0.64497300

C	0.73972600	-4.72649000	0.53221500
H	-1.13533000	-4.57845800	1.58557300
C	-1.08180600	-2.56341800	3.82458200
H	5.27364400	2.05520600	3.38204700
C	5.04103900	1.25359400	1.44243800
O	3.11382400	2.55272500	4.10821000
C	3.19634500	-3.70234100	-1.52853600
H	2.39698000	-5.63145000	-1.23327500
O	0.16660000	-5.25592100	-0.62622300
C	5.36738100	0.25644600	0.52034900
C	4.49410200	2.44116900	0.93708000
C	2.34998400	3.34946400	3.30706000
C	-2.56407700	-0.32192900	3.81230000
C	4.22652500	-2.78651000	-1.29611500
C	2.33749100	-3.44471900	-2.60607500
C	-0.55025400	-4.40034800	-1.42074100
C	-3.01645700	-1.53745300	3.20721200
C	5.18605200	0.40266600	-0.85760100
H	5.79017700	-0.66928000	0.89045400
C	4.26961600	2.62592600	-0.42441700
O	4.23166600	3.47692800	1.82289300
C	2.92777700	3.81436900	2.07581500
C	4.42891500	-1.65045800	-2.08512100
H	4.90567000	-2.97185400	-0.47272500
C	2.47980800	-2.31015900	-3.40170400
O	1.34469600	-4.36890400	-2.90626800
C	0.05310300	-3.97092700	-2.64754100
C	5.58578000	-0.68599400	-1.84684600
C	4.61820600	1.60358000	-1.29674900
H	3.85023100	3.55664000	-0.78502600
C	0.45986900	4.53441500	2.85251200
C	3.51783600	-1.43231200	-3.12362300
H	1.79659700	-2.13539600	-4.22304900
C	-2.42114900	-3.19605200	-1.92983200
H	5.78013200	-0.18795700	-2.79987900
O	4.45492900	1.83394100	-2.67343000
C	1.01685000	4.95674300	1.60556900
O	3.68453700	-0.32023000	-3.97067100
C	-1.84048600	-2.82855800	-3.18576400
C	-4.27592100	-1.56979200	2.56678500
C	-3.38356400	0.82899500	3.75760800
C	0.25661500	5.78826300	0.75007700
C	-0.83070100	4.97582800	3.22498000
C	-2.57606600	-2.01982500	-4.08460600
C	-3.69999000	-2.70150500	-1.58379200
C	6.88542600	-1.40314200	-1.45241500
H	7.16072600	-2.12558400	-2.22502400
H	6.80523800	-1.94681200	-0.50794900
C	6.52174100	0.32427600	3.30188600
H	7.38465600	0.86468900	2.90264800
H	6.56140200	-0.69635600	2.91122600
C	3.85414200	-3.83575500	4.50795900
H	4.23172900	-3.43932500	5.45443600
H	4.64032000	-3.70234000	3.75905100
C	4.29902500	-5.61987900	-0.26094700
H	4.08800800	-6.51962200	0.32277500
H	4.95867800	-4.98767200	0.34009900
H	4.84897000	-5.90986700	-1.16062400
H	3.68905500	-4.91060400	4.62159500
H	6.62322600	0.26142100	4.38837400
H	7.69599500	-0.67730700	-1.34749100
C	-4.38329100	-1.88733600	-2.46202900
C	-3.82384900	-1.55897100	-3.71994500
H	-4.38404000	-0.91569500	-4.38974700
H	-5.34396300	-1.47190500	-2.18798900
H	-2.12713100	-1.77794800	-5.04299600
H	-4.12221200	-2.96948100	-0.62234000
C	-4.60400500	0.76901200	3.11941800
H	-3.01564100	1.73935200	4.21911400
C	-5.05592500	-0.43366000	2.52389000
H	-4.60379000	-2.50103100	2.11964000
C	-1.54526400	5.79667200	2.38067900
H	-1.23119600	4.63715500	4.17393900
C	-1.00134800	6.19888000	1.13730800
H	0.69963300	6.09850000	-0.19070800
H	-1.58978800	6.83424000	0.48370100
H	-2.54626200	6.11228800	2.64714500
H	-6.02177700	-0.43910300	2.03299700
H	-5.23148600	1.65126500	3.05111300
P	3.21482100	1.16170300	-3.48740000
N	3.36202800	1.98628200	-4.93710600
C	3.31021400	3.45233000	-4.85085600
C	2.58952400	1.44460400	-6.06492300
H	3.95761300	3.80242600	-4.04742600
H	2.28786900	3.82539000	-4.68694800
H	3.68099700	3.86225600	-5.79382800
H	2.71927300	0.36430500	-6.12123400
H	2.97843900	1.88575900	-6.98623200
H	1.51779200	1.68063000	-5.98841500
Au	1.14510300	1.24888700	-2.48191400
N	-0.56878600	-3.21819300	-3.50598100
N	-1.73947300	-4.00987300	-1.06610900
N	-2.23036400	-2.65692100	3.22389500
N	-1.35096900	-0.26456600	4.44041800
N	1.15706800	3.70298900	3.68426100
N	2.28011300	4.57270600	1.24066700
C	-0.91971600	1.79314100	-2.08762000
C	-1.27942400	0.69713400	-2.54838000
H	-1.49482400	-0.27047900	-2.95559000
C	-1.49396300	3.04050600	-1.51367800
C	-2.59718100	2.73600500	-0.46539800
H	-1.92174900	3.60140500	-2.34852200
H	-0.72757800	3.66238300	-1.04633600
C	-3.63670100	1.68924500	-0.96247600
O	-3.55049100	1.07636400	-2.01944900
O	-4.62760500	1.58067500	-0.11558100
H	-5.37544100	0.95240100	-0.48543800
C	-1.97233100	2.32728100	0.89412200
H	-1.47375700	3.20891800	1.29782500
H	-2.78579300	2.08270800	1.57596500
C	-3.44550600	4.00067100	-0.24477000
O	-3.67700800	4.51661800	0.82374100
O	-3.93914600	4.44986600	-1.41517800
C	-4.87739700	5.53638100	-1.31204600
H	-5.19335400	5.74772800	-2.33244500
H	-5.72904100	5.23701200	-0.69787800
H	-4.40104700	6.41172400	-0.86387700
C	-0.99956500	1.17932700	0.79797800
C	0.38429300	1.40133500	0.78578900
C	-1.46560900	-0.13408500	0.67265700
C	1.28537000	0.34013500	0.63323200
H	0.76013700	2.41223500	0.89273100
C	-0.57477300	-1.19422500	0.51950700
H	-2.53283000	-0.32866500	0.69368700
C	0.80438200	-0.96402800	0.49306400
H	2.35355100	0.52859500	0.65081400
H	-0.96061400	-2.20141300	0.43994500
H	1.49517600	-1.79370200	0.38896700
S	-7.70758900	-0.20932300	-0.41281300
O	-7.75268400	0.26053100	0.98382000
O	-8.89090500	-0.00537200	-1.24915400
O	-6.40088900	0.11709500	-1.10376700
C	-7.55254400	-2.05482300	-0.26827500
F	-7.49075700	-2.62646000	-1.48139200
F	-6.41455100	-2.38537000	0.39211600
F	-8.58337500	-2.57714400	0.39990900

1a-Int$_6$⊂AuCav

C	3.14622900	4.51251400	2.37707800
C	3.19669600	4.35729300	0.86000900
C	2.44789300	3.31830500	3.02654700
H	2.51211500	5.37786500	2.57334100
C	4.18792600	3.60280300	0.22249900
C	2.22118000	4.94655700	0.04466500
C	3.14102700	2.19238700	3.47941600
C	1.05803300	3.33117800	3.21379700
C	4.22197400	3.39825200	-1.16124500
H	4.95208500	3.13448800	0.83293200
C	2.21644500	4.77630400	-1.33562600
O	1.22495200	5.73039700	0.62722000

C	2.50800800	1.12573100	4.12454000
H	4.21614200	2.15564200	3.34702000
C	0.37987700	2.28740300	3.83280900
O	0.36811200	4.48322600	2.85329300
C	5.30611500	2.56404700	-1.83629000
C	3.20075500	3.98774300	-1.91695500
H	1.43495000	5.21894300	-1.93853600
C	-0.03316300	5.17324800	0.60703400
C	3.26194000	-0.09801400	4.63306900
C	1.12055100	1.20367200	4.28772800
H	-0.69227000	2.32896500	3.97163300
C	-0.46501000	4.45394100	1.76901800
H	5.28349100	2.83355800	-2.89273900
C	4.98765300	1.07329600	-1.77332900
O	3.16723300	3.76748600	-3.30060900
C	3.34827200	-1.14355900	3.52789400
H	2.65014300	-0.53340300	5.42335500
O	0.50161500	0.19144400	5.02147400
C	5.39126500	0.26466700	-0.70711700
C	4.28431000	0.45194400	-2.81386500
C	2.18547100	2.89591200	-3.69908800
C	-2.00663400	4.69448400	-0.41462500
C	4.36063500	-1.10429300	2.56590000
C	2.39866300	-2.16953100	3.41934600
C	-0.36315900	-0.65700700	4.39514000
C	-2.41861900	3.93649600	0.72632900
C	5.15240800	-1.11187600	-0.66290300
H	5.93054900	0.72232900	0.11330100
C	4.01588900	-0.91292500	-2.81299900
O	3.89369000	1.22508400	-3.90470100
C	2.56263600	1.54130000	-3.98776500
C	4.47923500	-2.04701300	1.54315900
H	5.09274200	-0.30801700	2.62212200
C	2.46478700	-3.12302100	2.40496900
O	1.41463000	-2.28170000	4.39728700
C	0.11761500	-1.97331800	4.07094900
C	5.59704100	-1.97954800	0.50861700
C	4.46149600	-1.67603400	-1.74228500
H	3.48018300	-1.36326500	-3.63818300
C	0.00952000	2.36783100	-4.11392000
C	3.50663900	-3.05278900	1.48730500
H	1.72539900	-3.91232900	2.35221200
C	-2.43050000	-1.22493900	3.63103400
H	5.73006700	-2.99185900	0.11983800
O	4.29053000	-3.06398300	-1.80388700
C	0.38525000	1.01824200	-4.39120800
O	3.62585100	-4.06676300	0.52859600
C	-1.95631100	-2.53800400	3.31937900
C	-3.67277500	3.28438300	0.71002300
C	-2.86628600	4.79707600	-1.53174600
C	-0.61276300	0.07350900	-4.71857100
C	-1.35412400	2.73374200	-4.14264700
C	-2.85027500	-3.48633700	2.77444200
C	-3.78158200	-0.89367000	3.38130700
C	6.94434100	-1.55039100	1.10562700
H	7.23571200	-2.23975700	1.90243500
H	6.92428100	-0.54472500	1.53320100
C	6.70907500	2.89288600	-1.30313200
H	7.46404800	2.31097800	-1.83925800
H	6.82136400	2.67907400	-0.23646900
C	4.52704100	4.80144700	2.98347300
H	4.94648600	5.70927000	2.54129400
H	5.24602700	3.99426600	2.81578200
C	4.63029700	0.24296100	5.23975100
H	4.50624000	0.95415900	6.06078300
H	5.32051900	0.69156000	4.51979500
H	5.10495500	-0.66124400	5.63097600
H	4.44146400	4.94726600	4.06380300
H	6.92124100	3.95567100	-1.44666000
H	7.71706400	-1.56384900	0.33238100
C	-4.63263300	-1.83857100	2.84825900
C	-4.16595700	-3.13897600	2.54691400
H	-4.84574200	-3.85771300	2.10345000
H	-5.66078900	-1.57782800	2.63330400
H	-2.47144400	-4.47632900	2.54652900
H	-4.11346100	0.11430500	3.60236500
C	-4.08623700	4.15617200	-1.51629200
H	-2.53440300	5.39343600	-2.37514200
C	-4.49002600	3.39562600	-0.39393300
H	-3.96394600	2.69453800	1.57173500
C	-2.31047500	1.78926700	-4.44492600
H	-1.61474600	3.75484200	-3.89327700
C	-1.93678100	0.45817700	-4.74486500
H	-0.30639300	-0.94570100	-4.92820600
H	-2.70165300	-0.27539400	-4.97568600
H	-3.35902300	2.06276700	-4.42427400
H	-5.44637500	2.89093100	-0.41297700
H	-4.74920400	4.22656300	-2.37284000
P	3.08568300	-3.83327500	-1.00434200
N	3.36255800	-5.38646800	-1.59688600
C	3.28739700	-5.52655600	-3.05711900
C	2.66248300	-6.46425400	-0.88552000
H	3.86554400	-4.73731900	-3.53719300
H	2.25199100	-5.49753800	-3.42909100
H	3.72763800	-6.48941100	-3.33129500
H	2.78472100	-6.34089600	0.19033800
H	3.11577700	-7.41636000	-1.17560500
H	1.58961400	-6.49905100	-1.12796400
Au	0.89453000	-3.11687700	-1.14190700
N	-0.64869600	-2.88251000	3.54535600
N	-1.59053500	-0.29218000	4.17436500
N	-1.61216300	3.84624700	1.82830000
N	-0.78097600	5.30191700	-0.44748200
N	0.95155500	3.29744100	-3.77073200
N	1.69598000	0.63257900	-4.32384800
C	-1.14699800	-2.92231500	-1.23592500
C	-1.90408800	-3.74491700	-0.51341000
H	-1.59348100	-4.57776400	0.09977600
C	-1.87539400	-1.85005200	-2.00774700
C	-3.08640600	-1.32442600	-1.19518100
H	-2.22332800	-2.25712800	-2.96504800
H	-1.22854900	-1.00427900	-2.23633900
C	-3.93728600	-2.50407100	-0.73372300
O	-3.32194800	-3.64246200	-0.44762300
O	-5.15393100	-2.47446200	-0.54300500
H	-6.09602700	-1.42228000	-0.79422900
C	-2.63448100	-0.58229100	0.11493700
H	-3.42514200	0.12224100	0.37549100
H	-2.58976400	-1.32115000	0.91366900
C	-3.93540800	-0.41548200	-2.08453700
O	-4.06269200	0.78138200	-1.95633600
O	-4.52619900	-1.12513500	-3.06688800
C	-5.44992300	-0.39493100	-3.89852200
H	-5.95224100	-1.14960600	-4.50223000
H	-6.16465900	0.14415500	-3.27677600
H	-4.91328800	0.30757900	-4.53933900
C	-1.29237700	0.10946900	0.04383200
C	-1.10560800	1.28076400	-0.70059000
C	-0.19939700	-0.42324700	0.74209800
C	0.14470300	1.89953200	-0.74018100
H	-1.94195000	1.70580000	-1.24284700
C	1.05245800	0.19284800	0.70221100
H	-0.33093900	-1.33486700	1.31365400
C	1.22803000	1.35950000	-0.04322300
H	0.26885300	2.81171100	-1.30918700
H	1.88314900	-0.23265800	1.25485200
H	2.19544100	1.84608600	-0.07367300
S	-6.93368000	0.43465700	0.05128000
O	-6.83875800	-0.71951400	-1.01590800
O	-7.45697500	1.63047400	-0.59014500
O	-5.74414300	0.48088600	0.89883200
C	-8.29042100	-0.23389700	1.13008900
F	-8.49047800	0.60641900	2.14492700
F	-7.92182200	-1.42917100	1.61397200
F	-9.41482900	-0.37162500	0.43261600

1a-TS2$_6$⊂AuCav

C	6.08897400	-0.51224700	-1.09699100
C	5.03653200	-0.40919600	-2.19886400
C	5.44632500	-0.47441900	0.28971400

H	6.69676800	0.39019000	-1.17312600
C	4.34071600	-1.53124400	-2.66372200
C	4.71088500	0.82467300	-2.77974900
C	5.05144500	-1.62393900	0.98079600
C	5.24925600	0.75340100	0.93533500
C	3.34291800	-1.46344900	-3.64212600
H	4.57032900	-2.49679800	-2.22705000
C	3.73442300	0.94028900	-3.76287100
O	5.38537200	1.97922900	-2.37280700
C	4.49745200	-1.58054200	2.26492400
H	5.19736500	-2.58955100	0.51036100
C	4.68442800	0.85220700	2.20077400
O	5.70653000	1.90149200	0.29520300
C	2.57739000	-2.69537400	-4.11362600
C	3.04852600	-0.19837300	-4.16612700
H	3.48723100	1.90817900	-4.17798900
C	4.63621600	2.84718300	-1.61516800
C	4.08609700	-2.83341000	3.03035300
C	4.31761200	-0.32051900	2.84662500
H	4.54160500	1.81594600	2.67000300
C	4.76968200	2.76889400	-0.19044800
H	2.11744000	-2.42345000	-5.06419000
C	1.42795000	-3.02644200	-3.16617200
O	2.03338500	-0.07967400	-5.12035500
C	2.64870100	-3.20308300	2.68715800
H	4.09217800	-2.56607300	4.08732100
O	3.82813500	-0.26748100	4.15379300
C	1.58868100	-3.86716200	-2.06158600
C	0.14726200	-2.50244700	-3.39000200
C	0.88040000	0.50296700	-4.65324700
C	3.08299100	4.49029600	-1.36784600
C	2.34116500	-3.97036700	1.56023200
C	1.57701300	-2.76376900	3.47618300
C	2.57915500	0.23893400	4.36356400
C	3.18007400	4.37303600	0.05268400
C	0.53342400	-4.22454200	-1.21788200
H	2.57026500	-4.28094300	-1.86537200
C	-0.93355200	-2.81689400	-2.57123100
O	-0.05039600	-1.70217300	-4.51065400
C	-0.21526000	-0.35599900	-4.30551500
C	1.03727100	-4.33217000	1.21655900
H	3.15418500	-4.30235400	0.92621000
C	0.25863100	-3.08934700	3.16861600
O	1.84220600	-2.03321200	4.63189300
C	1.52260500	-0.69981900	4.62895100
C	0.72483400	-5.15662600	-0.02660300
C	-0.72477300	-3.67975300	-1.50331000
H	-1.91256500	-2.40567400	-2.77566100
C	-0.34624400	2.31071100	-4.01189300
C	0.01010400	-3.87347600	2.04981000
H	-0.54937600	-2.74795500	3.80316300
C	1.11975700	1.95836800	4.66912700
H	-0.23611300	-5.64554200	0.15015600
O	-1.83957900	-4.09958100	-0.76445300
C	-1.43900500	1.45534500	-3.67061900
O	-1.30620800	-4.27142700	1.79799100
C	0.07143500	1.02611600	4.94358100
C	2.35697900	5.17290900	0.87754600
C	2.17786600	5.42172500	-1.92641300
C	-2.62849400	2.01632800	-3.15562500
C	-0.45842100	3.70242400	-3.80066000
C	-1.22038500	1.50558400	5.25956100
C	0.84805100	3.34492400	4.71772000
C	1.74994600	-6.26971000	-0.28307300
H	1.80004100	-6.93684600	0.58147900
H	2.75917600	-5.89131800	-0.46445000
C	3.50171100	-3.89517600	-4.37000600
H	2.92403200	-4.74439400	-4.74545700
H	4.03199400	-4.22598000	-3.47248600
C	7.02181100	-1.71714000	-1.28783200
H	7.51239900	-1.65981800	-2.26324500
H	6.49758700	-2.67603600	-1.24127900
C	5.06289900	-4.00289300	2.84169200
H	6.06704600	-3.70284300	3.15290800
H	5.12973800	-4.34418000	1.80488800
H	4.75042400	-4.85642900	3.44966700
H	7.79088700	-1.72436200	-0.51056900
H	4.25622800	-3.63028500	-5.11544900
H	1.45531500	-6.85435100	-1.15846100
C	-0.42059000	3.78605200	5.03063500
C	-1.45788300	2.86400500	5.30537300
H	-2.45344800	3.23396900	5.52085000
H	-0.63803400	4.84850100	5.05346600
H	-2.00189300	0.78119100	5.46152900
H	1.66085700	4.03002700	4.50212600
C	1.38638900	6.18874100	-1.09983700
H	2.13287800	5.50176300	-3.00674300
C	1.47392500	6.06169800	0.30694300
H	2.44143300	5.05118000	1.95200900
C	-1.62237600	4.22348700	-3.27657200
H	0.38963600	4.32631400	-4.05512600
C	-2.71343700	3.37938800	-2.96307800
H	-3.46119900	1.36943500	-2.91777800
H	-3.62825900	3.79601800	-2.55676300
H	-1.70035400	5.29216400	-3.10677100
H	0.81882200	6.65556500	0.93331300
H	0.67424100	6.88803700	-1.52465000
P	-2.22778300	-3.48543300	0.69660300
N	-3.66973000	-4.30303400	0.95116600
C	-4.72339500	-4.05268900	-0.04858800
C	-4.18371700	-4.27688700	2.32740900
H	-4.30103400	-4.08291300	-1.05141800
H	-5.21881800	-3.08699600	0.10709200
H	-5.46484000	-4.85200000	0.03547100
H	-3.38863400	-4.52178200	3.03113700
H	-4.96461600	-5.03750200	2.41105000
H	-4.62161000	-3.30205400	2.58969800
Au	-2.42141100	-1.21253700	0.89627800
N	0.30823900	-0.32327000	4.90094100
N	2.38272600	1.52373200	4.37344500
N	4.06117400	3.49651200	0.62024600
N	3.83065400	3.69065800	-2.18949400
N	0.81634700	1.79338400	-4.51426400
N	-1.33580500	0.09995800	-3.83269200
C	-3.30476800	0.73159700	1.17054300
C	-3.64987000	0.97762800	2.45710900
H	-4.03240000	0.23128000	3.14500900
C	-2.89374400	1.92130900	0.33530500
C	-2.24812000	3.03004400	1.19094300
H	-3.78019400	2.30807300	-0.17223500
H	-2.19179400	1.63364700	-0.44824000
C	-3.06671900	3.30161600	2.44957800
O	-3.65038500	2.20399400	3.06174300
O	-3.16557400	4.36582700	2.99951900
H	-4.37729200	0.04109000	0.62730800
C	-0.82049900	2.65948100	1.71759700
H	-0.29958000	3.59924600	1.90992200
H	-0.93789600	2.15775500	2.67784000
C	-2.23724100	4.35008500	0.41410700
O	-1.26031600	5.01789000	0.15736800
O	-3.48729000	4.69167200	0.05763800
C	-3.63633700	5.99229200	-0.53122000
H	-4.69723600	6.09173100	-0.75562300
H	-3.31750600	6.76101900	0.17658600
H	-3.03956300	6.07406000	-1.44121300
C	0.01599700	1.77048900	0.83206700
C	0.53725800	2.22662700	-0.38451100
C	0.31707900	0.46434300	1.24801100
C	1.34394600	1.39371900	-1.16028400
H	0.32093500	3.23694900	-0.71455300
C	1.12224000	-0.37191800	0.46980900
H	-0.06309300	0.10865700	2.20055700
C	1.63748700	0.09385400	-0.74002400
H	1.75244300	1.76638800	-2.09032900
H	1.35384000	-1.37420300	0.81352400
H	2.26949000	-0.54536700	-1.34349800
S	-5.95303900	0.07510400	-1.19536400
O	-5.40721300	1.40738100	-1.48565100
O	-7.37271400	-0.18883800	-1.39023800
O	-5.44457900	-0.47645000	0.15308700

C	-5.06986700	-1.04032700	-2.39624000
F	-5.59321100	-2.27133200	-2.39162400
F	-3.76332600	-1.13553600	-2.06349400
F	-5.15058900	-0.53796000	-3.62892500

(TfO•3a)⊂AuCav

C	-5.76806800	-0.30181300	1.61996800
C	-4.59733500	-0.57716300	2.56153400
C	-5.29754000	0.04548300	0.20777600
H	-6.25666300	0.59529200	2.00217700
C	-3.98403400	-1.83329800	2.62909200
C	-4.08568700	0.42265200	3.40168600
C	-5.11180400	-0.91128900	-0.79601900
C	-5.06236600	1.38254200	-0.13897200
C	-2.91138700	-2.12310900	3.48038100
H	-4.35003900	-2.61993100	1.97900400
C	-3.02835000	0.18101400	4.27170900
O	-4.64590200	1.70418800	3.37084300
C	-4.72433400	-0.57706100	-2.09966800
H	-5.29078200	-1.95389400	-0.55979800
C	-4.67091000	1.76650400	-1.41519800
O	-5.28073100	2.35545600	0.83227100
C	-2.25821200	-3.50240300	3.50701400
C	-2.44742800	-1.08251500	4.29766600
H	-2.64379000	0.97352000	4.89982700
C	-3.86454900	2.63930600	2.73509300
C	-4.52487300	-1.61951500	-3.19554800
C	-4.50725400	0.77842700	-2.37680700
H	-4.49327500	2.80650800	-1.65030200
C	-4.16782600	2.94243500	1.36844600
H	-1.69314100	-3.55513700	4.43809500
C	-1.23897300	-3.63332800	2.37861100
O	-1.37911700	-1.31245100	5.16912200
C	-3.09374800	-2.14471900	-3.16333400
H	-4.63989800	-1.09487600	-4.14452200
O	-4.16940200	1.13875100	-3.68374200
C	-1.56978100	-4.11777500	1.11045600
C	0.09326200	-3.25393700	2.59316600
C	-0.19805600	-0.71886300	4.78666500
C	-2.08530500	4.05575100	2.64278900
C	-2.71085100	-3.18463700	-2.30889700
C	-2.09758300	-1.58499700	-3.97521400
C	-2.90273100	1.59712600	-3.90439500
C	-2.35424800	4.30878200	1.26278700
C	-0.63222800	-4.24140000	0.07912200
H	-2.59059000	-4.42829400	0.92273500
C	1.05981000	-3.33483800	1.59720000
O	0.44086400	-2.83489200	3.87347600
C	0.74411700	-1.51226500	4.05086400
C	-1.40488600	-3.67615000	-2.24210800
H	-3.46281600	-3.62537000	-1.66493600
C	-0.78494900	-2.04198500	-3.94356100
O	-2.43339600	-0.55583100	-4.85855900
C	-1.97908900	0.69701900	-4.54154800
C	-1.00036600	-4.79912400	-1.29248400
C	0.67773300	-3.82959200	0.35644700
H	2.07590300	-3.01395100	1.77536100
C	1.17893300	1.08470900	4.58191400
C	-0.45220000	-3.07765200	-3.07670700
H	-0.03429000	-1.58785400	-4.57737100
C	-1.29810200	3.21338200	-3.86477200
H	-0.08739500	-5.23635200	-1.70388800
O	1.67558500	-3.97447600	-0.61914200
C	2.09381100	0.30348100	3.80996000
O	0.87465200	-3.48814200	-3.03395900
C	-0.38323900	2.32619900	-4.51352800
C	-1.49132000	5.15237200	0.52589600
C	-0.97057000	4.67134800	3.25712900
C	3.25256100	0.90399400	3.26845300
C	1.44456200	2.45664900	4.79443900
C	0.92470200	2.77330900	-4.81166800
C	-0.87872700	4.52040300	-3.52510300
C	-2.04623900	-5.92136300	-1.22620400
H	-2.21398200	-6.33440600	-2.22443000
H	-3.01328100	-5.58287600	-0.84484500
C	-3.28962200	-4.63995600	3.52254900
H	-2.78252100	-5.60671900	3.58531200
H	-3.92324600	-4.65641400	2.63113300
C	-6.80419100	-1.43601400	1.63471000
H	-7.16830100	-1.59621500	2.65300200
H	-6.40088500	-2.38798100	1.27789900
C	-5.57170000	-2.74205700	-3.15537400
H	-6.57681100	-2.31909300	-3.23399900
H	-5.53404500	-3.33091100	-2.23474600
H	-5.41677400	-3.42938400	-3.99146600
H	-7.65403400	-1.17774800	0.99696500
H	-3.94785400	-4.53538500	4.38918400
H	-1.69571800	-6.72288000	-0.57069200
C	0.40317200	4.93048000	-3.82713500
C	1.30652100	4.05664300	-4.47734200
H	2.31645300	4.39253000	-4.67981200
H	0.73542800	5.92714900	-3.55688800
H	1.60400000	2.08385300	-5.30058800
H	-1.59337100	5.17231000	-3.03443000
C	-0.14553300	5.48783400	2.51713300
H	-0.78788500	4.46327400	4.30457500
C	-0.40478100	5.72713600	1.14588000
H	-1.71080800	5.31440800	-0.52368700
C	2.57295900	3.02775200	4.24286700
H	0.73873700	3.02722500	5.38709800
C	3.47663400	2.24794000	3.48255200
H	3.94289000	0.30030100	2.68926600
H	4.36294800	2.70557400	3.05714700
H	2.76800700	4.08530700	4.39073000
H	0.27861900	6.35162900	0.58243600
H	0.73212500	5.93255600	2.97152300
P	1.84298900	-2.87865400	-1.80964900
N	3.35377500	-3.16864300	-2.34608700
C	4.17983000	-4.32120800	-1.97879000
C	3.91672300	-2.28712600	-3.36585500
H	3.72556400	-4.85733700	-1.14970700
H	5.15584800	-3.95076200	-1.66041600
H	4.28380000	-4.99547000	-2.83778800
H	3.24007300	-1.44941100	-3.55799000
H	4.05666500	-2.83107600	-4.30776000
H	4.87172000	-1.89613000	-3.00512200
Au	0.98232600	-0.79931500	-1.27804900
N	-0.75939100	1.04618600	-4.83073400
N	-2.57118200	2.80857800	-3.56818400
N	-3.43178700	3.73657000	0.64875600
N	-2.86507400	3.18959400	3.35905000
N	0.01795800	0.53396100	5.05991600
N	1.84615700	-1.02043800	3.57542800
C	3.68733700	0.90056600	-1.70221700
C	3.78978900	1.52137000	-2.87323100
H	3.92168700	1.04729200	-3.83676300
C	3.55517600	1.67557100	-0.43139600
C	2.90375600	3.04404800	-0.70709500
H	4.54271400	1.79783600	0.02317400
H	2.96754100	1.11595300	0.29840300
C	3.48748800	3.71614900	-1.96191200
O	3.80412800	2.90695700	-3.01034100
O	3.57191500	4.91228000	-2.10078400
H	3.78492000	-0.17282500	-1.65288800
C	1.38041500	2.95977300	-1.03809900
H	0.96771900	3.96171700	-0.89382600
H	1.28461200	2.73069600	-2.09959400
C	3.17708100	4.01621000	0.44148700
O	2.33765200	4.62517200	1.06941000
O	4.49568500	4.12987600	0.66064800
C	4.88644500	5.14932400	1.59488900
H	5.97086400	5.08103700	1.66489700
H	4.58382500	6.13021900	1.22118000
H	4.42430600	4.97751800	2.56876900
C	0.51135000	1.98274100	-0.28797700
C	0.36484700	1.99010800	1.10034800
C	-0.31562600	1.10651400	-1.04978700
C	-0.59397800	1.17707800	1.71320900
H	0.95730000	2.66885700	1.70137700
C	-1.29646800	0.29701800	-0.41589000

H	-0.36375000	1.25071600	-2.12562100
C	-1.41390000	0.32337700	0.97361700
H	-0.72500100	1.23597000	2.78498100
H	-1.97178500	-0.30283700	-1.01706900
H	-2.15270500	-0.29423600	1.46674500
S	4.80591800	-1.45833800	0.55388600
O	3.31890600	-1.40584100	0.42773800
O	5.51452100	-1.83246000	-0.68853100
O	5.39207000	-0.32597100	1.30260300
C	5.04018600	-2.91435900	1.67940400
F	4.47062700	-2.67933200	2.87057800
F	6.33641600	-3.18329300	1.86612000
F	4.45248300	-4.01071400	1.15269700

1a⊂AuCav

C	-2.73020500	0.55734600	4.50758500
C	-1.92134300	-0.65784400	4.05790400
C	-2.75957400	1.61455000	3.40898900
H	-2.18029200	0.99774900	5.33988000
C	-2.50568300	-1.74099900	3.39204100
C	-0.54589100	-0.73435700	4.32298200
C	-3.71178500	1.59950800	2.38075400
C	-1.80608600	2.64191600	3.38363200
C	-1.78845200	-2.88474600	3.02902800
H	-3.56650100	-1.70196600	3.17370300
C	0.21205700	-1.85098100	3.98310400
O	0.04483900	0.29466600	5.04624200
C	-3.74244800	2.55497900	1.35684300
H	-4.46756500	0.82235500	2.38881600
C	-1.78605100	3.60459600	2.38094300
O	-0.89231300	2.73227900	4.42951400
C	-2.42205500	-4.06956600	2.30150800
C	-0.42652900	-2.91097100	3.34999100
H	1.26154900	-1.90579200	4.24111400
C	0.90884400	1.15506300	4.42735300
C	-4.79782400	2.54097100	0.25075400
C	-2.75211600	3.54923400	1.38346900
H	-1.03569200	4.38404900	2.37918500
C	0.41927000	2.47142700	4.13223900
H	-1.81158300	-4.94233700	2.53490100
C	-2.30488000	-3.84534000	0.79715700
O	0.26548700	-4.09419800	3.09521600
C	-4.33342600	1.67852900	-0.92076400
H	-4.85503000	3.56238800	-0.12711900
O	-2.78150400	4.53905000	0.40724300
C	-3.26323700	-3.10783100	0.09344000
C	-1.20925600	-4.33614900	0.06942800
C	1.26821500	-4.10171600	2.17960300
C	2.96456100	1.75757700	3.65785700
C	-4.52985100	0.29189200	-0.95272300
C	-3.65454300	2.25962500	-2.00500300
C	-1.78300100	4.50453500	-0.53084900
C	2.47649800	3.06944300	3.37472700
C	-3.16611600	-2.81399900	-1.27117700
H	-4.12683600	-2.74307900	0.63572500
C	-1.05231900	-4.04775200	-1.28306900
O	-0.28512600	-5.17149700	0.69103900
C	0.98801400	-4.71112900	0.90622200
C	-4.05554800	-0.52533300	-1.98838100
H	-5.06041600	-0.17293200	-0.12976400
C	-3.17464800	1.49183500	-3.05946100
O	-3.43913500	3.62816700	-2.02567000
C	-2.13423600	4.03325100	-1.83510700
C	-4.25738200	-2.04283600	-2.02067100
C	-2.01496700	-3.27839300	-1.92265300
H	-0.19310000	-4.42082100	-1.82454400
C	3.42776900	-3.76244600	1.54117600
C	-3.36835900	0.11863300	-3.02595800
H	-2.62337700	1.96019300	-3.86456800
C	0.35418700	4.84603100	-1.23080700
H	-4.16897300	-2.33694200	-3.06968900
O	-1.75066100	-2.91243700	-3.26031200
C	3.16849000	-4.42671500	0.30383500
O	-2.71560000	-0.63661600	-4.01738400
C	-0.00176100	4.39740900	-2.54132000
C	3.34865900	4.03248300	2.81945600
C	4.31986800	1.44791500	3.39893600
C	4.22443400	-4.60550500	-0.61931100
C	4.72286900	-3.26191300	1.81071400
C	0.97595800	4.38678900	-3.56230600
C	1.68235600	5.25138600	-0.96837000
C	-5.66859300	-2.44518900	-1.56278800
H	-6.41678700	-1.94398900	-2.18159300
H	-5.86939100	-2.17792000	-0.52231700
C	-3.85545400	-4.36936900	2.76012000
H	-4.24202600	-5.24791500	2.23702400
H	-4.54956600	-3.54431400	2.57739800
C	-4.12982400	0.18843000	5.01963000
H	-4.05223600	-0.53252400	5.83749900
H	-4.76830600	-0.25439300	4.24980100
C	-6.19315800	2.15076100	0.75910400
H	-6.50252500	2.82968000	1.55754400
H	-6.23692000	1.13329800	1.15818000
H	-6.92109400	2.21757900	-0.05365900
H	-4.63739800	1.08185700	5.39207400
H	-3.86635700	-4.57355300	3.83358500
H	-5.80215600	-3.52539800	-1.65947600
C	2.61470700	5.23248900	-1.98179300
C	2.25980500	4.80407500	-3.28349000
H	3.00999600	4.80575900	-4.06794000
H	3.63422100	5.54891500	-1.78546800
H	0.68026300	4.06150700	-4.55427600
H	1.92852500	5.56602800	0.03905600
C	5.15227200	2.40762200	2.86008800
H	4.67466000	0.45267400	3.64585300
C	4.66274200	3.70294700	2.56442100
H	2.94979000	5.01854700	2.60921900
C	5.72982700	-3.43055400	0.88314000
H	4.89037000	-2.76373500	2.75947100
C	5.48063700	-4.11215800	-0.33249900
H	4.02270900	-5.14616200	-1.53738000
H	6.29234200	-4.25652100	-1.03820000
H	6.72512800	-3.05028900	1.08761600
H	5.33559800	4.44271900	2.14272700
H	6.19282600	2.17233300	2.66124700
P	-1.33013200	-1.34633500	-3.47690300
N	-0.27755600	-1.42726900	-4.74059000
C	-0.42701700	-2.41998900	-5.81647300
C	0.32688500	-0.14914600	-5.15058700
H	-0.82112900	-3.35088400	-5.41069300
H	0.56035400	-2.60991400	-6.24610100
H	-1.09437500	-2.05516800	-6.60564200
H	0.41495700	0.51748400	-4.29043100
H	-0.27189500	0.34106900	-5.92671800
H	1.33351400	-0.34075600	-5.52865200
Au	-0.84873700	-0.43218300	-1.38029500
N	-1.27582400	3.97213300	-2.80967200
N	-0.57738200	4.88632700	-0.23210300
N	1.17315300	3.39704900	3.62075900
N	2.13635600	0.79874300	4.17998000
N	2.43843300	-3.61576000	2.47478600
N	1.90716700	-4.87176400	-0.00020600
C	0.13369800	-0.19868300	0.74131800
C	-1.02206500	0.22660800	0.73854900
H	-1.96803300	0.65031200	1.02801700
C	1.54125300	-0.56958700	0.86052100
C	2.42122300	0.05731500	-0.25343300
H	1.65411600	-1.65239300	0.85760300
H	1.87755300	-0.20145900	1.83447100
C	2.04564000	1.55761300	-0.36614800
O	1.82830400	2.19200700	0.64246500
O	1.98358700	2.10291800	-1.57323300
H	2.21027300	1.41841600	-2.25082500
C	3.91695700	-0.03998800	0.20952100
H	3.97516000	0.51984300	1.14453700
H	4.12457000	-1.08977700	0.42836100
C	2.31139500	-0.70153600	-1.57408800
O	2.36499300	-0.17512600	-2.68102000
O	2.23781900	-2.01724000	-1.41441700
C	2.25141800	-2.81053100	-2.62800400

H	1.47362500	-2.46285400	-3.30760300
H	3.22875800	-2.72030700	-3.10473600
H	2.06945900	-3.82807200	-2.29389400
C	4.90135800	0.50017600	-0.79254800
C	5.56052600	-0.36369200	-1.67499000
C	5.14201500	1.87824600	-0.87698800
C	6.44143700	0.13897800	-2.63234900
H	5.38707100	-1.43455800	-1.60010800
C	6.01955900	2.38092800	-1.83632700
H	4.64246900	2.55358500	-0.18864200
C	6.66949600	1.51343400	-2.71674300
H	6.95275500	-0.53962400	-3.30824800
H	6.20137200	3.44982000	-1.89334800
H	7.35652400	1.90605100	-3.45973400

1a⊂AuCav-1

C	-0.87665100	-0.99698800	4.84883100
C	0.41580900	-1.22498000	4.07251000
C	-2.02803900	-0.58092000	3.93969200
H	-0.68777600	-0.14832100	5.50636000
C	0.67874200	-2.46184400	3.47810400
C	1.41710400	-0.24733400	3.97620700
C	-2.90277300	-1.48544700	3.32885400
C	-2.26853600	0.78140000	3.72673600
C	1.89534500	-2.79111000	2.87853600
H	-0.09244000	-3.22124100	3.52104500
C	2.65155800	-0.52627500	3.38973800
O	1.22776800	1.00040900	4.58692700
C	-4.00651200	-1.07198000	2.57404600
H	-2.73165700	-2.54730500	3.46195400
C	-3.35846800	1.24848100	3.00312200
O	-1.40727500	1.66918500	4.36602300
C	2.11641000	-4.17103500	2.26147900
C	2.88606600	-1.79824700	2.86593000
H	3.43907200	0.21784700	3.39244100
C	0.88433500	2.07676900	3.81713000
C	-4.91234900	-2.05995600	1.83941700
C	-4.22269900	0.30778700	2.45026400
H	-3.54596300	2.30820700	2.89217900
C	-0.51516600	2.38209700	3.63326300
H	3.19445800	-4.32342000	2.21041700
C	1.60222600	-4.13260200	0.82608000
O	4.17318400	-2.12859200	2.43150400
C	-4.28908000	-2.29084200	0.46622100
H	-5.86638100	-1.55791100	1.67773700
O	-5.38497400	0.72715300	1.81156300
C	0.27836800	-4.40701600	0.47723500
C	2.46498200	-3.72016400	-0.19725700
C	4.64559900	-1.60555700	1.26118200
C	1.41157500	3.91831900	2.57418700
C	-3.30737400	-3.26348000	0.25511100
C	-4.61941900	-1.46499500	-0.61849200
C	-5.28931100	1.45123200	0.65863200
C	0.03169400	4.13180200	2.28279700
C	-0.20761000	-4.23824200	-0.82499800
H	-0.40440800	-4.75034600	1.24563600
C	2.02913800	-3.51495500	-1.49999000
O	3.79747000	-3.53987900	0.14365000
C	4.37716800	-2.31295500	0.03423200
C	-2.61486300	-3.40409100	-0.95223400
H	-3.07799000	-3.94818600	1.06255500
C	-3.91411000	-1.52304600	-1.81577500
O	-5.70683800	-0.60514100	-0.50292000
C	-5.48799200	0.74447600	-0.57650800
C	-1.64774000	-4.55813500	-1.21176100
C	0.68923200	-3.75490800	-1.78490100
H	2.70756900	-3.14628200	-2.25866700
C	5.84329800	-0.06658400	0.08093300
C	-2.89926800	-2.46140700	-1.94760200
H	-4.16684600	-0.86029500	-2.63277200
C	-5.07057300	3.40135400	-0.49444800
H	-1.64472000	-4.70930400	-2.29421400
O	0.22701900	-3.41352100	-3.06498100
C	5.46899700	-0.70146900	-1.14273500
O	-2.18299800	-2.45777700	-3.15489100
C	-5.30782800	2.70525800	-1.72045000
C	-0.35034700	5.20446200	1.44346700
C	2.36899300	4.83853300	2.09237500
C	5.89478000	-0.15421900	-2.37484500
C	6.70403400	1.05352600	0.04428600
C	-5.29705900	3.41935100	-2.93969400
C	-4.81500100	4.79121600	-0.51913400
C	-2.13387900	-5.87429800	-0.58283900
H	-3.14361900	-6.10714500	-0.93000700
H	-2.15856600	-5.84047600	0.50929700
C	1.52346200	-5.31434700	3.09506600
H	1.76218200	-6.27512600	2.63155500
H	0.43581000	-5.25891500	3.19394600
C	-1.23817900	-2.19411900	5.74270000
H	-0.41034400	-2.41926500	6.41981100
H	-1.45757600	-3.10355800	5.17682300
C	-5.18525400	-3.34849900	2.62389600
H	-5.65922000	-3.11044500	3.57948500
H	-4.28023000	-3.92276600	2.84139100
H	-5.85796900	-3.99708000	2.05679300
H	-2.12185300	-1.95723400	6.34088700
H	1.94580800	-5.30223600	4.10296700
H	-1.46801600	-6.69138800	-0.87137900
C	-4.80133700	5.46233400	-1.72347400
C	-5.04600300	4.77494600	-2.93676800
H	-5.03704400	5.32510400	-3.87203100
H	-4.60907900	6.53014900	-1.74594800
H	-5.48718600	2.87254600	-3.85677100
H	-4.64272400	5.29616400	0.42527200
C	1.97214000	5.89647900	1.30041500
H	3.40025300	4.69247100	2.38442700
C	0.60950800	6.06822800	0.95723300
H	-1.40429300	5.33104800	1.22098300
C	7.13458100	1.54864700	-1.16935800
H	7.00985700	1.49062800	0.98825800
C	6.70977100	0.95778500	-2.38338400
H	5.57393500	-0.63955900	-3.28928900
H	7.04518100	1.37595500	-3.32650900
H	7.80835600	2.39906600	-1.20134900
H	0.31869700	6.89952700	0.32356500
H	2.70580300	6.61109700	0.94071800
P	-0.61722000	-1.98819300	-3.15141100
N	-0.45074600	-1.43935400	-4.69463000
C	0.85874300	-0.88429900	-5.06208700
C	-1.21717400	-1.99972300	-5.81540300
H	1.38239400	-0.50602300	-4.18247700
H	0.70846100	-0.05578100	-5.76076800
H	1.48737600	-1.64422700	-5.54195600
H	-2.20014100	-2.32093900	-5.47552100
H	-0.69369700	-2.84906400	-6.27079900
H	-1.34292400	-1.21755500	-6.56969800
Au	0.02433100	-0.73296200	-1.30693000
N	-5.50045100	1.34876300	-1.72825200
N	-5.07198300	2.73283800	0.69983200
N	-0.92473400	3.33234500	2.84599700
N	1.81490400	2.83127900	3.30832800
N	5.37067200	-0.52557400	1.28354300
N	4.73711300	-1.85717200	-1.12736500
C	1.12603000	0.49979200	0.42507700
C	0.12273500	-0.07658700	0.84186500
H	-0.68873400	-0.44445800	1.43904700
C	2.41030300	1.18433600	0.24903800
C	2.62991800	2.10661700	-0.97291800
H	3.19194900	0.42402200	0.25401100
H	2.54923600	1.76999300	1.16263600
C	2.82072900	1.25783500	-2.24964700
O	2.45899000	0.09769900	-2.30226000
O	3.33338600	1.86907900	-3.30998700
H	3.75121700	2.71865900	-3.01344500
C	1.46801100	3.15293300	-1.21711700
H	1.29304900	3.65489000	-0.26463900
H	1.86460000	3.90455400	-1.90432900
C	3.87183500	2.99144900	-0.77469200
O	4.37281900	3.61221700	-1.70478700
O	4.29600000	3.08541800	0.47271300

C	5.37067200	4.02847300	0.70798600
H	6.20169300	3.81807500	0.03862800
H	5.00569000	5.04217500	0.53578000
H	5.65395200	3.88310100	1.74828900
C	0.16958800	2.61083800	-1.76280000
C	-0.94116100	2.45795300	-0.92574900
C	0.04217100	2.27785900	-3.11997200
C	-2.13317600	1.93352800	-1.41792700
H	-0.86834900	2.72421800	0.12166800
C	-1.15919500	1.77376500	-3.61814400
H	0.88391900	2.41691400	-3.79078200
C	-2.24865700	1.59168200	-2.76587300
H	-2.96353800	1.78898400	-0.74134200
H	-1.24240800	1.51976100	-4.66940700

1a⊂AuCav-2

C	2.20959200	-3.74203300	3.00485400
C	2.96568800	-2.49360000	2.55936000
C	0.81142400	-3.85399900	2.40116000
H	2.06032000	-3.62904300	4.07913900
C	3.72308000	-2.45915800	1.38385900
C	2.98416200	-1.34357400	3.36246500
C	0.56661600	-4.48180300	1.17669000
C	-0.30522200	-3.38596600	3.10823600
C	4.50137100	-1.35844800	1.00457100
H	3.72425200	-3.33603300	0.74716300
C	3.74797400	-0.22653400	3.03628800
O	2.24399500	-1.34179300	4.54633100
C	-0.72133400	-4.68674700	0.66964300
H	1.41216900	-4.83537800	0.59804300
C	-1.60656500	-3.60779800	2.67261900
O	-0.08442600	-2.73064800	4.32121000
C	5.32155800	-1.35424000	-0.28439300
C	4.49650900	-0.24726600	1.86120200
H	3.74736200	0.64716500	3.67431900
C	1.07094300	-0.63484700	4.50930700
C	-0.95400200	-5.28156800	-0.71792700
C	-1.79848600	-4.25745300	1.45542000
H	-2.45201200	-3.27536700	3.26042500
C	-0.15533300	-1.36704300	4.32474500
H	6.08137300	-0.58183100	-0.16389300
C	4.44614800	-0.92322700	-1.45760300
O	5.27440800	0.86436200	1.52205200
C	-0.83497800	-4.13623900	-1.71786500
H	-1.98787500	-5.62551400	-0.74807100
O	-3.09472400	-4.51764100	1.00545600
C	3.63088500	-1.82123200	-2.15482600
C	4.39601500	0.42457500	-1.84385900
C	4.57639600	1.96558900	1.10186400
C	-0.10299000	1.31738800	4.54723300
C	0.38206800	-3.78229500	-2.30634100
C	-1.95462200	-3.34766900	-2.02421000
C	-3.86867500	-3.43750600	0.67694900
C	-1.31030700	0.59991000	4.28698500
C	2.75963600	-1.42378200	-3.17689500
H	3.65274800	-2.86736400	-1.87256300
C	3.57244400	0.86258300	-2.87494800
O	5.16542900	1.34791300	-1.14338800
C	4.48463800	2.20292500	-0.31347700
C	0.53641000	-2.67374100	-3.14733500
H	1.24364500	-4.41174100	-2.12233600
C	-1.83999700	-2.19625400	-2.79793600
O	-3.20112100	-3.78590400	-1.59950000
C	-3.92294100	-3.04186100	-0.70341000
C	1.83793100	-2.39928100	-3.90397400
C	2.75135600	-0.06471700	-3.50860700
H	3.53110300	1.91270800	-3.13191000
C	3.27596400	3.79639400	1.48635500
C	-0.59555100	-1.86818400	-3.32480900
H	-2.71350100	-1.59546400	-3.01566000
C	-5.28338100	-1.73137700	1.21190500
H	1.54538200	-1.90652300	-4.83485500
O	1.77516900	0.43465600	-4.39034900
C	3.14975600	4.00755400	0.07804500
O	-0.54211400	-0.71788000	-4.13448600
C	-5.34968400	-1.34659400	-0.16417500
C	-2.52508000	1.31005300	4.14381800
C	-0.14525300	2.72353700	4.69809400
C	2.35372000	5.07677800	-0.39612600
C	2.61349500	4.66584900	2.38460000
C	-6.12724200	-0.22686500	-0.53466800
C	-5.98156500	-0.97886200	2.18343400
C	2.57348700	-3.68989700	-4.29992200
H	1.90534000	-4.35007000	-4.85832800
H	2.94915500	-4.24716800	-3.43808800
C	6.04465100	-2.68541800	-0.53708000
H	6.66061500	-2.61196900	-1.43710400
H	5.36026500	-3.52744200	-0.67291600
C	3.03538900	-5.02149600	2.78997300
H	3.99564700	-4.93778000	3.30529200
H	3.24332100	-5.22596100	1.73593300
C	-0.04961300	-6.47980100	-1.03039400
H	-0.22429400	-7.27543400	-0.30205600
H	1.01638800	-6.23737900	-0.99845300
H	-0.27109100	-6.86989100	-2.02738000
H	2.49886600	-5.88403700	3.19317700
H	6.69462900	-2.92325800	0.30856500
H	3.43118800	-3.44645500	-4.93162200
C	-6.72220300	0.11706300	1.79501500
C	-6.79672100	0.49144500	0.43223400
H	-7.38860400	1.35539700	0.14795800
H	-7.26005400	0.69733000	2.53776900
H	-6.16981600	0.04966300	-1.58015200
H	-5.91577300	-1.29411700	3.21912500
C	-1.34573500	3.39305000	4.56706900
H	0.78323000	3.24317400	4.90803400
C	-2.53730000	2.68365700	4.28058000
H	-3.42914300	0.75072700	3.92925900
C	1.84393800	5.70139900	1.89743000
H	2.72976300	4.48488600	3.44753200
C	1.71212500	5.90626400	0.50204000
H	2.28058900	5.22712600	-1.46826200
H	1.09635400	6.72385100	0.14305200
H	1.31902400	6.35909400	2.58106700
H	-3.46498100	3.23228900	4.16228100
H	-1.38071700	4.47258000	4.66119800
P	0.29210300	0.59706900	-3.67239600
N	-0.51486200	1.79189100	-4.44723600
C	-0.13727900	3.17991400	-4.17470400
C	-1.50726900	1.60524900	-5.51085700
H	0.56872700	3.22991800	-3.34112100
H	-1.03184800	3.75156400	-3.90690600
H	0.33737300	3.63229600	-5.05186100
H	-1.73267000	0.54830000	-5.63367700
H	-1.12592500	2.00388200	-6.45692600
H	-2.42554000	2.13161700	-5.23301800
Au	0.73208500	0.80169100	-1.39585300
N	-4.63707600	-2.03343900	-1.11373000
N	-4.52240400	-2.79879500	1.60223400
N	-1.30045500	-0.76542000	4.18330200
N	1.09486200	0.65989400	4.61926500
N	3.99822000	2.74120300	1.96989700
N	3.78209900	3.17862000	-0.80876600
C	0.74430800	1.73008500	0.83377400
C	1.52579300	0.79130400	0.67366600
H	2.25896400	0.02849600	0.85221900
C	-0.11639900	2.85083500	1.20419900
C	-1.57576200	2.88280300	0.67574600
H	0.35440300	3.78526100	0.89808600
H	-0.12711800	2.86525800	2.29825600
C	-2.26857000	4.09838500	1.34957900
O	-1.61828100	5.06749900	1.67261300
O	-3.59341900	4.05531300	1.45154500
H	-3.90408400	3.13957600	1.23644400
C	-1.54958800	3.21562600	-0.86494900
H	-1.31297500	4.28021300	-0.94424100
H	-0.69593700	2.67463600	-1.28357200
C	-2.33223700	1.57949700	0.90285700
O	-3.54297100	1.49564800	1.04563400
O	-1.52223300	0.53589800	0.85639200

C	-2.08793300	-0.78398200	0.94668500
H	-1.24784300	-1.46118700	0.81014900
H	-2.53639100	-0.93143700	1.92622200
H	-2.82189700	-0.90222200	0.15611300
C	-2.77503600	2.86987400	-1.66804500
C	-3.73558300	3.84048200	-1.97214600
C	-2.95781500	1.56307600	-2.14419100
C	-4.84863400	3.51616900	-2.75004300
H	-3.61083000	4.85306300	-1.59942400
C	-4.06582200	1.23674800	-2.92158700
H	-2.22826400	0.79569700	-1.90175000
C	-5.01275500	2.21668500	-3.23140800
H	-5.58391600	4.27982300	-2.98251100
H	-4.20047500	0.21856800	-3.27236400
H	-5.87532500	1.96691400	-3.84136800

1a⊂AuCav-3

C	0.07619900	-2.19760200	4.60468500
C	0.60885600	-0.81315900	4.24625500
C	-0.38754400	-2.90440300	3.33433400
H	-0.81351700	-2.04352900	5.21573500
C	1.96071400	-0.52219900	4.05338800
C	-0.30370400	0.23902900	4.10099900
C	0.53603200	-3.57923700	2.52971300
C	-1.72790700	-2.92639300	2.91792200
C	2.41411500	0.76622000	3.74828100
H	2.68862500	-1.31490200	4.17778300
C	0.08642400	1.53418000	3.78355800
O	-1.60789900	-0.06560800	4.44544500
C	0.19151300	-4.29976800	1.38273300
H	1.57436600	-3.57788900	2.83874400
C	-2.11095600	-3.58085400	1.74632500
O	-2.72827300	-2.40823200	3.74284500
C	3.89564600	1.09322100	3.56069700
C	1.44746800	1.77306400	3.61086900
H	-0.63447500	2.33851200	3.72020000
C	-2.69077700	0.00534500	3.63763300
C	1.24662300	-5.10850300	0.63067600
C	-1.15748700	-4.26581100	0.99315600
H	-3.15494100	-3.61959800	1.45885000
C	-3.35752800	-1.24699300	3.38285000
H	4.02105000	2.14176400	3.83239100
C	4.23651800	0.98073000	2.07877700
O	1.90605900	3.07207000	3.41750300
C	1.98873200	-4.20613800	-0.34759000
H	0.70290700	-5.83891900	0.03110500
O	-1.58470900	-5.01952500	-0.10243600
C	4.49437800	-0.25725500	1.47652100
C	4.22098900	2.11013100	1.24937900
C	1.68954100	3.73344300	2.24901500
C	-4.43428400	1.12605700	2.70680600
C	3.12986500	-3.46936100	-0.01609900
C	1.47797700	-4.05791100	-1.64302200
C	-1.87377300	-4.34279200	-1.26110100
C	-5.16065900	-0.09845700	2.60221500
C	4.67588400	-0.41624200	0.09839700
H	4.53356300	-1.13770200	2.10724300
C	4.36821500	2.00172400	-0.12882200
O	4.02072800	3.37489100	1.80152600
C	2.84309600	3.97301300	1.42562900
C	3.74188500	-2.57785600	-0.90978600
H	3.55302700	-3.58154400	0.97527400
C	2.05119400	-3.20033500	-2.56882200
O	0.35828100	-4.80228100	-1.97467300
C	-0.81508000	-4.14363000	-2.21077500
C	4.99017100	-1.76845800	-0.54853200
C	4.56298500	0.74557500	-0.67735200
H	4.28337000	2.87691100	-0.76040600
C	0.41515200	4.92519900	0.78765300
C	3.15931100	-2.45993900	-2.17933700
H	1.61780600	-3.08037900	-3.55240900
C	-3.29777300	-3.23196600	-2.65049800
H	5.48312800	-1.53954900	-1.49705800
O	4.54313000	0.66044600	-2.08301600
C	1.57186800	5.20412700	-0.00082400
O	3.60493100	-1.48752800	-3.08554200
C	-2.20871400	-2.90623900	-3.51564200
C	-6.50811900	-0.07391000	2.18068600
C	-5.05733100	2.33777600	2.33658900
C	1.44325300	5.97060900	-1.18043300
C	-0.84430600	5.41564000	0.37375000
C	-2.43433800	-2.09565700	-4.65187800
C	-4.60064600	-2.80167300	-2.98686800
C	5.98798400	-2.58510500	0.28835600
H	6.24987000	-3.50771200	-0.23556300
H	5.59232900	-2.86205900	1.26893400
C	4.82530900	0.26446100	4.45697300
H	5.85887200	0.59529400	4.32699000
H	4.79469800	-0.80641100	4.23690800
C	1.06022300	-3.03839300	5.42926000
H	1.31992900	-2.51087500	6.35081100
H	1.99230100	-3.25697600	4.90075800
C	2.18313800	-5.88241900	1.56967100
H	1.60183800	-6.54003400	2.22068600
H	2.78423600	-5.23231500	2.21146400
H	2.87259600	-6.49626300	0.98411800
H	0.60258000	-3.99449500	5.69521400
H	4.55042300	0.39386800	5.50693800
H	6.89987000	-2.00603400	0.45305900
C	-4.79944600	-2.02815800	-4.10924000
C	-3.71125000	-1.66034800	-4.93593000
H	-3.89163100	-1.04639800	-5.81257100
H	-5.79803700	-1.68694000	-4.35891000
H	-1.59120700	-1.86437000	-5.29433500
H	-5.41641500	-3.07970400	-2.33141400
C	-6.37002900	2.33312000	1.91247300
H	-4.48545500	3.25251300	2.44662700
C	-7.10166000	1.12470500	1.84547900
H	-7.04566100	-1.01473400	2.13219300
C	-0.94364100	6.15622200	-0.78730200
H	-1.70569100	5.19981900	0.99579600
C	0.20346200	6.43367900	-1.56748100
H	2.33607100	6.16318200	-1.76450500
H	0.10216000	7.01539000	-2.47748600
H	-1.90758600	6.53883500	-1.10655300
H	-8.13954600	1.14580900	1.52955900
H	-6.86059900	3.26605900	1.65280800
P	3.15076500	0.05236800	-2.68636800
N	2.93715200	0.91775900	-4.06167900
C	4.04716200	1.43112300	-4.87286200
C	1.63203000	0.86617900	-4.72482800
H	4.94350100	1.52284300	-4.26090400
H	3.77611100	2.42252700	-5.24718500
H	4.25201000	0.76960800	-5.72236200
H	1.66326600	0.22206500	-5.61150900
H	1.33387400	1.87758400	-5.01407300
H	0.87727000	0.47357200	-4.03847500
Au	1.45333800	-0.03143500	-1.07995000
N	-0.95917100	-3.40721300	-3.27247800
N	-3.08227100	-3.91907900	-1.48503000
N	-4.55981500	-1.29559400	2.89457200
N	-3.17553100	1.14822400	3.25007000
N	0.50736600	4.17372300	1.93028300
N	2.79188800	4.69017800	0.34544100
C	-0.42114100	0.50047900	0.29748100
C	0.10240800	-0.56985400	0.61267700
H	0.33629400	-1.49894800	1.10154000
C	-1.19982100	1.72978900	0.11692300
C	-1.44074300	2.17926800	-1.34427100
H	-0.72329500	2.52959600	0.68782900
H	-2.16745100	1.53717100	0.59031100
C	-0.14567500	2.76891900	-1.95925500
O	0.94253000	2.61504800	-1.43947800
O	-0.27435900	3.38389100	-3.12982500
H	-1.23012200	3.61680100	-3.25561700
C	-1.92261600	0.98441800	-2.25796800
H	-1.17873900	0.18557200	-2.18750300
H	-1.92673800	1.33334800	-3.29235300
C	-2.54941000	3.23005200	-1.45339700
O	-2.74579600	3.83862200	-2.50001900

O	-3.29710700	3.36893100	-0.37436300
C	-4.44242800	4.24747000	-0.50677300
H	-4.75926000	4.46629400	0.50848000
H	-4.16052100	5.15339900	-1.04191300
H	-5.22641300	3.71615000	-1.04863100
C	-3.29292800	0.48414100	-1.87135100
C	-4.43267500	1.03962400	-2.46916400
C	-3.45651900	-0.49217900	-0.88256700
C	-5.70844200	0.64974900	-2.06404600
H	-4.31740000	1.78373400	-3.25132600
C	-4.73116100	-0.89241300	-0.48682400
H	-2.58703300	-0.96328000	-0.43547900
C	-5.85904800	-0.31353000	-1.06716100
H	-6.58207400	1.08691200	-2.53811700
H	-4.83762100	-1.66439200	0.26583200
H	-6.84975400	-0.62317500	-0.75387300

1a⊂AuCav-4

C	-3.68059500	-2.56887900	2.96112300
C	-2.17355100	-2.80511200	2.99252100
C	-4.10425300	-1.57165000	1.88665600
H	-3.92830400	-2.10323400	3.91542400
C	-1.54294300	-3.78875200	2.22339000
C	-1.36629000	-2.06134900	3.86527300
C	-4.33530500	-1.96355500	0.56524500
C	-4.33776100	-0.22491900	2.20005000
C	-0.17411200	-4.07025900	2.32138400
H	-2.14537600	-4.37599100	1.54029300
C	-0.00635200	-2.31170900	4.01368200
O	-1.97845500	-1.08259400	4.64591000
C	-4.80956400	-1.09309000	-0.42068300
H	-4.14484800	-2.99544600	0.29344700
C	-4.84408400	0.67228200	1.26442000
O	-4.08076800	0.21666900	3.50225700
C	0.50615200	-5.14184700	1.47061600
C	0.56979600	-3.31846700	3.24242700
H	0.59174200	-1.73417300	4.70524100
C	-1.79399700	0.21812200	4.26532700
C	-4.98942600	-1.55264000	-1.86575000
C	-5.06925000	0.22941200	-0.03703600
H	-5.04132700	1.70057400	1.53811500
C	-2.90939400	0.90722400	3.67180200
H	1.41808100	-5.42188100	1.99821900
C	0.94452300	-4.54424900	0.13583200
O	1.92513300	-3.61823200	3.39388200
C	-3.63240300	-1.47626900	-2.55710600
H	-5.63348200	-0.81990600	-2.35213900
O	-5.58946900	1.11762000	-0.98335300
C	0.06789700	-4.43705700	-0.94910500
C	2.24436300	-4.04081800	-0.03326300
C	2.80586900	-2.68172300	2.92611800
C	-0.52389800	2.09885500	4.06543200
C	-2.74057800	-2.55086200	-2.59543000
C	-3.21993400	-0.27269700	-3.14845000
C	-4.75963500	2.12825400	-1.39142200
C	-1.62872100	2.78580200	3.47641100
C	0.42172000	-3.83928400	-2.16431700
H	-0.94118200	-4.81550600	-0.83669600
C	2.65016300	-3.45439100	-1.22895100
O	3.14115900	-4.11805700	1.02600300
C	3.44477500	-2.93828600	1.66468600
C	-1.46776700	-2.46874900	-3.17421200
H	-3.05911600	-3.49960400	-2.18186900
C	-1.94660300	-0.12242400	-3.68893000
O	-4.15528300	0.74558300	-3.25080700
C	-3.97450500	1.92273100	-2.57784700
C	-0.56015600	-3.69223800	-3.32402300
C	1.72818900	-3.35110100	-2.26443900
H	3.64833100	-3.04654100	-1.32253800
C	3.89985300	-0.69029100	3.07454500
C	-1.08931800	-1.21790800	-3.67900900
H	-1.65287600	0.81711500	-4.14029800
C	-3.84191100	4.20359300	-1.16090800
H	0.04133800	-3.51676100	-4.21938400
O	2.10553500	-2.60309500	-3.40400700
C	4.53686800	-0.94526300	1.82020500
O	0.16831300	-1.04428600	-4.28748400
C	-3.05486600	3.99383300	-2.33598400
C	-1.47266100	4.12730700	3.05701900
C	0.70282600	2.77830800	4.24559200
C	5.41852700	0.01925700	1.28313100
C	4.15417300	0.52512100	3.75261100
C	-2.17424400	5.00769000	-2.77502400
C	-3.71825500	5.41648700	-0.44598200
C	-1.34904400	-4.98812000	-3.57254900
H	-2.00999200	-4.86716800	-4.43422700
H	-1.96482000	-5.28237300	-2.71903300
C	-0.34541100	-6.40879300	1.30425900
H	0.20808700	-7.16097500	0.73619700
H	-1.28868000	-6.22812800	0.78127100
C	-4.47451500	-3.88272100	2.87171100
H	-4.19877200	-4.54419500	3.69709700
H	-4.30019700	-4.42659100	1.93902700
C	-5.66746300	-2.92383400	-1.98335300
H	-6.65479400	-2.88985800	-1.51647200
H	-5.10532500	-3.72678200	-1.49816000
H	-5.79200300	-3.19425100	-3.03534300
H	-5.54568200	-3.67571000	2.93562800
H	-0.58811700	-6.82539200	2.28492100
H	-0.65716800	-5.80898700	-3.77626000
C	-2.84840100	6.38781300	-0.89395500
C	-2.07830400	6.18462700	-2.06394000
H	-1.40849100	6.96837700	-2.40302100
H	-2.75674000	7.32328300	-0.35156200
H	-1.59822400	4.83255800	-3.67730900
H	-4.32851900	5.55250200	0.44039000
C	0.82822700	4.09110800	3.83740100
H	1.52241500	2.24205300	4.71200900
C	-0.26151100	4.76439400	3.23385000
H	-2.31871800	4.62452300	2.59521500
C	4.99813300	1.46106300	3.19205000
H	3.66356100	0.68841000	4.70614200
C	5.63198700	1.20697500	1.95156300
H	5.90136200	-0.19509000	0.33893700
H	6.28101700	1.95961700	1.51725100
H	5.17199400	2.40658000	3.69325200
H	-0.13352500	5.78993600	2.90532600
H	1.77214800	4.60936300	3.96063400
P	1.53271000	-1.07187700	-3.39664200
N	2.59856800	-0.28789100	-4.37190700
C	3.96821200	-0.73823800	-4.63841200
C	2.30773700	1.09977400	-4.75022600
H	4.10608700	-1.75951600	-4.28879800
H	4.68755800	-0.07794000	-4.14272600
H	4.14754900	-0.71327800	-5.71798400
H	1.26767500	1.34724400	-4.52929700
H	2.45796800	1.22035700	-5.82720000
H	2.96549000	1.79126200	-4.21189300
Au	1.18512000	-0.56665200	-1.15394600
N	-3.14219300	2.81560600	-3.02957200
N	-4.68985200	3.23095100	-0.70524600
N	-2.82355700	2.14613600	3.28444900
N	-0.63976700	0.78980600	4.44351700
N	3.02523300	-1.59582400	3.60592300
N	4.27786900	-2.09809100	1.12653100
C	1.45781100	0.34837100	1.00719100
C	0.90358400	-0.75127400	1.03912200
H	0.40197100	-1.64731200	1.35050300
C	2.11111900	1.64043700	1.19964800
C	1.63262000	2.85112600	0.36651800
H	3.18581100	1.53133600	1.04251500
H	1.98393400	1.87556400	2.26041700
C	2.28154800	4.11125400	0.99541500
O	3.24287600	4.03082400	1.72308000
O	1.76765900	5.28347200	0.61259500
H	0.90730700	5.12467200	0.15368900
C	2.11295000	2.78534800	-1.13618900
H	1.61176500	1.92715800	-1.59896400
H	1.72732900	3.67914600	-1.63298000
C	0.11748700	3.00736300	0.25979300

O	-0.41423700	4.02271900	-0.16729100
O	-0.53067300	1.89935500	0.56468700
C	-1.92495300	1.79571400	0.21373700
H	-2.21239500	0.78757600	0.49974800
H	-2.51266500	2.52802700	0.75990700
H	-2.02578800	1.93827600	-0.85968700
C	3.60397400	2.67767700	-1.33737700
C	4.20443800	1.44509300	-1.61886800
C	4.41472800	3.81925700	-1.26566900
C	5.57997800	1.35180200	-1.83607000
H	3.59183500	0.54947600	-1.66906100
C	5.79085900	3.72629600	-1.46397000
H	3.96636500	4.78577800	-1.05745900
C	6.37836200	2.49236600	-1.75356200
H	6.02749200	0.38816900	-2.06093800
H	6.40366900	4.61972600	-1.40250400
H	7.44858800	2.42303800	-1.91976600

1a⊂AuCav-5

C	-0.15748300	2.21376200	4.59694400
C	-0.75526500	0.86130400	4.21918100
C	0.39879900	2.88903500	3.34667100
H	0.69592800	2.01001300	5.24419500
C	-2.11273000	0.64872700	3.97046600
C	0.09932400	-0.24286700	4.11341900
C	-0.45122500	3.61065400	2.50192100
C	1.75521300	2.83619600	2.98858200
C	-2.62794200	-0.61246200	3.64967100
H	-2.79763800	1.48304600	4.06232900
C	-0.35277500	-1.51498800	3.78624500
O	1.40595100	-0.01167000	4.50252100
C	-0.01883700	4.30817100	1.37072800
H	-1.50063700	3.66560800	2.76532600
C	2.22416700	3.46918800	1.83652800
O	2.68956100	2.26108600	3.85276500
C	-4.11716700	-0.85169100	3.39845800
C	-1.71691800	-1.67521100	3.55849500
H	0.32224700	-2.35977200	3.75305600
C	2.50849100	-0.14533300	3.72982600
C	-0.99519900	5.16613400	0.56761400
C	1.34309300	4.20375600	1.04315500
H	3.28045200	3.45210500	1.59567800
C	3.26123800	1.06447100	3.51182700
H	-4.31865400	-1.88648700	3.67747100
C	-4.38444600	-0.74192200	1.90090600
O	-2.24125500	-2.94725300	3.35142600
C	-1.72485200	4.29875000	-0.45123400
H	-0.38775800	5.87113500	-0.00041000
O	1.85781700	4.93752200	-0.02742800
C	-4.53076700	0.49976200	1.26797100
C	-4.40648200	-1.88449700	1.08991800
C	-2.01405300	-3.62685700	2.19595700
C	4.20787900	-1.36563600	2.84371900
C	-2.91326200	3.61354900	-0.18139700
C	-1.15424100	4.12744400	-1.71856700
C	2.17515000	4.25516500	-1.17509500
C	5.01634400	-0.19026500	2.78744400
C	-4.62845600	0.64561800	-0.12065100
H	-4.54218300	1.39226700	1.88285900
C	-4.47868000	-1.79113200	-0.29472800
O	-4.30564800	-3.15162900	1.66440700
C	-3.14890800	-3.81516800	1.33440200
C	-3.51416500	2.74385100	-1.10392900
H	-3.38449700	3.74815400	0.78512200
C	-1.70959800	3.28561600	-2.66973600
O	0.00673200	4.82818600	-1.99542900
C	1.16164100	4.12169800	-2.18314100
C	-4.82170600	2.00192100	-0.80833400
C	-4.55368100	-0.53595500	-0.87071700
H	-4.41852200	-2.68231600	-0.90604700
C	-0.74688000	-4.87659500	0.77729100
C	-2.86337400	2.59169900	-2.33660800
H	-1.22631800	3.14340700	-3.62674500
C	3.61535700	3.09056100	-2.50205100
H	-5.26873900	1.78315100	-1.78168400
O	-4.43774900	-0.47993900	-2.27295100
C	-1.88744300	-5.10286200	-0.05032400
O	-3.29609900	1.62693700	-3.26399900
C	2.56253800	2.83867700	-3.43436900
C	6.37537600	-0.29771700	2.41981000
C	4.76330000	-2.60986500	2.47310300
C	-1.75273000	-5.86831100	-1.23004900
C	0.50362500	-5.41814600	0.40129100
C	2.81054300	2.03843000	-4.57302900
C	4.91028000	2.59458000	-2.77165200
C	-5.82483300	2.88517500	-0.04808900
H	-6.00113700	3.81241100	-0.59882700
H	-5.47798600	3.15436300	0.95288400
C	-5.03405800	0.04587300	4.23990200
H	-6.07882000	-0.22516000	4.06800000
H	-4.92933700	1.10907400	4.00570700
C	-1.12370500	3.11345200	5.37945600
H	-1.45182500	2.60507300	6.28987300
H	-2.01858000	3.38469600	4.81244500
C	-1.94392300	5.98147900	1.45787500
H	-1.36874500	6.61032000	2.14188400
H	-2.60854000	5.35927500	2.06363000
H	-2.57146000	6.62769300	0.83851500
H	-0.62203000	4.04189000	5.66336700
H	-4.81440200	-0.08155600	5.30303200
H	-6.77583400	2.35879400	0.06332200
C	5.13218700	1.83071500	-3.89633100
C	4.07647200	1.53880000	-4.79239300
H	4.27456900	0.93165200	-5.66990900
H	6.12380200	1.43920700	-4.09501600
H	1.99501200	1.86426800	-5.26695600
H	5.70037500	2.81630800	-2.06519600
C	6.08984900	-2.68547300	2.10167400
H	4.12848100	-3.48651500	2.54079200
C	6.90209700	-1.52797800	2.08754500
H	6.97484900	0.60613900	2.40856500
C	0.61023700	-6.15451200	-0.76182800
H	1.35325800	-5.24173400	1.05136800
C	-0.52119900	-6.37967400	-1.58091900
H	-2.63379300	-6.02212600	-1.84292200
H	-0.41438700	-6.96018500	-2.49106700
H	1.56735600	-6.57529200	-1.05228200
H	7.94849500	-1.61333000	1.81331300
H	6.52826700	-3.64446500	1.84368000
P	-3.00224500	0.08398500	-2.79956200
N	-2.81720700	-0.71362200	-4.23784600
C	-3.19415900	-2.13302700	-4.35357700
C	-1.64062100	-0.33540100	-5.03142900
H	-4.11024700	-2.31800100	-3.79573200
H	-2.39905300	-2.79228500	-3.98788600
H	-3.38350600	-2.34368900	-5.40959800
H	-1.42385600	0.72697400	-4.90585600
H	-1.85991800	-0.51169200	-6.08781400
H	-0.75795900	-0.92530100	-4.74984300
Au	-1.42039800	0.11308600	-1.07013700
N	1.32780100	3.39793400	-3.25016500
N	3.37089700	3.77180100	-1.33885900
N	4.48232400	1.04033700	3.07116000
N	2.93164900	-1.31252300	3.34279600
N	-0.84335500	-4.12168600	1.91747600
N	-3.09418400	-4.53473600	0.25588900
C	0.38321300	-0.54578400	0.33556100
C	-0.08671400	0.54420100	0.66667900
H	-0.28029300	1.47317100	1.17251500
C	1.09076900	-1.81503500	0.14350700
C	1.34475600	-2.24662200	-1.32103700
H	0.55085700	-2.59617700	0.68242900
H	2.05497100	-1.69342700	0.64608100
C	0.04085800	-2.75305800	-1.98692500
O	-1.05604800	-2.56897600	-1.49584900
O	0.17304700	-3.33245800	-3.17690400
H	1.11768000	-3.61646300	-3.28250100
C	1.91731200	-1.06131000	-2.19571700
H	1.21201800	-0.22774600	-2.13330200
H	1.93674900	-1.39106900	-3.23619500

C	2.39682200	-3.35554600	-1.42227000
O	2.58772700	-3.95642300	-2.47426400
O	3.10679500	-3.55220200	-0.32681200
C	4.21291400	-4.48107600	-0.44401700
H	4.49617200	-4.72421400	0.57582200
H	3.90223600	-5.36784800	-0.99522400
H	5.03244600	-3.98086800	-0.96241600
C	3.29884400	-0.64083600	-1.75731900
C	4.42647900	-1.25421300	-2.32092100
C	3.48187600	0.31705000	-0.75392900
C	5.70663800	-0.94286500	-1.86502900
H	4.29805700	-1.98222400	-3.11611500
C	4.76203600	0.63958700	-0.30818400
H	2.62470000	0.83264100	-0.33301000
C	5.87529900	0.00030900	-0.85203300
H	6.57068300	-1.42487500	-2.31247400
H	4.88487700	1.39849900	0.45515700
H	6.86987700	0.24911700	-0.49923200

1a⊂AuCav-6

C	-0.15739100	-1.80527000	4.78290900
C	0.47189900	-0.49019200	4.33030400
C	-0.64484100	-2.57523200	3.55857400
H	-1.04457000	-1.54745600	5.36189100
C	1.84311600	-0.28362600	4.16936400
C	-0.37993100	0.58680700	4.05456600
C	0.26232400	-3.34742100	2.82569000
C	-1.97739700	-2.56720300	3.11419300
C	2.36848600	0.95277100	3.77518200
H	2.52542100	-1.09662700	4.38743700
C	0.08298700	1.83341600	3.65395200
O	-1.70484600	0.35878400	4.36070800
C	-0.09004800	-4.13528800	1.72742300
H	1.29312300	-3.36827700	3.15888800
C	-2.36319900	-3.29225000	1.98421600
O	-2.97901500	-1.94866100	3.86697500
C	3.86596600	1.19708500	3.59213500
C	1.46002100	1.99112400	3.52652800
H	-0.59437200	2.66144500	3.49331900
C	-2.76186000	0.43382000	3.52098400
C	0.94392100	-5.03971900	1.06087200
C	-1.42833700	-4.07455900	1.30620900
H	-3.40176400	-3.30640500	1.67499400
C	-3.52874700	-0.77955000	3.40636700
H	4.03959000	2.25452300	3.79366600
C	4.21627500	0.96674700	2.12548400
O	1.99930700	3.24453600	3.26146700
C	1.75372700	-4.24260200	0.04538100
H	0.38147900	-5.78293200	0.49532900
O	-1.86495000	-4.89192900	0.26185900
C	4.42438600	-0.32293700	1.62183300
C	4.27274000	2.03038700	1.21338200
C	1.82700100	3.85654100	2.06116400
C	-4.39810600	1.57894800	2.44302000
C	2.93078000	-3.55415600	0.35486400
C	1.27854000	-4.15667500	-1.27042400
C	-2.10441600	-4.29015200	-0.94956300
C	-5.24071000	0.42834000	2.51915000
C	4.63870500	-0.59740000	0.26682500
H	4.40338800	-1.15331100	2.31757800
C	4.45639400	1.80712400	-0.14786100
O	4.13989700	3.34331100	1.65860300
C	3.00010100	3.98893500	1.24297800
C	3.62374500	-2.77932200	-0.58731100
H	3.32160700	-3.61673900	1.36367300
C	1.93347800	-3.41668700	-2.24387800
O	0.12862100	-4.85921100	-1.57475900
C	-1.01635800	-4.17384800	-1.87820300
C	4.90549500	-2.01293500	-0.25417300
C	4.60924200	0.50504000	-0.59781400
H	4.46244000	2.63994200	-0.84028200
C	0.63589300	5.05659200	0.53836900
C	3.08823600	-2.73346200	-1.88147400
H	1.53569000	-3.35106800	-3.24775000
C	-3.46500100	-3.24437500	-2.44861100

H	5.43977600	-1.89181200	-1.20003200
O	4.65977900	0.32118300	-1.99376900
C	1.82155700	5.25525600	-0.23428500
O	3.65284500	-1.89471300	-2.85402000
C	-2.34379300	-2.99197000	-3.29856700
C	-6.59048100	0.53021400	2.11860700
C	-4.90193100	2.77622000	1.89093600
C	1.75477200	5.99985100	-1.43415400
C	-0.59102100	5.58622300	0.07981000
C	-2.52049800	-2.24701400	-4.48702700
C	-4.74909300	-2.80981500	-2.84684000
C	5.83154900	-2.80560600	0.68212000
H	6.06266700	-3.77904300	0.24261700
H	5.38998300	-2.98329900	1.66598000
C	4.74530400	0.38812700	4.55459100
H	5.79587400	0.65463800	4.41359600
H	4.65962800	-0.69295500	4.41272500
C	0.75525400	-2.64202100	5.68948900
H	1.03086400	-2.06422100	6.57565000
H	1.68126100	-2.95745400	5.20054700
C	1.81850000	-5.79105700	2.07500800
H	1.18933500	-6.37894200	2.74770700
H	2.42941700	-5.12758900	2.69342400
H	2.49560300	-6.47136100	1.55169500
H	0.23255400	-3.54498100	6.01452400
H	4.46913800	0.60767100	5.58905000
H	6.76623800	-2.26042900	0.83393400
C	-4.89885200	-2.10058800	-4.01776100
C	-3.77957000	-1.80477500	-4.83253500
H	-3.92089100	-1.23660900	-5.74627700
H	-5.88151000	-1.75118500	-4.31450900
H	-1.65471100	-2.06270800	-5.11400300
H	-5.58747300	-3.02696800	-2.19718300
C	-6.21694800	2.84119700	1.47989300
H	-4.23771700	3.63162800	1.84888800
C	-7.06908200	1.72049600	1.61292500
H	-7.21937800	-0.34856000	2.21101300
C	-0.63295200	6.29062300	-1.10517600
H	-1.47621000	5.41840800	0.68275300
C	0.54344500	6.50279800	-1.86227800
H	2.67088300	6.15357800	-1.99503000
H	0.48769500	7.06918000	-2.78598300
H	-1.57267300	6.69978900	-1.46071800
H	-8.10687900	1.79992100	1.30626600
H	-6.61361600	3.76659800	1.07436700
P	3.30159500	-0.29313100	-2.65997400
N	3.28990900	0.39304600	-4.15155700
C	4.52213400	0.80686300	-4.84031100
C	2.18912200	-0.01407200	-5.03961700
H	5.25890400	1.14975300	-4.11570000
H	4.27502900	1.63192000	-5.51351700
H	4.94520100	-0.01720200	-5.42676800
H	2.47511100	-0.88146700	-5.64603100
H	1.93466600	0.82455400	-5.69074100
H	1.29996100	-0.25325900	-4.45413400
Au	1.51759100	-0.19144000	-1.14121900
N	-1.11234500	-3.50236800	-2.98715500
N	-3.29670800	-3.86057200	-1.23728800
N	-4.74273800	-0.77625900	2.94736600
N	-3.13961700	1.55765200	2.98844300
N	0.67331200	4.34311100	1.70614300
N	3.00289100	4.66861100	0.13576100
C	-0.37898400	0.45141300	0.19026200
C	0.15479300	-0.59100500	0.57391200
H	0.38137200	-1.48263100	1.13265400
C	-1.14213600	1.65453900	-0.13706600
C	-1.36973600	1.86881100	-1.65591700
H	-0.65672300	2.52363000	0.31251200
H	-2.11598600	1.54588900	0.35132100
C	-0.06117800	2.17617500	-2.39624400
O	0.23811400	1.75556200	-3.48998300
O	0.72618200	3.00258600	-1.68336000
H	1.49514500	3.23963000	-2.22847500
C	-2.03815100	0.64435400	-2.34218300
H	-1.40352500	-0.23040400	-2.17066900

H	-2.02300600	0.83383100	-3.41558900
C	-2.27220500	3.08834200	-1.91584500
O	-2.34766100	3.61203600	-3.00462100
O	-2.98515800	3.44563800	-0.84165400
C	-3.98798600	4.45828400	-1.07080600
H	-4.21679000	4.87995200	-0.09450700
H	-3.60842900	5.21873300	-1.75247800
H	-4.87438700	3.98397400	-1.49713300
C	-3.44389200	0.37506400	-1.87451700
C	-4.52549300	0.98309400	-2.52549700
C	-3.69742400	-0.49148600	-0.80683100
C	-5.83354200	0.72779500	-2.11734200
H	-4.33666400	1.64767700	-3.36367000
C	-5.00534800	-0.76490000	-0.41095900
H	-2.87237500	-0.99468900	-0.31000800
C	-6.07506300	-0.15367000	-1.06356400
H	-6.66319400	1.19958600	-2.63528300
H	-5.18438000	-1.46257000	0.39861100
H	-7.09243200	-0.36753600	-0.75478600

1a⊂AuCav-7

C	0.64759400	-4.36455800	3.10246000
C	0.97482600	-4.30406900	1.61182800
C	0.05319700	-3.03119200	3.54545800
H	1.59885900	-4.46979900	3.62428700
C	-0.00159200	-4.56722700	0.64839400
C	2.25569700	-3.97416500	1.14638200
C	-1.32029800	-2.76947600	3.55905800
C	0.91075000	-1.99649100	3.94315400
C	0.24894200	-4.54514300	-0.72617500
H	-1.00174800	-4.81440400	0.98552200
C	2.55129100	-3.92241800	-0.21392700
O	3.28194500	-3.78572600	2.07170100
C	-1.84676800	-1.53059300	3.94116300
H	-2.00322700	-3.56526100	3.28609000
C	0.44262600	-0.73430600	4.29578800
O	2.25093200	-2.31652700	4.08212600
C	-0.84933800	-4.87023600	-1.73419300
C	1.54753800	-4.21011600	-1.13615600
H	3.55637700	-3.69621400	-0.54971900
C	3.78507100	-2.52894900	2.26746900
C	-3.34659900	-1.24975100	4.01787500
C	-0.93195400	-0.52364800	4.27763000
H	1.12672400	0.04527900	4.60397300
C	3.21132200	-1.72771600	3.31564700
H	-0.34750300	-5.10775800	-2.67254800
C	-1.68750800	-3.62422900	-1.99666200
O	1.86570700	-4.27203900	-2.49268900
C	-3.85116800	-0.66399400	2.70290400
H	-3.47590000	-0.47097800	4.77009000
O	-1.46433500	0.68979100	4.71456700
C	-2.85842600	-3.33055400	-1.29591500
C	-1.28018500	-2.71499900	-2.98445200
C	2.10377700	-3.10234300	-3.16667300
C	5.27975900	-0.86419800	1.84059900
C	-4.16588900	-1.48395500	1.61384700
C	-4.00530200	0.71847200	2.52676600
C	-1.36485900	1.81076800	3.95849600
C	4.68049700	-0.05680300	2.85464600
C	-3.63780400	-2.19796700	-1.55748800
H	-3.19546400	-4.02538400	-0.53700300
C	-2.00749600	-1.56150900	-3.26936500
O	-0.20749900	-3.08163900	-3.76574900
C	0.99240700	-2.42270800	-3.77019200
C	-4.61956900	-0.99093300	0.38683200
H	-4.04307400	-2.55465700	1.72534200
C	-4.44614800	1.25930400	1.32056500
O	-3.73313400	1.58665900	3.58269500
C	-2.59794700	2.34209100	3.43432800
C	-4.92577400	-1.90630500	-0.79437700
C	-3.17570200	-1.32934600	-2.55296900
H	-1.69309800	-0.90326700	-4.06982100
C	3.51266000	-1.54908400	-4.05753400
C	-4.73983400	0.39723900	0.27391000
H	-4.56073000	2.33105100	1.21646600
C	-0.24826100	3.60758800	3.11848600
H	-5.57813300	-1.34877500	-1.47055500
O	-3.97862100	-0.24229200	-2.95743400
C	2.39574700	-0.82954900	-4.59016700
O	-5.18044300	0.95880600	-0.94206800
C	-1.48412900	4.17714200	2.68288500
C	5.20748700	1.22715600	3.12700900
C	6.39914900	-0.37356400	1.13028800
C	2.61918400	0.33433300	-5.35924200
C	4.82346500	-1.09592500	-4.32401800
C	-1.48970900	5.45580900	2.07742000
C	0.95588400	4.31719500	2.90618300
C	-5.69390200	-3.16966800	-0.38121600
H	-6.63937600	-2.89030400	0.08992100
H	-5.14521400	-3.79224500	0.32962200
C	-1.68655000	-6.09211500	-1.33178400
H	-2.43287700	-6.30910100	-2.10086000
H	-2.21497600	-5.96051700	-0.38327000
C	-0.22135300	-5.57369900	3.47405200
H	0.28169900	-6.49829500	3.18019900
H	-1.20170400	-5.56704900	2.98885900
C	-4.16606700	-2.46827600	4.46772700
H	-3.80141600	-2.82669100	5.43354000
H	-4.11486600	-3.30468700	3.76516100
H	-5.21840300	-2.19290600	4.57503800
H	-0.38799600	-5.59820900	4.55426600
H	-1.03768100	-6.96454300	-1.22377100
H	-5.91055500	-3.78133200	-1.26046000
C	0.92662500	5.56140000	2.30878600
C	-0.29937700	6.13568000	1.89772200
H	-0.30273000	7.12218300	1.44559300
H	1.85176500	6.10517000	2.15320700
H	-2.44412700	5.88090900	1.78372900
H	1.88348100	3.86616900	3.24135400
C	6.91010000	0.87129900	1.43316500
H	6.83163700	-1.00245600	0.36071500
C	6.30931400	1.67695000	2.43020500
H	4.73679800	1.81901100	3.90461000
C	5.01350900	0.02814700	-5.09664900
H	5.65138500	-1.65661200	-3.90576200
C	3.90940000	0.74692100	-5.61203600
H	1.75915900	0.85828700	-5.76290400
H	4.08507300	1.63154700	-6.21551600
H	6.01951600	0.37486500	-5.30842700
H	6.72127200	2.65774100	2.64332600
H	7.77820800	1.24334500	0.89855700
P	-4.09573200	1.14453200	-2.13727700
N	-4.96372800	2.06443400	-3.21057700
C	-4.46235200	2.15957500	-4.59031500
C	-5.56072800	3.30939400	-2.70311700
H	-4.11142200	1.18614500	-4.93040600
H	-3.65360100	2.89777600	-4.68452000
H	-5.29183000	2.46771100	-5.23131400
H	-5.98092300	3.14778800	-1.71101500
H	-6.37296300	3.59461900	-3.37600100
H	-4.83156800	4.13097200	-2.66631300
Au	-2.09801500	1.95606900	-1.33877900
N	-2.65822200	3.48350000	2.81805000
N	-0.22191800	2.40045400	3.76551000
N	3.62085800	-0.52593300	3.58523400
N	4.78018000	-2.10771900	1.54703600
N	3.32348500	-2.67566900	-3.30501700
N	1.12012800	-1.30106000	-4.41721800
C	0.18391000	2.69314900	-1.11334100
C	-0.60921500	3.41338400	-0.50950400
H	-1.02880300	4.20663000	0.08158900
C	1.24469500	1.91667500	-1.74222700
C	2.12069800	1.13958300	-0.73378200
H	1.90378200	2.60718900	-2.27718600
H	0.83414600	1.23381800	-2.49171600
C	3.36248600	0.64581700	-1.52369100
O	3.73573000	1.25049900	-2.49995000
O	4.03032900	-0.39629600	-1.03462700
H	3.56005600	-0.78557200	-0.26533400
C	2.65328100	2.07248200	0.42436200

H	1.78493900	2.40827300	0.99901800
H	3.25454600	1.44895200	1.08880300
C	1.39289700	-0.00641500	-0.05060600
O	1.97800200	-0.84015200	0.62482600
O	0.07269200	0.00625200	-0.21053300
C	-0.66650300	-1.01133400	0.51963400
H	-0.49720400	-0.88410900	1.58716400
H	-1.70865700	-0.84388600	0.26182100
H	-0.34056400	-2.00156500	0.20698600
C	3.45576900	3.25700700	-0.05637000
C	2.85263800	4.50348200	-0.26340500
C	4.82564100	3.12293900	-0.31358700
C	3.59976200	5.59333300	-0.71208500
H	1.79731000	4.62971000	-0.05461500
C	5.57487100	4.20940600	-0.75888300
H	5.30680800	2.16409300	-0.16248700
C	4.96483000	5.44889200	-0.95990500
H	3.11647300	6.55390200	-0.86433800
H	6.63623400	4.08729600	-0.95115300
H	5.54938400	6.29547700	-1.30587400

1a⊂AuCav-8

C	-1.16857600	-1.34366200	4.69982300
C	0.09768000	-1.66624000	3.91275100
C	-2.23011500	-0.66387400	3.84254800
H	-0.87431800	-0.61203600	5.45230200
C	0.21283200	-2.88143000	3.23733400
C	1.22467400	-0.82703000	3.90881100
C	-3.17989600	-1.34954800	3.08119600
C	-2.29278900	0.73343700	3.83350700
C	1.39170800	-3.33442200	2.64365600
H	-0.64999700	-3.53528300	3.21921300
C	2.42152000	-1.22835800	3.31160100
O	1.20497500	0.36242000	4.65445800
C	-4.18148400	-0.69532300	2.34974600
H	-3.14784900	-2.43288500	3.06205900
C	-3.27710400	1.43355100	3.14886800
O	-1.34079100	1.40427100	4.59263400
C	1.42401100	-4.69929800	1.95489800
C	2.50532100	-2.48171500	2.69943400
H	3.29844900	-0.59870500	3.38249400
C	0.99136200	1.56330800	4.03061600
C	-5.18970800	-1.46242000	1.49485000
C	-4.21215600	0.70531200	2.41537300
H	-3.31217300	2.51399900	3.17383900
C	-0.35416200	2.06970900	3.92820300
H	2.46873400	-5.00378900	1.89883800
C	0.94048500	-4.48102600	0.52669400
O	3.75295800	-2.95731900	2.27931300
C	-4.57514500	-1.75085500	0.12759700
H	-6.02910200	-0.78798400	1.32374700
O	-5.22160100	1.39751100	1.73057600
C	-0.39978800	-4.52892000	0.14000000
C	1.87974400	-4.10567800	-0.44557100
C	4.36231600	-2.51067100	1.13727600
C	1.74016500	3.43330100	2.94277800
C	-3.79007900	-2.88149200	-0.11990600
C	-4.73744500	-0.84136700	-0.92756000
C	-4.77457900	2.10821500	0.64922800
C	0.39243200	3.85193800	2.72299000
C	-0.83528100	-4.15118100	-1.13885200
H	-1.13567400	-4.87392100	0.85630000
C	1.49560200	-3.65812400	-1.70736600
O	3.20982000	-4.21674300	-0.09474800
C	4.02803600	-3.11797500	-0.12158100
C	-3.14866300	-3.11107700	-1.34241400
H	-3.65696300	-3.60342700	0.67687100
C	-4.12946300	-1.02939000	-2.16412300
O	-5.51978600	0.28778300	-0.71331900
C	-4.86782600	1.48984300	-0.64422600
C	-2.28121000	-4.33790600	-1.59864300
C	0.13678600	-3.64464000	-2.00979900
H	2.23172700	-3.31507400	-2.42226400
C	5.91642000	-1.22916300	0.05347700
C	-3.32824300	-2.15129500	-2.34510000
H	-4.25386100	-0.29877200	-2.95214300
C	-3.73038100	3.90030800	-0.29344200
H	-2.23699600	-4.45808400	-2.68422400
O	-0.21745700	-3.14554600	-3.27385000
C	5.46311800	-1.72651100	-1.20662500
O	-2.60776100	-2.24070600	-3.54772000
C	-3.75913800	3.25301600	-1.56788900
C	0.12695200	5.00074400	1.94428400
C	2.79429900	4.21201800	2.40987600
C	6.02961600	-1.23403600	-2.40309100
C	6.98998600	-0.31026400	0.08610600
C	-3.18577900	3.89671900	-2.68882400
C	-3.16164200	5.18923100	-0.17721400
C	-2.91089900	-5.62177800	-1.03460000
H	-3.90546900	-5.76600000	-1.46370200
H	-3.02150600	-5.60027500	0.05240800
C	0.65787900	-5.79219700	2.70973000
H	0.77008200	-6.74951100	2.19375900
H	-0.41309800	-5.58962100	2.79846700
C	-1.72558200	-2.56565000	5.44853200
H	-0.95207600	-3.00070500	6.08637300
H	-2.08572400	-3.35347000	4.78109800
C	-5.73339700	-2.71861200	2.18980800
H	-6.21004800	-2.44387100	3.13393000
H	-4.95731900	-3.45474600	2.41708500
H	-6.47787000	-3.20711100	1.55564700
H	-2.56513800	-2.26167800	6.07913300
H	1.05630900	-5.89682500	3.72178500
H	-2.29301800	-6.48670300	-1.28797700
C	-2.63209900	5.80525700	-1.29072400
C	-2.63493800	5.15371500	-2.54824600
H	-2.20845100	5.65602300	-3.41033600
H	-2.21622800	6.80398800	-1.20725300
H	-3.22317900	3.39156400	-3.64813600
H	-3.17791800	5.66852400	0.79549200
C	2.51150700	5.32818200	1.65116000
H	3.81564900	3.90988000	2.60042200
C	1.17386500	5.72134300	1.41207800
H	-0.90777700	5.28042600	1.78370800
C	7.54258500	0.13846100	-1.09348500
H	7.35804900	0.02525400	1.04884900
C	7.04876000	-0.30862900	-2.34186000
H	5.65340900	-1.61802000	-3.34463300
H	7.49179900	0.07037400	-3.25694600
H	8.36337500	0.84766500	-1.06886000
H	0.97892500	6.59459700	0.80204100
H	3.31949700	5.90865100	1.22051300
P	-1.03213600	-1.74318200	-3.42884800
N	-0.54104200	-1.23301400	-4.90680600
C	-0.24285000	-2.15805600	-6.00939200
C	-0.84946900	0.14293400	-5.30761500
H	0.05237900	-3.12797900	-5.61260700
H	0.58940100	-1.74942200	-6.58895500
H	-1.11248000	-2.28024800	-6.66510600
H	-1.76316600	0.19096800	-5.91190200
H	-0.01240300	0.53749400	-5.89021400
H	-0.97892000	0.77405900	-4.42406800
Au	-0.97917100	-0.25119500	-1.63853100
N	-4.35133000	2.02717800	-1.71087400
N	-4.23124400	3.27812100	0.81561200
N	-0.64494000	3.14734300	3.26252300
N	2.00485600	2.25199900	3.58662400
N	5.30554500	-1.61776600	1.21748900
N	4.52052100	-2.71532100	-1.25295200
C	-0.24759200	1.27976900	0.04785200
C	-1.43126500	0.95356900	0.15389000
H	-2.42113100	0.82977400	0.54682100
C	1.12633300	1.73806900	0.24226100
C	2.20356800	1.30868800	-0.78290800
H	1.41538900	1.36828400	1.22604000
H	1.11026800	2.83128500	0.27967000
C	2.08565800	-0.18728300	-1.10902100
O	2.23340000	-0.65608400	-2.21616400
O	1.77044300	-0.91523500	-0.02204600
H	1.63589500	-1.84064100	-0.30833200

C	2.11082000	2.09015700	-2.11529600
H	1.18698200	1.77195600	-2.60997500
H	2.93604200	1.74957000	-2.74128500
C	3.61032900	1.48978400	-0.14664800
O	4.60212200	1.73041100	-0.79582200
O	3.56902400	1.31036000	1.17350600
C	4.78186100	1.47772400	1.94461500
H	4.44675900	1.84048400	2.91386400
H	5.25571500	0.50364700	2.04896500
H	5.44700000	2.18166300	1.44420500
C	2.11941700	3.59656600	-1.99412200
C	3.32530500	4.30949700	-1.92306000
C	0.91491600	4.31313700	-2.00313900
C	3.32488900	5.70276600	-1.87003600
H	4.26164700	3.76409500	-1.92256100
C	0.91588800	5.70729900	-1.95820700
H	-0.03187300	3.78164100	-2.06754000
C	2.12025100	6.40743000	-1.89400000
H	4.26825100	6.23900300	-1.82973800
H	-0.02485600	6.24485900	-1.97851100
H	2.12111200	7.49299900	-1.87102200

1a⊂AuCav-9

C	-2.27629000	1.50069000	4.34331000
C	-1.53373900	2.41346700	3.37486200
C	-2.25694700	0.07871600	3.79318900
H	-3.31803200	1.82097300	4.34268400
C	-0.17318500	2.70041800	3.48898300
C	-2.23082900	2.99450100	2.30809000
C	-1.18222400	-0.77427300	4.05000900
C	-3.31283500	-0.43455300	3.02508800
C	0.50045100	3.53108600	2.59016100
H	0.37746400	2.28218100	4.32275000
C	-1.60003600	3.79976400	1.36097600
O	-3.61120000	2.85657100	2.33585400
C	-1.12817100	-2.09724700	3.61039600
H	-0.36335900	-0.39845000	4.65111600
C	-3.28044400	-1.73868200	2.52071500
O	-4.48019300	0.31772400	2.90853600
C	1.96640800	3.91327700	2.77333200
C	-0.23482200	4.03997300	1.51227500
H	-2.16218600	4.26808000	0.56283700
C	-4.37265600	2.21847200	1.40605500
C	0.01965600	-3.01446800	4.01902300
C	-2.18059600	-2.55030900	2.80230600
H	-4.12902200	-2.13427400	1.97618300
C	-4.87158500	0.90549200	1.73525200
H	2.10310500	4.86293900	2.25628500
C	2.87801000	2.90899200	2.08111000
O	0.42485800	4.91689000	0.64737900
C	1.19164700	-2.80689500	3.07266900
H	-0.32924100	-4.03537900	3.86710700
O	-2.17706000	-3.88706000	2.40005200
C	3.20931300	1.69011500	2.67616700
C	3.38297200	3.16043400	0.80038200
C	0.76436700	4.52336100	-0.60864300
C	-5.72363800	2.27351700	-0.42868700
C	2.17292600	-1.83828600	3.28873500
C	1.29002200	-3.58724000	1.91465500
C	-1.89525200	-4.25903700	1.11469800
C	-6.25811200	0.99762500	-0.07322000
C	4.01311500	0.73258600	2.05323600
H	2.80582700	1.46786900	3.65652100
C	4.20455200	2.24921700	0.14329400
O	3.04402000	4.35703700	0.16809500
C	2.16089700	4.26907100	-0.87514500
C	3.23538000	-1.62338700	2.40691300
H	2.12055300	-1.24091600	4.19006200
C	2.30687200	-3.38889300	0.98398500
O	0.38883400	-4.63247100	1.78358200
C	-0.54527600	-4.65899700	0.78785600
C	4.33648100	-0.60883200	2.69987500
C	4.49209600	1.04977100	0.78108600
H	4.58498600	2.46897000	-0.84612600
C	0.28303900	4.09588000	-2.79390400
C	3.25484000	-2.40209800	1.24358800
H	2.37155900	-4.01515500	0.10268900
C	-2.54035400	-4.80529900	-1.00307700
H	5.24287800	-0.98368200	2.21783000
O	5.31207100	0.11726700	0.09950900
C	1.66606900	3.86750800	-3.06075900
O	4.33590500	-2.29845700	0.34599600
C	-1.20396200	-5.18683000	-1.32255500
C	-7.30790100	0.44571200	-0.84156100
C	-6.24247900	2.96082100	-1.54971900
C	2.07646900	3.53871700	-4.37271500
C	-0.65814000	3.96897400	-3.84212300
C	-0.90650200	-5.67378800	-2.61501400
C	-3.55217400	-4.94868400	-1.97845500
C	4.65717700	-0.48025400	4.19626900
H	4.91432100	-1.45765200	4.61231300
H	3.82403600	-0.08032700	4.77911900
C	2.34749300	4.13057100	4.24580100
H	3.38128000	4.47902700	4.31605200
H	2.26556600	3.22236900	4.84940000
C	-1.76703000	1.59482300	5.78779600
H	-1.84920100	2.62460000	6.14535800
H	-0.72277000	1.29012800	5.90011500
C	0.39828900	-2.86983700	5.49994800
H	-0.47142200	-3.07698200	6.12844500
H	0.75797700	-1.87023000	5.75896600
H	1.18768200	-3.58194700	5.75520100
H	-2.36720600	0.95334800	6.43812900
H	1.69606200	4.88493400	4.69444600
H	5.50640800	0.19317100	4.33606000
C	-3.24084500	-5.43177100	-3.23355300
C	-1.91108800	-5.79128300	-3.55556400
H	-1.68563200	-6.17754700	-4.54449800
H	-4.02247300	-5.55068600	-3.97695400
H	0.11699700	-5.96248900	-2.82891300
H	-4.56908500	-4.69781700	-1.70096700
C	-7.27281000	2.40319600	-2.27697100
H	-5.82599300	3.93256700	-1.79212200
C	-7.80964200	1.14363700	-1.91947300
H	-7.70122700	-0.52104400	-0.54786000
C	-0.23406400	3.63253100	-5.11196900
H	-1.70262900	4.15792400	-3.61624700
C	1.13941500	3.42287700	-5.37976700
H	3.13650400	3.39520100	-4.55505400
H	1.45552600	3.18193400	-6.38987500
H	-0.95518600	3.54590300	-5.91847600
H	-8.62604400	0.72725000	-2.50084300
H	-7.68683900	2.93800300	-3.12595100
P	4.59657300	-1.08724400	-0.71454400
N	5.83164000	-1.62164400	-1.67066000
C	7.20446300	-1.68892800	-1.14042400
C	5.51295500	-2.66581500	-2.65224600
H	7.38906100	-0.84363500	-0.47929800
H	7.89798600	-1.63144700	-1.98314800
H	7.37797400	-2.62533500	-0.59674300
H	4.49914700	-2.52541100	-3.03748400
H	5.58774300	-3.67010800	-2.21719200
H	6.21201600	-2.58621700	-3.48877100
Au	2.76513600	-0.36414300	-1.90742200
N	-0.20972700	-5.09442400	-0.38616000
N	-2.85463500	-4.31970100	0.24121800
N	-5.78070700	0.31817200	1.01398300
N	-4.75632200	2.86027500	0.34024500
N	-0.14440300	4.41891500	-1.53203900
N	2.59359500	3.94847200	-2.05579000
C	0.81351400	0.37140300	-3.03642300
C	1.72564000	0.14903500	-3.82983200
H	2.35151000	0.03701700	-4.69069200
C	-0.41005300	0.74173500	-2.33147800
C	-0.65332600	0.12889400	-0.94390500
H	-1.24501000	0.52049300	-2.99764600
H	-0.38610900	1.82324900	-2.19946100
C	0.40598300	0.73379400	0.02971300
O	1.09888600	1.66204100	-0.34092400
O	0.46774300	0.25642400	1.25778200

H	-0.01967100	-0.60663500	1.28744200
C	-2.05403100	0.61963900	-0.42125100
H	-2.16360400	0.26839900	0.60631100
H	-2.02470300	1.71148200	-0.39629900
C	-0.63610100	-1.39929900	-0.82563900
O	-0.54715500	-1.87516900	0.29993900
O	-0.67048800	-2.24624600	-1.84572900
C	-1.26703000	-2.05822700	-3.15169900
H	-0.53416900	-1.63337700	-3.83729600
H	-2.16619100	-1.45073500	-3.08980100
H	-1.53049400	-3.06490200	-3.46955400
C	-3.18036100	0.12397800	-1.28711200
C	-3.62239200	0.88454100	-2.37796300
C	-3.75055400	-1.13483800	-1.06001500
C	-4.58764500	0.38302400	-3.25027600
H	-3.22076100	1.88243600	-2.53051100
C	-4.73213900	-1.62555800	-1.91889100
H	-3.43388900	-1.73414600	-0.21464100
C	-5.14196800	-0.87688500	-3.02257900
H	-4.92728600	0.98672300	-4.08483200
H	-5.18385700	-2.58854200	-1.71571100
H	-5.90873700	-1.26105100	-3.68727400

1a⊂AuCav-10

C	-4.93546900	-3.01922600	-0.47421500
C	-3.89981000	-3.13883900	0.64669200
C	-4.58166700	-1.93278400	-1.48991100
H	-5.85778100	-2.69103500	0.00655300
C	-2.78770900	-3.98687300	0.57301100
C	-4.05899000	-2.40559700	1.83082100
C	-3.67454800	-2.15975100	-2.53235500
C	-5.13784400	-0.64716100	-1.40558300
C	-1.86632500	-4.12541100	1.61956900
H	-2.63708300	-4.56677200	-0.33014300
C	-3.18734300	-2.52335500	2.90677600
O	-5.15521200	-1.54932200	1.92869100
C	-3.30692800	-1.17716500	-3.46061800
H	-3.20607200	-3.13515600	-2.60235400
C	-4.84263200	0.34597100	-2.33358600
O	-5.98234800	-0.33136300	-0.33523400
C	-0.63479300	-5.02472300	1.53077300
C	-2.10190500	-3.38031400	2.78224500
H	-3.33669700	-1.94196800	3.80564800
C	-4.89070000	-0.21389600	1.80568900
C	-2.25168600	-1.42928900	-4.53441500
C	-3.92435900	0.07439900	-3.34166300
H	-5.28273200	1.32981200	-2.23996700
C	-5.37752300	0.44396200	0.62535900
H	-0.35968000	-5.27198400	2.55644400
C	0.54836100	-4.25680400	0.94386100
O	-1.22005600	-3.54893600	3.85340200
C	-0.84540600	-1.26206300	-3.95996300
H	-2.37310100	-0.63864700	-5.27572300
O	-3.60381300	1.08537400	-4.25811400
C	0.74258900	-4.15411600	-0.43510800
C	1.49639600	-3.62721000	1.76340100
C	-0.39874200	-2.51357800	4.18189200
C	-3.98008500	1.74368300	2.52156200
C	-0.09979600	-2.33791600	-3.47783100
C	-0.22880900	-0.00285500	-3.93136100
C	-2.88629500	2.12672600	-3.73232600
C	-4.50687900	2.40952100	1.37444100
C	1.82753800	-3.48712300	-1.00900900
H	0.02656000	-4.63065200	-1.09310600
C	2.57719300	-2.92218500	1.23910900
O	1.38296500	-3.76021600	3.14927700
C	0.99530000	-2.64412200	3.84021900
C	1.21327800	-2.21265900	-3.01805300
H	-0.54379000	-3.32494600	-3.49770500
C	1.06711500	0.18222300	-3.45051500
O	-0.90966400	1.04815900	-4.53010000
C	-1.44914000	2.06247100	-3.79466600
C	1.99944100	-3.40966900	-2.51673000
C	2.71661500	-2.85052100	-0.14067200
H	3.28426900	-2.44185300	1.90002300
C	0.03594700	-0.49315200	5.13712200
C	1.77220800	-0.93114600	-3.00017000
H	1.52057300	1.16539000	-3.47716000
C	-2.73330700	4.10271900	-2.60873000
H	3.05242200	-3.20975800	-2.71901900
O	3.88748400	-2.24969700	-0.63800400
C	1.42328400	-0.64490900	4.83715700
O	3.13022200	-0.74962900	-2.70295700
C	-1.30850000	4.00022500	-2.61177500
C	-4.24139800	3.78448500	1.18441500
C	-3.19099100	2.46599600	3.44520200
C	2.33295500	0.37175700	5.21179900
C	-0.41225800	0.68136300	5.78644400
C	-0.53750900	4.99711300	-1.97155700
C	-3.35355800	5.20716100	-1.98099400
C	1.67148800	-4.72383700	-3.23796200
H	1.83371700	-4.61269000	-4.31324000
H	0.63920400	-5.04904400	-3.09186400
C	-0.90786600	-6.34400700	0.79387400
H	-0.01452900	-6.97390100	0.80703000
H	-1.19539500	-6.20162700	-0.25161200
C	-5.22139400	-4.36761300	-1.15400300
H	-5.52764400	-5.10727600	-0.40963100
H	-4.35365300	-4.77073800	-1.68404100
C	-2.45478600	-2.77240300	-5.25037800
H	-3.44229700	-2.79536000	-5.71776200
H	-2.39470800	-3.63153600	-4.57617400
H	-1.69948600	-2.90638700	-6.02954500
H	-6.02720300	-4.25437700	-1.88372400
H	-1.72035200	-6.88421500	1.28647100
H	2.31948800	-5.52209300	-2.86716900
C	-2.58055700	6.16613700	-1.36027000
C	-1.16924500	6.05647100	-1.35212300
H	-0.57811000	6.83068500	-0.87229700
H	-3.05513900	7.01725800	-0.88216200
H	0.53980200	4.89467400	-1.98343300
H	-4.43603600	5.26841600	-2.01038100
C	-2.94084600	3.80616500	3.23432800
H	-2.78924100	1.93199000	4.29864500
C	-3.46773800	4.46807800	2.09892100
H	-4.64340600	4.26495300	0.30104700
C	0.49245200	1.66418700	6.13158500
H	-1.46956200	0.76576500	6.01318200
C	1.87127200	1.50655300	5.84796600
H	3.38382200	0.21854900	4.99047900
H	2.56957700	2.28178900	6.14852200
H	0.15015300	2.56006500	6.63975500
H	-3.26245200	5.52344100	1.94724200
H	-2.34179200	4.36290000	3.94921900
P	4.21738000	-0.93795700	-1.51175000
N	5.61607800	-1.42899400	-2.25442400
C	5.73473600	-2.77420100	-2.83214300
C	6.43374000	-0.41823600	-2.93294700
H	5.24870300	-3.50124000	-2.18298800
H	6.79629100	-3.02705800	-2.89338700
H	5.30188900	-2.82576100	-3.83928500
H	6.33772700	0.54883000	-2.43070600
H	6.13994300	-0.29561200	-3.98286300
H	7.48293300	-0.72200000	-2.88812800
Au	4.64287100	0.99279400	-0.29599800
N	-0.68565300	2.95461400	-3.23979000
N	-3.50632800	3.12269200	-3.17257000
N	-5.20923400	1.71697200	0.42580500
N	-4.20820900	0.41048900	2.71874500
N	-0.86319800	-1.46942200	4.80266700
N	1.87783700	-1.75103500	4.17263000
C	4.30409000	2.76072900	1.38813000
C	5.32366900	2.88725400	0.71102200
H	6.24037200	3.27969800	0.31688600
C	3.09861500	2.69106700	2.21832800
C	1.82251700	2.28353900	1.41933300
H	2.97956800	3.66663800	2.69697900
H	3.23409100	1.94434600	3.00454300
C	2.05562700	0.79150500	1.00606300
O	2.54467200	0.03866100	1.82210500

O	1.69046300	0.37479200	-0.20150500
H	1.47645900	1.16756300	-0.75835400
C	0.54515700	2.22498800	2.33231200
H	0.82089500	1.67543400	3.23318500
H	0.23052700	3.22195400	2.63738500
C	1.66301000	3.22104100	0.20940600
O	1.71013600	2.80971800	-0.93969200
O	1.61264300	4.54890400	0.36819000
C	1.07513800	5.26985000	1.48993000
H	1.31706100	6.31292600	1.29043000
H	1.51996700	4.97439800	2.44064900
H	-0.00947600	5.14635200	1.52332900
C	-0.52927000	1.50635400	1.55805200
C	-1.22896000	2.17079700	0.54605400
C	-0.64553000	0.11728100	1.67553000
C	-1.99291700	1.44477600	-0.36199400
H	-1.18420000	3.25208000	0.45868900
C	-1.36943300	-0.61070100	0.73821700
H	-0.10105100	-0.39644900	2.45538200
C	-2.02665400	0.05309200	-0.29366800
H	-2.56557000	1.96104600	-1.11752600
H	-1.40044800	-1.69034900	0.79552500
H	-2.56362300	-0.51201900	-1.03756700

(TfO•1a)⊂AuCav-1

C	5.56213400	1.87662700	-1.01615800
C	4.38609900	1.89484300	-1.98931000
C	5.15699200	1.17595500	0.27833900
H	5.76014300	2.91652500	-0.75431200
C	4.14827800	0.82966400	-2.86415500
C	3.49826600	2.97771900	-2.04967400
C	5.32347400	-0.19662100	0.49491400
C	4.57473300	1.91903800	1.31261300
C	3.09860500	0.81233000	-3.78538700
H	4.83194600	-0.01104100	-2.84641300
C	2.40731000	2.98605000	-2.91469100
O	3.77144700	4.12891600	-1.30392300
C	4.94756400	-0.82314000	1.68912100
H	5.77958800	-0.79462600	-0.28571100
C	4.16826700	1.34482200	2.51054800
O	4.50704500	3.29619000	1.14237500
C	2.91910300	-0.32612700	-4.78725800
C	2.21894100	1.90513900	-3.77067300
H	1.74137600	3.83890900	-2.95387900
C	2.94882200	4.43278300	-0.25572500
C	5.12093600	-2.32188900	1.93149800
C	4.36307800	-0.02102200	2.67936700
H	3.73216900	1.94552600	3.29741400
C	3.30493200	3.92719500	1.04317900
H	2.35839800	0.09069900	-5.62455400
C	2.04798100	-1.42312200	-4.18844700
O	1.20201400	1.95303400	-4.71890800
C	3.85175600	-3.03943700	1.48512900
H	5.19219100	-2.45620200	3.01133100
O	4.06339600	-0.61920000	3.89687100
C	2.56107300	-2.41492700	-3.34446200
C	0.66746600	-1.42909200	-4.42626900
C	-0.09217100	1.73674500	-4.31853500
C	1.16518800	5.50435800	0.66841400
C	3.64279800	-3.39478400	0.14731400
C	2.81756200	-3.31588900	2.38959500
C	2.75981700	-0.75511200	4.28338000
C	1.46967300	4.92719800	1.94104200
C	1.74951300	-3.35962200	-2.70782100
H	3.62868800	-2.43352300	-3.15445100
C	-0.17638700	-2.34981700	-3.81870200
O	0.12217400	-0.46064900	-5.26304100
C	-0.67506300	0.46601900	-4.63038200
C	2.45296500	-3.95819500	-0.32354000
H	4.44678300	-3.22752800	-0.55989500
C	1.59776200	-3.82852500	1.95891500
O	3.00726200	-3.08614800	3.75267200
C	2.22982400	-2.08746300	4.29216400
C	2.29295000	-4.43309200	-1.76843900
C	0.37273800	-3.28127200	-2.95010700
H	-1.24646700	-2.29449300	-3.97087700
C	-2.07604600	2.41424800	-3.42715000
C	1.41752300	-4.10894600	0.61142300
H	0.79923000	-3.99244700	2.67103300
C	0.80254300	0.02564200	5.14106800
H	1.53284300	-5.21818400	-1.73973600
O	-0.51423200	-4.06221600	-2.20773800
C	-2.65014300	1.13658500	-3.71827500
O	0.14746500	-4.54181100	0.21865800
C	0.30680800	-1.31204800	5.23011900
C	0.65723100	5.22666100	3.05741400
C	0.08785400	6.41121000	0.55905700
C	-3.98734300	0.87021300	-3.35039100
C	-2.87476500	3.40972300	-2.82290300
C	-0.99656700	-1.54168100	5.72554000
C	-0.02602400	1.10223300	5.53225400
C	3.57973900	-5.07375600	-2.31310400
H	3.91347800	-5.87657500	-1.65037900
H	4.40182300	-4.35929000	-2.40817600
C	4.25135900	-0.85681700	-5.33609500
H	4.06308500	-1.61296600	-6.10309700
H	4.87808300	-1.31900800	-4.56797700
C	6.84462800	1.30324900	-1.63428200
H	7.11602100	1.87191500	-2.52760600
H	6.74910300	0.25474700	-1.93020800
C	6.39679600	-2.89670900	1.30252500
H	7.27453000	-2.36819900	1.68409500
H	6.41338300	-2.81606000	0.21192300
H	6.49284100	-3.95634600	1.55401900
H	7.66729700	1.36806500	-0.91692100
H	4.82667400	-0.04225400	-5.78430000
H	3.39393400	-5.49268000	-3.30547000
C	-1.30319700	0.85193400	5.98592000
C	-1.78909000	-0.47422600	6.08500200
H	-2.80288100	-0.64618800	6.42980400
H	-1.94963200	1.67819600	6.26308500
H	-1.35692500	-2.56241100	5.76534600
H	0.36878100	2.10894600	5.44818700
C	-0.65710500	6.72607300	1.67597600
H	-0.11383000	6.84951800	-0.41215000
C	-0.38673300	6.11631700	2.92344300
H	0.89723800	4.75843200	4.00557200
C	-4.18667000	3.13197900	-2.50341700
H	-2.42087100	4.37318700	-2.62297000
C	-4.74196700	1.85474400	-2.75067200
H	-4.38713500	-0.12120900	-3.52617300
H	-5.75545700	1.63798700	-2.43545300
H	-4.80255800	3.89433000	-2.03780800
H	-1.00812300	6.35418900	3.77999900
H	-1.46938500	7.44148600	1.59868400
P	-0.77188900	-3.48733100	-0.66127100
N	-2.28867800	-3.89952800	-0.22580200
C	-3.38377000	-2.96991300	-0.55775600
C	-2.69619900	-5.25631900	0.13391100
H	-3.00866900	-1.95562200	-0.69851300
H	-4.10308600	-2.94561200	0.26146000
H	-3.89730600	-3.28422700	-1.47337800
H	-1.83060900	-5.84434700	0.43858300
H	-3.19552300	-5.75343200	-0.70637300
H	-3.39707600	-5.19432000	0.97216100
Au	-0.07241600	-1.27962500	-0.51398300
N	1.05421000	-2.36378700	4.77096400
N	2.06550000	0.27271200	4.67317900
N	2.57198000	4.12852400	2.09779400
N	1.91530000	5.20047400	-0.43764900
N	-0.76636400	2.68005000	-3.72789500
N	-1.90385500	0.16873600	-4.33020700
C	0.85720100	0.89232600	-0.20846400
C	1.75931200	0.06375800	-0.33327700
H	2.70166600	-0.44012500	-0.39147200
C	0.00034700	2.06733100	0.00566500
C	-1.26327300	1.81508300	0.87896500
H	-0.28225400	2.49522800	-0.95963300
H	0.63012800	2.80309100	0.51457800
C	-2.31695600	1.06956000	0.03448500

O	-2.00272400	0.23283200	-0.81625100
O	-3.53809500	1.40288300	0.31254200
H	-4.22581100	0.70754100	-0.09096200
C	-0.90550600	1.03190700	2.19080900
H	0.14580000	0.74001700	2.14631000
H	-1.00852100	1.74203700	3.01182400
C	-1.92736400	3.15119900	1.25774700
O	-2.28020700	3.46009000	2.37273400
O	-2.12527100	3.90862100	0.16474400
C	-3.10264600	4.95329300	0.30873900
H	-4.09738800	4.50170700	0.29054800
H	-2.95645000	5.48927600	1.24395500
H	-2.96513000	5.61611600	-0.54443900
C	-1.73851600	-0.19490000	2.48399800
C	-1.15605300	-1.46606600	2.44378500
C	-3.09764200	-0.08927700	2.81067900
C	-1.91322900	-2.61100300	2.69931300
H	-0.09218600	-1.55887100	2.24934800
C	-3.86055500	-1.23027900	3.05188100
H	-3.56837300	0.88606100	2.84779000
C	-3.27009600	-2.49501200	2.99308200
H	-1.44440100	-3.58850800	2.66224800
H	-4.92048300	-1.12666600	3.25108100
H	-3.87145600	-3.38104600	3.17217600
S	-6.21342900	-0.68100900	0.43629200
O	-5.09673300	-0.32574300	-0.52903200
O	-6.34631100	0.27571200	1.54497800
O	-6.26742400	-2.11513100	0.75618600
C	-7.69037200	-0.38169000	-0.64465100
F	-7.70925000	0.90048400	-1.06358000
F	-7.65208500	-1.17151800	-1.72641300
F	-8.82302500	-0.62078000	0.02434400

(TfO•1a)⊂AuCav-2

C	3.37685900	-2.48486700	-3.67408300
C	2.09243900	-1.69527300	-3.90450400
C	3.66128300	-2.58555100	-2.17938600
H	4.18722400	-1.89443400	-4.10215800
C	0.83537900	-2.28498000	-4.05018100
C	2.15958100	-0.29995700	-3.98461200
C	3.07360600	-3.59292400	-1.41059400
C	4.52049600	-1.69880300	-1.51385200
C	-0.32224200	-1.53801900	-4.29236300
H	0.75667900	-3.36490900	-4.00628300
C	1.03851100	0.49395100	-4.19460000
O	3.43928000	0.24087300	-3.99147800
C	3.33327900	-3.78719800	-0.05097000
H	2.40383200	-4.28531600	-1.90603900
C	4.77058300	-1.81901500	-0.14669700
O	5.25453200	-0.76638400	-2.24922300
C	-1.68787700	-2.19184800	-4.49266600
C	-0.19281700	-0.14159500	-4.34709600
H	1.12073700	1.56911300	-4.28751800
C	3.91615100	1.08316800	-3.04271800
C	2.71669600	-4.97249300	0.68796400
C	4.18686000	-2.86216100	0.57264000
H	5.46590800	-1.14757300	0.34262300
C	4.94710100	0.56611800	-2.17808000
H	-2.27607800	-1.50166800	-5.09776400
C	-2.38900700	-2.29605300	-3.14395700
O	-1.31117700	0.59825800	-4.70057200
C	1.31218100	-4.60949100	1.15558500
H	3.31701000	-5.12103900	1.58581400
O	4.55965700	-3.04344100	1.90843400
C	-2.11713400	-3.33670300	-2.25128300
C	-3.27763100	-1.29622600	-2.72520600
C	-1.93534800	1.41527600	-3.79213100
C	4.26311400	3.17018400	-2.20921900
C	0.15776900	-4.79265200	0.38652000
C	1.16224000	-4.00423600	2.40839200
C	4.00123800	-2.20414600	2.84242700
C	5.36228600	2.68097700	-1.44155000
C	-2.65481200	-3.39130900	-0.96223400
H	-1.44629100	-4.12767100	-2.56658400
C	-3.81091900	-1.28439900	-1.43929700
O	-3.62306600	-0.29234200	-3.62331900
C	-3.19990200	0.97367700	-3.28205900
C	-1.10764000	-4.36705500	0.81399500
H	0.24474800	-5.27309600	-0.58139800
C	-0.06649800	-3.56998300	2.87843600
O	2.30610100	-3.85043800	3.17884100
C	2.75125800	-2.58575200	3.43647400
C	-2.37880100	-4.55754300	-0.01244300
C	-3.47162800	-2.31775700	-0.57618800
H	-4.44675800	-0.47283600	-1.10852700
C	-2.17143300	3.37002100	-2.65697400
C	-1.17944600	-3.73772600	2.06409000
H	-0.14906400	-3.07457000	3.83613900
C	3.99024900	-0.30341200	4.09891100
H	-3.20305300	-4.56251700	0.70539800
O	-3.92751900	-2.22297000	0.74845500
C	-3.44929100	2.94148200	-2.17937600
O	-2.37130900	-3.18139800	2.51354900
C	2.67139100	-0.60726000	4.55577400
C	6.15665100	3.59244200	-0.71178400
C	3.95663600	4.54824100	-2.19047200
C	-4.17831000	3.75894100	-1.28901700
C	-1.66294900	4.62098100	-2.24282800
C	1.98588000	0.31115500	5.38332500
C	4.62395400	0.87371500	4.55525700
C	-2.40437900	-5.91201500	-0.73761200
H	-2.28026200	-6.72591000	-0.01818300
H	-1.61247100	-6.00823500	-1.48528400
C	-1.61904100	-3.52933800	-5.24180400
H	-2.62901200	-3.91079700	-5.41456900
H	-1.06774900	-4.30086600	-4.69637200
C	3.38440800	-3.85086000	-4.37431900
H	3.23448000	-3.71850000	-5.44921200
H	2.60201600	-4.52498800	-4.01486200
C	2.76684300	-6.27381900	-0.12493700
H	3.79830200	-6.50333100	-0.40505800
H	2.17844600	-6.22933200	-1.04553000
H	2.37688000	-7.10119600	0.47422600
H	4.34477500	-4.34864000	-4.21553300
H	-1.12802300	-3.39572700	-6.20968900
H	-3.35948600	-6.04236800	-1.25274000
C	3.94209800	1.74739700	5.37323300
C	2.61249600	1.47516100	5.77348300
H	2.08873800	2.18624700	6.40420000
H	4.41981800	2.66514000	5.69812800
H	0.98002400	0.06266200	5.70440600
H	5.63175500	1.07670700	4.21609100
C	4.73643200	5.41424800	-1.45407200
H	3.12033900	4.88552000	-2.79087800
C	5.84689100	4.93542400	-0.72047300
H	6.99952800	3.20168400	-0.15307900
C	-2.38613200	5.39330300	-1.35611100
H	-0.69857100	4.93039500	-2.62768300
C	-3.64127600	4.95670400	-0.87001900
H	-5.12727500	3.40734200	-0.90923400
H	-4.18471600	5.55756400	-0.14913700
H	-1.99203600	6.34818200	-1.02247900
H	6.45607700	5.63465100	-0.15690100
H	4.51012800	6.47581300	-1.44797200
P	-2.80342100	-1.71882500	1.83199100
N	-3.64891400	-0.92607900	2.96895800
C	-4.93633900	-1.42829100	3.47105700
C	-3.01870100	0.13926300	3.75455100
H	-5.38718500	-2.09278500	2.73458600
H	-5.59383900	-0.56984400	3.61725800
H	-4.79474700	-1.97002300	4.41395000
H	-2.68270500	-0.23844700	4.72890300
H	-3.75252500	0.93510800	3.89013500
H	-2.16350200	0.54992000	3.21424200
Au	-0.95149200	-0.83984100	0.73012400
N	2.08398700	-1.79693700	4.22522500
N	4.61802800	-1.11290800	3.18847200
N	5.65433800	1.34240400	-1.41168300
N	3.55165700	2.33127500	-3.02374300
N	-1.42931400	2.57396700	-3.48902700

N	-3.94307800	1.71513700	-2.52300200
C	0.93837700	0.40435000	-0.46703700
C	1.02507800	-0.81940100	-0.37533500
H	1.41220600	-1.81504200	-0.48382900
C	0.94119100	1.86133600	-0.59290300
C	0.23778100	2.59195000	0.57913000
H	0.47157900	2.13670300	-1.53948900
H	1.98779000	2.17486200	-0.63659700
C	-1.26627200	2.24109100	0.50943900
O	-1.68549300	1.51768000	-0.38989100
O	-1.98367400	2.71871800	1.48785200
H	-2.95402900	2.36806100	1.38709500
C	0.81057300	2.17964400	1.97455500
H	0.67590600	1.10034000	2.10214000
H	0.19884600	2.67960200	2.72534800
C	0.39094700	4.11708100	0.48805400
O	-0.00968900	4.87412300	1.34130800
O	1.04652000	4.51850900	-0.61629000
C	1.24913400	5.94078400	-0.69964700
H	1.54877200	6.14070600	-1.72638900
H	0.32893300	6.46896800	-0.45066200
H	2.03865800	6.23906900	-0.00631400
C	2.26477000	2.55567800	2.13096800
C	2.61535400	3.77754500	2.72194400
C	3.28587100	1.72914100	1.64436800
C	3.95098100	4.17018200	2.80591000
H	1.83125500	4.42493500	3.09979000
C	4.62025500	2.12074100	1.72627100
H	3.04069600	0.76263300	1.21444800
C	4.95572100	3.34515400	2.30288300
H	4.20413900	5.12078800	3.26641700
H	5.39248100	1.46256500	1.34403700
H	5.99543400	3.65016500	2.36697400
S	-5.57293600	2.04755800	1.72546000
O	-5.94323200	3.41731900	1.34144500
O	-5.86582000	1.63536800	3.11205900
O	-4.21140700	1.61594600	1.24109000
C	-6.63484600	0.94726000	0.67694000
F	-7.92760500	1.11101500	0.97610200
F	-6.45614900	1.22072000	-0.62532200
F	-6.31339800	-0.34655900	0.87375400

(TfO•1a)⊂AuCav-3

C	-2.96015100	4.19712500	2.85595100
C	-3.05087600	2.71196000	3.19382900
C	-2.24124900	4.42804100	1.53112700
H	-2.32773300	4.64221600	3.62473600
C	-4.13649800	1.92205300	2.81078800
C	-2.04397100	2.09415700	3.95074100
C	-2.89539100	4.33191600	0.29905600
C	-0.87509500	4.74234800	1.50875200
C	-4.26872700	0.58118900	3.18369500
H	-4.92660600	2.37749100	2.22535400
C	-2.12030700	0.75740600	4.33025200
O	-1.00094800	2.88390900	4.41601100
C	-2.24246400	4.53561900	-0.92113200
H	-3.94766200	4.06794700	0.28939100
C	-0.19292400	4.98288400	0.32132500
O	-0.18502900	4.82769800	2.71504300
C	-5.47688100	-0.25469100	2.77558200
C	-3.23968800	0.02392400	3.95224400
H	-1.33692600	0.31071100	4.92856200
C	0.23978400	2.74438000	3.84522300
C	-2.93684400	4.36908600	-2.26533700
C	-0.88524100	4.86689400	-0.87879600
H	0.86370200	5.21524800	0.33279800
C	0.68229200	3.78764700	2.96972300
H	-5.54208000	-1.07586200	3.48973100
C	-5.22443300	-0.88670800	1.41214600
O	-3.40311200	-1.28355600	4.42871700
C	-3.02677600	2.89558000	-2.64449800
H	-2.28648100	4.83302500	-3.00752100
O	-0.21432400	5.06008000	-2.09028200
C	-5.49337600	-0.21658600	0.21517200
C	-4.67042200	-2.17054800	1.31929000
C	-2.59865600	-2.26156100	3.92327000
C	2.23094900	1.67664100	3.58132500
C	-4.14778400	2.12813100	-2.31754800
C	-1.99838300	2.26713300	-3.36921600
C	0.71492800	4.08002200	-2.34519300
C	2.67692100	2.72203100	2.71193700
C	-5.25373900	-0.77691800	-1.04376500
H	-5.90597700	0.78363300	0.26624500
C	-4.40801400	-2.77016900	0.09205000
O	-4.42500300	-2.87274600	2.49072500
C	-3.12161000	-3.07903800	2.86231000
C	-4.31935700	0.80556100	-2.73254100
H	-4.93557600	2.59287400	-1.73838700
C	-2.13952300	0.95779700	-3.82889800
O	-0.89678800	3.00985200	-3.76350000
C	0.32386000	2.94435300	-3.12674500
C	-5.54021100	-0.02147700	-2.34019100
C	-4.70731600	-2.06440100	-1.06430000
H	-3.98666800	-3.76634900	0.05158500
C	-0.67396300	-3.47882200	3.89144500
C	-3.30251400	0.25940500	-3.52066200
H	-1.36877100	0.51571800	-4.44796900
C	2.77540500	3.16299900	-2.01834400
H	-5.67968000	-0.77281400	-3.12100500
O	-4.47116300	-2.70457000	-2.29487600
C	-1.16805600	-4.24348300	2.78929600
O	-3.47649600	-1.03127400	-4.06392100
C	2.38464900	2.00854600	-2.77083200
C	3.96125600	2.64171100	2.12810900
C	3.09042000	0.58665100	3.85050500
C	-0.35396000	-5.24968900	2.21896400
C	0.60508200	-3.76680900	4.42076100
C	3.31292700	0.95395400	-2.92962400
C	4.06207100	3.22754200	-1.44040500
C	-6.83538700	0.80009300	-2.27845900
H	-7.01722100	1.28416200	-3.24146200
H	-6.81108900	1.58215800	-1.51561500
C	-6.80028400	0.52094300	2.83697900
H	-7.63593500	-0.13915200	2.58808200
H	-6.83411200	1.36969900	2.14827900
C	-4.32249600	4.90508300	2.90821000
H	-4.78955200	4.75439500	3.88539700
H	-5.02196200	4.54474100	2.14807200
C	-4.29263400	5.08889500	-2.31797400
H	-4.14480400	6.15844800	-2.14835000
H	-4.99494300	4.73500400	-1.55776400
H	-4.76193000	4.95383600	-3.29666200
H	-4.19042500	5.97832000	2.74698000
H	-6.95481000	0.91226700	3.84577300
H	-7.68108500	0.14602500	-2.05129000
C	4.93697000	2.17421800	-1.58901200
C	4.55868200	1.03600800	-2.34221600
H	5.25512800	0.21165100	-2.42388000
H	5.91145800	2.19842300	-1.11537900
H	3.03654300	0.08075700	-3.50844500
H	4.31972400	4.11790800	-0.87762900
C	4.33561900	0.53242100	3.25996300
H	2.73241300	-0.19410200	4.51325300
C	4.77623800	1.56099400	2.39091500
H	4.27434100	3.44008700	1.46634300
C	1.37131200	-4.76619800	3.86110100
H	0.95841200	-3.17086600	5.25472100
C	0.89308200	-5.50272600	2.75102400
H	-0.74847300	-5.81684000	1.38216500
H	1.51890100	-6.27539100	2.31631000
H	2.36475100	-4.96685400	4.24246300
H	5.75269100	1.49611200	1.92028400
H	4.98484600	-0.31747200	3.44148700
P	-3.13072200	-2.32279600	-3.13260400
N	-3.15007700	-3.53610000	-4.27899700
C	-3.20832500	-4.91593000	-3.77717000
C	-2.23499000	-3.36369900	-5.41726500
H	-3.95087100	-4.99413100	-2.98374700
H	-2.23382600	-5.26436300	-3.40401700
H	-3.51544600	-5.56543400	-4.60089500

H	-2.29168500	-2.34134900	-5.78982100
H	-2.55471800	-4.03827200	-6.21556500
H	-1.19380200	-3.59880800	-5.15341200
Au	-1.18433300	-1.85159800	-2.03535100
N	1.12620900	1.93305300	-3.31957500
N	1.89756300	4.18602000	-1.81752100
N	1.85964500	3.77919300	2.41989600
N	0.98386200	1.72019800	4.14134100
N	-1.41727400	-2.46358900	4.42745800
N	-2.42493400	-4.01811000	2.29319000
C	1.15925000	-2.05300600	-1.50194400
C	0.88555200	-1.08481600	-2.20948000
H	0.90530000	-0.17650500	-2.78904600
C	1.60560400	-3.14094900	-0.62744200
C	2.59284100	-2.62527900	0.45066500
H	2.09942700	-3.89200500	-1.24538900
H	0.74827000	-3.61058300	-0.13476900
C	3.72377600	-1.84279700	-0.27622600
O	3.84070500	-1.90035800	-1.48837800
O	4.51164200	-1.21418900	0.56038400
H	5.37271400	-0.88764400	0.08252100
C	1.88993300	-1.82551800	1.57766900
H	2.67021800	-1.37380300	2.18779300
H	1.37126400	-2.54605500	2.21054100
C	3.34169100	-3.79297300	1.11426000
O	3.69032900	-3.81300900	2.27235100
O	3.62055800	-4.77556900	0.23559100
C	4.46333100	-5.82759700	0.74013500
H	4.62050900	-6.50394500	-0.09873400
H	5.41188400	-5.41428500	1.08831300
H	3.97453000	-6.34537900	1.56914600
C	0.91104800	-0.77704600	1.11244200
C	-0.46593900	-1.04135800	1.10659300
C	1.35445600	0.47311000	0.66391600
C	-1.38318000	-0.09253100	0.64033600
H	-0.82310900	-1.99814300	1.47008800
C	0.44455800	1.42571600	0.20745200
H	2.41601900	0.69802400	0.66929400
C	-0.92526300	1.14702200	0.18398600
H	-2.44733100	-0.30388000	0.66867000
H	0.80561100	2.39523800	-0.11055000
H	-1.62915700	1.89606200	-0.15971300
S	7.78265900	0.17170800	-0.01144600
O	8.68075700	-0.62086000	0.83269500
O	6.63574400	-0.60324200	-0.61573300
O	7.37592100	1.50215800	0.49644300
C	8.74390600	0.56545400	-1.54616400
F	9.17851300	-0.55271600	-2.13988700
F	7.96789800	1.23385000	-2.42180500
F	9.80049200	1.33239400	-1.25141600

(TfO•1a)⊂AuCav-4

C	-4.47313800	-0.76275000	3.78145600
C	-2.96661700	-0.69029300	4.02113400
C	-4.81524200	-0.68321100	2.29651500
H	-4.89508300	0.13330800	4.23755500
C	-2.14529000	-1.82143100	4.06231900
C	-2.35431400	0.54920900	4.24661000
C	-4.82090700	-1.82235200	1.48406000
C	-5.16414900	0.53274000	1.69246200
C	-0.77798900	-1.75345300	4.35150100
H	-2.58940200	-2.79302700	3.87960200
C	-1.00176500	0.67337100	4.54281700
O	-3.17613200	1.67842900	4.25379200
C	-5.16748000	-1.79470600	0.13126200
H	-4.55161600	-2.77296300	1.92929200
C	-5.50484800	0.61568000	0.34417600
O	-5.23034700	1.69171700	2.48185100
C	0.12812800	-2.98063600	4.36936600
C	-0.23463100	-0.48675500	4.60382400
H	-0.55926100	1.64083400	4.73894100
C	-3.09841000	2.54858300	3.20981000
C	-5.16696300	-3.04852500	-0.74123700
C	-5.49611600	-0.54838900	-0.41853900
H	-5.76433400	1.56605100	-0.10320500
C	-4.22328300	2.60214900	2.31040200
H	0.98438000	-2.72697700	4.99423800
C	0.66803900	-3.21871600	2.96278500
O	1.09602600	-0.42329400	5.00575700
C	-3.76912700	-3.26265900	-1.30902800
H	-5.81995500	-2.83819700	-1.58842700
O	-5.87647600	-0.51333300	-1.76073000
C	-0.07038900	-3.93207400	2.01537400
C	1.90546700	-2.69481200	2.55632000
C	2.00362300	0.17802000	4.17661000
C	-2.08889100	4.22341100	2.04609800
C	-2.77276200	-3.93073900	-0.59076800
C	-3.41489600	-2.75448200	-2.56588900
C	-5.01962500	0.06230700	-2.65930800
C	-3.23594000	4.33101500	1.20170300
C	0.37327100	-4.13236600	0.70762700
H	-1.02897900	-4.34454100	2.30732100
C	2.38538700	-2.85811800	1.25828600
O	2.68506400	-2.02770900	3.50203700
C	2.84420500	-0.67072900	3.37458000
C	-1.45452900	-4.05427700	-1.03890900
H	-3.03761400	-4.38426000	0.35691100
C	-2.10737200	-2.83185000	-3.03805300
O	-4.40587000	-2.16657600	-3.36256200
C	-4.30009500	-0.80929200	-3.54899300
C	-0.42965500	-4.92010900	-0.31360600
C	1.60268600	-3.56834700	0.35280400
H	3.32614400	-2.42234500	0.94516600
C	3.08592700	2.00994600	3.37600900
C	-1.12076300	-3.41093300	-2.24021700
H	-1.86221700	-2.43243800	-4.01405000
C	-4.03937000	1.86685100	-3.64002100
H	0.28114400	-5.24478600	-1.07749100
O	2.05135200	-3.70320300	-0.96654900
C	3.91540000	1.16949400	2.56786900
O	0.17789600	-3.40734200	-2.72880100
C	-3.39471400	1.00828400	-4.58184800
C	-3.24351600	5.28460000	0.15773600
C	-0.96614000	5.04989800	1.80749500
C	4.89927200	1.75468800	1.73880800
C	3.27655000	3.41014700	3.35650900
C	-2.53540100	1.56358100	-5.55668900
C	-3.77609900	3.25466400	-3.66850200
C	-1.05284500	-6.19112100	0.28467200
H	-1.57718500	-6.75540700	-0.49113800
H	-1.77021900	-5.98080000	1.08231200
C	-0.54165400	-4.22075600	4.97665800
H	0.16646700	-5.05336400	5.00256100
H	-1.41961200	-4.55430200	4.41627100
C	-5.12059800	-1.97527800	4.46801600
H	-4.90616900	-1.95724400	5.54002500
H	-4.76229000	-2.93160700	4.07703700
C	-5.72334700	-4.28260000	-0.01771100
H	-6.74163900	-4.08674300	0.32897200
H	-5.13046500	-4.57325300	0.85405000
H	-5.74618800	-5.13724600	-0.69930700
H	-6.20467900	-1.94925500	4.32860800
H	-0.86591200	-4.00826400	5.99911200
H	-0.26846900	-6.82388600	0.70831900
C	-2.91420900	3.76850100	-4.61364100
C	-2.29918600	2.92182900	-5.56686200
H	-1.62694400	3.34843800	-6.30448000
H	-2.70178400	4.83284100	-4.63117200
H	-2.05912300	0.89156600	-6.26230600
H	-4.25727400	3.87980600	-2.92447700
C	-0.98910700	5.95477100	0.76522700
H	-0.10173000	4.93946700	2.45374500
C	-2.13558500	6.07930500	-0.05589000
H	-4.12622800	5.34675600	-0.46924500
C	4.25679100	3.95402200	2.55184200
H	2.64011100	4.02115800	3.98778400
C	5.06167700	3.12500500	1.73665300
H	5.49808100	1.11202600	1.10838600
H	5.80600700	3.57157700	1.08633200
H	4.40921500	5.02842400	2.53671200

H	-2.13539400	6.80036000	-0.86698900
H	-0.11821400	6.57138200	0.56927100
P	1.49596600	-2.60346000	-2.05978700
N	2.60557400	-2.67686200	-3.24765800
C	3.34187900	-3.89278000	-3.59447300
C	2.79138700	-1.53408400	-4.14469800
H	3.16793100	-4.66433800	-2.84489800
H	4.40710400	-3.64959000	-3.60628400
H	3.02559500	-4.26747700	-4.57557600
H	2.31165000	-1.71808800	-5.11485300
H	3.86291100	-1.37411000	-4.27567700
H	2.38176600	-0.62533300	-3.70073700
Au	1.17356400	-0.57424100	-0.97467900
N	-3.54404800	-0.34981500	-4.50162600
N	-4.88825600	1.35453900	-2.69901700
N	-4.29761200	3.47903800	1.35253600
N	-2.05903900	3.31742900	3.07009100
N	2.11514400	1.47228000	4.17632700
N	3.76454300	-0.19151900	2.59534400
C	1.09364500	1.44164100	0.48749800
C	0.63183400	0.41174000	0.97524700
H	0.15731600	-0.30589300	1.61241300
C	1.59994800	2.80162800	0.25557600
C	1.90437500	3.27710800	-1.19122800
H	2.52046400	2.90104200	0.82945700
H	0.86679600	3.47912200	0.70226400
C	2.89665200	2.27056800	-1.81163600
O	2.60128800	1.49497600	-2.70581100
O	4.05489600	2.34188500	-1.19505300
H	4.70023600	1.58789900	-1.43177500
C	0.63693700	3.51219000	-2.07475400
H	0.97812800	3.62017600	-3.10592800
H	0.20023900	4.46848400	-1.76703500
C	2.61219800	4.63515500	-1.16330400
O	3.13153900	5.12949000	-2.13720100
O	2.52342100	5.25760000	0.03189900
C	3.16790300	6.54136500	0.09537400
H	3.00238000	6.90218200	1.11007200
H	4.23566500	6.43818700	-0.10936300
H	2.73290300	7.22524300	-0.63688200
C	-0.40312400	2.43761700	-1.94232000
C	-1.34882700	2.51045300	-0.91541800
C	-0.38719900	1.30763900	-2.76339100
C	-2.18181300	1.43288700	-0.62755600
H	-1.39144500	3.40474700	-0.31215200
C	-1.22070300	0.22841700	-2.47629600
H	0.31432300	1.25383300	-3.58565900
C	-2.08814600	0.26707300	-1.38201700
H	-2.88928600	1.49235500	0.18808100
H	-1.18602000	-0.64424800	-3.10865600
H	-2.69060600	-0.59748100	-1.13161200
S	5.26813500	-0.91415100	-1.39303100
O	5.47483500	-1.73533000	-2.59490700
O	3.93160300	-0.98009200	-0.75275100
O	5.79032100	0.48927400	-1.49004800
C	6.37571300	-1.70250700	-0.12799000
F	5.88439700	-2.91104200	0.19498000
F	7.61378300	-1.85621200	-0.60684700
F	6.44014400	-0.96636400	0.99212900

(TfO•1a)⊂AuCav-5

C	4.75574000	-1.95381800	-2.47311600
C	3.34029500	-1.86145300	-3.03580700
C	4.75585400	-1.52373300	-1.00983300
H	5.35516800	-1.21874600	-3.01079700
C	2.46082900	-2.94173000	-3.11674800
C	2.88478300	-0.62682100	-3.51223800
C	4.41326300	-2.43618700	-0.00808700
C	5.09666200	-0.22673100	-0.59743000
C	1.18227900	-2.83045300	-3.67450500
H	2.79148100	-3.91219100	-2.76619100
C	1.61784700	-0.45149200	-4.05477000
O	3.84285500	0.37458200	-3.55184400
C	4.43607000	-2.14070700	1.35764200
H	4.14589200	-3.44244700	-0.30772900
C	5.07625800	0.13274800	0.75089800
O	5.59725500	0.69341100	-1.52226500
C	0.24169200	-4.02882600	-3.78893200
C	0.78097300	-1.56449500	-4.13135000
H	1.30351700	0.50460400	-4.45263200
C	3.79187000	1.55090100	-2.88493800
C	4.14890400	-3.23121700	2.38719400
C	4.75533900	-0.82090800	1.71710200
H	5.37138100	1.13010700	1.05449700
C	4.82033500	1.75843100	-1.89649200
H	-0.41770100	-3.82612000	-4.63322000
C	-0.64146900	-4.07882900	-2.54785700
O	-0.42969800	-1.46873900	-4.80231600
C	2.64473300	-3.37295700	2.58737300
H	4.55773300	-2.87497100	3.33304200
O	4.86679600	-0.47080700	3.06618400
C	-0.19673800	-4.62926100	-1.34074800
C	-1.90860300	-3.48135000	-2.56101800
C	-1.49756100	-0.80468600	-4.25234600
C	3.18230300	3.73279500	-2.70824000
C	1.82990200	-4.21812500	1.82754700
C	2.02161500	-2.57656500	3.55488200
C	3.85125600	0.28087500	3.60837400
C	4.30280100	3.97463800	-1.85797200
C	-0.93500000	-4.55694600	-0.15488900
H	0.77361200	-5.11241200	-1.31836100
C	-2.66564800	-3.35361600	-1.40096400
O	-2.40259400	-2.97616600	-3.75780000
C	-2.57737900	-1.60830700	-3.76512700
C	0.43712100	-4.26414100	1.98219400
H	2.29020200	-4.85573900	1.08150500
C	0.65020500	-2.58888600	3.75178300
O	2.83786500	-1.75924500	4.32320200
C	2.72694600	-0.40583800	4.17839400
C	-0.45890000	-5.18761100	1.15530600
C	-2.15508900	-3.86882200	-0.22033400
H	-3.60621000	-2.81638300	-1.41175200
C	-2.68794200	1.06958800	-3.75892100
C	-0.12453600	-3.41660900	2.94833500
H	0.19066300	-1.93546300	4.48077200
C	2.87658100	2.27030300	4.14146300
H	-1.35829300	-5.34603600	1.75607000
O	-2.87891800	-3.60547700	0.95403600
C	-3.77080500	0.26312400	-3.28617600
O	-1.50611200	-3.29828200	3.08085500
C	1.69266600	1.58532500	4.55375300
C	4.59582800	5.29648500	-1.45765400
C	2.34492500	4.80597800	-3.08098500
C	-4.90464700	0.87268800	-2.70665300
C	-2.78622300	2.47594400	-3.67016900
C	0.55896700	2.32545100	4.96049100
C	2.92421100	3.67955400	4.22779100
C	0.18023000	-6.56693900	0.92983800
H	0.44242600	-7.02153000	1.88891700
H	1.09075000	-6.51858900	0.32654300
C	0.96605600	-5.35260200	-4.06664800
H	0.23458100	-6.15171300	-4.21387400
H	1.62620400	-5.66152400	-3.25088100
C	5.40946300	-3.32564200	-2.68862800
H	5.43216200	-3.56412200	-3.75543700
H	4.88365200	-4.13924400	-2.18133500
C	4.84198200	-4.55977400	2.05233700
H	5.92033900	-4.40805300	1.95695000
H	4.48905800	-5.00543000	1.11824400
H	4.66204600	-5.28459500	2.85100100
H	6.43602700	-3.31442800	-2.31288700
H	1.57534300	-5.26903100	-4.97080100
H	-0.52247300	-7.22399200	0.41114400
C	1.81254200	4.37920700	4.64210500
C	0.62029400	3.70192600	4.99216100
H	-0.24878800	4.27501500	5.29874500
H	1.84138800	5.46253500	4.68056700
H	-0.33213000	1.78178800	5.25540900
H	3.83626000	4.18012400	3.92842600
C	2.63791600	6.08423400	-2.65524300

H	1.50306000	4.58541300	-3.72540900
C	3.77681300	6.33220200	-1.85431200
H	5.46428100	5.45915700	-0.82868500
C	-3.89977400	3.04748300	-3.08746200
H	-1.96045300	3.06890900	-4.04487000
C	-4.95547900	2.24500800	-2.59273200
H	-5.69484000	0.25299800	-2.30240200
H	-5.80299300	2.70106600	-2.09501500
H	-3.96901300	4.12679600	-2.99552200
H	3.99971900	7.34781700	-1.54344200
H	2.00208600	6.91283300	-2.95069200
P	-2.25995600	-2.40839100	1.88470700
N	-3.57490500	-1.69784800	2.52255500
C	-4.86656400	-2.37537900	2.71819000
C	-3.42107200	-0.40708100	3.19934200
H	-4.92334600	-3.25069900	2.07293700
H	-5.66436100	-1.68117100	2.44435000
H	-4.97702100	-2.68692600	3.76372000
H	-2.50081600	0.08400900	2.87105200
H	-3.38501600	-0.53652700	4.28798000
H	-4.26093600	0.23087900	2.92567200
Au	-0.63314000	-1.19767600	0.73747200
N	1.66390700	0.21875400	4.59042300
N	3.93765800	1.57819200	3.61936100
N	5.08252200	2.93694500	-1.41686800
N	2.96030300	2.48728200	-3.23365300
N	-1.54581800	0.49539300	-4.25257100
N	-3.68088300	-1.09947900	-3.31329100
C	0.74030700	0.38316400	-0.60507900
C	1.33604600	-0.59978400	-0.16374800
H	2.11986000	-1.30174900	0.04975800
C	0.16118300	1.60399700	-1.16420700
C	-0.93319000	2.25592100	-0.28287100
H	-0.23942400	1.38277100	-2.15592400
H	0.98797500	2.30750500	-1.29379100
C	-2.16925400	1.32728800	-0.27461200
O	-2.17559700	0.29039100	-0.93260200
O	-3.12618900	1.72585000	0.51343600
H	-3.93759400	1.08408100	0.45891900
C	-0.45604100	2.49981600	1.18717800
H	-0.17356200	1.53730400	1.62633200
H	-1.31861900	2.87341900	1.73893200
C	-1.35484300	3.63396300	-0.81809600
O	-2.18577500	4.32889500	-0.28227400
O	-0.66213600	4.01091100	-1.91006000
C	-0.97173400	5.33485100	-2.38085700
H	-0.55196100	5.40325200	-3.38273500
H	-2.05058100	5.48915600	-2.39921700
H	-0.51019800	6.07334900	-1.72119100
C	0.69283400	3.47563700	1.26422600
C	0.44191300	4.84392900	1.43807500
C	2.01863100	3.05018200	1.11647200
C	1.48975800	5.76383400	1.44567700
H	-0.58225300	5.18226900	1.55442900
C	3.06720400	3.96756100	1.13008000
H	2.23750700	1.99164000	1.01319700
C	2.80461100	5.32757800	1.28930600
H	1.27734600	6.82041000	1.58128400
H	4.08690700	3.61453600	1.02649400
H	3.62147600	6.04143500	1.29833900
S	-6.52448900	0.55997000	0.69144800
O	-5.07729300	0.15350100	0.52850000
O	-7.20034600	-0.20119200	1.75414900
O	-7.23929000	0.75371900	-0.57855400
C	-6.35859400	2.27527600	1.38615900
F	-5.61513500	2.25520000	2.51220300
F	-5.74558700	3.08332600	0.50408400
F	-7.55448700	2.79197600	1.67986500

(TfO•1a)⊂AuCav-6

C	-3.99726700	3.43495300	2.57563300
C	-3.92709100	1.92872500	2.80912700
C	-3.09433400	3.89584100	1.43595500
H	-3.59639400	3.89380600	3.47998700
C	-4.78433400	1.02299500	2.17699800
C	-3.01396700	1.40647300	3.73872600
C	-3.49578700	3.82175900	0.09924700
C	-1.83338300	4.45141500	1.69434300
C	-4.79162400	-0.34832500	2.46326000
H	-5.49558700	1.40393700	1.45370900
C	-2.98706000	0.05314500	4.06507600
O	-2.18218400	2.29684200	4.40435400
C	-2.71238000	4.28286000	-0.96307200
H	-4.46149700	3.38233300	-0.12555100
C	-1.02784300	4.94538900	0.67231600
O	-1.38009200	4.51498000	3.01672200
C	-5.74480900	-1.32059000	1.77078200
C	-3.88269500	-0.80406800	3.42911400
H	-2.28321500	-0.32361600	4.79491500
C	-0.86607900	2.37300400	4.01352600
C	-3.18230900	4.15157900	-2.41060100
C	-1.46608400	4.84387800	-0.64688900
H	-0.05809200	5.37158600	0.89424300
C	-0.43777600	3.56255300	3.33796900
H	-5.83759200	-2.18246500	2.43202100
C	-5.12923000	-1.83683000	0.47270500
O	-3.91434300	-2.15861000	3.78117600
C	-2.89971900	2.73510800	-2.89807200
H	-2.55315500	4.81656100	-3.00259400
O	-0.65169100	5.32167100	-1.67452000
C	-5.19903200	-1.11670400	-0.72476600
C	-4.42719900	-3.05057600	0.44994500
C	-2.85848500	-2.92275300	3.36661400
C	1.25750600	1.54898500	3.92100200
C	-3.82887900	1.69698100	-2.79905200
C	-1.64075400	2.43135300	-3.43873200
C	0.47109800	4.55931300	-1.93397600
C	1.69363200	2.74656400	3.27135500
C	-4.58265500	-1.54101400	-1.90808800
H	-5.72620000	-0.16954300	-0.72913600
C	-3.82606700	-3.52907100	-0.70852400
O	-4.29481000	-3.77394400	1.63444000
C	-3.04857500	-3.75835200	2.21124600
C	-3.54847100	0.38373900	-3.19681400
H	-4.81908000	1.92297400	-2.42285100
C	-1.29054200	1.12993100	-3.78775100
O	-0.77795300	3.48597600	-3.67668100
C	0.39443500	3.56384700	-2.95828600
C	-4.61154500	-0.71475700	-3.19077200
C	-3.89352600	-2.75812400	-1.86476400
H	-3.25725900	-4.44913500	-0.68628100
C	-0.70277600	-3.64027200	3.52510300
C	-2.24821900	0.13282300	-3.65275600
H	-0.29565900	0.91200800	-4.15427000
C	2.61130600	3.91036900	-1.48560600
H	-4.34786700	-1.39833000	-4.00185100
O	-3.10001800	-3.15483400	-2.95407200
C	-0.87945600	-4.44041000	2.35343100
O	-1.88060100	-1.16205300	-4.08064900
C	2.51986400	2.89313600	-2.49156900
C	3.04185200	2.86976400	2.86424300
C	2.19254500	0.51817300	4.17222200
C	0.21172900	-5.18593700	1.84994200
C	0.55588900	-3.61483400	4.17003800
C	3.60224800	2.00723100	-2.69227800
C	3.79138100	4.01730300	-0.71376900
C	-6.02073000	-0.18379500	-3.49697800
H	-6.01878600	0.37126200	-4.43852100
H	-6.40152500	0.48309700	-2.71920600
C	-7.15079500	-0.73803500	1.56433000
H	-7.80933900	-1.49497100	1.12985600
H	-7.16190700	0.12887100	0.89795500
C	-5.44053600	3.93560000	2.40436700
H	-6.04921400	3.63718600	3.26249100
H	-5.92381200	3.54762700	1.50305600
C	-4.64070800	4.58906300	-2.60214100
H	-4.75890500	5.63140800	-2.29513000
H	-5.34800600	3.99539700	-2.01557800
H	-4.92694400	4.50278600	-3.65422800
H	-5.44815500	5.02639200	2.33445000

H	-7.56966200	-0.42183400	2.52319900
H	-6.72083200	-1.01873500	-3.58417500
C	4.82818000	3.13423300	-0.92513800
C	4.73424900	2.12509800	-1.91604000
H	5.55445100	1.42379500	-2.02437400
H	5.71942000	3.17793200	-0.30867900
H	3.50083900	1.22454900	-3.43482200
H	3.83919300	4.79499800	0.04115000
C	3.50280700	0.66580100	3.76521800
H	1.84678500	-0.37413200	4.68475300
C	3.92889700	1.84192100	3.09944300
H	3.33904900	3.77369900	2.34527100
C	1.60398000	-4.35256800	3.66035800
H	0.66327800	-2.99762300	5.05537100
C	1.43148200	-5.13907200	2.49513200
H	0.05579000	-5.78944600	0.96195800
H	2.27161400	-5.70595900	2.10775000
H	2.57552000	-4.32023300	4.14139400
H	4.95456700	1.90794400	2.75252400
H	4.21524600	-0.13640400	3.91621500
P	-1.63935700	-2.35742100	-3.00924100
N	-0.57524700	-3.31488300	-3.80997600
C	0.04501500	-4.42807500	-3.08885900
C	-0.32214100	-3.27834800	-5.25254100
H	-0.16142200	-4.35028000	-2.01802200
H	1.12947200	-4.38980000	-3.23305600
H	-0.34192900	-5.38887300	-3.44633200
H	-0.75813400	-2.38053400	-5.68641000
H	-0.74739400	-4.16174400	-5.74237800
H	0.76034000	-3.26077100	-5.41502100
Au	-1.17568500	-1.68620000	-0.84915200
N	1.37862400	2.76182000	-3.23386600
N	1.55050200	4.73726500	-1.23074000
N	0.80235400	3.74426600	2.98967900
N	-0.05152100	1.39511500	4.28607000
N	-1.72874000	-2.87671400	4.00784200
N	-2.08980600	-4.48290600	1.71521900
C	-0.15708600	-1.32239900	1.28728600
C	-1.33047400	-0.94912900	1.23359100
H	-2.28541700	-0.53742600	1.49406200
C	1.26072700	-1.62382700	1.43132800
C	2.15125100	-1.12672100	0.24071100
H	1.41512800	-2.69304000	1.57393400
H	1.57949700	-1.12310500	2.35058200
C	3.52961100	-0.79539600	0.84364100
O	4.01817000	-1.53233300	1.68410800
O	4.04800100	0.28086000	0.32072100
H	5.00934200	0.48627100	0.66104200
C	2.32843000	-2.26914000	-0.79515400
H	2.79466200	-3.09617900	-0.24884900
H	1.33005400	-2.60345800	-1.09876200
C	1.46178500	0.08805400	-0.36112200
O	0.99453600	0.14199300	-1.48338000
O	1.27731300	1.02213800	0.57752700
C	0.35671100	2.08385200	0.28338800
H	-0.52990300	1.93712600	0.90436800
H	0.84326100	3.01550100	0.54705600
H	0.08305700	2.06375100	-0.76817500
C	3.14236300	-1.92956600	-2.02498700
C	4.53331600	-1.79159400	-1.95209800
C	2.50960100	-1.73434200	-3.25961700
C	5.27345900	-1.44187500	-3.08221800
H	5.05667000	-1.93669700	-1.01404300
C	3.24807500	-1.40888000	-4.39631500
H	1.42994100	-1.80637100	-3.31841000
C	4.63416300	-1.25512700	-4.30827200
H	6.34220000	-1.29309800	-2.97928200
H	2.74020900	-1.25216200	-5.34432700
H	5.21032000	-0.98289700	-5.18757700
S	7.51912800	0.41898100	0.31668500
O	8.74894300	1.16379100	0.58815000
O	7.18161800	0.11846800	-1.08945000
O	6.32801000	0.92378900	1.10719000
C	7.78684200	-1.25922100	1.06031600
F	8.87716300	-1.83207400	0.53248900
F	7.94632200	-1.17746000	2.38694700
F	6.73228900	-2.05964400	0.81235300

(TfO•1a)⊂AuCav-7

C	-4.89612400	2.70429700	2.26905900
C	-4.54000400	1.23566900	2.48529100
C	-3.97990900	3.37788900	1.25210300
H	-4.70585600	3.20038300	3.22132200
C	-5.10436000	0.19348200	1.74177000
C	-3.64192000	0.88307500	3.50357900
C	-4.20427200	3.26273900	-0.12298800
C	-2.89190700	4.16390900	1.65715900
C	-4.83532500	-1.15709400	1.99989100
H	-5.79878600	0.44151400	0.94756000
C	-3.35039800	-0.44276300	3.80788000
O	-3.09333500	1.90686700	4.26713200
C	-3.40935200	3.88971300	-1.08603100
H	-5.03674100	2.65456600	-0.45883100
C	-2.08179800	4.82223100	0.73537700
O	-2.60993000	4.29018600	3.02430400
C	-5.45944600	-2.29399500	1.19210900
C	-3.95949400	-1.44452400	3.05646400
H	-2.66316600	-0.68906000	4.60544100
C	-1.78691800	2.24914200	4.02181300
C	-3.68816800	3.72340600	-2.57850000
C	-2.33284100	4.66326500	-0.62555100
H	-1.24582300	5.42248100	1.07005800
C	-1.53491600	3.53354400	3.43632600
H	-5.43824400	-3.17392500	1.83558400
C	-4.59526900	-2.62873500	-0.02157500
O	-3.73488500	-2.78598300	3.37624000
C	-3.06134500	2.42241100	-3.06586000
H	-3.15900200	4.53139100	-3.08407900
O	-1.51840400	5.31348800	-1.55130600
C	-4.68590400	-1.90209300	-1.21315100
C	-3.64986100	-3.66512100	0.02811300
C	-2.49221300	-3.28250500	3.08944400
C	0.45236500	1.83319000	4.08884900
C	-3.74320800	1.20292400	-3.06651200
C	-1.73028800	2.42206700	-3.50896200
C	-0.24897200	4.80460000	-1.73557100
C	0.70885200	3.13330900	3.55213800
C	-3.87365500	-2.14923000	-2.32542900
H	-5.39839700	-1.08727100	-1.26844300
C	-2.83651400	-3.96477200	-1.06085400
O	-3.50018700	-4.39370000	1.20594400
C	-2.36307400	-4.12920700	1.93645100
C	-3.14947200	-0.00009600	-3.46763700
H	-4.78482600	1.19238400	-2.76909600
C	-1.07579800	1.24295100	-3.85305000
O	-1.10276200	3.64717900	-3.65785000
C	-0.02566000	3.93555400	-2.85061400
C	-3.94435300	-1.30126900	-3.59172200
C	-2.94996700	-3.19349800	-2.21213000
H	-2.08706000	-4.74131700	-0.97950600
C	-0.26176500	-3.49118400	3.49407400
C	-1.79317400	0.05404400	-3.81644200
H	-0.03480800	1.26631200	-4.14856100
C	1.95207700	4.61099300	-1.16672700
H	-3.45895500	-1.88087700	-4.38071700
O	-1.99133300	-3.38823100	-3.22945700
C	-0.13114100	-4.33419100	2.34613600
O	-1.12164600	-1.11178100	-4.23619000
C	2.17438700	3.76209800	-2.29836800
C	2.03894100	3.52139000	3.27081400
C	1.53532800	0.96359200	4.35208800
C	1.14326200	-4.82802700	1.99036100
C	0.88627300	-3.16023100	4.25097500
C	3.46689900	3.24629100	-2.54301200
C	3.02966000	4.89916900	-0.29669800
C	-5.39508800	-1.06967200	-4.04375000
H	-5.41234700	-0.49488500	-4.97315000
H	-5.98825400	-0.52452200	-3.30516400
C	-6.92413300	-2.02785900	0.81450100
H	-7.34147500	-2.89544500	0.29631000

H	-7.04722600	-1.16205300	0.15795500
C	-6.38202800	2.90547800	1.93176700
H	-7.00937900	2.46103900	2.70944500
H	-6.66879800	2.45552700	0.97695900
C	-5.17846800	3.85136900	-2.92233000
H	-5.55275300	4.82634900	-2.59961500
H	-5.79810800	3.08908200	-2.44106000
H	-5.32495300	3.76351800	-4.00255800
H	-6.60761500	3.97326800	1.87025200
H	-7.51486300	-1.84397700	1.71569200
H	-5.88812600	-2.02988300	-4.21652200
C	4.27745600	4.37351100	-0.55548300
C	4.50207900	3.55237000	-1.68791900
H	5.49673200	3.16020800	-1.86953300
H	5.10665000	4.56759600	0.11647100
H	3.60962400	2.60422500	-3.40478900
H	2.83888700	5.53894100	0.55838100
C	2.82300300	1.36522600	4.06567300
H	1.31690300	-0.01467100	4.76754200
C	3.07371700	2.64496700	3.51251100
H	2.20488100	4.50031400	2.83569700
C	2.12152800	-3.64136000	3.86908500
H	0.75834400	-2.52031900	5.11739400
C	2.25114000	-4.47668000	2.73339500
H	1.22733400	-5.45843300	1.11498100
H	3.23224200	-4.82201200	2.42680800
H	3.00784000	-3.36815800	4.43189900
H	4.09163700	2.92106300	3.26101600
H	3.65410700	0.68826100	4.22200700
P	-0.76312300	-2.29534300	-3.17458300
N	0.44655400	-3.08730300	-3.96526800
C	0.59470800	-4.54427600	-3.97106000
C	1.65770400	-2.33366400	-4.32271900
H	-0.33168700	-5.02066800	-3.65473100
H	1.41323600	-4.85251100	-3.31083100
H	0.82299500	-4.87310500	-4.99029500
H	1.48890800	-1.26107500	-4.21809800
H	1.91459400	-2.53878800	-5.36645700
H	2.49453300	-2.62401600	-3.67864600
Au	-0.66221600	-1.56254400	-0.98492100
N	1.14153800	3.44170400	-3.13366800
N	0.70916500	5.13014100	-0.91768900
N	-0.32601100	3.96783200	3.23144400
N	-0.82954600	1.42279600	4.32762600
N	-1.47884000	-2.97953800	3.84528700
N	-1.22287600	-4.63868000	1.57655500
C	0.12181500	-1.16921200	1.20335500
C	-1.10070200	-1.02858200	1.12366000
H	-2.12495100	-0.82957900	1.36854700
C	1.56638100	-1.21464500	1.37499300
C	2.36433200	-0.35790800	0.34187600
H	1.92074800	-2.24603000	1.34696400
H	1.76671100	-0.83558800	2.38090400
C	3.57853200	0.23364900	1.07531800
O	4.16646100	-0.39329200	1.93885000
O	3.87579500	1.42580000	0.61687100
H	4.87697200	1.64005100	0.75041000
C	2.88904200	-1.16595000	-0.88554500
H	2.06442300	-1.22877900	-1.59829500
H	3.66958600	-0.55777800	-1.35160600
C	1.47050600	0.75928900	-0.18947300
O	1.18100300	0.92586900	-1.35857400
O	0.93693900	1.45029000	0.82439100
C	-0.16312900	2.31896300	0.51900600
H	-0.99974900	2.00776900	1.14561400
H	0.12890900	3.33468700	0.76229300
H	-0.42744700	2.23685200	-0.53333100
C	3.40873600	-2.55308700	-0.61188800
C	2.62187400	-3.66378000	-0.94635100
C	4.68546600	-2.77009200	-0.07301200
C	3.09410300	-4.96524800	-0.76672300
H	1.62844500	-3.50104700	-1.35774200
C	5.14935300	-4.07019400	0.12663800
H	5.31683500	-1.92353000	0.16483600
C	4.36222600	-5.17165200	-0.22278800

H	2.47471300	-5.81294100	-1.04860400
H	6.14248500	-4.22138400	0.53888100
H	4.73936900	-6.18050600	-0.08307800
S	6.85546900	1.05000300	-0.57011100
O	7.61075500	1.84842000	-1.54407700
O	5.86208100	0.09772300	-1.11419000
O	6.33714500	1.85245700	0.60065500
C	8.10304400	-0.05234300	0.24400200
F	8.72488000	-0.80549500	-0.67401200
F	9.02049300	0.66109900	0.90634800
F	7.49228900	-0.87513000	1.11740600

(TfO•1a)⊂AuCav-8

C	3.91090000	-1.53988600	3.90800000
C	3.64817100	-0.10428900	3.47052400
C	3.27411500	-2.55063800	2.95982900
H	3.39961000	-1.66173700	4.86329600
C	4.48384500	0.55546600	2.56253100
C	2.56463400	0.61689200	3.99198400
C	3.91494900	-3.01636500	1.80611700
C	2.00848000	-3.08160500	3.24731500
C	4.30373400	1.89149100	2.20103600
H	5.31856800	0.00937800	2.13878800
C	2.34213000	1.95131800	3.66096600
O	1.73911100	0.00506200	4.93269700
C	3.37049600	-4.01097400	0.98510100
H	4.88233900	-2.60072200	1.54698700
C	1.43548900	-4.08390000	2.47542600
O	1.34630900	-2.62243800	4.38247700
C	5.19724400	2.60710000	1.18915600
C	3.22324500	2.56733800	2.78065000
H	1.51939600	2.49887400	4.10211800
C	0.49008400	-0.38562200	4.51308800
C	4.05412700	-4.47156900	-0.30130200
C	2.13425000	-4.55271300	1.36655600
H	0.46702100	-4.49445900	2.72897100
C	0.29199400	-1.76729200	4.18309500
H	5.14047400	3.67235700	1.41474500
C	4.58526400	2.41474400	-0.19372900
O	3.12113300	3.93041900	2.51637000
C	3.62992800	-3.54560600	-1.43844800
H	3.64729300	-5.45636300	-0.53281700
O	1.61604100	-5.61386500	0.63399700
C	4.86124700	1.28093100	-0.96389200
C	3.66632900	3.33536600	-0.71690600
C	2.05581400	4.44644900	1.84978500
C	-1.70022000	0.03111100	4.04006100
C	4.31201100	-2.36110500	-1.73348000
C	2.47976700	-3.83341700	-2.19077900
C	0.45251900	-5.35342400	-0.05401200
C	-1.87892500	-1.33226100	3.64047900
C	4.23594300	1.00810000	-2.18380700
H	5.59829200	0.57706600	-0.59658800
C	2.97417800	3.07612300	-1.89591800
O	3.48802800	4.56511700	-0.08209100
C	2.27544900	4.85174900	0.48613400
C	3.87693500	-1.45802200	-2.70924600
H	5.21232900	-2.12863700	-1.17668600
C	1.99494600	-2.95178700	-3.15154900
O	1.83232900	-5.03711900	-1.98072000
C	0.56724800	-5.00201900	-1.43258900
C	4.63539300	-0.18109200	-3.05464500
C	3.23985800	1.90367000	-2.59189400
H	2.24959800	3.78471100	-2.26974600
C	-0.03314900	5.34774300	1.78006600
C	2.69258400	-1.77479800	-3.38354300
H	1.08215600	-3.18096100	-3.68671400
C	-1.80728700	-5.14570700	-0.18830000
H	4.34004600	0.08005000	-4.07410700
O	2.46158900	1.63786300	-3.73040800
C	0.21976100	5.83271900	0.46086600
O	2.13754600	-0.86201800	-4.29371600
C	-1.69022400	-4.75185300	-1.56173300
C	-3.14140600	-1.76070800	3.17069100
C	-2.80022300	0.91891700	3.99861700

C	-0.75596900	6.62513300	-0.18505700
C	-1.27271800	5.62537900	2.40151600
C	-2.85637000	-4.43414800	-2.29378500
C	-3.08596600	-5.23376100	0.40557600
C	6.15752300	-0.39519100	-3.07352400
H	6.41162400	-1.19697800	-3.77162300
H	6.55964900	-0.67114500	-2.09540700
C	6.67092900	2.19050800	1.27220600
H	7.26354500	2.75942000	0.55062400
H	6.83138300	1.12875500	1.06542200
C	5.40313100	-1.82145300	4.14293800
H	5.80846700	-1.11545400	4.87292800
H	6.00384000	-1.73666800	3.23287500
C	5.57566600	-4.61048500	-0.16345000
H	5.81482700	-5.32234900	0.63096200
H	6.07357600	-3.66786100	0.08204600
H	6.00708400	-4.97672400	-1.09916900
H	5.53465700	-2.83590000	4.52872300
H	7.05907500	2.39015600	2.27445600
H	6.66279300	0.52044500	-3.39186600
C	-4.20563600	-4.93560000	-0.33769700
C	-4.09013400	-4.52197300	-1.68762200
H	-4.99252700	-4.25094200	-2.22260200
H	-5.18812700	-4.98094100	0.11517300
H	-2.74504600	-4.12202400	-3.32658100
H	-3.14890900	-5.53493600	1.44558400
C	-4.02822500	0.46286200	3.56564600
H	-2.64349300	1.94358700	4.32006300
C	-4.19859700	-0.87612400	3.14281300
H	-3.25976100	-2.78376500	2.83181200
C	-2.21434400	6.38754200	1.74240800
H	-1.44208100	5.23628300	3.39963800
C	-1.94906400	6.89947800	0.45039500
H	-0.53681600	7.00503200	-1.17694800
H	-2.70174300	7.50178900	-0.04733200
H	-3.16707100	6.60124100	2.21512500
H	-5.15534500	-1.21679300	2.76822000
H	-4.87685500	1.13907200	3.53024500
P	1.35078200	0.42464800	-3.61481000
N	0.21458300	0.92036700	-4.69457300
C	0.57596400	1.55270200	-5.96688300
C	-1.06418600	0.19011700	-4.69919400
H	1.49596200	2.12546500	-5.85166800
H	-0.22995200	2.23414300	-6.25484100
H	0.70949300	0.80742100	-6.76079000
H	-1.28409800	-0.19998900	-3.70418100
H	-1.04262900	-0.63366300	-5.42293500
H	-1.86243100	0.88898600	-4.95591400
Au	0.78123500	-0.00061700	-1.44004000
N	-0.46203000	-4.69362400	-2.16449800
N	-0.69398600	-5.42875800	0.55223400
N	-0.84037000	-2.21894900	3.73372700
N	-0.47293100	0.48619400	4.44548200
N	0.92759700	4.65253100	2.46372700
N	1.39368700	5.53586600	-0.18074100
C	0.40502600	0.55610900	0.83957800
C	1.24308200	-0.33986800	0.82619200
H	1.97900300	-1.09272400	1.02866100
C	-0.56920600	1.63537500	1.02299500
C	-1.78402500	1.67870400	0.04654000
H	-0.04552200	2.59087500	0.96050200
H	-0.95087800	1.54263600	2.04406600
C	-2.34208700	0.25650900	-0.18699500
O	-1.67417500	-0.59010700	-0.78242100
O	-3.52335600	0.07323800	0.31221100
H	-4.03394900	-0.77389800	-0.02755400
C	-2.81944900	2.66737900	0.68128300
H	-3.24078900	2.15803900	1.54856300
H	-2.24982400	3.52721000	1.04411400
C	-1.43304800	2.23275400	-1.33752200
O	-1.95363200	1.86793200	-2.36691800
O	-0.56786500	3.26753200	-1.27803300
C	-0.39218200	3.96433400	-2.52998700
H	0.03260800	3.29256000	-3.27759000
H	-1.35854500	4.32821600	-2.88382500
H	0.28053600	4.78842700	-2.30363200
C	-3.91975500	3.16066600	-0.23179800
C	-3.71951700	4.31068400	-1.00679500
C	-5.14969200	2.49950800	-0.31850500
C	-4.71567000	4.78007700	-1.86187800
H	-2.77892200	4.84374900	-0.92944300
C	-6.14714600	2.96463900	-1.17474900
H	-5.32096500	1.61358900	0.27514300
C	-5.93366000	4.10425000	-1.95127100
H	-4.54267200	5.67496900	-2.45374600
H	-7.08552800	2.42327900	-1.23283300
H	-6.71130000	4.46523400	-2.61791400
S	-6.19213600	-2.27340600	0.00624100
O	-5.94555400	-2.77433200	1.37238000
O	-6.95686400	-3.13770400	-0.90025400
O	-4.98409200	-1.66010900	-0.65209800
C	-7.28504800	-0.79576600	0.28513300
F	-8.48385300	-1.16524200	0.74240600
F	-6.72506200	0.02999200	1.20354000
F	-7.45334000	-0.09147400	-0.84477800

(TfO•1a)⊂AuCav-9

C	-3.99638700	-1.02096200	-4.09745400
C	-3.70876400	0.37778700	-3.56517000
C	-3.38001700	-2.11298000	-3.22874900
H	-3.48898600	-1.08496000	-5.06063700
C	-4.51553900	0.99038200	-2.60091900
C	-2.62284700	1.11542300	-4.05466200
C	-4.04812300	-2.67068700	-2.13371100
C	-2.11778000	-2.63731700	-3.54180900
C	-4.29888300	2.29475600	-2.15030200
H	-5.34756600	0.42936300	-2.19051900
C	-2.37151800	2.42048200	-3.64573000
O	-1.80652900	0.54229800	-5.02680300
C	-3.53416300	-3.74413300	-1.39788500
H	-5.01590500	-2.26729600	-1.85721300
C	-1.57496300	-3.71622100	-2.85523500
O	-1.43206500	-2.10644000	-4.63237300
C	-5.13342100	2.93393500	-1.04302000
C	-3.22088200	2.99046100	-2.70697800
H	-1.53969900	2.97678700	-4.05729300
C	-0.56545800	0.12734100	-4.61195000
C	-4.24391400	-4.30979300	-0.17021500
C	-2.30136100	-4.27073900	-1.80601200
H	-0.61193500	-4.12289900	-3.13490900
C	-0.37294800	-1.27482000	-4.37464500
H	-5.02291600	4.01393900	-1.14418800
C	-4.49945600	2.54554300	0.28735800
O	-3.05280200	4.32486000	-2.33449700
C	-3.81282100	-3.50987200	1.05601100
H	-3.86509200	-5.32260600	-0.03071000
O	-1.82366100	-5.41191300	-1.16714500
C	-4.85556700	1.37306900	0.95855100
C	-3.48158300	3.32270100	0.85689900
C	-1.92558100	4.69309800	-1.66832900
C	1.61562200	0.51220800	-4.08445700
C	-4.45648100	-2.32965100	1.43752400
C	-2.70026700	-3.90954500	1.81289500
C	-0.67191900	-5.25586200	-0.43530000
C	1.80108500	-0.88129700	-3.81453100
C	-4.23153700	0.93937900	2.13207000
H	-5.67163500	0.78186700	0.56178800
C	-2.78142200	2.89246400	1.97789600
O	-3.23408000	4.59588100	0.34391900
C	-2.03272700	4.87896300	-0.24440500
C	-4.02832600	-1.54553700	2.51327200
H	-5.32028000	-2.00332700	0.86999600
C	-2.22901900	-3.15178100	2.88106900
O	-2.07567800	-5.10358100	1.49812400
C	-0.80372700	-5.03871400	0.97102600
C	-4.73511200	-0.26246900	2.92360800
C	-3.14355400	1.69465700	2.58268200
H	-1.99465100	3.50312800	2.39398200
C	0.22278900	5.43998200	-1.60128100
C	-2.89500100	-1.97820600	3.20661600

H	-1.35249600	-3.47348100	3.42878000
C	1.58986600	-5.13619600	-0.23928400
H	-4.45802100	-0.07750500	3.96476100
O	-2.43895800	1.32954500	3.73628900
C	0.09271200	5.69582900	-0.20177700
O	-2.37321300	-1.19565700	4.25040400
C	1.45634400	-4.85985500	1.16040600
C	3.07371000	-1.34750000	-3.41349900
C	2.71037700	1.39905700	-3.96557100
C	1.17329800	6.27925900	0.49670100
C	1.44137600	5.73193100	-2.25640000
C	2.61579900	-4.64145600	1.93821300
C	2.87667100	-5.22349000	-0.81395200
C	-6.26501000	-0.39758100	2.88794800
H	-6.57971200	-1.21486300	3.54204800
H	-6.65091100	-0.61191700	1.88816900
C	-6.62818000	2.60506000	-1.13729000
H	-7.17622700	3.10165700	-0.33171000
H	-6.83878300	1.53415500	-1.06880500
C	-5.49472400	-1.25185500	-4.34971900
H	-5.88058900	-0.49292100	-5.03587100
H	-6.09489800	-1.20488000	-3.43650500
C	-5.76724800	-4.39496100	-0.33235500
H	-6.01573800	-5.01760300	-1.19586500
H	-6.23887900	-3.42002500	-0.48613700
H	-6.21725900	-4.84131100	0.55882300
H	-5.65179800	-2.23774100	-4.79552100
H	-7.02548000	2.95388200	-2.09406600
H	-6.73429600	0.52781100	3.23235000
C	3.98924500	-5.03452300	-0.02609700
C	3.85875200	-4.72377800	1.34941900
H	4.75811100	-4.53027700	1.92178400
H	4.97969000	-5.08672600	-0.45898300
H	2.49278300	-4.41541600	2.99209700
H	2.95221300	-5.43640700	-1.87452400
C	3.94811200	0.90951900	-3.60207300
H	2.54292800	2.44933100	-4.18149600
C	4.12948800	-0.46478700	-3.32390200
H	3.20443400	-2.39732500	-3.17746000
C	2.48624900	6.28501800	-1.54618600
H	1.51400600	5.51588000	-3.31675500
C	2.34697800	6.56954800	-0.16760200
H	1.05128800	6.48487400	1.55447200
H	3.18112900	7.00421300	0.37253400
H	3.42412600	6.50714000	-2.04429900
H	5.09605000	-0.83623500	-3.01147200
H	4.79464100	1.58413200	-3.51639800
P	-1.40026100	0.05889800	3.79966800
N	-0.54508800	0.47956500	5.14734400
C	-1.24706500	0.84380500	6.38314300
C	0.81038300	-0.05265700	5.33835400
H	-2.20777800	1.30117500	6.14702200
H	-0.64071200	1.57253000	6.92931600
H	-1.41025800	-0.03233900	7.02277600
H	1.30775200	-0.17933100	4.37585300
H	0.79876500	-1.00903700	5.87606800
H	1.38571600	0.67397000	5.91850600
Au	-0.17090400	-0.38265400	1.97533200
N	0.21853800	-4.82445000	1.74509100
N	0.48484700	-5.31122900	-1.02375600
N	0.76167000	-1.76058600	-3.96851600
N	0.39119200	0.99486000	-4.46402800
N	-0.82810500	4.93892600	-2.32053100
N	-1.06257300	5.37718000	0.46441200
C	-0.65853200	0.88566100	-0.63537400
C	-1.59050700	0.11234200	-0.59990200
H	-2.41036700	-0.57053300	-0.59608100
C	0.43613500	1.84339500	-0.77248900
C	1.69277200	1.67909700	0.14291500
H	0.04876800	2.84797900	-0.59620000
H	0.78213700	1.81689100	-1.81089200
C	2.18903300	0.22046400	0.15249100
O	1.51538200	-0.70637900	0.66829000
O	3.31014500	0.02358200	-0.42247000
H	3.89092500	-0.85923500	-0.14010000
C	2.76451200	2.67720800	-0.42086800
H	3.08620600	2.27469300	-1.38195600
H	2.23398800	3.61159300	-0.61570800
C	1.45752900	2.08388500	1.60171900
O	2.02692900	1.58313700	2.54994600
O	0.63899800	3.14294600	1.70771900
C	0.51533100	3.69328600	3.03463600
C	3.96150800	2.96575600	0.45703900
C	3.89504000	3.96899700	1.43347000
C	5.16069400	2.26285600	0.30258000
C	4.99156400	4.25274900	2.24571500
H	2.97927200	4.53762500	1.54595400
C	6.26005200	2.54233800	1.11379700
H	5.22875500	1.48614500	-0.44522600
C	6.17904800	3.53631200	2.08932400
H	4.92010800	5.03432000	2.99719200
H	7.17241300	1.97127300	0.98345000
H	7.03512200	3.75277000	2.72143600
S	5.93457700	-2.36360100	-0.33436100
O	5.59082000	-2.72707500	-1.71868700
O	6.61817500	-3.35968000	0.49510400
O	4.80967200	-1.67103300	0.41123400
C	7.19073100	-1.00831300	-0.52598600
F	8.30935500	-1.48574500	-1.07565500
F	6.71513100	-0.02967100	-1.33194200
F	7.49106700	-0.45686400	0.65773300
H	0.04286400	2.96751800	3.70016700
H	1.50294000	3.94888400	3.42279300
H	-0.10339400	4.58042000	2.91151600

(TfO•1a)⊂AuCav-10

C	1.19671000	3.87052000	3.78271700
C	0.53827900	4.04056500	2.41619300
C	1.79683500	2.47239300	3.88904200
H	0.39379800	3.91809600	4.51846100
C	1.26856800	4.45265900	1.29915600
C	-0.82576300	3.76461800	2.22958200
C	3.10933100	2.17943700	3.50608400
C	1.00900400	1.41605200	4.36892600
C	0.69482900	4.63252900	0.03827900
H	2.32791500	4.64995400	1.41960700
C	-1.43678100	3.90446000	0.98396400
O	-1.59422600	3.46698800	3.34691800
C	3.64455700	0.89051200	3.58639800
H	3.74102300	2.98795400	3.15660800
C	1.48159500	0.10742800	4.42482100
O	-0.22872500	1.73070100	4.89427800
C	1.52271900	5.08737300	-1.16114000
C	-0.67439500	4.35495200	-0.09295400
H	-2.49588600	3.70715400	0.86767800
C	-2.11579400	2.20261900	3.49927900
C	5.09413300	0.56083900	3.23752500
C	2.79012500	-0.12805000	4.02669200
H	0.84735700	-0.68959000	4.78942500
C	-1.37620000	1.26841700	4.29591400
H	0.81791000	5.49308300	-1.88734100
C	2.16583700	3.87060400	-1.81816800
O	-1.29375600	4.66895000	-1.29784400
C	5.20850900	0.13848400	1.77751100
H	5.36516500	-0.31217500	3.83200300
O	3.35750800	-1.40023500	4.15924600
C	3.43674500	3.40772100	-1.47327400
C	1.46046900	3.15650700	-2.80092900
C	-1.79628900	3.67238900	-2.09869600
C	-3.74993900	0.65058100	3.21715500
C	5.27445000	1.08972100	0.75386200
C	5.23252100	-1.21175600	1.39910100
C	3.00071400	-2.40939500	3.33159400
C	-2.98309600	-0.29689100	3.96510600
C	4.02536200	2.28265800	-2.06235400
H	4.00070300	3.95532600	-0.72847000
C	1.98636100	2.00587000	-3.38714800
O	0.29279000	3.70542900	-3.26977600
C	-0.94279900	3.11599800	-3.10324500
C	5.36697200	0.75690400	-0.59976500

H	5.24142000	2.13799100	1.02593400
C	5.30725100	-1.59368600	0.06082000
O	5.20579500	-2.21218500	2.37235400
C	4.02832100	-2.91454600	2.45501500
C	5.43000600	1.81520600	-1.69650400
C	3.25659300	1.59225600	-3.00641800
H	1.42685100	1.48907800	-4.15744500
C	-3.52253700	2.41409700	-2.89017000
C	5.36932000	-0.60527800	-0.91158100
H	5.32129700	-2.64407500	-0.20255700
C	1.59557800	-4.04520900	2.60558900
H	5.84279900	1.32839600	-2.58341300
O	3.82484000	0.50883400	-3.71312800
C	-2.64785300	1.81247500	-3.85464000
O	5.42377400	-1.00468800	-2.26368900
C	2.65447100	-4.61854800	1.83662700
C	-3.48182900	-1.60803700	4.14106000
C	-5.00932300	0.27929500	2.69922200
C	-3.16393500	0.85278300	-4.75224500
C	-4.88502000	2.05132100	-2.85799700
C	2.42474000	-5.81527000	1.11757500
C	0.31786400	-4.65033100	2.59135800
C	6.38162100	2.96738500	-1.34286900
H	7.38847200	2.57776200	-1.17222200
H	6.08204000	3.50728200	-0.44138700
C	2.52807800	6.19364000	-0.81597700
H	3.06874700	6.50880300	-1.71311900
H	3.26963800	5.88666700	-0.07260200
C	2.19666900	4.98734000	4.11046900
H	1.69694300	5.95883100	4.07071800
H	3.04226400	5.02714100	3.41723800
C	6.07311700	1.68773800	3.59841400
H	5.99098100	1.92471600	4.66224000
H	5.88924800	2.61075100	3.04169300
H	7.10011900	1.37580200	3.38928300
H	2.60234500	4.84587500	5.11621200
H	1.99904700	7.05844600	-0.40780200
H	6.41979600	3.68791400	-2.16381100
C	0.11289000	-5.80787700	1.86722500
C	1.17154200	-6.39867900	1.13771300
H	0.99338800	-7.31637200	0.58654200
H	-0.86879300	-6.26902700	1.85662700
H	3.25057000	-6.24679800	0.56133300
H	-0.47597900	-4.18408100	3.16481800
C	-5.47142200	-1.00713100	2.88147500
H	-5.59620100	1.00311100	2.14941200
C	-4.70201600	-1.95542600	3.59628500
H	-2.88089900	-2.31161700	4.70805100
C	-5.35908600	1.10445200	-3.73823600
H	-5.54009600	2.49521800	-2.12053600
C	-4.49781900	0.50826300	-4.68812000
H	-2.48934900	0.41311300	-5.47962000
H	-4.89409600	-0.23561900	-5.37281500
H	-6.39933400	0.80660600	-3.67080200
H	-5.07965100	-2.96612100	3.71735600
H	-6.42759300	-1.28607200	2.45369300
P	4.02511000	-0.97096000	-3.09778500
N	4.46644000	-1.80286200	-4.47283300
C	3.56676900	-1.69683900	-5.63148500
C	5.00880500	-3.15378400	-4.27008000
H	3.25698600	-0.66202700	-5.77109800
H	2.67766500	-2.33520300	-5.52545300
H	4.11868400	-2.01393900	-6.51987200
H	5.71964000	-3.15173100	-3.44414300
H	5.54016900	-3.44707000	-5.17898100
H	4.21897700	-3.89330500	-4.07265800
Au	2.30304700	-1.81415200	-1.83855600
N	3.86957600	-3.98942100	1.74346000
N	1.81043400	-2.93101800	3.36999200
N	-1.78146900	0.05730600	4.51921800
N	-3.27139000	1.91382500	2.99029600
N	-3.04661400	3.33418200	-1.99757300
N	-1.33614000	2.19824400	-3.93687300
C	0.15893400	-2.58182100	-1.12558900
C	1.05771300	-3.34492300	-0.77263700
H	1.58056100	-4.19089900	-0.37067500
C	-0.96538800	-1.73086200	-1.47382100
C	-1.57537300	-1.00776700	-0.25438400
H	-1.74522400	-2.35507300	-1.91899000
H	-0.67793400	-1.00409900	-2.23642000
C	-2.78078400	-0.22114500	-0.81748600
O	-3.21195700	-0.48050500	-1.92133100
O	-3.28129800	0.63821200	0.04611600
H	-4.25394100	0.79615100	-0.18201300
C	-2.12791500	-2.01378200	0.80991800
H	-1.26967300	-2.50058800	1.28439100
H	-2.61963600	-1.41947500	1.57737200
C	-0.58293600	-0.09578300	0.45826400
O	-0.69122200	0.27651500	1.60287700
O	0.47820100	0.23108600	-0.31983700
C	1.49327900	1.00869800	0.35587000
H	1.87390000	0.45805600	1.21818100
H	2.27454700	1.16704200	-0.38144300
H	1.08052100	1.95709200	0.69155400
C	-3.06248300	-3.05235900	0.24107000
C	-2.58158900	-4.33036300	-0.07449200
C	-4.40922100	-2.75586600	-0.01181400
C	-3.42086100	-5.29522500	-0.63250400
H	-1.54181500	-4.57051200	0.12580500
C	-5.24775100	-3.71785800	-0.57469200
H	-4.81126700	-1.77571900	0.21950400
C	-4.75807500	-4.98765000	-0.88562800
H	-3.03174000	-6.28235900	-0.86758200
H	-6.28246000	-3.46385700	-0.77051500
H	-5.41599700	-5.73337300	-1.32219200
S	-7.19623700	0.77114600	-0.29978200
O	-7.64819300	1.09004800	1.06460500
O	-5.75363100	0.37103200	-0.38924200
O	-7.64485300	1.65678400	-1.38752400
C	-8.00068300	-0.85642800	-0.67671100
F	-7.67609200	-1.77426800	0.26443800
F	-9.33296300	-0.74429200	-0.70152500
F	-7.59062400	-1.33491800	-1.86438000

1a-TS1$_{5anti}$H$^{+}\subset$AuCav

C	-1.57269000	4.50077900	2.69125000
C	-0.90130400	3.26392700	3.28253200
C	-1.85932800	4.30432400	1.20655200
H	-0.84037100	5.30578900	2.75766400
C	-1.63159500	2.20224100	3.82141600
C	0.49585100	3.16553200	3.32986700
C	-3.02811400	3.68746200	0.74824200
C	-0.94548900	4.74161900	0.23954400
C	-1.02885500	1.09583000	4.42686800
H	-2.71368500	2.25082500	3.79131600
C	1.14552600	2.08108400	3.90945600
O	1.24178900	4.24838800	2.86743600
C	-3.30111200	3.49079800	-0.60868900
H	-3.75480800	3.35059400	1.47837300
C	-1.17331500	4.57661500	-1.12253600
O	0.20573700	5.40814000	0.66242500
C	-1.84453000	-0.04950300	5.01809200
C	0.36955600	1.06654000	4.45892500
H	2.22614200	2.04491500	3.95750100
C	1.94617700	4.12628400	1.70720900
C	-4.58231500	2.82657900	-1.10417000
C	-2.33831500	3.93393100	-1.52409700
H	-0.45258200	4.92666600	-1.84944200
C	1.40087700	4.75915200	0.53372200
H	-1.19757000	-0.55838700	5.73276000
C	-2.15655700	-1.05585100	3.91710100
O	0.99091200	0.01968900	5.15146700
C	-4.42834500	1.30951900	-1.13207000
H	-4.71007200	3.13983700	-2.14052100
O	-2.59415800	3.74400300	-2.88455800
C	-3.28351400	-0.93795900	3.10060000
C	-1.29323300	-2.12975000	3.66367900
C	1.70885900	-0.89237300	4.44468200
C	3.77683900	3.50096900	0.51295700
C	-4.67594600	0.52370000	-0.00249300

C	-4.03629800	0.64429700	-2.30292600
C	-1.85957000	2.78513600	-3.52000900
C	3.24059300	4.13380400	-0.64914000
C	-3.58278800	-1.83960500	2.07546400
H	-3.96902700	-0.12035100	3.28426500
C	-1.52902800	-3.03959700	2.63668500
O	-0.21719200	-2.31972800	4.52484000
C	1.05655800	-2.12698600	4.07990000
C	-4.57094400	-0.86965000	-0.00504200
H	-4.97622300	1.01785700	0.91347900
C	-3.91069500	-0.74241700	-2.35308100
O	-3.81871000	1.38920600	-3.46014000
C	-2.51729700	1.55176000	-3.85885500
C	-4.84898700	-1.71128800	1.23509500
C	-2.66800200	-2.87709300	1.85603700
H	-0.85253800	-3.86967300	2.47665300
C	3.63605100	-1.64592900	3.49662300
C	-4.18219100	-1.47453100	-1.20395200
H	-3.62020100	-1.22889900	-3.27554500
C	0.05568800	2.02312700	-4.49233700
H	-5.09882800	-2.71603700	0.88653800
O	-2.94162600	-3.85860000	0.89208500
C	2.97820000	-2.84915000	3.09720700
O	-4.09502600	-2.87755000	-1.26537600
C	-0.61986200	0.83002800	-4.89344700
C	3.97161400	4.10443600	-1.85921400
C	5.04000900	2.86902300	0.44025700
C	3.70442200	-3.84261700	2.40213900
C	5.00975900	-1.47348500	3.20850100
C	0.07524400	-0.13887900	-5.65564400
C	1.42071400	2.19584200	-4.81505500
C	-6.05806400	-1.20945200	2.03794100
H	-6.94814100	-1.20778200	1.40398200
H	-5.92802000	-0.19504200	2.42295600
C	-3.09188600	0.42390900	5.77689100
H	-3.61519000	-0.43154400	6.21231000
H	-3.80446900	0.96036800	5.14440700
C	-2.81362400	4.93989500	3.48245000
H	-2.54719700	5.12101500	4.52697800
H	-3.61645700	4.19725400	3.47175000
C	-5.82569100	3.28228100	-0.32563400
H	-5.92869600	4.36818800	-0.39338600
H	-5.78750000	3.02253800	0.73589200
H	-6.72325900	2.82173200	-0.74675400
H	-3.21665900	5.86466700	3.06154000
H	-2.79976900	1.09925000	6.58485700
H	-6.24016200	-1.86667500	2.89192800
C	2.07926600	1.22906000	-5.54399300
C	1.40140000	0.06518500	-5.97642600
H	1.93658100	-0.67786100	-6.55818100
H	3.12817400	1.35609700	-5.78870900
H	-0.46259700	-1.02619800	-5.97272500
H	1.91412300	3.10187100	-4.48048400
C	5.73858900	2.86270300	-0.75048900
H	5.43601300	2.41562500	1.34287600
C	5.19973300	3.47746600	-1.90633900
H	3.54312300	4.59568900	-2.72620500
C	5.70264000	-2.46818300	2.54899900
H	5.48707700	-0.55449600	3.53036200
C	5.04708000	-3.65534000	2.14505300
H	3.18805100	-4.75434400	2.12113800
H	5.60971400	-4.43558600	1.64505200
H	6.76220400	-2.34251500	2.35010000
H	5.76300200	3.45933100	-2.83332400
H	6.71779800	2.39490200	-0.79849000
P	-2.74544000	-3.60485100	-0.70545700
N	-3.00459800	-5.15759400	-1.25971000
C	-2.18118400	-6.22342800	-0.67345200
C	-3.22176500	-5.30939100	-2.70594400
H	-2.12164900	-6.09948400	0.40722200
H	-1.16664200	-6.24587500	-1.09829400
H	-2.66473500	-7.18117200	-0.88194300
H	-3.89566300	-4.53313800	-3.06687400
H	-3.69422200	-6.27967400	-2.87873200
H	-2.28092100	-5.27094000	-3.27373100
Au	-0.87953600	-2.46914000	-1.43539600
N	-1.92039200	0.60813200	-4.52538300
N	-0.60837200	2.99909200	-3.80254900
N	2.02268700	4.75685300	-0.60776400
N	3.08924400	3.50731300	1.69577900
N	2.95528300	-0.66042300	4.15757500
N	1.65916000	-3.06082600	3.40448700
C	1.85247600	-1.85496000	-2.21896100
C	0.65607300	-1.60850100	-2.55597700
H	0.34405200	-0.96021800	-3.36565700
C	2.96147900	-2.42789700	-1.44460700
C	4.00766800	-1.32833000	-1.18264700
H	3.41284400	-3.26328400	-1.98706500
H	2.56378400	-2.81519100	-0.50166200
C	4.01007900	-0.49384300	-2.47161100
O	3.09810300	-0.66361200	-3.27688500
O	4.96133200	0.38265900	-2.68218200
H	5.65090200	0.27648500	-1.98182300
C	3.63335600	-0.42783100	0.05332100
H	4.35168600	0.39373900	0.09837300
H	3.79384900	-1.04264400	0.93902300
C	5.43706100	-1.81348800	-0.97074600
O	6.36537400	-1.01766000	-0.91451000
O	5.57334500	-3.12709800	-0.89216100
C	6.93322900	-3.61643400	-0.76560100
H	6.84400900	-4.69939800	-0.71521500
H	7.51575200	-3.31163200	-1.63606900
H	7.39091900	-3.21453200	0.13842700
C	2.22128900	0.09840300	0.03239800
C	1.23315900	-0.47168800	0.84292800
C	1.87118900	1.15431900	-0.81704800
C	-0.08319600	-0.00543100	0.80070800
H	1.49075700	-1.29002100	1.50432900
C	0.56012000	1.61846700	-0.86800900
H	2.62270700	1.61949100	-1.44565800
C	-0.42382800	1.03956500	-0.06100300
H	-0.83436000	-0.44554500	1.44623100
H	0.31208400	2.43782600	-1.52881900
H	-1.44211500	1.41148800	-0.09171700

1a-Int$_{5anti}$H^{+}⊂AuCav

C	-1.18934600	-4.48979100	-2.90031400
C	-0.62696800	-3.17134100	-3.42545100
C	-1.48111100	-4.39434500	-1.40698500
H	-0.39361500	-5.22679800	-3.01139800
C	-1.44851800	-2.15001600	-3.90836800
C	0.75627600	-2.95046200	-3.46829400
C	-2.68950000	-3.89071300	-0.91336300
C	-0.52875200	-4.80682100	-0.46735700
C	-0.94716500	-0.96680600	-4.45805300
H	-2.52210800	-2.29362100	-3.88084600
C	1.30690600	-1.78576100	-3.99249300
O	1.59641500	-3.99020700	-3.06710000
C	-2.96164700	-3.77498300	0.45302900
H	-3.44609100	-3.57483000	-1.62228200
C	-0.75476700	-4.72386200	0.90232700
O	0.67001200	-5.35618600	-0.92981400
C	-1.86135600	0.12951100	-4.99571000
C	0.44330300	-0.81539900	-4.48954900
H	2.38010600	-1.65443200	-4.04152600
C	2.29488300	-3.87955900	-1.90381700
C	-4.28192300	-3.23189900	0.99114000
C	-1.95850000	-4.18440200	1.33998700
H	-0.00220800	-5.04933500	1.60806500
C	1.80796100	-4.62080700	-0.76635700
H	-1.26381700	0.72306700	-5.68769500
C	-2.25653400	1.05745200	-3.85302500
O	0.96680100	0.31223700	-5.13404100
C	-4.24976700	-1.70966900	1.08344700
H	-4.36993400	-3.59749500	2.01439000
O	-2.21149100	-4.06546300	2.70818900
C	-3.36455900	0.80560200	-3.04106000
C	-1.49353200	2.19404400	-3.55622800
C	1.60447700	1.25661100	-4.39420200
C	4.07207300	-3.17284900	-0.67344900

C	-4.58559800	-0.90214500	-0.00709200
C	-3.89776300	-1.06128300	2.27659600
C	-1.54304800	-3.08137500	3.37717600
C	3.59084500	-3.90605000	0.45284700
C	-3.74070200	1.63637300	-1.98225000
H	-3.97310900	-0.06383400	-3.25568200
C	-1.80830700	3.03947800	-2.49567800
O	-0.43986600	2.51241400	-4.40998100
C	0.84764300	2.41383800	-3.97788600
C	-4.61091100	0.49310000	0.05654900
H	-4.85650500	-1.38202900	-0.93965700
C	-3.90189900	0.32793200	2.38648700
O	-3.60169200	-1.83298900	3.40104900
C	-2.28887500	-1.91884600	3.78242800
C	-4.98244200	1.35610500	-1.14325000
C	-2.92626200	2.74637700	-1.72127100
H	-1.20934000	3.92052900	-2.30263200
C	3.46688400	2.14459400	-3.43312300
C	-4.26255000	1.08290900	1.27583900
H	-3.64142000	0.79983300	3.32542400
C	0.31801000	-2.23192200	4.38113400
H	-5.32042000	2.31726700	-0.74957600
O	-3.29217500	3.66012000	-0.72641600
C	2.70827200	3.26669900	-2.98072700
O	-4.32182700	2.48097600	1.39636400
C	-0.44347200	-1.12168100	4.85739900
C	4.30891300	-3.86567700	1.67021200
C	5.27129200	-2.43214600	-0.55886800
C	3.35081800	4.29616100	-2.25567800
C	4.85594200	2.09057600	-3.17131000
C	0.17909100	-0.15510300	5.68184600
C	1.69345400	-2.32303300	4.69454100
C	-6.14687000	0.77771700	-1.96010000
H	-7.02646100	0.66436200	-1.32157100
H	-5.92628900	-0.20214000	-2.39095400
C	-3.06581600	-0.41639400	-5.77506500
H	-3.66222200	0.40926900	-6.17220600
H	-3.72763700	-1.03906900	-5.16694100
C	-2.39540700	-4.98840600	-3.70996700
H	-2.12389600	-5.08873200	-4.76403200
H	-3.25812500	-4.31860800	-3.65445000
C	-5.49681100	-3.75246900	0.20793100
H	-5.51504800	-4.84496700	0.23375500
H	-5.49276600	-3.44965000	-0.84282200
H	-6.42140400	-3.37923800	0.65606600
H	-2.71487900	-5.96574200	-3.33909400
H	-2.71973700	-1.02801600	-6.61203700
H	-6.39880800	1.45150000	-2.78293300
C	2.27818400	-1.36231500	5.49208000
C	1.51590900	-0.28201100	5.99669500
H	1.99215400	0.45586700	6.63419100
H	3.33185000	-1.43484700	5.74181500
H	-0.42154500	0.67035300	6.04834500
H	2.25071500	-3.16882400	4.30655400
C	5.95702300	-2.40986800	0.64017200
H	5.62899900	-1.90382000	-1.43649600
C	5.47099600	-3.12582800	1.76056100
H	3.92343900	-4.43579500	2.50865300
C	5.46475800	3.12215200	-2.48627600
H	5.41123900	1.23483100	-3.53956300
C	4.70888000	4.22572300	-2.02460300
H	2.75603600	5.14283300	-1.93016600
H	5.20566200	5.03454600	-1.49996500
H	6.53662000	3.09562100	-2.31644200
H	6.02618400	-3.10367300	2.69283600
H	6.88520000	-1.85149100	0.72041200
P	-3.05593800	3.37839200	0.86810800
N	-3.51734600	4.87315500	1.46130700
C	-2.82783100	6.04822600	0.91364400
C	-3.75446500	4.95556500	2.90958000
H	-2.74603900	5.96356700	-0.16945700
H	-1.82628800	6.18489600	1.34823500
H	-3.42710500	6.93337700	1.14276300
H	-4.32328500	4.08909300	3.24538100
H	-4.34874200	5.85160600	3.10713200
H	-2.81820900	5.02246000	3.48244700
Au	-1.08411500	2.41374100	1.59281600
N	-1.75893800	-0.97556800	4.50385200
N	-0.27610200	-3.21448700	3.63870300
N	2.43124800	-4.62881500	0.37429300
N	3.38592700	-3.17395900	-1.85755500
N	2.86988300	1.12685600	-4.12533200
N	1.37243700	3.37031500	-3.26952500
C	1.76649400	1.69745100	2.37579300
C	0.47837500	1.53176300	2.56812400
H	0.21763900	0.75959200	3.28586500
C	2.69037700	2.50945900	1.51021700
C	3.84957300	1.53353600	1.22766200
H	3.05556400	3.39756000	2.03752400
H	2.18851500	2.83136500	0.59836000
C	3.77427200	0.64199300	2.44733400
O	2.68878300	0.72868200	3.09278800
O	4.70758700	-0.14754600	2.83898800
H	5.50944600	0.05790500	2.26720700
C	3.60045900	0.66878000	-0.07761500
H	4.38552000	-0.08886700	-0.13229800
H	3.74038000	1.35631300	-0.91263900
C	5.27119000	2.06314400	1.15783500
O	6.22385500	1.30711300	1.34206600
O	5.37467900	3.34608500	0.89359600
C	6.72481200	3.87995300	0.82500800
H	6.60076100	4.94277700	0.63227300
H	7.23560000	3.70919500	1.77320500
H	7.27137200	3.39228900	0.01767200
C	2.23388200	0.03746800	-0.11815700
C	1.20775900	0.58713900	-0.89333200
C	1.95956700	-1.08779400	0.66742000
C	-0.07241100	0.03083300	-0.87707400
H	1.40511100	1.46286800	-1.49860000
C	0.68242300	-1.63790600	0.69708600
H	2.74290200	-1.54029500	1.26608000
C	-0.33991900	-1.07975300	-0.07529000
H	-0.85690700	0.46181700	-1.48722000
H	0.49172000	-2.50270100	1.31708100
H	-1.33307800	-1.51415200	-0.05900100

1a-TS1$_{5syn}$H^{+}⊂AuCav

C	-1.96707700	0.35570200	4.74169100
C	-0.73385800	-0.38331500	4.23298500
C	-2.77088700	0.93017500	3.58162000
H	-1.59800300	1.20809900	5.31234900
C	-0.75532500	-1.75082900	3.95213300
C	0.49264600	0.27972400	4.08317000
C	-3.72665800	0.18008600	2.89095300
C	-2.58092100	2.25486200	3.17340800
C	0.38132600	-2.47639600	3.58743400
H	-1.69044500	-2.28403500	4.06869600
C	1.65424300	-0.39301600	3.70646800
O	0.55915300	1.61500100	4.48599100
C	-4.49188700	0.70188400	1.84482600
H	-3.88897300	-0.84938600	3.18809100
C	-3.30885400	2.82257100	2.13135300
O	-1.68811200	3.04297800	3.90698300
C	0.32050300	-3.98329300	3.35029300
C	1.58418800	-1.76519700	3.46683000
H	2.60261900	0.12716700	3.65751400
C	0.66778300	2.64342200	3.60332300
C	-5.52617700	-0.14097900	1.10042700
C	-4.25188300	2.03294300	1.47725200
H	-3.16544700	3.85841300	1.85131100
C	-0.51877000	3.42022000	3.32245600
H	1.32363200	-4.36777800	3.53503900
C	0.01303900	-4.24411600	1.88076300
O	2.75951700	-2.48697500	3.24719100
C	-4.81225100	-0.90179800	-0.01011900
H	-6.21002400	0.55630900	0.61639700
O	-5.04337000	2.58749400	0.47129300
C	-1.28550800	-4.20837900	1.36446400
C	1.06028500	-4.45505300	0.97664000
C	3.42963600	-2.43576500	2.06223600

C	1.89641300	4.09414700	2.34395100
C	-4.22154500	-2.15071400	0.20120600
C	-4.67871300	-0.33363400	-1.28447700
C	-4.45685700	2.88278500	-0.72706600
C	0.72479900	4.86281600	2.07211500
C	-1.55954200	-4.32567500	-0.00265100
H	-2.11237300	-4.04860700	2.04692900
C	0.84092100	-4.58711800	-0.38863400
O	2.35828500	-4.50713700	1.47676700
C	3.21262300	-3.50352600	1.11764600
C	-3.48002000	-2.82419900	-0.77554400
H	-4.35994300	-2.62906000	1.16296800
C	-3.89742000	-0.93213100	-2.26928100
O	-5.38727300	0.83152000	-1.56787200
C	-4.67216200	1.97245300	-1.81916900
C	-2.97463700	-4.25446500	-0.57084200
C	-0.46309800	-4.49098000	-0.85497400
H	1.67484600	-4.69746600	-1.06973800
C	5.01409500	-1.56283000	0.67498000
C	-3.28784400	-2.15001900	-1.98961100
H	-3.78768900	-0.46116200	-3.23857800
C	-3.23547400	4.22761800	-2.10115800
H	-2.92720200	-4.70095100	-1.56722900
O	-0.65165300	-4.41581300	-2.24800400
C	4.75175200	-2.58456600	-0.28406600
O	-2.50514000	-2.71897800	-3.00776200
C	-3.48318400	3.34276600	-3.19519200
C	0.81708700	6.01310700	1.25508200
C	3.13545300	4.49940100	1.79645100
C	5.46871200	-2.60293300	-1.50240100
C	6.01651400	-0.60098000	0.40444900
C	-2.92397300	3.62646600	-4.46161000
C	-2.42180200	5.36629400	-2.29585500
C	-3.95749000	-5.10817100	0.24770400
H	-4.95104900	-5.08106500	-0.20662600
H	-4.05476400	-4.76943600	1.28233900
C	-0.63859300	-4.71311500	4.30112500
H	-0.57452600	-5.79162600	4.13540600
H	-1.68424900	-4.42342700	4.16525400
C	-2.82507900	-0.49349200	5.69158300
H	-2.21934300	-0.85031100	6.52867300
H	-3.26843600	-1.36799600	5.20756900
C	-6.35422600	-1.03914900	2.02878000
H	-6.87437600	-0.42876200	2.77125400
H	-5.75081800	-1.77149900	2.57241100
H	-7.10015900	-1.59033500	1.45007100
H	-3.64474200	0.11043400	6.08899400
H	-0.37027400	-4.50603500	5.34027500
H	-3.61332200	-6.14504100	0.27334000
C	-1.87919400	5.61475600	-3.53830600
C	-2.13376200	4.74414900	-4.62557800
H	-1.70172300	4.96359500	-5.59662300
H	-1.25394900	6.48858200	-3.69116500
H	-3.13167200	2.94380300	-5.27840100
H	-2.24389100	6.01779700	-1.44736400
C	3.19852800	5.62288600	0.99623800
H	4.01406100	3.90687200	2.02228800
C	2.03554000	6.38197100	0.72302800
H	-0.08930000	6.57698000	1.06432400
C	6.71297300	-0.64815700	-0.78689900
H	6.22870800	0.14397200	1.16419300
C	6.43107800	-1.64739900	-1.74864600
H	5.24535200	-3.38884000	-2.21523300
H	6.98498900	-1.66286300	-2.68111200
H	7.48885500	0.08191500	-0.99267300
H	2.10781400	7.26423700	0.09564200
H	4.15073500	5.93803500	0.58058300
P	-0.89342000	-2.88096800	-2.78389100
N	-0.39235000	-3.02518400	-4.36904400
C	0.98679900	-3.49100900	-4.57384100
C	-0.81232700	-1.98553900	-5.31527500
H	1.20352400	-4.31912800	-3.89954400
H	1.72351800	-2.69015900	-4.41683400
H	1.07626100	-3.85490000	-5.60071400
H	-1.85525600	-1.72158600	-5.14281100
H	-0.72466500	-2.38596500	-6.32881200
H	-0.18670500	-1.08324500	-5.24421300
Au	0.03217600	-1.43098800	-1.23014700
N	-4.21259300	2.19893800	-3.01481000
N	-3.75274900	3.96788700	-0.86197800
N	-0.48989200	4.48234900	2.57276000
N	1.83119000	2.96099400	3.11223500
N	4.30377400	-1.49573300	1.84460000
N	3.82801400	-3.55852900	-0.02526700
C	2.04829000	0.32344900	0.06200400
C	0.94262600	-0.26967400	0.25045500
H	0.34933700	-0.07602400	1.14187100
C	3.13602700	1.28447100	0.27865500
C	3.72885700	1.76247300	-1.05926500
H	3.91204900	0.83157400	0.89748600
H	2.71097700	2.11906100	0.83994100
C	3.67755100	0.53729200	-1.97356900
O	2.90637500	-0.36908300	-1.66831600
O	4.37256500	0.52503200	-3.08418700
H	5.04830800	1.25299000	-3.02251200
C	2.88278200	2.91358000	-1.75439000
H	3.03227400	3.81442000	-1.15320900
H	3.31897400	3.09018300	-2.74145400
C	5.14736000	2.29787500	-0.97928200
O	5.87339800	2.34916100	-1.96568500
O	5.48217100	2.76362000	0.21280700
C	6.80667200	3.34608600	0.32756700
H	6.86715400	3.71704300	1.34832600
H	7.56236300	2.58045300	0.14610700
H	6.92155400	4.15393200	-0.39607900
C	1.42032300	2.57562200	-1.83386900
C	0.90948600	1.77200200	-2.85997400
C	0.57158000	2.98463900	-0.80007600
C	-0.40400000	1.31146000	-2.79372600
H	1.54534700	1.48152900	-3.69176500
C	-0.73163100	2.50818600	-0.71749300
H	0.95058100	3.65823400	-0.04452900
C	-1.20731700	1.63883700	-1.69821500
H	-0.80295000	0.68859300	-3.58712800
H	-1.37093600	2.81015200	0.10427800
H	-2.20355600	1.22595000	-1.62485000

1a-Int$_{5syn}$H^{+}⊂AuCav

C	-1.90657100	3.65513300	3.50934900
C	-1.57924500	2.17596100	3.69254800
C	-1.78473600	4.02602100	2.03509500
H	-1.13013600	4.21045900	4.03615700
C	-2.57411900	1.19730300	3.63087500
C	-0.27175700	1.73943300	3.95237200
C	-2.82488300	3.85425300	1.11636800
C	-0.57636000	4.52749100	1.54186200
C	-2.33222100	-0.16115900	3.84654500
H	-3.59338500	1.51495300	3.45064400
C	0.03153700	0.39075100	4.13135300
O	0.71956700	2.69724500	4.18650400
C	-2.68991300	4.14776700	-0.24551400
H	-3.77477400	3.47249200	1.47287300
C	-0.38514700	4.83082500	0.20200300
O	0.45614400	4.75270400	2.45550100
C	-3.46887600	-1.17920800	3.88485900
C	-1.00261600	-0.54119900	4.07611700
H	1.03955800	0.08163500	4.37801300
C	1.72769600	2.90809000	3.29669500
C	-3.83652200	3.97056700	-1.23925100
C	-1.44198900	4.61814700	-0.67400300
H	0.56395800	5.20557000	-0.15555000
C	1.57473800	3.98951400	2.35164400
H	-3.11770800	-2.00571400	4.50326300
C	-3.71985700	-1.75407800	2.49633300
O	-0.73971900	-1.87567000	4.40463600
C	-3.90144600	2.53343900	-1.74805400
H	-3.58960200	4.58879100	-2.10311000
O	-1.25647700	4.89235600	-2.03204600
C	-4.50804400	-1.10684000	1.53957000
C	-3.11912300	-2.96043900	2.12136300

C	-0.13658700	-2.70731600	3.51408700
C	3.82498000	2.53071500	2.49038500
C	-4.62856300	1.53669500	-1.08783000
C	-3.21974900	2.15986100	-2.91462000
C	-0.47787200	3.98917800	-2.69976400
C	3.63658400	3.54095900	1.50007200
C	-4.68662900	-1.59981900	0.24156300
H	-4.98042000	-0.16855900	1.80640300
C	-3.28012900	-3.50311800	0.85476800
O	-2.31108700	-3.61623500	3.05071500
C	-0.97276400	-3.63157100	2.78638400
C	-4.69184800	0.21114400	-1.53286800
H	-5.17556400	1.80658600	-0.19264800
C	-3.24604200	0.85674800	-3.39434400
O	-2.51070400	3.12753900	-3.62488100
C	-1.14579400	3.05022300	-3.55778000
C	-5.51179200	-0.85996300	-0.81165500
C	-4.04174600	-2.80257800	-0.07006600
H	-2.77408300	-4.41972400	0.58217900
C	1.70068600	-3.63529100	2.53568300
C	-3.97119200	-0.09553600	-2.69400600
H	-2.69750300	0.59503300	-4.28950600
C	1.52474300	3.05579500	-3.24528700
H	-5.78088400	-1.60090200	-1.56853900
O	-4.04943000	-3.28654900	-1.38925600
C	0.86785300	-4.50263500	1.77009100
O	-3.94910900	-1.40777000	-3.19482500
C	0.86342900	2.13975100	-4.11905000
C	4.67679900	3.82737500	0.58597600
C	5.07196700	1.87406800	2.58067800
C	1.45506500	-5.42577000	0.87427600
C	3.10655000	-3.74492600	2.41837900
C	1.63012500	1.21511000	-4.86592700
C	2.93314600	3.00790200	-3.11880300
C	-6.82691600	-0.31203500	-0.23622100
H	-7.41325000	0.16423300	-1.02603300
H	-6.67103800	0.42811800	0.55289100
C	-4.74783000	-0.62904700	4.53384100
H	-5.49318100	-1.42438500	4.61577100
H	-5.20176500	0.18707200	3.96511000
C	-3.25642700	4.05930800	4.11833000
H	-3.28817300	3.79325600	5.17817300
H	-4.11020300	3.58050600	3.63094100
C	-5.17877300	4.46785300	-0.68201400
H	-5.09801600	5.52093000	-0.40093600
H	-5.50600600	3.91692900	0.20421300
H	-5.96067200	4.36989900	-1.43979600
H	-3.39364600	5.13983300	4.02770100
H	-4.53040100	-0.25273300	5.53673800
H	-7.41502700	-1.12822900	0.19073200
C	3.65861000	2.10217300	-3.86352900
C	3.00381800	1.20848000	-4.74571300
H	3.59661000	0.51525600	-5.33276000
H	4.73920700	2.06790600	-3.77840900
H	1.10851700	0.54231100	-5.53822100
H	3.40814200	3.70861300	-2.44075400
C	6.08437000	2.19239600	1.69813100
H	5.19751900	1.13830200	3.36500400
C	5.88230500	3.16220400	0.68709300
H	4.50607900	4.60061200	-0.15520900
C	3.65735100	-4.67622500	1.56001400
H	3.71910100	-3.10742000	3.04716600
C	2.82806300	-5.50990300	0.77355500
H	0.79768100	-6.07061700	0.30144500
H	3.27937400	-6.22869000	0.09793900
H	4.73532400	-4.77009900	1.48222700
H	6.68879900	3.39615400	-0.00020900
H	7.05113300	1.70548200	1.78470400
P	-3.01802100	-2.48542700	-2.38940000
N	-2.80413700	-3.63744700	-3.57992900
C	-2.29775500	-4.94598400	-3.14927100
C	-2.26616000	-3.18519000	-4.86836400
H	-2.79085800	-5.25088900	-2.22642500
H	-1.20812200	-4.94239700	-2.99771200
H	-2.53845700	-5.68029400	-3.92279800
H	-2.73261600	-2.24392900	-5.15736000
H	-2.51140900	-3.93480200	-5.62565400
H	-1.17353900	-3.05970900	-4.84316700
Au	-1.35230200	-1.46014000	-1.09379900
N	-0.50045800	2.15194800	-4.24155300
N	0.81169600	3.98133000	-2.53443100
N	2.47824100	4.26813800	1.45969100
N	2.81853400	2.20179900	3.36385300
N	1.15930200	-2.70864600	3.38734200
N	-0.49098700	-4.47300900	1.92133800
C	1.29745700	-0.98055300	0.27487800
C	0.03451100	-0.61546500	0.16789600
H	-0.25289300	0.18551500	0.84988700
C	2.51033100	-0.58800800	1.08683800
C	3.68675600	-1.04733200	0.19956300
H	2.51281600	-1.09070300	2.05731800
H	2.53534100	0.48462400	1.27035800
C	3.03549800	-2.14267200	-0.60069500
O	1.76483900	-2.07868400	-0.62579600
O	3.65743000	-3.04941400	-1.25277100
H	4.62297200	-2.98494600	-0.94684200
C	4.08894300	0.12714300	-0.79780400
H	3.17096100	0.42910300	-1.31196900
H	4.38617200	0.95431700	-0.15050600
C	4.94453400	-1.60757900	0.83105700
O	5.62040500	-2.45599900	0.25149200
O	5.25846300	-1.07931100	1.99208800
C	6.49941400	-1.53424500	2.59385700
H	6.61383800	-0.93739200	3.49574000
H	6.42280400	-2.59510200	2.83630400
H	7.32558000	-1.37221400	1.90086000
C	5.17999100	-0.19936600	-1.77899200
C	6.50630300	0.14769600	-1.49458800
C	4.89494000	-0.85498100	-2.98377300
C	7.52817300	-0.15269200	-2.39419400
H	6.73156600	0.66677300	-0.56813500
C	5.91547000	-1.16255700	-3.88225400
H	3.86729200	-1.09877200	-3.23411200
C	7.23475500	-0.81179000	-3.58906200
H	8.55100300	0.12933100	-2.16553000
H	5.68061100	-1.66725400	-4.81416300
H	8.02861100	-1.04557600	-4.29099000

1a-TS1$_6$H$^+$⊂AuCav

C	-2.53555200	3.73191600	3.16292300
C	-1.70872600	2.53250500	3.62120800
C	-2.67830000	3.75409900	1.64436300
H	-1.95707200	4.61754400	3.42752700
C	-2.28465000	1.29632800	3.92562300
C	-0.32177500	2.64696000	3.78752100
C	-3.68527300	3.04559100	0.97997300
C	-1.79125200	4.49553400	0.85339400
C	-1.54599500	0.21394800	4.41332600
H	-3.35510900	1.18042200	3.80359300
C	0.46041500	1.59822000	4.25855100
O	0.26304000	3.89097900	3.55980600
C	-3.82358000	3.04502400	-0.41158800
H	-4.38645600	2.46818600	1.57143300
C	-1.89537800	4.53996500	-0.53261800
O	-0.79423100	5.24231600	1.48244600
C	-2.19109900	-1.12692900	4.75075000
C	-0.16817500	0.39922900	4.57314200
H	1.52567900	1.72386300	4.40114700
C	1.04920800	4.05459900	2.45765100
C	-4.91367300	2.25446500	-1.13027000
C	-2.89717600	3.79692800	-1.14463700
H	-1.19697900	5.12143300	-1.11943400
C	0.49107200	4.79228500	1.35556600
H	-1.52287400	-1.62792100	5.45131900
C	-2.23423100	-1.99082900	3.49572100
O	0.57156000	-0.63822500	5.15359400
C	-4.45703800	0.81786200	-1.36500200
H	-5.02425700	2.70615500	-2.11633100
O	-3.01596600	3.80770100	-2.53731200
C	-3.28589600	-1.91337100	2.57922400

C	-1.18947600	-2.87572900	3.19696000
C	1.47935700	-1.29846800	4.38725800
C	3.01224500	3.85980900	1.32808600
C	-4.64564800	-0.18202900	-0.40626700
C	-3.82702400	0.44649200	-2.56155600
C	-2.06100500	3.13244700	-3.23992100
C	2.46232500	4.59893900	0.23716000
C	-3.33884000	-2.67871400	1.41101200
H	-4.10425500	-1.23657900	2.79058700
C	-1.18343800	-3.64515000	2.03638700
O	-0.16629100	-3.02547000	4.12698900
C	1.08045600	-2.56263600	3.81950400
C	-4.25625300	-1.51018000	-0.59999700
H	-5.13770800	0.08116000	0.52187000
C	-3.39763100	-0.85869800	-2.79199600
O	-3.68875500	1.39587500	-3.57062000
C	-2.42827900	1.86940600	-3.82073300
C	-4.51235500	-2.59367000	0.44187500
C	-2.25707600	-3.53071200	1.16224400
H	-0.36934800	-4.33169800	1.84125500
C	3.55120600	-1.55989000	3.48132000
C	-3.61891100	-1.81294100	-1.80765600
H	-2.92922500	-1.11820000	-3.73298800
C	0.02492300	2.92738400	-4.13422300
H	-4.54893800	-3.54484000	-0.09439100
O	-2.27806300	-4.36871700	0.03489900
C	3.14488600	-2.79593700	2.89148500
O	-3.25948400	-3.14470800	-2.08417000
C	-0.36745000	1.71365400	-4.77769200
C	3.25666700	4.84997600	-0.90550500
C	4.34934600	3.40457600	1.25599100
C	4.07146800	-3.54241100	2.12931200
C	4.87815500	-1.10512700	3.30201200
C	0.55282500	1.04441300	-5.61946000
C	1.34426800	3.40808500	-4.29534800
C	-5.86553900	-2.43606000	1.15073800
H	-6.67339200	-2.43979300	0.41486900
H	-5.94606100	-1.50873400	1.72312500
C	-3.55892300	-0.98757400	5.43313700
H	-3.95300900	-1.97400000	5.69171100
H	-4.30415000	-0.48747900	4.80850700
C	-3.88786200	3.83194100	3.88433900
H	-3.73400600	3.86888200	4.96590200
H	-4.55107300	2.98785900	3.67473200
C	-6.27472900	2.34167400	-0.42429200
H	-6.58469300	3.38662700	-0.34517200
H	-6.26197900	1.92724900	0.58757800
H	-7.03163600	1.79779600	-0.99562100
H	-4.40866500	4.74226100	3.57637100
H	-3.45718500	-0.40359400	6.35119000
H	-6.02222000	-3.26611400	1.84407900
C	2.22952100	2.72376100	-5.09997500
C	1.82787800	1.54816600	-5.77668100
H	2.53725500	1.03325400	-6.41559100
H	3.24523200	3.08469700	-5.21836100
H	0.22384900	0.14436000	-6.12892500
H	1.62151500	4.31961100	-3.77772600
C	5.10610500	3.67005800	0.13294500
H	4.74992200	2.86239300	2.10603000
C	4.55575900	4.38920300	-0.95526700
H	2.81578000	5.41692300	-1.71824900
C	5.76958000	-1.85992400	2.56689200
H	5.16186100	-0.16525800	3.76253300
C	5.36447200	-3.08324300	1.98247600
H	3.74637600	-4.48681200	1.70583800
H	6.08248000	-3.67761100	1.42912100
H	6.79082400	-1.51515000	2.44167400
H	5.16577800	4.58038900	-1.83152900
H	6.13775900	3.33396600	0.08557700
P	-1.91647300	-3.80656800	-1.44855400
N	-1.88079600	-5.24519600	-2.29188000
C	-0.98074300	-6.28830800	-1.77999900
C	-1.89660200	-5.14983500	-3.75922400
H	-1.07397700	-6.36566900	-0.69730500
H	0.06956600	-6.09853600	-2.04674500
H	-1.28247100	-7.24276000	-2.21835900
H	-2.63724400	-4.41772900	-4.07898000
H	-2.18451400	-6.12506900	-4.15943200
H	-0.91224000	-4.87971100	-4.16853600
Au	-0.05818600	-2.45069400	-1.58985400
N	-1.61318800	1.18382900	-4.56693900
N	-0.86528200	3.62757200	-3.36805500
N	1.17125200	5.05100700	0.27853400
N	2.26444400	3.59335900	2.44210200
N	2.67288300	-0.81216900	4.21713800
N	1.87383600	-3.27482900	3.07534500
C	1.84591500	-1.57954900	-2.01976700
C	1.47266400	-0.75302900	-2.88547600
H	0.91944600	-0.15110000	-3.57802100
C	3.18241400	-1.89735800	-1.42761000
C	4.01028200	-0.62999200	-1.11370000
H	3.71113900	-2.52634900	-2.14820100
H	3.09049100	-2.47939100	-0.50922100
C	4.00677400	0.41805400	-2.26240800
O	3.25307800	0.39096800	-3.22459700
O	4.86968700	1.41455400	-2.14841600
H	5.49728800	1.23129300	-1.40964000
C	3.49178900	0.05232000	0.20893700
H	3.70009300	-0.64863900	1.01702000
H	4.09235400	0.94361100	0.39799700
C	5.48825500	-0.96343300	-0.86443300
O	6.29522400	-0.10251400	-0.53992500
O	5.80942900	-2.23491600	-1.05531300
C	7.21508100	-2.55944700	-0.91429700
H	7.27621900	-3.63598700	-1.05937500
H	7.79132700	-2.02948300	-1.67427200
H	7.56962100	-2.27191100	0.07549500
C	2.02671000	0.39885100	0.19108500
C	1.09119700	-0.41214500	0.84470200
C	1.57233100	1.51623200	-0.51729800
C	-0.27597400	-0.12223400	0.78253500
H	1.43135000	-1.27859800	1.39968700
C	0.21194700	1.80474400	-0.58861200
H	2.28111800	2.16546400	-1.01999000
C	-0.71876900	0.98530800	0.05768500
H	-0.98659400	-0.74773600	1.30981200
H	-0.11654800	2.67956100	-1.13308800
H	-1.77570100	1.22066300	0.01290800

1a-Int$_6$H$^+$⊂AuCav

C	-0.70180500	4.21248200	3.41965100
C	-0.62228700	2.72726000	3.76552700
C	-0.63204100	4.43271100	1.91183700
H	0.19578500	4.66938900	3.83722800
C	-1.75775300	1.92263600	3.89630500
C	0.61881700	2.12348000	4.00937600
C	-1.76323800	4.33063500	1.09601100
C	0.58106500	4.73961000	1.28305400
C	-1.69777800	0.58167000	4.28854000
H	-2.72966700	2.36696900	3.71746200
C	0.73583000	0.79002300	4.38607800
O	1.75312000	2.93621500	3.98000400
C	-1.72090700	4.50370600	-0.29041600
H	-2.71353600	4.09089000	1.55951700
C	0.67213900	4.95173900	-0.08881800
O	1.73538300	4.84049600	2.06467000
C	-2.94710800	-0.27938600	4.45892300
C	-0.42861700	0.04308400	4.52818600
H	1.70479000	0.35616100	4.59527000
C	2.65061700	2.80713900	2.96518200
C	-2.95916400	4.36115400	-1.17013400
C	-0.47625500	4.80077400	-0.85835700
H	1.62450700	5.17403900	-0.55157700
C	2.64477000	3.82704900	1.94536000
H	-2.67821900	-1.08395100	5.14356000
C	-3.30377900	-0.93903600	3.13116500
O	-0.35777900	-1.26507500	5.02237500
C	-3.21738800	2.89451200	-1.50031600
H	-2.72433100	4.85053300	-2.11554000
O	-0.40377200	4.94691300	-2.24591600

C	-4.06522800	-0.28063400	2.16185900
C	-2.83785100	-2.22133200	2.81184900
C	0.15948400	-2.24076300	4.23126900
C	4.40858200	1.79562400	1.93489400
C	-4.01334400	2.08205800	-0.68807400
C	-2.67413500	2.31040000	-2.65377300
C	0.21747500	3.94488100	-2.93713900
C	4.39068100	2.79343300	0.91397900
C	-4.37930500	-0.84546400	0.92286600
H	-4.42973100	0.71507700	2.38253300
C	-3.11539400	-2.82546800	1.58801900
O	-2.09982200	-2.91581200	3.76882600
C	-0.76714900	-3.10700900	3.54328000
C	-4.30792800	0.75061100	-0.99379800
H	-4.45011100	2.51509700	0.20319400
C	-2.92269100	0.98093200	-2.99181200
O	-1.96400100	3.12449100	-3.53359000
C	-0.60746900	2.99698100	-3.64129500
C	-5.21453100	-0.10261000	-0.11378100
C	-3.87677500	-2.12572900	0.65904000
H	-2.74457200	-3.81965600	1.37368700
C	1.89620900	-3.43181000	3.36745400
C	-3.74072300	0.22247800	-2.15971600
H	-2.51874200	0.56793200	-3.90778500
C	2.08262600	2.91664100	-3.75034300
H	-5.66082400	-0.86005600	-0.76213400
O	-4.20244700	-2.76115100	-0.54557800
C	0.98095600	-4.26221400	2.65203100
O	-4.09696200	-1.07133800	-2.56777700
C	1.26481100	2.03425700	-4.51952200
C	5.31425500	2.71611000	-0.15342000
C	5.37010200	0.76034300	1.87644500
C	1.47571900	-5.30829700	1.83952500
C	3.28491000	-3.68490500	3.27483500
C	1.87332500	1.10684100	-5.39647900
C	3.49001500	2.81520800	-3.83607800
C	-6.37500700	0.68798000	0.50732500
H	-6.96709600	1.16165900	-0.27970600
H	-6.04423000	1.47470700	1.18999200
C	-4.12574200	0.48333800	5.08057400
H	-4.97194200	-0.19204200	5.23165400
H	-4.47475000	1.31529000	4.46270300
C	-1.91301000	4.91006500	4.05693300
H	-1.90061800	4.76893700	5.14083700
H	-2.87040200	4.53364600	3.68564000
C	-4.18625200	5.06766500	-0.57472500
H	-3.97064100	6.13014600	-0.43699500
H	-4.48322400	4.66655600	0.39831800
H	-5.04161500	4.97174900	-1.24875700
H	-1.87901700	5.98194700	3.84578100
H	-3.83303100	0.89490900	6.04977800
H	-7.02354200	0.01411500	1.07266100
C	4.05949600	1.89527800	-4.69096700
C	3.24859900	1.04865000	-5.48344400
H	3.71716400	0.34611500	-6.16526500
H	5.13956200	1.82555700	-4.76867400
H	1.23209600	0.46370900	-5.98972000
H	4.08740500	3.49319100	-3.23668700
C	6.26747700	0.70980300	0.82715400
H	5.37720400	0.02684100	2.67591400
C	6.23431900	1.68783300	-0.19519400
H	5.27737300	3.48634300	-0.91582000
C	3.74089400	-4.72913800	2.49660600
H	3.95738000	-3.05105300	3.84271800
C	2.83418300	-5.53872200	1.77218300
H	0.76081400	-5.92953100	1.31078100
H	3.20915300	-6.36164800	1.17289000
H	4.80410900	-4.94284700	2.45266000
H	6.94877600	1.63516800	-1.01043600
H	7.00822600	-0.08283200	0.78677200
P	-3.40857100	-2.41376400	-1.93474400
N	-4.11165800	-3.56304600	-2.92733200
C	-3.99483100	-4.96116500	-2.49098800
C	-3.92250500	-3.38533200	-4.37404100
H	-4.21126700	-5.04044600	-1.42602000
H	-2.99839500	-5.38041900	-2.69523000
H	-4.73685600	-5.55144800	-3.03491600
H	-4.08852200	-2.34402400	-4.64777100
H	-4.66539800	-3.99767000	-4.89179800
H	-2.92076700	-3.69584000	-4.70527800
Au	-1.10877600	-2.22989700	-1.86254600
N	-0.10076600	2.07353100	-4.40542900
N	1.51599900	3.87830300	-2.95955000
N	3.47753100	3.81323500	0.94791200
N	3.50135200	1.82392900	2.95953300
N	1.44728700	-2.39866600	4.14323600
N	-0.37097900	-4.06638900	2.76000500
C	0.92924600	-1.97079300	-2.06276800
C	1.27828800	-0.84220200	-2.66139600
H	0.68549700	-0.07951000	-3.13394800
C	2.01037300	-2.86374200	-1.49795200
C	3.26637100	-2.07662200	-1.05503200
H	2.28049000	-3.60913700	-2.25599800
H	1.65285200	-3.43102500	-0.63641700
C	3.56912500	-0.94790500	-2.02062600
O	2.66979300	-0.40441700	-2.73480900
O	4.74994100	-0.45531700	-2.13500600
H	5.37467000	-1.05232500	-1.59246900
C	3.05961000	-1.42361200	0.38544100
H	2.98015100	-2.26892200	1.07276800
H	3.96640600	-0.87016600	0.63803200
C	4.52667700	-2.92752000	-0.92206500
O	5.65718700	-2.43406200	-0.92878400
O	4.29670500	-4.20933700	-0.75691500
C	5.46494600	-5.05700900	-0.58944200
H	5.07006600	-6.06269500	-0.46987500
H	6.09872800	-4.98602500	-1.47396500
H	6.02410400	-4.74319900	0.29214900
C	1.84451900	-0.54279300	0.47105900
C	0.63340200	-1.04604500	0.95310200
C	1.88571400	0.76516400	-0.02343500
C	-0.52310400	-0.26741900	0.91973900
H	0.58717900	-2.06184300	1.32396300
C	0.72791000	1.53507100	-0.07880500
H	2.81766000	1.18033000	-0.39206100
C	-0.48231100	1.02092700	0.39128900
H	-1.45417900	-0.66867200	1.29194600
H	0.77590600	2.53574700	-0.48116600
H	-1.38185300	1.62203000	0.35053300

1a-Int$_{5anti}$'⊂AuCav

C	-1.89159400	4.52727100	2.44729800
C	-1.14367100	3.36021100	3.08809700
C	-2.16045900	4.26553800	0.96957700
H	-1.21234000	5.37928000	2.48702000
C	-1.81253700	2.28212400	3.67069100
C	0.25740700	3.35040300	3.14962000
C	-3.27779200	3.55291200	0.52152000
C	-1.26658700	4.73257100	-0.00138900
C	-1.15314500	1.24770200	4.33988200
H	-2.89498700	2.25994000	3.62476400
C	0.96184600	2.33275000	3.78684700
O	0.94403600	4.45913300	2.65673800
C	-3.51086800	3.28224700	-0.83053200
H	-3.98736500	3.18668200	1.25490600
C	-1.45523600	4.49136200	-1.35777500
O	-0.17340800	5.49291100	0.41408900
C	-1.91406700	0.09193000	4.98018100
C	0.24471900	1.30223000	4.38767900
H	2.04275400	2.36156200	3.83919800
C	1.64880600	4.34906500	1.48860700
C	-4.72707700	2.50370700	-1.32262500
C	-2.56231900	3.74872900	-1.74927400
H	-0.73886900	4.84712100	-2.08573600
C	1.06314500	4.91162700	0.30346000
H	-1.24884300	-0.34455300	5.72529100
C	-2.16228000	-0.98520600	3.93167100
O	0.92001100	0.34396400	5.15491200
C	-4.46830500	1.00173500	-1.27641700
H	-4.84949600	2.75969700	-2.37530800

O	-2.77020000	3.46921200	-3.10169000
C	-3.27681800	-0.96966100	3.09067200
C	-1.23727700	-2.02050400	3.74936900
C	1.69330900	-0.58345500	4.51899900
C	3.49999600	3.78091500	0.29552100
C	-4.68433100	0.25729900	-0.11317500
C	-4.00707200	0.30892900	-2.40544400
C	-1.95429000	2.52060300	-3.65984800
C	2.91348500	4.33271500	-0.88432600
C	-3.50273100	-1.93883800	2.10952900
H	-4.00508600	-0.17729300	3.21266200
C	-1.40212000	-2.99427500	2.76928600
O	-0.16752000	-2.09434500	4.63578500
C	1.10126400	-1.85482000	4.19017000
C	-4.48490400	-1.12321800	-0.04361700
H	-5.03571800	0.77434600	0.77173200
C	-3.77542400	-1.06468200	-2.37716800
O	-3.82759000	1.00490100	-3.60233000
C	-2.52707600	1.24436200	-3.97947800
C	-4.74666000	-1.92089200	1.22871200
C	-2.53324300	-2.94016200	1.96195500
H	-0.67045000	-3.78389500	2.65526200
C	3.67617000	-1.30237100	3.66008500
C	-4.02132200	-1.76142100	-1.19970100
H	-3.41282600	-1.57200400	-3.26208500
C	0.04860100	1.82471000	-4.49495700
H	-4.92545100	-2.95336000	0.91960200
O	-2.73959600	-3.97646600	1.04848700
C	3.06263200	-2.52628300	3.25088000
O	-3.84723500	-3.15166100	-1.18835200
C	-0.55160000	0.59800000	-4.91414800
C	3.60090300	4.23449100	-2.11617500
C	4.77041100	3.16575000	0.21516200
C	3.82200700	-3.48203000	2.53795400
C	5.04777400	-1.08879100	3.39354300
C	0.23445600	-0.35869000	-5.59797300
C	1.43427800	2.02740000	-4.68591900
C	-6.00530100	-1.45552300	1.97510300
H	-6.87547300	-1.52822100	1.31739000
H	-5.94165800	-0.42049400	2.32062500
C	-3.19277000	0.53659200	5.70334700
H	-3.67222000	-0.32197200	6.18196200
H	-3.92803500	0.99418300	5.03569400
C	-3.16206200	4.91305700	3.21934100
H	-2.91606000	5.13857500	4.26063400
H	-3.91818700	4.12275100	3.22384700
C	-6.01831300	2.90556100	-0.59388900
H	-6.19742800	3.97713200	-0.71677900
H	-5.98545100	2.70057000	0.47985000
H	-6.87062900	2.35985700	-1.00819800
H	-3.61775000	5.79910100	2.76878800
H	-2.94817800	1.27284600	6.47361600
H	-6.17631800	-2.08589700	2.85186600
C	2.18725500	1.05758000	-5.30988100
C	1.57928000	-0.12652000	-5.79057700
H	2.19112400	-0.87537500	-6.28222300
H	3.25945500	1.18560500	-5.40988700
H	-0.24214000	-1.27736000	-5.92231200
H	1.87437800	2.94391500	-4.30961200
C	5.40628800	3.05394400	-1.00316900
H	5.19812500	2.75677000	1.12400000
C	4.81699900	3.58693800	-2.17415400
H	3.13082200	4.66018800	-2.99634700
C	5.77295400	-2.04579000	2.71651900
H	5.49024800	-0.15145200	3.71122700
C	5.15456700	-3.23999100	2.27645000
H	3.33037200	-4.39595100	2.22239300
H	5.73666600	-3.97186100	1.72572700
H	6.81555600	-1.86625600	2.48290200
H	5.32663900	3.46889900	-3.12408200
H	6.34909300	2.52353500	-1.07448500
P	-2.45859500	-3.77727400	-0.56118400
N	-2.68226700	-5.38211100	-1.03071400
C	-1.77193800	-6.34838600	-0.40350100
C	-2.82801400	-5.59604600	-2.47708700
H	-1.73888800	-6.18364600	0.67340200
H	-0.75132200	-6.29157500	-0.81213800
H	-2.16149100	-7.35434100	-0.58435500
H	-3.54874600	-4.88850900	-2.88610300
H	-3.21154800	-6.60808500	-2.63572000
H	-1.87405700	-5.49322300	-3.01540700
Au	-0.59556500	-2.67172200	-1.35323700
N	-1.85809300	0.32493800	-4.61129800
N	-0.70429600	2.79289000	-3.89228700
N	1.67586400	4.91164600	-0.84288900
N	2.83217900	3.81086300	1.48920500
N	2.94487900	-0.32570400	4.27960600
N	1.75241700	-2.78393200	3.55465500
C	2.18805900	-1.72025900	-2.15940100
C	0.89127200	-1.80069800	-2.42452200
H	0.57812200	-1.23556000	-3.29739800
C	3.07175400	-2.25546400	-1.06514700
C	4.09348700	-1.11586500	-0.88220200
H	3.57610100	-3.16883300	-1.39260600
H	2.50619700	-2.47185000	-0.15858800
C	4.08477400	-0.45441100	-2.28309800
O	3.01265300	-0.88598300	-2.97356100
O	4.88435200	0.33462400	-2.72747300
C	3.65037800	-0.09646900	0.20257400
H	4.31480800	0.76327600	0.13644600
H	3.83369600	-0.56604300	1.16811800
C	5.53790700	-1.51399300	-0.61429300
O	6.34030100	-0.83333500	-0.01383100
O	5.85156400	-2.70092200	-1.17661300
C	7.22967700	-3.08506000	-1.05926400
H	7.32183300	-4.02264900	-1.60647800
H	7.87735700	-2.31841300	-1.48996700
H	7.50087800	-3.22568000	-0.00967400
C	2.20846900	0.33409100	0.10929800
C	1.23880100	-0.21040400	0.95829100
C	1.80714800	1.27791200	-0.84191200
C	-0.10110800	0.16767900	0.86015100
H	1.53400900	-0.95088400	1.69076400
C	0.47460800	1.66842700	-0.93761400
H	2.54000400	1.70491400	-1.51729400
C	-0.48725600	1.11358300	-0.08999200
H	-0.83773000	-0.26536500	1.52746700
H	0.19190400	2.41071400	-1.67079000
H	-1.52442100	1.42105700	-0.16374500

(TfO•2a)⊂AuCav-1

C	4.08229100	4.19022300	1.05704400
C	2.65045700	4.45371500	0.60304200
C	4.45307100	2.70970900	1.07138600
H	4.12693600	4.51604000	2.09690100
C	2.32743300	4.59581000	-0.75035100
C	1.60928100	4.61432400	1.53020800
C	4.94727500	2.02651000	-0.04471900
C	4.34765800	1.98558000	2.26500200
C	1.04732000	4.93096500	-1.19508200
H	3.10911000	4.44197600	-1.48538000
C	0.32401000	4.97782500	1.13679000
O	1.87762000	4.47630800	2.89333900
C	5.36148600	0.68908500	0.00870100
H	5.00994700	2.55283100	-0.99096400
C	4.78606100	0.67557500	2.38134900
O	3.80164200	2.62625100	3.37656300
C	0.67351800	4.98222200	-2.67397600
C	0.06853900	5.14578500	-0.21872500
H	-0.45289800	5.13195600	1.87473200
C	1.49590700	3.28346900	3.46316300
C	5.79704800	-0.09079300	-1.22991800
C	5.29964800	0.04885100	1.25238800
H	4.70340400	0.14370300	3.31876600
C	2.50652900	2.28699600	3.67054600
H	-0.21450300	5.60885200	-2.75811000
C	0.26417700	3.57107000	-3.08102100
O	-1.16731100	5.62002200	-0.65184800
C	4.55763300	-0.65570700	-1.92137500
H	6.37223900	-0.94572400	-0.87392800

O	5.76435900	-1.26401000	1.35346100
C	1.19617200	2.64333700	-3.54533900
C	-1.06006600	3.13034600	-2.94997600
C	-2.31071700	4.90414000	-0.46920300
C	-0.06392200	1.86571300	4.32628500
C	3.83609000	0.08802500	-2.85912600
C	4.06359400	-1.92898500	-1.59551000
C	4.79420400	-2.19289800	1.63280200
C	0.92732400	0.84332700	4.44469500
C	0.87922500	1.31800100	-3.85191600
H	2.21510000	2.97397900	-3.70208600
C	-1.40907200	1.79977400	-3.17026800
O	-2.05156400	4.08126300	-2.72325700
C	-2.79499200	4.11930200	-1.57681100
C	2.67388500	-0.39030300	-3.46569300
H	4.19643400	1.07374700	-3.12872900
C	2.89429000	-2.43697300	-2.15941600
O	4.79237100	-2.71226300	-0.70738200
C	4.24894900	-2.93549200	0.53481800
C	1.90009300	0.39886100	-4.50712800
C	-0.42805200	0.89566700	-3.57623600
H	-2.44044900	1.48390900	-3.08291200
C	-4.23735500	4.48304700	0.67172000
C	2.21466400	-1.65270800	-3.08284700
H	2.53271500	-3.41840700	-1.87845500
C	3.37906500	-3.26025600	3.05799500
H	1.33014600	-0.33025900	-5.08801300
O	-0.85074600	-0.40079200	-3.86846700
C	-4.74440100	3.76023100	-0.45093800
O	1.07408500	-2.16350400	-3.70544100
C	2.82232100	-3.99082300	1.96087000
C	0.55543000	-0.44567800	4.88964100
C	-1.39259200	1.59012100	4.72380600
C	-6.07454600	3.28238400	-0.42734300
C	-5.05760400	4.68006200	1.80491000
C	1.76133600	-4.89174500	2.19018600
C	2.86448100	-3.45670400	4.35930400
C	2.81637300	1.13187500	-5.49848200
H	3.45883300	0.40915000	-6.00824100
H	3.46864300	1.86563800	-5.01838900
C	1.76374400	5.59285500	-3.56226300
H	1.43412300	5.61356800	-4.60471500
H	2.70826200	5.04292500	-3.52571700
C	5.10188000	5.02847900	0.26834700
H	4.84704800	6.08998600	0.33135900
H	5.14491000	4.76138700	-0.79143900
C	6.69656200	0.72210500	-2.17046300
H	7.59415200	1.04737200	-1.63771100
H	6.20783300	1.61828200	-2.56351300
H	7.00131500	0.11104700	-3.02464400
H	6.10314600	4.88418000	0.68315500
H	1.97152400	6.61760300	-3.24327500
H	2.21952300	1.65938000	-6.24740300
C	1.81884000	-4.33615500	4.55273100
C	1.26236800	-5.05079000	3.46606700
H	0.41756400	-5.71051200	3.61945200
H	1.41283000	-4.48112500	5.54926100
H	1.34722000	-5.42749000	1.34516900
H	3.31017600	-2.89615400	5.17425800
C	-1.72942500	0.32679400	5.16424900
H	-2.13082400	2.37967600	4.63435300
C	-0.75583200	-0.69857000	5.23181700
H	1.31732100	-1.21587900	4.92709000
C	-6.35563600	4.21831700	1.79679200
H	-4.64210500	5.21754200	2.64967600
C	-6.86914700	3.53200200	0.67178900
H	-6.44736900	2.75386900	-1.29792900
H	-7.90180500	3.19842800	0.67543600
H	-6.99287600	4.38169700	2.65946500
H	-1.05008700	-1.69374400	5.54858800
H	-2.75711700	0.10591500	5.43252000
P	-0.45308800	-1.82907300	-3.16626200
N	-1.39620500	-2.82380800	-4.09594100
C	-1.58925300	-2.57261200	-5.52670200
C	-1.41170300	-4.25150600	-3.74392700
H	-1.66335400	-1.50335500	-5.71661500
H	-2.52538900	-3.04817000	-5.83400400
H	-0.77002000	-2.99331100	-6.12529200
H	-1.22103500	-4.40097700	-2.68061200
H	-0.65131000	-4.80241800	-4.31219500
H	-2.39884800	-4.66135400	-3.97544100
Au	-0.65135700	-2.26330300	-0.97571300
N	3.29441100	-3.80259800	0.68809600
N	4.38166600	-2.35365300	2.85443000
N	2.22980600	1.10025500	4.12123700
N	0.25455100	3.08883300	3.79775000
N	-2.99155500	5.04785200	0.62943900
N	-3.97182000	3.56546100	-1.56565300
C	-3.81810700	-1.00417200	-1.02714700
C	-4.10966800	-1.49670800	-2.22533200
H	-4.21544500	-0.82680500	-3.06965300
C	-3.56554100	0.41827200	-0.60415900
C	-3.51225700	0.36479000	0.93043200
H	-4.31912700	1.09682900	-0.99818600
H	-2.59093200	0.74470900	-0.97608900
C	-3.45317700	-1.15092800	1.22136500
O	-3.66473000	-1.84745200	0.06741400
O	-3.30191200	-1.67730100	2.28890700
H	-4.23026400	-2.55942100	-2.38124100
C	-2.33656700	1.12057400	1.57358700
H	-2.40593300	0.95325600	2.64564300
H	-2.49622700	2.19069000	1.41410000
C	-4.81694200	0.78402200	1.61650700
O	-4.89740100	1.48903600	2.59504800
O	-5.86682500	0.16703700	1.03500500
C	-7.11015500	0.24198200	1.75213800
H	-7.84888200	-0.23481100	1.10918800
H	-7.02292400	-0.29643300	2.69921400
H	-7.37668500	1.27955700	1.95130400
C	-0.96106100	0.72721000	1.08757000
C	-0.35302500	-0.46056900	1.52676000
C	-0.23751300	1.56863800	0.23408600
C	0.94593700	-0.78652600	1.12192900
C	1.06175500	1.25055700	-0.16607500
H	-0.68416400	2.49756500	-0.09946000
C	1.65881200	0.07330100	0.28011500
H	1.60576400	1.92623000	-0.81374300
H	2.67023400	-0.16650800	-0.01980800
S	-1.32221000	-4.89068000	0.61716300
O	-1.30604800	-5.60755400	1.89182900
O	-1.11342000	-3.38187200	0.80817700
O	-0.53489400	-5.42234100	-0.51023600
C	-3.08844500	-5.03843200	0.04535400
F	-3.37377100	-6.33393200	-0.13127800
F	-3.25830100	-4.41300600	-1.13932400
F	-3.93292600	-4.52460300	0.93362700
H	-0.87938400	-1.12269400	2.20292400
H	1.39983300	-1.69989800	1.48943200

(TfO•2a)⊂AuCav-2

C	4.84946000	3.38053900	-0.96827500
C	3.35955200	3.72238100	-0.95652200
C	5.08560400	1.89520200	-0.69758600
H	5.28882900	3.91517300	-0.12557900
C	2.57530200	3.70200900	-2.11382600
C	2.72482600	4.08481800	0.24120000
C	4.97255300	0.92795000	-1.70181700
C	5.39582500	1.44213900	0.59151600
C	1.22071100	4.04442500	-2.11411300
H	3.04426400	3.43158200	-3.05266100
C	1.37115400	4.41024100	0.29793200
O	3.51629400	4.19940300	1.38021200
C	5.15497800	-0.43993500	-1.47089300
H	4.71893400	1.25430700	-2.70474900
C	5.58151100	0.09270000	0.87251300
O	5.50686900	2.37402400	1.62724300
C	0.36099800	4.04343300	-3.37672000
C	0.64069500	4.38888900	-0.88570200
H	0.90970100	4.69177200	1.23532900
C	3.39966400	3.27077100	2.38220600

C	5.03447700	-1.47858800	-2.58545100
C	5.45446400	-0.83030300	-0.15871100
H	5.78714400	-0.23463100	1.88312000
C	4.48056200	2.34074900	2.54429900
H	-0.42255300	4.78599900	-3.22388900
C	-0.33380400	2.69236500	-3.49076000
O	-0.67045800	4.85429800	-0.93352600
C	3.58312300	-1.89585400	-2.81111500
H	5.55230000	-2.36862100	-2.22620800
O	5.65534000	-2.18916400	0.11486500
C	0.32108700	1.58736700	-4.03891300
C	-1.63454700	2.49179600	-3.00856200
C	-1.73463500	4.22771200	-0.35961200
C	2.33096900	2.33377200	4.15781500
C	2.73169800	-1.21539800	-3.68532300
C	3.04656200	-2.99291500	-2.12130900
C	4.65251800	-2.80351900	0.82292100
C	3.42522900	1.43575500	4.34422100
C	-0.23779700	0.30870400	-4.09532600
H	1.31355600	1.73115800	-4.44810800
C	-2.20721400	1.21992500	-2.98078900
O	-2.39006300	3.58421900	-2.59541000
C	-2.70305200	3.65437200	-1.25571000
C	1.39858700	-1.58735400	-3.88630600
H	3.11964700	-0.36116200	-4.22741300
C	1.72762700	-3.40338000	-2.29290300
O	3.87586700	-3.69890600	-1.26197300
C	3.67285500	-3.55070200	0.08957200
C	0.46244800	-0.83607100	-4.81979500
C	-1.48739500	0.14322200	-3.48596700
H	-3.20566300	1.08486800	-2.58270800
C	-3.11763500	3.78309200	1.39234000
C	0.92417700	-2.68971500	-3.17215300
H	1.34533500	-4.24813700	-1.73542900
C	3.56458000	-3.29943100	2.75796700
H	-0.31940300	-1.54153300	-5.11178600
O	-2.10654000	-1.11955900	-3.44047100
C	-4.11952700	3.30784900	0.48870700
O	-0.41276300	-3.09689300	-3.34280100
C	2.55327600	-3.98550100	2.01949700
C	3.37531200	0.48099700	5.38413500
C	1.20491600	2.24309800	5.00592000
C	-5.36061900	2.85949900	0.99421200
C	-3.36873100	3.76032300	2.78223100
C	1.44532700	-4.53552200	2.70198700
C	3.45063400	-3.19467600	4.16242400
C	1.15066900	-0.37849400	-6.11478600
H	1.56851200	-1.24296200	-6.63755100
H	1.96803400	0.32474700	-5.93800500
C	1.13123300	4.43379400	-4.64483600
H	0.45172900	4.46879200	-5.50099200
H	1.93613000	3.73608300	-4.89272100
C	5.56179800	3.85742600	-2.24277300
H	5.40455600	4.93018000	-2.38650600
H	5.21092000	3.34767200	-3.14494600
C	5.72671900	-1.02928100	-3.88108000
H	6.78092500	-0.81718200	-3.68448400
H	5.28678100	-0.12374200	-4.30867400
H	5.66469300	-1.81689900	-4.63759700
H	6.63593900	3.67032400	-2.16069700
H	1.58219400	5.42168300	-4.51849400
H	0.42867100	0.11348700	-6.77189600
C	2.35852900	-3.73434200	4.80386400
C	1.35029900	-4.40576300	4.07070600
H	0.49684500	-4.82263300	4.59682300
H	2.26012600	-3.63835900	5.88050300
H	0.69061500	-5.05171200	2.11838200
H	4.22560600	-2.65660600	4.69574500
C	1.17646900	1.29693200	6.00665500
H	0.37551400	2.91728300	4.82588600
C	2.26687300	0.41480300	6.19873900
H	4.21751200	-0.19231200	5.49662500
C	-4.58042400	3.29977800	3.25141300
H	-2.58535900	4.10596700	3.44766900
C	-5.58137800	2.85863200	2.35545000
H	-6.11230400	2.50878100	0.29558200
H	-6.52498900	2.49339500	2.74328500
H	-4.77026800	3.26759900	4.31927500
H	2.21975700	-0.32685000	6.99028200
H	0.30596300	1.21744900	6.65020100
P	-1.58859100	-2.33335100	-2.47695600
N	-2.90801500	-3.32861800	-2.55144800
C	-3.76971400	-3.40290100	-3.73598100
C	-2.80866700	-4.58825600	-1.80251100
H	-3.86293000	-2.41954700	-4.19335200
H	-4.75947200	-3.74039600	-3.41533700
H	-3.37791000	-4.11222000	-4.47652100
H	-2.12389200	-4.46647000	-0.96087800
H	-2.43567800	-5.39886600	-2.44113900
H	-3.78824400	-4.84460800	-1.39598300
Au	-0.81308900	-1.81259200	-0.43939600
N	2.64955300	-4.11296800	0.66112200
N	4.61432700	-2.70455600	2.11820100
N	4.50264500	1.46524800	3.50298500
N	2.35794200	3.26770900	3.15790700
N	-1.92610500	4.26945300	0.92553800
N	-3.86469700	3.23457100	-0.85525600
C	-3.27360400	0.18118100	0.83018700
C	-2.15771700	0.80258500	0.47495100
H	-1.86641300	0.82418000	-0.56392100
C	-4.26557000	-0.59285400	0.00764200
C	-4.96395400	-1.48313500	1.06048900
H	-3.80437400	-1.15584000	-0.80446800
H	-4.99965300	0.09726100	-0.42128000
C	-4.74412200	-0.65880400	2.34333800
O	-3.71621300	0.21275200	2.14932300
O	-5.35028100	-0.73658900	3.37871000
H	-1.54090600	1.30641400	1.20360900
C	-6.45222200	-1.79551900	0.80799500
H	-6.82623200	-2.32482000	1.68872900
H	-6.50166700	-2.49583700	-0.02949800
C	-4.18910500	-2.80183700	1.21360900
O	-4.54445600	-3.87024300	0.76464400
O	-3.04998400	-2.60402300	1.89300800
C	-2.22040700	-3.74956600	2.16599900
H	-1.29676400	-3.34258900	2.57102500
H	-2.72294300	-4.40407900	2.88205400
H	-2.02323900	-4.30413900	1.24705700
C	-7.30203300	-0.57805400	0.53440800
C	-7.88857900	0.13880200	1.58577300
C	-7.50782700	-0.13112600	-0.77778000
C	-8.65612400	1.27507800	1.32943500
C	-8.26475000	1.01223500	-1.03681800
H	-7.07480100	-0.68926000	-1.60432600
C	-8.84068200	1.72042400	0.01898300
H	-8.40814000	1.34563200	-2.06008000
H	-9.43321900	2.60838700	-0.17814000
S	0.94375600	-0.40729600	1.93109900
O	2.12536500	-0.84127600	2.67007800
O	0.12943300	-1.61515700	1.44027100
O	0.12434700	0.67041600	2.48277000
C	1.64509300	0.28045700	0.34524900
F	2.53256400	1.22340700	0.65367100
F	0.70712900	0.83268000	-0.44770100
F	2.26067900	-0.68547800	-0.35399400
H	-7.72958300	-0.19543500	2.60558300
H	-9.11088300	1.81472200	2.15498100

(TfO•2a)⊂AuCav-3

C	-3.58828500	-1.55665800	4.21639600
C	-2.80532900	-2.57693000	3.39471300
C	-3.45993900	-0.14333000	3.65251600
H	-3.11344900	-1.53195600	5.19767000
C	-3.31185900	-3.16807100	2.23260100
C	-1.51090500	-2.94177400	3.78459700
C	-4.32073900	0.33383000	2.66082700
C	-2.47610000	0.73953800	4.12621500
C	-2.58595800	-4.09566200	1.47649500
H	-4.30453500	-2.88667700	1.89811900
C	-0.75665100	-3.86992000	3.07722500

O	-0.96179000	-2.33072500	4.91131600
C	-4.27378800	1.64197000	2.16862200
H	-5.07045900	-0.34055600	2.26325100
C	-2.39361100	2.05159100	3.67251600
O	-1.60302000	0.30602300	5.12851100
C	-3.12493100	-4.71819100	0.19081200
C	-1.30555400	-4.43013000	1.93196800
H	0.24527900	-4.12416500	3.39437600
C	0.01372100	-1.40309300	4.62665000
C	-5.23011600	2.13042900	1.08092900
C	-3.30449600	2.49220000	2.71634800
H	-1.63422500	2.71869700	4.05851700
C	-0.31976800	-0.01446500	4.74750300
H	-2.54455000	-5.62563200	0.02068700
C	-2.84048500	-3.79909700	-0.99259400
O	-0.58090900	-5.39239700	1.22323600
C	-4.66991100	1.83280300	-0.30970900
H	-5.26238800	3.21697200	1.16954400
O	-3.31550600	3.84478300	2.35887300
C	-3.75271400	-2.82684400	-1.40542800
C	-1.64179300	-3.90037900	-1.71534900
C	0.54048000	-4.97764500	0.56229800
C	2.10299400	-0.84674400	3.91866500
C	-4.91183900	0.63591000	-0.98758800
C	-3.85033800	2.77302300	-0.95063700
C	-2.27689300	4.32865700	1.61215800
C	1.78906800	0.53769100	4.08086200
C	-3.52958300	-1.97516500	-2.49080500
H	-4.69300000	-2.74605800	-0.87490200
C	-1.34934500	-3.03886900	-2.77025000
O	-0.78800700	-4.95939700	-1.43530100
C	0.43410500	-4.74560400	-0.85222200
C	-4.37366600	0.35631700	-2.24899100
H	-5.52883000	-0.11591800	-0.50976700
C	-3.30089700	2.54564200	-2.20909900
O	-3.64680100	3.98594100	-0.31756800
C	-2.41304000	4.31622200	0.18163600
C	-4.58465600	-0.97651700	-2.95248700
C	-2.29244500	-2.08382700	-3.13519600
H	-0.41788500	-3.14073300	-3.31321500
C	2.76893200	-4.52783400	0.45931400
C	-3.56735500	1.33394600	-2.83631300
H	-2.67783700	3.29927300	-2.67327300
C	-0.25657700	5.34500700	1.39086800
H	-4.40852700	-0.80317300	-4.01687500
O	-2.00161700	-1.29252800	-4.26295300
C	2.67013400	-4.33336200	-0.95179900
O	-2.99276600	1.09460200	-4.10063600
C	-0.34447400	5.22921000	-0.03048800
C	2.75251900	1.50853700	3.72517100
C	3.35781600	-1.22764400	3.39242200
C	3.83519600	-4.03629300	-1.69595100
C	4.02768800	-4.40692000	1.09412000
C	0.70056100	5.73479800	-0.83672600
C	0.85123200	6.01002700	1.96561500
C	-6.01934100	-1.50600200	-2.81849500
H	-6.72210100	-0.78055200	-3.23676900
H	-6.31393200	-1.68568100	-1.78169400
C	-4.60095000	-5.12483000	0.30141200
H	-4.93897900	-5.58865700	-0.62969400
H	-5.26425400	-4.28116300	0.51205100
C	-5.05197100	-1.97804200	4.42002200
H	-5.09489400	-2.95688500	4.90577000
H	-5.60884100	-2.05388800	3.48150600
C	-6.66165400	1.60704500	1.26495300
H	-7.04001400	1.89560800	2.24908300
H	-6.73260000	0.51784900	1.19463400
H	-7.31993800	2.03005900	0.50071400
H	-5.56943300	-1.25130400	5.05233800
H	-4.72518900	-5.84527900	1.11407300
H	-6.12599000	-2.44949000	-3.36046500
C	1.83506400	6.53303900	1.15382700
C	1.76816800	6.38042100	-0.25094200
H	2.56830300	6.77501300	-0.86841700
H	2.68240200	7.04969300	1.59217500

H	0.61762100	5.62305600	-1.91250100
H	0.88763100	6.09589700	3.04613700
C	4.27158400	-0.25883500	3.04122000
H	3.55237700	-2.28511400	3.25172200
C	3.96924200	1.11253600	3.21226500
H	2.48566700	2.55431000	3.82209000
C	5.14611500	-4.11174800	0.34513300
H	4.07495400	-4.56840300	2.16560600
C	5.05062700	-3.92558600	-1.05540000
H	3.74008400	-3.88051500	-2.76375500
H	5.94084300	-3.68016200	-1.62533600
H	6.11283100	-4.02137700	0.82771700
H	4.69128900	1.85908400	2.90338800
H	5.22170900	-0.54521100	2.60148900
P	-1.56275900	0.27408500	-4.17095300
N	-0.98914100	0.51520800	-5.69859600
C	-1.74164000	0.03496300	-6.86336700
C	-0.15067700	1.69071000	-5.94840700
H	-2.27296700	-0.88175900	-6.60960000
H	-1.03472100	-0.18552000	-7.66836800
H	-2.45915100	0.78719800	-7.21447500
H	0.41338400	1.94754200	-5.04859300
H	-0.74771600	2.56013900	-6.25295300
H	0.56187200	1.45286300	-6.74340400
Au	-0.31716200	0.88844600	-2.42244800
N	-1.46911400	4.71259400	-0.61721300
N	-1.24209600	4.84903900	2.19873800
N	0.54861300	0.92412300	4.51262200
N	1.18340800	-1.80661700	4.23028400
N	1.66724300	-4.85763500	1.19966300
N	1.46048300	-4.43488000	-1.58714100
C	3.44275300	-0.92481600	-1.85327300
C	2.15425700	-1.18770200	-2.04931500
H	1.47194500	-1.10415900	-1.21748800
C	4.18167100	-0.48875000	-0.61801300
C	5.48855800	0.07747000	-1.19345300
H	4.38724700	-1.36584600	-0.00006700
H	3.61424200	0.21228100	-0.01247300
C	5.62081800	-0.72876100	-2.49657800
O	4.36747000	-1.13921700	-2.87771900
O	6.60021800	-0.96045100	-3.15087100
H	1.79973300	-1.58502100	-2.99192800
C	5.36982700	1.57560100	-1.62355900
H	4.61596000	1.63074400	-2.41575700
H	6.33004700	1.86627300	-2.05343700
C	6.74284700	-0.11818000	-0.35244300
O	7.71203800	0.60598700	-0.38539700
O	6.67742300	-1.22344400	0.41987600
C	7.85845200	-1.48809900	1.19704400
H	7.62655400	-2.37119800	1.79136200
H	8.71081300	-1.67436700	0.53997600
H	8.08659400	-0.63814400	1.84343000
C	4.97366800	2.49765400	-0.49615900
C	3.61821300	2.74263800	-0.24293400
C	5.93552800	3.10481400	0.32216300
C	3.22372700	3.55549600	0.81683200
C	5.54295800	3.94123200	1.36901500
H	6.98688300	2.91701300	0.13380700
C	4.18777300	4.16395000	1.62123600
H	6.29805300	4.41563500	1.98960200
H	3.88142500	4.80397400	2.44267300
S	0.49443000	1.07304900	0.72433500
O	0.12601000	2.09109700	1.70418500
O	0.52800800	1.68667600	-0.68446000
O	1.63292600	0.18834500	0.97771300
C	-0.93516800	-0.12768000	0.68462200
F	-1.05035500	-0.68230300	1.88970400
F	-0.71562200	-1.11702300	-0.20712000
F	-2.07992000	0.47917700	0.35793400
H	2.85984000	2.28897700	-0.87185700
H	2.16990000	3.70575500	1.01322700

(TfO•2a)⊂AuCav-4

C	4.44760800	1.25813700	3.26742400
C	3.29640500	2.20225900	2.94314600

C	4.42362200	-0.00721700	2.41855400
H	4.28795900	0.93380800	4.29587600
C	3.40865400	3.19532400	1.96739900
C	2.09724000	2.14540000	3.66873900
C	4.96389900	-0.05786200	1.13095600
C	3.88240600	-1.19158300	2.93320700
C	2.41506500	4.14791600	1.73527100
H	4.32128800	3.24548000	1.38578800
C	1.07910200	3.08088800	3.48200200
O	1.99964100	1.20497700	4.69719100
C	5.01737200	-1.23381800	0.37742900
H	5.35912700	0.85487200	0.69889100
C	3.93522300	-2.39295400	2.23380500
O	3.32596600	-1.15690700	4.21678700
C	2.54217200	5.20726700	0.64399500
C	1.25939300	4.07955600	2.52516800
H	0.18293400	3.05271800	4.08853700
C	1.26154200	0.06439500	4.52251100
C	5.54851300	-1.25721300	-1.05316300
C	4.50935300	-2.39864600	0.96514400
H	3.53300000	-3.30116400	2.66243000
C	1.95678800	-1.17427100	4.28293200
H	1.90829000	6.04360500	0.93820900
C	1.94876200	4.63038700	-0.63579100
O	0.34566500	5.12634300	2.40043100
C	4.42033300	-0.83089200	-1.98543300
H	5.77609400	-2.29708000	-1.28701900
O	4.64339900	-3.60001400	0.26797900
C	2.68973500	3.80517400	-1.48485000
C	0.60736100	4.85843400	-0.97119400
C	-0.91254900	4.93085000	1.90597500
C	-0.72350700	-1.06340900	4.50727000
C	4.21186100	0.50413900	-2.33042200
C	3.51404000	-1.77733800	-2.48660400
C	3.50075600	-4.21574400	-0.17365300
C	-0.03516100	-2.28253900	4.23123400
C	2.14010200	3.16728200	-2.60007700
H	3.73931700	3.64723400	-1.26699500
C	0.01128900	4.22041400	-2.05848900
O	-0.12050800	5.77815200	-0.21839200
C	-1.16714500	5.31238300	0.54179700
C	3.14838500	0.92015900	-3.13326200
H	4.91605500	1.24724100	-1.97708600
C	2.40358100	-1.40266400	-3.24328700
O	3.82099300	-3.11669500	-2.28752800
C	3.04637400	-3.93822000	-1.50961900
C	2.98872800	2.36470100	-3.57599900
C	0.76816700	3.32991800	-2.81644400
H	-1.02565600	4.41699700	-2.30196400
C	-3.11170500	4.39772500	2.15772600
C	2.23287000	-0.05356200	-3.54625000
H	1.70140200	-2.15090300	-3.59018300
C	1.77052100	-5.66628800	0.10499600
H	2.43298000	2.33912300	-4.51654500
O	0.13005500	2.65129700	-3.86227300
C	-3.37055900	4.80936800	0.81491800
O	1.16466400	0.31858700	-4.37941200
C	1.32178900	-5.39311500	-1.22217300
C	-0.77187500	-3.46898800	4.01760900
C	-2.13271500	-1.07024300	4.61555200
C	-4.68062500	4.70794000	0.29429800
C	-4.16794700	3.88237000	2.94252300
C	0.15271600	-6.02325100	-1.70422600
C	1.03543700	-6.55560500	0.92213600
C	4.33052700	3.05128500	-3.87590000
H	4.88499900	2.47721200	-4.62301300
H	4.96807600	3.14693000	-2.99356300
C	3.96906400	5.74145900	0.46621800
H	3.98153800	6.52930000	-0.29197100
H	4.68148100	4.97335200	0.15255000
C	5.81564800	1.95758100	3.21492000
H	5.81806000	2.83328300	3.86999300
H	6.08598400	2.29420300	2.21009200
C	6.83717700	-0.44415600	-1.23107200
H	7.61949400	-0.84157300	-0.57894600
H	6.71597000	0.61466600	-0.98489900
H	7.18385900	-0.50544300	-2.26661400
H	6.59666800	1.26935500	3.54921300
H	4.33232100	6.16048000	1.40846600
H	4.15627900	4.05794500	-4.26519200
C	-0.10806500	-7.14743500	0.43144400
C	-0.54907500	-6.88185900	-0.88742800
H	-1.45623000	-7.35424600	-1.25077400
H	-0.68084600	-7.82257400	1.05952000
H	-0.17534400	-5.78598900	-2.70933400
H	1.39643600	-6.74054900	1.92811700
C	-2.82867600	-2.24444800	4.41593400
H	-2.63300700	-0.13701700	4.85084500
C	-2.14707300	-3.44364800	4.10267700
H	-0.22755100	-4.37065500	3.76170100
C	-5.43476000	3.78430700	2.40827200
H	-3.94272800	3.56611300	3.95494800
C	-5.69288100	4.20010700	1.08011800
H	-4.85023100	5.02380900	-0.72923100
H	-6.69731800	4.11112500	0.67871800
H	-6.24569200	3.38294000	3.00790600
H	-2.71781200	-4.34835300	3.92241000
H	-3.91165800	-2.25380400	4.49143100
P	-0.21025900	1.03713900	-3.82058800
N	-1.25132200	0.95385400	-5.09869600
C	-0.83243300	1.44665100	-6.41414800
C	-2.29399000	-0.07785000	-5.12142500
H	-0.12488900	2.26777300	-6.29782000
H	-1.71151800	1.82311300	-6.94556200
H	-0.36748300	0.65199300	-7.01114600
H	-2.67301900	-0.26841600	-4.11548800
H	-1.93129400	-1.01724800	-5.55983700
H	-3.12715100	0.29252200	-5.72584200
Au	-0.95688300	0.10811000	-1.93015800
N	2.00512200	-4.51912300	-2.02313300
N	2.89312900	-5.06250200	0.60106100
N	1.32860600	-2.30294200	4.14566400
N	-0.03298000	0.11065000	4.64601100
N	-1.85476100	4.49165000	2.68678000
N	-2.35685000	5.27064300	0.02048600
C	-5.44788200	-3.21732200	1.06870700
C	-5.21344600	-4.45906400	1.47056300
H	-4.24544600	-4.70129600	1.89077200
C	-4.57887400	-1.99605400	1.14138800
C	-5.15281000	-1.07642000	0.04688900
H	-3.52412800	-2.22915500	1.03533900
H	-4.72345000	-1.51283600	2.11355000
C	-6.58951800	-1.63049300	-0.09858300
O	-6.66824600	-2.86393100	0.48463100
O	-7.52781700	-1.10085800	-0.62961300
H	-5.95598500	-5.24012000	1.36307000
C	-5.20006000	0.42329000	0.41097700
H	-5.79854800	0.52021500	1.32328000
H	-5.73348000	0.94946700	-0.38273500
C	-4.47911100	-1.30086900	-1.31609600
O	-4.27307900	-0.43916800	-2.14677300
O	-4.20772100	-2.59925200	-1.50050300
C	-3.60435500	-2.96084300	-2.75575800
H	-3.60052300	-4.04947300	-2.76753000
H	-4.19194800	-2.56136300	-3.58529100
H	-2.57959300	-2.58933600	-2.79566100
C	-3.82783100	1.01535400	0.61709000
C	-3.11365200	0.81667800	1.80573200
C	-3.21668300	1.73868200	-0.40738400
C	-1.79731300	1.25177500	1.94284300
C	-1.89989000	2.18193900	-0.27227900
H	-3.76230900	1.93180500	-1.32309500
C	-1.17343200	1.91439800	0.88857600
H	-1.45723200	2.77223500	-1.05765300
H	-0.14365500	2.24382700	0.97762100
S	-0.95414600	-2.47216800	-0.15577800
O	-0.54087400	-3.06666900	-1.43232900
O	-1.66797500	-1.13128900	-0.34348500
O	-1.63333900	-3.30923500	0.83456500
C	0.62191700	-1.93012800	0.68343900

F	1.14672900	-2.97385300	1.32537600
F	0.38430700	-0.94734300	1.56217700
F	1.51343800	-1.47457600	-0.21262400
H	-3.58564900	0.29932000	2.63339400
H	-1.26309100	1.08580900	2.86927300

(TfO•2a)⊂AuCav-5

C	6.08111500	-0.68916800	1.10383700
C	5.30751100	0.60411800	1.34431200
C	5.23580100	-1.78834800	0.46468900
H	6.35336400	-1.06024300	2.09292600
C	5.24054400	1.63854300	0.40377700
C	4.66229800	0.81638700	2.57022200
C	4.99501600	-1.84025600	-0.91163700
C	4.68769000	-2.81993600	1.23988500
C	4.58751700	2.85159600	0.65271700
H	5.72428300	1.49729200	-0.55577100
C	4.02280800	2.00945800	2.87605500
O	4.69814400	-0.19937400	3.52783800
C	4.27059500	-2.86960500	-1.52494300
H	5.36584800	-1.03109200	-1.53184900
C	4.01049200	-3.88904300	0.67203100
O	4.77766000	-2.75183500	2.63819200
C	4.48188300	3.96554100	-0.38717000
C	4.00151000	3.01149700	1.91435500
H	3.54190600	2.15119800	3.83348400
C	3.55248700	-0.93983100	3.64329400
C	3.93301900	-2.83337000	-3.01049700
C	3.81304600	-3.90665300	-0.70472300
H	3.59864900	-4.67129600	1.29512100
C	3.60785600	-2.30541400	3.20554000
H	4.32889100	4.89171700	0.16814300
C	3.23380700	3.76665000	-1.24501700
O	3.40166300	4.23614900	2.21671500
C	2.76619900	-1.87743500	-3.24312500
H	3.57366100	-3.82962200	-3.27182600
O	3.09555600	-4.97309100	-1.26009900
C	3.23134300	2.92861800	-2.36629100
C	2.02384200	4.39500300	-0.91731100
C	2.04210800	4.22596800	2.34846300
C	1.35149400	-1.19801300	4.17519700
C	2.94673500	-0.55486200	-3.64971300
C	1.44637200	-2.32034800	-3.05376000
C	1.78456900	-4.97770800	-0.84107100
C	1.41038700	-2.56211700	3.75442800
C	2.09219100	2.69212300	-3.14264600
H	4.15256500	2.42508900	-2.63588200
C	0.86612500	4.18833300	-1.65789900
O	1.96880000	5.25230200	0.18323600
C	1.27575200	4.77657100	1.26777900
C	1.87825700	0.31289400	-3.89865700
H	3.95531200	-0.19203000	-3.80336700
C	0.34659800	-1.50379600	-3.29673600
O	1.28183600	-3.66304500	-2.74669100
C	0.84833900	-4.13963800	-1.54492600
C	2.09545800	1.75435600	-4.34641200
C	0.91072600	3.33146800	-2.75056400
H	-0.05851600	4.66981000	-1.36766600
C	0.12099300	3.76923800	3.48070700
C	0.58667500	-0.19737300	-3.71688400
H	-0.66337800	-1.87967400	-3.19052000
C	0.16277400	-5.54000800	0.64741200
H	1.22746200	2.02638600	-4.95202600
O	-0.28580700	3.08446200	-3.42342100
C	-0.64274100	4.32930900	2.41078000
O	-0.52489700	0.60035400	-4.04484000
C	-0.73655900	-4.62595200	0.01684100
C	0.24398700	-3.35895700	3.79855700
C	0.12403700	-0.66565900	4.63483700
C	-2.05174400	4.35869800	2.50803900
C	-0.54359200	3.24890800	4.61508500
C	-2.04221100	-4.46759100	0.53650900
C	-0.26692000	-6.28784100	1.76700100
C	3.33709800	1.93966100	-5.23005500
H	3.28771900	1.27264000	-6.09484600
H	4.27147500	1.72976900	-4.70293100
C	5.75779500	4.13010800	-1.22493100
H	5.64999700	4.97419800	-1.91157900
H	5.99305000	3.24675600	-1.82514200
C	7.38517300	-0.45570000	0.32348400
H	7.99949600	0.29494400	0.82857800
H	7.21122900	-0.10988300	-0.69938100
C	5.15763000	-2.53286500	-3.88534200
H	5.91600300	-3.30606300	-3.73673800
H	5.62420700	-1.57134500	-3.65187700
H	4.87755600	-2.51966700	-4.94251100
H	7.95332200	-1.38759900	0.26066300
H	6.61325200	4.32027000	-0.57114200
H	3.38844400	2.97167000	-5.58710800
C	-1.54277800	-6.10856500	2.26193000
C	-2.42611000	-5.19048400	1.64873500
H	-3.42049300	-5.05047700	2.05899700
H	-1.87234200	-6.67455400	3.12789300
H	-2.72901600	-3.80421900	0.02140000
H	0.43619500	-6.98041700	2.21726700
C	-0.99930900	-1.46639700	4.67183400
H	0.09283300	0.37786900	4.92715000
C	-0.94089400	-2.81703500	4.24948300
H	0.30734500	-4.38262600	3.45133100
C	-1.91982400	3.27979000	4.67694900
H	0.06219900	2.83649400	5.41496500
C	-2.67717100	3.83746500	3.61993600
H	-2.60868900	4.79873700	1.68859700
H	-3.75955900	3.83130400	3.67718300
H	-2.43602700	2.86170500	5.53396700
H	-1.84705200	-3.41028900	4.25103600
H	-1.94666100	-1.05832200	5.00816000
P	-1.10758100	1.69928800	-2.99344500
N	-2.63122200	1.94613300	-3.53731600
C	-2.93953300	2.01880400	-4.96678200
C	-3.63321200	2.50852500	-2.63513600
H	-2.20649400	1.44539600	-5.53345900
H	-2.94263600	3.05827700	-5.31830000
H	-3.92228600	1.57331400	-5.13243800
H	-3.31472600	2.39018300	-1.59913300
H	-4.57297200	1.96704200	-2.75774600
H	-3.79301100	3.57682800	-2.83015500
Au	-0.63832600	1.12173400	-0.80114100
N	-0.35572700	-3.93058300	-1.10535700
N	1.45251800	-5.66854300	0.20514800
N	2.57298400	-3.08990900	3.26296100
N	2.46176100	-0.40064100	4.10479800
N	1.48634400	3.73179400	3.41551500
N	-0.02386800	4.82567600	1.29390500
C	-1.20185300	0.00817300	1.57517400
C	0.07927400	0.27396700	1.12825600
H	0.61794400	-0.56424600	0.68929000
C	-2.10416900	-1.13612300	1.22135700
C	-3.49497700	-0.61176600	1.64772700
H	-1.81425300	-1.99926900	1.82975200
H	-2.05739600	-1.40198600	0.16632400
C	-3.11708700	0.37249100	2.74410300
O	-1.79249700	0.82248700	2.46580100
O	-3.71548000	0.79915300	3.67915000
C	-4.17048400	0.17494100	0.47533800
H	-3.36423500	0.55890600	-0.15152300
H	-4.68358900	-0.57070100	-0.12573500
C	-4.32952200	-1.75156100	2.23271300
O	-3.90497400	-2.50280700	3.08769200
O	-5.54152600	-1.84554600	1.68893300
C	-6.33026800	-2.97542200	2.10474300
H	-7.31019700	-2.82668700	1.65442400
H	-5.87646100	-3.89518900	1.72956100
H	-6.39750400	-3.01389600	3.19328800
H	0.66585800	1.01762900	1.66002400
C	-5.08474800	1.31198600	0.85473300
C	-6.44665200	1.11654100	1.11334800
C	-4.57109500	2.61298400	0.92205500
C	-7.26910400	2.19427100	1.43932000
H	-6.85514800	0.11459400	1.05428600

C	-5.39119400	3.69465400	1.24265400
H	-3.51479400	2.77609400	0.72736900
C	-6.74534600	3.48676700	1.50577800
H	-8.32391100	2.02614500	1.63560200
H	-4.97709400	4.69824100	1.28192700
H	-7.38889200	4.32531400	1.75381900
S	-3.33113800	-1.99101100	-2.29654700
O	-2.75818200	-2.92647300	-3.27226000
O	-3.99600300	-2.57591900	-1.10366400
O	-2.46590200	-0.82051400	-1.96211800
C	-4.75266300	-1.21176800	-3.20591300
F	-4.33068900	-0.63123900	-4.34347600
F	-5.34461900	-0.25409800	-2.45342400
F	-5.67921500	-2.12258300	-3.51552400

(TfO•2a)⊂AuCav-6

C	3.96357500	0.62974700	4.01588100
C	2.97350800	1.68770800	3.54145000
C	3.98555000	-0.58662300	3.09764200
H	3.58722400	0.27564800	4.97584900
C	3.34208500	2.72107600	2.67713100
C	1.65184600	1.68155500	4.01153100
C	4.75135700	-0.62309800	1.92944700
C	3.25033200	-1.73484900	3.41896200
C	2.47178200	3.75220500	2.31799700
H	4.35476100	2.73711600	2.29191800
C	0.74610300	2.69038000	3.68398600
O	1.30325600	0.70726300	4.94153500
C	4.82583100	-1.74878000	1.10517800
H	5.31664600	0.26041100	1.65440900
C	3.29906600	-2.88362500	2.63355000
O	2.49802800	-1.73524600	4.59546800
C	2.87017400	4.85917300	1.34642900
C	1.17644500	3.72080400	2.85105400
H	-0.25319400	2.69288300	4.10002700
C	0.51377400	-0.35327300	4.57608700
C	5.65274500	-1.75904000	-0.17717100
C	4.08179200	-2.87416700	1.48290500
H	2.73134100	-3.76251600	2.90843500
C	1.13442200	-1.64085700	4.44227300
H	2.23837500	5.71939300	1.56707800
C	2.50523800	4.38919500	-0.05599100
O	0.37583900	4.83869100	2.62106200
C	4.81818100	-1.16464200	-1.30430400
H	5.82241100	-2.80534700	-0.43106000
O	4.19863300	-4.03139000	0.70971000
C	3.36015800	3.57247700	-0.79843400
C	1.26370700	4.70806400	-0.62423000
C	-0.78052600	4.77370400	1.89996300
C	-1.52155300	-1.29727900	4.16780000
C	4.79275000	0.20593900	-1.56645100
C	4.00164700	-1.98917200	-2.09169000
C	3.11684600	-4.43838000	-0.02863000
C	-0.90382900	-2.58044400	4.04954200
C	3.02003900	3.04004200	-2.04430400
H	4.33724200	3.34356100	-0.39086800
C	0.86376700	4.16287900	-1.84387100
O	0.45759900	5.64475000	0.01427800
C	-0.74780500	5.24333400	0.54102500
C	3.98981500	0.77051500	-2.55998000
H	5.43055300	0.85772600	-0.98171300
C	3.15134900	-1.46670200	-3.06471500
O	4.12095000	-3.36156000	-1.93073800
C	3.07228100	-4.08671300	-1.41968000
C	4.01849800	2.25566800	-2.88552200
C	1.72702600	3.29854300	-2.51119800
H	-0.09414900	4.43357100	-2.26942500
C	-3.02963500	4.46921000	1.73107800
C	3.15356700	-0.09187900	-3.27455800
H	2.51790100	-2.12641900	-3.64484600
C	1.16813200	-5.58985300	-0.27249900
H	3.68883600	2.35129600	-3.92304600
O	1.29575500	2.76126200	-3.73572100
C	-3.00332100	4.98166000	0.39826000
O	2.33309200	0.43199200	-4.28919400
C	1.14207300	-5.26467500	-1.66311600
C	-1.69806100	-3.71276200	3.76136200
C	-2.92094500	-1.18447000	4.01374000
C	-4.20818400	5.07815200	-0.33448400
C	-4.25733300	4.04831600	2.29101200
C	0.08490000	-5.73997300	-2.47007200
C	0.11504000	-6.34975200	0.28459100
C	5.43381900	2.85051200	-2.81805700
H	6.09934300	2.30360000	-3.49115000
H	5.86895000	2.80457600	-1.81684500
C	4.33146800	5.30468600	1.48133600
H	4.53952200	6.12465600	0.78830000
H	5.04796900	4.50601900	1.26992400
C	5.37041700	1.20003600	4.25503300
H	5.32414600	2.03810400	4.95616000
H	5.84832700	1.56066500	3.33958200
C	7.02368100	-1.08895100	-0.01414800
H	7.59365600	-1.59516200	0.76944500
H	6.95638700	-0.03309400	0.26323200
H	7.58799600	-1.14988400	-0.94916100
H	6.01763100	0.42680900	4.67778500
H	4.52232300	5.65378100	2.49963700
H	5.41422700	3.90088700	-3.12063700
C	-0.91526800	-6.78310700	-0.52091700
C	-0.92489800	-6.48583500	-1.90452100
H	-1.74562000	-6.84084400	-2.51993700
H	-1.72933700	-7.36034800	-0.09371400
H	0.08930800	-5.48110100	-3.52308100
H	0.14914400	-6.56726800	1.34651400
C	-3.67440100	-2.30248700	3.72925800
H	-3.37251900	-0.20485800	4.12471900
C	-3.05779200	-3.56888400	3.59814900
H	-1.20015300	-4.67041300	3.65573700
C	-5.41749300	4.14919500	1.55406000
H	-4.24784200	3.64860400	3.29878100
C	-5.39343500	4.66795500	0.23721700
H	-4.16186000	5.46960300	-1.34491700
H	-6.31865700	4.73352600	-0.32639600
H	-6.36027000	3.82362000	1.98187700
H	-3.66842800	-4.43107600	3.35070000
H	-4.74135800	-2.20198900	3.56904500
P	0.91169300	1.18083800	-3.93446600
N	0.12818800	1.27740300	-5.39115200
C	0.70861200	2.06055700	-6.48872400
C	-0.63857200	0.10675900	-5.82930900
H	1.19829700	2.94931400	-6.09276700
H	-0.09973300	2.37655000	-7.15441100
H	1.43338300	1.47104800	-7.06497900
H	-1.08137400	-0.39783700	-4.96729600
H	-0.01290300	-0.61119000	-6.37609300
H	-1.44718600	0.44298400	-6.48465700
Au	-0.22840100	0.15761500	-2.30523600
N	2.12670100	-4.49216200	-2.21403800
N	2.19621600	-5.17000100	0.52426100
N	0.44749700	-2.71639100	4.19859600
N	-0.76934400	-0.18674300	4.44026900
N	-1.88383300	4.39333800	2.47251300
N	-1.82217900	5.35984400	-0.18030700
C	-4.75420600	-2.60673700	-0.71331200
C	-4.38382100	-3.77924400	-1.20629000
H	-4.04312700	-3.85183400	-2.23106500
C	-4.78752100	-1.24357700	-1.35440200
C	-5.72159500	-0.43131900	-0.42668800
H	-5.12026800	-1.28289400	-2.39375300
H	-3.78535800	-0.80526300	-1.32461000
C	-5.66381700	-1.25852900	0.87072400
O	-5.15687600	-2.48908800	0.61762100
O	-6.02126400	-0.90898600	1.96834200
H	-4.35426800	-4.66441000	-0.58426600
C	-5.35067800	1.04484000	-0.18448800
H	-5.91636000	1.37341600	0.69283600
H	-5.72463500	1.62235100	-1.03192200
C	-7.15905700	-0.49690700	-0.95982500
O	-7.77183300	0.43153000	-1.43741800
O	-7.64757200	-1.74789600	-0.86751700

C	-8.97900400	-1.93709700	-1.37526600
H	-9.20492200	-2.99039700	-1.21569700
H	-9.68490200	-1.30300400	-0.83433600
H	-9.01978900	-1.68895100	-2.43844300
C	-3.88153700	1.32038500	0.01357100
C	-3.22545100	0.98346600	1.20486800
C	-3.15074200	1.95654000	-0.99181300
C	-1.87653700	1.27675100	1.38958000
C	-1.80115600	2.26077900	-0.80861400
H	-3.64820600	2.24595000	-1.91351000
C	-1.15742400	1.91897800	0.38119000
H	-1.27057600	2.80172400	-1.58001100
H	-0.10787200	2.15886800	0.52443900
S	-1.22864700	-2.15047900	-0.21315300
O	-0.97430600	-3.26555100	-1.12646400
O	-1.46815500	-0.82431500	-0.94855700
O	-2.18698300	-2.29115300	0.87744000
C	0.40335200	-1.87195500	0.63588400
F	0.67409400	-2.95772800	1.35897800
F	0.37875800	-0.80403100	1.44491200
F	1.39629400	-1.68763400	-0.25954200
H	-3.78028400	0.49300200	1.99513900
H	-1.39540200	1.01652800	2.32434900

(TfO•2a)⊂AuCav-7

C	2.73427700	1.14998200	4.69903200
C	1.65717100	1.99453500	4.02864900
C	3.26331500	0.05955800	3.77505100
H	2.24242400	0.63479900	5.52422600
C	1.97332600	3.12911200	3.27767100
C	0.29799800	1.68807000	4.19010100
C	4.29783600	0.28276500	2.86383000
C	2.73600300	-1.23585800	3.84148800
C	1.00618700	3.98073000	2.74107400
H	3.01853000	3.37303100	3.12925600
C	-0.70618900	2.50750200	3.67273800
O	-0.04199300	0.60885600	5.00710900
C	4.83721900	-0.73074400	2.06747600
H	4.70739200	1.28301200	2.77982500
C	3.24290100	-2.28224700	3.07543700
O	1.73176500	-1.47657600	4.78112900
C	1.37702200	5.20916500	1.91323400
C	-0.33760200	3.65035200	2.96413800
H	-1.74994000	2.27907000	3.84889800
C	-0.47939100	-0.56594900	4.45031700
C	5.94014000	-0.45701300	1.04866600
C	4.29538000	-2.01611800	2.20232900
H	2.83842900	-3.28157000	3.16630600
C	0.45340000	-1.65492800	4.32373400
H	0.53569900	5.89903200	1.97682800
C	1.48368200	4.77258200	0.45741900
O	-1.29936500	4.57884000	2.56664100
C	5.28283600	-0.00223600	-0.24893500
H	6.42089900	-1.41283600	0.84100400
O	4.90623600	-3.05818900	1.50531000
C	2.65431000	4.22326700	-0.06801100
C	0.36869100	4.84007600	-0.38859400
C	-2.19628700	4.30915700	1.57133200
C	-2.13353700	-1.90523000	3.63000800
C	4.97919100	1.33662900	-0.49856200
C	4.90324900	-0.94283900	-1.21747100
C	4.22448000	-3.69641700	0.50208600
C	-1.19197000	-2.96242900	3.44536100
C	2.73895200	3.70763700	-1.36383100
H	3.53974200	4.19771200	0.55557400
C	0.38654900	4.28207700	-1.66632700
O	-0.75399300	5.52310400	0.06356800
O	-1.92311200	4.82955400	0.25885300
C	4.31013400	1.75712800	-1.65013400
H	5.28471200	2.08209300	0.22572400
C	4.18434800	-0.57757500	-2.35438100
O	5.34403400	-2.24718500	-1.05468100
C	4.44861200	-3.26683600	-0.84975400
C	4.05646400	3.22516300	-1.95463200
C	1.55988700	3.68513000	-2.11707800
H	-0.49807700	4.31876200	-2.29023200
C	-4.21043300	3.54642100	0.83432700
C	3.88878000	0.76857300	-2.54449800
H	3.88447700	-1.32893700	-3.07478000
C	2.85263100	-5.34232100	-0.26344700
H	3.95347900	3.30194400	-3.03983800
O	1.55692400	3.13616600	-3.40789900
C	-3.93665700	4.06422500	-0.46915900
O	3.21245600	1.14628900	-3.71597400
C	3.08949600	-4.92374100	-1.60805300
C	-1.60759100	-4.17354400	2.84834800
C	-3.48652300	-2.11352000	3.27401800
C	-4.88574100	3.89782200	-1.50294900
C	-5.44943100	2.91274500	1.08016700
C	2.46985900	-5.61468700	-2.67239400
C	1.97711300	-6.42316100	-0.01464900
C	5.23821100	4.12502600	-1.56050600
H	6.14775700	3.78582800	-2.06325200
H	5.43656500	4.12314000	-0.48595800
C	2.62335000	5.94182900	2.42555700
H	2.80858800	6.83403600	1.82092800
H	3.52849200	5.32893000	2.39269300
C	3.86292500	2.00330700	5.29909800
H	3.45004500	2.73691600	5.99711900
H	4.43472000	2.55113000	4.54464800
C	7.01735800	0.50701600	1.56191200
H	7.47874400	0.10217200	2.46660400
H	6.62654900	1.49774600	1.81100300
H	7.79544400	0.64051400	0.80494900
H	4.56515400	1.36333300	5.84026300
H	2.47510800	6.25049200	3.46379400
H	5.03800900	5.15825900	-1.85653600
C	1.37250000	-7.06981100	-1.07072900
C	1.62596100	-6.66902700	-2.40413400
H	1.13736300	-7.19223600	-3.21990600
H	0.69387100	-7.89576600	-0.88202400
H	2.66277200	-5.27429500	-3.68342600
H	1.80326900	-6.71241000	1.01607000
C	-3.87205800	-3.30890200	2.70205700
H	-4.18673500	-1.30260900	3.44314300
C	-2.92351100	-4.33416300	2.47453900
H	-0.86176200	-4.94125300	2.67833600
C	-6.37107300	2.79246400	0.06223200
H	-5.65103600	2.54658400	2.08108600
C	-6.08042700	3.26445700	-1.23974100
H	-4.64027400	4.27931400	-2.48809800
H	-6.80860200	3.11901800	-2.03053200
H	-7.32692200	2.32441500	0.25569800
H	-3.24125300	-5.25233600	1.99218200
H	-4.90040100	-3.46655900	2.39411900
P	1.60904700	1.52482700	-3.70263600
N	1.18472000	1.53460400	-5.30385900
C	1.79993500	2.49954800	-6.22293100
C	0.83645500	0.25282500	-5.92676900
H	1.96131800	3.44860500	-5.71336700
H	1.11526300	2.66628200	-7.05949700
H	2.75590000	2.12900100	-6.61511200
H	0.34246300	-0.39874300	-5.20235400
H	1.72228100	-0.26369300	-6.31946500
H	0.14148300	0.44136100	-6.74997000
Au	0.40761100	0.13317900	-2.43064000
N	3.90913600	-3.86078900	-1.87107000
N	3.45815900	-4.70592000	0.78501900
N	0.10833700	-2.80727900	3.83421800
N	-1.73368700	-0.69330400	4.12660000
N	-3.30356400	3.68665500	1.84823300
N	-2.76125000	4.71385700	-0.72671000
C	-7.40631000	-0.25663800	0.74861000
C	-8.35762700	0.00207700	1.63778600
H	-8.18664000	-0.20682900	2.68634100
C	-6.02093200	-0.81590200	0.92271000
C	-5.74922000	-1.43346300	-0.45444200
H	-5.96383300	-1.54066500	1.73096200
H	-5.30984100	-0.00381900	1.10504700
C	-6.58280000	-0.51589400	-1.35971000

O	-7.60424200	0.01953500	-0.60612000
O	-6.43982000	-0.24973900	-2.52005500
H	-9.31246300	0.41608600	1.33715900
C	-4.28099200	-1.54151800	-0.93480100
H	-4.31728300	-1.78455400	-1.99935100
H	-3.80636700	-2.38573900	-0.42762700
C	-6.33112900	-2.85596900	-0.49048500
O	-6.48769300	-3.55883800	0.48561100
O	-6.58580600	-3.24841400	-1.74553100
C	-7.04363700	-4.60531300	-1.89130200
H	-7.20982600	-4.74262300	-2.95859200
H	-6.28516900	-5.30039100	-1.52432500
H	-7.96880100	-4.75602700	-1.33085700
C	-3.42093500	-0.32259200	-0.71577800
C	-3.21171200	0.61646700	-1.73101100
C	-2.71892000	-0.18944700	0.48652400
C	-2.24704600	1.61305500	-1.57301200
C	-1.77484000	0.81671500	0.65809900
H	-2.86173700	-0.92889100	1.26263500
C	-1.50735200	1.69594700	-0.38774400
H	-1.22276000	0.88881600	1.58674600
H	-0.73351600	2.44391400	-0.27898300
S	-0.36566100	-2.70480100	-1.01146800
O	0.49185600	-3.44395400	-1.93918400
O	-0.77842700	-1.32698800	-1.53152400
O	-1.51176700	-3.37514300	-0.39528700
C	0.74992100	-2.24935300	0.40704900
F	1.02753600	-3.34402600	1.11516000
F	0.18059800	-1.34254100	1.21272900
F	1.90192500	-1.72105700	-0.05374100
H	-3.77601700	0.53869000	-2.65488400
H	-2.07730500	2.33191100	-2.36844700

(TfO•2a)⊂AuCav-8

C	-5.19338100	-0.86133200	2.75742800
C	-4.26968800	-1.91862000	2.16183400
C	-4.76356300	0.55399700	2.37707000
H	-5.07362500	-0.93309700	3.83900200
C	-4.51428600	-2.45874900	0.89501500
C	-3.17284000	-2.43031600	2.87090000
C	-5.16002500	1.20031300	1.20295400
C	-3.93807500	1.27620300	3.24521900
C	-3.74781000	-3.47956900	0.33118100
H	-5.36720700	-2.08943000	0.33933100
C	-2.34777600	-3.41789400	2.33261100
O	-2.95482900	-2.02687800	4.19460600
C	-4.79119600	2.51850100	0.89892900
H	-5.76911400	0.65589200	0.48968500
C	-3.58011300	2.59425400	3.01635100
O	-3.48833700	0.62294400	4.39305800
C	-4.09766800	-4.09761100	-1.02219800
C	-2.64404800	-3.93010600	1.07220000
H	-1.51752800	-3.81120700	2.90541900
C	-1.91169200	-1.17120600	4.42687700
C	-5.14542800	3.16399800	-0.43991800
C	-4.01995500	3.20615900	1.84659100
H	-2.95172500	3.13207200	3.71280000
C	-2.18114600	0.24229900	4.41137500
H	-3.70746300	-5.11580900	-1.00719300
C	-3.34508600	-3.35589400	-2.11900500
O	-1.91554700	-5.00942400	0.57414700
C	-4.11201300	2.72941500	-1.47723700
H	-5.02356300	4.23976500	-0.30859600
O	-3.66840900	4.53735700	1.62365000
C	-3.78329000	-2.13110000	-2.63547500
C	-2.11714800	-3.84274600	-2.57870500
C	-0.65967300	-4.83666200	0.06559400
C	0.28203300	-0.72429100	4.83909700
C	-4.27395100	1.59743000	-2.28037700
C	-2.91465000	3.44828200	-1.61354600
C	-2.31568700	4.72466200	1.43284200
C	0.04264200	0.66808300	4.62621400
C	-3.01539400	-1.35919000	-3.51382800
H	-4.74302200	-1.74393700	-2.31184800
C	-1.30743800	-3.10064700	-3.42921300
O	-1.65566600	-5.07467700	-2.11151200
C	-0.51415200	-4.95854900	-1.35817700
C	-3.29377000	1.15371800	-3.17592300
H	-5.20228900	1.04294700	-2.21390300
C	-1.89641100	3.02808200	-2.46128900
O	-2.78023500	4.62698600	-0.90135400
C	-1.82999600	4.69028100	0.08879600
C	-3.50900600	-0.03933500	-4.10305200
C	-1.74793100	-1.85555200	-3.84282700
H	-0.32746100	-3.46398900	-3.70866600
C	1.59684500	-4.57954100	0.25482700
C	-2.09818300	1.88202000	-3.21611400
H	-0.96920400	3.58345300	-2.51890300
C	-0.18569800	4.97466300	2.18894400
H	-2.89001800	0.14825000	-4.98437000
O	-0.83034600	-1.05711300	-4.53758300
C	1.73769700	-4.71500500	-1.16076100
O	-1.05128300	1.48361100	-4.06110300
C	0.30728800	4.87214600	0.84713600
C	1.12328600	1.57965600	4.65319000
C	1.57884500	-1.15371200	5.20342900
C	3.01739700	-4.63775300	-1.75371400
C	2.75289200	-4.39188100	1.04348300
C	1.69692600	4.90399400	0.60774700
C	0.72904300	5.14542400	3.25274600
C	-4.96098300	-0.15093700	-4.59518900
H	-5.27462200	0.78767100	-5.05949800
H	-5.66503700	-0.37513700	-3.78954200
C	-5.60818600	-4.17961700	-1.28148600
H	-5.79432700	-4.70684700	-2.22100100
H	-6.08400700	-3.19794600	-1.35839800
C	-6.67503400	-1.11926100	2.44220400
H	-6.96101900	-2.12496900	2.76227500
H	-6.90837200	-1.03102400	1.37747900
C	-6.59440600	2.90246000	-0.87017200
H	-7.28301300	3.28941500	-0.11442000
H	-6.81806100	1.83972600	-1.00136800
H	-6.80350500	3.40365700	-1.81941100
H	-7.29758000	-0.39406700	2.97289700
H	-6.10313600	-4.72456500	-0.47314200
H	-5.04288600	-0.95210500	-5.33427800
C	2.08298900	5.19002600	2.98674500
C	2.56794200	5.05851400	1.66481300
H	3.63159200	5.04027100	1.46112200
H	2.78842400	5.31699400	3.80273100
H	2.07697200	4.77664300	-0.39812000
H	0.33147400	5.23793500	4.25830500
C	2.60562200	-0.23439700	5.29310100
H	1.73075200	-2.20887100	5.40526600
C	2.38464800	1.12959800	4.98400300
H	0.92896200	2.62037000	4.41832700
C	3.99349900	-4.32630900	0.44386700
H	2.64030900	-4.30758900	2.11739600
C	4.12595400	-4.44518900	-0.95847100
H	3.08770400	-4.73971300	-2.83119900
H	5.11378000	-4.38970700	-1.40469200
H	4.87684400	-4.18935300	1.05124700
H	3.21534400	1.82691000	5.00786800
H	3.60050100	-0.56408500	5.57438600
P	-0.17571800	0.16759200	-3.65628700
N	1.23717100	0.44542000	-4.47583500
C	2.17895000	-0.69083300	-4.48445200
C	1.88871400	1.75145700	-4.22086300
H	1.65718000	-1.59836200	-4.79283800
H	2.64401500	-0.84700000	-3.50470300
H	2.95397800	-0.47294800	-5.22344100
H	1.17162400	2.55382900	-4.39131100
H	2.70346200	1.85820600	-4.93837900
H	2.30042300	1.81987000	-3.20924800
Au	-0.25742600	-0.21789500	-1.37169000
N	-0.56569600	4.74089400	-0.20149900
N	-1.52536900	4.87628300	2.45276600
N	-1.23169700	1.12834200	4.44035800
N	-0.72308200	-1.64099300	4.66573600
N	0.36111600	-4.63789400	0.84793900

N	0.63946000	-4.89976300	-1.95145400
C	0.72137100	-0.05567800	1.15433100
C	-0.60339300	-0.30767700	0.85587400
H	-0.97939800	-1.30512800	1.06455200
C	1.80345300	-1.01941500	1.56224900
C	3.10609100	-0.26920000	1.20220000
H	1.69945300	-1.98999300	1.08569000
H	1.72026800	-1.14752500	2.64660300
C	2.61356000	1.18919600	1.32490400
O	1.19624700	1.19776800	1.16124600
O	3.18276800	2.16073100	1.69053000
C	4.32276200	-0.33773100	2.15823400
H	5.04147900	0.37948400	1.75727300
H	3.98270000	0.06316000	3.11432700
C	3.43945100	-0.65039300	-0.25454800
O	2.58683500	-0.99769000	-1.05521000
O	4.73733800	-0.65092300	-0.48031200
C	5.15618400	-0.86290400	-1.84004000
H	6.23647600	-0.73854300	-1.82748200
H	4.87001500	-1.86709000	-2.16132100
H	4.70005500	-0.10636500	-2.47435200
H	-1.31123400	0.51601200	0.92765400
C	5.00860300	-1.65772200	2.41399000
C	6.26112400	-1.92314000	1.84528100
C	4.47939100	-2.58335100	3.32306900
C	6.96676700	-3.07944200	2.17971200
H	6.68204500	-1.20632900	1.14860300
C	5.17958800	-3.74252400	3.65886200
H	3.51727400	-2.38720200	3.78280600
C	6.42982200	-3.99317500	3.08967700
H	7.94084400	-3.26272300	1.73604900
H	4.75330900	-4.44526800	4.36879200
H	6.98098500	-4.89006100	3.35501400
S	4.59186000	2.73224200	-1.18881800
O	5.77662500	2.01523000	-0.68039400
O	3.35911600	1.88567800	-1.28263200
O	4.37133900	4.09935000	-0.68309700
C	4.99477000	3.02777800	-2.98174100
F	6.15681700	3.67342000	-3.11840100
F	4.03317000	3.75939200	-3.57748900
F	5.08875800	1.85952500	-3.66667200

(TfO•3a)⊂AuCav-1

C	4.41087700	-2.40319600	2.70858600
C	4.21800400	-2.54812400	1.20414300
C	3.28780100	-1.56747200	3.31356400
H	5.32780800	-1.82975400	2.84603400
C	3.46656200	-3.58122800	0.64027500
C	4.81158000	-1.63373800	0.32346700
C	2.07811000	-2.13626500	3.71996600
C	3.43961100	-0.18540200	3.49757900
C	3.30596300	-3.74202400	-0.73994900
H	3.00638700	-4.30572900	1.30167600
C	4.65946300	-1.73135200	-1.05639500
O	5.65337700	-0.66449200	0.86100000
C	1.05616100	-1.39841000	4.32561700
H	1.93204400	-3.20100600	3.57641000
C	2.44852200	0.59369500	4.08375100
O	4.66777800	0.39247600	3.15946400
C	2.52781500	-4.91495200	-1.32701000
C	3.90755600	-2.78540100	-1.56881500
H	5.13792400	-1.01997400	-1.71705500
C	5.25762500	0.64409100	0.84295400
C	-0.24950700	-2.04885900	4.77201900
C	1.27137700	-0.02463700	4.50239300
H	2.58366800	1.65963400	4.21603300
C	4.73516700	1.20376100	2.06190600
H	2.87175100	-5.02602400	-2.35572200
C	1.04013600	-4.59126800	-1.40433800
O	3.80921800	-2.94193800	-2.95679300
C	-1.21233100	-2.16579100	3.59448300
H	-0.71100300	-1.35631400	5.47601100
O	0.30079800	0.72245300	5.16041100
C	0.16518500	-4.78989700	-0.32883600
C	0.49908600	-4.05854700	-2.58146600
C	2.98025800	-2.07393800	-3.61101000
C	5.07655600	2.67505400	-0.16956800
C	-1.24332100	-3.28673600	2.76054700
C	-2.10752600	-1.12003800	3.31228600
C	-0.39964400	1.61458400	4.37578400
C	4.54456200	3.22831300	1.03532400
C	-1.19792700	-4.48227100	-0.39262700
H	0.56435600	-5.19088800	0.59581200
C	-0.85086700	-3.75835700	-2.69957700
O	1.35179100	-3.83467000	-3.66465500
C	1.66011800	-2.53411800	-3.95377100
C	-2.13535600	-3.41341600	1.69002200
H	-0.55887300	-4.10171800	2.96325000
C	-3.00936400	-1.19256000	2.25242800
O	-2.12294900	-0.04354200	4.18197200
C	-1.63423000	1.18415400	3.79034300
C	-2.14612400	-4.65146300	0.79398800
C	-1.68247300	-3.96877400	-1.60368800
H	-1.24246200	-3.33677100	-3.61577000
C	2.51455800	-0.05341300	-4.55575600
C	-3.01387700	-2.34248000	1.46617700
H	-3.67463400	-0.36957100	2.02859000
C	-0.63898900	3.64379100	3.38135500
H	-3.15204800	-4.71977100	0.37354500
O	-3.00595400	-3.57643600	-1.71872300
C	1.18708000	-0.49282000	-4.85168700
O	-3.94309800	-2.41416800	0.41329300
C	-1.83407100	3.19194300	2.73805200
C	4.16102300	4.58869500	1.06540600
C	5.24110700	3.50207200	-1.30544500
C	0.27988600	0.39792400	-5.46926300
C	2.90659900	1.26074600	-4.89912100
C	-2.52447800	4.05134300	1.85358800
C	-0.17851200	4.96011100	3.15360900
C	-1.89059800	-5.95285900	1.56835100
H	-2.61663300	-6.05665900	2.37886200
H	-0.89242600	-5.99702600	2.01232100
C	2.80857800	-6.23833600	-0.59952000
H	2.28168300	-7.05702700	-1.09711600
H	2.48863100	-6.22913300	0.44600700
C	4.59802800	-3.75136900	3.41977000
H	5.44566600	-4.29014600	2.98726700
H	3.72184400	-4.40168100	3.34640600
C	-0.02706200	-3.38063300	5.50307800
H	0.60870000	-3.22248800	6.37829400
H	0.45798900	-4.13783500	4.88010800
H	-0.98365600	-3.79151100	5.83771500
H	4.79498500	-3.58734600	4.48245300
H	3.88022000	-6.45411200	-0.61377400
H	-1.98971600	-6.81108000	0.89837700
C	-0.88011000	5.78594000	2.30252400
C	-2.04536100	5.32582600	1.64315700
H	-2.56216300	5.98086500	0.94955100
H	-0.53261800	6.80030400	2.12903500
H	-3.39506200	3.67700300	1.32953100
H	0.72727700	5.27890000	3.65774000
C	4.86653900	4.82771600	-1.24633200
H	5.65294100	3.05776100	-2.20520200
C	4.31708300	5.36999300	-0.05943500
H	3.72928000	4.97426600	1.98172500
C	2.00555200	2.10606700	-5.51277200
H	3.91841800	1.57071600	-4.66160900
C	0.68703600	1.67458400	-5.79401300
H	-0.72468000	0.04548000	-5.67579600
H	-0.00786300	2.35923900	-6.26958500
H	2.30384600	3.11589000	-5.77509900
H	4.00534300	6.40887600	-0.04418800
H	4.97904500	5.46245000	-2.11924800
P	-3.40168100	-2.06494000	-1.08996800
N	-4.75135300	-1.68808300	-1.91393600
C	-6.12261200	-1.80467700	-1.41514300
C	-4.59275100	-1.19825300	-3.28243600
H	-6.11264200	-2.04930100	-0.35568000
H	-6.65245200	-2.59183500	-1.96546800
H	-6.61799100	-0.84150500	-1.54827500

H	-3.53223500	-1.04092900	-3.50568500
H	-5.10880100	-0.23981500	-3.37058300
H	-4.98736900	-1.92648000	-4.00130600
Au	-1.34722200	-0.96578400	-0.83132300
N	-2.31927200	1.93615200	2.98675500
N	0.07558400	2.80834300	4.19407700
N	4.38801200	2.45316400	2.15155900
N	5.41715300	1.35082700	-0.23754700
N	3.39481500	-0.87759100	-3.90875700
N	0.78796700	-1.76394000	-4.53571400
C	0.91087500	-0.64637600	-0.46790700
C	0.55119100	-0.36836200	0.82989500
H	0.27506500	-1.12150400	1.56080900
C	1.49016300	0.45796000	-1.31185600
C	0.96380200	1.84018400	-0.87674500
H	1.24260800	0.32057100	-2.36266600
H	2.58535200	0.43812800	-1.23403800
C	1.06264800	1.97027200	0.63881400
O	0.60277300	0.85834700	1.37810800
O	1.48290700	2.89327200	1.26661000
H	1.15249200	-1.68152200	-0.69975900
C	1.77510200	2.96698500	-1.58750000
H	2.67279500	3.14296400	-0.99918300
H	2.08989200	2.56320100	-2.55231500
C	-0.52289600	2.04535900	-1.19623800
O	-1.29011600	2.53886100	-0.40780100
O	-0.80796300	1.70172200	-2.46341300
C	-2.14103100	2.05025900	-2.92276700
H	-2.05382100	2.14695300	-4.00421600
H	-2.83332200	1.25547900	-2.65131200
H	-2.46805700	2.97787200	-2.45500100
C	1.03445700	4.26520300	-1.81899000
C	0.86494900	5.21029800	-0.80006100
C	0.47813000	4.52316800	-3.07819200
C	0.16166800	6.38871900	-1.04440500
H	1.26351200	5.00862700	0.18599200
C	-0.23086900	5.69920600	-3.32101700
H	0.59323900	3.78582400	-3.86560400
C	-0.38869200	6.63869300	-2.30219400
H	0.03484300	7.11090900	-0.24436800
H	-0.65937300	5.88077200	-4.30242500
H	-0.94121500	7.55535200	-2.48489700
S	-4.76181300	1.64412100	-0.56072700
O	-4.40065700	3.07198900	-0.50478600
O	-5.58616400	1.25168100	-1.72745000
O	-3.66314600	0.68449000	-0.24219800
C	-5.88668600	1.40856900	0.89735100
F	-5.28941300	1.82560700	2.02445400
F	-6.18538900	0.09935900	1.05729900
F	-7.03342900	2.08128700	0.74640900

(TfO•3a)⊂AuCav-2

C	-5.88580000	-1.65000000	-0.81976000
C	-5.49263700	-0.28457300	-0.25984100
C	-4.69324300	-2.56770300	-1.08539700
H	-6.33019200	-1.45320800	-1.79636700
C	-5.31449300	-0.04625500	1.10829800
C	-5.32709600	0.80934800	-1.11979300
C	-4.13564800	-3.38122600	-0.09285600
C	-4.13546200	-2.66579800	-2.36834800
C	-4.99854300	1.21646600	1.62184400
H	-5.41595800	-0.87743700	1.79730700
C	-5.04643800	2.08729200	-0.65703100
O	-5.42997800	0.60050400	-2.50063200
C	-3.09036500	-4.28037100	-0.33739000
H	-4.52803800	-3.30757200	0.91529500
C	-3.12706200	-3.57004900	-2.67004200
O	-4.59728000	-1.81594100	-3.38235200
C	-4.71306800	1.46830000	3.09896700
C	-4.88539000	2.27049600	0.70936800
H	-4.92253800	2.91273600	-1.34378900
C	-4.21321600	0.54028900	-3.12708700
C	-2.45080400	-5.10905600	0.77136200
C	-2.62175100	-4.37085900	-1.65275100
H	-2.71847900	-3.62676500	-3.66943900
C	-3.77426600	-0.74047200	-3.60255500
H	-4.83729600	2.53904600	3.26304700
C	-3.24744800	1.15237600	3.37859500
O	-4.61588600	3.54264400	1.20703500
C	-1.40327200	-4.28175600	1.51310700
H	-1.91069200	-5.91818700	0.27872200
O	-1.60126600	-5.28155000	-1.95472800
C	-2.84007100	-0.13066400	3.75182800
C	-2.24928600	2.13139100	3.25392700
C	-3.41623200	4.10458200	0.86337000
C	-2.24173900	1.46613100	-3.79636600
C	-1.72550600	-3.50512100	2.62940900
C	-0.06555800	-4.27277000	1.08599100
C	-0.41243400	-4.67686600	-2.27252100
C	-1.81135800	0.19488800	-4.28707500
C	-1.51221000	-0.47131200	4.01799900
H	-3.59845600	-0.89473100	3.86794400
C	-0.90647800	1.82669200	3.46512100
O	-2.62241800	3.45566800	3.03599100
C	-2.36227100	4.07259000	1.83795700
C	-0.77233400	-2.76263900	3.33173100
H	-2.75565900	-3.47807900	2.96398400
C	0.92691900	-3.56946400	1.76284500
O	0.26726900	-5.05995800	-0.01485800
C	0.54179000	-4.46793800	-1.21562200
C	-1.12769000	-1.86504300	4.51004700
C	-0.54978800	0.53375500	3.84052800
H	-0.15439100	2.60119600	3.39040600
C	-2.09636400	5.32609300	-0.52986700
C	0.54848300	-2.83518700	2.88226900
H	1.96395500	-3.58558900	1.44590300
C	0.96245900	-3.59627500	-3.72782100
H	-0.21547600	-1.74435600	5.09888000
O	0.78900100	0.29925400	4.14855200
C	-1.07037900	5.35697400	0.46489500
O	1.53433400	-2.13341100	3.59150300
C	1.87772400	-3.32386300	-2.66522100
C	-0.52596700	0.06955800	-4.86217500
C	-1.37229600	2.57850600	-3.87490700
C	0.09807100	6.11571800	0.22634900
C	-1.90925100	6.00954000	-1.75318600
C	3.04719000	-2.57475600	-2.93119900
C	1.24675100	-3.13485500	-5.03357300
C	-2.17931800	-2.48731600	5.43948500
H	-1.81348300	-3.44265400	5.82497100
H	-3.13304200	-2.67961500	4.94110800
C	-5.68681200	0.73722400	4.03276400
H	-5.44964800	0.95923200	5.07693400
H	-5.66442700	-0.34952900	3.91242200
C	-6.95639600	-2.33934400	0.04223400
H	-7.82914700	-1.68905900	0.14719200
H	-6.60172900	-2.57975600	1.04845900
C	-3.48719500	-5.74499900	1.70917300
H	-4.16065200	-6.38841900	1.13664400
H	-4.10450500	-5.00697400	2.22911900
H	-2.98749100	-6.35239500	2.46886500
H	-7.27168200	-3.27491200	-0.42764200
H	-6.70998100	1.06559400	3.83120700
H	-2.37669400	-1.82221300	6.28437500
C	2.39553900	-2.40722600	-5.26824600
C	3.29284400	-2.12362800	-4.21203900
H	4.19323300	-1.55131000	-4.41321500
H	2.61591700	-2.05194400	-6.27028300
H	3.71631800	-2.36556700	-2.11024200
H	0.53865200	-3.36851500	-5.82155600
C	-0.12571300	2.43086000	-4.44489300
H	-1.70126000	3.52385200	-3.45766700
C	0.29589900	1.17416700	-4.94280800
H	-0.21123400	-0.90637100	-5.21170600
C	-0.75453600	6.73248200	-1.96205400
H	-2.70615300	5.96539000	-2.48808400
C	0.24362300	6.79508900	-0.96317600
H	0.86586700	6.13879300	0.98745800
H	1.14599300	7.36988300	-1.14422300
H	-0.61115600	7.26594700	-2.89655400

H	1.28711800	1.07924100	-5.37399100
H	0.55234800	3.27427400	-4.48201400
P	1.80398200	-0.58327000	3.17802500
N	3.27288300	-0.28801400	3.82559800
C	4.10499700	-1.26109900	4.53656500
C	3.87975200	1.02846500	3.61231200
H	3.64872500	-2.24635600	4.49875400
H	4.22995100	-0.94706100	5.58006500
H	5.07615800	-1.31334300	4.03941400
H	3.20495700	1.68411600	3.06004900
H	4.80064100	0.91088900	3.03559400
H	4.08834300	1.50269400	4.57787000
Au	1.44833900	-0.24015400	0.91805600
N	1.63656600	-3.79248200	-1.39829500
N	-0.20321700	-4.27129400	-3.48860200
N	-2.61440100	-0.90693400	-4.16738000
N	-3.47194500	1.60487700	-3.21644900
N	-3.28009800	4.68572300	-0.29098800
N	-1.22884800	4.68305000	1.64940400
C	1.14966100	0.10063700	-1.38607500
C	0.16284800	0.92808900	-0.92361200
H	-0.81982300	0.60022700	-0.60475400
C	2.36378800	0.67280600	-2.06744200
C	2.64353300	2.13281300	-1.62326100
H	2.18127200	0.63334100	-3.14373700
H	3.23770700	0.05967700	-1.84676200
C	1.34525600	2.92642300	-1.52811200
O	0.24431400	2.28773100	-0.95527800
O	1.20766900	4.06744800	-1.86408600
H	0.90520300	-0.94338800	-1.56250500
C	3.40026300	2.14481600	-0.25517900
H	3.09752800	1.25907600	0.30646400
H	4.45376400	1.98952600	-0.49074000
C	3.51577100	2.85116700	-2.66834800
O	4.55763000	3.41821900	-2.43546000
O	2.98897300	2.75229900	-3.90403900
C	3.71465900	3.44341200	-4.93695700
H	3.15299700	3.27006200	-5.85424000
H	3.76593000	4.51032900	-4.71029900
H	4.72827500	3.04612400	-5.02120400
C	3.23509500	3.35370300	0.63237800
C	2.16689300	3.41953500	1.53775500
C	4.17671000	4.38919500	0.62636700
C	2.06333100	4.47065300	2.44836000
H	1.41537200	2.63441800	1.53977400
C	4.07144300	5.44640300	1.53010600
H	4.99110200	4.35318200	-0.08878800
C	3.02366500	5.48354500	2.45278100
H	1.21803600	4.51405100	3.12659200
H	4.81614900	6.23684000	1.52173300
H	2.95022700	6.30082300	3.16404100
S	4.59657500	-1.74753600	0.90445700
O	5.48843600	-0.72525900	1.48145200
O	3.98665200	-2.70468800	1.84934500
O	3.62438600	-1.20812200	-0.10684700
C	5.70356900	-2.79564900	-0.15641600
F	6.16047500	-2.07782300	-1.19910900
F	5.02681600	-3.84766600	-0.64050400
F	6.74720900	-3.24192400	0.54769900

(TfO•1b)⊂AuCav

C	-5.99722600	1.03521100	-1.26797400
C	-5.12049200	1.88606000	-0.35244800
C	-5.24124900	-0.18900600	-1.77761800
H	-6.20720100	1.64945300	-2.14396800
C	-5.06047800	1.69398600	1.03050000
C	-4.34862700	2.92704900	-0.88544000
C	-5.16605600	-1.37191600	-1.03249400
C	-4.60194800	-0.17667200	-3.02496600
C	-4.29750300	2.50471700	1.87699200
H	-5.64826000	0.89629600	1.46886100
C	-3.55977800	3.75361500	-0.09054000
O	-4.46674400	3.17857000	-2.25259000
C	-4.50092200	-2.51562100	-1.48188900
H	-5.65376800	-1.40302000	-0.06489500
C	-3.91380400	-1.28543700	-3.51077900
O	-4.69740000	0.97374600	-3.82401300
C	-4.24835100	2.29435100	3.38693400
C	-3.55166600	3.53257800	1.28391100
H	-2.98846500	4.56225900	-0.52671500
C	-3.41613800	2.86921000	-3.06574700
C	-4.43800500	-3.80838900	-0.66818800
C	-3.86615800	-2.43567300	-2.72916300
H	-3.40862300	-1.24849600	-4.46665800
C	-3.56234700	1.73152700	-3.93614400
H	-3.97217300	3.25317900	3.82596700
C	-3.12543600	1.32000600	3.72273200
O	-2.87653400	4.41514800	2.12947000
C	-3.26495400	-3.76048400	0.30545500
H	-4.21855600	-4.60668200	-1.37714400
O	-3.21109400	-3.57296000	-3.19629700
C	-3.30781700	-0.06470000	3.65508400
C	-1.85034800	1.78202700	4.07340400
C	-1.51241900	4.42875300	2.15677000
C	-1.34794600	3.26624700	-3.92730000
C	-3.37585300	-3.15746700	1.56068300
C	-2.01524600	-4.29680600	-0.03925800
C	-1.83905900	-3.55579200	-3.20100900
C	-1.51343200	2.17159100	-4.83063600
C	-2.28253100	-0.97513900	3.91487400
H	-4.28581200	-0.44623500	3.38568900
C	-0.79473900	0.91137400	4.32829200
O	-1.65080500	3.15960500	4.19709200
C	-0.86128500	3.76969200	3.26114400
C	-2.30190500	-3.02753100	2.44565700
H	-4.34396600	-2.78073200	1.86799500
C	-0.90714700	-4.16361800	0.79814100
O	-1.89806700	-4.98468100	-1.24988300
C	-1.15514700	-4.35763000	-2.22563900
C	-2.48642800	-2.48063800	3.85651600
C	-1.02134200	-0.45873700	4.23499600
H	0.18554200	1.29745500	4.57732800
C	0.51270200	5.12376800	1.38635100
C	-1.05066900	-3.47849200	2.00274400
H	0.05827900	-4.56103600	0.50687900
C	0.16334100	-2.88812700	-4.05081500
H	-1.68790200	-2.92011400	4.45907500
O	0.04105100	-1.32836000	4.44532400
C	1.15899000	4.45973800	2.47406300
O	0.08464200	-3.33663600	2.80448700
C	0.84102800	-3.75483400	-3.13896300
C	-0.45098200	1.81819000	-5.69428700
C	-0.11711700	3.96244000	-3.89047600
C	2.56680400	4.51839600	2.58938300
C	1.28956300	5.84505900	0.45038400
C	2.25233800	-3.78887800	-3.12003000
C	0.91677600	-2.05608100	-4.90855500
C	-3.81620400	-2.91768100	4.49039700
H	-3.90220400	-4.00730600	4.47166700
H	-4.68993100	-2.50870200	3.97624300
C	-5.59961300	1.88072700	3.98627700
H	-5.51717900	1.80311500	5.07374900
H	-5.95750800	0.91629700	3.61542500
C	-7.34499200	0.66982100	-0.62800300
H	-7.87725800	1.57680600	-0.32824000
H	-7.24194800	0.03936900	0.25960700
C	-5.77195400	-4.14370700	0.01438100
H	-6.56579200	-4.22175500	-0.73340900
H	-6.08235000	-3.39011800	0.74385000
H	-5.69430500	-5.09902100	0.54067200
H	-7.96278100	0.12443400	-1.34639500
H	-6.36095200	2.62834600	3.74757900
H	-3.85993500	-2.58091300	5.52961800
C	2.29435900	-2.09516900	-4.86003800
C	2.96362100	-2.96579000	-3.96681700
H	4.04714000	-2.96860100	-3.92663200
H	2.87616300	-1.44618800	-5.50754800
H	2.73851200	-4.43422700	-2.40078100
H	0.38019100	-1.38821500	-5.57318900
C	0.91689900	3.57446500	-4.71800600

H	-0.01239300	4.77582000	-3.18015600
C	0.74416800	2.50394100	-5.62922100
H	-0.59933100	0.98149200	-6.36810600
C	2.66051700	5.90163000	0.59403700
H	0.77440500	6.34559600	-0.36250700
C	3.30081600	5.23282900	1.66479800
H	3.03596100	3.99305800	3.41355100
H	4.38044200	5.27997900	1.75687000
H	3.25693400	6.45502500	-0.12390800
H	1.56998400	2.21395500	-6.27071600
H	1.87782100	4.07487400	-4.65694600
P	0.86088000	-1.92042500	3.10370300
N	2.28398500	-2.46141300	3.69492300
C	3.36432300	-1.48641800	3.89076600
C	2.42513400	-3.71354000	4.44563700
H	3.28833800	-0.67688700	3.16179800
H	4.31479900	-1.99081300	3.71307200
H	3.34142000	-1.07396800	4.90824000
H	1.60185200	-4.38491100	4.20800800
H	2.43656700	-3.51769600	5.52548600
H	3.35735300	-4.18541200	4.13045300
Au	0.85464000	-0.24337800	1.53369900
N	0.13691500	-4.48579700	-2.22236800
N	-1.20250900	-2.84052100	-4.07945700
N	-2.65009200	1.40994400	-4.80580000
N	-2.34517000	3.60673700	-3.05606300
N	-0.84825100	5.07984400	1.24844500
N	0.42934100	3.77816000	3.40959500
C	0.78064300	1.94751300	0.18871200
C	-0.26145700	1.60846900	0.73889100
H	-1.28300700	1.51911100	1.04227600
C	1.93444400	2.58948400	-0.44632300
C	3.04650300	1.71250300	-1.08513700
H	2.40727400	3.21523000	0.31119700
H	1.53600000	3.24986100	-1.22475700
C	3.61670900	0.76097000	-0.02147100
O	3.27222200	0.82352300	1.15582600
O	4.46785900	-0.11007200	-0.50828500
H	4.65902500	-0.84246500	0.18823300
C	2.57855500	0.94801500	-2.35553200
H	2.37059800	1.69112300	-3.12660700
H	3.41126100	0.32877300	-2.69677200
C	4.14477100	2.70501900	-1.52362500
O	4.13339600	3.27430200	-2.59612500
O	5.05581200	2.85644600	-0.56025800
C	6.33177700	3.56819300	-0.79129500
C	1.36429900	0.11706200	-2.06629800
C	0.09268600	0.58709400	-2.39730600
C	1.47816000	-1.06332700	-1.32952800
C	-1.05378900	-0.05893500	-1.94108200
H	0.01429100	1.49815600	-2.96838900
C	0.33346000	-1.70375300	-0.85850200
H	2.44253800	-1.49288400	-1.09836900
C	-0.93759100	-1.19155900	-1.13918100
H	-2.03292500	0.33286400	-2.18031200
H	0.46286100	-2.61204600	-0.29556900
H	-1.82197300	-1.67886000	-0.74688000
S	4.11713300	-3.31093900	0.95700800
O	4.83491000	-2.00187500	1.14455800
O	2.72052000	-3.17167800	0.48950600
O	4.37246400	-4.27773400	2.03166500
C	5.00162900	-3.99575900	-0.52367100
F	4.94965600	-3.10224900	-1.53535200
F	6.28491700	-4.24361200	-0.24995600
F	4.42797500	-5.13578400	-0.94494900
C	6.07136700	5.04232800	-1.09870500
H	7.02825600	5.56642200	-1.18257000
H	5.52176100	5.15732400	-2.03253600
H	5.50251300	5.50690800	-0.28944100
C	7.04653400	3.39586200	0.54646900
H	8.02549300	3.88189700	0.51275600
H	6.46214800	3.84349700	1.35523600
H	7.18666600	2.33566900	0.77157000
C	7.08644700	2.86047100	-1.91663500
H	7.16768300	1.79228300	-1.69688300
H	6.57955200	2.98932700	-2.87370400
H	8.09539700	3.27560400	-1.99645300

1b-TS1$_{5anti}$⊂AuCav

C	-3.60001900	3.69742800	3.15793800
C	-3.49763500	2.19319900	3.39073200
C	-2.94483700	4.10659600	1.84344100
H	-3.01140500	4.16476000	3.94797800
C	-4.48545200	1.30433400	2.96305400
C	-2.40960200	1.65311300	4.09315700
C	-3.60842100	4.02089800	0.61573800
C	-1.62708400	4.58396000	1.82814700
C	-4.44927800	-0.06430600	3.24387900
H	-5.33438500	1.69869000	2.41703800
C	-2.31761400	0.29380700	4.37877300
O	-1.46448000	2.53086100	4.61021100
C	-3.00896200	4.38830800	-0.59361400
H	-4.62220000	3.63485900	0.59925100
C	-1.00200100	4.98933600	0.65469500
O	-0.93173300	4.66727700	3.03153400
C	-5.55624700	-1.00930700	2.79015500
C	-3.34747600	-0.54357100	3.96225000
H	-1.47709800	-0.09395300	4.93975700
C	-0.22295200	2.59457000	4.02894800
C	-3.70447600	4.23879100	-1.93942100
C	-1.69993300	4.87515900	-0.54234100
H	0.01958700	5.34493200	0.67042100
C	0.06444800	3.73407700	3.20907700
H	-5.50680000	-1.88152300	3.44230600
C	-5.25774000	-1.50527000	1.38073800
O	-3.34579200	-1.88939300	4.35196500
C	-3.62417300	2.79739400	-2.42807700
H	-3.12940700	4.83194700	-2.65099700
O	-1.08140300	5.23005000	-1.74472800
C	-5.63204400	-0.78944000	0.23992200
C	-4.55089500	-2.69970100	1.18748400
C	-2.44019600	-2.72854200	3.77206400
C	1.89620000	1.83110200	3.70783700
C	-4.63453900	1.87645100	-2.13830800
C	-2.54579300	2.35331700	-3.21412900
C	-0.04950700	4.38814000	-2.08353800
C	2.18092600	2.96359500	2.88155500
C	-5.35129000	-1.22421300	-1.05941500
H	-6.16392800	0.14525800	0.36942600
C	-4.24320900	-3.17330100	-0.08349500
O	-4.19716800	-3.44598200	2.30268400
C	-2.87287500	-3.51665900	2.64981300
C	-4.65473900	0.57531600	-2.64539300
H	-5.45684400	2.19845400	-1.51198900
C	-2.53744900	1.07093600	-3.76295100
O	-1.55190200	3.25191400	-3.57130700
C	-0.31546400	3.28577500	-2.96177200
C	-5.75631900	-0.42080300	-2.29415800
C	-4.65216200	-2.42981300	-1.18159400
H	-3.70229900	-4.10327300	-0.20223900
C	-0.38700200	-3.70628700	3.64699100
C	-3.59928000	0.21482600	-3.48819800
H	-1.73073200	0.76858100	-4.41891500
C	2.11018400	3.70037000	-1.85266400
H	-5.82145300	-1.12793200	-3.12443700
O	-4.36936000	-2.94644800	-2.45822800
C	-0.79338600	-4.42639800	2.48067500
O	-3.62802600	-1.04634300	-4.11853800
C	1.85123400	2.58902500	-2.71836300
C	3.45570900	3.08748200	2.28352900
C	2.90644600	0.86689600	3.93087600
C	0.13444100	-5.26931500	1.82499500
C	0.91876500	-3.88685800	4.15710500
C	2.90387900	1.68866300	-2.99797200
C	3.38780600	3.86453900	-1.27421500
C	-7.13985400	0.22948600	-2.15530700
H	-7.40125300	0.75266800	-3.07874900
H	-7.19309100	0.95443500	-1.33934000
C	-6.96170500	-0.40666500	2.92529900
H	-7.71692600	-1.14384500	2.63855100

H	-7.11206200	0.47892800	2.30167000
C	-5.03840100	4.22324200	3.27882900
H	-5.46142900	3.94741600	4.24873100
H	-5.70432300	3.83177000	2.50414900
C	-5.13775700	4.79067000	-1.92312000
H	-5.11682200	5.85457100	-1.67343400
H	-5.77785800	4.29789900	-1.18569800
H	-5.60581400	4.67307000	-2.90464300
H	-5.04428100	5.31319600	3.19336700
H	-7.14076600	-0.11057100	3.96211600
H	-7.89454300	-0.53738500	-1.96285100
C	4.38625800	2.95295200	-1.53992000
C	4.14164300	1.86675700	-2.41384900
H	4.93599800	1.15314600	-2.58961600
H	5.35921400	3.05002900	-1.07192100
H	2.72356100	0.85372400	-3.66524400
H	3.54121700	4.71668600	-0.62153600
C	4.13915400	1.01261400	3.32983000
H	2.67200800	0.01900700	4.56575600
C	4.41786200	2.12280400	2.49508300
H	3.64251400	3.94768800	1.65221700
C	1.79658400	-4.73216300	3.51413000
H	1.20239500	-3.32990600	5.04296400
C	1.40751600	-5.41429900	2.33675600
H	-0.19364900	-5.80256700	0.93856700
H	2.12425700	-6.05770200	1.83647300
H	2.80703900	-4.84773500	3.88492500
H	5.38556700	2.20543700	2.00965800
H	4.90411900	0.25718100	3.47428800
P	-3.10063100	-2.34494600	-3.28331300
N	-3.00244600	-3.47079900	-4.51469500
C	-2.86812900	-4.87564800	-4.10590700
C	-2.14225700	-3.10761400	-5.65060500
H	-3.57543400	-5.10192500	-3.30863200
H	-1.84817900	-5.11608800	-3.77065500
H	-3.10691100	-5.50574000	-4.96660400
H	-2.33783000	-2.07898600	-5.95209900
H	-2.38997800	-3.76435000	-6.48850000
H	-1.07380100	-3.22227800	-5.41712100
Au	-1.19672300	-1.70624700	-2.19444100
N	0.60094400	2.40697700	-3.26067100
N	1.11933800	4.58705800	-1.55234500
N	1.22356500	3.91221700	2.64901700
N	0.66026300	1.67176500	4.27226600
N	-1.24042800	-2.83236600	4.26214900
N	-2.07358900	-4.31520500	2.00556200
C	1.36542400	-1.74565200	-1.60764900
C	0.78297800	-0.87445200	-2.26814500
H	0.81739400	0.04772100	-2.82341900
C	1.90425500	-2.75925900	-0.70397400
C	2.75192400	-2.07914600	0.40340300
H	2.52745200	-3.45203900	-1.27374000
H	1.08306500	-3.32219700	-0.24761500
C	3.66540600	-1.06398600	-0.31589300
O	3.65560300	-0.97226200	-1.53510500
O	4.45947600	-0.42323700	0.50194200
H	5.25544700	-0.01589600	-0.02926200
C	1.89633700	-1.47915900	1.53910400
H	2.57077600	-0.93082900	2.19594500
H	1.50105000	-2.31198900	2.12132700
C	3.78306200	-3.05438700	1.01585100
O	3.92703200	-3.22787200	2.20636700
O	4.52697900	-3.58368900	0.04091600
C	5.87485600	-4.15306500	0.30679500
C	0.76934100	-0.58439200	1.08705200
C	-0.54907800	-1.05585900	1.04327200
C	1.02193700	0.73500300	0.69029400
C	-1.59421000	-0.23948700	0.59572200
H	-0.76020200	-2.07007600	1.36098700
C	-0.01602800	1.55591700	0.25313500
H	2.03568800	1.12106800	0.72452300
C	-1.32676400	1.07346400	0.19660700
H	-2.61328000	-0.61177200	0.59457800
H	0.19781200	2.58000200	-0.02276700
H	-2.13311300	1.71998800	-0.12928900
S	7.53960600	1.10489400	-0.15842700
O	8.56464600	0.34420700	0.56253100
O	6.46927100	0.24799200	-0.80078700
O	6.98494500	2.30549000	0.50393000
C	8.37488600	1.77114500	-1.67309800
F	8.91084400	0.77947000	-2.39504300
F	7.48438800	2.42257000	-2.44552500
F	9.34423200	2.62784100	-1.33274600
C	6.73572800	-3.09605200	0.99763500
H	6.77457600	-2.17504600	0.41111100
H	6.36038400	-2.86644500	1.99553700
H	7.75682000	-3.47623100	1.09404500
C	6.38579900	-4.43617700	-1.10290600
H	6.41713800	-3.51137000	-1.68424100
H	7.39562800	-4.85251800	-1.05419300
H	5.73737700	-5.15367200	-1.61491000
C	5.72867000	-5.43549100	1.12371800
H	5.35634300	-5.21891000	2.12512200
H	5.04156200	-6.12817700	0.62662400
H	6.70309200	-5.92611000	1.20784100

1b-Int$_{5anti}$⊂AuCav

C	-5.11910000	2.59309300	2.04265700
C	-3.91069700	2.05281800	2.80199400
C	-5.01324900	2.30404900	0.54940600
H	-5.07962800	3.67779100	2.14590500
C	-3.89199700	0.76436500	3.33726100
C	-2.78614900	2.85739800	3.03575000
C	-5.43806000	1.10012000	-0.01840900
C	-4.47291700	3.26346500	-0.31521200
C	-2.84228100	0.27611300	4.11913600
H	-4.74850000	0.12310500	3.16692400
C	-1.69759100	2.40438500	3.77744100
O	-2.83674300	4.19003000	2.62553300
C	-5.33754900	0.82931300	-1.38718200
H	-5.85513300	0.33892900	0.63143900
C	-4.35864300	3.04693400	-1.68335800
O	-4.08127300	4.48688800	0.22591200
C	-2.89059900	-1.12236700	4.72643500
C	-1.74390200	1.12186400	4.32001800
H	-0.85033900	3.05269200	3.96255100
C	-2.08730400	4.61879400	1.56520500
C	-5.79566600	-0.49432700	-1.99212700
C	-4.77533200	1.82310400	-2.19693000
H	-3.91717100	3.79699300	-2.32560200
C	-2.74139800	4.76129100	0.29200600
H	-2.18895500	-1.12381600	5.56046300
C	-2.36512100	-2.12780000	3.71049700
O	-0.72589700	0.70312800	5.18752900
C	-4.71632400	-1.55896400	-1.83305500
H	-5.90187800	-0.32287400	-3.06347300
O	-4.63106600	1.56303300	-3.56588800
C	-3.17470500	-2.70759400	2.73135800
C	-1.00419300	-2.45485300	3.69454900
C	0.49526300	0.36660600	4.67376200
C	-0.16755400	5.44288700	0.65993500
C	-4.62748300	-2.36102800	-0.69139600
C	-3.75763600	-1.76278100	-2.83568400
C	-3.34225300	1.33144900	-3.97452300
C	-0.80258900	5.53153500	-0.61601200
C	-2.68037500	-3.58584500	1.76202300
H	-4.23094200	-2.46682100	2.72301100
C	-0.45728200	-3.30142600	2.73620700
O	-0.20862200	-1.93032600	4.70721500
C	0.75351500	-1.01817100	4.37398300
C	-3.65413100	-3.35020300	-0.53008800
H	-5.35607200	-2.21813300	0.09746000
C	-2.76064400	-2.72708500	-2.71254800
O	-3.85919200	-1.01820400	-4.01176600
C	-2.93630900	-0.02444000	-4.21757700
C	-3.58164100	-4.22763700	0.71356400
C	-1.30468500	-3.84965100	1.77896300
H	0.60183200	-3.52646100	2.73690700
C	2.61858100	0.87862500	4.01928000
C	-2.72362900	-3.51352500	-1.56437300

H	-2.03642200	-2.86645000	-3.50477600
C	-1.23977200	2.04648200	-4.49011600
H	-3.09086600	-5.15516200	0.40844600
O	-0.75200900	-4.75398400	0.87242700
C	2.83946600	-0.48123800	3.63810100
O	-1.77601400	-4.53503800	-1.48871100
C	-0.85817700	0.70600400	-4.80338800
C	-0.05768900	5.96640800	-1.73656900
C	1.18907500	5.82339500	0.78832700
C	4.06982700	-0.85822300	3.05340100
C	3.65754600	1.82086500	3.85044600
C	0.45773300	0.44814500	-5.25255700
C	-0.27662700	3.07991200	-4.54790700
C	-4.96293800	-4.60994300	1.26329400
H	-5.54539900	-5.11778400	0.48993400
H	-5.54460000	-3.74746700	1.59870900
C	-4.27307600	-1.49242500	5.28139500
H	-4.23506000	-2.47743000	5.75512700
H	-5.05023400	-1.52726000	4.51282700
C	-6.45496300	2.12227500	2.63724600
H	-6.51048000	2.38788100	3.69651400
H	-6.60170800	1.04110500	2.56057800
C	-7.16297900	-0.94262300	-1.45477100
H	-7.91373100	-0.17554900	-1.66272600
H	-7.16280500	-1.11430700	-0.37469300
H	-7.47259000	-1.87351500	-1.93817200
H	-7.28578600	2.60252900	2.11305800
H	-4.58477000	-0.75828400	6.02914000
H	-4.85304100	-5.28356700	2.11737300
C	1.01288400	2.79232100	-4.93751700
C	1.37291000	1.47664900	-5.31467200
H	2.39220200	1.27377200	-5.62572200
H	1.76697700	3.57147400	-4.93380100
H	0.72033200	-0.57138000	-5.51443700
H	-0.58247600	4.07910100	-4.25921900
C	1.89721900	6.22792900	-0.32281100
H	1.65478200	5.74317800	1.76426000
C	1.27309000	6.29414100	-1.59057300
H	-0.56404400	6.02212400	-2.69414200
C	4.85925000	1.42330800	3.30339900
H	3.47446900	2.84910600	4.14026000
C	5.06063200	0.08650300	2.88736800
H	4.19490400	-1.88682900	2.74031800
H	6.00075800	-0.19796300	2.42691000
H	5.64975800	2.15025700	3.16923600
H	1.85523400	6.59632600	-2.45432800
H	2.95009600	6.47033900	-0.23133000
P	-0.34409800	-4.32455000	-0.66342900
N	0.54415900	-5.65684900	-1.07205200
C	0.10674700	-7.00919200	-0.72287500
C	1.46907800	-5.59215400	-2.20593600
H	-0.50217500	-6.98567300	0.17997600
H	0.99046100	-7.62492500	-0.52854700
H	-0.46990600	-7.46519700	-1.53831000
H	1.73728900	-4.55576200	-2.41752500
H	1.02169100	-6.03162100	-3.10751000
H	2.38694100	-6.12936800	-1.95351600
Au	0.64744200	-2.31208800	-1.17440500
N	-1.74239900	-0.32468100	-4.63969600
N	-2.51830400	2.33046100	-4.10097000
N	-2.11880600	5.19109000	-0.76495400
N	-0.84537300	4.95987600	1.74624100
N	1.40712000	1.28086600	4.51734200
N	1.87107400	-1.42410100	3.85053000
C	2.30261800	0.28003300	-1.57229200
C	1.58432300	-0.72583200	-2.07520700
H	1.23449100	-0.53080900	-3.08654200
C	3.03795700	0.49772300	-0.27701700
C	2.98835100	2.02739800	-0.10977400
H	4.07221100	0.17347700	-0.39336100
H	2.59528100	-0.05550200	0.54749100
C	2.81759900	2.49809000	-1.57155300
O	2.48385900	1.44154300	-2.35191800
O	2.95420500	3.61203100	-2.01277200
H	3.12253400	-1.85066600	-1.87497600
C	1.79822100	2.48846700	0.76578700
H	1.68488800	3.56117900	0.63291300
H	2.07964400	2.32352400	1.80619200
C	4.28561000	2.69732200	0.34491700
O	4.34019300	3.64107700	1.10335900
O	5.32535800	2.13743000	-0.29498800
C	6.64212700	2.79395700	-0.38390500
C	0.51525600	1.76303100	0.44852800
C	0.12206400	0.64352600	1.19139400
C	-0.28132900	2.15715600	-0.63168700
C	-1.02900200	-0.07299300	0.86600800
H	0.73464000	0.32151900	2.02260700
C	-1.43528400	1.44827000	-0.95626400
H	0.00570400	3.01658800	-1.22901600
C	-1.81550000	0.33024500	-0.21360100
H	-1.31557100	-0.93409800	1.45575300
H	-2.04039300	1.78444700	-1.78235700
H	-2.71684800	-0.21534000	-0.46626100
S	4.19345000	-3.19057500	-0.50036600
O	4.36913700	-4.61222300	-0.74829400
O	3.29079000	-2.70466000	0.53844200
O	3.96640400	-2.42538600	-1.87672300
C	5.88451400	-2.52122500	-0.07832600
F	6.18317700	-2.91248100	1.16475200
F	5.88872800	-1.18200800	-0.11723700
F	6.79362500	-2.98679300	-0.92732800
C	6.46544000	4.17407000	-1.01807000
H	5.88940700	4.09209900	-1.94359600
H	5.94240800	4.85196700	-0.34275000
H	7.44718100	4.59701600	-1.25140300
C	7.41043900	1.85037300	-1.30758600
H	6.92882100	1.79873900	-2.28775900
H	8.43634800	2.20499700	-1.44003100
H	7.43771500	0.84254500	-0.88467000
C	7.29655400	2.86740200	0.99451500
H	6.73454300	3.52762300	1.65481900
H	7.35687200	1.87104300	1.44205400
H	8.31493300	3.25478000	0.89245600

1b-TS2$_{5anti}$⊂AuCav

C	-5.12842000	2.56412700	2.03649700
C	-3.92750000	2.01367000	2.80023400
C	-5.00926000	2.29468300	0.54054000
H	-5.08970500	3.64740700	2.15414400
C	-3.91459100	0.72005800	3.32335000
C	-2.80441800	2.81476400	3.05157900
C	-5.43017700	1.09849500	-0.04582800
C	-4.46360700	3.26526300	-0.30828900
C	-2.87158100	0.22356000	4.10908500
H	-4.77050000	0.08145300	3.14051100
C	-1.72210600	2.35404000	3.79729200
O	-2.85188000	4.15177500	2.65496300
C	-5.32395100	0.84603400	-1.41768200
H	-5.85054100	0.32870600	0.59158700
C	-4.34189900	3.06676600	-1.67848100
O	-4.07514200	4.48191300	0.25035200
C	-2.92499600	-1.18029200	4.70360200
C	-1.77403800	1.06630900	4.32686000
H	-0.87620200	2.99985100	3.99668200
C	-2.09258100	4.59302700	1.60783100
C	-5.78225000	-0.46993400	-2.03962200
C	-4.75732000	1.85049500	-2.21115400
H	-3.89655500	3.82496800	-2.30844300
C	-2.73599900	4.75429600	0.33112500
H	-2.23016400	-1.18936500	5.54323900
C	-2.39101200	-2.17563700	3.68229800
O	-0.76318400	0.63776000	5.19679900
C	-4.70609300	-1.53798100	-1.88277500
H	-5.88038300	-0.28726600	-3.10989200
O	-4.60338400	1.61074500	-3.58299900
C	-3.19256100	-2.74063200	2.68790000
C	-1.03016900	-2.50443300	3.67533300
C	0.46044000	0.30685200	4.68414700
C	-0.16494200	5.42988800	0.73105700
C	-4.62590900	-2.35385500	-0.75002400

C	-3.73604900	-1.72527200	-2.87733600
C	-3.31093600	1.38333500	-3.98295400
C	-0.78962300	5.53919100	-0.54849800
C	-2.69025300	-3.60420300	1.70955300
H	-4.24834900	-2.49811700	2.67349200
C	-0.47546300	-3.33764800	2.70958300
O	-0.24210800	-1.99122100	4.69954800
C	0.72186500	-1.07553900	4.37815100
C	-3.64715800	-3.33811800	-0.58902400
H	-5.36402600	-2.22482300	0.03230300
C	-2.73477200	-2.68475600	-2.75527800
O	-3.82867800	-0.96424400	-4.04308400
C	-2.90152900	0.02877900	-4.22848500
C	-3.58236800	-4.23123900	0.64404300
C	-1.31473100	-3.86812300	1.73505900
H	0.58383900	-3.56249600	2.71431000
C	2.58150500	0.82844100	4.03004500
C	-2.70450300	-3.48282200	-1.61526300
H	-2.00187700	-2.81195700	-3.54147400
C	-1.20480900	2.10213600	-4.47800800
H	-3.08774300	-5.15378500	0.32996200
O	-0.75293900	-4.75381200	0.81709300
C	2.80640100	-0.52946400	3.64353100
O	-1.75299400	-4.49980200	-1.54575100
C	-0.81615800	0.76155100	-4.78313100
C	-0.03818300	6.00092200	-1.65373100
C	1.18933400	5.81206100	0.87811900
C	4.03426500	-0.90021300	3.04992800
C	3.61579400	1.77560200	3.85908500
C	0.50262300	0.50570600	-5.22397000
C	-0.24692900	3.13994300	-4.54404600
C	-4.96716100	-4.62325500	1.17790300
H	-5.54345200	-5.12039100	0.39306400
H	-5.55216100	-3.76646400	1.52200000
C	-4.31204300	-1.55532200	5.24358800
H	-4.27781300	-2.54456300	5.70864400
H	-5.08278800	-1.58324100	4.46827600
C	-6.47008400	2.08638800	2.61226900
H	-6.53549800	2.33868800	3.67420000
H	-6.61642400	1.00634100	2.52064300
C	-7.15442600	-0.91942700	-1.51604400
H	-7.90150100	-0.14802300	-1.72106000
H	-7.16195600	-1.10222200	-0.43782600
H	-7.46355500	-1.84429500	-2.01114700
H	-7.29574200	2.57358500	2.08636300
H	-4.63003900	-0.82809100	5.99542000
H	-4.86239300	-5.30919900	2.02271000
C	1.04294500	2.85644900	-4.93591200
C	1.41145400	1.53893100	-5.29802100
H	2.43086500	1.33921000	-5.61119500
H	1.78987800	3.64235500	-4.94899200
H	0.76906200	-0.51409200	-5.48071000
H	-0.55860200	4.14063900	-4.26673700
C	1.90277100	6.24691200	-0.21800600
H	1.64818500	5.71309900	1.85551600
C	1.28788500	6.33826300	-1.48862300
H	-0.53649700	6.07239400	-2.61446800
C	4.81553700	1.38418800	3.30271500
H	3.43053300	2.80211300	4.15379200
C	5.01980400	0.04964500	2.88040200
H	4.15905800	-1.92452800	2.72422200
H	5.95658400	-0.23078200	2.41113600
H	5.60301500	2.11462200	3.16821500
H	1.87362000	6.66597300	-2.34062900
H	2.95258200	6.49690500	-0.11165100
P	-0.32777000	-4.30676500	-0.70837500
N	0.56011600	-5.63085300	-1.12795600
C	0.12017200	-6.98867200	-0.80483500
C	1.56596600	-5.55272600	-2.19048700
H	-0.55258900	-6.97423000	0.05171800
H	0.99670400	-7.59072100	-0.54569100
H	-0.38931600	-7.45343100	-1.65878300
H	1.83834200	-4.51444700	-2.38411600
H	1.18844000	-5.99885100	-3.11989400
H	2.47113300	-6.07458400	-1.87036000

Au	0.71746900	-2.32421800	-1.17263900
N	-1.70121000	-0.27025700	-4.63215800
N	-2.48672400	2.38366900	-4.09727300
N	-2.10409900	5.19847600	-0.71427500
N	-0.85136300	4.92848300	1.80371700
N	1.36896000	1.22484200	4.53057500
N	1.84051300	-1.47584800	3.85343600
C	2.35253000	0.27285700	-1.58040000
C	1.86896000	-0.85497800	-2.13956900
H	1.54467400	-0.70995700	-3.16871200
C	3.04414600	0.51765100	-0.26789800
C	2.93619200	2.04039600	-0.08406600
H	4.08780800	0.22099000	-0.37953800
H	2.61691400	-0.06869500	0.54120900
C	2.71253800	2.51899700	-1.52921700
O	2.37630100	1.44650600	-2.31267600
O	2.79525000	3.63036100	-1.97849000
H	2.98950400	-1.79506400	-1.93241100
C	1.73974200	2.44567700	0.81184200
H	1.59871800	3.51867400	0.71162900
H	2.03224700	2.25558000	1.84440700
C	4.21866100	2.74877600	0.36078600
O	4.25401000	3.67761000	1.13786400
O	5.25922300	2.23215200	-0.30885600
C	6.57445700	2.89724500	-0.37126400
C	0.47692100	1.69508000	0.47415200
C	0.11423400	0.54629400	1.18786100
C	-0.33020400	2.09627100	-0.59642700
C	-1.02000700	-0.18863600	0.84484400
H	0.73485900	0.21785400	2.01056500
C	-1.46479100	1.36495500	-0.94072100
H	-0.07002500	2.98125500	-1.16858100
C	-1.81672700	0.22112200	-0.22482400
H	-1.28582500	-1.06893800	1.41391800
H	-2.07840600	1.70527600	-1.75877600
H	-2.70430100	-0.33991900	-0.48988400
S	4.19707500	-3.17078300	-0.50534700
O	4.39752600	-4.61021800	-0.63760300
O	3.28872400	-2.66072400	0.53008000
O	3.95751900	-2.48661300	-1.87419600
C	5.87196300	-2.47730100	-0.06496000
F	6.15672100	-2.80074500	1.20417500
F	5.87849000	-1.13424400	-0.16389800
F	6.81352400	-2.96741600	-0.86697600
C	6.39159900	4.29477900	-0.96327600
H	5.82844300	4.23677900	-1.89873600
H	5.85482200	4.94604000	-0.27244500
H	7.37153300	4.73428900	-1.17193600
C	7.35068800	1.98346100	-1.31768400
H	6.87758500	1.96171200	-2.30316400
H	8.37715400	2.34304600	-1.43071800
H	7.37482300	0.96306500	-0.92581200
C	7.21948400	2.93073100	1.01283500
H	6.65156000	3.56771400	1.69084700
H	7.28162600	1.92091200	1.42844800
H	8.23661900	3.32530600	0.92845100

(TfO•2b)⊂AuCav

C	-5.35678000	1.04654300	2.79520300
C	-4.03307300	0.53184800	3.35408000
C	-5.29798400	1.23697000	1.28304300
H	-5.49632000	2.04152500	3.21925300
C	-3.74465000	-0.83089100	3.47091900
C	-3.06031000	1.43114600	3.81141100
C	-5.53414200	0.18829100	0.38869800
C	-5.00867200	2.49159000	0.73069400
C	-2.56472300	-1.30997200	4.04808000
H	-4.47645500	-1.54775500	3.11680800
C	-1.86778000	1.00647500	4.38519600
O	-3.34929300	2.79617700	3.75730800
C	-5.49683700	0.34538800	-1.00126400
H	-5.74509000	-0.79579800	0.79191700
C	-4.97792500	2.70332100	-0.64229400
O	-4.76484700	3.56805400	1.58718400
C	-2.25855600	-2.79956500	4.17170500

C	-1.64293200	-0.36064000	4.50576600
H	-1.13921600	1.72138500	4.74390100
C	-2.69893900	3.54465700	2.81684700
C	-5.72431800	-0.82241000	-1.95650100
C	-5.21304800	1.62663300	-1.49016100
H	-4.74306600	3.68015800	-1.04318500
C	-3.45636700	3.97132800	1.67316700
H	-1.52129200	-2.89947900	4.96850100
C	-1.58434200	-3.28582900	2.89396000
O	-0.50597700	-0.81681200	5.17248700
C	-4.43780600	-1.62212900	-2.14184400
H	-5.95479600	-0.38393600	-2.92791300
O	-5.16098600	1.82900300	-2.87471100
C	-2.32202100	-3.71405700	1.78790000
C	-0.18714600	-3.28238100	2.77229300
C	0.70665400	-0.64412300	4.56440500
C	-0.87427100	4.64126800	2.01356500
C	-4.09338900	-2.69469200	-1.31280900
C	-3.54033300	-1.29368000	-3.16698600
C	-3.89500700	2.03961100	-3.36018300
C	-1.62969300	5.07290300	0.88052700
C	-1.73002200	-4.13889100	0.59531500
H	-3.40280400	-3.72294700	1.85985800
C	0.45071400	-3.66432400	1.59498700
O	0.56916400	-2.94564600	3.89138700
C	1.27644900	-1.77054400	3.87689000
C	-2.92661300	-3.44420900	-1.48771000
H	-4.76858400	-2.96666800	-0.51028100
C	-2.36391200	-2.00518000	-3.37504100
O	-3.88122700	-0.25733600	-4.03324900
C	-3.19401500	0.92151400	-3.92513100
C	-2.56233300	-4.61952800	-0.58814900
C	-0.33180200	-4.08378300	0.52409200
H	1.52795800	-3.63108100	1.51244900
C	2.55457300	0.58693800	4.06752600
C	-2.07531100	-3.07662300	-2.53780500
H	-1.69689100	-1.73331400	-4.18222600
C	-2.07092900	3.35549600	-3.71399800
H	-1.91716800	-5.27388800	-1.17915700
O	0.32298000	-4.52860400	-0.62969900
C	3.11311700	-0.52697300	3.36526300
O	-0.94103500	-3.83839500	-2.80524600
C	-1.36324200	2.23561100	-4.25023700
C	-0.99756300	5.83666300	-0.12698200
C	0.49198800	4.99145700	2.11174700
C	4.36601400	-0.39145000	2.72378600
C	3.27579100	1.80062800	4.13859500
C	-0.02754500	2.40126800	-4.67756000
C	-1.42467700	4.60795800	-3.61419500
C	-3.77935100	-5.45416200	-0.16505100
H	-4.30531900	-5.82236000	-1.04997400
H	-4.49910700	-4.89052100	0.43422400
C	-3.48432500	-3.63619500	4.56330300
H	-3.20120800	-4.68586600	4.68101600
H	-4.28889700	-3.59343000	3.82368900
C	-6.55539900	0.18820800	3.22773600
H	-6.59585100	0.12207900	4.31839500
H	-6.51381800	-0.83259800	2.83732600
C	-6.91824300	-1.69593300	-1.54436900
H	-7.82606000	-1.08840000	-1.49978800
H	-6.78887300	-2.16196800	-0.56351500
H	-7.07012200	-2.49655600	-2.27361400
H	-7.48587100	0.63788700	2.87047400
H	-3.89299200	-3.27750400	5.51183400
H	-3.45602600	-6.31141400	0.43130900
C	-0.11606100	4.73747200	-4.02546600
C	0.58231500	3.63108400	-4.56170700
H	1.61745900	3.75094600	-4.86088600
H	0.39259800	5.69054400	-3.92888900
H	0.49346700	1.53849300	-5.07719000
H	-1.98827400	5.43901500	-3.20575900
C	1.09153300	5.71730900	1.10356700
H	1.04894100	4.64497300	2.97559900
C	0.34346700	6.13979200	-0.02045700
H	-1.59108700	6.14314300	-0.98008000
C	4.49937200	1.90688500	3.51174800
H	2.83055900	2.62912500	4.67848000
C	5.03828900	0.81174300	2.79593100
H	4.76883200	-1.22970500	2.17210000
H	5.99476500	0.91583900	2.29370300
H	5.04231600	2.84441200	3.54605800
H	0.83953300	6.69026800	-0.81212400
H	2.15261800	5.93292300	1.15643000
P	0.46977900	-3.58923500	-1.96250200
N	1.54507900	-4.48172000	-2.83082900
C	2.23952100	-5.68118400	-2.36287700
C	2.10461200	-3.88321800	-4.04191600
H	1.78948200	-6.04516300	-1.44304300
H	3.29095100	-5.44401400	-2.17664700
H	2.16507400	-6.46097800	-3.12970500
H	1.57492200	-2.96057800	-4.29219200
H	1.98947500	-4.57590300	-4.88302600
H	3.16202900	-3.65039100	-3.88698500
Au	0.97540500	-1.36990400	-1.59217000
N	-1.96787800	1.01170600	-4.34922900
N	-3.35554700	3.21880000	-3.26611800
N	-2.94248400	4.71444200	0.73832100
N	-1.45068900	3.86841500	2.98376600
N	1.32407600	0.49524400	4.66053900
N	2.43729900	-1.71693100	3.29882300
C	2.77637600	0.74330400	-2.08762000
C	2.67183700	-0.33172300	-2.91396200
H	2.20266300	-0.20185800	-3.88323700
C	3.55751500	0.92055400	-0.81246500
C	3.05729500	2.27148200	-0.27263600
H	4.62392500	0.94541200	-1.04657100
H	3.39893000	0.09637100	-0.11942200
C	2.43071300	2.91759400	-1.51091400
O	2.20687000	1.93683800	-2.45967900
O	2.14733700	4.06493900	-1.71256400
H	3.31837100	-1.18968000	-2.75322700
C	1.98698400	2.10229900	0.83332600
H	1.77935900	3.08884700	1.24187000
H	2.45249100	1.51578600	1.62316200
C	4.15802900	3.22486500	0.21179300
O	4.04872000	3.94947200	1.17815600
O	5.20468300	3.16978100	-0.61746200
C	6.37608600	4.05786700	-0.45432100
C	0.69305600	1.45555900	0.40167200
C	0.42446300	0.10599000	0.70134600
C	-0.29741400	2.21071900	-0.23643500
C	-0.83085000	-0.45389700	0.39764800
H	1.16760900	-0.48970500	1.22136800
C	-1.53823700	1.65054500	-0.54768900
H	-0.10884400	3.25062400	-0.47564700
C	-1.81479000	0.32318300	-0.22571400
H	-1.05244300	-1.46859400	0.70116500
H	-2.29561000	2.26805100	-1.01310300
H	-2.78616900	-0.10506800	-0.43931700
C	5.92810700	5.51374000	-0.58255800
H	5.35438100	5.65148200	-1.50360400
H	5.31217600	5.81355800	0.26509100
H	6.80895700	6.16128800	-0.62515000
C	7.26416900	3.64707400	-1.62635700
H	6.74929800	3.81529700	-2.57618500
H	8.18504700	4.23658700	-1.62050200
H	7.52812100	2.58830300	-1.55659700
C	7.05703100	3.76405800	0.88206100
H	6.42157100	4.05387000	1.71862100
H	7.29268200	2.69868300	0.96053300
H	7.99451100	4.32489500	0.94231600
S	4.19823800	-2.57801200	-0.37287700
O	5.37996600	-1.75533000	-0.05134300
O	2.91290900	-2.03909800	0.17126100
O	4.10463200	-3.05693700	-1.77196800
C	4.41711300	-4.12772100	0.62321600
F	5.46638000	-4.83317200	0.18747800
F	3.31647900	-4.90299000	0.51793500
F	4.59542100	-3.83379400	1.91666400

1b-TS1$_{5syn}$⊂AuCav

C	-5.36508400	0.70867900	2.88807500
C	-4.06022200	0.05747200	3.33959400
C	-5.30840100	1.10213100	1.41574800
H	-5.44893400	1.63989100	3.44911800
C	-3.85123900	-1.32342800	3.29730700
C	-3.02416500	0.84244800	3.86284500
C	-5.60062900	0.18991500	0.39623200
C	-4.96885800	2.40520900	1.02910700
C	-2.69399300	-1.93488600	3.78981900
H	-4.63503100	-1.95249700	2.89251300
C	-1.84711800	0.28697600	4.35669600
O	-3.26333800	2.21094900	3.99864000
C	-5.57163500	0.52582500	-0.95974600
H	-5.86558400	-0.82406600	0.67277400
C	-4.91240900	2.78677900	-0.30889900
O	-4.72562500	3.36278300	2.02343700
C	-2.49954900	-3.44826100	3.75340400
C	-1.70378800	-1.10003200	4.32879600
H	-1.07869500	0.91089000	4.79476100
C	-2.63140100	3.10635100	3.19101100
C	-5.90151500	-0.48290100	-2.05927500
C	-5.20188700	1.83775100	-1.28459800
H	-4.63328500	3.79481500	-0.58598100
C	-3.42598200	3.76035100	2.18151000
H	-1.78550100	-3.68645300	4.54200200
C	-1.84255900	-3.84716800	2.43602800
O	-0.60641900	-1.68952100	4.95266800
C	-4.64868200	-1.27713300	-2.40692700
H	-6.15943000	0.09747900	-2.94532000
O	-5.17580000	2.19414200	-2.63360000
C	-2.57622200	-4.02672400	1.26067600
C	-0.45038900	-3.98629600	2.35448000
C	0.62670500	-1.57888100	4.36161300
C	-0.83647300	4.37185600	2.59107600
C	-4.27400300	-2.42333100	-1.69839000
C	-3.79320500	-0.85269000	-3.43252700
C	-3.95038700	2.39560800	-3.21112800
C	-1.63574300	5.05308700	1.62305600
C	-1.97428000	-4.30636900	0.03003600
H	-3.65381400	-3.91349900	1.29734200
C	0.19240600	-4.27856800	1.15639300
O	0.29863700	-3.80187900	3.51050300
C	1.08838400	-2.67847300	3.56342900
C	-3.07645900	-3.10934700	-1.92394800
H	-4.95135000	-2.80623100	-0.94461600
C	-2.57498300	-1.48292100	-3.66814800
O	-4.18275000	0.22342700	-4.23448700
C	-3.45622200	1.38241500	-4.10240900
C	-2.76993200	-4.45066100	-1.25854400
C	-0.58116500	-4.41653300	0.01057100
H	1.27249500	-4.33422800	1.11164800
C	2.58338800	-0.48488200	3.97015300
C	-2.19627900	-2.55836700	-2.86807500
H	-1.92484700	-1.12237700	-4.45492700
C	-2.08491100	3.64064100	-3.59969900
H	-2.12395800	-4.98723500	-1.95825700
O	0.08390500	-4.54561300	-1.21966100
C	3.00664900	-1.54344100	3.10477300
O	-0.93819400	-3.10586600	-3.09997400
C	-1.63279700	2.67233000	-4.54752900
C	-1.04576200	6.04617100	0.80676100
C	0.53498800	4.69696700	2.71266100
C	4.23805300	-1.44218600	2.42368900
C	3.44425900	0.61463800	4.19024700
C	-0.40116000	2.87088600	-5.21196600
C	-1.27646100	4.76303000	-3.31062600
C	-4.02974100	-5.30997000	-1.06597700
H	-4.55495700	-5.43170200	-2.01700600
H	-4.73418500	-4.87728200	-0.35072000
C	-3.78812000	-4.23282800	4.03736400
H	-3.57289700	-5.30439100	4.06717400
H	-4.56233700	-4.07588700	3.28093600
C	-6.60081600	-0.14092200	3.22099800
H	-6.63023000	-0.35818000	4.29219100
H	-6.62059200	-1.09668200	2.68998000
C	-7.10694700	-1.36791000	-1.71307000
H	-7.98598800	-0.74568700	-1.52508900
H	-6.94626300	-1.98386500	-0.82380700
H	-7.32923000	-2.04161000	-2.54517800
H	-7.51014200	0.40240400	2.95044700
H	-4.20548900	-3.93520500	5.00313500
H	-3.74934800	-6.29801800	-0.69163800
C	-0.06957200	4.92274400	-3.95697000
C	0.36557100	3.97809000	-4.91728900
H	1.31779500	4.12516900	-5.41690000
H	0.55764200	5.77887000	-3.72953100
H	-0.07994300	2.12375300	-5.92957900
H	-1.63087300	5.46835300	-2.56717200
C	1.09650200	5.64482200	1.88031200
H	1.11773600	4.17293200	3.46312400
C	0.30019800	6.32479400	0.92716300
H	-1.67510400	6.54573900	0.07826000
C	4.65865400	0.67204800	3.53498100
H	3.11636400	1.39368500	4.87069300
C	5.04548100	-0.34538600	2.63087700
H	4.52456400	-2.22943400	1.74055400
H	5.96877600	-0.26765800	2.07339600
H	5.31757300	1.51975100	3.69580100
H	0.76088400	7.06721800	0.28379800
H	2.15813200	5.86117000	1.93822500
P	0.31131200	-3.10297700	-1.98799200
N	1.64263800	-3.38020600	-2.90483700
C	1.85003900	-4.63957000	-3.62200200
C	2.32926600	-2.20687300	-3.46222200
H	1.35188400	-5.45342700	-3.09445500
H	2.92310600	-4.84416500	-3.64547300
H	1.46686600	-4.58537800	-4.64884200
H	2.26136800	-1.36890900	-2.76636600
H	1.90523100	-1.91821800	-4.43360900
H	3.38820300	-2.43328300	-3.57765800
Au	0.42716400	-1.39972300	-0.43097900
N	-2.35155100	1.52971900	-4.77211200
N	-3.28281100	3.48210900	-2.96058600
N	-2.94675500	4.70929200	1.43262500
N	-1.37647400	3.39275600	3.38011400
N	1.35549400	-0.52290000	4.57325100
N	2.22518500	-2.65293800	2.93685100
C	1.42954800	0.84208800	1.07256300
C	0.50232600	0.00084000	1.16362800
H	-0.35518900	-0.02648700	1.82490900
C	2.31068100	1.99809100	1.18858400
C	2.96890100	2.45639200	-0.13782000
H	3.10058900	1.76194100	1.90464900
H	1.69723200	2.80976800	1.59607200
C	3.42601400	1.16444000	-0.82141900
O	2.97524200	0.09356100	-0.41637100
O	4.21513200	1.32087000	-1.84395800
H	4.57413900	0.38675100	-2.16015100
C	2.02585700	3.24664400	-1.08909000
H	1.84036400	4.21913900	-0.63033100
H	2.57532700	3.42284000	-2.01781600
C	4.17126200	3.35683800	0.20222800
O	4.14038100	4.56722800	0.09544000
O	5.19499700	2.62296800	0.63155300
C	6.56669000	3.18898300	0.72771800
C	0.74135200	2.51241400	-1.34024200
C	0.66700700	1.48562600	-2.28419500
C	-0.37265500	2.77587200	-0.53870400
C	-0.47172700	0.68794800	-2.35872400
H	1.50981500	1.29280400	-2.94032000
C	-1.50713500	1.97472100	-0.60025400
H	-0.32404300	3.59755600	0.15950900
C	-1.54894800	0.90557100	-1.49264500
H	-0.52436600	-0.09611500	-3.10085000
H	-2.35348300	2.17477600	0.04323800
H	-2.41737900	0.25996300	-1.52867700
S	5.95249200	-1.60000000	-1.52831800
O	6.08597000	-0.81671700	-0.28537300
O	7.15390000	-2.22473800	-2.08207900

O	5.08609300	-0.91051800	-2.56168600
C	4.88656600	-3.04725600	-1.03971300
F	3.66707000	-2.66295500	-0.62527200
F	5.46334500	-3.72754500	-0.03638200
F	4.72447800	-3.88785700	-2.07961500
C	6.60378200	4.27294200	1.80343600
H	6.01643700	5.14342000	1.51220500
H	6.21554700	3.88096400	2.74890500
H	7.64104700	4.58089300	1.96744600
C	6.96605400	3.70392800	-0.65513600
H	6.36666500	4.56685100	-0.94792100
H	8.01885700	3.99997400	-0.64020400
H	6.83850300	2.90996200	-1.39560800
C	7.41272300	1.98155400	1.11913000
H	7.17945000	1.65718400	2.13672100
H	7.23563000	1.14686200	0.43692100
H	8.47137400	2.25462600	1.08853700

1b-Int$_{5syn}$⊂AuCav

C	-3.52175800	-4.69004200	1.66808500
C	-3.05712200	-4.47075300	0.23239100
C	-3.07308200	-3.53466000	2.55773300
H	-2.99744500	-5.57559900	2.02801400
C	-3.80913800	-3.72487300	-0.67911800
C	-1.84607100	-5.00846200	-0.22482400
C	-3.83891100	-2.37915400	2.73885500
C	-1.85076900	-3.60698700	3.23769900
C	-3.41211000	-3.51713600	-2.00195000
H	-4.74775200	-3.29733300	-0.34655900
C	-1.39751900	-4.81588700	-1.52909600
O	-1.11098300	-5.82116900	0.63737500
C	-3.44066400	-1.33451700	3.57768800
H	-4.78641800	-2.29647500	2.21858600
C	-1.40053200	-2.58759700	4.06824000
O	-1.12266600	-4.79352300	3.14011300
C	-4.25747000	-2.73081900	-2.99644600
C	-2.18609400	-4.06591600	-2.39582000
H	-0.45918200	-5.24231700	-1.85931300
C	0.07170200	-5.34714400	1.13796400
C	-4.27632700	-0.07507200	3.78887600
C	-2.20904400	-1.46785400	4.22845300
H	-0.45351000	-2.66764400	4.58516200
C	0.06414900	-4.79697200	2.46703500
H	-3.93202900	-3.04024400	-3.98971300
C	-3.95916300	-1.24066900	-2.89131900
O	-1.80186600	-3.90425000	-3.72794500
C	-3.94705000	0.94744000	2.70825600
H	-3.94971900	0.35787700	4.73439800
O	-1.84035300	-0.45962100	5.11781300
C	-4.61564500	-0.42160300	-1.96945200
C	-3.01002700	-0.63618800	-3.72708100
C	-0.76218800	-3.06633100	-4.01166700
C	2.32424900	-5.04066400	1.03089300
C	-4.60146100	0.93837400	1.47434900
C	-2.95991000	1.92403100	2.90188800
C	-0.77696400	0.32904900	4.77755900
C	2.31623500	-4.49669800	2.35153900
C	-4.38373500	0.95219800	-1.87749000
H	-5.34405200	-0.87056100	-1.30466500
C	-2.74321500	0.72930300	-3.66832000
O	-2.38589800	-1.42150700	-4.70067500
C	-1.06987800	-1.76152100	-4.54248000
C	-4.31759900	1.84206300	0.44902900
H	-5.37881300	0.20215100	1.31119000
C	-2.61752800	2.82521400	1.89396800
O	-2.36538600	2.03630600	4.15995300
C	-1.05209500	1.65758700	4.30657500
C	-5.09334900	1.83445900	-0.86154300
C	-3.43691200	1.50780100	-2.74385400
H	-2.01481000	1.17383800	-4.33457400
C	1.46148700	-2.64314200	-4.26281900
C	-3.28555600	2.76498300	0.67159800
H	-1.85081800	3.56827500	2.06872600
C	1.45512500	0.72420400	4.59977800
H	-5.05281100	2.85383900	-1.25225700
O	-3.20798300	2.88450900	-2.71316500
C	1.15846600	-1.36204400	-4.81528700
O	-2.98656900	3.73377100	-0.28511500
C	1.18619500	2.06773600	4.19220300
C	3.53381600	-4.08884000	2.94386200
C	3.54849100	-5.14831600	0.33032800
C	2.21312900	-0.51600000	-5.22954300
C	2.80998500	-3.05485300	-4.15035400
C	2.26370000	2.92490300	3.87571900
C	2.79033800	0.25940400	4.63807400
C	-6.57623600	1.47799700	-0.68796000
H	-7.04672800	2.16041900	0.02499300
H	-6.73313900	0.46017000	-0.32163000
C	-5.75611000	-3.04749200	-2.88181400
H	-6.31473800	-2.50058600	-3.64638100
H	-6.17867700	-2.77857300	-1.90978600
C	-5.02780400	-4.97200100	1.76902800
H	-5.28934100	-5.84059600	1.15832500
H	-5.64527600	-4.13500100	1.43101500
C	-5.77888300	-0.36725800	3.90596100
H	-5.96035800	-1.05488400	4.73643600
H	-6.19992100	-0.82453500	3.00606900
H	-6.32972400	0.55864300	4.09374800
H	-5.29972000	-5.18109200	2.80724100
H	-5.92360000	-4.11820600	-3.02690700
H	-7.09359400	1.56321000	-1.64738200
C	3.81838300	1.10426500	4.27728900
C	3.55398900	2.44352800	3.90222400
H	4.37033500	3.08910700	3.60045500
H	4.84126500	0.74240600	4.26869600
H	2.04987900	3.94080100	3.57611600
H	2.96208800	-0.77104500	4.93013400
C	4.72072000	-4.73917200	0.93041500
H	3.52677000	-5.55875100	-0.67372000
C	4.71376200	-4.21013200	2.24283400
H	3.49841900	-3.67769900	3.94695000
C	3.81908400	-2.21761900	-4.57760600
H	3.01076600	-4.03492200	-3.73113500
C	3.51959400	-0.94258700	-5.11439100
H	1.95985700	0.46015200	-5.62748100
H	4.32886000	-0.29388700	-5.43407500
H	4.85479300	-2.53237300	-4.49502500
H	5.64558300	-3.87186600	2.68106700
H	5.65947700	-4.80179500	0.39105100
P	-2.08006700	3.49980200	-1.65601100
N	-1.94439600	5.04347900	-2.20961200
C	-0.64024100	5.66362800	-2.43264300
C	-3.09745600	5.93609700	-2.28883800
H	0.15828200	4.92776100	-2.33452700
H	-0.45823000	6.46150300	-1.70366500
H	-0.60283500	6.09034200	-3.44131700
H	-4.02372700	5.37033200	-2.18797100
H	-3.10450000	6.44023700	-3.26151700
H	-3.05975000	6.69414300	-1.49655700
Au	-0.23514300	2.11464300	-1.70372500
N	-0.10328000	2.50770800	4.04544700
N	0.43313400	-0.12452600	4.91495500
N	1.14746800	-4.38108400	3.05279600
N	1.16244100	-5.45992900	0.43927900
N	0.45999700	-3.48398000	-3.86206100
N	-0.13965500	-0.94089200	-4.93255600
C	2.42096900	0.59131400	-1.52223600
C	1.22963600	0.75563500	-2.06919000
H	0.95907000	-0.05448000	-2.73671700
C	3.41440200	-0.53465100	-1.57606000
C	4.01003800	-0.52895700	-0.15198700
H	4.19930200	-0.33215000	-2.30993200
H	2.93528100	-1.47909100	-1.83203000
C	3.89636200	0.95027900	0.22319300
O	2.94764800	1.53692800	-0.57755200
O	4.47455400	1.56210900	1.08089100
H	2.03360400	2.63583600	0.21768300
C	3.18461100	-1.39183200	0.84586500
H	3.58481500	-1.21881000	1.84797400
H	3.39151500	-2.43068200	0.59280000

C	5.48517200	-0.92241300	-0.09714100
O	5.93918300	-1.79526100	0.61279100
O	6.18583500	-0.14865800	-0.93717600
C	7.66070800	-0.17926600	-0.97497400
C	1.69827400	-1.12454200	0.81087500
C	0.81854200	-1.97232000	0.12481000
C	1.17990100	0.02463300	1.41739000
C	-0.54094600	-1.66689200	0.03155800
H	1.19872100	-2.86861500	-0.35546800
C	-0.16853100	0.34714100	1.30098500
H	1.84372900	0.69024400	1.95541100
C	-1.03851900	-0.49882900	0.60982400
H	-1.20724300	-2.32895300	-0.50673400
H	-0.53242900	1.27039600	1.72683900
H	-2.09101600	-0.25041900	0.52545400
S	2.06694800	4.76287000	0.59988100
O	2.18128100	5.04870100	-0.82548300
O	3.12185800	5.13736700	1.52975900
O	1.56634400	3.27132700	0.84109400
C	0.51477600	5.58434200	1.21874400
F	0.61748200	6.89214900	0.97269300
F	0.36286400	5.38306800	2.52552900
F	-0.56003700	5.10811800	0.57898800
C	8.20543300	0.18444000	0.40679500
H	7.74352400	1.11201500	0.75638700
H	8.00287400	-0.60417000	1.13153500
H	9.28712900	0.33628100	0.34340100
C	7.98596100	0.90837600	-1.99639700
H	9.06900600	0.99015400	-2.12343300
H	7.53899900	0.67146100	-2.96615800
H	7.59837500	1.87467600	-1.66334000
C	8.13424100	-1.55042700	-1.45725300
H	7.88499900	-2.32689500	-0.73423300
H	7.67061500	-1.79491000	-2.41812800
H	9.21928900	-1.53246500	-1.59805000

1b-TS2$_{5syn}$⊂AuCav

C	-5.29121100	0.58868500	3.00062200
C	-3.88132100	0.38775700	3.54780400
C	-5.28048800	0.56271200	1.47448700
H	-5.59096000	1.59697600	3.28759200
C	-3.34208300	-0.88532600	3.75697400
C	-3.06321400	1.48579400	3.84737800
C	-5.43309600	-0.61685700	0.74005800
C	-5.12032900	1.75044700	0.74940600
C	-2.05205000	-1.09007900	4.25296600
H	-3.95288200	-1.75056200	3.52673300
C	-1.76631200	1.33475100	4.33036000
O	-3.59688200	2.76836200	3.71489500
C	-5.45109000	-0.64248500	-0.65764600
H	-5.56128400	-1.54938500	1.27739200
C	-5.10274200	1.77589000	-0.64024700
O	-5.07543900	2.94975100	1.46240800
C	-1.46819500	-2.47601600	4.49309200
C	-1.28018700	0.04566200	4.51978400
H	-1.15541400	2.19897200	4.55650900
C	-3.12252000	3.56027200	2.70597400
C	-5.65521100	-1.92902900	-1.45335600
C	-5.27148300	0.57641800	-1.32277700
H	-4.97590500	2.70667500	-1.17690100
C	-3.90855100	3.65473600	1.50521900
H	-0.64474500	-2.34731200	5.19538900
C	-0.85736500	-3.02610200	3.21023000
O	-0.01211400	-0.15344000	5.06230200
C	-4.31762500	-2.62180200	-1.68288000
H	-6.01965500	-1.62839100	-2.43587400
O	-5.34222900	0.57629500	-2.71786800
C	-1.63270600	-3.73017800	2.28624600
C	0.50634000	-2.86458200	2.92073400
C	1.08185500	0.13093700	4.29798400
C	-1.63852000	5.05546600	1.84517200
C	-3.76483400	-3.49167800	-0.73852400
C	-3.57496300	-2.38828700	-2.84713900
C	-4.18038600	0.79288100	-3.40405400
C	-2.42539500	5.15726000	0.65730700
C	-1.09947300	-4.30418000	1.13228900
H	-2.68998400	-3.85439100	2.48706600
C	1.08616200	-3.41936100	1.77921600
O	1.31447600	-2.23667500	3.87702900
C	1.79158000	-0.96908200	3.68936100
C	-2.52499900	-4.11420300	-0.90387000
H	-4.32683500	-3.69448900	0.16509100
C	-2.32826200	-2.97419300	-3.04943700
O	-4.12198100	-1.57427700	-3.84196400
C	-3.54642300	-0.34095400	-4.01697200
C	-1.95647900	-5.07194400	0.13531300
C	0.27070000	-4.13709800	0.90193500
H	2.14740500	-3.32032800	1.58086100
C	2.66140000	1.58427400	3.53231400
C	-1.80534900	-3.81305700	-2.06895500
H	-1.77565700	-2.77707200	-3.95954300
C	-2.53324000	2.14345000	-4.20226700
H	-1.28254300	-5.74578200	-0.39860300
O	0.84133900	-4.75221100	-0.21539300
C	3.39320700	0.49526400	2.97018000
O	-0.58443700	-4.42308900	-2.33248900
C	-1.91203400	1.02013000	-4.82986300
C	-2.01571900	6.03220200	-0.37548600
C	-0.45264800	5.81765500	1.96283900
C	4.61652900	0.74847800	2.30516200
C	3.16573800	2.90129000	3.42152000
C	-0.67951100	1.19115400	-5.50192600
C	-1.90473400	3.40929400	-4.25934700
C	-3.02797100	-5.94298900	0.80526700
H	-3.58371100	-6.50375200	0.04886900
H	-3.75281100	-5.36384100	1.38336600
C	-2.47661400	-3.43914900	5.13727700
H	-2.00470500	-4.40754000	5.32449500
H	-3.35898700	-3.61666000	4.51624800
C	-6.30856500	-0.38700500	3.60930300
H	-6.32027400	-0.28550500	4.69796200
H	-6.09013200	-1.43378600	3.38008000
C	-6.70959600	-2.85508800	-0.83020300
H	-7.66299100	-2.32766700	-0.73927500
H	-6.43482200	-3.20892000	0.16746200
H	-6.85715500	-3.73519300	-1.46221000
H	-7.31004500	-0.16968600	3.22808400
H	-2.82428800	-3.03095700	6.09025100
H	-2.55576500	-6.65267700	1.48974900
C	-0.69126700	3.54376400	-4.89979800
C	-0.07687500	2.43107200	-5.52436500
H	0.89032300	2.55659800	-5.99682700
H	-0.18459400	4.50278500	-4.91131300
H	-0.22016700	0.32152500	-5.95920500
H	-2.38937700	4.24085700	-3.75916400
C	-0.07576100	6.66098200	0.93933000
H	0.13010000	5.71648000	2.87232400
C	-0.86091200	6.77039000	-0.23306200
H	-2.63061100	6.08985800	-1.26713000
C	4.36595000	3.12138300	2.77867800
H	2.58885500	3.70727800	3.86233700
C	5.09078300	2.04169100	2.21906500
H	5.15584200	-0.08304900	1.87283100
H	6.03095300	2.23417700	1.71180200
H	4.76191700	4.12936900	2.70004600
H	-0.52822000	7.42152600	-1.03303700
H	0.83803900	7.23934500	1.02060000
P	0.86618400	-3.95934400	-1.67214100
N	1.87857900	-4.85926800	-2.60145800
C	3.15792100	-4.32618500	-3.07592500
C	1.74901600	-6.31536400	-2.65596600
H	3.15989400	-3.23728800	-3.00998300
H	3.29088800	-4.61024400	-4.12545800
H	3.99542200	-4.70140600	-2.48306800
H	0.73939300	-6.61675400	-2.37554000
H	2.46796900	-6.79672800	-1.98214200
H	1.93715200	-6.65519800	-3.67971600
Au	1.46150300	-1.80206500	-1.18538900
N	-2.45809700	-0.22995300	-4.72032300
N	-3.69528200	1.99536400	-3.49968900

N	-3.57128900	4.42381500	0.51272100
N	-2.01950600	4.22954700	2.86885500
N	1.48907800	1.36173200	4.20149200
N	2.91173100	-0.78583600	3.05512000
C	2.41154800	1.07353000	-0.75100400
C	2.40253100	-0.17518800	-0.24380200
H	2.27579200	-0.17800600	0.83295000
C	2.38177200	2.39847000	-0.04834200
C	1.81336500	3.34380200	-1.11490800
H	3.40786800	2.67505600	0.20335400
H	1.80115200	2.36132600	0.87102800
C	2.20937300	2.61507000	-2.40790900
O	2.51153000	1.30315700	-2.10804300
O	2.24452200	3.03989900	-3.52872800
H	3.59988700	-0.84047500	-0.48140400
C	0.26535000	3.47042000	-1.01207200
H	-0.10260800	3.94398000	-1.92457800
H	0.06906300	4.15809200	-0.19093700
C	2.45834400	4.73074200	-1.15633600
O	1.84759300	5.75659700	-1.36578100
O	3.77835200	4.62598500	-0.95672200
C	4.66690200	5.80461600	-0.97474400
C	-0.42441100	2.15034200	-0.76353700
C	-0.83417400	1.77807400	0.52510400
C	-0.60995800	1.24167700	-1.80983000
C	-1.41066700	0.52855500	0.76172700
H	-0.69392700	2.46595700	1.35318600
C	-1.17468600	-0.00966500	-1.57660300
H	-0.27837500	1.50051400	-2.80735000
C	-1.58116000	-0.37253800	-0.28957900
H	-1.71950000	0.25621700	1.76268900
H	-1.28964200	-0.70394300	-2.39659000
H	-2.02243900	-1.34628400	-0.11000000
S	5.02842300	-2.76787800	-0.36467000
O	5.90436000	-3.27080500	-1.42142800
O	3.80395300	-3.52314800	-0.05312000
O	4.77850600	-1.25208100	-0.48921600
C	6.03151700	-2.83158700	1.19875300
F	6.52893100	-4.05992200	1.35927200
F	5.26106000	-2.53194100	2.25105500
F	7.04011800	-1.95175500	1.14241800
C	4.62802900	6.44892400	-2.35984400
H	4.84892300	5.70279500	-3.12821100
H	3.64997100	6.88505400	-2.56283100
H	5.38546200	7.23656400	-2.41606700
C	6.03399100	5.18728600	-0.68785000
H	6.80136600	5.96596400	-0.67278900
H	6.03081100	4.68263700	0.28271000
H	6.29396600	4.45504500	-1.45669300
C	4.25472900	6.76890900	0.13836600
H	3.28345000	7.21720600	-0.07015900
H	4.20554800	6.24101100	1.09597500
H	4.99994200	7.56512300	0.22515100

(TfO•2b')⊂AuCav

C	6.01019100	0.91740400	1.31033100
C	5.19924200	1.91047600	0.48256400
C	5.22919300	-0.34777500	1.65883800
H	6.20919100	1.41390900	2.26098500
C	5.10013000	1.81997600	-0.91050400
C	4.53566200	2.98032100	1.09826100
C	5.20515600	-1.47608700	0.83023100
C	4.53661300	-0.43754300	2.87461000
C	4.39371200	2.74762400	-1.68223300
H	5.58184400	0.98710400	-1.41069600
C	3.84690200	3.94346400	0.37295000
O	4.55538200	3.08020600	2.49458700
C	4.55884300	-2.66655700	1.18366800
H	5.71795600	-1.42779600	-0.12365300
C	3.90568100	-1.60443400	3.28402300
O	4.51480100	0.67508500	3.72006600
C	4.20449900	2.59713200	-3.18802000
C	3.78746700	3.81215400	-1.00746500
H	3.34205500	4.75795400	0.87381200
C	3.37964200	2.68621500	3.08163900
C	4.49124800	-3.88105900	0.26053600
C	3.93660500	-2.70579800	2.43729100
H	3.38953800	-1.65132600	4.23258000
C	3.35125000	1.39790400	3.71352700
H	3.91905200	3.57839100	-3.56814700
C	3.01959700	1.67044400	-3.43737100
O	3.11592900	4.77261900	-1.76195800
C	3.28646900	-3.76337800	-0.67111000
H	4.30317200	-4.74413300	0.89964600
O	3.34384900	-3.90229400	2.84603000
C	3.18611500	0.28789600	-3.55753000
C	1.71212000	2.17301900	-3.53170200
C	1.76318800	4.86347500	-1.58261300
C	1.17282300	2.97196100	3.56601600
C	3.35527800	-3.04843300	-1.87048000
C	2.05227800	-4.34352200	-0.33856700
C	1.97794000	-3.87839500	2.92760900
C	1.14351000	1.68835800	4.19351300
C	2.12696600	-0.59768800	-3.77144200
H	4.19106600	-0.11300600	-3.51201800
C	0.62125300	1.32406300	-3.69856000
O	1.52225800	3.55288600	-3.58220100
C	0.91714200	4.22836400	-2.55357400
C	2.26481300	-2.89721200	-2.73080700
H	4.29423600	-2.57869000	-2.13890400
C	0.93366400	-4.21651300	-1.15982300
O	1.95343300	-5.07613300	0.84417800
C	1.23789300	-4.49510800	1.86316000
C	2.34987100	-2.08861200	-4.02040100
C	0.83894000	-0.04627900	-3.80795700
H	-0.37908600	1.72478200	-3.79193400
C	-0.07120000	5.68709300	-0.51726300
C	1.06125000	-3.49822800	-2.34475700
H	-0.02222400	-4.63104600	-0.86265900
C	0.02861800	-3.26969300	3.93410800
H	1.52067900	-2.41974500	-4.65000800
O	-0.26392900	-0.85636400	-4.07315600
C	-0.91370600	5.11818100	-1.52090100
O	-0.07756100	-3.32883200	-3.14734100
C	-0.71050100	-3.89303500	2.87995600
C	-0.06873800	1.21103700	4.74261300
C	-0.01155500	3.73990600	3.48840800
C	-2.30724600	5.35429700	-1.46801000
C	-0.64195600	6.43157800	0.54010900
C	-2.11952800	-3.82759300	2.88283300
C	-0.66258800	-2.59411700	4.96550000
C	3.64113300	-2.37481400	-4.80235900
H	3.70415000	-3.44090200	-5.03574900
H	4.54448400	-2.10734400	-4.24800500
C	5.48529000	2.16844200	-3.91599200
H	5.30090500	2.08373100	-4.99061000
H	5.87526400	1.20696400	-3.57018100
C	7.36804000	0.60171100	0.66244400
H	7.93186100	1.52555900	0.50741100
H	7.27199300	0.10916500	-0.30927200
C	5.80385100	-4.13620900	-0.49394700
H	6.62312000	-4.27164500	0.21728900
H	6.08142300	-3.31552800	-1.16147400
H	5.71841400	-5.04056300	-1.10261500
H	7.95040700	-0.05852400	1.31098800
H	6.26917700	2.91343200	-3.75558400
H	3.64781500	-1.81069700	-5.73876100
C	-2.04121500	-2.52706600	4.93279700
C	-2.76967900	-3.14153100	3.88695500
H	-3.84965500	-3.05541100	3.85176400
H	-2.57505000	-1.99627500	5.71521100
H	-2.65848900	-4.29157900	2.07190200
H	-0.07798700	-2.14119300	5.75917500
C	-1.18039800	3.24986500	4.03054900
H	0.02642100	4.69138700	2.97044700
C	-1.20848100	1.98518600	4.66612900
H	-0.07201400	0.22898500	5.20144100
C	-2.00515100	6.63095200	0.57753300
H	0.02497500	6.84749000	1.28781000
C	-2.83559600	6.10277400	-0.43862000

H	-2.93657800	4.92633100	-2.23724400
H	-3.90423100	6.28849300	-0.40269100
H	-2.44686200	7.21029000	1.38251600
H	-2.14818400	1.61650200	5.06059400
H	-2.09573800	3.82628100	3.95563400
P	-0.87457400	-1.90438900	-2.95405900
N	-2.31471700	-2.24260700	-3.65413100
C	-2.90291200	-3.58648600	-3.68421800
C	-3.23553700	-1.15189200	-4.00124800
H	-2.27536900	-4.29334300	-3.14760800
H	-3.00158100	-3.91066400	-4.72710700
H	-3.88096400	-3.55692400	-3.20562700
H	-2.73336600	-0.18700500	-3.94513200
H	-4.08375200	-1.15569400	-3.31407300
H	-3.58144600	-1.29641500	-5.03059400
Au	-0.36756200	-1.21441700	-0.78546100
N	-0.06019100	-4.51122200	1.84369200
N	1.39619200	-3.29037800	3.93073100
N	2.26945500	0.91157300	4.24750300
N	2.32806700	3.44703400	3.01005700
N	1.28620400	5.54871100	-0.58636200
N	-0.37716800	4.36262100	-2.53130700
C	-0.62963200	0.12198900	1.40869300
C	0.42230000	-0.75351200	1.29041300
H	0.32540300	-1.71673600	1.77467300
C	-2.02263400	-0.09750000	1.92205800
C	-2.80775800	1.00409600	1.18784800
H	-2.02321600	0.09131400	2.99921600
H	-2.41136300	-1.10059700	1.74218700
C	-1.72597200	2.05685100	0.94853900
O	-0.47056500	1.42332600	1.04526400
O	-1.79896500	3.22451800	0.72270500
C	-3.37499400	0.40177500	-0.14001400
H	-2.62999400	-0.32397800	-0.48213100
H	-4.23795900	-0.19513700	0.14918700
C	-3.89709600	1.65657700	2.03919600
O	-3.98757600	1.52220700	3.24342500
O	-4.66665600	2.40648400	1.25887700
C	-5.64732000	3.36682100	1.79973400
H	1.42181000	-0.36794400	1.11316100
C	-3.71637500	1.27120400	-1.31725100
C	-5.03967700	1.32833100	-1.77004400
C	-2.72178900	1.90078100	-2.07369700
C	-5.36421500	1.99822000	-2.94854500
H	-5.81007100	0.81465000	-1.20411100
C	-3.04046900	2.57228500	-3.25526200
H	-1.68464700	1.86199800	-1.75507300
C	-4.36359000	2.62154100	-3.69727100
H	-6.39527400	2.02263200	-3.28876800
H	-2.25331800	3.07466800	-3.80723700
H	-4.61167800	3.13867600	-4.61935800
C	-6.18004700	4.02955300	0.53143100
H	-6.66359500	3.29288900	-0.11433100
H	-6.90720300	4.80422300	0.79074500
H	-5.35859400	4.48091300	-0.02961900
C	-4.90888600	4.37606200	2.68041000
H	-5.58470300	5.19545000	2.94272000
H	-4.55756400	3.90891900	3.60153700
H	-4.05359300	4.78712900	2.13624100
C	-6.74812800	2.61666500	2.54667900
H	-6.35523000	2.12911400	3.43890200
H	-7.53134900	3.32147900	2.84255900
H	-7.19722500	1.85951300	1.89749300
S	-3.90353800	-2.95536300	-0.25623200
O	-4.61654200	-2.26020300	-1.34980100
O	-2.45897900	-3.22245900	-0.51585800
O	-4.19315400	-2.44576000	1.10475800
C	-4.64952400	-4.65799300	-0.25727600
F	-5.96628000	-4.59559400	-0.03207700
F	-4.09259300	-5.42453300	0.69883700
F	-4.44813600	-5.26343700	-1.44052800

1b-TS1$_6$⊂AuCav

C	3.37267800	3.96824200	2.99001200
C	3.39004500	4.07129400	1.46844900
C	2.71489400	2.66980000	3.45234600
H	2.72700300	4.77425000	3.34035800
C	4.37868300	3.46316500	0.68862700
C	2.38824200	4.77854000	0.79079500
C	3.44535100	1.50473500	3.69757400
C	1.33244900	2.62053600	3.68778700
C	4.39237600	3.52193700	-0.70966200
H	5.16083500	2.90469700	1.19112500
C	2.36415600	4.87245500	-0.59639700
O	1.39114700	5.41144300	1.52640000
C	2.86097900	0.33758300	4.19745400
H	4.51486500	1.51637700	3.52201300
C	0.70161900	1.47712700	4.16722500
O	0.60772800	3.79633900	3.54029600
C	5.47341500	2.83368600	-1.53764800
C	3.35375700	4.22912700	-1.32861500
H	1.56514100	5.40556900	-1.09412400
C	0.14636400	4.82771800	1.43697600
C	3.66909200	-0.92488200	4.47855000
C	1.47994800	0.35484600	4.42888300
H	-0.36391300	1.47262700	4.35732500
C	-0.25142700	3.93233300	2.48139600
H	5.43199500	3.28260500	-2.53064200
C	5.15035000	1.35491600	-1.72425900
O	3.30420000	4.28470800	-2.72919500
C	3.72682700	-1.77747400	3.21722500
H	3.10467400	-1.49794100	5.21435800
O	0.90852800	-0.76462700	5.03425600
C	5.57921500	0.37093300	-0.82896800
C	4.39530100	0.93194500	-2.82652000
C	2.31135000	3.51232400	-3.27639100
C	-1.84080300	4.48433700	0.38865800
C	4.68126900	-1.55804400	2.22062700
C	2.78528800	-2.79289400	2.99552000
C	0.03797800	-1.53332900	4.31281800
C	-2.23280100	3.57293200	1.42094900
C	5.30649800	-0.98962400	-1.00403900
H	6.16531300	0.67392600	0.02985300
C	4.08580800	-0.40706300	-3.03864300
O	3.99598200	1.88155000	-3.75697500
C	2.66612900	2.21635100	-3.77617200
C	4.74163400	-2.30853000	1.04317400
H	5.40807500	-0.76864600	2.36869500
C	2.79421100	-3.55655100	1.83093400
O	1.85689600	-3.07181300	3.98479100
C	0.54125000	-2.74821800	3.74173400
C	5.79696700	-2.05376200	-0.02818900
C	4.54992400	-1.34296700	-2.12655100
H	3.50979200	-0.70141900	-3.90596300
C	0.13187800	3.09868500	-3.79267200
C	3.77047700	-3.30135900	0.87855100
H	2.05760700	-4.33718600	1.68956900
C	-2.04090800	-2.03228400	3.52649900
H	5.89935900	-2.98066600	-0.59802900
O	4.31735100	-2.69898800	-2.41426200
C	0.48423700	1.79493000	-4.25964100
O	3.79791500	-4.11479800	-0.26976200
C	-1.52889600	-3.22908200	2.92797700
C	-3.50305600	2.95545800	1.35566300
C	-2.72423700	4.75139000	-0.68085800
C	-0.52745400	0.93213600	-4.73959500
C	-1.22167700	3.50313400	-3.80684300
C	-2.40398300	-4.07686700	2.21102100
C	-3.41655900	-1.72701100	3.41113400
C	7.18002000	-1.74201900	0.56099400
H	7.50689300	-2.56692400	1.19923000
H	7.19228000	-0.83334400	1.16785600
C	6.88308400	3.07177800	-0.97640100
H	7.63202900	2.59527900	-1.61534900
H	7.01175900	2.67961000	0.03633500
C	4.76294300	4.18675200	3.60513800
H	5.15112700	5.16578700	3.31127300
H	5.49436900	3.43712100	3.28891400
C	5.05604300	-0.64115200	5.07167800
H	4.95408600	-0.06704700	5.99626400

H	5.70246600	-0.06831600	4.40070100
H	5.56797600	-1.58021500	5.30009000
H	4.70271900	4.14891600	4.69623900
H	7.08981900	4.14438600	-0.93830200
H	7.90903600	-1.61209600	-0.24312200
C	-4.24921300	-2.58453100	2.72584600
C	-3.74171200	-3.75775500	2.11726000
H	-4.41238100	-4.38844700	1.54529000
H	-5.29738800	-2.33641600	2.62728000
H	-1.99606300	-4.98015300	1.76850600
H	-3.79873900	-0.80873100	3.84006600
C	-3.95614500	4.13606200	-0.71617000
H	-2.40449200	5.45696000	-1.44001500
C	-4.34976600	3.23943900	0.30439100
H	-3.79528900	2.27610400	2.14953300
C	-2.18861600	2.64093600	-4.27223400
H	-1.46348100	4.48835000	-3.42734400
C	-1.83953800	1.35408300	-4.74499100
H	-0.23752100	-0.05003700	-5.09852100
H	-2.61971000	0.69129700	-5.10361700
H	-3.23007200	2.93882900	-4.25447600
H	-5.32692100	2.77745900	0.27181500
H	-4.64190100	4.34193100	-1.53198100
P	3.16191900	-3.55652200	-1.66095500
N	3.23033400	-4.95818800	-2.57828500
C	3.00011600	-4.79018500	-4.01993500
C	2.54460200	-6.12962900	-2.01673200
H	3.58821300	-3.95391200	-4.39691600
H	1.93785200	-4.62698000	-4.25668400
H	3.33274900	-5.69986600	-4.52652500
H	2.79179900	-6.23728300	-0.96102100
H	2.89925700	-7.01840400	-2.54509100
H	1.45183700	-6.06926800	-2.13345700
Au	1.04635500	-2.64912800	-1.46454400
N	-0.20531100	-3.56017000	3.05233700
N	-1.20970000	-1.18547500	4.20613800
N	-1.39531800	3.31576400	2.47287000
N	-0.61854300	5.09646800	0.42219800
N	1.08491000	3.94333000	-3.29496600
N	1.78737900	1.37956600	-4.24318400
C	-1.10973200	-2.44465900	-1.70244300
C	-1.24188100	-3.57945300	-1.20932700
H	-1.25208400	-4.54838500	-0.76149200
C	-1.97796700	-1.35504700	-2.22040300
C	-3.11274100	-1.04915300	-1.20607300
H	-2.41018300	-1.71831700	-3.15892600
H	-1.42369800	-0.44254400	-2.42599800
C	-3.94900700	-2.32422300	-0.90887800
O	-3.62068100	-3.43794500	-1.31047700
O	-5.00757800	-2.19074500	-0.14938200
H	-5.23337200	-1.23705900	0.11516000
C	-2.52891600	-0.50744400	0.15492800
H	-3.23297900	0.22560200	0.55134200
H	-2.52407600	-1.33901000	0.85966600
C	-3.97882500	0.01897400	-1.91227400
O	-3.60639400	1.16138800	-2.08067900
O	-5.10433000	-0.52183800	-2.36983900
C	-6.15288900	0.24693100	-3.08139400
C	-1.13163400	0.07473400	0.10659600
C	-0.86023700	1.30295800	-0.51134900
C	-0.06542300	-0.61424700	0.70918500
C	0.43603100	1.82160200	-0.52406200
H	-1.66926600	1.84421100	-0.98716300
C	1.23695900	-0.09919500	0.69236300
H	-0.26226400	-1.55365400	1.21397100
C	1.49091700	1.12556500	0.07120800
H	0.61551400	2.78392500	-0.98602600
H	2.03771500	-0.63773600	1.18771400
H	2.49264900	1.54002900	0.06806300
S	-6.46318400	0.55798500	1.67238300
O	-5.59118800	0.18941000	0.49407400
O	-7.06749100	1.88750300	1.52410500
O	-5.87549500	0.20461500	2.97449300
C	-7.87601700	-0.62683100	1.45287600
F	-8.79480400	-0.46100600	2.40638900
F	-7.43262900	-1.89840700	1.49899800
F	-8.46162300	-0.43854200	0.25577000
C	-5.63019700	0.55912800	-4.48206900
H	-4.79373100	1.25721800	-4.43015200
H	-5.30548100	-0.35680600	-4.98558200
H	-6.42384700	1.01600400	-5.08048800
C	-6.54793500	1.50870100	-2.31641400
H	-7.40792200	1.96026900	-2.82111900
H	-6.82976500	1.26714000	-1.29233100
H	-5.73255500	2.23007600	-2.29387000
C	-7.30773500	-0.75137000	-3.11242100
H	-7.62360300	-0.98664900	-2.09397300
H	-8.15599200	-0.32206300	-3.65303600
H	-7.00603100	-1.67742200	-3.60970100

1b-Int_6⊂AuCav

C	3.46220600	4.19391900	2.67901900
C	3.51956200	4.14031500	1.15595500
C	2.75191500	2.96921500	3.25144700
H	2.83524400	5.05065100	2.92861800
C	4.51207500	3.43105100	0.47129000
C	2.55477800	4.79664700	0.38095100
C	3.43584500	1.80863700	3.62267500
C	1.36486200	2.98806400	3.45692800
C	4.56637900	3.34475800	-0.92445300
H	5.26464200	2.90816600	1.05097000
C	2.57252400	4.75010500	-1.00829500
O	1.55566700	5.53178800	1.01786100
C	2.80027900	0.71345000	4.21450000
H	4.50780500	1.76620400	3.46868100
C	0.68408500	1.91899200	4.02881400
O	0.68250800	4.16673200	3.17410000
C	5.65183200	2.55075800	-1.64719800
C	3.56536500	4.01309000	-1.64183500
H	1.80396000	5.24967500	-1.58260300
C	0.29488000	4.98487500	0.96408200
C	3.55023300	-0.55101800	4.62157100
C	1.41713900	0.80180200	4.41097000
H	-0.38491700	1.96606900	4.18973400
C	-0.15146700	4.21777500	2.09047100
H	5.66289200	2.91465100	-2.67515500
C	5.29009800	1.06997200	-1.72312500
O	3.55941900	3.93220200	-3.04147500
C	3.58372800	-1.51645900	3.44262200
H	2.95460800	-1.02988100	5.39897100
O	0.79572200	-0.23778400	5.10362800
C	5.63106800	0.15688500	-0.72117200
C	4.58741600	0.56945100	-2.82650200
C	2.57424800	3.11918700	-3.54247300
C	-1.69231600	4.60732600	-0.07375200
C	4.56379400	-1.43150800	2.45112200
C	2.60115200	-2.50402200	3.28868600
C	-0.09838300	-1.03937900	4.45617800
C	-2.12647600	3.81685300	1.03590100
C	5.31103700	-1.20264800	-0.79028200
H	6.18005600	0.51544100	0.14119000
C	4.22960900	-0.76988500	-2.93265200
O	4.26353000	1.44801700	-3.85783300
C	2.94006800	1.79249800	-3.94457500
C	4.60778200	-2.28564100	1.34709700
H	5.32982500	-0.67176700	2.54762800
C	2.58319200	-3.35720300	2.18767000
O	1.66140600	-2.67283800	4.30008600
C	0.35758600	-2.33736900	4.03884600
C	5.70899300	-2.19082600	0.29848000
C	4.59349100	-1.63592200	-1.91174300
H	3.68001700	-1.12036500	-3.79577000
C	0.39806800	2.65241200	-4.02499600
C	3.58502500	-3.23531000	1.23230800
H	1.81016300	-4.10971800	2.09537800
C	-2.19411000	-1.54796300	3.72921900
H	5.77486500	-3.17220200	-0.17750900
O	4.30849700	-2.99573800	-2.07412100
C	0.76249800	1.32310900	-4.39895700
O	3.60975100	-4.14938500	0.17255700

C	-1.74185900	-2.84278000	3.32226100
C	-3.40951600	3.22294900	1.00589600
C	-2.55250600	4.78703200	-1.18104000
C	-0.24074900	0.40774000	-4.78590700
C	-0.96240700	3.03138700	-4.03644000
C	-2.65982900	-3.75540400	2.75753900
C	-3.55131100	-1.19763100	3.55019800
C	7.08866400	-1.89728900	0.90477600
H	7.34675400	-2.66839400	1.63543500
H	7.13693100	-0.93216300	1.41540000
C	7.04762200	2.79202900	-1.05306000
H	7.80186200	2.24479700	-1.62550800
H	7.12859100	2.47383400	-0.00989900
C	4.84314900	4.43145200	3.30794300
H	5.27216800	5.36186400	2.92596200
H	5.55583700	3.63024300	3.09191900
C	4.94100600	-0.27070100	5.20704800
H	4.85400500	0.38887500	6.07461300
H	5.61816200	0.21123900	4.49624000
H	5.41049100	-1.20545700	5.52606800
H	4.75397400	4.50894700	4.39502400
H	7.28841500	3.85780800	-1.08867600
H	7.84860200	-1.89219600	0.11885800
C	-4.42634000	-2.11006100	3.00050600
C	-3.98094900	-3.39245900	2.60545400
H	-4.68350600	-4.08113200	2.15038200
H	-5.46195500	-1.83711500	2.85184400
H	-2.29398000	-4.73155100	2.45852700
H	-3.87162400	-0.20265800	3.83594300
C	-3.79777000	4.19727500	-1.18271500
H	-2.20176200	5.40308600	-2.00206900
C	-4.22846200	3.41350900	-0.08646400
H	-3.72545100	2.61947100	1.84908400
C	-1.92375100	2.11659200	-4.40505800
H	-1.21601200	4.03610800	-3.72107700
C	-1.56018000	0.80356600	-4.78642500
H	0.05931900	-0.59736200	-5.06231500
H	-2.33011900	0.09584800	-5.06976700
H	-2.96998100	2.39598500	-4.37244400
H	-5.21354400	2.96447700	-0.10699400
H	-4.46089800	4.33022500	-2.03164800
P	3.05467700	-3.73154600	-1.31634000
N	3.19768800	-5.24214500	-2.05316500
C	3.07013800	-5.24006400	-3.51632600
C	2.43575700	-6.32626100	-1.41944300
H	3.69324500	-4.45446900	-3.94332600
H	2.02969900	-5.10028400	-3.84717700
H	3.42730200	-6.20301500	-3.89222700
H	2.60056000	-6.31678500	-0.34227200
H	2.80279700	-7.27784800	-1.81439100
H	1.35651400	-6.25556500	-1.62307800
Au	0.89448300	-2.89171100	-1.33137500
N	-0.43114500	-3.20769500	3.48220800
N	-1.32913400	-0.65338300	4.29782700
N	-1.31759700	3.64486700	2.12613900
N	-0.45031800	5.18057300	-0.08225300
N	1.34567100	3.54102700	-3.59771400
N	2.06817600	0.92281600	-4.35960700
C	-1.15191400	-2.66462800	-1.32797800
C	-1.87031500	-3.51794000	-0.60150900
H	-1.52922900	-4.37744400	-0.04354100
C	-1.93229400	-1.56213300	-2.00363600
C	-3.08404300	-1.05796200	-1.09013400
H	-2.35662600	-1.94025900	-2.94421800
H	-1.30464000	-0.70949200	-2.25772200
C	-3.89497000	-2.24097100	-0.57518100
O	-3.27970200	-3.40803800	-0.43657300
O	-5.06726000	-2.20316200	-0.19707700
H	-6.00724000	-1.15965400	-0.20461900
C	-2.53648100	-0.33448500	0.20413400
H	-3.24359400	0.45621000	0.45836600
H	-2.57211500	-1.04866700	1.02758200
C	-3.93721700	-0.11347000	-1.95031400
O	-3.67554800	1.06271000	-2.09252600
O	-4.91457400	-0.78149700	-2.57073300
C	-5.86296100	-0.16667800	-3.53287700
C	-1.12986600	0.21564600	0.12618200
C	-0.82534800	1.35276400	-0.63264400
C	-0.09826900	-0.40579400	0.84375500
C	0.47676600	1.85398200	-0.66359700
H	-1.61167800	1.83773700	-1.19850300
C	1.20622200	0.09115000	0.81123400
H	-0.31937900	-1.29430700	1.42418400
C	1.49787900	1.22703800	0.05478600
H	0.68845000	2.74600400	-1.23835200
H	1.98774900	-0.40071400	1.37999000
H	2.50653400	1.62362800	0.03169200
S	-6.75151100	0.63118900	0.88915200
O	-6.81816100	-0.48006100	-0.21589800
O	-7.29234200	1.87542600	0.36111100
O	-5.46660800	0.60747400	1.58769600
C	-8.01876800	-0.01769500	2.08431500
F	-8.02486400	0.75592800	3.16862300
F	-7.70694100	-1.27235100	2.43787800
F	-9.22356000	-0.01137800	1.51970200
C	-5.14296600	-0.04012400	-4.87243500
H	-4.36637500	0.72257300	-4.81966800
H	-4.69451600	-0.99474700	-5.16318900
H	-5.85525900	0.25493200	-5.64848700
C	-6.40779400	1.18135400	-3.05907000
H	-7.20183500	1.48455900	-3.74910800
H	-6.83820700	1.10597000	-2.06186200
H	-5.63502900	1.94794900	-3.05141800
C	-6.97609900	-1.21122800	-3.59380500
H	-7.45763100	-1.30611500	-2.61836100
H	-7.72824100	-0.90992100	-4.32824500
H	-6.57432200	-2.18581500	-3.88463700

1b-TS2$_6$⊂AuCav

C	6.04041800	1.58780500	-1.07387500
C	5.04339200	1.24653100	-2.17891100
C	5.41045300	1.43552400	0.31012400
H	6.26751700	2.64847600	-1.18783600
C	4.82684300	-0.07121700	-2.59761200
C	4.29237100	2.24601700	-2.81369700
C	5.46239200	0.24798300	1.04597900
C	4.76109300	2.52455600	0.90631200
C	3.90266200	-0.41857000	-3.58900800
H	5.38899500	-0.86345400	-2.11586100
C	3.37084100	1.95110900	-3.81194900
O	4.47638500	3.58314800	-2.45073500
C	4.91555000	0.12939200	2.32818900
H	5.96254600	-0.61060500	0.61300700
C	4.18583500	2.45340500	2.16890500
O	4.76477900	3.73489500	0.21916000
C	3.66398300	-1.86514300	-4.00977700
C	3.17525100	0.62443400	-4.17591200
H	2.79062100	2.73953200	-4.27224000
C	3.44637600	4.13630800	-1.72874100
C	4.98402300	-1.16018800	3.13887300
C	4.27500900	1.25350800	2.86156600
H	3.68934500	3.31158500	2.60078200
C	3.57962700	4.17089500	-0.30177400
H	3.16004100	-1.82281200	-4.97586000
C	2.69725600	-2.56251700	-3.05703200
O	2.21937800	0.32020400	-5.15079100
C	3.78657600	-2.04186600	2.80744800
H	4.87708300	-0.87414000	4.18551000
O	3.78907600	1.16998100	4.16830500
C	3.13085000	-3.23885500	-1.91376100
C	1.31913800	-2.55831900	-3.31343800
C	0.92255200	0.45415400	-4.71663300
C	1.39302000	5.09858100	-1.54826800
C	3.79799500	-2.90925700	1.71168100
C	2.61592500	-1.99536600	3.57650000
C	2.43991100	1.19044300	4.36731700
C	1.50757500	5.08472600	-0.12427800
C	2.26145200	-3.92336800	-1.05994100
H	4.19099800	-3.25309900	-1.69205400
C	0.41098400	-3.21382100	-2.48725600

O	0.86712600	-1.92944900	-4.46886300
C	0.21877100	-0.73004900	-4.31692500
C	2.72050000	-3.73275000	1.37951900
H	4.68755200	-2.94804500	1.09490500
C	1.50959700	-2.78569500	3.27632500
O	2.58234300	-1.18446900	4.70809000
C	1.79788500	-0.06094200	4.66636500
C	2.75435500	-4.66727800	0.17599600
C	0.89802700	-3.89213000	-1.37778800
H	-0.64614500	-3.19835800	-2.71475400
C	-0.88314800	1.71216700	-4.13577200
C	1.58089200	-3.64532300	2.18792100
H	0.62271300	-2.73892200	3.89556200
C	0.45194200	2.26638900	4.63016800
H	2.03389700	-5.46454400	0.37360200
O	-0.00271000	-4.65522800	-0.62187900
C	-1.57856600	0.53369600	-3.72395500
O	0.50238500	-4.50144900	1.94780100
C	-0.18437200	1.02454100	4.94065200
C	0.43898000	5.56310700	0.66720200
C	0.21712600	5.60953100	-2.14398100
C	-2.87495200	0.64621400	-3.17639300
C	-1.49302000	2.97470800	-3.97234200
C	-1.56263100	1.00879900	5.25446600
C	-0.30782300	3.45865500	4.63803800
C	4.11909600	-5.34068400	-0.02339000
H	4.39130500	-5.90428900	0.87287900
H	4.92492500	-4.62922500	-0.22098800
C	4.97507100	-2.64210300	-4.20219100
H	4.76526100	-3.66019400	-4.54183400
H	5.56806300	-2.71393900	-3.28599600
C	7.35923700	0.81371400	-1.21558700
H	7.80618600	1.01180800	-2.19353800
H	7.23174400	-0.26906600	-1.12754000
C	6.32439700	-1.89438900	2.99279800
H	7.14382200	-1.23632300	3.29394900
H	6.52633600	-2.22105200	1.96891200
H	6.33880200	-2.78259500	3.63052200
H	8.06405600	1.12629700	-0.44020200
H	5.59465500	-2.14510000	-4.95336000
H	4.07467100	-6.03101500	-0.86979100
C	-1.65063900	3.41526800	4.94875500
C	-2.27963600	2.18737800	5.26231900
H	-3.34198300	2.17469400	5.47540300
H	-2.24149500	4.32490300	4.94054800
H	-2.02647000	0.05598400	5.48497900
H	0.19896700	4.38608600	4.39400600
C	-0.81016300	6.06992200	-1.34921400
H	0.15973100	5.62267700	-3.22644900
C	-0.69983800	6.04450100	0.06132600
H	0.54822200	5.52684300	1.74569500
C	-2.74800500	3.06083600	-3.40862400
H	-0.93915200	3.85153100	-4.28505300
C	-3.44193000	1.89274800	-3.01526100
H	-3.40654400	-0.24697500	-2.88104300
H	-4.42615700	1.96733100	-2.56707000
H	-3.20719300	4.03132500	-3.26402700
H	-1.53287000	6.38612400	0.66372200
H	-1.72031300	6.44788100	-1.80311600
P	-0.61637200	-4.15247000	0.80402000
N	-1.67203000	-5.42263100	1.09745800
C	-2.72247300	-5.62117700	0.08294300
C	-2.19233200	-5.51525600	2.46837100
H	-2.29682400	-5.54206000	-0.91589900
H	-3.53909600	-4.89722200	0.18792500
H	-3.12326600	-6.63118100	0.20566600
H	-1.37886000	-5.42109400	3.18722000
H	-2.64845200	-6.50128600	2.59106200
H	-2.95806700	-4.75243100	2.67508100
Au	-1.62185100	-2.09763400	0.90326700
N	0.52869600	-0.14596600	4.93557700
N	1.78768000	2.31438200	4.33934200
N	2.64155200	4.61781700	0.47847400
N	2.39387500	4.59779600	-2.33662700
N	0.38569000	1.63490300	-4.64014800
N	-0.98829100	-0.69570000	-3.83863200
C	-3.17115800	-0.61909600	1.14693000
C	-3.56805000	-0.51758300	2.43773100
H	-3.63365300	-1.35001700	3.13000600
C	-3.24864000	0.63537600	0.30877700
C	-3.05654100	1.90932700	1.15552600
H	-4.22237200	0.65908900	-0.18559600
H	-2.50097000	0.62843600	-0.48453100
C	-3.90054400	1.85367400	2.42598700
O	-4.02214400	0.62135300	3.04415900
O	-4.38989900	2.80164200	2.98270500
H	-3.90652400	-1.67075100	0.61309200
C	-1.58655700	2.10634600	1.66083700
H	-1.44755600	3.17820700	1.81376800
H	-1.49973600	1.63142100	2.63811200
C	-3.56903700	3.14224600	0.39034300
O	-2.90401800	4.12471300	0.13833800
O	-4.85829200	2.96935200	0.07729900
C	-5.74759500	4.10666800	-0.24913600
C	-0.48578800	1.55961800	0.78557700
C	-0.18247200	2.12879100	-0.45714400
C	0.28958200	0.48116900	1.24004400
C	0.87521300	1.63164800	-1.22006000
H	-0.76626700	2.96889100	-0.81818200
C	1.34804600	-0.01852100	0.47606100
H	0.07797100	0.04627000	2.21201700
C	1.64224600	0.55824700	-0.75991600
H	1.10882100	2.09332300	-2.17063000
H	1.94533600	-0.84256500	0.85159300
H	2.46667800	0.18223300	-1.35329400
S	-5.36211600	-2.25870800	-1.20843400
O	-5.31750700	-0.82948900	-1.54425800
O	-6.59644600	-3.01113900	-1.39146700
O	-4.70667400	-2.55145800	0.15780100
C	-4.13034100	-3.03302100	-2.37074100
F	-4.18473200	-4.36837800	-2.31470800
F	-2.87706400	-2.64741900	-2.04232800
F	-4.37393500	-2.63901300	-3.62078500
C	-7.11542600	3.43332300	-0.32625200
H	-7.37640900	2.98742200	0.63693100
H	-7.88000500	4.16923200	-0.59073300
H	-7.11251100	2.64343900	-1.08233500
C	-5.69281900	5.12399800	0.89103500
H	-5.86042500	4.62760900	1.84900600
H	-4.72575500	5.62773600	0.92442100
H	-6.47283200	5.87591400	0.73883100
C	-5.35971100	4.72854300	-1.58901800
H	-4.34670300	5.12883200	-1.54789000
H	-5.42924100	3.99324200	-2.39434700
H	-6.05288700	5.54349400	-1.81926200

(TfO•3b)⊂AuCav

C	5.45801400	-2.20426100	1.77830300
C	4.48104800	-1.47690700	2.69953900
C	4.95276800	-2.25860000	0.33731000
H	5.48617600	-3.23779500	2.12558500
C	4.47684400	-0.08258900	2.81681000
C	3.54583000	-2.17997000	3.47298800
C	5.25144900	-1.28040600	-0.61719900
C	4.17531900	-3.34403700	-0.08752800
C	3.60575600	0.61814200	3.65904100
H	5.17793900	0.48595900	2.21634900
C	2.66663200	-1.53024800	4.33190100
O	3.48828200	-3.57508900	3.38681400
C	4.82152700	-1.36321400	-1.94731500
H	5.85491700	-0.43073600	-0.31944800
C	3.71887100	-3.47092300	-1.39326000
O	3.90081700	-4.34920300	0.83566600
C	3.62360500	2.14250000	3.74018400
C	2.70063500	-0.14188100	4.41361900
H	1.94662700	-2.09497000	4.90911100
C	2.41005100	-4.04968200	2.67899500
C	5.14784900	-0.29765200	-2.98923400
C	4.05045600	-2.47652000	-2.30395800
H	3.11803700	-4.31916700	-1.68992000

C	2.61894400	-4.41055000	1.30832200
H	3.09962700	2.40593300	4.65943400
C	2.81435900	2.74750500	2.59613700
O	1.80782200	0.50093700	5.27609700
C	4.08512200	0.79559900	-2.96698100
H	5.07057500	-0.78377500	-3.96226100
O	3.65797700	-2.60602400	-3.63834500
C	3.37722100	3.08383700	1.36236600
C	1.44175800	2.97965700	2.75993600
C	0.50373900	0.49791300	4.83561800
C	0.19939700	-4.54649000	2.46081700
C	4.15011500	1.86666700	-2.06889200
C	2.98399700	0.75420300	-3.83334900
C	2.33099600	-2.46163000	-3.92367000
C	0.40047800	-4.85053600	1.07956200
C	2.63111400	3.63814500	0.31629100
H	4.43874300	2.92545100	1.21455700
C	0.64916600	3.50570000	1.74605600
O	0.89358000	2.70787300	4.00910400
C	0.03894500	1.64361600	4.10863500
C	3.18343500	2.87345300	-2.01029300
H	4.98816900	1.91438700	-1.38351000
C	1.99656400	1.73268800	-3.80983100
O	2.88568200	-0.28222400	-4.76496900
C	1.92020400	-1.22638300	-4.53537100
C	3.26312500	4.02757100	-1.01634400
C	1.26113400	3.82602400	0.54056500
H	-0.41237500	3.65258200	1.88355200
C	-1.50561400	-0.51951800	4.49415100
C	2.10425400	2.77764400	-2.89816300
H	1.15337700	1.67253100	-4.48576500
C	0.18809400	-3.22594700	-4.03085400
H	2.64772900	4.83126800	-1.42777900
O	0.46694600	4.42175800	-0.45067000
C	-1.94389900	0.59947900	3.72011600
O	1.08498300	3.72159000	-2.86774800
C	-0.21947400	-2.00606400	-4.65598400
C	-0.70408800	-5.21711800	0.27663400
C	-1.10086400	-4.63544900	3.00905400
C	-3.20424700	0.56735700	3.08385700
C	-2.34917700	-1.64616700	4.62482400
C	-1.57315300	-1.83404600	-5.02655200
C	-0.76975900	-4.23653700	-3.78428000
C	4.68730500	4.58231000	-0.86919200
H	5.06062300	4.91426100	-1.84163300
H	5.39530200	3.84562300	-0.48039100
C	5.04778100	2.70854400	3.83648700
H	5.01410700	3.79700100	3.93594500
H	5.66180600	2.47546300	2.96181200
C	6.88351500	-1.64105200	1.88346300
H	7.23035300	-1.69309600	2.91891500
H	6.95483000	-0.59667900	1.56663900
C	6.57413200	0.25531900	-2.85793800
H	7.29943200	-0.55936600	-2.93256500
H	6.75043900	0.76610400	-1.90727600
H	6.77323200	0.97315300	-3.65828500
H	7.56531000	-2.22327400	1.25748100
H	5.55576400	2.29478000	4.71171200
H	4.68926600	5.43454300	-0.18456400
C	-2.08370200	-4.04208200	-4.15581600
C	-2.48606500	-2.84005800	-4.78477700
H	-3.52908900	-2.70012000	-5.04168200
H	-2.82637500	-4.80733300	-3.95701500
H	-1.86257400	-0.90072600	-5.49660200
H	-0.43298500	-5.15103700	-3.30805600
C	-2.15945100	-4.99439300	2.20543800
H	-1.22804300	-4.39763500	4.05832500
C	-1.96117200	-5.28436000	0.83391100
H	-0.52449600	-5.42887300	-0.77173700
C	-3.56897000	-1.66467700	3.98012000
H	-2.00004400	-2.47827500	5.22573700
C	-3.99141900	-0.55780600	3.20628200
H	-3.52872000	1.42258800	2.50229400
H	-4.94573300	-0.58867700	2.69428700
H	-4.21121300	-2.53502000	4.06363800
H	-2.81738600	-5.53752400	0.21992300
H	-3.16357600	-5.03238400	2.61177300
P	-0.10494300	3.55066500	-1.70008700
N	-1.31480100	4.48965700	-2.25627400
C	-1.58187000	5.86915700	-1.84254600
C	-2.15441000	3.98007700	-3.33760200
H	-0.98388300	6.12035400	-0.97046000
H	-2.63706300	5.94342500	-1.57375300
H	-1.34234100	6.55842500	-2.66161900
H	-1.89505200	2.94107700	-3.56021800
H	-2.00301000	4.56878900	-4.25037300
H	-3.20024000	4.02481000	-3.02274400
Au	-0.26182000	1.28582800	-1.26034900
N	0.68664300	-1.00232600	-4.88362800
N	1.49311700	-3.42284600	-3.66961200
N	1.64825800	-4.78350200	0.52790300
N	1.24093600	-4.12832400	3.24300200
N	-0.24942800	-0.54163200	5.04273500
N	-1.13784500	1.69246900	3.56596700
C	-3.41701400	0.94421500	-1.85737200
C	-3.68566200	0.50577200	-3.08296000
H	-3.53012700	1.05100100	-4.00431200
C	-3.72401400	0.10662000	-0.65778500
C	-3.69448500	-1.38873400	-1.02846200
H	-4.70384400	0.38778500	-0.25995500
H	-3.01225600	0.31243800	0.14348200
C	-4.40352800	-1.66283000	-2.36597600
O	-4.27769700	-0.72696600	-3.34525200
O	-4.96295700	-2.70154100	-2.62825800
H	-3.04651700	1.94764800	-1.71682700
C	-2.25780800	-1.94614800	-1.28465300
H	-2.31871400	-3.03330900	-1.18782900
H	-2.00223900	-1.73868900	-2.32416200
C	-4.45459400	-2.22422900	0.01205300
O	-3.96850500	-3.13794400	0.64759600
O	-5.72078400	-1.80792600	0.08930100
C	-6.79436600	-2.66570200	0.64505000
C	-1.10530000	-1.46663200	-0.43896200
C	-1.06401500	-1.59151200	0.95075200
C	0.06474700	-1.00521100	-1.11051500
C	0.11074600	-1.29820500	1.65159100
H	-1.92821000	-1.97005900	1.48250200
C	1.25668200	-0.72699800	-0.38856800
H	0.11416700	-1.11220300	-2.19073800
C	1.26324800	-0.85601700	1.00045000
H	0.13596200	-1.44999000	2.72198000
H	2.16140300	-0.45778100	-0.92379500
H	2.16349100	-0.63970500	1.56051400
C	-8.05024700	-1.86281100	0.31683300
H	-8.15342400	-1.73978600	-0.76431600
H	-8.93455300	-2.38209000	0.69682000
H	-8.00250000	-0.87191900	0.77645000
C	-6.78848500	-4.00771500	-0.08799000
H	-6.77849700	-3.84793400	-1.16808000
H	-5.91590800	-4.60312700	0.18396500
H	-7.68945600	-4.56664300	0.18171900
C	-6.62341000	-2.83535400	2.15325100
H	-5.67588200	-3.32329200	2.38051800
H	-6.66730600	-1.87003300	2.66372600
H	-7.43994900	-3.45434400	2.53771300
S	-3.51061900	3.45244600	0.49328000
O	-2.18782500	2.76481900	0.40763500
O	-3.92029300	4.15323700	-0.74242100
O	-4.56747500	2.65351800	1.14971800
C	-3.15155700	4.81409800	1.70108600
F	-2.81297600	4.30357600	2.89379100
F	-4.20849500	5.61751600	1.85933700
F	-2.11403100	5.56153900	1.26503800

(TfO•1b)⊂AuCav-1

C	5.85181400	1.14111800	-1.84312200
C	4.67864300	0.59405000	-2.65273400
C	5.47058500	1.27906400	-0.37073400
H	6.02877100	2.15301600	-2.20923400
C	4.49219900	-0.77692600	-2.85954500

C	3.74437200	1.45688100	-3.24264800
C	5.59278600	0.22797800	0.54497100
C	4.96753400	2.49113900	0.11728200
C	3.44441700	-1.29884200	-3.62463600
H	5.21559500	-1.46456800	-2.43827400
C	2.65620100	0.98591300	-3.97019400
O	3.97862900	2.83075300	-3.17582900
C	5.24119300	0.35167900	1.89356700
H	5.97980900	-0.72227900	0.19464500
C	4.59286700	2.66302600	1.44356500
O	4.88710900	3.57440900	-0.75905200
C	3.31353800	-2.79390200	-3.90320400
C	2.51490600	-0.38681000	-4.14834900
H	1.94854300	1.67406200	-4.41379200
C	3.15609400	3.60552000	-2.41177700
C	5.36818400	-0.80026500	2.88772000
C	4.72826000	1.58652300	2.31085900
H	4.19920400	3.60847200	1.79112800
C	3.63800200	3.99407100	-1.11346900
H	2.75850000	-2.88317600	-4.83760000
C	2.45744600	-3.44772600	-2.82686000
O	1.49752100	-0.87111900	-4.96206300
C	4.10845700	-1.65626500	2.83428800
H	5.39610700	-0.35047000	3.88066400
O	4.38531600	1.73977700	3.65308100
C	2.97088300	-3.79741100	-1.57179300
C	1.08958500	-3.65154000	-3.04561000
C	0.20948100	-0.92402600	-4.49199400
C	1.27750100	4.84321500	-2.07632500
C	3.96308500	-2.69968300	1.91465200
C	3.02833900	-1.40257700	3.69061600
C	3.05156600	1.84889900	3.94200400
C	1.73717300	5.19274500	-0.76876700
C	2.16777600	-4.28698900	-0.53706400
H	4.02929300	-3.65167200	-1.38494000
C	0.25524100	-4.14049100	-2.04815900
O	0.53568100	-3.29875400	-4.27402000
C	-0.32009000	-2.22132600	-4.19683400
C	2.79703400	-3.46177000	1.79262300
H	4.80000400	-2.93520400	1.26842700
C	1.83840800	-2.11582200	3.58985100
O	3.15846600	-0.43422900	4.68567600
C	2.41023000	0.70823400	4.53101500
C	2.70653300	-4.65523900	0.84236300
C	0.80054600	-4.42134000	-0.80472700
H	-0.80907400	-4.23274000	-2.22159200
C	-1.81583100	0.00047300	-4.00866300
C	1.72414800	-3.10866600	2.62430500
H	1.01647300	-1.89298400	4.25715400
C	1.09542800	3.00838600	4.06345200
H	1.96834400	-5.33114600	1.28193600
O	-0.08692100	-4.72651400	0.22623800
C	-2.34899600	-1.29922700	-3.73652000
O	0.48752300	-3.75612800	2.52581100
C	0.47228000	1.89463100	4.70795300
C	0.95543500	6.04603000	0.04284100
C	0.06634000	5.38999800	-2.55575100
C	-3.69671300	-1.44056100	-3.33760400
C	-2.66248900	1.12696200	-3.89385500
C	-0.88715500	1.97926000	5.08607100
C	0.33655600	4.16928500	3.78881300
C	4.02862600	-5.43377000	0.75103000
H	4.36624600	-5.72696200	1.74861300
H	4.83095000	-4.85480700	0.28588400
C	4.66635500	-3.49463800	-4.09335300
H	4.50637600	-4.53889000	-4.37493300
H	5.28165800	-3.49124400	-3.18906500
C	7.14728200	0.34262800	-2.04658800
H	7.40427500	0.30550400	-3.10869400
H	7.07767100	-0.68823000	-1.68800500
C	6.65993200	-1.60893400	2.70464700
H	7.52858700	-0.95367200	2.81077400
H	6.73085800	-2.08945800	1.72477400
H	6.72256900	-2.39474600	3.46242800
H	7.96816500	0.82140600	-1.50613200

H	5.23932500	-3.00482300	-4.88537900
H	3.88609900	-6.33571200	0.15019800
C	-0.99449000	4.21688800	4.14717100
C	-1.60529800	3.12130900	4.80324800
H	-2.65827000	3.17130700	5.05742300
H	-1.58891200	5.09326300	3.91212400
H	-1.34632700	1.11634900	5.55313200
H	0.82934500	4.99408400	3.28583700
C	-0.65099300	6.25913500	-1.76204000
H	-0.25533000	5.12457100	-3.55688200
C	-0.21687600	6.57512000	-0.45321600
H	1.32452100	6.28685900	1.03373500
C	-3.98177900	0.95343600	-3.53117000
H	-2.25034000	2.10705700	-4.11011800
C	-4.50245900	-0.32875500	-3.24112100
H	-4.06252100	-2.43057700	-3.09214200
H	-5.52649100	-0.43121900	-2.90153300
H	-4.63798200	1.81197000	-3.45258700
H	-0.80941200	7.24774400	0.15835300
H	-1.56064400	6.70941900	-2.14216900
P	-0.46197600	-3.43697800	1.21554700
N	-1.96239500	-3.70537500	1.78980500
C	-3.11680800	-3.17027800	1.04552900
C	-2.27480000	-4.72455300	2.79030000
H	-2.81667800	-2.34775400	0.39657600
H	-3.85703900	-2.79205800	1.75119700
H	-3.57959500	-3.95034100	0.43149100
H	-1.37911700	-4.99772200	3.34806700
H	-2.69623100	-5.61987800	2.31807600
H	-3.01208100	-4.30945700	3.48375800
Au	0.02725700	-1.44661900	0.14578800
N	1.16878200	0.73271200	4.91461300
N	2.41397300	2.95582200	3.70052600
N	2.94319900	4.73933100	-0.30725200
N	2.01119100	4.00859500	-2.87752100
N	-0.50849500	0.15663600	-4.38638800
N	-1.55274500	-2.40480300	-3.82989000
C	0.52852200	0.87821700	-0.76667600
C	1.46205300	0.08063500	-0.80726900
H	2.42589700	-0.35833100	-0.97468300
C	-0.47183900	1.95326600	-0.77450000
C	-1.68436800	1.76049700	0.18398800
H	-0.82865700	2.09475100	-1.79824000
H	0.05729700	2.86873900	-0.48454600
C	-2.56297400	0.61536800	-0.34726000
O	-2.07120800	-0.42855400	-0.78086400
O	-3.83290100	0.84718500	-0.25827000
H	-4.40364400	-0.04165000	-0.35250600
C	-1.19441800	1.45312200	1.63271700
H	-0.17782500	1.05761700	1.57737900
H	-1.13476400	2.41388700	2.14660700
C	-2.56784500	3.02860600	0.22053800
O	-2.94883100	3.52695800	1.25886500
O	-2.87574300	3.45706800	-1.00704200
C	-4.04101500	4.35856600	-1.21600100
C	-2.01381500	0.49094400	2.46605100
C	-1.34632600	-0.55373900	3.11320400
C	-3.39204700	0.63613200	2.67739500
C	-2.02230400	-1.43116000	3.96003200
H	-0.27545800	-0.65165200	2.98082500
C	-4.07917700	-0.25945400	3.49585700
H	-3.93752600	1.43548200	2.19571000
C	-3.39665800	-1.29015800	4.14777500
H	-1.47786900	-2.23025200	4.45410400
H	-5.15328200	-0.15867900	3.60054300
H	-3.93843700	-1.97931700	4.78858800
S	-6.29067200	-1.14624000	0.69797500
O	-5.15162800	-1.23225400	-0.30576500
O	-6.49599700	0.21582600	1.21555000
O	-6.29940600	-2.25779000	1.65793400
C	-7.73256900	-1.45766800	-0.42592200
F	-7.73615800	-0.55764800	-1.43048800
F	-7.65879900	-2.68211000	-0.96345000
F	-8.88632200	-1.34805900	0.24129300
C	-3.93803400	5.63976200	-0.38335800

H	-4.65651700	6.36771400	-0.77394700
H	-4.15361000	5.45302000	0.66620900
H	-2.93899500	6.06975500	-0.45863300
C	-3.94813600	4.68086100	-2.70589200
H	-4.78963600	5.31288400	-3.00176000
H	-3.01927500	5.20937200	-2.93094800
H	-3.97367500	3.76977300	-3.30478900
C	-5.31216600	3.57716300	-0.88657700
H	-5.38164600	2.66791900	-1.48448600
H	-5.33946500	3.28663200	0.16427000
H	-6.18318100	4.20596900	-1.09498500

(TfO•1b)⊂AuCav-2

C	3.68944300	-3.90404800	2.86137600
C	3.59429300	-2.41810100	3.19480100
C	2.99820100	-4.22977500	1.54160500
H	3.12273000	-4.42269800	3.63529400
C	4.57051200	-1.49891400	2.80494400
C	2.52128700	-1.92955500	3.95532600
C	3.62787700	-4.05570900	0.30529000
C	1.68324900	-4.71609200	1.52808900
C	4.53390700	-0.15098600	3.17408200
H	5.40892100	-1.85274200	2.21635500
C	2.43067900	-0.59320800	4.33239100
O	1.58668000	-2.84274800	4.42664700
C	2.99964900	-4.34603000	-0.91027800
H	4.63716400	-3.65805100	0.28818400
C	1.03175900	-5.04848600	0.34570600
O	1.01327000	-4.87956600	2.73801400
C	5.62237100	0.83245900	2.75795800
C	3.44687400	0.27426600	3.94690500
H	1.60037800	-0.24656600	4.93369200
C	0.33690700	-2.85979500	3.85987900
C	3.65770400	-4.09400800	-2.25931100
C	1.69688600	-4.84913400	-0.85875600
H	0.01340900	-5.41338000	0.36411400
C	0.02479900	-3.95317700	2.98924800
H	5.58524500	1.65646200	3.47086600
C	5.28380300	1.42468500	1.39525100
O	3.44642300	1.59338500	4.41672200
C	3.55561400	-2.62143000	-2.63854800
H	3.06702300	-4.63816900	-2.99685600
O	1.04903900	-5.12949800	-2.06571400
C	5.62827500	0.78865200	0.19896300
C	4.57170500	2.62841600	1.30188800
C	2.51710600	2.45517500	3.91189900
C	-1.77114200	-2.04669400	3.59567000
C	4.57301700	-1.71798500	-2.32011700
C	2.44940900	-2.12878800	-3.35372200
C	-0.00179900	-4.27951300	-2.31458100
C	-2.08597000	-3.14165300	2.73026900
C	5.31386500	1.30918600	-1.06039400
H	6.16282500	-0.15199800	0.25138400
C	4.23149800	3.18567300	0.07354100
O	4.24152200	3.29858600	2.47104200
C	2.92260000	3.33154200	2.84796500
C	4.57367300	-0.38514000	-2.73736700
H	5.41762800	-2.07893800	-1.74709400
C	2.42119700	-0.81248100	-3.81475700
O	1.44818500	-3.00601900	-3.74110900
C	0.23243600	-3.10231200	-3.09897700
C	5.68418100	0.58961600	-2.35602900
C	4.61177900	2.51879200	-1.08207200
H	3.68745000	4.12052300	0.03224300
C	0.44556300	3.40027000	3.88237400
C	3.49011400	0.02728400	-3.51801800
H	1.59615200	-0.47065700	-4.42722100
C	-2.16489300	-3.64468300	-1.98047300
H	5.72367200	1.34907800	-3.14044100
O	4.29280400	3.11912000	-2.31383300
C	0.82955200	4.22305700	2.77793100
O	3.49903100	1.32635800	-4.06809900
C	-1.93487900	-2.45175000	-2.73928200
C	-3.37117200	-3.22211500	2.14776300
C	-2.75872700	-1.07082300	3.86393400
C	-0.11682400	5.10575400	2.20599500
C	-0.85941400	3.51270700	4.41444700
C	-2.99843600	-1.53617100	-2.90408700
C	-3.43019900	-3.88050600	-1.39931900
C	7.07226200	-0.06329900	-2.30014400
H	7.30681400	-0.52617100	-3.26218600
H	7.15122600	-0.83787000	-1.53337200
C	7.03469600	0.23316500	2.81273900
H	7.77749900	0.99445800	2.55841900
H	7.17331400	-0.60546800	2.12473300
C	5.13043000	-4.43455000	2.90725800
H	5.58028500	-4.22303000	3.88123600
H	5.77454200	-3.99128000	2.14209400
C	5.09383800	-4.63501500	-2.32102700
H	5.08458100	-5.71463500	-2.15096200
H	5.75011100	-4.19435100	-1.56508600
H	5.53592100	-4.44158000	-3.30259100
H	5.13383000	-5.51638400	2.74940300
H	7.24360300	-0.13333800	3.82107300
H	7.83093500	0.69270800	-2.08223900
C	-4.44176400	-2.95808700	-1.55546900
C	-4.22269500	-1.78561700	-2.31779300
H	-5.02362600	-1.06330400	-2.40752900
H	-5.40524000	-3.10707300	-1.08138100
H	-2.84247400	-0.63344100	-3.48192400
H	-3.56253800	-4.79421800	-0.83052400
C	-4.00160000	-1.17302400	3.27529800
H	-2.49889000	-0.24960200	4.52352000
C	-4.31290600	-2.24934400	2.40814200
H	-3.58178300	-4.05446000	1.48741900
C	-1.75608200	4.39481600	3.85283900
H	-1.12529200	2.87707100	5.25160900
C	-1.38681400	5.18518100	2.73796700
H	0.19462600	5.71838700	1.36608600
H	-2.11919600	5.85549800	2.30016100
H	-2.76646000	4.46039700	4.23605300
H	-5.28989200	-2.29861400	1.93688700
H	-4.75087400	-0.40952200	3.45544600
P	3.00305000	2.57222800	-3.14054900
N	2.87017800	3.77232100	-4.29428400
C	2.76162100	5.15126100	-3.79819300
C	1.97342400	3.48357900	-5.42346400
H	3.49507100	5.32426200	-3.01135800
H	1.75461200	5.37857900	-3.41788600
H	2.97951700	5.82975000	-4.62697500
H	2.15157400	2.47389400	-5.79235100
H	2.20134800	4.18813100	-6.22735300
H	0.91352200	3.59073900	-5.15104900
Au	1.13188600	1.86854200	-2.03676100
N	-0.69895400	-2.20886000	-3.29188900
N	-1.15720000	-4.54058900	-1.78078400
N	-1.14518100	-4.09138900	2.44066100
N	-0.52748200	-1.93394800	4.15345900
N	1.32061700	2.49709800	4.41896200
N	2.10463400	4.16809100	2.27966100
C	-1.24848500	1.78204600	-1.55712900
C	-0.82317800	0.86086500	-2.25327400
H	-0.73225200	-0.04657200	-2.82797400
C	-1.86270700	2.77642700	-0.67204000
C	-2.73072000	2.07866900	0.40644800
H	-2.49251900	3.43170200	-1.27744400
H	-1.09414700	3.38396600	-0.18370100
C	-3.69328800	1.11067600	-0.32356400
O	-3.80493200	1.12151400	-1.53672700
O	-4.40609800	0.39581200	0.51765300
H	-5.24199100	0.02662400	0.03580200
C	-1.89148800	1.44019200	1.53442800
H	-2.57714800	0.88544800	2.17353500
H	-1.48944900	2.25430500	2.13799300
C	-3.73098700	3.07224600	1.04481500
O	-3.88181400	3.20382300	2.24029100
O	-4.44377400	3.67117300	0.08789500
C	-5.78268400	4.25517000	0.36474900
C	-0.76916400	0.54344000	1.07552300
C	0.55457800	1.00475800	1.05446400

C	-1.02872700	-0.76449700	0.64759100
C	1.59724200	0.18972700	0.59646400
H	0.77165000	2.00846800	1.40166300
C	0.00669400	-1.58333000	0.19922400
H	-2.04631400	-1.14115000	0.66432600
C	1.32179600	-1.11076400	0.16341500
H	2.61966400	0.55247900	0.61309700
H	-0.21255700	-2.59960300	-0.10185100
H	2.12535000	-1.75597200	-0.17155300
S	-7.55881200	-1.11773100	-0.04134400
O	-8.59293600	-0.44411200	0.74916700
O	-6.53915400	-0.18536900	-0.65646400
O	-6.94602900	-2.33656400	0.53239500
C	-8.41241400	-1.72950300	-1.56856300
F	-8.99417000	-0.71955600	-2.22694900
F	-7.52347100	-2.31528400	-2.39495700
F	-9.34858000	-2.63156400	-1.25184000
C	-6.26875100	4.61636100	-1.03590500
H	-6.30341100	3.72250100	-1.66347200
H	-7.27288300	5.04556500	-0.98096200
H	-5.60199800	5.34845000	-1.50166300
C	-6.67206300	3.18252900	0.99327800
H	-6.71241800	2.28710100	0.36752100
H	-6.31936800	2.90382000	1.98692700
H	-7.68914400	3.57371100	1.08831300
C	-5.62413600	5.49367200	1.24478900
H	-5.26838400	5.22194900	2.23871200
H	-4.91857400	6.19682400	0.79010600
H	-6.59071800	5.99749400	1.34110300

(TfO•1b)⊂AuCav-3

C	-4.67015800	0.07198700	3.77198900
C	-3.18231500	-0.19251200	3.99177700
C	-5.00184100	0.23083400	2.29144300
H	-4.87678900	1.03773100	4.23418700
C	-2.64059000	-1.48064700	4.03197900
C	-2.30056100	0.87584500	4.20420400
C	-5.25896700	-0.87196800	1.46961900
C	-5.07925900	1.49861900	1.70028300
C	-1.29364800	-1.72852400	4.31565800
H	-3.29707600	-2.32508200	3.85833300
C	-0.95298200	0.68578000	4.49121100
O	-2.84251200	2.16316300	4.22684100
C	-5.58997300	-0.75505700	0.11723800
H	-5.20366600	-1.86297000	1.90496800
C	-5.38996200	1.66870600	0.35350100
O	-4.88496800	2.63255600	2.50402600
C	-0.70185900	-3.13432300	4.34051100
C	-0.47183500	-0.61966600	4.55947600
H	-0.30005700	1.52659900	4.68521300
C	-2.60299100	3.00660600	3.18530900
C	-5.86725300	-1.96934700	-0.76734900
C	-5.63309500	0.53831500	-0.42053000
H	-5.43143800	2.65693200	-0.08476900
C	-3.71042600	3.30680200	2.31279500
H	0.18945100	-3.08659200	4.96602800
C	-0.23255100	-3.49699000	2.93535100
O	0.83424100	-0.86034900	4.97562500
C	-4.55196900	-2.49020200	-1.33374400
H	-6.45179800	-1.60966600	-1.61436100
O	-5.98283100	0.67099900	-1.76513000
C	-1.11362900	-4.01704700	1.98429000
C	1.09345200	-3.27484100	2.53364300
C	1.87136300	-0.50272000	4.15714400
C	-1.29016900	4.44656700	2.00827000
C	-3.73863400	-3.37755300	-0.62229900
C	-4.08453100	-2.06216400	-2.58342800
C	-5.00487300	1.04728800	-2.64594400
C	-2.40321100	4.78914300	1.18091500
C	-0.72396000	-4.30821600	0.67608600
H	-2.14320400	-4.19542500	2.27175400
C	1.52772200	-3.53892400	1.23669700
O	1.99922300	-2.80809800	3.48550500
C	2.48059700	-1.53050000	3.35529800
C	-2.48116300	-3.79403200	-1.06899600
H	-4.10542300	-3.77053300	0.31842600
C	-2.82794100	-2.43210800	-3.05417300
O	-4.90904100	-1.25316200	-3.37569600
C	-4.49239000	0.04563100	-3.54151400
C	-1.68490800	-4.87983100	-0.35256100
C	0.60365000	-4.04235600	0.32611400
H	2.54462800	-3.33101400	0.92742400
C	3.39633200	0.99829100	3.38896900
C	-2.00343400	-3.23021600	-2.26162800
H	-2.49303300	-2.08755100	-4.02442200
C	-3.62218200	2.59073400	-3.58683200
H	-1.06648500	-5.35175600	-1.12004800
O	1.01125100	-4.26254100	-0.99489500
C	3.98242800	-0.01368900	2.56450000
O	-0.73649200	-3.51588600	-2.74891700
C	-3.18016500	1.61989200	-4.53646900
C	-2.23285600	5.71926900	0.13004700
C	-0.02761700	5.03113000	1.75189300
C	5.09177400	0.30641600	1.74929300
C	3.96207000	2.29300400	3.41458900
C	-2.20411400	1.97614100	-5.49439300
C	-3.04553200	3.88056200	-3.58944000
C	-2.58466300	-5.97903200	0.23370200
H	-3.22446600	-6.39948200	-0.54677300
H	-3.23464200	-5.61727400	1.03480000
C	-1.64484800	-4.18036800	4.95020800
H	-1.14955300	-5.15444100	4.98481900
H	-2.57306300	-4.30478400	4.38540000
C	-5.56372400	-0.96866300	4.46409100
H	-5.33425700	-1.00957100	5.53235700
H	-5.43823000	-1.97753000	4.06138900
C	-6.69222000	-3.05093000	-0.05622400
H	-7.64222900	-2.63255800	0.28714100
H	-6.18571000	-3.47305900	0.81625400
H	-6.90287900	-3.87408300	-0.74439500
H	-6.61567300	-0.69628600	4.34344300
H	-1.91630200	-3.89199900	5.96945800
H	-1.96742100	-6.77975500	0.64959100
C	-2.07675400	4.19534200	-4.51810600
C	-1.66165700	3.24352100	-5.48009500
H	-0.89992200	3.51399900	-6.20437300
H	-1.62403300	5.18190900	-4.51524200
H	-1.88600000	1.22235700	-6.20638200
H	-3.37756500	4.58837500	-2.83813500
C	0.11612400	5.91955100	0.70520900
H	0.80706300	4.75230400	2.38645500
C	-0.99031300	6.27057000	-0.10405800
H	-3.09543100	5.95902200	-0.48204000
C	5.06975700	2.56707800	2.63867400
H	3.51270000	3.03571200	4.06523800
C	5.62294300	1.57852000	1.79254000
H	5.50112800	-0.45717700	1.10261600
H	6.47150300	1.82392800	1.16288600
H	5.52506100	3.55085400	2.67758700
H	-0.85341600	6.97304800	-0.91988000
H	1.08854700	6.34888800	0.49389100
P	0.72905900	-3.04574000	-2.06837400
N	1.79348100	-3.35060700	-3.26053300
C	2.23399600	-4.69648300	-3.62849000
C	2.22813600	-2.26585400	-4.14368100
H	1.89906400	-5.41823900	-2.88392900
H	3.32644800	-4.69801900	-3.65115500
H	1.83184900	-4.97910300	-4.60906800
H	1.71049700	-2.31872900	-5.11041000
H	3.30657300	-2.35332500	-4.28520200
H	2.04244200	-1.29492600	-3.68166900
Au	0.86951600	-1.01441200	-0.94623500
N	-3.63835800	0.33151200	-4.47927100
N	-4.57895600	2.27495900	-2.66295400
N	-3.61720000	4.18188500	1.35554800
N	-1.42951000	3.54550900	3.02847600
N	2.31026500	0.72000300	4.17365500
N	3.49122100	-1.29133500	2.57752600
C	1.31427200	1.04591500	0.53386900
C	0.64328900	0.11950200	0.98275600

H	0.02603100	-0.46936500	1.63045700
C	2.10111400	2.25703500	0.27310200
C	2.48230000	2.60863000	-1.19167200
H	3.02696600	2.17297600	0.84347400
H	1.53262700	3.08996800	0.69738600
C	3.20607700	1.38753600	-1.79475300
O	2.76271000	0.72192000	-2.71509200
O	4.32099200	1.15774000	-1.13490700
H	4.77469200	0.28063600	-1.37995400
C	1.28654200	3.10817100	-2.06378700
H	1.63112800	3.12509200	-3.09931800
H	1.07885500	4.14113100	-1.76334100
C	3.48248400	3.77363300	-1.22815000
O	4.11616200	4.03324600	-2.22829400
O	3.48218000	4.48491800	-0.09045100
C	4.34835000	5.67381200	0.06082700
C	0.03374200	2.29464200	-1.91172200
C	-0.86915400	2.58924400	-0.88567600
C	-0.20789400	1.18231700	-2.72082600
C	-1.92427700	1.73105900	-0.58751700
H	-0.70849300	3.47750700	-0.29336000
C	-1.26513300	0.32391400	-2.42499200
H	0.46253600	0.96285600	-3.54153800
C	-2.10098300	0.56833500	-1.33242500
H	-2.59902700	1.95737700	0.22669900
H	-1.43306500	-0.53686700	-3.05158800
H	-2.88678000	-0.13297500	-1.07808500
S	4.78651100	-2.29357000	-1.41056800
O	4.81877500	-3.09111600	-2.64568700
O	3.46152400	-2.08656600	-0.77672400
O	5.60913900	-1.03960800	-1.44681600
C	5.67430100	-3.36117900	-0.17744100
F	4.92491500	-4.44516000	0.08629100
F	6.85657800	-3.76190700	-0.65461300
F	5.87974400	-2.70748900	0.97678200
C	5.81383000	5.26223500	-0.08619700
H	6.03114800	4.94046000	-1.10403900
H	6.45595400	6.11248700	0.16250700
H	6.04708200	4.44267200	0.59762800
C	3.94281300	6.74162400	-0.95593000
H	2.87386000	6.96285000	-0.87514900
H	4.49452700	7.66415400	-0.75150700
H	4.16011300	6.41724800	-1.97302500
C	4.04640100	6.13000000	1.48734200
H	4.23643100	5.32111000	2.19572400
H	4.67960000	6.98218000	1.74851800
H	3.00205100	6.43649600	1.59176200

(TfO•1b)⊂AuCav-4

C	-3.30025900	-2.58749700	3.86882300
C	-1.84609100	-2.14207200	3.94891800
C	-3.86158200	-2.41040600	2.46294400
H	-3.86311300	-1.90717300	4.50818300
C	-0.76972100	-3.01176100	3.75232000
C	-1.54676400	-0.81207100	4.26930400
C	-3.66495600	-3.37733700	1.47633100
C	-4.63825300	-1.29698700	2.11491700
C	0.56198800	-2.61238300	3.90275700
H	-0.97664700	-4.04793900	3.51278700
C	-0.24020500	-0.35752900	4.41169700
O	-2.62664900	0.01582100	4.56650000
C	-4.25549800	-3.32231700	0.21121200
H	-3.03543400	-4.22803100	1.71047700
C	-5.24900500	-1.19481800	0.86695300
O	-4.89364600	-0.32105000	3.08169600
C	1.72501500	-3.58574000	3.72160000
C	0.80028100	-1.26832900	4.23253700
H	-0.03158100	0.66885300	4.68430800
C	-2.99688400	1.02991300	3.73765000
C	-3.96689100	-4.41333900	-0.81579300
C	-5.06977700	-2.21526200	-0.06806200
H	-5.90262100	-0.35922700	0.64802200
C	-4.22199100	0.87017500	2.99386700
H	2.55026500	-3.19541500	4.31742700
C	2.18051000	-3.54880300	2.26751500
O	2.10460900	-0.88936700	4.50911800
C	-2.62044400	-4.09476800	-1.45577300
H	-4.72346100	-4.32306700	-1.59573800
O	-5.80411300	-2.16658400	-1.25732000
C	1.52853700	-4.27493700	1.26651200
C	3.23113600	-2.70516000	1.87943800
C	2.79898800	-0.05361100	3.66911700
C	-2.83117400	3.17730400	2.99896100
C	-1.40874100	-4.64425500	-1.03694400
C	-2.57876000	-3.14745800	-2.48789300
C	-5.44471100	-1.22759700	-2.19055100
C	-4.07033200	3.03675400	2.30387500
C	1.85131400	-4.14984400	-0.08803400
H	0.71952000	-4.94005500	1.54778400
C	3.57509500	-2.52876100	0.54214800
O	3.92904500	-2.02037700	2.86464900
C	3.80406700	-0.64817200	2.83937500
C	-0.17922200	-4.27844200	-1.59990900
H	-1.41907100	-5.39732300	-0.25827600
C	-1.38603800	-2.72650600	-3.05815300
O	-3.80641400	-2.74369400	-2.97543500
C	-4.27903200	-1.46908700	-2.99999000
C	1.13445700	-4.93487400	-1.18292900
C	2.85833400	-3.23463500	-0.41554900
H	4.35218100	-1.83155900	0.25535700
C	3.43105000	2.00436100	2.93866100
C	-0.19870300	-3.29312600	-2.59742900
H	-1.38072600	-1.99596600	-3.85726900
C	-5.78816200	0.72137100	-3.31197700
H	1.78357700	-4.89666200	-2.06128400
O	3.09790200	-2.93023900	-1.76401600
C	4.44108500	1.40893300	2.11864200
O	0.97781900	-2.87131500	-3.21103100
C	-4.53494100	0.56424200	-3.98058000
C	-4.62196700	4.15085700	1.63331900
C	-2.16789400	4.42521000	2.99800200
C	5.26599200	2.22128500	1.30962300
C	3.29661100	3.41024400	2.95567100
C	-4.08566600	1.57311100	-4.86045800
C	-6.59641600	1.84226500	-3.60290100
C	0.97077000	-6.41623600	-0.80946100
H	0.48845200	-6.96215600	-1.62481300
H	0.36944300	-6.56324200	0.09172600
C	1.41670300	-5.00425900	4.21927100
H	2.30870800	-5.63022500	4.13094000
H	0.61746000	-5.49415300	3.65552700
C	-3.52124100	-4.01028500	4.40516700
H	-3.15665600	-4.08382900	5.43340800
H	-3.00772500	-4.77571600	3.81681700
C	-4.06121200	-5.82972200	-0.23554000
H	-5.06316600	-6.00218000	0.16594300
H	-3.34921300	-6.01192700	0.57427000
H	-3.86878800	-6.56985000	-1.01735700
H	-4.58759100	-4.25100800	4.39535600
H	1.11260700	-4.97942600	5.26929800
H	1.95152700	-6.86106300	-0.62273300
C	-6.15043300	2.79611800	-4.49298200
C	-4.88280100	2.67086400	-5.10735200
H	-4.53992700	3.44417200	-5.78716600
H	-6.76813400	3.66061200	-4.71509200
H	-3.12044800	1.44566600	-5.33788500
H	-7.55164900	1.92788700	-3.09625300
C	-2.73768700	5.49974400	2.34885200
H	-1.23166200	4.50599500	3.53874600
C	-3.96485600	5.36170700	1.65892000
H	-5.56476800	4.01978100	1.11709800
C	4.13124900	4.18370300	2.17601700
H	2.54170300	3.84496800	3.60233400
C	5.10444000	3.58904400	1.33719900
H	5.99611100	1.75281700	0.66363300
H	5.72992800	4.21113400	0.70566800
H	4.04889100	5.26521400	2.20912100
H	-4.39055000	6.21984300	1.14926800
H	-2.24897300	6.46732100	2.37545900
P	2.02811900	-1.86318200	-2.40537500

N	2.89127700	-1.10902900	-3.55750400
C	3.89751400	-1.80717700	-4.37178000
C	2.48477500	0.20985900	-4.04923600
H	4.23805800	-2.70334900	-3.85565500
H	4.74548700	-1.13180600	-4.50029900
H	3.47596800	-2.08290000	-5.34591100
H	1.93414400	0.12248300	-4.99455700
H	3.38603900	0.80840100	-4.19176300
H	1.85585100	0.71261900	-3.31406700
Au	0.88198700	-0.77763300	-0.70734200
N	-3.80464900	-0.58527800	-3.82579700
N	-6.19557000	-0.18279300	-2.36638600
N	-4.73594100	1.84046800	2.29815400
N	-2.30800600	2.13294900	3.71173200
N	2.60924300	1.23202700	3.71470400
N	4.59873100	0.05344000	2.09322100
C	-0.34713600	0.81650700	0.88718500
C	-0.51977300	-0.39221900	1.03477000
H	-0.88325600	-1.31669900	1.44095000
C	-0.20506400	2.27014100	0.77306200
C	0.44118000	2.74495700	-0.55323300
H	0.39789600	2.61818900	1.61398100
H	-1.20364200	2.70551800	0.86332900
C	1.87912500	2.17773100	-0.58356900
O	2.25183500	1.39426100	0.28096900
O	2.58836300	2.55897200	-1.61346500
H	3.44865200	1.97965700	-1.62756700
C	-0.34265700	2.26020400	-1.81124700
H	-0.23388400	1.17302100	-1.89349600
H	0.14492700	2.70582900	-2.67794200
C	0.56177900	4.27797200	-0.63650800
O	0.57165700	4.86915800	-1.69493100
O	0.70731000	4.83993600	0.56641700
C	0.95096900	6.30053300	0.66980700
C	-1.80951100	2.61866400	-1.76252400
C	-2.26306600	3.84856800	-2.25797000
C	-2.73763600	1.74941300	-1.17027000
C	-3.60562400	4.20700900	-2.14460800
H	-1.55313100	4.52346100	-2.72126100
C	-4.07495900	2.11590600	-1.04061800
H	-2.41051400	0.78111600	-0.80556200
C	-4.51135200	3.34800900	-1.52589200
H	-3.94282600	5.15984900	-2.54203900
H	-4.77400600	1.44317100	-0.55925400
H	-5.55610800	3.62545500	-1.43941500
S	5.80006000	0.96475800	-2.26181000
O	6.62889200	2.05215400	-1.72192600
O	5.79706900	0.77203400	-3.72485100
O	4.42913900	0.87475000	-1.63957100
C	6.55387600	-0.58969300	-1.58785000
F	7.78721500	-0.76555500	-2.07276400
F	6.62123800	-0.54341000	-0.24773700
F	5.80955900	-1.66301100	-1.92395500
C	2.27584000	6.64215500	-0.01276200
H	2.20484900	6.52380400	-1.09334100
H	2.53975300	7.68012300	0.21200900
H	3.07202100	5.99364900	0.35793200
C	-0.22617000	7.07133900	0.06775600
H	-0.22563100	7.00913500	-1.01921900
H	-1.17162100	6.67042500	0.43961800
H	-0.15494700	8.12270200	0.36354300
C	1.03493900	6.51678300	2.17868900
H	1.82859100	5.90461000	2.61017800
H	1.24763100	7.56753200	2.39275800
H	0.09312500	6.24827300	2.66204700

(TfO•1b)⊂AuCav-5

C	4.61647100	-0.70090900	-3.33509000
C	3.19456800	-1.18338800	-3.59857200
C	4.73872700	-0.14729000	-1.91956900
H	4.78741200	0.14000600	-4.00740400
C	2.77452400	-2.49908100	-3.39445200
C	2.24512000	-0.27622900	-4.08194500
C	4.98635700	-0.99830100	-0.83972100
C	4.64458200	1.22421400	-1.63972100
C	1.47734600	-2.93267800	-3.69000300
H	3.49087000	-3.22284600	-3.02393800
C	0.93820400	-0.64600400	-4.37377800
O	2.72113000	0.99353200	-4.38638900
C	5.20634200	-0.55345400	0.46630800
H	5.05193800	-2.06199300	-1.03471900
C	4.81540700	1.71376200	-0.34468600
O	4.52038500	2.13324800	-2.69320000
C	1.05014400	-4.38517000	-3.48146300
C	0.57169000	-1.97809900	-4.18289600
H	0.23069600	0.06593700	-4.77909400
C	2.33217300	2.11441500	-3.72778800
C	5.54580000	-1.55053200	1.57244400
C	5.10846500	0.82873600	0.69440600
H	4.79173700	2.78027000	-0.15634500
C	3.32369600	2.76523400	-2.90753700
H	0.22414000	-4.56693300	-4.16944700
C	0.48584400	-4.52965700	-2.07240100
O	-0.67443700	-2.40855300	-4.61493400
C	4.24782700	-2.11619100	2.13529300
H	6.01545800	-0.97826400	2.37266400
O	5.43846200	1.35228200	1.94736900
C	1.29887800	-4.72471500	-0.95151100
C	-0.88950200	-4.37079700	-1.85606600
C	-1.81518300	-2.05926900	-3.93085000
C	0.93307500	3.88526400	-3.44179500
C	3.60878600	-3.25261300	1.63390800
C	3.60913800	-1.42131300	3.16882400
C	4.43319900	1.83030100	2.75243800
C	1.95876200	4.58135700	-2.73408200
C	0.80026300	-4.70940000	0.35592600
H	2.36290900	-4.87160600	-1.09916500
C	-1.42838600	-4.30820900	-0.57579200
O	-1.72054000	-4.24872700	-2.95667200
C	-2.38477700	-3.04689800	-3.06740700
C	2.35702700	-3.68090000	2.09717900
H	4.09457400	-3.82350400	0.85106400
C	2.36066700	-1.78822700	3.64672300
O	4.29144500	-0.34752600	3.71503900
C	3.76178700	0.90677800	3.62314700
C	1.68037400	-4.95059600	1.58107800
C	-0.56961800	-4.45303100	0.50143600
H	-2.48186300	-4.10188200	-0.42844600
C	-3.57035300	-0.68471100	-3.48634900
C	1.74179800	-2.89938200	3.08641300
H	1.87297500	-1.20985100	4.42031100
C	3.14443100	3.51245500	3.58723800
H	1.00647600	-5.27561400	2.37786800
O	-1.11441400	-4.25630000	1.78082800
C	-4.11587200	-1.65902500	-2.59137100
O	0.44622100	-3.17823500	3.51880900
C	2.38516400	2.56660300	4.34227700
C	1.74090700	5.91865000	-2.33606100
C	-0.30078300	4.52512000	-3.69184600
C	-5.30691100	-1.38556300	-1.88492000
C	-4.27795600	0.51744800	-3.70636900
C	1.28507700	3.00321400	5.11419800
C	2.82190700	4.88405100	3.67803900
C	2.68436300	-6.09162100	1.35177500
H	3.25123600	-6.28399000	2.26655000
H	3.40232800	-5.87147000	0.55728200
C	2.15638200	-5.40008600	-3.79746200
H	1.76657200	-6.41688900	-3.69945700
H	3.02080000	-5.31666000	-3.13221300
C	5.68133800	-1.76231200	-3.64718400
H	5.59322000	-2.08272400	-4.68879600
H	5.59749200	-2.65477400	-3.02087600
C	6.54088800	-2.63004300	1.12610700
H	7.45912700	-2.16582400	0.75693100
H	6.15368300	-3.27080400	0.32900900
H	6.79370200	-3.27519500	1.97201100
H	6.68090100	-1.34683600	-3.49388500
H	2.51447100	-5.26289900	-4.82139500
H	2.15310900	-7.00438800	1.07026600
C	1.75353200	5.28809800	4.44808200

C	0.97363300	4.34485700	5.15752400
H	0.12823400	4.68569700	5.74647700
H	1.49392600	6.34017800	4.49927200
H	0.72050000	2.26219200	5.66960300
H	3.41549500	5.58765000	3.10778200
C	-0.49179700	5.82738600	-3.28000700
H	-1.05857500	3.97057100	-4.23292200
C	0.53499300	6.52875300	-2.60606600
H	2.54143900	6.43529300	-1.82054500
C	-5.46404400	0.74308800	-3.03861800
H	-3.86851000	1.22841900	-4.41600300
C	-5.96615900	-0.19670700	-2.10638600
H	-5.67189900	-2.10269100	-1.16021500
H	-6.87314000	0.01593800	-1.55154400
H	-6.02050100	1.65637400	-3.22471000
H	0.36708700	7.55554600	-2.29802200
H	-1.43252100	6.32756400	-3.48529400
P	-0.78339400	-2.80635200	2.45804400
N	-2.11084000	-2.51047500	3.35440200
C	-3.12350500	-3.52085400	3.70426100
C	-2.17242000	-1.26510000	4.12627400
H	-3.10995500	-4.32617300	2.97331300
H	-4.10363000	-3.04079100	3.67970900
H	-2.92319000	-3.92866000	4.70244000
H	-1.48442200	-0.52471800	3.71252500
H	-1.91767400	-1.44809400	5.17704900
H	-3.18418300	-0.85994700	4.05690200
Au	0.08145700	-1.34701500	0.84191300
N	2.74504000	1.24737100	4.35742900
N	4.15297100	3.09951600	2.75638100
N	3.14689900	3.96516200	-2.44079900
N	1.15951100	2.63238900	-3.94465700
N	-2.38809700	-0.91092100	-4.14056500
N	-3.48580300	-2.85640800	-2.40991400
C	0.39818700	0.58036800	-0.86609300
C	1.28509900	-0.26062900	-0.70632700
H	2.22843400	-0.74424900	-0.88108400
C	-0.62770400	1.60927000	-1.02834100
C	-1.52015100	1.72947700	0.23369400
H	-1.24540700	1.36545700	-1.89357900
H	-0.12039300	2.56002100	-1.21524500
C	-2.28872000	0.39000600	0.35499900
O	-2.40616300	-0.35364900	-0.60767700
O	-2.76542300	0.16848600	1.55348000
H	-3.41313800	-0.64623000	1.51550900
C	-0.70159900	2.07501200	1.51285900
H	-0.00392100	1.25770000	1.72033100
H	-1.40822000	2.12006700	2.34014200
C	-2.62701000	2.79926900	0.10553500
O	-3.19972400	3.22163100	1.08756900
O	-2.87606500	3.15248100	-1.15475100
C	-3.91791700	4.17986200	-1.42839500
C	0.05179600	3.37879200	1.36522600
C	-0.55445100	4.59792000	1.70301900
C	1.34456800	3.40504500	0.82452000
C	0.09686000	5.80740200	1.46580100
H	-1.54786900	4.58882200	2.13518100
C	2.00124000	4.61297100	0.59726500
H	1.84738500	2.47226000	0.58438700
C	1.37279300	5.81855400	0.90474600
H	-0.39330000	6.74080400	1.72721700
H	2.99979000	4.60621800	0.17592400
H	1.88166400	6.76054800	0.72347900
S	-5.83070900	-1.44649200	1.89030300
O	-4.41571600	-1.69335700	1.41567800
O	-6.03913800	-1.84150000	3.29404200
O	-6.84787600	-1.83498700	0.90337500
C	-5.91427600	0.41490400	1.93602300
F	-5.13377500	0.89617200	2.91686600
F	-5.48945000	0.94040300	0.76766900
F	-7.16785700	0.82675600	2.14812600
C	-5.27107100	3.72912100	-0.87633500
H	-5.29932100	3.77028700	0.21031900
H	-6.05055300	4.38344100	-1.27953700
H	-5.48362300	2.70524700	-1.18726300
C	-3.95051900	4.23035400	-2.95260700
H	-4.20154200	3.25040000	-3.35955200
H	-4.70326700	4.95179500	-3.28181200
H	-2.98161500	4.53308800	-3.34949400
C	-3.45164900	5.51324900	-0.84235400
H	-3.46893900	5.49035300	0.24691200
H	-2.43497400	5.73901000	-1.17572600
H	-4.11688400	6.31137200	-1.18638700

(TfO•1b)⊂AuCav-6

C	-3.74642700	-4.33243400	2.30453300
C	-3.20017200	-4.42104300	0.88217600
C	-3.42364000	-2.97347100	2.92128500
H	-3.19568600	-5.06974400	2.88889800
C	-3.89863900	-3.92888400	-0.22672100
C	-1.94492100	-4.99925000	0.64839800
C	-4.29087500	-1.88390900	2.79898200
C	-2.24005300	-2.77856900	3.65367400
C	-3.38782900	-3.98522100	-1.52771000
H	-4.87877900	-3.49159200	-0.06945200
C	-1.38465100	-5.05450700	-0.62557200
O	-1.25816300	-5.54471900	1.73118400
C	-4.03187200	-0.64373400	3.38605800
H	-5.21165400	-2.01602300	2.24279000
C	-1.93263700	-1.55248100	4.24384200
O	-1.41782300	-3.87684400	3.87563800
C	-4.16690500	-3.50361500	-2.75098200
C	-2.10722600	-4.53001500	-1.69092500
H	-0.39723100	-5.46981900	-0.77510600
C	-0.12131900	-4.86735400	2.11351200
C	-4.97752500	0.54450700	3.26595500
C	-2.83729500	-0.50602800	4.09924200
H	-1.01531200	-1.42772100	4.80465000
C	-0.21191000	-3.95784100	3.21593700
H	-3.70969400	-3.98970700	-3.61294700
C	-3.98673800	-2.00576600	-2.96202700
O	-1.57618500	-4.56351000	-2.98069800
C	-4.66646200	1.34527000	2.00717200
H	-4.76089000	1.20112200	4.10837000
O	-2.63350200	0.71817300	4.73968600
C	-4.78987500	-1.07597400	-2.29801100
C	-2.99427200	-1.50473800	-3.81952200
C	-0.58549800	-3.64948700	-3.24458200
C	2.06674400	-4.30723300	1.82804800
C	-5.19846100	0.97374900	0.76920700
C	-3.84945000	2.48585000	2.04344600
C	-1.64057700	1.55889800	4.33955600
C	1.97196800	-3.37836800	2.91383200
C	-4.65197300	0.30639800	-2.44683900
H	-5.56857100	-1.44719200	-1.64373400
C	-2.80582700	-0.13274100	-3.98645300
O	-2.20110400	-2.39462100	-4.53131600
C	-0.89683800	-2.53672500	-4.08899500
C	-4.99636100	1.70530900	-0.40221700
H	-5.80762400	0.07954800	0.72088800
C	-3.63346900	3.25841000	0.90042200
O	-3.33109200	2.92078100	3.25901300
C	-2.00739600	2.72702700	3.57893300
C	-5.58541100	1.28544400	-1.74292000
C	-3.62081100	0.74586300	-3.28407200
H	-2.02075800	0.22466100	-4.64084200
C	1.55302800	-2.88444500	-3.02952600
C	-4.21321200	2.85698600	-0.29892200
H	-3.05107800	4.16895100	0.96419500
C	0.51415900	2.29584200	4.43704300
H	-5.63104100	2.18272900	-2.36455300
O	-3.45022100	2.12508000	-3.53037900
C	1.25412700	-1.79672400	-3.91337100
O	-4.03949200	3.65496900	-1.44987600
C	0.14277300	3.48568500	3.74166400
C	3.08166800	-2.55641200	3.21775900
C	3.26937900	-4.40642800	1.09349100
C	2.26521700	-0.85452800	-4.21461800
C	2.84412200	-2.99314300	-2.46368800
C	1.10330800	4.50831900	3.55712500

C	1.84401300	2.13472400	4.88916700
C	-7.02534500	0.76551700	-1.62033200
H	-7.66197600	1.53808000	-1.18143800
H	-7.10819600	-0.12407600	-0.99147300
C	-5.64388700	-3.92427200	-2.70724200
H	-6.15764900	-3.60166900	-3.61727900
H	-6.18435400	-3.50461700	-1.85386100
C	-5.23702800	-4.69142300	2.38909800
H	-5.39713600	-5.70388100	2.00887100
H	-5.87505400	-4.02001400	1.80676900
C	-6.45854000	0.14758400	3.35843600
H	-6.64472500	-0.36405900	4.30622800
H	-6.77498100	-0.52493500	2.55638200
H	-7.09256200	1.03746100	3.31310500
H	-5.57728100	-4.65186700	3.42758100
H	-5.71600700	-5.01244300	-2.63427100
H	-7.41662200	0.50957100	-2.60820100
C	2.75994700	3.14549600	4.69725400
C	2.38522300	4.34150200	4.03882700
H	3.12241200	5.12523200	3.90017100
H	3.78085100	3.02724400	5.04437500
H	0.80046300	5.42551000	3.06225500
H	2.10089100	1.20957800	5.39195200
C	4.33079500	-3.58356000	1.39974200
H	3.31352200	-5.12166700	0.27918300
C	4.23289800	-2.65461600	2.46520400
H	2.97878800	-1.83647700	4.02197300
C	3.81074700	-2.06145200	-2.77114100
H	3.04376400	-3.81459000	-1.78597600
C	3.51712200	-0.99018800	-3.65203300
H	2.02342400	-0.04032300	-4.89024700
H	4.29268100	-0.26258800	-3.85737000
H	4.80039600	-2.15154300	-2.33737700
H	5.07488200	-1.99817900	2.65476600
H	5.23242100	-3.61730000	0.79859100
P	-2.87378800	3.24446600	-2.51629800
N	-2.91337200	4.56940500	-3.53040600
C	-2.27975000	4.43242900	-4.85037500
C	-2.66559000	5.87070800	-2.89322000
H	-2.55922300	3.48243900	-5.30310100
H	-1.18382900	4.50479800	-4.79421700
H	-2.64786300	5.24034100	-5.48777400
H	-3.21522000	5.93899800	-1.95456400
H	-3.03399900	6.65212500	-3.56266500
H	-1.59632800	6.04540900	-2.70466200
Au	-0.96709500	2.99730800	-1.25356700
N	-1.13941600	3.65255700	3.28035700
N	-0.41736500	1.34012100	4.72159700
N	0.80015100	-3.24316200	3.61117900
N	0.98243500	-5.04905200	1.45294400
N	0.59501700	-3.80833700	-2.72335500
N	-0.00810700	-1.64716100	-4.42424000
C	1.16756200	3.28253700	-0.21489100
C	0.27680000	3.85734100	0.41068300
H	-0.32445700	4.35113300	1.14955200
C	2.30499700	2.53893100	-0.74496500
C	2.76930800	1.48994400	0.29271700
H	3.13186700	3.22469300	-0.95257000
H	2.05221900	2.04409700	-1.68419900
C	3.77462400	0.55972600	-0.40935700
O	4.30105500	0.86838600	-1.46001200
O	4.00943900	-0.50551500	0.32396700
H	4.96227200	-0.87062300	0.18754500
C	3.48905700	2.14604600	1.50276000
H	2.76129400	2.77856600	2.01205000
H	3.73834700	1.33695500	2.18999100
C	1.59071800	0.67485800	0.83124500
O	1.44311700	0.37800400	1.99609300
O	0.74501200	0.36103100	-0.16748300
C	-0.41249000	-0.52653800	0.08153800
C	4.71043200	2.94938700	1.13499400
C	4.62356700	4.34211700	1.00598600
C	5.93968400	2.32282800	0.88304400
C	5.73477800	5.09652400	0.62519000
H	3.67673000	4.83634500	1.21398800
C	7.04683300	3.07366000	0.49234100
H	6.04197100	1.24645400	0.98034300
C	6.94876900	4.46070600	0.36302600
H	5.65209800	6.17597000	0.53399000
H	7.98087900	2.56444900	0.28032000
H	7.81425500	5.04271100	0.06049500
S	7.29379300	-1.91535200	-0.43800800
O	8.57489400	-2.28260500	0.16713600
O	6.41320900	-1.08348800	0.46771900
O	6.53275500	-2.96543700	-1.14771800
C	7.73019100	-0.69655700	-1.77055000
F	8.21300500	0.43768500	-1.23477800
F	8.66093100	-1.20879100	-2.58528200
F	6.65093100	-0.38489000	-2.50773300
C	0.09623200	-1.89034500	0.51498400
H	0.57618800	-1.82573200	1.48655100
H	-0.74894700	-2.57725300	0.57458200
H	0.80894100	-2.28654800	-0.21128800
C	-1.07305900	-0.60558900	-1.27777800
H	-1.92470100	-1.28637600	-1.23791600
H	-1.43810600	0.37628700	-1.59018500
H	-0.36432400	-0.95429600	-2.02473400
C	-1.35675400	0.10129600	1.09948400
H	-1.64591700	1.10942100	0.78408400
H	-2.26533200	-0.50186700	1.16336700
H	-0.89710600	0.15013200	2.08280200

(TfO•1b)⊂AuCav-7

C	4.91919900	0.22773900	-3.61130100
C	4.15367300	-1.04943500	-3.28148200
C	4.52460600	1.39830300	-2.71462300
H	4.61007500	0.50839500	-4.61890100
C	4.57161000	-1.94501700	-2.28765500
C	2.99463700	-1.37880600	-3.99759900
C	5.15212100	1.64845700	-1.48970600
C	3.53699200	2.30738500	-3.12235800
C	3.89064700	-3.13482100	-2.01613400
H	5.45526200	-1.70147100	-1.70805000
C	2.29662700	-2.55996500	-3.77767500
O	2.53771700	-0.50621200	-4.98233100
C	4.86864100	2.77201600	-0.70639700
H	5.90228700	0.94800200	-1.14007000
C	3.23933200	3.45102500	-2.39262500
O	2.88169800	2.08738100	-4.33342500
C	4.25043600	-4.08841100	-0.87903200
C	2.76237400	-3.41280200	-2.79078000
H	1.40807700	-2.79187900	-4.34840100
C	1.42025300	0.21575700	-4.63459800
C	5.50540200	2.99842500	0.66232700
C	3.92344200	3.67885300	-1.20362900
H	2.48999200	4.14964500	-2.74023200
C	1.60592900	1.58671300	-4.26257300
H	3.83990800	-5.06425700	-1.13848500
C	3.51486600	-3.61548300	0.37042700
O	2.13497100	-4.62944200	-2.54848000
C	4.65938200	2.29011700	1.71723900
H	5.43045700	4.06641500	0.86875500
O	3.70004200	4.85797800	-0.49721400
C	4.09764900	-2.65720500	1.20317400
C	2.22905300	-4.06286600	0.71391400
C	0.84138300	-4.66988300	-2.14695700
C	-0.81800500	0.42185200	-4.28431600
C	4.83877400	0.93841200	2.02864200
C	3.61936800	2.96971200	2.37048400
C	2.45286500	5.00547100	0.05758200
C	-0.63430900	1.78744000	-3.89595800
C	3.47836100	-2.13426800	2.33882000
H	5.10145000	-2.32499800	0.97140200
C	1.53920700	-3.50688600	1.79077300
O	1.67112500	-5.17020200	0.06502300
C	0.60039100	-5.05857000	-0.78059200
C	4.02603700	0.25699900	2.93966800
H	5.63813100	0.39533900	1.53776100
C	2.77585200	2.32660000	3.27147800
O	3.44534400	4.32302900	2.12868500

C	2.30741100	4.69607800	1.44527500
C	4.22181600	-1.20970400	3.29724200
C	2.16131700	-2.54277200	2.57998000
H	0.53969800	-3.85045700	2.02444200
C	-1.38648300	-4.77840000	-2.58470200
C	2.98955100	0.98125500	3.53231500
H	1.96844600	2.87214900	3.74290500
C	0.24502700	5.53989600	-0.05349900
H	3.75879300	-1.35141100	4.27709400
O	1.46092000	-2.06473800	3.69754100
C	-1.60737900	-5.33620600	-1.28865500
O	2.11759700	0.33825500	4.42252000
C	0.09245400	5.20509200	1.33093600
C	-1.76031200	2.56455500	-3.53678300
C	-2.12149700	-0.12716500	-4.31307200
C	-2.89529100	-5.81646600	-0.96223500
C	-2.47148800	-4.60983500	-3.47519600
C	-1.17855000	5.32349600	1.93749000
C	-0.87254700	5.99532700	-0.78843200
C	5.70438000	-1.58311800	3.44998600
H	6.16536400	-0.95635600	4.21778800
H	6.27647800	-1.44491400	2.52913200
C	5.76207000	-4.25795600	-0.68234600
H	5.96334200	-4.94556500	0.14377900
H	6.27743800	-3.31846300	-0.46447800
C	6.43909100	0.00145300	-3.64097200
H	6.68641200	-0.78861800	-4.35539400
H	6.84615000	-0.29458300	-2.66982100
C	6.98899200	2.61136700	0.71752800
H	7.55099100	3.18071800	-0.02758900
H	7.16447800	1.55012900	0.51850000
H	7.40001500	2.83268800	1.70649400
H	6.94918600	0.91943700	-3.94503700
H	6.20795700	-4.66825200	-1.59225900
H	5.80256000	-2.63071600	3.74679600
C	-2.09624300	6.11089000	-0.16831400
C	-2.25208700	5.76620100	1.19657100
H	-3.24030900	5.83061100	1.63586100
H	-2.96248700	6.44045700	-0.72915200
H	-1.27484400	5.05508600	2.98419200
H	-0.73158500	6.24002700	-1.83559600
C	-3.20239500	0.66499000	-3.98773000
H	-2.23227800	-1.16410000	-4.61425000
C	-3.02256500	2.01327800	-3.59795800
H	-1.61180600	3.58971300	-3.21635100
C	-3.72716100	-5.05019000	-3.11348400
H	-2.27174600	-4.16382800	-4.44355500
C	-3.93101000	-5.68167500	-1.86383700
H	-3.03812100	-6.29610300	-0.00382300
H	-4.91601800	-6.06250700	-1.61310400
H	-4.56167400	-4.93402000	-3.79724700
H	-3.87492300	2.61943300	-3.31815200
H	-4.20712800	0.25441900	-4.01842800
P	0.87390500	-0.53258400	3.78478700
N	-0.19402000	-0.69914200	5.02936800
C	0.16992000	-1.42652800	6.24733200
C	-1.26346900	0.29135600	5.19081600
H	0.92088900	-2.18299700	6.02139800
H	-0.72204200	-1.92955300	6.63391100
H	0.55799600	-0.74804900	7.01706200
H	-1.54788800	0.70305100	4.22111200
H	-0.96179400	1.10596600	5.86097200
H	-2.14046300	-0.20993300	5.61128900
Au	0.02413200	0.27176600	1.88061200
N	1.16722300	4.77821900	2.06467500
N	1.45819500	5.41706700	-0.67045300
N	0.61713500	2.34420200	-3.89050600
N	0.25186000	-0.35360000	-4.63881100
N	-0.11959100	-4.47519800	-2.99930200
N	-0.58466900	-5.41300300	-0.37372100
C	0.24923200	-1.22595500	-0.66828400
C	1.39496100	-0.83681400	-0.62864800
H	2.40581600	-0.49782500	-0.59918300
C	-1.09416200	-1.76669400	-0.85700600
C	-2.32256200	-1.08964000	-0.15935000

H	-1.10791400	-2.81345600	-0.54639000
H	-1.29607000	-1.74989900	-1.93383200
C	-2.20590400	0.44761700	-0.17620400
O	-1.25101300	1.06536600	0.35640400
O	-3.14964800	1.05491500	-0.78355600
H	-3.30712100	2.10959200	-0.50288900
C	-3.57040300	-1.60204100	-0.95457500
H	-3.63821300	-0.98484200	-1.85343800
H	-3.33575100	-2.61854300	-1.27733300
C	-2.51735000	-1.51247000	1.31342500
O	-2.68357100	-0.71223700	2.21708900
O	-2.57211800	-2.83568600	1.44007100
C	-3.01294500	-3.46244800	2.72341800
C	-4.90799500	-1.64881400	-0.24335300
C	-5.62453700	-2.85362600	-0.26527000
C	-5.48005700	-0.54715500	0.40666400
C	-6.87709100	-2.96169600	0.33806900
H	-5.18861300	-3.71419900	-0.76094700
C	-6.72947300	-0.65504900	1.01763800
H	-4.96222600	0.39970700	0.43431800
C	-7.43344300	-1.85905800	0.98787400
H	-7.41351300	-3.90585800	0.30501200
H	-7.15526700	0.21698300	1.50251000
H	-8.40763500	-1.93580400	1.46135900
S	-4.76097900	4.11823300	-0.68272400
O	-4.36100200	4.44778700	-2.06013400
O	-5.25361100	5.20010700	0.17308600
O	-3.76134500	3.22970000	0.04260400
C	-6.20228800	2.96493100	-0.89072500
F	-7.22626400	3.58532600	-1.48035600
F	-5.84168700	1.91237900	-1.65138600
F	-6.60889600	2.49931400	0.30161900
C	-4.36196200	-2.89815200	3.16901000
H	-4.28179200	-1.86248000	3.49381400
H	-5.09335600	-2.95932400	2.36247000
H	-4.72349000	-3.50328400	4.00662200
C	-1.93070200	-3.26127500	3.77713600
H	-2.23437500	-3.76728300	4.69910900
H	-0.97998600	-3.69411100	3.45834300
H	-1.77842800	-2.20710800	3.99323200
C	-3.14314600	-4.93212700	2.34582400
H	-2.20691000	-5.30567600	1.92327900
H	-3.38929300	-5.51768100	3.23607600
H	-3.94319200	-5.06069400	1.61366900

(TfO•1b)⊂AuCav-8

C	-5.92240200	1.56389500	-0.80124500
C	-5.41208700	0.71623200	0.35689500
C	-4.75096400	1.89033600	-1.71914300
H	-6.25987700	2.50829200	-0.37446500
C	-5.42566300	-0.67874000	0.34776700
C	-4.89321200	1.35189700	1.49402700
C	-4.35470700	0.99766100	-2.71362800
C	-4.02577200	3.08435200	-1.60494700
C	-4.96926200	-1.44544100	1.42531400
H	-5.84255900	-1.18762000	-0.51298600
C	-4.38524800	0.63623500	2.57603300
O	-5.07854800	2.72148400	1.55504200
C	-3.31769600	1.25533600	-3.61028200
H	-4.90490600	0.07016500	-2.81938500
C	-2.93961100	3.35907000	-2.43681100
O	-4.51286000	4.08076700	-0.75445600
C	-5.08501600	-2.96688200	1.44159700
C	-4.43171100	-0.75780100	2.52330800
H	-4.01306300	1.14471300	3.45649200
C	-4.10857600	3.67705200	1.60224700
C	-3.02719900	0.27850300	-4.74438400
C	-2.59101400	2.44419300	-3.43459800
H	-2.40964200	4.30075600	-2.35745100
C	-3.87320800	4.43773600	0.39833100
H	-5.09997100	-3.26033600	2.49141400
C	-3.84621200	-3.61255000	0.83340900
O	-4.05459900	-1.50816700	3.63846500
C	-2.14214900	-0.84808500	-4.23189200
H	-2.43857300	0.82622200	-5.48029000

O	-1.61080700	2.79070300	-4.36080600
C	-3.67884900	-3.74967300	-0.54834000
C	-2.80826900	-4.06661900	1.65845900
C	-2.72562500	-1.67402500	3.91405700
C	-2.82977100	5.14207200	2.78601900
C	-2.63438200	-2.03837300	-3.69231700
C	-0.75184500	-0.67178500	-4.27433200
C	-0.27431500	2.69087300	-4.03108300
C	-2.65346400	5.93419200	1.61132300
C	-2.53049300	-4.30044900	-1.12913600
H	-4.46261500	-3.38468500	-1.20201300
C	-1.66039800	-4.64794100	1.13197800
O	-2.92591100	-3.91235900	3.03680600
C	-2.13011400	-2.94556900	3.60688100
C	-1.79554800	-3.05024100	-3.20675700
H	-3.70668400	-2.19107400	-3.66124900
C	0.12246800	-1.63896300	-3.79428900
O	-0.28888000	0.46254900	-4.89885000
C	0.40000800	1.44091100	-4.22016000
C	-2.33295900	-4.36876300	-2.64403100
C	-1.53665300	-4.74540000	-0.24914300
H	-0.85782900	-4.96170400	1.78696800
C	-0.74116000	-0.96151500	4.77936700
C	-0.41681900	-2.81054000	-3.27607200
H	1.19266600	-1.48120500	-3.84108000
C	1.70616300	3.62443400	-3.39810200
H	-1.55801700	-5.11825600	-2.82231600
O	-0.29618900	-5.18233300	-0.75295800
C	-0.14586800	-2.22339200	4.46503500
O	0.49077700	-3.78497300	-2.82912100
C	2.33918800	2.33997700	-3.44218800
C	-1.94549600	7.15520800	1.69506500
C	-2.28649700	5.58424500	4.01415500
C	1.22045800	-2.44199600	4.75796900
C	0.04558900	0.04507900	5.38810900
C	3.69176200	2.21273200	-3.05445900
C	2.47791300	4.76405700	-3.07877500
C	-3.59733200	-4.85459200	-3.36901900
H	-3.40681400	-4.93558500	-4.44220100
H	-4.44913100	-4.18308600	-3.23393600
C	-6.38400200	-3.47652300	0.79890900
H	-6.45476800	-4.56176800	0.91176200
H	-6.45062600	-3.25303600	-0.26947900
C	-7.11434200	0.93997200	-1.53907700
H	-7.93810100	0.76473000	-0.84140500
H	-6.87686500	-0.01528200	-2.01563600
C	-4.30009600	-0.21840100	-5.44422200
H	-4.86916600	0.63104000	-5.83077500
H	-4.96430500	-0.78277200	-4.78341900
H	-4.03656000	-0.86988800	-6.28227900
H	-7.46142000	1.61772800	-2.32327500
H	-7.24884300	-3.01916200	1.28658200
H	-3.89056200	-5.83727400	-2.99053400
C	3.81165600	4.61666700	-2.75906700
C	4.41514900	3.33594300	-2.71225800
H	5.44763100	3.23280500	-2.39305200
H	4.40520000	5.48914800	-2.50383900
H	4.14648800	1.22998300	-3.01687000
H	1.98621000	5.73093400	-3.07646800
C	-1.60598800	6.78216900	4.07056600
H	-2.43781100	4.96634700	4.89255100
C	-1.43742400	7.57168900	2.90800400
H	-1.82929200	7.74126700	0.78991900
C	1.37936200	-0.18924600	5.64827200
H	-0.42743300	0.99405100	5.61677500
C	1.97091800	-1.43455600	5.32646900
H	1.65705500	-3.39919500	4.49935000
H	3.02958200	-1.58215300	5.50585500
H	1.99493200	0.59170700	6.08066600
H	-0.90000700	8.51220500	2.97502400
H	-1.19585700	7.12752700	5.01409700
P	0.69121600	-3.95305700	-1.21245400
N	2.17832300	-4.62373600	-1.08717800
C	2.49386800	-5.89596400	-0.43956500
C	3.32150900	-3.87664200	-1.62828800
H	1.59135500	-6.34662600	-0.03069100
H	3.21167700	-5.72026100	0.36714100
H	2.93057600	-6.58565200	-1.17094400
H	3.02203200	-2.86824500	-1.92180300
H	3.72169800	-4.38797200	-2.51024200
H	4.09508000	-3.78383000	-0.86356600
Au	-0.05716000	-2.03985300	-0.10203300
N	1.64422200	1.25417800	-3.89896900
N	0.36780900	3.75696400	-3.65476100
N	-3.17536200	5.53471100	0.41147900
N	-3.57904200	3.99718100	2.74591600
N	-2.05238700	-0.71511900	4.47916700
N	-0.88237000	-3.20727900	3.86200600
C	-0.06872000	-0.31111700	1.54666700
C	-1.16477100	-0.45468100	0.99708600
H	-2.20040000	-0.32334100	0.75093100
C	1.19848100	-0.02558800	2.20668800
C	2.28952800	0.50183000	1.25036400
H	1.56808400	-0.89235900	2.75172700
H	0.98754800	0.74631300	2.95112000
C	3.48263500	1.00401600	2.08525400
O	3.74427400	0.56909500	3.18932300
O	4.16680600	1.88497100	1.38058700
H	5.17702800	1.86839400	1.50398900
C	2.86354800	-0.55250300	0.27190600
H	2.05244800	-0.88759100	-0.38738500
H	3.57848300	-0.03062000	-0.36811400
C	1.69152400	1.67835400	0.48159800
O	1.51985300	1.71454800	-0.71732500
O	1.33657900	2.61040200	1.37423900
C	0.99004500	3.98969500	0.99163400
C	3.55089300	-1.72149100	0.94589900
C	2.85267300	-2.74433900	1.60461400
C	4.95048500	-1.79106800	0.91263400
C	3.52966300	-3.80212500	2.21377400
H	1.76733900	-2.73303300	1.64702300
C	5.63060600	-2.84687900	1.51935400
H	5.49941200	-1.01689300	0.39019200
C	4.92437800	-3.85753300	2.17420000
H	2.96542500	-4.58267000	2.71754200
H	6.71491700	-2.86809900	1.47849200
H	5.45252000	-4.67816300	2.65086100
S	6.85231800	1.47577000	-0.37717500
O	7.29467700	2.64524000	-1.15202300
O	5.77725000	0.65930600	-0.99296600
O	6.66677600	1.71823800	1.09411700
C	8.30689100	0.32449300	-0.37514600
F	8.61728100	-0.03535200	-1.62816300
F	9.37846800	0.89650900	0.18383200
F	8.01711500	-0.79675600	0.31935200
C	2.22694300	4.62124700	0.36443600
H	2.51522700	4.09585500	-0.54211600
H	2.01923500	5.66399600	0.10602500
H	3.06479000	4.58796700	1.06480000
C	0.66972200	4.62295900	2.33950300
H	-0.17702100	4.12006900	2.81194600
H	1.53371500	4.55028400	3.00542400
H	0.41460800	5.67542400	2.20853000
C	-0.22251300	3.99244500	0.06360400
H	0.01110200	3.54241700	-0.89877800
H	-1.04875200	3.43914000	0.52181400
H	-0.55562700	5.01960100	-0.10027600

(TfO•1b)⊂AuCav-9

C	5.26077000	3.33978000	1.11276700
C	5.12212100	2.47638000	-0.13442200
C	4.03888700	3.15710100	2.00744200
H	5.24502600	4.37593100	0.77545700
C	5.56926300	1.15333900	-0.17964200
C	4.52711700	2.99919800	-1.29062500
C	3.96164800	2.12947100	2.94702000
C	2.94645100	4.03107400	1.92663600
C	5.45115700	0.35449800	-1.31901200
H	6.05534100	0.74198100	0.69693500
C	4.33992900	2.22718900	-2.43775900

O	4.23957700	4.36323600	-1.30328100
C	2.88426700	1.96821100	3.82143000
H	4.79021200	1.43471300	3.01930000
C	1.83836500	3.90229900	2.76270000
O	3.05194900	5.11791500	1.05492900
C	6.02768400	-1.05645000	-1.40106400
C	4.80165200	0.91263800	-2.42779500
H	3.88575300	2.65335100	-3.32318000
C	2.94210700	4.79557000	-1.33459600
C	2.89140500	0.84863700	4.85987000
C	1.81449300	2.87067400	3.70509800
H	1.02117900	4.61104000	2.71053100
C	2.30964000	5.16003600	-0.09152900
H	6.21346700	-1.25249800	-2.45704000
C	5.00011400	-2.08445800	-0.94576500
O	4.75789900	0.12974100	-3.58694000
C	2.39311300	-0.43733000	4.21184200
H	2.15565400	1.12276100	5.61559400
O	0.76552100	2.80707000	4.61918600
C	4.80181800	-2.36225200	0.40916900
C	4.20931300	-2.78400700	-1.86801800
C	3.59642300	-0.43247000	-4.01331100
C	1.05381400	5.38143900	-2.46692800
C	3.23206300	-1.36526400	3.59471400
C	1.01556600	-0.69534500	4.19661100
C	-0.46258900	2.32873000	4.20714800
C	0.41392000	5.71281800	-1.23348500
C	3.86589200	-3.28977800	0.87075200
H	5.39865500	-1.82683200	1.13778700
C	3.25108900	-3.71114400	-1.45399700
O	4.42226700	-2.57624200	-3.23539500
C	3.46182900	-1.86606400	-3.91096400
C	2.74682500	-2.51946400	2.96923800
H	4.30215700	-1.19907500	3.62088100
C	0.47574600	-1.79631300	3.53533800
O	0.22650100	0.15190100	4.94447100
C	-0.72802500	0.92695400	4.33157900
C	3.67149600	-3.56347300	2.35630400
C	3.08575300	-3.94461600	-0.08847900
H	2.65323900	-4.23845700	-2.18722600
C	1.58212800	-0.33112900	-5.08479900
C	1.35814100	-2.68421900	2.93118900
H	-0.59595500	-1.95341400	3.51281500
C	-2.57799100	2.63095100	3.40985300
H	3.15733700	-4.52431900	2.43425200
O	2.08568900	-4.84178100	0.33149000
C	1.49687500	-1.75850600	-5.06744000
O	0.80649000	-3.86047500	2.38200400
C	-2.80348400	1.21632700	3.45434300
C	-0.93367600	6.14030800	-1.23862400
C	0.33251800	5.49151000	-3.67788700
C	0.35920100	-2.39539400	-5.61416600
C	0.50982400	0.42656500	-5.61022200
C	-4.01793500	0.67808200	2.98067000
C	-3.59917200	3.47193600	2.90829100
C	5.00463300	-3.71155700	3.10417600
H	4.82082300	-3.93685200	4.15785500
H	5.62106900	-2.81020900	3.06131500
C	7.36764100	-1.19890100	-0.66329600
H	7.77040800	-2.20474100	-0.81006600
H	7.28516000	-1.03157700	0.41408600
C	6.58430700	3.11765900	1.85860900
H	7.42839100	3.30763100	1.18983800
H	6.69474400	2.10197700	2.24883900
C	4.24649000	0.69236900	5.56321300
H	4.52833800	1.63303000	6.04337300
H	5.05778500	0.41741700	4.88290700
H	4.18284700	-0.08369200	6.33122900
H	6.65239800	3.80258800	2.70786700
H	8.08772800	-0.47542700	-1.05439800
H	5.58677900	-4.52776000	2.66843700
C	-4.77501500	2.91767800	2.44445500
C	-4.98818800	1.51740800	2.47976600
H	-5.91217500	1.10361700	2.09104100
H	-5.54905800	3.55054600	2.02259400
H	-4.14985600	-0.39589100	3.00486000
H	-3.41143700	4.53981700	2.87291500
C	-0.98342000	5.90256800	-3.65498200
H	0.84084600	5.23423400	-4.60087700
C	-1.61933100	6.22619800	-2.43148000
H	-1.39942000	6.37797000	-0.28882000
C	-0.60148700	-0.21849400	-6.10995800
H	0.58983300	1.50790200	-5.59201200
C	-0.67300300	-1.63409000	-6.12000600
H	0.31986900	-3.47918000	-5.59634200
H	-1.55494900	-2.11829500	-6.52682200
H	-1.43197700	0.35879400	-6.50316300
H	-2.65771300	6.54100500	-2.43697400
H	-1.54358000	5.97853400	-4.58146500
P	0.64155300	-4.16971800	0.80129900
N	-0.46942900	-5.37521200	0.84167400
C	-1.23275200	-5.71039200	-0.36100000
C	-0.76833600	-6.19959300	2.01615000
H	-0.97542900	-5.03387600	-1.17942900
H	-2.30372000	-5.61048000	-0.15383400
H	-1.01558800	-6.73533200	-0.68052200
H	-0.17952500	-5.87116000	2.86989400
H	-0.54455600	-7.25095200	1.80504500
H	-1.83106500	-6.09795200	2.26142800
Au	0.24926100	-2.46283800	-0.67318000
N	-1.84445400	0.38352200	3.95706800
N	-1.36868600	3.15891500	3.78124700
N	1.08489800	5.59737400	-0.04756600
N	2.33981900	4.91665100	-2.48153800
N	2.67137300	0.30638500	-4.56009900
N	2.46030600	-2.50350400	-4.44508400
C	-0.66920400	-1.08590500	-2.50465500
C	0.54730700	-1.05703300	-2.32002200
H	1.57505100	-0.78134200	-2.42200100
C	-2.10716400	-0.95029500	-2.66133200
C	-2.85782900	-0.82572600	-1.29183700
H	-2.51986200	-1.78107600	-3.23896700
H	-2.27233600	-0.04291600	-3.25003700
C	-4.10080000	0.06062400	-1.54263000
O	-4.63623400	0.08303100	-2.63559500
O	-4.44803200	0.70374100	-0.45787000
H	-5.35873600	1.20955000	-0.53731900
C	-3.28661000	-2.25406000	-0.85138300
H	-3.96360300	-2.61123800	-1.63636000
H	-2.39029800	-2.88674100	-0.89823200
C	-1.93558300	-0.13733400	-0.28269900
O	-1.63756700	-0.57833100	0.80928400
O	-1.44194000	0.95934700	-0.85900600
C	-0.40930000	1.84135000	-0.27760400
C	-3.93722400	-2.44149300	0.50002700
C	-5.26267900	-2.05571500	0.72388100
C	-3.22775900	-3.05346100	1.54192200
C	-5.86143500	-2.26106900	1.96778500
H	-5.84437600	-1.58279700	-0.05743700
C	-3.82484700	-3.27291100	2.78161000
H	-2.19324700	-3.34041400	1.38492000
C	-5.14709200	-2.87458900	2.99769100
H	-6.87684700	-1.91169000	2.11621200
H	-3.25602100	-3.73977500	3.58132800
H	-5.61251700	-3.03222900	3.96606100
S	-7.87011100	1.20178200	-0.05809900
O	-9.02471500	2.08304400	0.12648900
O	-7.60817700	0.18254100	0.98136500
O	-6.62150000	1.93173100	-0.49598600
C	-8.27682200	0.17779400	-1.55100900
F	-9.41004200	-0.50961700	-1.34913600
F	-8.42827800	0.94116200	-2.63846400
F	-7.29094200	-0.71142200	-1.79611700
C	-1.18439000	2.96579500	0.39022600
H	-1.87049000	2.55127400	1.12356300
H	-0.51096000	3.66339500	0.89149700
H	-1.77036000	3.51280800	-0.35172300
C	0.35110700	2.32334800	-1.50723500
H	0.85175600	1.49942500	-2.01837800
H	-0.31845200	2.81826200	-2.21341800

H	1.11762400	3.02731700	-1.19455500
C	0.55263000	1.13623600	0.68317900
H	0.07430500	0.85183500	1.61632200
H	0.97764800	0.23406600	0.23319800
H	1.37593800	1.82241000	0.89788600

(TfO•2b)⊂AuCav-1

C	-5.41745800	-2.91933600	1.12439800
C	-4.59690000	-3.15301900	-0.14121300
C	-4.77148100	-1.84598800	2.00045200
H	-5.36783100	-3.84741500	1.69516600
C	-4.78236300	-2.38727600	-1.29829400
C	-3.60228100	-4.14006600	-0.18271900
C	-5.05795300	-0.48123200	1.86697500
C	-3.85532800	-2.21124800	2.99590500
C	-4.01675700	-2.56202600	-2.45397700
H	-5.54872400	-1.62097200	-1.29339400
C	-2.80439800	-4.34318600	-1.30423200
O	-3.43101500	-4.97517900	0.92011900
C	-4.47957200	0.49462900	2.68605300
H	-5.76287200	-0.17044200	1.10382700
C	-3.25138300	-1.28089700	3.82979400
O	-3.59227300	-3.56842000	3.19199600
C	-4.20134400	-1.72156800	-3.71274100
C	-3.01437400	-3.53758300	-2.41380700
H	-2.03528500	-5.10376100	-1.30289200
C	-2.27477800	-4.80889200	1.63648000
C	-4.77383500	1.98888900	2.56764000
C	-3.57392500	0.05820900	3.65871300
H	-2.54017000	-1.58891500	4.58311200
C	-2.35683500	-4.03417700	2.84219800
H	-3.79603300	-2.30577300	-4.53935500
C	-3.35238600	-0.45963500	-3.61858700
O	-2.25604800	-3.72066000	-3.56769000
C	-3.83794400	2.63961200	1.55403700
H	-4.52888900	2.42665300	3.53549800
O	-3.01639100	1.02118500	4.49294400
C	-3.83041000	0.68997500	-2.98220700
C	-2.06164300	-0.40067700	-4.16040400
C	-0.94067700	-3.37134900	-3.57463000
C	-0.05888100	-5.15680300	2.00975100
C	-4.14460800	2.62272700	0.19003300
C	-2.63550500	3.25827400	1.93510300
C	-1.68040500	1.26720500	4.32092600
C	-0.13517400	-4.36622700	3.19767100
C	-3.10011100	1.87567600	-2.89391100
H	-4.82887900	0.66686600	-2.56317800
C	-1.27686700	0.74443800	-4.05389000
O	-1.58049400	-1.47622700	-4.91437200
C	-0.57511700	-2.23382800	-4.37899100
C	-3.32410300	3.18880500	-0.78524500
H	-5.06397900	2.14300300	-0.12294400
C	-1.78206700	3.83397200	0.99081300
O	-2.30930300	3.35542400	3.28869700
C	-1.30581300	2.52675500	3.75102400
C	-3.67303100	3.13965200	-2.26528100
C	-1.80409600	1.86711300	-3.42456400
H	-0.28955500	0.77399100	-4.49812500
C	1.25863500	-3.78888100	-3.17153400
C	-2.13284700	3.78627300	-0.35587400
H	-0.86305900	4.31155400	1.30674200
C	0.51045900	0.69368600	4.51785300
H	-3.15769000	3.98092400	-2.73498800
O	-1.05145000	3.03522500	-3.45607600
C	1.62213600	-2.75397500	-4.08709900
O	-1.31179900	4.41994900	-1.29068100
C	0.88994400	1.99563000	4.06398100
C	1.02760900	-4.17532700	3.97858000
C	1.17471700	-5.73647500	1.63585400
C	2.98308900	-2.56914400	-4.42207500
C	2.26911000	-4.55340000	-2.54608400
C	2.26073800	2.31665100	3.96151700
C	1.50513000	-0.27339500	4.78753600
C	-5.17209200	3.32318100	-2.53928300
H	-5.51350400	4.27319700	-2.11985100
H	-5.78811300	2.53275700	-2.10290200
C	-5.67582200	-1.43316000	-4.02855900
H	-5.75680100	-0.86449300	-4.95894300
H	-6.17852600	-0.85809600	-3.24611400
C	-6.89718000	-2.63517800	0.82947200
H	-7.33036900	-3.45616900	0.25142200
H	-7.05161100	-1.71447100	0.25961500
C	-6.25463300	2.28221500	2.28580200
H	-6.87486400	1.85818400	3.08015000
H	-6.60358500	1.86022200	1.33892000
H	-6.42591700	3.36161500	2.24895400
H	-7.45262500	-2.53708400	1.76603200
H	-6.22236000	-2.37278200	-4.14605100
H	-5.35922100	3.32879700	-3.61647200
C	2.83410600	0.04943000	4.61560300
C	3.21209000	1.35236900	4.21219900
H	4.25780900	1.58991100	4.05753600
H	3.60188100	-0.69958700	4.77666700
H	2.54597900	3.30596100	3.64114400
H	1.18197100	-1.25818300	5.10484000
C	2.29466600	-5.53432400	2.41436700
H	1.20517600	-6.33071800	0.72877900
C	2.21967200	-4.75035300	3.58949700
H	0.94311500	-3.57899800	4.88012700
C	3.59052900	-4.35074900	-2.88623600
H	1.97277000	-5.30795500	-1.82600400
C	3.94687800	-3.37029800	-3.84222100
H	3.23412900	-1.79343200	-5.13747300
H	4.99136600	-3.23438100	-4.10419200
H	4.36233000	-4.94748600	-2.41603300
H	3.11365000	-4.59731600	4.18549600
H	3.24716900	-5.95177500	2.11063200
P	-0.17011200	3.62692400	-2.19353900
N	0.59049200	4.85787500	-2.98451400
C	1.92877200	5.31337100	-2.59942000
C	-0.19859800	5.84378000	-3.72809000
H	2.47455800	4.53677200	-2.06550100
H	1.87899300	6.19657400	-1.95254100
H	2.48387100	5.56932700	-3.50772900
H	-1.13291000	5.40133900	-4.07296100
H	0.37377000	6.16382800	-4.60440000
H	-0.42062600	6.72178700	-3.10892500
Au	1.13465900	2.27231000	-0.95285700
N	-0.06268400	2.89596000	3.65908200
N	-0.80654200	0.37298900	4.67348600
N	-1.32144700	-3.81054900	3.59543700
N	-1.16723200	-5.35290300	1.22884900
N	-0.05833400	-4.08801500	-2.94488600
N	0.66468600	-1.94349400	-4.64077300
C	3.42891600	0.22066500	-1.14286900
C	3.29288900	1.20693900	-2.04702700
H	2.84995500	0.97325700	-3.00815900
C	2.98865500	-1.21686400	-1.23807700
C	3.31821100	-1.81384700	0.14380000
H	3.54340300	-1.70631000	-2.03555000
H	1.92626300	-1.29217600	-1.47064400
C	4.00957000	-0.64679000	0.87238600
O	4.08836700	0.44162700	0.04271100
O	4.46074800	-0.64524700	1.98405300
H	3.76599000	2.16913200	-1.88589400
C	2.10077500	-2.33726300	0.93431500
H	2.39033100	-2.40984900	1.98614900
H	1.90029600	-3.35325400	0.59460900
C	4.40156700	-2.89996500	0.04656700
O	4.27583700	-4.04609700	0.41870800
O	5.50508000	-2.36094000	-0.49297000
C	6.83357200	-2.99946900	-0.37397900
C	0.87018000	-1.48170800	0.76847500
C	-0.25412900	-1.97983500	0.09797900
C	0.84717300	-0.15910200	1.23333700
C	-1.38117800	-1.17834400	-0.10970300
H	-0.24915600	-3.00274000	-0.26746400
C	-0.27401600	0.64691300	1.01565800
H	1.69068500	0.25589100	1.77382600
C	-1.39322200	0.13852400	0.34374400

H	-2.24441500	-1.58070400	-0.62471600
H	-0.28429100	1.65300500	1.41167200
H	-2.26362500	0.76319900	0.18797800
C	7.13572700	-3.22707200	1.10737600
H	6.98281400	-2.30055900	1.66747600
H	6.49385100	-4.00247400	1.52737900
H	8.17855000	-3.53647100	1.22277100
C	7.75718900	-1.94027500	-0.97015200
H	8.79308800	-2.28942900	-0.94293400
H	7.48577500	-1.73565700	-2.00968800
H	7.68572200	-1.00791500	-0.40442500
C	6.87425600	-4.29256800	-1.18571000
H	6.18572100	-5.03178900	-0.77585000
H	6.61641500	-4.09580400	-2.23037800
H	7.88795100	-4.70386300	-1.16102300
S	3.40453900	3.68435900	0.89504800
O	3.92278200	3.92370800	-0.46662800
O	2.38165000	2.56768600	0.96270000
O	4.36286600	3.65936100	2.00160400
C	2.35403600	5.19398000	1.22814800
F	3.05487500	6.30598700	0.98663400
F	1.92029000	5.21956900	2.49228800
F	1.26097500	5.21339700	0.42347700

(TfO•2b)⊂AuCav-2

C	1.19603500	5.64324400	1.18229300
C	0.86870700	5.08085200	-0.19518700
C	1.25539100	4.52197800	2.21379400
H	0.35360600	6.27579400	1.46292000
C	1.87243100	4.63728500	-1.05807700
C	-0.45322700	5.00567300	-0.65799800
C	2.41025300	3.77756800	2.46400100
C	0.11202400	4.19680700	2.95424600
C	1.62219900	4.16233300	-2.34731100
H	2.90078500	4.69644700	-0.72281100
C	-0.75956000	4.49413200	-1.91854900
O	-1.46778000	5.57145400	0.11524500
C	2.46351800	2.76515800	3.42936900
H	3.30451400	3.99328600	1.88962800
C	0.11426400	3.20781500	3.92752900
O	-1.03718700	4.95298000	2.72787800
C	2.76132900	3.77885800	-3.28647600
C	0.28151600	4.08038900	-2.74872400
H	-1.78470300	4.46967100	-2.26753100
C	-2.36464500	4.74822100	0.74367700
C	3.72760800	1.95244600	3.68046300
C	1.29681400	2.50996200	4.15946400
H	-0.78149000	2.97644400	4.48875800
C	-2.09946700	4.36501200	2.10400900
H	2.35707700	3.83338800	-4.29749300
C	3.16297600	2.32751800	-3.05934200
O	-0.00250900	3.69435700	-4.06149300
C	3.87033100	0.83465500	2.65198200
H	3.58791900	1.46089100	4.64388100
O	1.31993300	1.53331900	5.15715500
C	4.09845800	1.93657000	-2.09501000
C	2.55992100	1.31635900	-3.81654100
C	-0.53873400	2.45873400	-4.29910400
C	-4.32004200	3.58094800	0.80796000
C	4.57827600	1.00360700	1.45789800
C	3.29300900	-0.42458000	2.88956400
C	0.57685300	0.42162700	4.82896100
C	-3.99596700	3.10527500	2.11628800
C	4.44429500	0.59990200	-1.86968200
H	4.57499700	2.70356500	-1.49575100
C	2.86155300	-0.02570600	-3.62348900
O	1.67631000	1.69691200	-4.82298600
C	0.35092200	1.39490000	-4.68314700
C	4.76972600	-0.03082400	0.53621600
H	5.00276900	1.97663900	1.24141700
C	3.49255200	-1.49672300	2.02500800
O	2.59638200	-0.60647900	4.07646700
C	1.22265500	-0.64231700	4.11385200
C	5.46379200	0.18733000	-0.80606600
C	3.79737000	-0.36843200	-2.65274900
H	2.37554000	-0.78825900	-4.21841000
C	-2.31128300	1.05187000	-4.57132300
C	4.24734200	-1.28552700	0.87335600
H	3.05461800	-2.46847400	2.21700700
C	-1.40613800	-0.67780400	4.68440900
H	5.85685400	-0.78333900	-1.11660000
O	4.09073300	-1.71438300	-2.48984400
C	-1.42370100	-0.01463700	-4.91117500
O	4.46629000	-2.36823800	0.00774600
C	-0.79524700	-1.66603500	3.85197500
C	-4.86372800	2.19520200	2.76287700
C	-5.53559200	3.18142400	0.20360400
C	-1.94777300	-1.28727500	-5.22911900
C	-3.70635300	0.82243500	-4.58475200
C	-1.59861200	-2.69007700	3.30179400
C	-2.77666500	-0.78115900	5.01239600
C	6.65498900	1.15155600	-0.71958400
H	7.38065600	0.78396300	0.01073900
H	6.36738500	2.16195000	-0.41659200
C	3.94972500	4.74826200	-3.21036400
H	4.70634300	4.46880400	-3.94865700
H	4.43505100	4.75779400	-2.23057500
C	2.45560400	6.52168900	1.18893200
H	2.35398000	7.33285800	0.46266300
H	3.36645600	5.96923500	0.94180000
C	4.98074900	2.83437000	3.78450100
H	4.85928500	3.55587900	4.59686100
H	5.18166900	3.40247100	2.87159000
H	5.86101300	2.21989500	3.99313700
H	2.59819200	6.95805200	2.18116600
H	3.61654400	5.76781600	-3.42178800
H	7.14716700	1.22812500	-1.69278200
C	-3.52520100	-1.81774900	4.49957400
C	-2.93581500	-2.76234600	3.62804300
H	-3.54630700	-3.55270600	3.20388000
H	-4.57900800	-1.90122400	4.74795800
H	-1.14355200	-3.38139200	2.61319900
H	-3.20341700	-0.02234600	5.65969700
C	-6.37594200	2.31298100	0.86573000
H	-5.76041300	3.55455100	-0.78960600
C	-6.03257800	1.81088800	2.14376900
H	-4.57016800	1.81274100	3.73361900
C	-4.19305000	-0.42694600	-4.90798300
H	-4.36123900	1.65371800	-4.34600600
C	-3.31076800	-1.48572600	-5.22795400
H	-1.25079500	-2.08334900	-5.46403800
H	-3.71589400	-2.46240100	-5.47126800
H	-5.26311200	-0.60624800	-4.91426900
H	-6.69402000	1.10071200	2.62776300
H	-7.29081600	1.97579300	0.39066800
P	3.40161200	-2.55462000	-1.22305700
N	3.72463300	-4.08531500	-1.73970200
C	2.99306100	-4.54848100	-2.92364700
C	4.27868600	-5.14910600	-0.90042000
H	2.61838700	-3.69667100	-3.49625200
H	2.15073000	-5.17895400	-2.62527100
H	3.67904200	-5.10762100	-3.56876300
H	4.70476900	-4.72814300	0.00632700
H	5.06470400	-5.66107000	-1.46822100
H	3.49497600	-5.85868100	-0.62445300
Au	1.43022400	-1.42142700	-0.57964700
N	0.55496300	-1.63549000	3.60992900
N	-0.68451600	0.39205100	5.13348000
N	-2.86796200	3.54158800	2.75383200
N	-3.44719200	4.38275600	0.12099500
N	-1.82805700	2.28998300	-4.24182600
N	-0.07329800	0.19566200	-4.95542900
C	-0.11311500	0.52347900	-0.03205400
C	1.22207100	0.81058200	-0.08128300
H	1.62440200	1.27286300	-0.97447700
C	-1.15876600	0.68911800	-1.09920500
C	-2.34327100	-0.12662100	-0.55521500
H	-0.83541100	0.33730300	-2.07644900
H	-1.42196100	1.75158000	-1.16868300
C	-2.07554600	-0.06662500	0.95126000

O	-0.73299100	0.24003400	1.16201400
O	-2.82146500	-0.15411100	1.88066200
C	-3.69310300	0.54286200	-0.93684200
H	-3.73863700	1.48083600	-0.37834600
H	-3.59697800	0.82073500	-1.98849800
C	-2.28659700	-1.55637900	-1.15377600
O	-1.74504600	-1.75952200	-2.22286400
O	-2.96027000	-2.42268100	-0.42075900
C	-3.41212600	-3.73802600	-0.97005200
H	1.79660800	0.89552800	0.83469900
C	-5.01215300	-0.18378800	-0.76468300
C	-5.96112400	-0.05341800	-1.78538000
C	-5.37638300	-0.88674200	0.39235100
C	-7.23650100	-0.60895400	-1.66915400
H	-5.70007200	0.49812400	-2.68141300
C	-6.64682300	-1.44792300	0.50875600
H	-4.66819000	-0.98596600	1.20196000
C	-7.58457300	-1.31411200	-0.51807800
H	-7.95230000	-0.49203000	-2.47766000
H	-6.90693400	-1.99114900	1.41297900
H	-8.57267100	-1.75375400	-0.42075000
C	-4.00074200	-4.41144500	0.26551100
H	-3.21597000	-4.59773000	1.00103000
H	-4.77486900	-3.78316900	0.71208800
H	-4.44573200	-5.36973500	-0.01684700
C	-2.25554000	-4.55283700	-1.53758800
H	-2.61674500	-5.56849600	-1.73026900
H	-1.88128300	-4.12866700	-2.46773100
H	-1.43238800	-4.61055600	-0.83221600
C	-4.48098700	-3.44949600	-2.02362000
H	-4.06457200	-2.85774400	-2.84160800
H	-4.83471200	-4.39921600	-2.43697000
H	-5.33108200	-2.91746300	-1.59464000
S	0.96975000	-4.46182300	0.56918200
O	0.90572900	-5.27578900	-0.65961700
O	2.32791700	-4.18753900	1.08733800
O	0.06856100	-3.26114800	0.55710900
C	0.23066100	-5.56849400	1.87013900
F	-1.09762400	-5.69925100	1.67952700
F	0.43401000	-5.07182500	3.09498400
F	0.78842600	-6.78060000	1.80069500

(TfO•3b)⊂AuCav-1

C	-3.56188000	-4.27674900	1.93610500
C	-2.46510700	-3.58613300	2.73770700
C	-3.60648500	-3.72328900	0.51654600
H	-4.50642800	-3.99887500	2.40410700
C	-1.13793200	-4.02599100	2.73553900
C	-2.76689900	-2.45995100	3.51411000
C	-2.81234700	-4.25174200	-0.50308500
C	-4.45926100	-2.66197400	0.17989800
C	-0.13153100	-3.40395000	3.48173000
H	-0.88103900	-4.89547500	2.14110400
C	-1.80089700	-1.79689200	4.26228000
O	-4.09795200	-2.04996800	3.58465800
C	-2.86459000	-3.79606000	-1.82324700
H	-2.14388200	-5.07092800	-0.26512200
C	-4.53322900	-2.15884200	-1.11515600
O	-5.32258600	-2.17350200	1.16427100
C	1.30798700	-3.90966100	3.50247700
C	-0.49678700	-2.28226100	4.23669900
H	-2.05761700	-0.93237500	4.86000500
C	-4.44145300	-0.87147400	2.98292400
C	-2.01282000	-4.42796600	-2.91888900
C	-3.73949500	-2.73756600	-2.10263900
H	-5.19636500	-1.33708800	-1.35320100
C	-5.08815600	-0.93669300	1.69858800
H	1.76210800	-3.49952600	4.40475900
C	2.10373500	-3.34334600	2.33095100
O	0.47910800	-1.66865700	5.02260800
C	-0.62635000	-3.79422000	-2.94839900
H	-2.48837100	-4.16749900	-3.86468200
O	-3.87050000	-2.31257500	-3.42564100
C	2.13477900	-3.97452200	1.08410800
C	2.84627200	-2.15995800	2.47155200
C	0.90433400	-0.44225900	4.58392000
C	-4.64609600	1.39619600	2.97023100
C	0.44026600	-4.26305900	-2.17365100
C	-0.38347800	-2.68971000	-3.77686200
C	-3.34562100	-1.08919500	-3.75481400
C	-5.28315900	1.33273200	1.69269000
C	2.87530000	-3.49027600	0.00172200
H	1.56562100	-4.88702400	0.95316600
C	3.59698700	-1.63412000	1.42262500
O	2.86379100	-1.53770600	3.71931400
C	2.15301000	-0.37247600	3.87610100
C	1.71568900	-3.68894200	-2.21349200
H	0.27397300	-5.11437000	-1.52379500
C	0.86712500	-2.08653200	-3.84951600
O	-1.41830000	-2.24733000	-4.58922900
C	-2.02401300	-1.04742600	-4.31715200
C	2.87252200	-4.19215000	-1.35376300
C	3.60038900	-2.30770800	0.20355200
H	4.14225200	-0.71032700	1.54579000
C	0.63979300	1.80673900	4.34285700
C	1.90278600	-2.59688300	-3.07182100
H	1.02516400	-1.23473900	-4.49840000
C	-3.49393200	1.16978000	-3.96549300
H	3.79017400	-3.88939100	-1.86296000
O	4.34853900	-1.76377600	-0.85252100
C	1.87994100	1.87859300	3.63351600
O	3.14679000	-1.97859800	-3.15468800
C	-2.15736000	1.21755000	-4.46544200
C	-5.67493400	2.53032000	1.05151400
C	-4.43552100	2.65358800	3.58396300
C	2.32282200	3.11802200	3.11864100
C	-0.13094700	2.97694300	4.52579000
C	-1.58982900	2.46258200	-4.81975000
C	-4.24029500	2.36550200	-3.85617000
C	2.90650600	-5.72258600	-1.23372700
H	2.96681400	-6.17368900	-2.22770500
H	2.02186800	-6.13326300	-0.73983100
C	1.39311000	-5.44059600	3.59464100
H	2.43820200	-5.75643200	3.65494800
H	0.94380600	-5.94800000	2.73628000
C	-3.45719000	-5.80818000	1.97933900
H	-3.49482200	-6.15584700	3.01533200
H	-2.53106600	-6.18874900	1.53948500
C	-1.96642900	-5.96026000	-2.82877000
H	-2.98017700	-6.36744800	-2.86955300
H	-1.50466700	-6.32319400	-1.90620500
H	-1.39335200	-6.36816600	-3.66604500
H	-4.28993700	-6.25656400	1.43080300
H	0.87177900	-5.78908300	4.49018300
H	3.78026900	-6.03386600	-0.65520000
C	-3.67194200	3.56286000	-4.23313000
C	-2.33922200	3.61352400	-4.70798800
H	-1.90830400	4.56985700	-4.98615700
H	-4.24885600	4.47951300	-4.16947400
H	-0.56735100	2.47521300	-5.17963400
H	-5.25404400	2.29976300	-3.47715300
C	-4.83235400	3.80561300	2.93799800
H	-3.94994400	2.67281500	4.55371300
C	-5.44893500	3.74355700	1.66538300
H	-6.12581200	2.45619800	0.06888100
C	0.32223200	4.17636200	4.01473200
H	-1.06899900	2.89375900	5.06412100
C	1.54596100	4.24317800	3.30692700
H	3.25373400	3.15961700	2.56426700
H	1.88052000	5.19203000	2.90121200
H	-0.26496700	5.07879800	4.15727000
H	-5.72158500	4.66394000	1.16077700
H	-4.65694800	4.77301400	3.39704700
P	3.54994600	-0.86572800	-1.97177400
N	4.74002300	0.01927500	-2.63849900
C	6.17688400	-0.19614000	-2.46224600
C	4.35790100	1.07114400	-3.58153600
H	6.35127600	-0.94391300	-1.69229500
H	6.62534200	0.74755000	-2.14518300
H	6.62309300	-0.53382000	-3.40551800

H	3.27308400	1.20425900	-3.57848700
H	4.67168200	0.80637600	-4.59844500
H	4.81741500	2.00815900	-3.26189400
Au	1.57591900	-0.17614400	-0.97034100
N	-1.43943200	0.06615900	-4.64239400
N	-4.06261900	-0.01830500	-3.59841200
N	-5.49621600	0.12929500	1.07679700
N	-4.22341400	0.25481500	3.59607800
N	0.17197300	0.60858700	4.80772300
N	2.62273800	0.74775300	3.41882600
C	-0.01846700	-0.35112100	0.68090500
C	-0.78253400	-0.94621500	-0.28961700
H	-0.66505800	-1.97055400	-0.62676200
C	-0.43958200	0.97593500	1.24179900
C	-1.26310200	1.81868800	0.23472600
H	0.42284800	1.56496300	1.55561500
H	-1.05045300	0.79984900	2.13726200
C	-2.26760400	0.93316800	-0.50501400
O	-1.85702700	-0.35817400	-0.85846600
O	-3.37813200	1.24179100	-0.82670900
H	0.62203700	-0.99844300	1.27520900
C	-1.97715500	2.93375300	1.04619800
H	-2.89080700	2.49352500	1.44509200
H	-1.33678000	3.14763300	1.90358300
C	-0.34333300	2.38618900	-0.86256200
O	-0.33380800	1.91624300	-1.99158700
O	0.38212800	3.38530400	-0.40981800
C	1.17372900	4.26824000	-1.32967900
C	-2.30567100	4.24945900	0.37868500
C	-3.10901600	4.34983800	-0.76369400
C	-1.83232700	5.42794400	0.96963000
C	-3.43146200	5.60074600	-1.29042900
H	-3.48776700	3.45227800	-1.23435800
C	-2.15816300	6.67910800	0.44728000
H	-1.19557900	5.35730800	1.84606500
C	-2.96403500	6.76978700	-0.68851600
H	-4.05652500	5.66071500	-2.17548800
H	-1.77893800	7.57906300	0.92241500
H	-3.22118700	7.74032800	-1.10199700
C	1.89955900	5.18446700	-0.35260200
H	2.44629500	5.94692400	-0.91485300
H	1.17638800	5.68450900	0.29755400
H	2.61879900	4.62765200	0.25113400
C	2.15112100	3.43949600	-2.15732500
H	2.92122800	4.10158000	-2.56322300
H	2.64980500	2.70171500	-1.53000900
H	1.64380600	2.93168400	-2.97768400
C	0.17835400	5.03538500	-2.19578000
H	0.73653600	5.69838300	-2.86443000
H	-0.41750200	4.35545800	-2.80636000
H	-0.48980100	5.64226400	-1.58293900
S	4.76305100	2.35772000	0.21478100
O	3.58987300	1.43170500	0.21416600
O	5.40934200	2.54881900	-1.09835700
O	4.56514000	3.58820100	1.01156800
C	6.01576300	1.40456900	1.19799600
F	5.57204700	1.18480300	2.44477000
F	7.17879800	2.06006100	1.26425500
F	6.24417700	0.19997500	0.62784300

(TfO•3b)⊂AuCav-2

C	5.91455600	-0.11391400	1.98865700
C	5.45046600	0.88883000	0.93512600
C	4.82068100	-1.10744200	2.37177000
H	6.11761500	0.46856100	2.88817900
C	5.48809300	0.60715200	-0.43496400
C	4.98069700	2.15542600	1.30919500
C	4.61560600	-2.30999200	1.68331500
C	3.98870100	-0.85228000	3.47079600
C	5.10423500	1.53288100	-1.41031900
H	5.81899300	-0.37552900	-0.75286600
C	4.61685300	3.11825000	0.37750300
O	4.86951900	2.46175600	2.67082800
C	3.64457500	-3.24327900	2.06215500
H	5.24103000	-2.52855100	0.82512800
C	3.02380500	-1.75534000	3.89782600
O	4.15572800	0.34166500	4.17895700
C	5.05707800	1.19312300	-2.89563900
C	4.68833900	2.79397100	-0.97114000
H	4.25718100	4.08780300	0.69384800
C	3.58282200	2.42597100	3.14245900
C	3.38616000	-4.54494300	1.30880200
C	2.87029400	-2.93814800	3.18686200
H	2.39716600	-1.53554600	4.75059800
C	3.20182800	1.29601000	3.94286900
H	5.03140300	2.14175200	-3.43256700
C	3.73926800	0.48782700	-3.19670500
O	4.34629200	3.74738100	-1.92685000
C	2.37864800	-4.32058100	0.18392600
H	2.90770200	-5.22108200	2.01771500
O	1.93649700	-3.88382900	3.60485700
C	3.62661500	-0.90389100	-3.15134100
C	2.58537100	1.21760300	-3.52470600
C	3.03134300	4.13704000	-1.93924400
C	1.46543000	3.25093900	3.30366700
C	2.79204300	-3.86020600	-1.06926800
C	1.00571000	-4.56239200	0.36104800
C	0.62041000	-3.54411100	3.46421100
C	1.08998400	2.13150500	4.10941200
C	2.44262900	-1.58754500	-3.43641200
H	4.51227800	-1.48262900	-2.92093600
C	1.37238700	0.57897300	-3.77477100
O	2.70105000	2.58566100	-3.73943000
C	2.15220700	3.50846300	-2.88143800
C	1.91440900	-3.66016500	-2.13623600
H	3.84331300	-3.64368400	-1.21801800
C	0.09123500	-4.38033400	-0.67700100
O	0.55143500	-5.04833400	1.58598100
C	-0.12254100	-4.17616600	2.41024400
C	2.37892200	-3.11136800	-3.47836600
C	1.31118300	-0.81207000	-3.72666000
H	0.50677700	1.16087700	-4.06184200
C	1.32605900	5.45673200	-1.22018500
C	0.56421000	-3.94028300	-1.91012800
H	-0.96386500	-4.57348300	-0.52190300
C	-1.25459600	-2.44036700	4.13082500
H	1.60833200	-3.36896500	-4.20863300
O	0.12507600	-1.42410500	-4.12593600
C	0.45511500	4.85872700	-2.18234600
O	-0.35440100	-3.73485600	-2.95053000
C	-2.01182600	-3.11449900	3.12290900
C	-0.23967800	2.02690900	4.57634100
C	0.49978800	4.22653500	2.96896600
C	-0.88290700	5.30475000	-2.27036900
C	0.83446100	6.46218700	-0.35675400
C	-3.40440200	-2.89058300	3.03197100
C	-1.89583300	-1.52628100	4.99856500
C	3.68401100	-3.76119800	-3.96063700
H	3.54648100	-4.84159000	-4.05485000
H	4.52200200	-3.59714900	-3.27817400
C	6.29262900	0.41847300	-3.37303000
H	6.21702200	0.20331100	-4.44264000
H	6.43120800	-0.53332900	-2.85226500
C	7.22339500	-0.81142900	1.58551900
H	8.00139000	-0.06651200	1.39788200
H	7.12296300	-1.41559200	0.67941700
C	4.67659000	-5.22015400	0.82161000
H	5.33316600	-5.42430100	1.67169200
H	5.24024500	-4.60777800	0.11214700
H	4.44321900	-6.16689700	0.32684400
H	7.55982500	-1.47358100	2.38809600
H	7.19199800	1.01587800	-3.20149000
H	3.96784600	-3.35862300	-4.93661500
C	-3.25209600	-1.31168100	4.87723600
C	-4.00577800	-2.00232100	3.89834400
H	-5.07421800	-1.82978900	3.82602800
H	-3.75115600	-0.62074000	5.54991800
H	-3.96710500	-3.41187600	2.26803500
H	-1.29591800	-1.04039700	5.76049700
C	-0.79310700	4.09942500	3.43255100

H	0.79425000	5.03944600	2.31566800
C	-1.16087400	2.99996400	4.24352400
H	-0.50481100	1.16588100	5.17930900
C	-0.48066400	6.86200500	-0.45172600
H	1.52195700	6.89662000	0.36137400
C	-1.33846800	6.28376500	-1.41592300
H	-1.53311100	4.84627300	-3.00119000
H	-2.37348900	6.60462700	-1.47293600
H	-0.86412900	7.62820800	0.21493300
H	-2.18486800	2.91128400	4.58884100
H	-1.54377600	4.82444600	3.14078000
P	-0.92951000	-2.20819700	-3.11828400
N	-2.18754100	-2.43530100	-4.14684700
C	-2.71278200	-3.74239900	-4.54673800
C	-3.09971900	-1.30993200	-4.35524000
H	-1.97477700	-4.51943500	-4.36015500
H	-2.93139500	-3.71455100	-5.62008000
H	-3.62097300	-3.96843200	-3.98241400
H	-2.66939000	-0.38084800	-3.97517800
H	-4.04456300	-1.48765900	-3.83617800
H	-3.27982700	-1.18070300	-5.42786100
Au	-0.98699400	-1.25512000	-1.00772200
N	-1.39588400	-3.97334500	2.24876000
N	0.08096500	-2.68830700	4.28075300
N	1.99770800	1.15211100	4.41170700
N	2.74434200	3.36850500	2.83046400
N	2.63257100	5.07209300	-1.12940500
N	0.90623300	3.86187400	-3.00766300
C	-0.63955700	-0.28872900	1.12127000
C	0.32822300	0.36864700	0.41049800
H	1.30598000	-0.03761700	0.17528200
C	-1.82942500	0.46531200	1.64882000
C	-2.15908500	1.71600300	0.79080700
H	-1.60361600	0.76905000	2.67552400
H	-2.69965700	-0.18795500	1.69026700
C	-0.90047100	2.45878300	0.36512000
O	0.22779400	1.67145500	0.04431500
O	-0.76958300	3.63429300	0.21910800
H	-0.37710500	-1.23523600	1.58464500
C	-2.97442200	1.25477300	-0.47850500
H	-2.59596100	0.27550900	-0.77334000
H	-3.98397100	1.05668200	-0.12085600
C	-3.15082600	2.65347300	1.50508500
O	-3.29488700	3.83352300	1.28134400
O	-3.93520800	1.90955700	2.30452500
C	-5.24883900	2.39781900	2.77014500
C	-3.01026900	2.11662900	-1.71041400
C	-2.05306500	1.92610200	-2.71540200
C	-4.06074100	3.01044800	-1.94436100
C	-2.16767000	2.57032500	-3.94595700
H	-1.22131300	1.25123700	-2.53488400
C	-4.17501400	3.66531800	-3.17057800
H	-4.80577000	3.17093200	-1.17244600
C	-3.24039500	3.43219600	-4.18204100
H	-1.41585200	2.41359300	-4.71234400
H	-5.00480700	4.34406100	-3.34343900
H	-3.33943900	3.92916600	-5.14218900
C	-5.82389500	1.16807200	3.46797300
H	-5.18040400	0.86656300	4.29785700
H	-6.82051400	1.39011800	3.85940900
H	-5.89652900	0.33437600	2.76522400
C	-6.10665000	2.78117200	1.56291100
H	-5.69920900	3.65155600	1.04774100
H	-6.17623000	1.94049600	0.86631800
H	-7.11840500	3.02165200	1.90229100
C	-5.06186400	3.55938100	3.74485400
H	-4.43569000	3.25346000	4.58822100
H	-4.60333800	4.41487800	3.24923900
H	-6.03558600	3.86039300	4.14340600
S	-4.19359300	-2.68567500	-0.49084600
O	-3.77696100	-3.20478500	-1.80685600
O	-4.93257300	-3.60231700	0.38868100
O	-3.12474100	-1.88805100	0.19677100
C	-5.45110400	-1.37272600	-0.89408600
F	-4.97956000	-0.51829200	-1.82754700
F	-5.74503300	-0.64410900	0.20641300
F	-6.57963000	-1.91123800	-1.35765200

1b⊂AuCav

C	1.25963900	4.34133200	-2.91602300
C	1.66630500	2.90793300	-3.23805200
C	0.55140500	4.37745100	-1.56584500
H	0.51791300	4.62739600	-3.66215100
C	2.92644300	2.37889700	-2.93989200
C	0.73903500	2.06075700	-3.86366000
C	1.28197400	4.43738200	-0.37660300
C	-0.84640300	4.35892600	-1.45863900
C	3.28775600	1.06414500	-3.26082300
H	3.66149600	3.02255700	-2.47118300
C	1.04774600	0.74785700	-4.20413600
O	-0.45101500	2.63693900	-4.26012500
C	0.68884800	4.52225000	0.88457600
H	2.36406600	4.45963600	-0.43952200
C	-1.48359900	4.38963600	-0.22017900
O	-1.62037900	4.42374200	-2.61937400
C	4.66572300	0.48083700	-2.94520300
C	2.31971400	0.27553900	-3.89489600
H	0.33253100	0.12600800	-4.72622600
C	-1.67922700	2.30626000	-3.76659300
C	1.54036900	4.66949300	2.14328900
C	-0.71288600	4.48138000	0.93585600
H	-2.56463800	4.37930000	-0.15879500
C	-2.33795300	3.31451300	-2.97861100
H	4.84970000	-0.29725900	-3.68667900
C	4.63618000	-0.21602800	-1.58611000
O	2.70913700	-0.99885100	-4.30258700
C	1.98274700	3.29184800	2.61954600
H	0.88282600	5.06882400	2.91576900
O	-1.35188400	4.64586400	2.16692100
C	4.78417500	0.49773200	-0.39077400
C	4.41955800	-1.59803000	-1.48100800
C	2.17164500	-2.10059900	-3.72143500
C	-3.58132600	1.06505200	-3.77353100
C	3.16175600	2.67243100	2.19467300
C	1.15652000	2.57981100	3.50111300
C	-1.96678300	3.55820800	2.72848000
C	-4.25770500	2.10352600	-3.06300200
C	4.68865900	-0.08993900	0.87664300
H	4.96377600	1.56494600	-0.44891500
C	4.30872600	-2.22885100	-0.24576900
O	4.29806000	-2.37010500	-2.63453600
C	3.04629700	-2.87457200	-2.87947000
C	3.52872600	1.38089900	2.59767700
H	3.82462200	3.21829700	1.53380600
C	1.46574000	1.29094900	3.91676800
O	0.03773600	3.22356100	3.98963100
C	-1.21109600	2.76580100	3.65757600
C	4.83890400	0.71817200	2.16622200
C	4.42187700	-1.46464800	0.90653500
H	4.09951900	-3.28996500	-0.19250200
C	0.53273100	-3.65396800	-3.43439100
C	2.63990900	0.71439500	3.45020700
H	0.80372000	0.75802200	4.58719900
C	-3.76356400	2.20698100	3.07078300
H	5.08998900	-0.00031400	2.95067400
O	4.13167000	-2.10896200	2.12992300
C	1.42071400	-4.45034600	-2.64785800
O	2.89654400	-0.61044100	3.84962200
C	-2.98551400	1.37721600	3.93682500
C	-5.63214500	1.96164800	-2.76742300
C	-4.29555200	-0.09097500	-4.16256300
C	0.99172200	-5.71121900	-2.17494200
C	-0.77701800	-4.11987800	-3.69439200
C	-3.56708700	0.22658200	4.51401100
C	-5.11937500	1.88562700	2.84103200
C	6.00535900	1.71588400	2.08704700
H	6.12320900	2.22980700	3.04410500
H	5.86099400	2.47851600	1.31770900
C	5.80053800	1.50852000	-3.05815800
H	6.76430300	1.02143600	-2.88915900

H	5.71486600	2.32615600	-2.33699500
C	2.42110800	5.34013900	-3.01046600
H	2.86735100	5.30607300	-4.00807100
H	3.21686900	5.14835500	-2.28492100
C	2.70503100	5.65404200	1.96855900
H	2.32217100	6.63406800	1.67370600
H	3.42412300	5.34184500	1.20579000
H	3.24903300	5.76395400	2.91068500
H	2.05364500	6.35329300	-2.83002600
H	5.80659700	1.95040400	-4.05765800
H	6.93411100	1.18783800	1.85734900
C	-5.67136000	0.77211000	3.43845500
C	-4.89089300	-0.06898100	4.26444500
H	-5.34570100	-0.94658200	4.71238300
H	-6.71308400	0.52730200	3.26920600
H	-2.95318400	-0.38788100	5.16408900
H	-5.69404200	2.53482300	2.18965500
C	-5.63603700	-0.20799600	-3.85452800
H	-3.76499800	-0.85943600	-4.71530000
C	-6.30667700	0.82265300	-3.15602000
H	-6.12236600	2.76643100	-2.23060800
C	-1.17132600	-5.35526200	-3.22255600
H	-1.43222200	-3.49722700	-4.29421300
C	-0.27976100	-6.15846600	-2.47200400
H	1.69267600	-6.31060400	-1.60400500
H	-0.60070400	-7.13763500	-2.13194900
H	-2.16754000	-5.72532900	-3.44223200
H	-7.36049100	0.71060200	-2.92371400
H	-6.18576600	-1.09382600	-4.15559000
P	2.64372000	-1.77440800	2.72070600
N	2.25635900	-3.14417700	3.55406200
C	3.28590700	-4.01508600	4.14267100
C	0.99972300	-3.06684000	4.31569700
H	4.15843400	-4.05004000	3.49222400
H	2.87042700	-5.02235200	4.23156000
H	3.58706200	-3.66411500	5.13684800
H	0.26947100	-2.45464800	3.77964300
H	1.16011200	-2.64061800	5.31301800
H	0.58843200	-4.07436500	4.41674600
Au	1.38361000	-0.88727300	0.96773800
N	-1.68549600	1.69731300	4.22278700
N	-3.20876200	3.29587000	2.45563400
N	-3.59061300	3.22517000	-2.65446600
N	-2.26174700	1.19914500	-4.12061600
N	0.94370400	-2.45705900	-3.95944800
N	2.68366700	-4.00501800	-2.35327300
C	0.18787100	-0.39908800	-0.99630200
C	1.03768300	0.46228700	-0.77220200
H	1.68607700	1.31516000	-0.83545200
C	-0.96681900	-1.19768700	-1.39902000
C	-2.14386100	-1.16450100	-0.35993600
H	-0.68998000	-2.23171100	-1.61149100
H	-1.32573700	-0.74771000	-2.33276100
C	-2.25697700	0.32040300	0.08169900
O	-1.83453900	0.65323600	1.29719400
O	-2.63708100	1.14877600	-0.71377900
H	-1.58401200	-0.16688000	1.79253100
C	-3.42489000	-1.59639800	-1.11860800
H	-3.56371400	-0.87095200	-1.91850700
H	-3.22564100	-2.56680600	-1.57950300
C	-1.80172900	-2.14685500	0.76612000
O	-1.34512100	-1.80179800	1.85922300
O	-1.99315100	-3.39365600	0.39760400
C	-1.69243400	-4.56254600	1.28437100
C	-4.68009500	-1.66184900	-0.28127600
C	-5.13348400	-2.87780600	0.24654400
C	-5.44554600	-0.50774000	-0.07150200
C	-6.32209200	-2.94197900	0.97384700
H	-4.56403800	-3.78138000	0.05893700
C	-6.64443500	-0.57558100	0.63589100
H	-5.11298900	0.43622000	-0.48644000
C	-7.08450700	-1.78892000	1.16606000
H	-6.66455200	-3.89403000	1.36821700
H	-7.23977400	0.32278600	0.76476600
H	-8.02103300	-1.83950600	1.71297900
C	-2.36391100	-4.39213800	2.64595200
H	-1.87344500	-3.64121300	3.26157100
H	-3.41205600	-4.10846300	2.51709900
H	-2.33459200	-5.35326200	3.16761600
C	-0.17391100	-4.69691400	1.35174200
H	0.08903800	-5.56581500	1.96226700
H	0.23928800	-4.84715300	0.35102300
H	0.28878800	-3.81791100	1.79837000
C	-2.31836500	-5.72446300	0.51983800
H	-1.95405800	-5.74502200	-0.50773300
H	-2.04973300	-6.66641000	1.00540500
H	-3.40824400	-5.64620500	0.50890800

1b-TS1$_{5anti}$H^{+}$\subset$AuCav

C	-3.22484400	4.27569200	1.87333600
C	-2.34586900	3.34112100	2.70031500
C	-3.27000600	3.83168700	0.41543700
H	-2.72907700	5.24672600	1.88217400
C	-2.85068600	2.20060100	3.32867000
C	-0.98475200	3.62008700	2.88382100
C	-4.18376600	2.87805400	-0.04490100
C	-2.38426900	4.37439700	-0.52386700
C	-2.07269500	1.37644600	4.14719700
H	-3.89841500	1.95790700	3.19608200
C	-0.16471200	2.82785400	3.67983100
O	-0.47903400	4.79307600	2.32634900
C	-4.23445400	2.45511100	-1.37644400
H	-4.88529600	2.45126700	0.66248600
C	-2.39865800	3.99281700	-1.86124200
O	-1.49529200	5.36667500	-0.10741500
C	-2.64752300	0.13837700	4.82796600
C	-0.72668300	1.72237300	4.30931500
H	0.87640700	3.08382300	3.82788800
C	0.36848200	4.71916600	1.26162400
C	-5.24109400	1.42198100	-1.87379200
C	-3.31012100	3.02303900	-2.26211000
H	-1.70606600	4.43051900	-2.56778500
C	-0.16961800	5.04465600	-0.03454300
H	-1.98778800	-0.09387900	5.66409500
C	-2.56546600	-1.04080700	3.86578600
O	0.04705400	0.97222800	5.20512900
C	-4.71294000	0.00768700	-1.65830000
H	-5.31566900	1.55956000	-2.95268500
O	-3.34476900	2.61208300	-3.59743400
C	-3.57871000	-1.32524000	2.94761400
C	-1.43763800	-1.87172200	3.85035600
C	1.04716000	0.20484600	4.69495000
C	2.42930000	4.46376200	0.33584500
C	-4.89697400	-0.67201100	-0.45052200
C	-4.02253000	-0.66712900	-2.67557200
C	-2.32193000	1.81182500	-4.01851500
C	1.89909000	4.79624800	-0.94744900
C	-3.51674600	-2.39313600	2.04779300
H	-4.46300300	-0.70055500	2.94631000
C	-1.31329500	-2.93203300	2.95733800
O	-0.45745300	-1.65665800	4.81380000
C	0.76638600	-1.18722700	4.44082500
C	-4.44702100	-1.97713500	-0.23396200
H	-5.42502800	-0.16779400	0.34963100
C	-3.54848300	-1.96599100	-2.50526200
O	-3.85661100	-0.03728200	-3.90700000
C	-2.59924100	0.41420400	-4.21297300
C	-4.66047700	-2.70516000	1.08835500
C	-2.35391700	-3.17324100	2.06792300
H	-0.43609900	-3.56601200	2.98071700
C	3.18759100	-0.11247400	3.99024900
C	-3.77063400	-2.59814100	-1.28821900
H	-3.03247900	-2.46686600	-3.31446900
C	-0.17437000	1.47358700	-4.70278800
H	-4.60796500	-3.77485900	0.87286200
O	-2.25589200	-4.30404500	1.24349600
C	2.89113400	-1.47474400	3.67972400
O	-3.33027500	-3.92513200	-1.13164700
C	-0.46992800	0.09999600	-4.96019100
C	2.75775800	4.82692300	-2.07029200

C	3.80949500	4.18493700	0.46664400
C	3.89636300	-2.29639900	3.12178500
C	4.49250500	0.38483700	3.77049900
C	0.53538200	-0.73749900	-5.49956700
C	1.12973100	1.96437900	-4.93933400
C	-6.04643500	-2.44486500	1.69620800
H	-6.82325500	-2.75255900	0.99190000
H	-6.22010400	-1.39316800	1.93698400
C	-4.05624500	0.35909300	5.39600700
H	-4.40277100	-0.54396900	5.90559300
H	-4.79568100	0.61146300	4.63102500
C	-4.62353000	4.46696500	2.47838400
H	-4.54091300	4.83753100	3.50342000
H	-5.20973600	3.54413800	2.50834900
C	-6.64363500	1.62866600	-1.28223400
H	-7.00375400	2.63204500	-1.52294700
H	-6.67126800	1.52324800	-0.19414800
H	-7.34100800	0.89952900	-1.70312500
H	-5.18809200	5.19512300	1.89026900
H	-4.04018000	1.17962000	6.11761700
H	-6.16525500	-3.01926200	2.61835700
C	2.09536600	1.12354000	-5.44914000
C	1.79361000	-0.22703500	-5.74237600
H	2.56656500	-0.86844700	-6.15229000
H	3.09878700	1.49530300	-5.62560400
H	0.28329800	-1.77080400	-5.71409900
H	1.33185000	3.00617500	-4.71550400
C	4.62936000	4.23151700	-0.64331700
H	4.19188000	3.95477500	1.45547900
C	4.10051800	4.54865300	-1.91762700
H	2.32974700	5.08551100	-3.03284900
C	5.46210800	-0.44179200	3.24101200
H	4.69335400	1.41979000	4.02445100
C	5.16019300	-1.78414200	2.91078400
H	3.65107000	-3.32967700	2.89966700
H	5.93199600	-2.42166000	2.49669900
H	6.46266400	-0.05926800	3.07392300
H	4.76030600	4.57813500	-2.77835900
H	5.69233600	4.03529500	-0.53622100
P	-1.92586000	-4.20384300	-0.34868900
N	-1.72733500	-5.82640900	-0.68641200
C	-0.75002900	-6.56371200	0.12524700
C	-1.71651800	-6.20340500	-2.10737000
H	-0.85749400	-6.29613600	1.17577900
H	0.28466900	-6.37669100	-0.19840400
H	-0.95726100	-7.63182300	0.02119900
H	-2.50857800	-5.67814600	-2.64005400
H	-1.91183000	-7.27658500	-2.17592300
H	-0.74972600	-5.99124500	-2.58640100
Au	-0.31105400	-2.72809700	-1.06868500
N	-1.70596800	-0.41321600	-4.66994700
N	-1.14233000	2.31620100	-4.23153800
N	0.56842300	5.07559500	-1.10402800
N	1.62210700	4.42525700	1.44002600
N	2.22063400	0.72110100	4.48153400
N	1.64655300	-1.99363500	3.92506000
C	2.26902700	-1.53703000	-1.71984000
C	1.09680900	-1.66245000	-2.18183900
H	0.73111000	-1.23965700	-3.11013100
C	3.40363200	-1.65596300	-0.79699500
C	4.07815700	-0.27907400	-0.64444800
H	4.12205400	-2.39128800	-1.16805000
H	3.03090000	-2.00876600	0.16983300
C	3.98874200	0.34308900	-2.04867500
O	3.28034000	-0.20287100	-2.88924300
O	4.65560500	1.44042200	-2.31822600
H	5.24741700	1.64500000	-1.55364400
C	3.34658800	0.62848300	0.40523500
H	3.82110300	1.61157500	0.38948700
H	3.55135700	0.19236800	1.38183900
C	5.57647800	-0.30287600	-0.30954500
O	6.19249800	0.75738100	-0.26508600
O	6.08577400	-1.50851900	-0.17596800
C	7.57212000	-1.75613100	-0.22324500
C	1.85843400	0.75378700	0.19256600
C	0.95710100	0.05515800	1.00383300
C	1.35343800	1.56030700	-0.83396400
C	-0.42068000	0.14804800	0.79057300
H	1.33346000	-0.57283700	1.80140400
C	-0.01772600	1.65231700	-1.05492700
H	2.03043300	2.12386300	-1.46687600
C	-0.91183600	0.94513000	-0.24571100
H	-1.10610400	-0.38670100	1.43850000
H	-0.38543700	2.28677100	-1.84955500
H	-1.98038800	1.03007200	-0.41081700
C	8.09023500	-1.24500800	-1.56556800
H	7.51742700	-1.68021000	-2.38964400
H	8.04311900	-0.15726100	-1.62693700
H	9.13370800	-1.54937400	-1.68280900
C	7.64397200	-3.27624500	-0.13893100
H	7.11922100	-3.73927500	-0.97866000
H	8.68912400	-3.59446100	-0.16754400
H	7.20137600	-3.63852400	0.79272600
C	8.26675800	-1.09184600	0.96113400
H	8.13741400	-0.01015500	0.94379500
H	7.89845800	-1.48937200	1.90905200
H	9.33612100	-1.31432700	0.90399800

1b-Int$_{5anti}$H^{+}⊂AuCav

C	-2.31239500	4.14108300	2.91529100
C	-1.68786400	2.84861500	3.43271400
C	-2.37612700	4.13932600	1.39245900
H	-1.62846100	4.94308600	3.19370100
C	-2.45413500	1.72428700	3.74495700
C	-0.30680600	2.76099600	3.65447800
C	-3.42402500	3.53464000	0.69246100
C	-1.36723700	4.74506400	0.63461500
C	-1.91214400	0.56245500	4.30175100
H	-3.52436000	1.76805800	3.58286300
C	0.28744600	1.61994700	4.18494700
O	0.45535200	3.91375500	3.45734700
C	-3.48917900	3.50703300	-0.70350400
H	-4.21778800	3.06034000	1.25818900
C	-1.39319900	4.76234100	-0.75590300
O	-0.31953400	5.37500300	1.31011300
C	-2.78703200	-0.63068100	4.67489100
C	-0.52808300	0.53949600	4.51138200
H	1.35187400	1.59032400	4.37975700
C	1.32544100	4.01149200	2.41648200
C	-4.64203400	2.84762900	-1.45369600
C	-2.44112700	4.11641200	-1.40402100
H	-0.59479400	5.22711100	-1.31923600
C	0.91241500	4.78953500	1.27170100
H	-2.23304400	-1.20120300	5.42032100
C	-2.95189000	-1.54007700	3.46298300
O	0.02625500	-0.56577300	5.17332800
C	-4.41885500	1.34507300	-1.58590200
H	-4.61959300	3.24967000	-2.46671100
O	-2.46706100	4.06915600	-2.80085800
C	-3.94071000	-1.33527000	2.49802800
C	-2.07002200	-2.60674900	3.25460600
C	0.81281100	-1.42619300	4.47227800
C	3.36713000	3.70435600	1.45883600
C	-4.84951900	0.44291600	-0.60902900
C	-3.78276000	0.81134200	-2.71614200
C	-1.57760500	3.21564700	-3.39318900
C	2.94880200	4.44694700	0.31360800
C	-4.07930900	-2.14342100	1.36539100
H	-4.63716300	-0.51764000	2.63731700
C	-2.15298100	-3.43151400	2.13696700
O	-1.10631800	-2.84783500	4.23051300
C	0.20077300	-2.61359100	3.92594500
C	-4.69989800	-0.94127000	-0.73164100
H	-5.34358400	0.83333300	0.27221100
C	-3.60507700	-0.56077400	-2.88102400
O	-3.40259000	1.68404100	-3.73432400
C	-2.07388200	1.96363400	-3.90315300
C	-5.18438300	-1.91019100	0.34048900
C	-3.15144700	-3.18079900	1.20247700
H	-1.45681200	-4.25001200	2.00773400

C	2.82196800	-2.10350300	3.64061700
C	-4.07239600	-1.41659700	-1.88848300
H	-3.13140200	-0.94492200	-3.77548400
C	0.50608300	2.69373100	-4.15683300
H	-5.36116600	-2.86716000	-0.15558200
O	-3.26545400	-4.05561200	0.11603400
C	2.19691100	-3.23011900	3.02493100
O	-3.97052500	-2.80237700	-2.08747800
C	-0.00033300	1.48362700	-4.72133600
C	3.84850400	4.64050700	-0.75921400
C	4.68619100	3.19806800	1.51400800
C	2.96679700	-4.11773300	2.23936900
C	4.21803700	-1.92404200	3.50138000
C	0.86233800	0.65130600	-5.47192700
C	1.87160900	3.02165400	-4.31840100
C	-6.51598300	-1.48808500	0.97781800
H	-7.28375000	-1.39316400	0.20587400
H	-6.45838300	-0.53163500	1.50350000
C	-4.12660100	-0.22403000	5.30517000
H	-4.68457300	-1.11480500	5.60569300
H	-4.76468100	0.35140300	4.62891300
C	-3.67113700	4.45020300	3.56144000
H	-3.57072700	4.48453700	4.64940800
H	-4.43946400	3.70907900	3.32396400
C	-6.01001600	3.19639200	-0.84734400
H	-6.15109700	4.28014500	-0.84996800
H	-6.12164700	2.85498500	0.18551700
H	-6.81070100	2.74067600	-1.43590000
H	-4.03520200	5.42100600	3.21540200
H	-3.95020800	0.39027700	6.19170900
H	-6.83998400	-2.24171100	1.69994900
C	2.69127900	2.19380500	-5.05616900
C	2.18258400	1.01089600	-5.64273800
H	2.84151300	0.38030800	-6.23105500
H	3.73602300	2.45246100	-5.19481500
H	0.45198800	-0.25291400	-5.90842700
H	2.23237900	3.94121700	-3.87060100
C	5.55282200	3.41193300	0.45934100
H	4.98835700	2.65902400	2.40576400
C	5.13018300	4.13182400	-0.68328800
H	3.50578900	5.21042600	-1.61604200
C	4.95164200	-2.82144100	2.75311400
H	4.67690700	-1.07796900	4.00157500
C	4.32345200	-3.91155600	2.10652700
H	2.46526600	-4.96252900	1.77958700
H	4.91632600	-4.60144000	1.51674300
H	6.02519900	-2.69858300	2.66892000
H	5.82643800	4.29862900	-1.49932400
H	6.56723300	3.02994100	0.50789300
P	-2.75328300	-3.64730800	-1.38483600
N	-2.97974300	-5.12331800	-2.14094300
C	-2.28875000	-6.27686200	-1.55053900
C	-2.95534200	-5.11638200	-3.61047000
H	-2.40023100	-6.26555300	-0.46668400
H	-1.21964900	-6.30068000	-1.80954800
H	-2.75710000	-7.18793000	-1.93225100
H	-3.53401200	-4.27484200	-3.98981400
H	-3.42288000	-6.03955400	-3.96304500
H	-1.93235300	-5.06652000	-4.01089600
Au	-0.78626500	-2.47355300	-1.70655300
N	-1.31079500	1.12684100	-4.54298200
N	-0.32433400	3.55009400	-3.48805100
N	1.68978200	4.98185600	0.24832400
N	2.50984400	3.48197300	2.50325600
N	2.08551400	-1.18926200	4.34393200
N	0.86010100	-3.46615000	3.19911300
C	1.98690100	-1.16256900	-2.09099500
C	0.72180100	-1.29748400	-2.42074400
H	0.37241200	-0.54751300	-3.12684800
C	3.01767900	-1.77894300	-1.18290600
C	3.86116800	-0.56641100	-0.73430800
H	3.64399800	-2.49836800	-1.72201400
H	2.55015400	-2.29430900	-0.34440000
C	3.64440900	0.37435500	-1.90198200
O	2.67841400	0.05670800	-2.65716700
O	4.34963300	1.41573700	-2.14753000
H	5.13572900	1.37140400	-1.51220400
C	3.29072000	0.10452100	0.58165500
H	3.85680700	1.02015500	0.76210300
H	3.51683300	-0.59440200	1.38950700
C	5.37548900	-0.71243500	-0.57878100
O	6.07543700	0.30136400	-0.65852000
O	5.80222700	-1.93163000	-0.38703900
C	7.28155900	-2.26126900	-0.43254200
C	1.81082900	0.38122300	0.50333300
C	0.88590500	-0.49044700	1.08646400
C	1.33445700	1.48152200	-0.21975200
C	-0.48528100	-0.28042000	0.94156300
H	1.24092100	-1.35139500	1.63584000
C	-0.03266300	1.68055100	-0.38516400
H	2.02858700	2.18405900	-0.66957200
C	-0.94992000	0.80203900	0.19519000
H	-1.18783200	-0.96307000	1.40072400
H	-0.37604000	2.52767700	-0.95868600
H	-2.01434500	0.96393600	0.07006700
C	7.78216100	-1.94425900	-1.83913300
H	7.19512800	-2.48284300	-2.58846800
H	7.74588100	-0.87449200	-2.04755500
H	8.82161000	-2.27179000	-1.92589900
C	7.29302900	-3.76104900	-0.16598300
H	6.64449400	-4.28706100	-0.87104000
H	8.31116100	-4.13941800	-0.28625600
H	6.96700000	-3.98525500	0.85111000
C	8.02771900	-1.48933400	0.65131500
H	8.07987800	-0.42474300	0.42913600
H	7.55124500	-1.62540800	1.62476400
H	9.04541500	-1.88419800	0.71850200

1b-TS1$_{5syn}$H^{+}⊂AuCav

C	1.92951900	4.11157600	3.19551500
C	1.60745600	4.29587500	1.71579200
C	1.79389600	2.64793600	3.60529900
H	1.16089200	4.65500700	3.74557400
C	2.56611300	4.09875300	0.71741500
C	0.32281800	4.67179500	1.30145100
C	2.86326800	1.74983600	3.54646500
C	0.57507000	2.15842700	4.09274800
C	2.29089600	4.26616900	-0.64273500
H	3.56523200	3.79893200	1.01206300
C	0.00231800	4.87265900	-0.03688600
O	-0.65551100	4.88063800	2.27361100
C	2.76094500	0.42341200	3.97384700
H	3.81996800	2.10625800	3.18326900
C	0.41305100	0.84089900	4.50713000
O	-0.46962700	3.06944100	4.26458900
C	3.33292400	4.02391700	-1.72780500
C	0.99097500	4.64972400	-0.98831900
H	-0.99916500	5.15798100	-0.32912300
C	-1.65226000	3.95318500	2.37995700
C	3.93901800	-0.54547600	3.94067700
C	1.51445700	-0.00571000	4.44401300
H	-0.53395700	0.49335500	4.89883200
C	-1.56048700	2.99689200	3.45559600
H	2.96840500	4.52382700	-2.62522500
C	3.42058600	2.54032100	-2.07080900
O	0.69683600	4.79896300	-2.34838100
C	3.98537100	-1.24135400	2.58622600
H	3.72771300	-1.31779200	4.68022000
O	1.43264300	-1.30001200	4.95696200
C	4.27839200	1.67937800	-1.37966200
C	2.66739800	1.99078200	-3.11941200
C	-0.11560300	3.85219600	-2.89464000
C	-3.66280200	3.07371800	1.78103500
C	4.61032300	-0.65200100	1.48439700
C	3.37258900	-2.48641800	2.38740500
C	0.70061800	-2.24184700	4.30005100
C	-3.59369700	2.15855100	2.87453600
C	4.44309500	0.33286000	-1.71391900
H	4.86362800	2.08344200	-0.56303000
C	2.80865200	0.65654700	-3.50018100

O	1.84491500	2.83821000	-3.86933300
C	0.48892400	2.82531400	-3.70985100
C	4.65496100	-1.24441300	0.22016200
H	5.09347900	0.30747200	1.62170100
C	3.37446900	-3.11114900	1.14117800
O	2.78522600	-3.12686000	3.47381500
C	1.41685100	-3.22023700	3.52277900
C	5.38585900	-0.58991400	-0.94936300
C	3.69863500	-0.15091700	-2.79451200
H	2.25183800	0.26848700	-4.34389700
C	-2.17882900	2.97352000	-3.31208000
C	4.00250200	-2.47547300	0.07638100
H	2.89403600	-4.07364100	1.01554500
C	-1.25471400	-3.30979800	3.83153100
H	5.66465000	-1.39281700	-1.63530100
O	3.88783000	-1.48208400	-3.20381400
C	-1.59559100	2.01085600	-4.18632900
O	4.03907200	-3.14364300	-1.15671500
C	-0.54320900	-4.29190500	3.07506400
C	-4.67677400	1.28400100	3.12025200
C	-4.79423700	3.06170200	0.93389700
C	-2.42983600	1.10068000	-4.87635700
C	-3.58055800	2.98455700	-3.11733200
C	-1.24621100	-5.37930100	2.50948700
C	-2.65553500	-3.42815100	3.98478800
C	6.68992400	0.10239000	-0.52640700
H	7.34914800	-0.61468600	-0.03109700
H	6.53143500	0.93480300	0.16376400
C	4.69810700	4.63727900	-1.38027200
H	5.40708700	4.46879900	-2.19516100
H	5.13567200	4.22326200	-0.46776700
C	3.28695100	4.71881000	3.58000800
H	3.30410700	5.78304600	3.33112700
H	4.13001100	4.24840200	3.06618400
C	5.27330500	0.11072100	4.32295800
H	5.19877800	0.55029500	5.32080900
H	5.57581600	0.90622000	3.63620800
H	6.07054700	-0.63720700	4.33225100
H	3.45437900	4.61071500	4.65473800
H	4.59109500	5.71458500	-1.23087900
H	7.20368200	0.49729400	-1.40648200
C	-3.31492500	-4.50882300	3.43593900
C	-2.60704800	-5.48920500	2.70077100
H	-3.14502200	-6.33125700	2.27821700
H	-4.38614800	-4.61702800	3.57695800
H	-0.68434700	-6.11683900	1.94719200
H	-3.17040100	-2.67235600	4.56832100
C	-5.83660400	2.19414400	1.19263400
H	-4.82363000	3.76809500	0.11126800
C	-5.78269900	1.30882100	2.29513800
H	-4.60734900	0.61259800	3.96926100
C	-4.37399200	2.08592500	-3.80123500
H	-3.99561600	3.72786600	-2.44545900
C	-3.79670000	1.14862900	-4.69183600
H	-1.96762500	0.38430800	-5.54670700
H	-4.43827200	0.46130300	-5.23406600
H	-5.45069400	2.10156700	-3.66629500
H	-6.61695900	0.64404700	2.49196700
H	-6.71516000	2.20806900	0.55590000
P	3.09911700	-2.68373000	-2.40820300
N	3.38943600	-3.95502500	-3.44850700
C	2.94491700	-3.77415000	-4.83810500
C	3.15841500	-5.31094700	-2.92890400
H	3.21033100	-2.77729200	-5.18887000
H	1.86188700	-3.92602100	-4.95075700
H	3.46722700	-4.50579700	-5.45975800
H	3.57130900	-5.40250500	-1.92511900
H	3.67971600	-6.01738900	-3.57987900
H	2.09062700	-5.57273000	-2.91279100
Au	0.98152300	-1.86415000	-2.10318400
N	0.81575400	-4.20032500	2.91702400
N	-0.59229600	-2.27383200	4.43135200
N	-2.49683500	2.12725100	3.69289700
N	-2.65568600	3.97422000	1.55466100
N	-1.39691100	3.89607800	-2.67586500
N	-0.23306000	1.93719700	-4.33266800
C	-1.90769500	-0.97195500	-2.20668700
C	-0.72655000	-0.77356700	-2.59020900
H	-0.40890100	0.08989900	-3.16764000
C	-3.31819800	-0.77815600	-1.88812100
C	-3.66743900	-1.36232700	-0.50891800
H	-3.95012100	-1.23324000	-2.65638300
H	-3.50720700	0.30055400	-1.90884800
C	-2.93384400	-2.70958100	-0.45838500
O	-1.97694100	-2.87345500	-1.20619900
O	-3.31393700	-3.61693600	0.40682600
H	-4.17794500	-3.32737800	0.80210600
C	-3.17833200	-0.45650100	0.68440400
H	-3.77478300	0.45496600	0.63218300
H	-3.44503800	-0.96827100	1.61281900
C	-5.16799800	-1.56972500	-0.30516600
O	-5.58366700	-2.35673100	0.54117200
O	-5.89911100	-0.81662400	-1.09752600
C	-7.38673500	-0.97852300	-1.22647100
C	-1.70976800	-0.12097900	0.65831300
C	-0.76181100	-1.05547700	1.09000500
C	-1.26804600	1.11254300	0.16288100
C	0.60025300	-0.78107600	0.98791500
H	-1.08802900	-2.00761800	1.49336600
C	0.09472400	1.39000300	0.06801600
H	-1.98910000	1.85658100	-0.16156000
C	1.03483100	0.44189300	0.47046900
H	1.32435000	-1.51469300	1.30997200
H	0.42451900	2.34360600	-0.31665900
H	2.09462800	0.65825000	0.40015400
C	-8.07257400	-0.70915200	0.10882700
H	-7.79127300	-1.44649200	0.85983100
H	-7.82892600	0.28977500	0.47399100
H	-9.15505400	-0.75716700	-0.03963300
C	-7.66300500	-2.38365000	-1.75435800
H	-7.39503800	-3.14470200	-1.02016500
H	-8.72967800	-2.48018800	-1.97412500
H	-7.10918400	-2.56332200	-2.68033800
C	-7.72758600	0.09187500	-2.25767200
H	-7.44832800	1.08300600	-1.88941800
H	-7.20162000	-0.09412400	-3.19807900
H	-8.80226800	0.08622000	-2.45545900

1b-Int$_{5syn}$H$^+$⊂AuCav

C	-1.58999000	4.69443300	-2.42942400
C	-1.44755600	4.55496500	-0.91690800
C	-1.38097200	3.35061000	-3.12165300
H	-0.77194700	5.33679800	-2.75611600
C	-2.51977600	4.18512500	-0.10071300
C	-0.22094600	4.80056400	-0.28629700
C	-2.43217300	2.46358700	-3.36695000
C	-0.10929900	2.97154700	-3.57022100
C	-2.40984300	4.06025400	1.28698000
H	-3.47917400	3.98809300	-0.56468400
C	-0.06001500	4.70383200	1.09160600
O	0.85600900	5.20561900	-1.07592400
C	-2.26127100	1.25727500	-4.05073400
H	-3.42796100	2.73715500	-3.03888700
C	0.12079900	1.76824800	-4.22952700
O	0.92818600	3.89823300	-3.44173400
C	-3.58426900	3.64664900	2.16555900
C	-1.15649400	4.31590900	1.85447000
H	0.90021800	4.89293700	1.55228500
C	1.88468800	4.33669800	-1.28610300
C	-3.42214500	0.31358500	-4.34564600
C	-0.96426600	0.92889900	-4.46019600
H	1.11036200	1.50868500	-4.58291600
C	1.93642300	3.66149900	-2.56099900
H	-3.33161200	3.94765700	3.18222200
C	-3.73150000	2.12904700	2.19263200
O	-1.02485300	4.16476400	3.23918000
C	-3.62154800	-0.62390700	-3.16248200
H	-3.11423200	-0.30662500	-5.18727500
O	-0.80165200	-0.23447900	-5.21575600
C	-4.47880600	1.44452600	1.22932300

C	-3.13785000	1.36449500	3.20732400
C	-0.31981100	3.07941500	3.66974600
C	3.87321500	3.39004600	-0.71497600
C	-4.40021000	-0.25853100	-2.06206200
C	-3.00302600	-1.88067200	-3.12275500
C	-0.13709700	-1.30225700	-4.69612800
C	3.94912000	2.76611400	-1.99646800
C	-4.68532800	0.06301500	1.26559200
H	-4.93772400	2.01541100	0.43134200
C	-3.32199200	-0.01439500	3.29049400
O	-2.42068400	2.02618000	4.20836600
C	-1.05553600	1.95876400	4.20532400
C	-4.59760400	-1.08611600	-0.95468700
H	-4.89088400	0.70662100	-2.07820400
C	-3.14870600	-2.73425200	-2.03044300
O	-2.28422100	-2.30116500	-4.24054300
C	-0.92083100	-2.40169400	-4.18457700
C	-5.49855900	-0.67141800	0.20544400
C	-4.10163300	-0.64253300	2.32310400
H	-2.87701400	-0.57581500	4.10217400
C	1.63893500	2.00651700	4.12390800
C	-3.93756000	-2.32275700	-0.96028000
H	-2.66948900	-3.70557200	-2.03018300
C	1.76784900	-2.49044100	-4.30589900
H	-5.85958500	-1.59268100	0.66780600
O	-4.34552200	-2.02043500	2.43125000
C	0.91543700	0.93082800	4.71990600
O	-4.14670800	-3.22626300	0.08830700
C	0.99215300	-3.58561200	-3.81665100
C	5.09457900	2.01221000	-2.33858900
C	4.92656000	3.20708000	0.21067000
C	1.62584900	-0.14704600	5.29964200
C	3.05308100	1.96795600	4.09383400
C	1.63924600	-4.78508700	-3.44228800
C	3.17507700	-2.61143000	-4.38192000
C	-6.73919400	0.11217900	-0.24728300
H	-7.30864400	-0.47454200	-0.97242500
H	-6.49740100	1.06987100	-0.71509600
C	-4.88813900	4.36333400	1.78192100
H	-5.69273700	4.07077000	2.46167100
H	-5.21735000	4.14030500	0.76336100
C	-2.90292000	5.37904000	-2.83756400
H	-2.97215700	6.36402600	-2.36868400
H	-3.79243700	4.81365500	-2.54595700
C	-4.70655100	1.04745200	-4.75647200
H	-4.51651700	1.66267200	-5.63961100
H	-5.09579300	1.70677200	-3.97578100
H	-5.49041100	0.32509100	-4.99907200
H	-2.93602100	5.50769600	-3.92252900
H	-4.75089800	5.44538500	1.85189800
H	-7.38061900	0.31984100	0.61286800
C	3.78099900	-3.79981200	-4.02571400
C	3.00936200	-4.89167100	-3.56086300
H	3.50371300	-5.82044700	-3.29497600
H	4.85769600	-3.90667500	-4.11842200
H	1.02782100	-5.60815900	-3.08900900
H	3.73980800	-1.76733800	-4.76342300
C	6.03292700	2.46099500	-0.14588000
H	4.84698200	3.69282000	1.17741400
C	6.12222700	1.87081700	-1.42890000
H	5.13667100	1.56989500	-3.32815000
C	3.72202300	0.90545900	4.66666900
H	3.57584300	2.80098700	3.63600600
C	3.00559600	-0.15257400	5.27614300
H	1.05709400	-0.94932500	5.75693500
H	3.54897400	-0.97353500	5.73298400
H	4.80733800	0.88107000	4.65750200
H	7.00826900	1.30832300	-1.70266000
H	6.85256200	2.34677600	0.55656900
P	-3.42390000	-3.05550500	1.54778100
N	-3.90254600	-4.50190600	2.23940000
C	-3.70266000	-4.62312000	3.69041500
C	-3.58388300	-5.73136100	1.50138800
H	-4.01458700	-3.70567200	4.18868700
H	-2.65686400	-4.84233600	3.95007300
H	-4.33131700	-5.44013200	4.05428600
H	-3.81399000	-5.60375500	0.44429200
H	-4.21076700	-6.53674300	1.89324100
H	-2.52968000	-6.02444100	1.61483100
Au	-1.24073900	-2.32952200	1.71110300
N	-0.37383100	-3.49555600	-3.74374900
N	1.16232500	-1.33869100	-4.73193300
N	2.93243500	2.89970500	-2.90266300
N	2.80682500	4.18276300	-0.38291500
N	0.97909000	3.08390300	3.60175100
N	-0.45495900	0.92372800	4.71548100
C	1.77587300	-1.81259700	1.75877000
C	0.58135700	-1.60728500	2.26486600
H	0.58292400	-0.84827300	3.04014900
C	3.13799600	-1.18584300	1.89057800
C	3.71560600	-1.33397700	0.47157900
H	3.76578800	-1.71707700	2.61456200
H	3.06763000	-0.14756100	2.20960800
C	2.97919400	-2.56158000	0.00477900
O	1.91471700	-2.80874800	0.65468400
O	3.34872400	-3.32064500	-0.95522500
H	4.28342200	-3.02337600	-1.20187500
C	3.33550300	-0.11865000	-0.47272700
H	3.90616900	0.72480000	-0.08303800
H	3.71184900	-0.34482500	-1.47334700
C	5.21165200	-1.57944200	0.33211900
O	5.64037700	-2.23434800	-0.62161400
O	5.90978700	-1.02589800	1.28721200
C	7.38696900	-1.26878000	1.47264900
C	1.86089300	0.18951600	-0.49999200
C	1.00574900	-0.55762300	-1.31915500
C	1.31337600	1.17458200	0.33202400
C	-0.37177700	-0.36147200	-1.27280900
H	1.41988600	-1.30150100	-1.99143200
C	-0.06583600	1.37104700	0.37206700
H	1.95948000	1.78619300	0.95412900
C	-0.91474400	0.59860000	-0.41921900
H	-1.02665000	-0.95606900	-1.89216700
H	-0.48226900	2.12882100	1.01787500
H	-1.98725300	0.75377900	-0.38249100
C	8.15526700	-0.75101200	0.26276700
H	7.89261100	-1.29632000	-0.64360600
H	7.96595200	0.31303300	0.11363700
H	9.22525200	-0.88005600	0.44820700
C	7.59226400	-2.76171600	1.71043000
H	7.36766400	-3.34474100	0.81629800
H	8.63716500	-2.93743400	1.97991300
H	6.96758000	-3.10998500	2.53795500
C	7.68056500	-0.44799600	2.72267400
H	7.44580600	0.60654300	2.55545400
H	7.09278100	-0.80813100	3.57123600
H	8.74033100	-0.53097400	2.97599000

1b-TS1$_6$H$^+$$\subset$AuCav

C	1.73139200	3.86338900	3.67699200
C	1.10349800	4.31967400	2.36403600
C	1.93356400	2.35118200	3.69201600
H	1.00032800	4.08017700	4.45633000
C	1.86239500	4.54245300	1.21138000
C	-0.27781000	4.52735400	2.26725100
C	3.13109800	1.75494400	3.28844500
C	0.92012700	1.50248000	4.15925800
C	1.30273300	4.96461500	0.00074100
H	2.93117500	4.36494300	1.25425600
C	-0.88012700	4.96574000	1.09410600
O	-1.06866600	4.31307300	3.39548600
C	3.35232300	0.37876000	3.36932600
H	3.93429800	2.39130300	2.93729800
C	1.08249500	0.12173200	4.24038800
O	-0.22581600	2.09602900	4.68095500
C	2.14028900	5.13238400	-1.26352500
C	-0.08173600	5.17085700	-0.02478300
H	-1.94932100	5.12740900	1.05039800
C	-1.87922700	3.21092000	3.36344500
C	4.66877000	-0.27149200	2.95710300

C	2.30289100	-0.41606700	3.84687200
H	0.29537000	-0.50600500	4.63732500
C	-1.41890400	2.01938800	4.02756000
H	1.54585100	5.72835800	-1.95649700
C	2.33266400	3.76749800	-1.91720000
O	-0.67431700	5.61001800	-1.21252500
C	4.56637300	-0.69612500	1.49793900
H	4.76707100	-1.18470300	3.54401200
O	2.58865700	-1.76506400	4.04893700
C	3.43811700	2.95943500	-1.64566100
C	1.38214800	3.26961800	-2.82032800
C	-1.49945800	4.71651700	-1.83387500
C	-3.81678700	2.15422400	2.81970200
C	4.77223300	0.21929300	0.46239500
C	4.21930900	-2.00586400	1.13960300
C	2.00484100	-2.76971300	3.34981100
C	-3.36576500	0.97582700	3.48851900
C	3.63885600	1.71279000	-2.24497600
H	4.19688900	3.33426300	-0.97082400
C	1.51171400	2.01468500	-3.41018700
O	0.35993100	4.12129400	-3.21264800
C	-0.94287100	3.89361100	-2.87553600
C	4.65672200	-0.11827900	-0.88725700
H	5.03243100	1.23889900	0.72015400
C	4.06793500	-2.38475800	-0.19380000
O	4.08158600	-2.96900300	2.13444500
C	2.82538900	-3.45527000	2.38292300
C	4.89970800	0.89419900	-1.99856800
C	2.63751900	1.25604900	-3.11001000
H	0.77411300	1.67071600	-4.12454100
C	-3.53976100	3.77379200	-2.19375600
C	4.28438100	-1.43229500	-1.18163200
H	3.81240700	-3.40731500	-0.44377200
C	0.35391100	-4.30452500	3.01957300
H	5.09089700	0.32614900	-2.91203500
O	2.83300900	0.07296600	-3.84327600
C	-2.98809000	2.96393000	-3.23327700
O	4.14646200	-1.81257100	-2.52982600
C	1.19744700	-5.02421400	2.11867100
C	-4.21600300	-0.15123100	3.55574200
C	-5.09420400	2.16980600	2.21612900
C	-3.84408100	2.13214400	-3.99107800
C	-4.92351800	3.70776400	-1.91147000
C	0.75206300	-6.25215200	1.57479200
C	-0.93177600	-4.81243800	3.32148100
C	6.14784600	1.75305200	-1.74691200
H	7.02757200	1.10988100	-1.66659000
H	6.08706700	2.33969200	-0.82698400
C	3.45524400	5.88227100	-1.01108300
H	4.01573300	5.99142900	-1.94350400
H	4.10464800	5.38047600	-0.28821500
C	3.00961700	4.64612900	4.01275700
H	2.78469400	5.71381900	4.07621800
H	3.79809500	4.52312500	3.26476600
C	5.90138800	0.59783000	3.24064000
H	5.94204600	0.85239600	4.30274600
H	5.90827400	1.53470600	2.67675800
H	6.81180200	0.05164300	2.98044200
H	3.41123500	4.31822000	4.97518100
H	3.24120400	6.87890000	-0.61739100
H	6.29967000	2.44862500	-2.57610800
C	-1.34645100	-6.00607800	2.77002400
C	-0.49982800	-6.73178200	1.89933400
H	-0.84575200	-7.67131400	1.48247600
H	-2.33414700	-6.39127800	2.99525300
H	1.42724400	-6.80046900	0.92485500
H	-1.55494300	-4.25019000	4.00849200
C	-5.90548900	1.05725300	2.30065900
H	-5.41112700	3.07846500	1.71580900
C	-5.46594800	-0.10741100	2.97311500
H	-3.86335700	-1.02459000	4.09424200
C	-5.73917300	2.90202900	-2.67714000
H	-5.31178100	4.33255400	-1.11449800
C	-5.19847800	2.12328600	-3.72829100
H	-3.41166600	1.54845900	-4.79671800
H	-5.86287200	1.52260500	-4.33964400
H	-6.80719400	2.87477500	-2.48706500
H	-6.11905600	-0.97072000	3.02759900
H	-6.89785300	1.07847500	1.86281200
P	2.75008600	-1.44688200	-3.28911100
N	2.97670200	-2.26786300	-4.72336100
C	2.11088900	-1.90981300	-5.85564100
C	3.23470100	-3.71124700	-4.61361700
H	2.02495900	-0.82704900	-5.93460400
H	1.10846600	-2.35412700	-5.76824200
H	2.57678200	-2.28468200	-6.77036400
H	3.93727400	-3.90661800	-3.80395000
H	3.68988600	-4.04712500	-5.54859900
H	2.31226000	-4.28621400	-4.44659500
Au	0.93492500	-2.10483300	-2.02391600
N	2.43271300	-4.53896500	1.78161400
N	0.79334500	-3.15838100	3.62570300
N	-2.13163600	0.93326000	4.07958000
N	-3.03061800	3.27225600	2.76562400
N	-2.75026100	4.64094900	-1.48870500
N	-1.65430300	3.03198400	-3.54028600
C	-0.81360700	-3.06113700	-1.27227600
C	-0.36793100	-4.15019100	-0.84177200
H	0.22586100	-4.97313500	-0.50050700
C	-2.19926200	-2.50650200	-1.34310000
C	-2.94506500	-2.73901200	-0.01640800
H	-2.71703300	-3.01028400	-2.16311400
H	-2.19427400	-1.44132600	-1.56358500
C	-2.98963700	-4.23874400	0.37588200
O	-2.22733600	-5.07666100	-0.08667100
O	-3.85388400	-4.57484300	1.31922500
H	-4.46295900	-3.81270100	1.48782800
C	-2.22755700	-2.03071800	1.20378800
H	-2.98840400	-1.83335300	1.96208100
H	-1.51991100	-2.73972700	1.63613100
C	-4.40501500	-2.27140500	-0.12255500
O	-5.16481800	-2.36198100	0.83786300
O	-4.72587800	-1.85346000	-1.32597800
C	-6.13548500	-1.53168200	-1.72387800
C	-1.48835100	-0.76541800	0.85041600
C	-2.17365300	0.39983200	0.48438100
C	-0.08690500	-0.73984500	0.89101000
C	-1.47186500	1.56280100	0.16723800
H	-3.26010600	0.40929000	0.47305200
C	0.61983300	0.42234700	0.57163100
H	0.45037500	-1.62953700	1.20060400
C	-0.07464300	1.57567400	0.20601600
H	-2.01671900	2.46568000	-0.08145000
H	1.70332500	0.43258900	0.63452500
H	0.46874700	2.48252900	-0.02520000
C	-7.04211300	-2.72317000	-1.42535900
H	-6.63268200	-3.63754400	-1.86488300
H	-7.18344600	-2.87106000	-0.35556100
H	-8.01816300	-2.54022300	-1.88361200
C	-5.98763000	-1.31584700	-3.22554300
H	-6.94388800	-0.99531100	-3.64752700
H	-5.24015200	-0.54717200	-3.42722700
H	-5.68621400	-2.24239000	-3.72167000
C	-6.55642300	-0.26331800	-0.99249900
H	-5.85083300	0.54848300	-1.18417900
H	-7.54194800	0.04874100	-1.35072800
H	-6.61642500	-0.43855500	0.08073600

1b-Int$_6$H$^+$⊂AuCav

C	1.05791400	4.13668900	3.66715200
C	0.47529100	4.38944900	2.28041800
C	1.41667200	2.66425500	3.84899700
H	0.25810800	4.34695500	4.37766000
C	1.30563200	4.60714400	1.17847100
C	-0.90695600	4.42344700	2.04865600
C	2.67602900	2.13525000	3.55222500
C	0.45587900	1.78109700	4.35540100
C	0.82777000	4.87974600	-0.10564700
H	2.37794100	4.56720500	1.32909600
C	-1.43340600	4.69284200	0.78734500

O	-1.78860800	4.25720000	3.12153400
C	2.99441300	0.79057500	3.77038500
H	3.43983300	2.79527500	3.15773500
C	0.71860400	0.43862500	4.59269800
O	-0.77452000	2.32673800	4.70823300
C	1.78446600	5.10244100	-1.27062100
C	-0.56256600	4.91701800	-0.27616600
H	-2.50557600	4.75367000	0.64385700
C	-2.46399300	3.07043600	3.20178200
C	4.35678200	0.19057700	3.43944700
C	1.99261300	-0.02956100	4.29833200
H	-0.03769600	-0.21492300	5.00555100
C	-1.88512900	2.01373800	3.99271600
H	1.21140100	5.59732600	-2.05507300
C	2.21635100	3.75226300	-1.83201300
O	-1.09086500	5.28551500	-1.51636100
C	4.36190300	-0.28975300	1.99211200
H	4.46680700	-0.69768700	4.06152500
O	2.34175700	-1.34682000	4.59973200
C	3.37038200	3.08786200	-1.41567600
C	1.43363100	3.12780400	-2.81169100
C	-1.73030900	4.34116300	-2.26905300
C	-4.24175700	1.72708400	2.74643600
C	4.67115200	0.58376800	0.94601900
C	4.04626800	-1.61334600	1.64906800
C	1.75413500	-2.35730600	3.90962800
C	-3.61982000	0.65234400	3.45065600
C	3.78005600	1.86802500	-1.96244300
H	3.99351100	3.55239200	-0.66178700
C	1.78606200	1.90624400	-3.37710900
O	0.36228000	3.85583500	-3.30077600
C	-0.94322500	3.50896800	-3.14684900
C	4.71588600	0.19016600	-0.39211900
H	4.90241500	1.61345900	1.18869700
C	4.06469300	-2.05050800	0.32496400
O	3.80448700	-2.53972700	2.66395800
C	2.53672500	-3.00152400	2.88278200
C	5.05608000	1.16823200	-1.51025800
C	2.96100300	1.29900300	-2.94567400
H	1.18343300	1.47145400	-4.16462400
C	-3.62259900	3.37062700	-3.08323300
C	4.40504300	-1.14433400	-0.67383800
H	3.84847000	-3.08553900	0.09123300
C	0.06453800	-3.83777500	3.55500300
H	5.41262900	0.57601600	-2.35595300
O	3.39798200	0.15474400	-3.62619000
C	-2.83526800	2.47098800	-3.86546600
O	4.51062400	-1.59952300	-1.99584300
C	0.85097600	-4.50176100	2.56430600
C	-4.27626000	-0.59696400	3.53975400
C	-5.53277900	1.54512900	2.20172800
C	-3.47954800	1.54563700	-4.71925300
C	-5.02965400	3.36477900	-3.21958900
C	0.33942100	-5.66314400	1.94266800
C	-1.22084600	-4.33155700	3.87700700
C	6.18811200	2.13591600	-1.13866100
H	7.08950300	1.57235000	-0.88534100
H	5.94860700	2.77237700	-0.28312700
C	2.96247600	6.01800400	-0.91019100
H	3.59862700	6.17499100	-1.78538300
H	3.59182500	5.61752200	-0.11063800
C	2.22455700	5.08042700	3.99508700
H	1.89952500	6.12057400	3.91074300
H	3.08342900	4.95018800	3.33071600
C	5.52691400	1.12994900	3.76370700
H	5.50967600	1.38944800	4.82527100
H	5.49960300	2.06519600	3.19784600
H	6.47778500	0.63811400	3.54243400
H	2.56975800	4.90619800	5.01755800
H	2.58855100	6.98874000	-0.57532600
H	6.41353400	2.79034900	-1.98446900
C	-1.70129500	-5.46019100	3.24451700
C	-0.91405300	-6.13307800	2.28069400
H	-1.30250300	-7.02595000	1.80274400
H	-2.68237300	-5.84911900	3.49752400

H	0.96372500	-6.17517900	1.21875400
H	-1.79060100	-3.80782900	4.63707500
C	-6.17346700	0.33090500	2.34357400
H	-5.99962500	2.38695100	1.70171300
C	-5.53552100	-0.75135900	2.99604200
H	-3.77687000	-1.39645700	4.07703700
C	-5.63058000	2.48941100	-4.10048500
H	-5.60120400	4.07800600	-2.63571200
C	-4.85473000	1.56669900	-4.84043300
H	-2.86193800	0.87492000	-5.30717600
H	-5.34656300	0.88509800	-5.52711900
H	-6.70828300	2.50544800	-4.22906700
H	-6.05620200	-1.69923800	3.09207900
H	-7.17976700	0.20535200	1.95857800
P	3.27837100	-1.36471900	-3.04827400
N	3.90027000	-2.18718200	-4.36774400
C	3.25611300	-1.94120900	-5.66515100
C	4.24823300	-3.59798400	-4.14861400
H	3.08562100	-0.87402600	-5.80222000
H	2.30322300	-2.48141200	-5.76724100
H	3.93371400	-2.28310100	-6.45187000
H	4.77848800	-3.71051400	-3.20336500
H	4.91639800	-3.91331600	-4.95425900
H	3.36337800	-4.25146800	-4.15248000
Au	1.27070800	-2.13155500	-2.20725900
N	2.09574500	-4.03379900	2.22497000
N	0.55250300	-2.74535400	4.21610400
N	-2.42070400	0.83317400	4.08203100
N	-3.60914800	2.93386100	2.60321100
N	-3.02730400	4.27138400	-2.23929800
N	-1.46786900	2.56856900	-3.87505400
C	-0.39462000	-3.17343400	-1.59309100
C	-0.23264600	-4.39119100	-1.09001200
H	0.65696000	-4.98357400	-0.95672800
C	-1.79745600	-2.62286900	-1.65748600
C	-2.64328500	-3.13526800	-0.47121700
H	-2.26164200	-2.91595200	-2.60968900
H	-1.80510000	-1.53470200	-1.62783600
C	-2.48362000	-4.63629600	-0.38119300
O	-1.36101400	-5.19343100	-0.64618900
O	-3.42935200	-5.40951300	-0.01312500
H	-4.29104800	-4.80355000	-0.01208200
C	-2.11726600	-2.55372500	0.93200400
H	-2.97411300	-2.50930700	1.61082800
H	-1.42284100	-3.28050300	1.35260400
C	-4.12697800	-2.80060700	-0.63802200
O	-5.01715600	-3.60970400	-0.33210400
O	-4.31626900	-1.60732100	-1.10651300
C	-5.64656900	-1.02806500	-1.54360900
C	-1.42595600	-1.21664500	0.82829100
C	-2.14694700	-0.03711200	0.60417200
C	-0.03003600	-1.14638100	0.92622500
C	-1.48295600	1.17865400	0.44332900
H	-3.22748800	-0.07006800	0.54832000
C	0.63606400	0.07233500	0.78846200
H	0.53888600	-2.05331000	1.09425400
C	-0.08940800	1.23724200	0.53206400
H	-2.05374500	2.08140500	0.25890300
H	1.71628600	0.11103000	0.87826600
H	0.42520100	2.18278500	0.41097000
C	-6.80745600	-1.48338400	-0.66628500
H	-7.10748800	-2.50984400	-0.86832600
H	-6.55716400	-1.38798400	0.38995800
H	-7.65428000	-0.82202900	-0.87350600
C	-5.80080700	-1.46417100	-2.99506600
H	-6.71326200	-1.02471900	-3.40756000
H	-4.95522900	-1.12095800	-3.59516100
H	-5.88708200	-2.55198600	-3.06847400
C	-5.38763400	0.46378000	-1.40030900
H	-4.49031800	0.75663900	-1.94702100
H	-6.23157500	1.01935800	-1.81099600
H	-5.27464100	0.73713600	-0.34953200

(TfO•1c)⊂AuCav

C	5.84128200	0.70883900	-1.45328700

C	4.71828900	0.20840300	-2.35788500
C	5.31457500	0.90719300	-0.03488900
H	6.11549500	1.69774600	-1.82190700
C	4.43890000	-1.15539000	-2.48502200
C	3.92712700	1.09211600	-3.10478000
C	5.34911000	-0.09145400	0.94358600
C	4.75001800	2.13614100	0.32719100
C	3.44427200	-1.66053800	-3.32524700
H	5.04834200	-1.85799200	-1.92895000
C	2.88827600	0.64191700	-3.91585200
O	4.26675100	2.44803500	-3.13546800
C	4.85906500	0.10465600	2.23978500
H	5.79076500	-1.04925100	0.69356100
C	4.22602200	2.37611200	1.59183300
O	4.83061000	3.16523900	-0.60234800
C	3.23407300	-3.16099600	-3.51189200
C	2.65796300	-0.72769100	-4.01809000
H	2.30115600	1.34081400	-4.49858800
C	3.44488600	3.35804800	-2.53359900
C	4.89932300	-0.98442500	3.31023800
C	4.28877000	1.35228100	2.53070600
H	3.80663400	3.34006200	1.84821300
C	3.71023400	3.69908300	-1.16093400
H	2.74682700	-3.28186900	-4.47988400
C	2.25875900	-3.69104400	-2.46910300
O	1.70491400	-1.19448000	-4.91933800
C	3.61004900	-1.79333000	3.23176200
H	4.88963800	-0.47436200	4.27395500
O	3.87224900	1.58228400	3.83628000
C	2.65377600	-4.03852600	-1.17225600
C	0.89574500	-3.78729400	-2.77661300
C	0.37686000	-1.10073400	-4.58355800
C	1.77394100	4.90475800	-2.57606100
C	3.45835600	-2.85326200	2.32980800
C	2.50223900	-1.46243800	4.02321800
C	2.54197800	1.73378200	4.11321200
C	1.99018300	5.18793900	-1.19123900
C	1.74262600	-4.42754700	-0.18464600
H	3.70549200	-3.97499800	-0.91512200
C	-0.04308900	-4.18024800	-1.83238400
O	0.46939900	-3.43939000	-4.05287200
C	-0.28004300	-2.28703600	-4.12346900
C	2.25938300	-3.55257400	2.15789900
H	4.31663100	-3.15218400	1.73940500
C	1.27595800	-2.09656200	3.85250500
O	2.62909200	-0.49228400	5.01618100
C	1.90472300	0.66040200	4.81818100
C	2.15376600	-4.77414300	1.24364900
C	0.39143900	-4.46898500	-0.54743400
H	-1.09649200	-4.19156200	-2.07979100
C	-1.59435300	0.03059100	-4.43110800
C	1.15869500	-3.10460400	2.90450900
H	0.42615100	-1.79499300	4.45093600
C	0.61485400	2.94001500	4.21723800
H	1.34029100	-5.37985300	1.65150300
O	-0.58892700	-4.66815600	0.42572200
C	-2.23662900	-1.13981000	-3.91563100
O	-0.11117000	-3.65905500	2.72432800
C	0.00412200	1.90044900	4.98540500
C	1.21590400	6.18050100	-0.54998200
C	0.81734300	5.65581600	-3.29459900
C	-3.59775900	-1.08312000	-3.54096100
C	-2.35073900	1.20596800	-4.62727500
C	-1.33970900	2.03733000	5.40150500
C	-0.14001000	4.08533300	3.87327100
C	3.42262400	-5.64062900	1.28242400
H	3.66135300	-5.91826700	2.31250200
H	4.29624500	-5.13271200	0.86510900
C	4.55063700	-3.95036300	-3.55093000
H	4.34707100	-5.00296700	-3.76580600
H	5.10344300	-3.91000200	-2.60796400
C	7.09787100	-0.17040300	-1.51178000
H	7.45819500	-0.24014300	-2.54153000
H	6.92797400	-1.19004700	-1.15467500
C	6.16476700	-1.84988200	3.25067700
H	7.05419800	-1.22122000	3.34540800
H	6.25845300	-2.41240900	2.31744400
H	6.16172000	-2.57333300	4.07041700
H	7.88846200	0.26561300	-0.89496000
H	5.20458500	-3.55572500	-4.33322900
H	3.26602500	-6.55274400	0.70077500
C	-1.45589600	4.18121100	4.27048200
C	-2.05799200	3.15388600	5.03646400
H	-3.10372100	3.23901100	5.31114900
H	-2.04890500	5.04160600	3.97939800
H	-1.78728200	1.22477800	5.96088300
H	0.34163700	4.85714400	3.28270700
C	0.10575600	6.64873100	-2.65475800
H	0.68271200	5.43555100	-4.34790200
C	0.29333300	6.90146400	-1.27589200
H	1.39059900	6.36349400	0.50442200
C	-3.68580700	1.22321800	-4.28690600
H	-1.84574200	2.06869900	-5.04453400
C	-4.30635800	0.08454200	-3.72282300
H	-4.05815600	-1.95536600	-3.09258300
H	-5.34276700	0.13251800	-3.41494900
H	-4.27206900	2.12407200	-4.43739200
H	-0.29699200	7.67052000	-0.78938200
H	-0.61405800	7.24108500	-3.21083100
P	-0.91700800	-3.30150200	1.32416500
N	-2.47663000	-3.38764300	1.79756900
C	-3.49840800	-2.76538600	0.93793200
C	-2.97029800	-4.36538500	2.76656600
H	-3.06722000	-1.97555000	0.32190200
H	-4.26633900	-2.30242000	1.55900300
H	-3.96756800	-3.50650900	0.28180000
H	-2.15876600	-4.70273900	3.41152600
H	-3.41732800	-5.23047800	2.26197700
H	-3.73618600	-3.88485800	3.38308100
Au	-0.12970000	-1.41392200	0.25075500
N	0.68556200	0.74557700	5.25984200
N	1.91526900	2.83080500	3.80336900
N	2.98497100	4.55072900	-0.49800600
N	2.50602800	3.94120000	-3.21920900
N	-0.25901600	0.02441800	-4.73375900
N	-1.53334400	-2.30379400	-3.77918700
C	1.09685500	0.52418000	-0.57491300
C	1.86314900	-0.29906700	-0.07935600
H	2.70837400	-0.82883000	0.30803400
C	0.40946900	1.65752500	-1.20547500
C	-0.91846400	2.10495700	-0.52798300
H	0.23372200	1.43491200	-2.26123700
H	1.11551500	2.49295300	-1.16307600
C	-2.01706900	1.07126100	-0.84613300
O	-1.75842800	-0.11849800	-1.04446600
O	-3.21312300	1.57351700	-0.87293900
H	-3.93068500	0.81032500	-0.80547000
C	-0.73955800	2.30479300	1.02332600
H	0.28941900	2.04307700	1.27934300
H	-0.84909600	3.37102900	1.21916500
C	-1.37812200	3.42612300	-1.17269500
O	-1.61885200	4.45200300	-0.57634000
O	-1.51173300	3.27770300	-2.50023200
C	-2.29115900	4.28133700	-3.17529000
H	-3.34334600	3.99542700	-3.10916600
H	-2.13537700	5.25519300	-2.71799400
H	-1.96061200	4.28296400	-4.21286500
S	-6.13718700	-0.40886400	0.00040300
O	-4.73229800	-0.35032900	-0.55662200
O	-6.92064900	0.81118500	-0.21872200
O	-6.21324700	-1.05873400	1.31969800
C	-6.85822800	-1.67733800	-1.14936800
F	-6.96844900	-1.18882300	-2.40058200
F	-6.06759600	-2.76806400	-1.20687600
F	-8.07311300	-2.05763500	-0.74239700
C	-1.66455200	1.49656700	1.90868500
C	-3.02710000	1.87767000	2.13393100
C	-1.18001800	0.35475200	2.51855900
C	-3.57757000	3.09801700	1.65762000
C	-3.87634400	1.00191600	2.88659200

C	-2.01256700	-0.49375300	3.28389000
H	-0.12302300	0.12007200	2.43738300
C	-4.89685400	3.41377800	1.88521100
H	-2.95699800	3.78108300	1.09409100
C	-5.23904200	1.34716600	3.08156600
C	-3.34260600	-0.19191800	3.43493000
H	-1.60144100	-1.38956900	3.73221600
C	-5.74367200	2.52513400	2.58730000
H	-5.29914700	4.34655700	1.50161900
H	-5.88197300	0.64328000	3.59825100
H	-4.00449300	-0.85009300	3.98994700
H	-6.79302600	2.76681900	2.71815600

1c-TS1$_{5\text{anti}}$⊂AuCav

C	-3.47496800	4.25561600	2.62193200
C	-3.41425200	2.78335600	3.01777400
C	-2.80613600	4.50189100	1.27251100
H	-2.87813900	4.79272200	3.35960700
C	-4.45003500	1.87824600	2.76345600
C	-2.29036800	2.30404200	3.70608100
C	-3.46033300	4.19819400	0.07510500
C	-1.51064400	5.03322000	1.17823700
C	-4.40260200	0.54993900	3.19533500
H	-5.33540400	2.23034700	2.24696800
C	-2.18605400	0.98375900	4.13822500
O	-1.33058900	3.23880500	4.06705100
C	-2.88600600	4.41068700	-1.18043500
H	-4.44674900	3.74904100	0.12608600
C	-0.91796200	5.30647800	-0.05305400
O	-0.78210400	5.27095900	2.34537100
C	-5.51962300	-0.46246200	2.95499700
C	-3.25479600	0.13365000	3.88000200
H	-1.31604600	0.64590000	4.68509800
C	-0.10052900	3.23796600	3.46665000
C	-3.57898600	3.99682000	-2.47011100
C	-1.60684800	4.97507500	-1.21741500
H	0.09137300	5.69433100	-0.10042100
C	0.20609900	4.34015200	2.60153000
H	-5.49157400	-1.16808800	3.78519800
C	-5.20065000	-1.25548000	1.69277400
O	-3.31275300	-1.15085900	4.41321000
C	-3.46200400	2.49269900	-2.68865800
H	-3.02380700	4.46473200	-3.28334200
O	-0.99894800	5.14204300	-2.46440200
C	-5.45890500	-0.69966500	0.43749000
C	-4.60977900	-2.52778200	1.72142700
C	-2.47696500	-2.15466300	4.05282800
C	1.99016100	2.39748600	3.15235300
C	-4.45854800	1.61149000	-2.26232400
C	-2.36576900	1.95004200	-3.38145100
C	0.05865800	4.28532400	-2.67763500
C	2.30779000	3.50940400	2.31132000
C	-5.16194400	-1.33568700	-0.76772900
H	-5.91658100	0.28119300	0.39966700
C	-4.26810300	-3.19133300	0.53984700
O	-4.38225400	-3.16588900	2.94201900
C	-3.07469900	-3.27080400	3.36358900
C	-4.44476400	0.24793200	-2.56077200
H	-5.30260700	2.01294600	-1.71577400
C	-2.31918800	0.59312400	-3.71435300
O	-1.41552000	2.82081400	-3.89300300
C	-0.17421600	3.02738700	-3.33227400
C	-5.53824100	-0.70717600	-2.10595800
C	-4.51332200	-2.57425900	-0.68358100
H	-3.80081200	-4.16763000	0.58628500
C	-0.50079200	-3.27474200	4.15194500
C	-3.37083600	-0.22896200	-3.31648800
H	-1.50336200	0.21072500	-4.31477000
C	2.26028400	3.75378700	-2.42003100
H	-5.57731300	-1.51737200	-2.83725800
O	-4.15691300	-3.25685200	-1.85697200
C	-1.11867100	-4.41945600	3.56284300
O	-3.42660400	-1.56269600	-3.76761900
C	2.03057900	2.48783800	-3.04901900
C	3.57252800	3.56665900	1.68317400
C	2.95552700	1.38513500	3.35986600
C	-0.34952500	-5.58251300	3.33184200
C	0.87336500	-3.31348500	4.47990100
C	3.10793700	1.58471300	-3.17200700
C	3.54037400	4.06779200	-1.90859200
C	-6.93491700	-0.06873000	-2.08287700
H	-7.18212800	0.32008000	-3.07419000
H	-7.01802800	0.75839600	-1.37368000
C	-6.92105300	0.16247700	2.92468000
H	-7.67747600	-0.61750000	2.80245400
H	-7.05561800	0.88070500	2.11105900
C	-4.89769100	4.83366900	2.66517700
H	-5.34133800	4.67552100	3.65223700
H	-5.56435100	4.38296500	1.92401600
C	-5.02664900	4.50636600	-2.53943700
H	-5.03248600	5.59837200	-2.49208600
H	-5.64717400	4.14364400	-1.71485500
H	-5.49864700	4.19667700	-3.47625400
H	-4.86835700	5.90797600	2.46502600
H	-7.11399400	0.68926900	3.86300800
H	-7.68229600	-0.81611800	-1.80451900
C	4.56150600	3.14662500	-2.00329000
C	4.34265400	1.90713100	-2.64779300
H	5.14529100	1.18339500	-2.68856700
H	5.53416900	3.33999700	-1.56372000
H	2.94983900	0.62562600	-3.64714100
H	3.66956800	5.03006200	-1.42525300
C	4.17931600	1.46312600	2.72809400
H	2.69071000	0.55407000	4.00523900
C	4.48594200	2.55240800	1.87427300
H	3.78174500	4.40776000	1.03280200
C	1.60447600	-4.45729400	4.23630900
H	1.31857200	-2.42635900	4.91662400
C	0.99018900	-5.59716200	3.66372400
H	-0.83971400	-6.44085700	2.88484200
H	1.58213700	-6.48907200	3.48429600
H	2.66003900	-4.48846000	4.48711500
H	5.43852900	2.57026700	1.35664700
H	4.92575000	0.68648300	2.85748000
P	-2.91960700	-2.80122900	-2.83034900
N	-3.01399700	-4.04988000	-3.94405300
C	-2.96294500	-5.41627900	-3.40645100
C	-2.19681200	-3.87341900	-5.15404300
H	-3.63900000	-5.50958600	-2.55755500
H	-1.94669800	-5.70747300	-3.10047800
H	-3.29652500	-6.10227400	-4.18942100
H	-2.32607600	-2.86575500	-5.54778800
H	-2.54754300	-4.58438900	-5.90658200
H	-1.12842900	-4.05550800	-4.96710800
Au	-0.84614600	-2.19894900	-2.06333800
N	0.77829600	2.15371900	-3.50276200
N	1.23322500	4.63390100	-2.24567400
N	1.37507200	4.47699300	2.05115700
N	0.75662200	2.29705300	3.73262700
N	-1.22805200	-2.14663400	4.41181200
N	-2.42742000	-4.37667900	3.15329100
C	1.89446800	-2.14099500	-1.98351700
C	1.08067000	-1.33557300	-2.46490600
H	0.98179900	-0.38966700	-2.97813200
C	2.76397900	-3.07907000	-1.28614700
C	3.66282400	-2.31863100	-0.27640400
H	3.40135800	-3.59676800	-2.00494200
H	2.15882900	-3.83224400	-0.77609000
C	4.21923000	-1.08790200	-1.05703700
O	4.06892300	-1.08298500	-2.27379200
O	4.74368000	-0.09284700	-0.40496900
H	5.46203100	-0.20127600	0.37099100
C	2.94143700	-1.91315200	1.05651800
H	3.42710900	-1.01989500	1.44802000
H	3.12095500	-2.70470000	1.78833100
C	4.87479100	-3.20368300	0.04460700
O	5.16156600	-4.22835200	-0.53694700
O	5.57894300	-2.65565000	1.03529600
C	6.85708900	-3.24638800	1.34593900
H	7.34359300	-2.52583700	1.99874500

H	6.71066300	-4.20934800	1.84080000
H	7.43472200	-3.38314800	0.43204300
S	7.66956400	0.82177400	0.60970200
O	8.81117700	0.99438100	1.50705600
O	7.12736900	2.00456700	-0.08142100
O	6.58289300	-0.06400400	1.20987600
C	8.29090700	-0.25795600	-0.76975300
F	9.21779400	0.38283900	-1.48626100
F	7.28557700	-0.61380200	-1.59110300
F	8.83544500	-1.38485500	-0.27578500
C	1.44631500	-1.73475000	0.94499200
C	0.82957700	-0.47474900	0.65705500
C	0.65438800	-2.86035700	1.08069800
C	1.56193800	0.70266600	0.35954400
C	-0.60374100	-0.40955000	0.59625800
C	-0.74750200	-2.80897200	0.93221700
H	1.11833000	-3.81382400	1.31330600
C	0.90504700	1.86981000	0.03852900
H	2.64368000	0.69181200	0.36609900
C	-1.24991900	0.81596200	0.30519100
C	-1.36804500	-1.59404600	0.73575200
H	-1.33117300	-3.71626700	1.00707800
C	-0.50572500	1.93347400	0.02403100
H	1.47939400	2.75630100	-0.18840000
H	-2.33555900	0.84800500	0.28443700
H	-2.44382200	-1.51937500	0.66748000
H	-0.99914800	2.85906100	-0.21804100

1c-Int$_{5anti}$⊂AuCav

C	-3.51276900	3.68889600	3.14771300
C	-2.76199600	2.43610900	3.58497100
C	-3.51868800	3.79573800	1.62757300
H	-2.93718100	4.53885400	3.51476500
C	-3.39035300	1.22426200	3.87842800
C	-1.36814000	2.49577800	3.72395700
C	-4.42428400	3.05079900	0.86902300
C	-2.63475200	4.63320400	0.93264000
C	-2.68244200	0.11037100	4.33848000
H	-4.46733200	1.15569300	3.77877800
C	-0.61532300	1.41202600	4.16687200
O	-0.80280400	3.75093600	3.56243800
C	-4.49988800	3.12032500	-0.52217500
H	-5.10836300	2.39071700	1.39029100
C	-2.66435800	4.72453500	-0.46001200
O	-1.74533800	5.44060200	1.64570500
C	-3.34700600	-1.22244500	4.66883500
C	-1.29582400	0.23838500	4.48078000
H	0.45543300	1.49265600	4.30241200
C	0.05546900	4.09578700	2.56199100
C	-5.55163600	2.33856400	-1.29803600
C	-3.58200100	3.95498500	-1.17252900
H	-1.97779000	5.38144500	-0.97910000
C	-0.41598700	5.08125300	1.62491900
H	-2.71715300	-1.71285800	5.41081000
C	-3.32713500	-2.10383800	3.42549300
O	-0.64268000	-0.82744100	5.09362900
C	-5.12276900	0.89043400	-1.48689200
H	-5.59051200	2.77987100	-2.29391600
O	-3.62870800	4.04338700	-2.56924400
C	-4.29322800	-1.95431700	2.42787900
C	-2.32613300	-3.06107000	3.21017400
C	0.30046300	-1.57010300	4.45407800
C	2.13595300	4.21902700	1.65195400
C	-5.43962700	-0.11660300	-0.57295400
C	-4.39078700	0.52635000	-2.62543100
C	-2.63723300	3.38299500	-3.25350400
C	1.69654900	5.28134000	0.80288300
C	-4.30729500	-2.70232700	1.25014400
H	-5.07591000	-1.22125900	2.58035300
C	-2.28358800	-3.81536700	2.03648100
O	-1.37928400	-3.29574800	4.20572100
C	-0.08011500	-2.88729300	4.01194400
C	-5.09101500	-1.45455200	-0.77644200
H	-5.99626900	0.14624700	0.31849200
C	-4.02594700	-0.79476200	-2.87499300
O	-4.13512400	1.52311300	-3.56145500
C	-2.87569200	2.03887000	-3.71420500
C	-5.42697300	-2.55235600	0.22402000
C	-3.25461500	-3.60893900	1.06040400
H	-1.49130300	-4.53944300	1.88814500
C	2.44882900	-1.97858200	3.82878500
C	-4.39591400	-1.76721100	-1.94921100
H	-3.47924000	-1.05228400	-3.77297700
C	-0.54690800	3.30938100	-4.16284700
H	-5.46388700	-3.48856500	-0.33729500
O	-3.20886000	-4.39374300	-0.09462400
C	2.08129200	-3.29630900	3.41623100
O	-4.11709500	-3.11501400	-2.21966300
C	-0.76537300	1.95999200	-4.57326900
C	2.62007600	5.90171600	-0.06872500
C	3.47438500	3.77364100	1.57395200
C	3.06625300	-4.15311600	2.87422900
C	3.79048400	-1.55450800	3.69827300
C	0.27191600	1.25553300	-5.22684800
C	0.70199200	3.92164500	-4.41346300
C	-6.80630100	-2.37113900	0.87476700
H	-7.57957100	-2.33624600	0.10269800
H	-6.88703600	-1.45320500	1.46253500
C	-4.74925500	-1.07506700	5.27485800
H	-5.14981200	-2.05821000	5.53696600
H	-5.46434700	-0.59997500	4.59740800
C	-4.92317200	3.79340600	3.74569300
H	-4.87345400	3.74907300	4.83732300
H	-5.59182200	2.99468200	3.41203900
C	-6.95061500	2.47346900	-0.67706700
H	-7.23909500	3.52721200	-0.63786300
H	-7.00627800	2.08224000	0.34263700
H	-7.68541500	1.93335000	-1.28084400
H	-5.38066500	4.74336200	3.45652700
H	-4.70175300	-0.46591500	6.18160400
H	-7.01796900	-3.20941300	1.54372800
C	1.70514200	3.20447600	-5.02891300
C	1.49025500	1.86604500	-5.43412400
H	2.29631800	1.31490000	-5.90635800
H	2.67274900	3.66461400	-5.20139300
H	0.08412700	0.23422900	-5.53821900
H	0.84337800	4.94643400	-4.08757000
C	4.35380900	4.38689400	0.70689400
H	3.77956900	2.93898400	2.19413000
C	3.92743400	5.46239100	-0.10779500
H	2.26374200	6.71029400	-0.69809600
C	4.73286800	-2.41258500	3.17292100
H	4.03985000	-0.54663000	4.01138800
C	4.36868700	-3.71439500	2.75392200
H	2.76365800	-5.14401500	2.55331500
H	5.11775400	-4.35817300	2.30813900
H	5.75698500	-2.08006200	3.05397400
H	4.63745500	5.93579800	-0.77899300
H	5.37455600	4.02863200	0.63862300
P	-2.76833000	-3.79580300	-1.56709700
N	-2.79301500	-5.25765300	-2.40687400
C	-1.89120500	-6.29564400	-1.89238000
C	-2.74458600	-5.13846100	-3.87076200
H	-2.00652300	-6.38821900	-0.81276300
H	-0.83579700	-6.09103500	-2.13035700
H	-2.16902400	-7.24936400	-2.34996600
H	-3.46234000	-4.39057500	-4.20657300
H	-3.02675400	-6.10380500	-4.30072500
H	-1.74324000	-4.86794200	-4.23689300
Au	-0.97683100	-2.39687900	-1.96408200
N	-1.96816900	1.35026100	-4.34145800
N	-1.51742600	3.99871800	-3.49063400
N	0.38533100	5.67583600	0.79389100
N	1.27728500	3.65371200	2.55530900
N	1.51868100	-1.12862600	4.35740400
N	0.78116100	-3.72234100	3.51106800
C	1.79635500	-1.47311800	-2.62673200
C	0.50079700	-1.24588900	-2.76946900
H	0.23205200	-0.33021700	-3.28616100
C	2.59064300	-2.51482400	-1.89853700

C	3.74600000	-1.67123400	-1.30463700
H	2.99033100	-3.26060200	-2.59764200
H	2.00249700	-3.02670800	-1.13982700
C	3.86079500	-0.55989700	-2.35212100
O	2.76271900	-0.50493200	-3.09316000
O	4.77086300	0.24456800	-2.53192300
H	6.02980600	0.31989200	-1.83028200
C	3.37808500	-1.08234400	0.10712300
H	4.08324500	-0.28592000	0.34859000
H	3.56670200	-1.90024300	0.80103800
C	4.99423100	-2.53471200	-1.16037700
O	5.09751800	-3.41175800	-0.33050300
O	5.92954600	-2.25716900	-2.08025800
C	7.15439800	-3.00805400	-1.97753800
H	7.75782600	-2.69345100	-2.82721000
H	7.65765700	-2.76499100	-1.04061800
H	6.94892100	-4.07947500	-2.01515900
S	6.94985600	1.14340900	0.00080700
O	5.57568500	1.41215800	0.42797900
O	7.97608100	2.16526400	0.10812200
O	6.98240400	0.46082200	-1.41247800
C	7.51997200	-0.29356500	1.02758600
F	7.53074000	0.05379000	2.31529600
F	8.73920900	-0.68412100	0.65770500
F	6.67010400	-1.32169100	0.86773200
C	1.94675000	-0.62832500	0.28032800
C	1.48914200	0.65621300	-0.15181900
C	1.03864000	-1.50530300	0.83969300
C	2.34135600	1.61351300	-0.76122300
C	0.09773300	0.97985100	-0.01760400
C	-0.32180400	-1.16728100	1.00679100
H	1.37571000	-2.48776500	1.14539200
C	1.83835100	2.78651800	-1.27116700
H	3.40566900	1.43484000	-0.81538400
C	-0.38008600	2.21679200	-0.51394100
C	-0.78779700	0.05502200	0.58681300
H	-0.99881100	-1.87909000	1.45142800
C	0.46711400	3.09747700	-1.14190300
H	2.50625800	3.49737700	-1.74190900
H	-1.43265300	2.44579600	-0.39595500
H	-1.83427500	0.32288300	0.70472200
H	0.08985300	4.03918000	-1.51712700

1c-TS2$_{5anti}$⊂AuCav

C	-4.13683600	1.10097500	3.87065900
C	-2.64119700	0.92544600	4.08927400
C	-4.54903300	0.75905000	2.44606800
H	-4.33817800	2.16477400	3.99578300
C	-2.07839900	-0.27743600	4.51196400
C	-1.77952600	2.01626200	3.92428600
C	-4.85216600	-0.54881200	2.07157100
C	-4.70236100	1.75810000	1.47504200
C	-0.72493500	-0.41212200	4.83276900
H	-2.72480200	-1.13916300	4.62801500
C	-0.42146600	1.93443800	4.22047900
O	-2.36705500	3.23738100	3.61093800
C	-5.36794500	-0.88595500	0.81910900
H	-4.70841200	-1.33912500	2.79870900
C	-5.20724100	1.47103900	0.20626800
O	-4.49613700	3.08378100	1.85721200
C	-0.15703300	-1.75505800	5.28389800
C	0.08636500	0.72238900	4.68900100
H	0.21736100	2.80420000	4.13069300
C	-2.24672100	3.82811700	2.38609800
C	-5.67071700	-2.33865500	0.47041500
C	-5.55274700	0.15361900	-0.10160600
H	-5.36500000	2.26607200	-0.51210300
C	-3.37492300	3.78020600	1.48807200
H	0.77524400	-1.54482800	5.80741500
C	0.20197100	-2.55147500	4.03480900
O	1.40929000	0.66632900	5.14552500
C	-4.36331600	-2.98369200	0.03056100
H	-6.33127700	-2.32745600	-0.39619400
O	-6.21174300	-0.12584300	-1.30856200
C	-0.71081300	-3.36376300	3.36092200
C	1.47428700	-2.40601400	3.46805000
C	2.44138400	0.70513000	4.24956800
C	-1.19220000	5.27910000	0.98214900
C	-3.49803500	-3.63300900	0.91010100
C	-3.95957300	-2.87358600	-1.30591000
C	-5.51676700	-0.07516600	-2.48508200
C	-2.33014700	5.27533300	0.12137200
C	-0.39803300	-4.00843700	2.16037000
H	-1.69778700	-3.50425100	3.78541700
C	1.81939400	-2.97974900	2.24857000
O	2.40331700	-1.69102300	4.21144900
C	2.92943700	-0.53771800	3.70968500
C	-2.27191300	-4.16675500	0.50341000
H	-3.79623700	-3.74682400	1.94524900
C	-2.72849000	-3.34475500	-1.75070400
O	-4.90735200	-2.36985400	-2.18684200
C	-4.74124200	-1.22104700	-2.89673900
C	-1.37582700	-4.94105900	1.45982000
C	0.86514300	-3.76475700	1.60903400
H	2.79588200	-2.81972900	1.80634500
C	4.06286500	1.81184200	3.09116100
C	-1.89236800	-3.98182000	-0.83329900
H	-2.44492800	-3.25062300	-2.79149300
C	-4.90843600	0.99236700	-4.40459200
H	-0.77504500	-5.61349600	0.84288200
O	1.23427800	-4.40092900	0.42494100
C	4.47682400	0.58641200	2.48237200
O	-0.70744200	-4.54232400	-1.30164700
C	-4.03664400	-0.08774700	-4.73948300
C	-2.33355400	6.09675000	-1.02961700
C	-0.07572100	6.08676600	0.65982700
C	5.53189100	0.58360800	1.54376500
C	4.76062800	3.00148900	2.78831700
C	-3.22721600	0.00140900	-5.89408900
C	-4.97931200	2.11938700	-5.25521400
C	-2.16385500	-5.82142300	2.44091500
H	-2.81129100	-6.50900600	1.89014200
H	-2.79741200	-5.24586700	3.12066500
C	-1.07451700	-2.51325400	6.25157800
H	-0.59281900	-3.44056800	6.57413900
H	-2.03938400	-2.78055800	5.81168600
C	-4.97769300	0.35148800	4.91645800
H	-4.69891600	0.67687600	5.92251700
H	-4.84942500	-0.73373400	4.87443800
C	-6.38881800	-3.09724200	1.59362300
H	-7.33393400	-2.60237000	1.83227100
H	-5.80707600	-3.15235300	2.51788100
H	-6.60327300	-4.12197400	1.27724800
H	-6.03954000	0.56243000	4.76308200
H	-1.27580700	-1.90077000	7.13452600
H	-1.47312500	-6.40661700	3.05383900
C	-4.18212900	2.18044500	-6.37913100
C	-3.29744700	1.12206300	-6.69488400
H	-2.67240900	1.19476300	-7.57908600
H	-4.22632600	3.05004300	-7.02709000
H	-2.56695800	-0.82825000	-6.12228200
H	-5.65187400	2.92474200	-4.98068700
C	-0.09315100	6.85673600	-0.48410400
H	0.77519300	6.08025600	1.33221500
C	-1.23049500	6.86760000	-1.32759000
H	-3.21333600	6.07968100	-1.66321900
C	5.81883100	2.96574900	1.90436700
H	4.43924400	3.92371400	3.25835000
C	6.19159600	1.76155400	1.26243900
H	5.79890500	-0.35770100	1.08237400
H	7.00127300	1.76284200	0.54016800
H	6.35078900	3.88321100	1.68094000
H	-1.22648400	7.48726600	-2.21861500
H	0.77006000	7.46160600	-0.74093100
P	0.78963200	-3.84813600	-1.05575500
N	1.80049800	-4.75684000	-1.98424400
C	2.39019300	-6.03011800	-1.57465400
C	2.00524500	-4.42205700	-3.39341900
H	2.22251000	-6.20323200	-0.51377000
H	3.46858600	-5.98400300	-1.75549300

H	1.95800100	-6.85952100	-2.14873900
H	1.61286900	-3.42619000	-3.60999600
H	1.50007000	-5.14868200	-4.04236100
H	3.07764200	-4.41476000	-3.60454400
Au	1.04027900	-1.62721700	-1.57297200
N	-4.00138100	-1.21563700	-3.96526500
N	-5.62113500	0.98140000	-3.23518100
N	-3.41227500	4.48820500	0.39701500
N	-1.18494200	4.53477800	2.12763700
N	3.00133500	1.84273000	3.95864700
N	3.88586200	-0.59469000	2.83442300
C	2.25627500	1.17088000	-2.27851700
C	1.74136800	0.00576200	-2.72269800
H	1.17517900	0.11933100	-3.64840300
C	3.20849700	1.48525600	-1.15468100
C	2.91238700	2.96780600	-0.87008100
H	4.22725800	1.36592500	-1.53172600
H	3.08464100	0.82082100	-0.30382500
C	2.38522000	3.43907100	-2.23891400
O	2.01805800	2.34180100	-2.97822500
O	2.28694100	4.56004300	-2.65569000
H	2.94506900	-0.82647300	-2.78546400
C	1.85385700	3.16077700	0.26093500
H	1.46741400	4.17732700	0.19331100
H	2.39788000	3.10030200	1.20486400
C	4.11314900	3.87382300	-0.61132200
O	4.11862100	4.81555100	0.14660400
O	5.14022100	3.54614600	-1.42032700
C	6.25066300	4.45705300	-1.42343800
H	6.89202700	4.13366300	-2.24230600
H	5.90060600	5.47892400	-1.58168200
H	6.79104300	4.40581900	-0.47597100
S	4.58880300	-2.12418200	-1.72131400
O	4.83242100	-3.54709500	-1.94934200
O	3.95059600	-1.69724500	-0.46732900
O	3.97934200	-1.42814400	-2.96005100
C	6.27348800	-1.33264200	-1.70429800
F	6.94669100	-1.75706300	-0.62599900
F	6.16645100	0.00619500	-1.63160100
F	6.95657700	-1.65057500	-2.80176600
C	0.74815300	2.13171600	0.25938200
C	-0.35533300	2.21937100	-0.64602900
C	0.83115300	1.04251600	1.10595700
C	-0.52372100	3.30612500	-1.54105000
C	-1.31612400	1.15498000	-0.68043300
C	-0.13165100	0.00805000	1.09720300
H	1.66794700	0.97429000	1.78762200
C	-1.54861300	3.32135100	-2.45583400
H	0.14870800	4.15017200	-1.50069800
C	-2.37392200	1.21608600	-1.61930700
C	-1.18535300	0.05758800	0.21155500
H	-0.03688600	-0.82050300	1.78808900
C	-2.48691200	2.27141500	-2.49505800
H	-1.64908200	4.16141100	-3.13394300
H	-3.09013500	0.40586700	-1.62649600
H	-1.92901100	-0.73379500	0.18832800
H	-3.29871300	2.31491700	-3.20495700

(TfO•2c)⊂AuCav

C	1.43711900	5.34317000	1.68795800
C	0.05094500	4.93259700	1.21079300
C	2.48513500	4.26177100	1.45810700
H	1.35959800	5.45783500	2.76937800
C	-0.40740900	5.16914600	-0.08677700
C	-0.83895800	4.33091200	2.10940200
C	3.15132000	4.13901500	0.23784900
C	2.86892000	3.39151100	2.48796600
C	-1.71560000	4.88161300	-0.48500700
H	0.27464300	5.60316200	-0.80878600
C	-2.15863400	4.05005000	1.77091000
O	-0.39551000	4.13678700	3.41030700
C	4.21557700	3.25810800	0.03436700
H	2.83857800	4.77066200	-0.58570100
C	3.93463200	2.50670000	2.33845200
O	2.24368400	3.50176800	3.73021300
C	-2.18771900	5.03828400	-1.92851200
C	-2.57894200	4.34989100	0.47984200
H	-2.84046800	3.63241500	2.50057200
C	-0.08973100	2.88116400	3.86210300
C	4.87298300	3.11119700	-1.33346100
C	4.61512500	2.47055000	1.12231300
H	4.24488800	1.87843600	3.16426400
C	1.29694500	2.57049100	4.08121000
H	-3.27324100	5.13431500	-1.90375200
C	-1.86918100	3.72504500	-2.63487600
O	-3.92347000	4.22911600	0.13059900
C	4.02513600	2.14138700	-2.14927900
H	5.83821900	2.63176800	-1.17142900
O	5.76991800	1.69288500	0.99822400
C	-0.63331900	3.49761800	-3.24071500
C	-2.78155100	2.66054700	-2.62713300
C	-4.56664700	3.03141000	0.10993500
C	-0.68768100	0.90258500	4.81259500
C	2.96808900	2.56093300	-2.95744800
C	4.25588700	0.76127600	-2.04715800
C	5.61085600	0.33075800	1.03287000
C	0.68465300	0.62483800	5.09414600
C	-0.26872400	2.27264800	-3.80461700
H	0.07282600	4.31675400	-3.29262200
C	-2.43332700	1.39744700	-3.09945300
O	-4.09396600	2.92682500	-2.25081600
C	-4.68053800	2.35470600	-1.15814400
C	2.15941600	1.66193000	-3.65637500
H	2.77558600	3.62259000	-3.05625000
C	3.45230400	-0.17536200	-2.69359300
O	5.38609400	0.37145200	-1.34808600
C	5.30542700	-0.35651500	-0.19146900
C	1.03460800	2.11157000	-4.57464400
C	-1.16956200	1.21065900	-3.65637900
H	-3.15156900	0.58675600	-3.08094300
C	-5.97287300	1.50247000	1.04517900
C	2.41302900	0.29812800	-3.48907300
H	3.64798500	-1.23592800	-2.58956000
C	5.55911300	-1.65474100	2.14299000
H	0.87380400	1.30038000	-5.28871600
O	-0.89987500	-0.04134100	-4.21143400
C	-6.12347200	0.86210500	-0.22271300
O	1.64793200	-0.61746200	-4.21950100
C	5.14626600	-2.31961900	0.94504300
C	1.02111700	-0.53009300	5.83570900
C	-1.68589600	0.00157000	5.25029300
C	-7.03598900	-0.20882300	-0.35710400
C	-6.71447700	1.03758600	2.15429600
C	4.83526600	-3.69576100	0.97892700
C	5.70519100	-2.39836700	3.33573600
C	1.39822900	3.35665400	-5.39739200
H	2.29151700	3.15624600	-5.99477800
H	1.60868000	4.23298700	-4.77934400
C	-1.62322900	6.27650700	-2.63382300
H	-2.00983600	6.33846800	-3.65489200
H	-0.53136300	6.27862900	-2.69417900
C	1.87548500	6.70007100	1.11340300
H	1.13605100	7.46773000	1.35823400
H	1.99077300	6.68762600	0.02586000
C	5.12647500	4.45080200	-2.03590000
H	5.77636400	5.07707300	-1.41898000
H	4.21104200	5.01868700	-2.22509100
H	5.61683700	4.28473800	-2.99931500
H	2.83800400	6.99325000	1.54113500
H	-1.92108000	7.17947100	-2.09437400
H	0.57736600	3.61719700	-6.07097900
C	5.40702400	-3.74582600	3.33922100
C	4.95522500	-4.39009200	2.16370800
H	4.67775500	-5.43759100	2.19812400
H	5.50093000	-4.31680400	4.25782100
H	4.46619300	-4.16148900	0.07370400
H	6.02609800	-1.87415800	4.22931800
C	-1.32962600	-1.11500900	5.97714200
H	-2.72058100	0.23073300	5.01932200
C	0.02703600	-1.37536100	6.28020500

H	2.06996700	-0.71848200	6.03527300
C	-7.60709600	-0.00003000	1.99409500
H	-6.56962500	1.52989400	3.10911400
C	-7.77688600	-0.61597100	0.73248100
H	-7.14980600	-0.66705000	-1.33361800
H	-8.49938700	-1.41796400	0.62159800
H	-8.18241500	-0.35285500	2.84319000
H	0.28539100	-2.25980800	6.85380500
H	-2.09617700	-1.80199200	6.32131600
P	0.18185400	-1.15765300	-3.69074400
N	-0.17982400	-2.36478800	-4.76801100
C	-0.37509300	-2.05213600	-6.18755600
C	0.41450200	-3.68719900	-4.53289500
H	-0.88962500	-1.09833500	-6.29480900
H	-0.99796000	-2.83487500	-6.63057400
H	0.58005100	-2.01394200	-6.72788800
H	0.52099900	-3.88231200	-3.46568600
H	1.40240400	-3.77364100	-5.00355100
H	-0.24837900	-4.44733700	-4.95684800
Au	0.27597900	-1.87770800	-1.57983800
N	5.05712900	-1.62956800	-0.23409300
N	5.75957600	-0.30090800	2.15894800
N	1.67001200	1.47797900	4.67951300
N	-1.04747500	2.06251700	4.18417700
N	-5.16542000	2.60014800	1.18068000
N	-5.42604700	1.30121700	-1.31711500
C	-2.97048800	-2.68955700	-1.24558100
C	-2.83375700	-2.94812700	-2.54296900
H	-3.22757500	-2.25758600	-3.27805900
C	-3.61676300	-1.51084200	-0.56195800
C	-3.47871500	-1.80332400	0.94484300
H	-4.65341600	-1.40050400	-0.87562500
H	-3.08480500	-0.59428100	-0.82141200
C	-2.67085500	-3.12230400	0.97227700
O	-2.45601400	-3.56126400	-0.30128400
O	-2.34214000	-3.75300100	1.93820800
H	-2.32320100	-3.83815800	-2.88478100
C	-2.85456600	-0.65863900	1.78404000
H	-2.79297500	-1.00223600	2.81618300
H	-3.58545100	0.15452900	1.78449800
C	-4.79856900	-2.22445300	1.60448600
O	-5.19255500	-1.85243800	2.68439200
O	-5.42558800	-3.14940200	0.84711000
C	-6.53675300	-3.81256000	1.47182500
H	-6.95887000	-4.46278700	0.70659600
H	-6.18599400	-4.40052900	2.32357000
H	-7.27344000	-3.08659300	1.81636300
C	-1.52311600	-0.12289900	1.30977700
C	-0.28676600	-0.65152200	1.80229100
C	-1.49106800	0.93083600	0.41314800
C	-0.22151200	-1.71770800	2.73685100
C	0.94622400	-0.08163200	1.33536800
C	-0.27897200	1.48895900	-0.05041600
H	-2.42610600	1.36055900	0.07310600
C	0.98530800	-2.21467000	3.16097200
H	-1.12947600	-2.17684000	3.09546900
C	2.17284300	-0.60690300	1.81539400
C	0.92016700	0.99022100	0.40286000
H	-0.30257700	2.31729000	-0.74728900
C	2.19566100	-1.65688900	2.70085500
H	1.00983500	-3.05372500	3.84603600
H	3.09159000	-0.15836800	1.46325000
H	1.85967200	1.41187000	0.05872800
H	3.13532100	-2.06097600	3.05179600
S	1.37058100	-4.30428400	0.02845600
O	1.84443900	-4.75843800	1.33311300
O	0.47169200	-3.06438500	0.14578100
O	2.32574900	-4.19787000	-1.08904400
C	0.16595400	-5.60953800	-0.52771300
F	0.82919800	-6.75100700	-0.75108700
F	-0.42296100	-5.23681200	-1.68595300
F	-0.77496900	-5.82267700	0.38478700

1c-TS1$_{5syn}$⊂AuCav

C	4.49915400	3.06051900	2.46437900
C	3.75447500	3.43488700	1.18785800
C	4.09646000	1.65226600	2.88693400
H	4.13911000	3.73339900	3.24294400
C	4.26165300	3.11520800	-0.07397200
C	2.52965100	4.11360700	1.23198000
C	4.76882700	0.50709300	2.44814200
C	2.99864700	1.46732200	3.73754900
C	3.62504200	3.46462300	-1.26669700
H	5.21356400	2.60079800	-0.12968900
C	1.82964200	4.43944500	0.07360500
O	2.07079000	4.58496300	2.46522500
C	4.39988700	-0.78272300	2.84185000
H	5.62548300	0.62733800	1.79561900
C	2.56814900	0.20449400	4.13121900
O	2.41627200	2.60686100	4.28115900
C	4.26830200	3.19149600	-2.62540900
C	2.38425900	4.11308000	-1.16246600
H	0.89475100	4.98335400	0.12571700
C	0.95761000	4.04312700	3.03945500
C	5.16589200	-2.03058600	2.40428700
C	3.27869500	-0.90212300	3.67556100
H	1.72628900	0.08586700	4.80087500
C	1.13681700	2.96572200	3.97822200
H	3.83032700	3.91081700	-3.31813500
C	3.87438200	1.81015600	-3.13399600
O	1.75474400	4.54684000	-2.32476700
C	4.56446200	-2.57141800	1.11277800
H	4.98700900	-2.78600800	3.17012200
O	2.95703600	-2.17278900	4.13602500
C	4.53561600	0.63828500	-2.74774800
C	2.76432900	1.67845100	-3.97842200
C	0.64601000	3.86803900	-2.77072800
C	-1.27839700	4.03899600	3.48340500
C	4.92931000	-2.06484100	-0.13918000
C	3.57953600	-3.56780000	1.13923500
C	1.81620600	-2.79167900	3.70983600
C	-1.10509300	2.93680400	4.37862200
C	4.09581500	-0.63676800	-3.12446600
H	5.40313700	0.71963000	-2.10227700
C	2.29586100	0.43588300	-4.38340500
O	2.09051100	2.82341800	-4.38110400
C	0.82004800	2.96430900	-3.86617500
C	4.32556700	-2.46140000	-1.33666700
H	5.71027600	-1.31497500	-0.18273700
C	2.93034800	-3.97521500	-0.02157800
O	3.24210500	-4.17324500	2.34836000
C	1.97819100	-3.90980400	2.82439300
C	4.78970800	-1.92982400	-2.69514000
C	2.94854700	-0.69750900	-3.92491000
H	1.39687300	0.35983700	-4.98065700
C	-1.58776200	3.42293800	-2.77581200
C	3.27894100	-3.39102800	-1.23216900
H	2.14475100	-4.71726400	0.03466200
C	-0.42429000	-3.18419000	3.79148700
H	4.49238700	-2.68515600	-3.42729100
O	2.32458400	-1.92848100	-4.16442800
C	-1.40443200	2.48714300	-3.84247300
O	2.48429200	-3.71053800	-2.34685300
C	-0.25043500	-4.34828300	2.97954100
C	-2.22491200	2.40210200	5.05563900
C	-2.56627100	4.59498800	3.30925700
C	-2.51345600	1.76610800	-4.33766200
C	-2.88718200	3.64539000	-2.27108500
C	-1.36973700	-5.14965600	2.66319100
C	-1.71787000	-2.83702200	4.24266200
C	6.32046700	-1.80968900	-2.77148300
H	6.78832100	-2.76560700	-2.52162100
H	6.72006600	-1.05651800	-2.08732400
C	5.78787600	3.41286200	-2.62305700
H	6.18362700	3.28758900	-3.63470200
H	6.32252700	2.71358800	-1.97373400
C	6.01838600	3.24913000	2.35700100
H	6.24829900	4.28556800	2.09609400
H	6.48015400	2.61055700	1.59882100
C	6.68174200	-1.80695100	2.31501300

H	7.06722800	-1.45718300	3.27643300
H	6.96379800	-1.06818300	1.55956300
H	7.18307300	-2.74451500	2.05987800
H	6.49305000	3.01776300	3.31453200
H	6.02193600	4.42450400	-2.28085100
H	6.62086400	-1.52576200	-3.78339800
C	-2.79499600	-3.62852300	3.90640100
C	-2.61929700	-4.79094500	3.11959300
H	-3.48458600	-5.38746400	2.85342600
H	-3.79226400	-3.35240900	4.23052000
H	-1.21756800	-6.01662800	2.03166900
H	-1.82611200	-1.94425300	4.84884500
C	-3.63525400	4.07310000	4.00585700
H	-2.67408000	5.43273000	2.62915700
C	-3.46761300	2.96698600	4.87138400
H	-2.06637700	1.55935700	5.71957700
C	-3.95432000	2.93291300	-2.77491800
H	-3.00133000	4.36987900	-1.47427800
C	-3.76555600	1.97858700	-3.80316600
H	-2.34289300	1.04722900	-5.13189000
H	-4.61953200	1.39988400	-4.13449600
H	-4.95250200	3.07819200	-2.37497800
H	-4.33092700	2.55878900	5.38526800
H	-4.62285300	4.50541000	3.88476700
P	1.48511800	-2.48801500	-2.84220700
N	0.16295300	-3.27932200	-3.39840400
C	-1.10658600	-2.54229500	-3.48954300
C	0.23089700	-4.54025700	-4.13369300
H	-1.07367200	-1.63643200	-2.88043200
H	-1.91105200	-3.17538900	-3.10644700
H	-1.31938700	-2.26069100	-4.52731000
H	1.16860800	-5.05180700	-3.91433900
H	0.15319200	-4.37513000	-5.21513300
H	-0.59882900	-5.17721600	-3.81128000
Au	1.29043800	-0.85145000	-1.21862500
N	0.98313400	-4.67056100	2.47948000
N	0.65252000	-2.41963200	4.15445300
N	0.14003300	2.41256500	4.60473300
N	-0.21226100	4.56040100	2.79972500
N	-0.52066300	4.09293600	-2.24024800
N	-0.16329100	2.28468400	-4.37629400
C	0.17915500	1.05365000	0.56951100
C	1.30178000	0.50780900	0.41075900
H	2.20476400	0.54820300	1.00870400
C	-1.02738100	1.69831500	1.06095300
C	-2.28467100	0.81081700	0.83580300
H	-1.16442000	2.67297400	0.58509800
H	-0.88360700	1.84685300	2.13603000
C	-2.21337400	0.44856400	-0.66724500
O	-1.09563700	0.56261200	-1.19474700
O	-3.21221600	-0.03998400	-1.31343200
H	-4.16736000	0.07672200	-0.88945200
C	-2.20953500	-0.47836600	1.71209600
H	-1.17042200	-0.80545400	1.74580700
H	-2.47877700	-0.16357100	2.72247300
C	-3.51466500	1.64192800	1.25309700
O	-4.16024400	1.43772800	2.25697600
O	-3.70198500	2.66333200	0.41837100
C	-4.88127500	3.47236900	0.63852600
H	-5.74286000	2.96632300	0.20102300
H	-5.02636100	3.63200100	1.70504200
H	-4.68724300	4.41492400	0.12746100
S	-6.55674700	0.63832700	-1.38418300
O	-6.22366800	0.23036600	-2.75577700
O	-7.16419200	1.96627900	-1.19417700
O	-5.45333700	0.35790100	-0.38193300
C	-7.84672600	-0.56949100	-0.81913000
F	-7.44172800	-1.82720600	-1.05167100
F	-8.99420800	-0.36226800	-1.47371400
F	-8.07194200	-0.43747400	0.49801100
C	-3.11795300	-1.60807500	1.27773200
C	-2.61882900	-2.71436900	0.51752100
C	-4.45720300	-1.56981000	1.61202300
C	-1.24629300	-2.88829300	0.19527000
C	-3.53690400	-3.73570100	0.09698900
C	-5.35905800	-2.56648300	1.18128900
H	-4.83166600	-0.73061900	2.18339200
C	-0.80332600	-4.00315700	-0.47851600
H	-0.51736600	-2.15084600	0.50783500
C	-3.05106000	-4.85899000	-0.62246900
C	-4.91128400	-3.62425600	0.43024500
H	-6.41155400	-2.46477200	1.41994700
C	-1.71054600	-4.99988100	-0.89991100
H	0.25072900	-4.12049000	-0.68394300
H	-3.76279400	-5.61899400	-0.93374700
H	-5.59891300	-4.38878900	0.07971700
H	-1.34562800	-5.87613100	-1.42751800

1c-Int$_{5syn}$⊂AuCav

C	4.93588200	-3.64748400	-0.37046600
C	4.20753200	-3.24649300	0.90827800
C	4.41737900	-2.81148400	-1.53619200
H	4.65148900	-4.67948900	-0.57789400
C	4.66552600	-2.20606300	1.72138000
C	3.04258900	-3.90990500	1.31580000
C	4.97080700	-1.57682900	-1.88645800
C	3.32971700	-3.26349500	-2.29348300
C	4.03066500	-1.82442900	2.90566800
H	5.57326900	-1.68970500	1.43293200
C	2.34456900	-3.53564200	2.45984800
O	2.64312600	-5.05203300	0.61503200
C	4.49233400	-0.80787500	-2.95104600
H	5.81399900	-1.20620500	-1.31547000
C	2.79768600	-2.52680300	-3.34564500
O	2.85016400	-4.54506800	-2.02946000
C	4.61562700	-0.75681900	3.82805500
C	2.84576200	-2.49708700	3.23891600
H	1.44985400	-4.06529200	2.76089100
C	1.49364800	-5.02945300	-0.11898600
C	5.11556800	0.53002800	-3.34446100
C	3.38870000	-1.30658900	-3.65593700
H	1.96394500	-2.90293700	-3.92409300
C	1.59912400	-4.72499200	-1.52252800
H	4.21926200	-0.96678600	4.82212500
C	4.11376500	0.63231200	3.45241400
O	2.21563100	-2.18382500	4.44284900
C	4.44443600	1.65257100	-2.56175700
H	4.86597900	0.69096700	-4.39371100
O	2.94831400	-0.58675300	-4.76351700
C	4.68564600	1.39085400	2.42525500
C	3.00788500	1.17889200	4.11490700
C	1.06270600	-1.44436800	4.39456500
C	-0.73889600	-5.39454000	-0.37795600
C	4.86722100	2.01703900	-1.27935500
C	3.34136500	2.33498500	-3.09145500
C	1.73578500	0.03993500	-4.72791100
C	-0.64162900	-5.04339200	-1.75943600
C	4.17021000	2.62697100	2.01849300
H	5.53987300	0.98490100	1.89492700
C	2.47821200	2.41306500	3.76432800
O	2.38330300	0.43397900	5.11313000
C	1.14869300	-0.06179000	4.76221500
C	4.22537200	2.98975400	-0.50682000
H	5.73596500	1.52062900	-0.86400900
C	2.64496800	3.28077300	-2.34693700
O	2.95437900	2.08740600	-4.40758000
C	1.74368400	1.47106600	-4.60072800
C	4.75565300	3.42991600	0.85888100
C	3.04548300	3.10282200	2.70286900
H	1.58592300	2.78107900	4.25254900
C	-1.18150500	-1.21314600	4.08261500
C	3.07745000	3.57490100	-1.06030600
H	1.77527400	3.77050000	-2.76682800
C	-0.52619700	0.08051600	-4.97752400
H	4.40766200	4.45694000	0.99727700
O	2.34532100	4.21731500	2.21983800
C	-1.08940100	0.17261400	4.42099000
O	2.30387900	4.47866200	-0.31948600
C	-0.50950000	1.50851500	-4.92090000
C	-1.80201400	-5.05761100	-2.56797500

C	-1.99210400	-5.77785300	0.15449100
C	-2.25126100	0.97769500	4.40140400
C	-2.44430000	-1.76222500	3.76141100
C	-1.71554800	2.22611800	-5.08359400
C	-1.75389200	-0.59363400	-5.17002900
C	6.29152900	3.46551800	0.90395800
H	6.68043600	4.08910300	0.09447700
H	6.74104500	2.47407800	0.80359700
C	6.14796600	-0.82183500	3.91611900
H	6.50974800	-0.09924700	4.65288300
H	6.64072500	-0.59518700	2.96636000
C	6.46396600	-3.60840600	-0.23323100
H	6.78338400	-4.25839300	0.58591800
H	6.85247800	-2.60705000	-0.02774600
C	6.64557200	0.53983700	-3.22346100
H	7.07530400	-0.25516700	-3.83885700
H	6.99686200	0.38720900	-2.19912500
H	7.04259500	1.49901200	-3.56720500
H	6.93159000	-3.95789600	-1.15784400
H	6.46700700	-1.82165200	4.22287900
H	6.62559100	3.88101200	1.85834700
C	-2.91645500	0.13125200	-5.32276700
C	-2.89616900	1.54514700	-5.28515500
H	-3.82683500	2.09201000	-5.38627500
H	-3.86193900	-0.38234000	-5.45828100
H	-1.68131800	3.30709800	-5.01719200
H	-1.74177600	-1.67778200	-5.20129300
C	-3.10212300	-5.80634400	-0.66197400
H	-2.04001600	-6.04673400	1.20419900
C	-3.01071600	-5.43358700	-2.02435300
H	-1.70058100	-4.77574600	-3.61044400
C	-3.56339300	-0.95778900	3.76616100
H	-2.49732400	-2.81494900	3.50826100
C	-3.46741800	0.41892100	4.07653500
H	-2.14822100	2.02857600	4.65061900
H	-4.36627100	1.02270500	4.03517900
H	-4.53445400	-1.35822900	3.50302200
H	-3.90599100	-5.43566000	-2.63570500
H	-4.06353400	-6.10940800	-0.26172300
P	1.40237400	3.81589900	0.89993400
N	0.09787900	4.82819300	0.91992800
C	-1.14702100	4.33798100	1.52092300
C	0.24077800	6.28151500	0.84877200
H	-1.17262100	3.24606400	1.49811200
H	-1.99396600	4.70374500	0.93336100
H	-1.25154700	4.67578600	2.55999700
H	1.14864500	6.53909200	0.30248800
H	0.28081400	6.73179300	1.84888800
H	-0.61782400	6.69631700	0.31066700
Au	1.05957800	1.51507800	0.73688000
N	0.66111000	2.18354700	-4.69900100
N	0.63633800	-0.63712500	-4.88196800
N	0.56502100	-4.70754500	-2.31152000
N	0.36189600	-5.35488900	0.43485400
N	-0.06476000	-2.00378200	4.06480900
N	0.11300500	0.72363500	4.76403400
C	-0.60605900	-0.90657400	0.59510700
C	0.64367100	-0.47416600	0.48863200
H	1.37650200	-1.20985800	0.16202700
C	-1.33521900	-2.17344700	0.23911100
C	-2.69589900	-1.61465100	-0.26036700
H	-1.47897800	-2.81122200	1.11637100
H	-0.81469200	-2.73936300	-0.53031400
C	-2.81648800	-0.36548500	0.61938100
O	-1.61815500	0.01802300	1.03010500
O	-3.81717700	0.28289200	0.89235700
H	-5.17679100	-0.20272800	0.62153200
C	-2.60973700	-1.17369700	-1.75991100
H	-1.56720200	-0.95282600	-1.98987200
H	-2.89369600	-2.04813200	-2.34740600
C	-3.87948200	-2.55907800	-0.05715500
O	-4.55610200	-3.03047500	-0.94389200
O	-4.08110900	-2.80618000	1.24579100
C	-5.25177500	-3.58620700	1.56034300
H	-6.13899500	-3.09876900	1.15400900
H	-5.15798100	-4.59109100	1.14603500
H	-5.29108300	-3.62139000	2.64817500
S	-7.13964600	0.08591700	1.52577900
O	-6.49065400	0.30503000	2.81564300
O	-8.41173700	-0.60745500	1.42217300
O	-6.14086500	-0.55615900	0.48304600
C	-7.34606200	1.76962300	0.76254700
F	-6.14617400	2.33779700	0.61657500
F	-8.10062200	2.52420700	1.56293800
F	-7.93479100	1.66695700	-0.43010900
C	-3.50363700	-0.00261500	-2.09718700
C	-3.01322900	1.34195300	-2.02183500
C	-4.83013100	-0.22523200	-2.40848600
C	-1.65405500	1.66158000	-1.75877800
C	-3.92773800	2.42550800	-2.24234900
C	-5.72666300	0.84526100	-2.62453500
H	-5.19547600	-1.24492100	-2.44954100
C	-1.22096900	2.96739900	-1.72633100
H	-0.92850500	0.87757600	-1.58608700
C	-3.45154800	3.76199400	-2.18410000
C	-5.28827500	2.14376500	-2.53122700
H	-6.76877800	0.63172600	-2.83766400
C	-2.12492400	4.03216600	-1.93573500
H	-0.17445400	3.17644500	-1.54701200
H	-4.15847100	4.57016500	-2.35247800
H	-5.97553300	2.97285900	-2.67485600
H	-1.76617300	5.05683500	-1.90634600

1c-TS2$_{5syn}$⊂AuCav

C	-5.62490200	2.01409200	1.70653400
C	-4.36936100	1.95075900	2.56955200
C	-5.37363000	1.37006300	0.34707700
H	-5.81421000	3.07093300	1.51703200
C	-4.05228900	0.78722800	3.27574400
C	-3.49961100	3.04106100	2.71060600
C	-5.67601600	0.03828500	0.05320700
C	-4.82385000	2.14857800	-0.67934800
C	-2.94531200	0.67459600	4.11720400
H	-4.71150300	-0.06711700	3.17559600
C	-2.37239900	2.97100200	3.53040200
O	-3.79728800	4.25536300	2.08019200
C	-5.48373300	-0.50879700	-1.21925000
H	-6.09822000	-0.58609400	0.83183400
C	-4.60689200	1.65398000	-1.96092000
O	-4.64224200	3.49373200	-0.38723100
C	-2.68113000	-0.59466300	4.91945600
C	-2.09500700	1.78554300	4.20715400
H	-1.73321600	3.83610200	3.65298900
C	-3.03345900	4.58618200	0.98662300
C	-5.78528000	-1.96883500	-1.55107300
C	-4.95631200	0.33000000	-2.20963400
H	-4.20850900	2.28409100	-2.74497300
C	-3.41791000	4.07960400	-0.30509700
H	-2.00440200	-0.31500800	5.72666900
C	-1.93859000	-1.60666800	4.05904900
O	-1.00246700	1.72138700	5.07516200
C	-4.51088000	-2.77893700	-1.33897300
H	-6.01151500	-2.01054900	-2.61662100
O	-4.90791300	-0.18765700	-3.50124600
C	-2.59685300	-2.52271100	3.23739100
C	-0.53809500	-1.62688400	4.05789500
C	0.26110800	1.58254800	4.56119000
C	-1.26568200	5.64534100	0.02152900
C	-4.14036000	-3.22765800	-0.06885600
C	-3.62988900	-3.05522000	-2.39247300
C	-3.72840700	-0.37783100	-4.15283900
C	-1.57818600	5.03766400	-1.23223600
C	-1.91100000	-3.43847100	2.43685200
H	-3.68035700	-2.53387100	3.23133600
C	0.19054300	-2.50348200	3.25652600
O	0.10536400	-0.78863100	4.96443400
C	0.84604000	0.26632800	4.51193200
C	-2.93920600	-3.88970500	0.19366700
H	-4.82253300	-3.05683400	0.75513700
C	-2.39886000	-3.67060100	-2.17008400

O	-4.00442100	-2.73290000	-3.69911100
C	-3.29071300	-1.73797600	-4.32032100
C	-2.62562500	-4.46125400	1.56944400
C	-0.51265200	-3.39580100	2.44831000
H	1.27368000	-2.49971600	3.27590700
C	2.22339800	2.46440800	3.79939700
C	-2.04281900	-4.03527600	-0.87407400
H	-1.72653500	-3.85772100	-2.99909000
C	-1.96956500	0.33933700	-5.40125700
H	-1.91768700	-5.27787900	1.40928000
O	0.19575200	-4.31462200	1.67237100
C	2.80739800	1.16159200	3.77351600
O	-0.80693200	-4.65218200	-0.69966800
C	-1.57395100	-1.01482500	-5.62608100
C	-0.74375900	5.27153400	-2.34808600
C	-0.14773400	6.50520500	0.11661700
C	4.15423500	0.99828800	3.37536800
C	3.00489500	3.57935400	3.41609800
C	-0.42217900	-1.28531000	-6.40016900
C	-1.18972200	1.38996800	-5.93507300
C	-3.85894200	-5.06839700	2.25482400
H	-4.30805700	-5.83177300	1.61385700
H	-4.63274000	-4.32950800	2.47817900
C	-3.95521100	-1.16477800	5.55990800
H	-3.70970300	-2.04249100	6.16420100
H	-4.70650500	-1.46917800	4.82584400
C	-6.85777400	1.44737300	2.42569900
H	-7.02872300	1.99202500	3.35808900
H	-6.75855100	0.38829400	2.67971600
C	-6.99439000	-2.53408900	-0.79489000
H	-7.88239800	-1.93131200	-1.00381700
H	-6.85575700	-2.54951700	0.28981900
H	-7.18871600	-3.56145400	-1.11464400
H	-7.74329700	1.55381300	1.79287700
H	-4.41614100	-0.41422500	6.20758500
H	-3.56657700	-5.53289000	3.20030800
C	-0.05844000	1.10071500	-6.66793700
C	0.32440900	-0.24140800	-6.90561800
H	1.21887400	-0.44630400	-7.48482800
H	0.54899700	1.90624300	-7.06792000
H	-0.14205200	-2.32141300	-6.55613800
H	-1.50433500	2.40871400	-5.73691200
C	0.64059500	6.73351300	-0.99226300
H	0.06643300	6.95885800	1.07842200
C	0.34543900	6.10852200	-2.22712200
H	-0.99416600	4.78151900	-3.28257500
C	4.32046700	3.39602400	3.03995900
H	2.54511600	4.56153200	3.44355900
C	4.89786300	2.10272000	3.01978100
H	4.56347100	-0.00443500	3.35117900
H	5.92503500	1.97056500	2.69764500
H	4.92194300	4.25247400	2.75088500
H	0.98700300	6.29163500	-3.08313200
H	1.50133000	7.39085600	-0.92015200
P	0.50809500	-3.99808100	0.06577500
N	1.61442500	-5.15883800	-0.34595400
C	3.04404500	-4.83602900	-0.25384500
C	1.30453400	-6.57822600	-0.15012800
H	3.21414500	-3.78730300	-0.49275300
H	3.58638000	-5.44631800	-0.98133500
H	3.44752000	-5.03089500	0.74703300
H	0.24099800	-6.75552300	-0.30665400
H	1.58495400	-6.91333200	0.85681300
H	1.86505300	-7.16371800	-0.88508100
Au	1.34268500	-1.84051200	-0.00204000
N	-2.25928400	-2.04936500	-5.04697900
N	-3.08478900	0.62849500	-4.66498400
N	-2.69785000	4.26477200	-1.37082700
N	-2.00887500	5.37033100	1.13844800
N	0.92797700	2.64157300	4.20639200
N	2.07080200	0.06523200	4.12813100
C	3.20490300	0.60080200	-0.36666200
C	2.57781100	-0.22474200	0.51188400
H	2.34942300	0.28386500	1.44377800
C	3.80228600	1.95768800	-0.14993600
C	3.74786600	2.57929700	-1.55606800
H	4.83860700	1.77623500	0.15112600
H	3.29524500	2.52485100	0.62342300
C	3.80079100	1.31988400	-2.43177700
O	3.46344100	0.21979600	-1.65340400
O	4.01931700	1.22234100	-3.60410100
H	3.49337200	-1.12141400	0.86679900
C	2.43342400	3.38929900	-1.80723100
H	2.24662000	3.42869000	-2.88344100
H	2.64909100	4.41291000	-1.49859100
C	4.96420900	3.45481800	-1.85457000
O	4.93216600	4.64512800	-2.07551700
O	6.08672900	2.72258000	-1.80488800
C	7.31363800	3.42981400	-2.05513300
H	8.10235800	2.68637800	-1.95325400
H	7.30573700	3.85337100	-3.06185500
H	7.44272000	4.23505000	-1.32837500
S	5.82565300	-1.55521200	1.03602400
O	6.79054800	-2.06253000	2.00527700
O	5.94012500	-0.16115700	0.57733700
O	4.37983000	-1.91840300	1.40748100
C	6.04726900	-2.57159500	-0.50084500
F	7.23016100	-2.31637000	-1.06249500
F	5.07584600	-2.28097000	-1.38334800
F	5.97600200	-3.87977100	-0.21273400
C	1.24883000	2.87402900	-1.02606400
C	0.47157500	1.75837600	-1.46796900
C	0.99909000	3.40771100	0.22176500
C	0.72346200	1.10058400	-2.69884700
C	-0.53278200	1.21553000	-0.59665200
C	-0.00392400	2.88985100	1.06833100
H	1.59627800	4.24368400	0.56946200
C	0.10248800	-0.08506400	-3.00649900
H	1.44780000	1.50479200	-3.39397000
C	-1.21737500	0.03431300	-0.98010400
C	-0.76098300	1.81605400	0.66746800
H	-0.15816800	3.34162200	2.03783600
C	-0.87671300	-0.62272900	-2.14155700
H	0.36691400	-0.60694000	-3.91678200
H	-1.98154100	-0.36984500	-0.32165300
H	-1.52154200	1.39693700	1.31918200
H	-1.35793100	-1.55475300	-2.39214700

1c-TS1$_6$⊂AuCav

C	3.72222600	2.55730800	3.87446100
C	3.84230100	3.05047100	2.43689700
C	2.87497600	1.29336200	3.97634700
H	3.17627600	3.33122800	4.41466800
C	4.74121900	2.45629800	1.54989400
C	3.08731000	4.13016700	1.95434000
C	3.41314100	0.00495800	4.00504800
C	1.48446200	1.41684700	4.10805200
C	4.94187100	2.90206000	0.24143900
H	5.30809100	1.59803800	1.89379700
C	3.27409300	4.62557000	0.66337100
O	2.15531200	4.74691400	2.78819300
C	2.62246100	-1.13025000	4.20793700
H	4.48568700	-0.11589100	3.90628600
C	0.65290900	0.31721800	4.30580600
O	0.99366700	2.70952300	4.19482400
C	5.92009000	2.18268900	-0.67893300
C	4.18561100	4.00054200	-0.18632800
H	2.70112500	5.48005000	0.32517900
C	0.83265800	4.42418400	2.53947700
C	3.19504100	-2.54417500	4.22678200
C	1.24397200	-0.94183400	4.37317100
H	-0.41380000	0.44252100	4.43835800
C	0.25861400	3.28389700	3.19463900
H	6.07383900	2.82878000	-1.54346200
C	5.26007400	0.90995100	-1.19175000
O	4.36837000	4.50060200	-1.48636900
C	3.12214400	-3.11010700	2.81323000
H	2.52789800	-3.14767200	4.84184300
O	0.50239900	-2.06534900	4.73662400
C	5.44348900	-0.33920200	-0.60008600

C	4.40957700	0.98219500	-2.30346100
C	3.33357700	4.26349500	-2.35859100
C	-1.15008200	4.79312200	1.48332000
C	4.08285800	-2.77829800	1.85493500
C	2.05945600	-3.92937900	2.40488000
C	-0.46142300	-2.57141200	3.91473400
C	-1.68712600	3.59540800	2.05423100
C	4.84475000	-1.50309600	-1.09181800
H	6.10851800	-0.41856500	0.25051400
C	3.76078700	-0.14011300	-2.81185600
O	4.36964400	2.19981800	-2.95776700
C	3.26107600	2.99206100	-3.03179900
C	4.02663000	-3.21913800	0.53183700
H	4.90665500	-2.14026000	2.15207200
C	1.94765500	-4.37333600	1.08583300
O	1.10753700	-4.32106900	3.33966000
C	-0.15518400	-3.78109200	3.20351200
C	5.11119300	-2.87126300	-0.48021300
C	3.99644700	-1.36645500	-2.19657800
H	3.13463700	-0.05922900	-3.69131900
C	1.38181000	4.87540500	-3.36255900
C	2.92018500	-3.99317500	0.16965500
H	1.10761000	-4.99143300	0.79341100
C	-2.56222000	-2.58986100	3.03537800
H	5.03581300	-3.60000000	-1.29093200
O	3.50896900	-2.52035800	-2.83132400
C	1.25561700	3.57079500	-3.93104300
O	2.82010800	-4.45799000	-1.15447600
C	-2.26146700	-3.80430700	2.33848100
C	-3.00522700	3.19632000	1.73419900
C	-1.95611100	5.57622600	0.62638400
C	0.09851700	3.24314300	-4.67182400
C	0.35856200	5.82775800	-3.57095500
C	-3.22683200	-4.36630000	1.47141700
C	-3.81826800	-1.96992900	2.84605100
C	6.52352900	-3.01443600	0.10595600
H	6.67814900	-4.04028600	0.44985700
H	6.70262300	-2.35432600	0.95811700
C	7.28656500	1.94785800	-0.02107400
H	7.96119900	1.43859500	-0.71545100
H	7.22590900	1.34485200	0.88929800
C	5.09932100	2.41249600	4.54076600
H	5.62612500	3.37025600	4.51899100
H	5.73957100	1.67704900	4.04474100
C	4.60133500	-2.63487400	4.83387500
H	4.59383200	-2.23924200	5.85298600
H	5.35170100	-2.07410700	4.26928200
H	4.92882900	-3.67762700	4.86857000
H	4.98308100	2.10254500	5.58304100
H	7.73236200	2.90685100	0.25485800
H	7.27271700	-2.78474100	-0.65636300
C	-4.73782100	-2.53613100	1.98839000
C	-4.44067400	-3.73420600	1.29438200
H	-5.16718700	-4.13041900	0.59381800
H	-5.68510300	-2.04106300	1.82136600
H	-2.97458200	-5.28422900	0.95004100
H	-4.02190100	-1.02757600	3.34053600
C	-3.24406400	5.17316300	0.34433800
H	-1.52662500	6.47992800	0.20678500
C	-3.77107800	3.98028400	0.89498300
H	-3.40683100	2.28460400	2.16371200
C	-0.76045200	5.48307700	-4.30043200
H	0.47587700	6.80919600	-3.12469300
C	-0.89359400	4.18448600	-4.84677400
H	0.01947800	2.24279000	-5.08340100
H	-1.78959000	3.92940700	-5.40315300
H	-1.55393900	6.20825700	-4.44948400
H	-4.79249000	3.68685400	0.67672200
H	-3.86761300	5.77407000	-0.31067700
P	2.29871700	-3.49291600	-2.36188400
N	2.37671000	-4.60411200	-3.62059800
C	2.23884300	-4.04470900	-4.97280000
C	1.59465500	-5.83358800	-3.43009800
H	2.89358100	-3.18180000	-5.08882600
H	1.20268000	-3.75149300	-5.20084700
H	2.54799000	-4.80991200	-5.68947900
H	1.77501100	-6.24207900	-2.43632300
H	1.92849000	-6.56667000	-4.16892700
H	0.51513200	-5.66802400	-3.56836400
Au	0.18718100	-2.60522000	-2.10260000
N	-1.02235200	-4.37911800	2.44157200
N	-1.62453200	-1.99743600	3.83415400
N	-0.94321400	2.86251200	2.93788200
N	0.14327200	5.16774200	1.72790000
N	2.44302600	5.18859700	-2.55526800
N	2.25247300	2.64604100	-3.77312000
C	-1.94059500	-2.20444700	-2.47843600
C	-2.14011000	-3.39821600	-2.20270300
H	-2.22941000	-4.42461900	-1.92308800
C	-2.67521600	-0.96589800	-2.84172400
C	-3.85913200	-0.68500100	-1.86960200
H	-3.06231000	-1.13227100	-3.85310100
H	-2.02800300	-0.09007200	-2.86426900
C	-4.76482400	-1.93234800	-1.65918300
O	-4.51155300	-3.03643200	-2.12799300
O	-5.81832300	-1.77131500	-0.89037800
H	-6.03812100	-0.80470400	-0.66199800
C	-3.35934300	-0.15286500	-0.46978100
H	-3.73083700	0.85983600	-0.34205200
H	-3.84563800	-0.73272600	0.31123100
C	-4.65812500	0.43050600	-2.57496500
O	-4.28053900	1.57887800	-2.65888500
O	-5.76906300	-0.03908000	-3.15093400
C	-6.66751100	0.96339200	-3.66838200
H	-7.49392200	0.40912600	-4.11052500
H	-7.01218600	1.58365500	-2.83916800
H	-6.16346300	1.57894100	-4.41604900
S	-6.45647900	1.21040400	0.98740400
O	-6.41776700	0.65387700	-0.41698000
O	-6.76372800	2.64496300	1.03050100
O	-5.35403700	0.72342800	1.84117000
C	-7.95632700	0.36711900	1.67901700
F	-8.10991500	0.66440900	2.97284000
F	-7.81960800	-0.97143000	1.56334200
F	-9.05859900	0.73115200	1.01586800
C	-1.87214400	-0.18393400	-0.20643500
C	-1.03713200	0.94843600	-0.45597900
C	-1.30811700	-1.31267000	0.35510000
C	-1.52191800	2.12022700	-1.09815700
C	0.33603500	0.91406100	-0.03061000
C	0.06198500	-1.37290600	0.70512800
H	-1.93603600	-2.17516200	0.52413900
C	-0.71398500	3.22058300	-1.24101100
H	-2.53421300	2.14688600	-1.47769500
C	1.12864400	2.08241200	-0.16337800
C	0.86764600	-0.26788500	0.54008500
H	0.46414300	-2.27707800	1.13614600
C	0.60202800	3.21642300	-0.72916100
H	-1.09628700	4.11241400	-1.72092200
H	2.14773200	2.06524700	0.20858200
H	1.90624700	-0.28447200	0.85913000
H	1.18346500	4.12257900	-0.77104600

1c-Int$_6$⊂AuCav

C	3.45207500	2.17377000	4.21969700
C	3.70246300	2.75061400	2.82987000
C	2.56629900	0.93276000	4.18407300
H	2.88785200	2.93192800	4.76318700
C	4.66821200	2.18440800	1.99755000
C	3.03018600	3.88479300	2.34736500
C	3.05589800	-0.37576000	4.16271700
C	1.17669100	1.10199700	4.23457000
C	5.02698200	2.71159500	0.75563200
H	5.16680300	1.28246400	2.33450100
C	3.37624000	4.46450100	1.12524300
O	2.03000200	4.48009000	3.12033000
C	2.21106600	-1.48858600	4.23195600
H	4.12762200	-0.53401400	4.12549400
C	0.29300300	0.02969500	4.30211300
O	0.73150800	2.40934000	4.36346800

C	6.06716100	2.00144700	-0.10210500
C	4.36159100	3.87047700	0.33481300
H	2.86037900	5.35374700	0.78474800
C	0.72253800	4.19557000	2.77304000
C	2.71793700	-2.92762800	4.19032200
C	0.83366400	-1.25242400	4.31493400
H	-0.77510000	0.18885600	4.36846800
C	0.07731500	3.04854600	3.35064200
H	6.34070100	2.68817800	-0.90317400
C	5.40960900	0.79367700	-0.75805300
O	4.70321300	4.45487500	-0.89621000
C	2.73963200	-3.39376200	2.73874000
H	1.97752900	-3.53829600	4.70650500
O	0.02761000	-2.36799000	4.53507400
C	5.47626000	-0.49871100	-0.23876300
C	4.67914800	0.97802600	-1.93902400
C	3.77845400	4.32398600	-1.90424700
C	-1.19509700	4.66838100	1.63599100
C	3.79564100	-3.04043000	1.89565700
C	1.68200000	-4.12228800	2.17331500
C	-0.90687300	-2.76764800	3.62930700
C	-1.79231800	3.46458700	2.12293700
C	4.87647100	-1.59521100	-0.86636100
H	6.05083700	-0.66836100	0.66327900
C	4.03067100	-0.07210100	-2.58266300
O	4.77159700	2.23998600	-2.50409400
C	3.72701600	3.09746500	-2.66331400
C	3.84375300	-3.38457900	0.54524200
H	4.61380700	-2.46400500	2.31073400
C	1.67643600	-4.47041300	0.82095000
O	0.62527600	-4.52836900	2.98690400
C	-0.60114700	-3.92514000	2.82761700
C	5.02956300	-3.01391900	-0.33612000
C	4.14425900	-1.34944600	-2.03567400
H	3.49656700	0.09749100	-3.50888200
C	2.02051500	5.11562600	-3.12012400
C	2.74878100	-4.08284900	0.02366800
H	0.83894400	-5.01657100	0.40436000
C	-2.99375500	-2.68780000	2.72197900
H	4.99810900	-3.67744200	-1.20348700
O	3.67337900	-2.42956100	-2.78578900
C	1.89544000	3.85386600	-3.77670400
O	2.76982000	-4.45279800	-1.32560000
C	-2.70163500	-3.85209800	1.94414400
C	-3.09633700	3.10961100	1.70791700
C	-1.93933800	5.50866800	0.77690000
C	0.82871600	3.63941000	-4.67721200
C	1.08577800	6.13899500	-3.39432600
C	-3.68720800	-4.36969400	1.07457000
C	-4.25492000	-2.06217800	2.59600300
C	6.37518400	-3.26399600	0.36065700
H	6.45691200	-4.31800000	0.63858700
H	6.50239400	-2.67563500	1.27293200
C	7.34424400	1.66191300	0.67825000
H	8.06715500	1.16491600	0.02486700
H	7.16198200	1.00565800	1.53390200
C	4.76734500	1.94573800	4.98168300
H	5.32624700	2.88285200	5.05096500
H	5.41941000	1.21152000	4.49980100
C	4.06328500	-3.12605100	4.90144400
H	3.98440000	-2.80804300	5.94459600
H	4.87920000	-2.55789400	4.44580200
H	4.34734800	-4.18180300	4.88032800
H	4.55767800	1.58915500	5.99408000
H	7.79763900	2.57900600	1.06327700
H	7.19994100	-3.01549000	-0.31271700
C	-5.19544100	-2.58292200	1.73156900
C	-4.91193900	-3.74271500	0.97314000
H	-5.65725200	-4.11912800	0.28180200
H	-6.15034300	-2.08589300	1.61456800
H	-3.44586000	-5.25344100	0.49391400
H	-4.44399200	-1.16089500	3.16765000
C	-3.21597600	5.14689200	0.39965800
H	-1.46993700	6.42037400	0.42272400
C	-3.79530900	3.93842500	0.85543400
H	-3.52701000	2.18491500	2.07582000
C	0.05164300	5.90452000	-4.27698500
H	1.19823600	7.08561900	-2.87709100
C	-0.07924000	4.64937600	-4.91682400
H	0.75083700	2.66972100	-5.15674800
H	-0.90438000	4.48338300	-5.60218400
H	-0.67397700	6.68582600	-4.47988800
H	-4.79212700	3.66142700	0.53125200
H	-3.78529700	5.78928700	-0.26510600
P	2.36771700	-3.37826800	-2.50724800
N	2.57561400	-4.41318000	-3.82586800
C	2.58328600	-3.76025100	-5.14182100
C	1.74373000	-5.62459700	-3.80468200
H	3.25600800	-2.90322600	-5.12895300
H	1.58018600	-3.43180400	-5.45393400
H	2.95711700	-4.47884300	-5.87665500
H	1.80649900	-6.10295900	-2.82770900
H	2.13540700	-6.31940900	-4.55300900
H	0.68938600	-5.41422800	-4.03921500
Au	0.22723100	-2.53086800	-2.34506200
N	-1.46886500	-4.44960900	2.01404000
N	-2.05850100	-2.16810400	3.57343300
N	-1.12129600	2.67578200	3.01410700
N	0.09416400	4.99373000	1.96254700
N	2.98001300	5.31562100	-2.16190200
N	2.80950300	2.86084300	-3.55118700
C	-1.78718800	-2.13806100	-2.40957400
C	-2.64502500	-3.07356300	-2.01118500
H	-2.44066100	-4.09480200	-1.72597300
C	-2.37525200	-0.78571300	-2.73936400
C	-3.52386700	-0.44476900	-1.75607800
H	-2.76203300	-0.79523600	-3.76523500
H	-1.62816500	0.00623700	-2.68315500
C	-4.50686200	-1.61304000	-1.68475500
O	-4.04475900	-2.83950600	-1.87214700
O	-5.70392000	-1.50011400	-1.41867000
H	-6.49113200	-0.29314400	-1.11484400
C	-3.01036200	-0.20018500	-0.28432200
H	-3.49845500	0.70779100	0.06825800
H	-3.39056100	-0.99998200	0.35039100
C	-4.31447100	0.76035100	-2.28378700
O	-4.43439900	1.83464300	-1.73537700
O	-4.87766900	0.47502500	-3.46932700
C	-5.72256400	1.51044400	-4.01145000
H	-6.10826200	1.10852900	-4.94725600
H	-6.53264900	1.72709400	-3.31273400
H	-5.14238000	2.41868100	-4.18765400
S	-6.86867900	1.22272100	0.43211700
O	-7.10536900	0.52884700	-0.96766100
O	-7.08650500	2.65424900	0.31025900
O	-5.68859100	0.68103000	1.10311800
C	-8.32644400	0.53547300	1.35622500
F	-8.28065900	0.96399800	2.61736600
F	-8.27005200	-0.80437900	1.34434200
F	-9.46447500	0.92936200	0.78999300
C	-1.51650100	-0.11381200	-0.06196700
C	-0.77301300	1.06776600	-0.36270200
C	-0.85411000	-1.18400500	0.50754700
C	-1.35354600	2.20507100	-0.98723100
C	0.61774000	1.11986600	-0.01539800
C	0.52119300	-1.13762400	0.82323600
H	-1.40692600	-2.09160600	0.70263500
C	-0.61808300	3.34640100	-1.20031400
H	-2.39857600	2.19248600	-1.25681900
C	1.33366700	2.32173200	-0.22024100
C	1.24860700	-0.00118100	0.57538500
H	0.99750800	-1.99578700	1.26964000
C	0.72302200	3.42230700	-0.76699400
H	-1.07951200	4.21016300	-1.66436500
H	2.36703800	2.35297300	0.09594600
H	2.30017100	0.05976500	0.84062600
H	1.25174800	4.35905500	-0.83981000

1c-TS2$_6$⊂AuCav

C	5.76397600	-2.07697500	-0.01298700

C	5.20349000	-1.34272200	-1.22646700
C	4.70561800	-2.17331800	1.08205100
H	6.56579000	-1.45394800	0.38389500
C	4.35326400	-2.00367900	-2.11410100
C	5.49529900	0.00031400	-1.50448400
C	3.92097900	-3.30579800	1.30496200
C	4.52619700	-1.08227500	1.94380200
C	3.79621800	-1.40320400	-3.24413600
H	4.10060000	-3.03780700	-1.90694300
C	4.96572000	0.64199100	-2.62561800
O	6.34750700	0.71377700	-0.65423500
C	3.00946800	-3.38700600	2.36238600
H	4.05671500	-4.16976400	0.66531700
C	3.60847100	-1.09801700	2.99116700
O	5.45682700	-0.06397700	1.79796400
C	2.90585900	-2.20865200	-4.18043300
C	4.09380300	-0.05155100	-3.46596100
H	5.19148500	1.68422900	-2.81458800
C	5.75475400	1.64471100	0.16855200
C	2.19497100	-4.64269000	2.65553000
C	2.86777100	-2.26307200	3.18628500
H	3.51241200	-0.25498500	3.66319200
C	5.18685600	1.21261400	1.42215600
H	2.76397300	-1.60009300	-5.07378900
C	1.52332400	-2.41052900	-3.57470100
O	3.49398000	0.62165600	-4.53990600
C	0.84626800	-4.55439600	1.95376600
H	1.99102300	-4.63874600	3.72612600
O	2.08006500	-2.42629900	4.32555200
C	1.15672400	-3.54663600	-2.84979200
C	0.54590900	-1.42790200	-3.77769000
C	2.44599600	1.44633800	-4.18077400
C	5.13912800	3.78232600	0.65274200
C	0.72765900	-4.82883600	0.58955800
C	-0.30971400	-4.14273800	2.62969700
C	0.90189500	-1.77546300	4.52740300
C	4.50526800	3.33716300	1.85206100
C	-0.14470400	-3.75236800	-2.38064600
H	1.89716500	-4.31910700	-2.68212700
C	-0.76454700	-1.58724000	-3.33566600
O	0.91311500	-0.35377300	-4.57548900
C	1.11876500	0.89815500	-4.06557400
C	-0.47460000	-4.72265900	-0.11081000
H	1.61832900	-5.11901000	0.04490300
C	-1.53711700	-4.02911000	1.97614500
O	-0.22849200	-3.85059900	3.98943400
C	-0.31591100	-2.53178100	4.36753800
C	-0.55145400	-5.00167700	-1.60835300
C	-1.09058500	-2.75665200	-2.65505500
H	-1.51577700	-0.84313500	-3.55211900
C	1.64155500	3.46753400	-3.49394800
C	-1.59859700	-4.30284100	0.61291900
H	-2.41387200	-3.69791600	2.51893200
C	-0.31092500	0.00570100	5.26858200
H	-1.60098300	-5.19204200	-1.84418200
O	-2.43896800	-3.01648100	-2.38058000
C	0.36097200	2.88410500	-3.24595100
O	-2.82673000	-4.18244700	-0.04407900
C	-1.51974500	-0.73446700	5.08695500
C	3.82139600	4.26725900	2.66645300
C	5.08088200	5.15024700	0.30196500
C	-0.66204700	3.66671300	-2.66178700
C	1.85740300	4.83052000	-3.18868800
C	-2.76157700	-0.12214200	5.37420600
C	-0.37343300	1.32462000	5.77379500
C	0.23041500	-6.25839800	-2.01515300
H	-0.15766900	-7.12727900	-1.47737200
H	1.29904300	-6.18895500	-1.79613600
C	3.57519200	-3.52219200	-4.61323700
H	2.92676900	-4.06885000	-5.30388200
H	3.80097100	-4.18305000	-3.77147500
C	6.37657700	-3.43298400	-0.39200300
H	7.17817500	-3.28783200	-1.12099200
H	5.65213300	-4.12096600	-0.83736900
C	2.93988700	-5.94525700	2.32992300

H	3.88985200	-5.97946700	2.87017500
H	3.16085600	-6.05935900	1.26493400
H	2.33671300	-6.80566500	2.63227600
H	6.79579900	-3.91900600	0.49353300
H	4.51962800	-3.30700800	-5.11983900
H	0.12292500	-6.43616400	-3.08848100
C	-1.59673500	1.89592500	6.05657300
C	-2.79575500	1.17471800	5.84630000
H	-3.74730200	1.65718000	6.04032300
H	-1.64881500	2.91749200	6.41732900
H	-3.66708000	-0.69695500	5.21227800
H	0.55785800	1.86420300	5.90653200
C	4.40742700	6.03915300	1.11453000
H	5.56628100	5.46298400	-0.61627500
C	3.77336000	5.59534900	2.29978300
H	3.33513100	3.89989300	3.56316400
C	0.84269100	5.57438900	-2.62373300
H	2.83808000	5.24960000	-3.38665100
C	-0.41547800	4.98721900	-2.35155300
H	-1.62839800	3.21997900	-2.47358400
H	-1.20334600	5.58235400	-1.90174800
H	1.01060400	6.61860500	-2.37830100
H	3.24007900	6.30824500	2.92044200
H	4.35840400	7.08923100	0.84369500
P	-3.18794400	-2.85246200	-0.94163400
N	-4.73853100	-3.29806700	-1.40839200
C	-5.33073100	-2.50348400	-2.50152200
C	-5.67930300	-3.51314200	-0.29970200
H	-4.62065300	-2.41438800	-3.32131400
H	-5.63301500	-1.50099200	-2.17291200
H	-6.21117100	-3.04140000	-2.86304000
H	-5.22393100	-4.14438300	0.46335500
H	-6.55692800	-4.03106600	-0.69550300
H	-6.01070200	-2.56741000	0.15526100
Au	-3.09302200	-0.78738300	0.03156700
N	-1.48423900	-2.02456100	4.62592700
N	0.90114000	-0.55063400	4.96370400
N	4.56718900	2.02313400	2.22632400
N	5.75363500	2.89278100	-0.18888300
N	2.68328100	2.70139700	-3.94547000
N	0.12266000	1.58205700	-3.59129300
C	-3.68598300	1.17738500	0.72436300
C	-4.41254300	1.06127600	1.85972100
H	-5.13019600	0.27213900	2.05584200
C	-2.80469400	2.40064200	0.58843800
C	-2.33297700	2.92512600	1.95864200
H	-3.36635500	3.17320500	0.05867200
H	-1.92701800	2.18885700	-0.02532000
C	-3.50081900	2.98834800	2.94964900
O	-4.40790000	1.94553800	2.90644400
O	-3.64962800	3.82281100	3.80151000
H	-4.53731400	1.02749600	-0.38045300
C	-1.27187900	1.98598200	2.64268200
H	-0.63674200	2.62907100	3.25338800
H	-1.80385900	1.33346000	3.33189800
C	-1.82527700	4.37644100	1.89533700
O	-0.95812400	4.84352200	2.60246200
O	-2.49358500	5.09081100	0.97866500
C	-2.12764100	6.47819600	0.89192600
H	-2.78045600	6.90350000	0.13085500
H	-2.27872100	6.97032100	1.85483500
H	-1.07822800	6.57229600	0.60184700
S	-5.02300400	2.01829500	-2.50259000
O	-4.46902400	3.28229600	-2.00637500
O	-6.08644900	2.01653300	-3.49873100
O	-5.32063200	1.01879800	-1.36140000
C	-3.56532000	1.20012700	-3.31556700
F	-3.88389900	-0.00442900	-3.81619400
F	-2.58798800	1.01784600	-2.40194500
F	-3.09078900	1.96278700	-4.29778100
C	-0.44977000	1.09298700	1.74330600
C	0.59314300	1.59315700	0.90502500
C	-0.69569400	-0.26963000	1.75205100
C	0.95548600	2.96558000	0.85594300
C	1.31908300	0.67458300	0.07771200

C	0.04421800	-1.17310200	0.95116500
H	-1.46266300	-0.66189600	2.40928300
C	1.94270700	3.40934900	0.00913300
H	0.47852400	3.66990700	1.51961500
C	2.34161800	1.16292500	-0.76507500
C	1.02833300	-0.70958400	0.11822000
H	-0.17349700	-2.22604700	0.99693500
C	2.64440100	2.50093300	-0.81167900
H	2.20341700	4.46056900	-0.01388300
H	2.87597100	0.44749300	-1.37040000
H	1.60034000	-1.39066800	-0.50463900
H	3.42899900	2.85905700	-1.46290700

(TfO•3c)⊂AuCav

C	4.40242100	-3.57831200	1.98277600
C	4.74639200	-2.63231500	0.83938200
C	2.92931000	-3.49133200	2.36341800
H	4.96251200	-3.22556000	2.84928200
C	4.44830200	-2.97609200	-0.48076600
C	5.39847300	-1.40876700	1.04806900
C	1.94840500	-4.35210400	1.86442600
C	2.52789000	-2.53148600	3.30071000
C	4.80410800	-2.19720300	-1.58395900
H	3.90883200	-3.89952800	-0.65844000
C	5.79403100	-0.60830000	-0.02231700
O	5.70435600	-0.99076600	2.35063800
C	0.62288100	-4.31760000	2.31004000
H	2.23100000	-5.09560000	1.12853800
C	1.22768900	-2.46188400	3.78841200
O	3.53964600	-1.73956300	3.83622400
C	4.42426700	-2.63652500	-2.99414000
C	5.48977600	-1.00334700	-1.32472700
H	6.33730300	0.31101800	0.15932700
C	4.87524900	-0.01874400	2.86336000
C	-0.46210000	-5.23439500	1.75070200
C	0.29849800	-3.37793800	3.29896000
H	0.94950000	-1.74349200	4.54793900
C	3.66340500	-0.42011800	3.53220300
H	5.00869200	-2.02505500	-3.68215700
C	2.95775700	-2.29769400	-3.23412200
O	5.92316800	-0.20110700	-2.39067100
C	-1.14814300	-4.53024400	0.58290700
H	-1.21272400	-5.34442400	2.53340800
O	-0.96937200	-3.46174100	3.86249000
C	1.91569400	-3.21043300	-3.07059400
C	2.61672700	-0.99427400	-3.61656100
C	5.25283600	0.98471000	-2.55519600
C	4.32173200	2.16289900	3.21295900
C	-0.60022600	-4.54968500	-0.70226600
C	-2.32648900	-3.79150400	0.76954500
C	-1.86295700	-2.44068900	3.69080000
C	3.05806100	1.76253300	3.74420100
C	0.57586800	-2.87110200	-3.28696900
H	2.15461600	-4.23063500	-2.79620300
C	1.29980900	-0.59558900	-3.80954400
O	3.68676400	-0.16308900	-3.92117100
C	3.99433200	0.98052000	-3.26076700
C	-1.16522000	-3.86399100	-1.77999300
H	0.31331700	-5.10927800	-0.86781200
C	-2.91230000	-3.06794000	-0.26881900
O	-2.93687200	-3.81536000	2.02443900
C	-2.92656700	-2.64508000	2.74684300
C	-0.54706500	-3.89486600	-3.17222200
C	0.29103800	-1.54473400	-3.64284800
H	1.06614100	0.41502200	-4.11948700
C	5.04274200	3.22900500	-2.23715400
C	-2.31323600	-3.10650600	-1.52557800
H	-3.80139700	-2.47819300	-0.09421000
C	-2.72444800	-0.40221000	4.22411600
H	-1.32721100	-3.57540700	-3.86702000
O	-1.00791300	-1.16724700	-3.96321900
C	3.74724200	3.20045300	-2.83752300
O	-2.86529000	-2.33905700	-2.56301600
C	-3.80315200	-0.61700300	3.30792500
C	2.12151300	2.74621200	4.13407300
C	4.63836400	3.53799100	3.13492800
C	2.99284100	4.39266700	-2.92476600
C	5.56261000	4.45181300	-1.75510300
C	-4.76568700	0.39673100	3.10659000
C	-2.63687800	0.82466700	4.92073100
C	-0.12532800	-5.31076500	-3.59152900
H	-0.99328100	-5.97509800	-3.57560800
H	0.62932100	-5.74550000	-2.93063800
C	4.77227700	-4.10591300	-3.26722700
H	4.50313200	-4.37285200	-4.29309400
H	4.25704400	-4.80024600	-2.59764000
C	4.86283600	-5.01517000	1.69126800
H	5.94058300	-5.03106400	1.50830800
H	4.37614900	-5.45029100	0.81397200
C	0.04629600	-6.63646500	1.38998900
H	0.50038400	-7.10913700	2.26535400
H	0.79492700	-6.63106100	0.59256400
H	-0.78625200	-7.25915400	1.05171000
H	4.64479600	-5.66069000	2.54659100
H	5.84609800	-4.26437800	-3.13748300
H	0.28690300	-5.29866000	-4.60424700
C	-3.58315100	1.80341200	4.69652200
C	-4.64420500	1.59121700	3.78404400
H	-5.35116900	2.38748700	3.58333700
H	-3.50982600	2.75403400	5.21622500
H	-5.54983400	0.24318200	2.37805500
H	-1.81005400	0.96418000	5.60904100
C	3.71822900	4.47871800	3.54765900
H	5.60706900	3.81657700	2.73435200
C	2.45159500	4.08184900	4.03706600
H	1.15955500	2.41505200	4.50992700
C	4.81718200	5.60696500	-1.87105900
H	6.54816600	4.44422800	-1.30233300
C	3.53030600	5.57528900	-2.45889600
H	2.00343600	4.34292700	-3.36532100
H	2.96954700	6.49987900	-2.55839000
H	5.21851900	6.54941500	-1.51200100
H	1.73606200	4.83838400	4.34429900
H	3.96074400	5.53517000	3.48765600
P	-2.19685900	-0.86186000	-2.83811900
N	-3.35905700	-0.09590600	-3.68169100
C	-4.50732200	-0.72825100	-4.32795100
C	-3.20429300	1.34421900	-3.90727200
H	-4.55754500	-1.78166600	-4.06087100
H	-4.42890300	-0.63353900	-5.41803100
H	-5.41304700	-0.23103500	-3.97189900
H	-2.41821300	1.75017200	-3.26743000
H	-4.13765800	1.83677000	-3.63221900
H	-2.95072100	1.54142500	-4.95638600
Au	-1.46296400	-0.05424800	-0.83440200
N	-3.87241900	-1.77215000	2.57559000
N	-1.76255100	-1.35873200	4.40546100
N	2.76607000	0.43763300	3.91778200
N	5.20709000	1.23120400	2.73955800
N	5.77093000	2.07809400	-2.08272400
N	3.25112300	2.04087800	-3.36960000
C	-0.52238100	6.60218400	0.02753900
C	0.21407400	5.90504600	-0.83196300
H	1.20064900	6.17040600	-1.18517700
C	-1.87037900	6.09101100	0.43631000
C	-1.89332900	4.53913300	0.39673600
H	-2.64493400	6.48375100	-0.23341500
H	-2.13205600	6.41526200	1.44861100
C	-1.25365700	4.00663600	-0.88794400
O	-0.20003200	4.71480600	-1.40527000
O	-1.56307600	2.97667800	-1.43197400
H	-0.14027400	7.53903900	0.41642200
C	-1.12795200	3.97063400	1.63659300
H	-0.31234800	4.65047900	1.88260300
H	-1.84833100	4.00083900	2.45650400
C	-3.37505600	4.12132000	0.46831500
O	-3.90027000	3.70438700	1.47779800
O	-3.99637000	4.41171800	-0.67413600
C	-5.43129200	4.19592600	-0.73777400
H	-5.62534700	3.69367100	-1.68265000

H	-5.75572400	3.55863200	0.08204000
H	-5.91308500	5.17634900	-0.69891300
C	-0.62448700	2.55791800	1.46785200
C	0.72047400	2.29823900	1.02904300
C	-1.49441800	1.51181200	1.66663100
C	1.66096300	3.33322500	0.79758300
C	1.14572800	0.94707900	0.80765800
C	-1.08278300	0.16492400	1.43632400
H	-2.51951000	1.69805700	1.95861400
C	2.94192300	3.05027500	0.38794000
H	1.37915100	4.36528600	0.95226400
C	2.47334600	0.68150700	0.39613100
C	0.22307800	-0.11761300	1.02015800
H	-1.74573900	-0.64087500	1.71387100
C	3.35585700	1.71637800	0.19804700
H	3.64742000	3.85620000	0.22593500
H	2.78223300	-0.34905900	0.24750400
H	0.56694900	-1.14478300	0.94703500
H	4.37844700	1.51380300	-0.07642500
S	-5.39767600	0.63545800	-0.52719800
O	-4.03055200	0.24549600	-0.07523100
O	-6.21261300	1.31491900	0.50135500
O	-5.47197400	1.21611100	-1.88502300
C	-6.24275700	-1.00646600	-0.71070400
F	-5.58964000	-1.78281500	-1.60451300
F	-7.50173700	-0.85560200	-1.13877100
F	-6.26220400	-1.66355500	0.45962400

(TfO•1c)⊂AuCav-1

C	-2.89077100	-4.52565800	2.55272900
C	-1.51916500	-3.96726300	2.92141400
C	-3.46564700	-3.83911800	1.31581400
H	-3.55618800	-4.27238000	3.37869500
C	-0.29619600	-4.51479300	2.52076600
C	-1.47521600	-2.83158600	3.73651600
C	-3.04830800	-4.25000900	0.04843000
C	-4.44592600	-2.83368900	1.36364200
C	0.93278100	-3.97884700	2.93218400
H	-0.29651100	-5.39024100	1.88142200
C	-0.29117700	-2.27476800	4.19426600
O	-2.70473200	-2.33352400	4.14252900
C	-3.58751700	-3.77387600	-1.14690000
H	-2.26475100	-4.99646700	-0.00950000
C	-5.01481400	-2.32764700	0.19345100
O	-4.93697900	-2.35638000	2.59143200
C	2.28525300	-4.52065200	2.47475000
C	0.89839400	-2.87109100	3.78912000
H	-0.28681000	-1.40988900	4.84215200
C	-3.14946700	-1.13742900	3.67929500
C	-3.06769000	-4.29962200	-2.48047700
C	-4.59034300	-2.79958300	-1.05007200
H	-5.80167500	-1.58494500	0.25247300
C	-4.42492100	-1.15011900	3.01257000
H	3.00615000	-4.24333800	3.24418200
C	2.72159700	-3.80721800	1.19607700
O	2.11050700	-2.39838800	4.28128300
C	-1.75976100	-3.58869400	-2.80643400
H	-3.79012500	-3.99987400	-3.24001100
O	-5.23921400	-2.34410800	-2.20222700
C	2.25801700	-4.22916000	-0.05416700
C	3.57541800	-2.69143300	1.22236700
C	2.49845300	-1.14736100	3.88852900
C	-3.11980400	1.13159600	3.60272700
C	-0.49745500	-4.11538800	-2.53487600
C	-1.81733700	-2.31874400	-3.39495800
C	-4.96705200	-1.06562400	-2.62942800
C	-4.48090100	1.12288200	3.17091700
C	2.60815500	-3.59812700	-1.24952500
H	1.58503300	-5.07772500	-0.09573200
C	3.95299100	-2.02845100	0.05661100
O	4.07758800	-2.24208500	2.44591100
C	3.56040900	-1.06118400	2.92578100
C	0.68542600	-3.43411300	-2.84376200
H	-0.42804600	-5.10385900	-2.09739600
C	-0.67373500	-1.58667400	-3.69283300
O	-3.07804600	-1.91230100	-3.79262600
C	-3.74500800	-0.81341300	-3.35020900
C	2.05988400	-4.04757300	-2.59828700
C	3.45635300	-2.49040200	-1.15745400
H	4.58540600	-1.14937900	0.09115300
C	2.39342700	1.12002900	3.98863600
C	0.56870100	-2.15809800	-3.41464200
H	-0.74654600	-0.62004700	-4.17515400
C	-5.52886500	1.12689700	-2.86725700
H	2.72397400	-3.63238700	-3.35999300
O	3.79059300	-1.78750200	-2.32294000
C	3.47518500	1.21079900	3.05669200
O	1.70170400	-1.47276100	-3.84135700
C	-4.24800300	1.40083300	-3.43971100
C	-5.16817600	2.34878600	3.04173400
C	-2.44629800	2.36149800	3.77708700
C	3.95081300	2.48346100	2.66940400
C	1.80663800	2.29907300	4.50193800
C	-3.91016400	2.72668900	-3.78779200
C	-6.46229400	2.17629800	-2.72268100
C	2.08563200	-5.57382400	-2.76348700
H	1.70944200	-5.85377200	-3.75119000
H	1.47985900	-6.09529000	-2.01760300
C	2.31099500	-6.04960000	2.34106300
H	3.31300500	-6.38429000	2.05964500
H	1.61540100	-6.42673600	1.58604200
C	-2.89373600	-6.05599600	2.41551800
H	-2.54155600	-6.51485800	3.34365400
H	-2.25300100	-6.41485800	1.60534200
C	-2.96493300	-5.83020200	-2.51552700
H	-3.94662000	-6.27362800	-2.32942400
H	-2.27788400	-6.23266300	-1.76586300
H	-2.61489100	-6.16134300	-3.49734500
H	-3.90736900	-6.41203500	2.21356900
H	2.04453800	-6.51335400	3.29483200
H	3.11091400	-5.93964200	-2.66387500
C	-6.11642100	3.45846900	-3.09371900
C	-4.83163200	3.73753100	-3.61325400
H	-4.57025700	4.75811300	-3.87119200
H	-6.82619600	4.26877200	-2.96613300
H	-2.92329900	2.91316700	-4.19692600
H	-7.43166700	1.94193500	-2.29639600
C	-3.12971000	3.54528900	3.58806100
H	-1.40325400	2.33929200	4.07533200
C	-4.50168000	3.53823200	3.24884900
H	-6.20945400	2.32300800	2.74080500
C	2.28767500	3.52995300	4.10308300
H	0.99274400	2.19667100	5.21218600
C	3.36191700	3.61898400	3.18725700
H	4.76105100	2.54399200	1.95691500
H	3.71634900	4.59353000	2.87060100
H	1.83874800	4.43886100	4.49152700
H	-5.02317600	4.47906500	3.11249000
H	-2.61596200	4.49165800	3.71669100
P	2.72756900	-0.64338900	-2.82713400
N	3.64195000	0.25439500	-3.82865200
C	4.92438400	-0.16656400	-4.39639900
C	3.13611600	1.54795100	-4.29414600
H	5.24118500	-1.11006800	-3.95590000
H	5.66648300	0.59947400	-4.16140400
H	4.83225700	-0.28920700	-5.48246300
H	2.91180700	1.50254800	-5.36681300
H	3.89058700	2.31025600	-4.08950200
H	2.23342700	1.82453600	-3.74872100
Au	1.64885800	0.24182400	-0.97232800
N	-3.37400900	0.38056900	-3.70305100
N	-5.84636600	-0.13194900	-2.42895300
N	-5.09364100	-0.05364900	2.81624000
N	-2.48602300	-0.04310900	3.90051900
N	1.92613100	-0.09804000	4.39737700
N	4.04116100	0.07801400	2.53193000
C	0.59215100	1.07673200	0.92251700
C	0.67788700	-0.14984600	1.01337200
H	0.63547700	-1.18215700	1.29593500
C	0.30625200	2.50106200	1.14937700

C	0.36429400	3.44845300	-0.07777400
H	1.00682600	2.86455600	1.90414900
H	-0.69630300	2.54897600	1.58348100
C	1.81160400	3.46338800	-0.62120700
O	2.12136500	2.91768000	-1.66979700
O	2.64677600	4.05735400	0.19747700
H	3.61169700	3.85151300	-0.07661800
C	-0.59164900	3.03894800	-1.23824900
H	-0.10211400	2.22138800	-1.77058800
H	-0.62382500	3.87550800	-1.93988300
C	0.04256200	4.88309400	0.34485300
O	0.16410400	5.82819800	-0.40443900
O	-0.42642500	4.97894000	1.60081000
C	-0.83459900	6.29906400	2.00181300
H	-1.00971800	6.23954900	3.07559700
H	-0.05366100	7.02584400	1.77362700
H	-1.75373600	6.57123600	1.47698400
S	5.27378700	2.07256300	-0.98562500
O	5.62273300	2.15851300	-2.41061000
O	4.27070500	1.04997300	-0.59723000
O	5.06378000	3.39628800	-0.30643000
C	6.81733300	1.44100800	-0.17059600
F	7.03005700	0.17147400	-0.55675200
F	7.87849000	2.17736400	-0.51313800
F	6.69675400	1.45736800	1.16541600
C	-1.96423200	2.58410800	-0.81684600
C	-3.04454300	3.49196300	-0.57776800
C	-2.17583400	1.22832700	-0.64937400
C	-2.94067400	4.89690600	-0.77845500
C	-4.30856500	2.96464500	-0.15504600
C	-3.40716300	0.71701200	-0.19849600
H	-1.37349100	0.53668500	-0.87790700
C	-4.01145500	5.73182300	-0.54536900
H	-2.01139200	5.32451900	-1.13064300
C	-5.39298600	3.85287200	0.06597300
C	-4.45865600	1.56858400	0.03371900
H	-3.52155200	-0.34982900	-0.05442700
C	-5.25196400	5.20838000	-0.11113800
H	-3.90520300	6.79982200	-0.71219000
H	-6.34565400	3.43305000	0.37449000
H	-5.41643200	1.17950500	0.36011200
H	-6.09127800	5.87528000	0.06224500

(TfO•1c)⊂AuCav-2

C	-4.49415300	3.26164900	2.50862800
C	-3.99826700	1.87941700	2.92454000
C	-3.87970600	3.70420600	1.18535700
H	-4.12013500	3.95392800	3.26353100
C	-4.67329600	0.70060800	2.59250500
C	-2.84399900	1.76050400	3.71570600
C	-4.38929600	3.27088800	-0.04218400
C	-2.78054600	4.57292100	1.15192200
C	-4.27128100	-0.55938900	3.05234500
H	-5.56489200	0.76963700	1.98045500
C	-2.40513000	0.53062000	4.19995100
O	-2.20664400	2.93057200	4.10405200
C	-3.85357500	3.65952600	-1.27287200
H	-5.23974700	2.59783600	-0.03851500
C	-2.21422100	4.99008100	-0.05071500
O	-2.23881600	5.02971700	2.35810600
C	-5.01945000	-1.84450800	2.70391800
C	-3.13530700	-0.61009600	3.87229300
H	-1.52511400	0.46498300	4.82553900
C	-1.01910400	3.29029000	3.51447100
C	-4.44710100	3.17396800	-2.59252200
C	-2.74063300	4.51270300	-1.24834700
H	-1.35637400	5.64989900	-0.05321800
C	-1.02724000	4.45280800	2.67485300
H	-4.78640100	-2.55909600	3.49348700
C	-4.47539800	-2.44233900	1.40882400
O	-2.78482700	-1.84910400	4.41083800
C	-3.92789900	1.77946200	-2.92005200
H	-4.05820900	3.83617300	-3.36640900
O	-2.16219000	4.90516400	-2.45701500
C	-4.90840300	-1.99396800	0.15688900
C	-3.49475600	-3.44718000	1.43041800
C	-1.63435000	-2.43402200	3.95754100
C	1.22964400	3.07642800	3.20089700
C	-4.57865300	0.61359500	-2.50506200
C	-2.74211200	1.63627500	-3.65716100
C	-0.95198900	4.31365300	-2.75453300
C	1.23193000	4.27395700	2.41607700
C	-4.40761900	-2.48870900	-1.05230900
H	-5.65216100	-1.20664800	0.12118800
C	-2.97933900	-3.98492800	0.25387600
O	-3.02483500	-3.91203900	2.65532000
C	-1.75571100	-3.52281900	3.02784300
C	-4.09899000	-0.66967300	-2.79296100
H	-5.50798600	0.70863100	-1.95661300
C	-2.20711600	0.38246400	-3.93731100
O	-2.14836800	2.78049000	-4.16623300
C	-0.94219400	3.18651100	-3.63746700
C	-4.85834700	-1.94023200	-2.40487400
C	-3.43434800	-3.49017400	-0.96429400
H	-2.19583100	-4.73105500	0.29626000
C	0.63251800	-2.63488100	3.89813100
C	-2.89372100	-0.74490600	-3.50257000
H	-1.27844700	0.29887000	-4.48685700
C	1.31052000	4.13180700	-2.51987200
H	-4.59181800	-2.69470500	-3.14917400
O	-2.77769100	-3.93146100	-2.13492800
C	0.51492200	-3.72431000	2.97754100
O	-2.34996100	-1.99888300	-3.83929700
C	1.31632600	3.00574900	-3.40293200
C	2.43850600	4.72966700	1.83789800
C	2.43804200	2.36872900	3.38458100
C	1.68666900	-4.34572400	2.48694500
C	1.91444000	-2.18406600	4.29075600
C	2.52957000	2.31984400	-3.64618800
C	2.51744700	4.53934000	-1.90384200
C	-6.38348100	-1.76625500	-2.47579000
H	-6.67581100	-1.41824500	-3.46977900
H	-6.76336700	-1.04509500	-1.74771200
C	-6.54419300	-1.66403300	2.67357000
H	-7.03182000	-2.62382700	2.48256300
H	-6.87883400	-0.96571800	1.90139100
C	-6.02691700	3.36707700	2.50158200
H	-6.43089400	3.08271500	3.47721300
H	-6.49690100	2.72812800	1.74831100
C	-5.97921000	3.27316000	-2.61844700
H	-6.28684600	4.30847800	-2.45043700
H	-6.46188400	2.66178200	-1.85046200
H	-6.36302500	2.95007000	-3.59029600
H	-6.32608200	4.39646800	2.28772200
H	-6.89399400	-1.28206700	3.63620500
H	-6.87732000	-2.72158200	-2.27990100
C	3.67788400	3.83645100	-2.13852200
C	3.68556600	2.71962400	-3.01208400
H	4.60851200	2.16209600	-3.13468000
H	4.59552300	4.10489700	-1.62593100
H	2.50575600	1.46023400	-4.30699100
H	2.48508900	5.38903300	-1.23156300
C	3.59995800	2.83187900	2.80396100
H	2.41837300	1.45395900	3.96664600
C	3.60075800	4.01208800	2.02279800
H	2.40679700	5.63381400	1.24019700
C	3.04074600	-2.79051100	3.77617200
H	1.97296200	-1.34911500	4.98056900
C	2.92489700	-3.87734300	2.87666800
H	1.58757100	-5.16584500	1.78755700
H	3.82140700	-4.33099400	2.47020700
H	4.02509000	-2.42341700	4.04521700
H	4.52608400	4.32247500	1.54917900
H	4.51608900	2.26207500	2.89803800
P	-1.62366200	-2.89021700	-2.66804200
N	-0.57088400	-3.88634700	-3.44316800
C	-0.90286200	-5.22647300	-3.93140100
C	0.58501500	-3.24684800	-4.08416300
H	-1.73860000	-5.63372300	-3.36425000
H	-0.03007300	-5.87079900	-3.78872900

H	-1.16435400	-5.20714100	-4.99633700
H	0.76143400	-2.25719500	-3.65412600
H	0.42069700	-3.12908800	-5.16138300
H	1.47498200	-3.85606000	-3.90648300
Au	-0.97349100	-1.65169500	-0.84256100
N	0.15093700	2.56332600	-3.96430000
N	0.14119600	4.77732600	-2.22301700
N	0.06383700	4.93779000	2.16089500
N	0.06602800	2.61437500	3.75569000
N	-0.48161300	-2.00637400	4.37811400
N	-0.71870700	-4.14682000	2.55404000
C	0.16364600	-0.96775100	1.12556600
C	-1.04812100	-0.75984300	1.20380700
H	-2.02070600	-0.47764300	1.55485900
C	1.60938100	-1.08726300	1.05917600
C	2.27726300	-0.24451200	-0.07545500
H	1.89911300	-2.13555300	0.98003000
H	2.01353800	-0.73401500	2.01298400
C	3.45824600	0.51378900	0.55664300
O	3.98830400	0.13926400	1.58863900
O	3.79449200	1.52965900	-0.19191100
H	4.70018200	1.96016400	0.05992900
C	2.81969700	-1.09500100	-1.26146200
H	1.96257000	-1.43439800	-1.84153600
H	3.37091300	-0.40239200	-1.90279600
C	1.23649400	0.72325100	-0.62660900
O	0.65841800	0.57039900	-1.68905500
O	0.92634000	1.65454800	0.27377600
C	-0.24452900	2.45338600	0.03154800
H	-0.92064800	2.29110700	0.87108200
H	0.06406600	3.49235000	-0.00973500
H	-0.71920200	2.15648100	-0.90117400
S	7.00633600	1.86049600	-0.72379300
O	8.09519100	2.69839700	-1.22634700
O	6.31851600	0.96824100	-1.68426000
O	6.03175900	2.59154000	0.17882700
C	7.80306100	0.67285500	0.46163700
F	8.58917700	-0.19193200	-0.20139600
F	8.54484300	1.31781300	1.36831100
F	6.86494000	-0.04107200	1.11092000
C	3.71821600	-2.23874300	-0.86497800
C	3.22652200	-3.57905400	-0.75066500
C	5.05175200	-1.98157700	-0.61141700
C	1.87801400	-3.94178400	-1.01944000
C	4.13325200	-4.62285500	-0.36165000
C	5.93420000	-3.00466900	-0.20089200
H	5.43798100	-0.97749400	-0.74856500
C	1.44354500	-5.24542600	-0.93273900
H	1.16730800	-3.17506200	-1.29899200
C	3.65327100	-5.95761000	-0.27036000
C	5.48656000	-4.29963200	-0.07764900
H	6.96806300	-2.74689000	0.00441300
C	2.34220200	-6.27057600	-0.55424600
H	0.40502500	-5.47940100	-1.14568800
H	4.35102800	-6.73715700	0.02377800
H	6.16171500	-5.09578600	0.22439000
H	1.99657900	-7.29736600	-0.48413000

(TfO•1c)⊂AuCav-3

C	-4.53562800	-2.88228800	2.19902800
C	-3.13805900	-2.70916800	2.78729000
C	-4.57338600	-2.28633900	0.79562900
H	-5.21056000	-2.28144000	2.80923600
C	-2.15748200	-3.70149200	2.78122100
C	-2.80819200	-1.48443500	3.37964600
C	-4.11287100	-3.03238300	-0.29283900
C	-5.05956600	-0.99889900	0.52246100
C	-0.89965400	-3.51695200	3.36624700
H	-2.38866500	-4.66307700	2.33860000
C	-1.57031900	-1.23785200	3.95967600
O	-3.86425800	-0.59548200	3.49024600
C	-4.14891400	-2.58733700	-1.61677800
H	-3.73634200	-4.03010300	-0.10069500
C	-5.05520100	-0.48650500	-0.77627000
O	-5.68364500	-0.25259500	1.52699400
C	0.15864800	-4.61819200	3.37793800
C	-0.62980400	-2.26784800	3.94849100
H	-1.35426800	-0.29729800	4.44941400
C	-3.94846700	0.64433100	2.95800700
C	-3.73626900	-3.52030300	-2.75239700
C	-4.60708000	-1.27760500	-1.83465200
H	-5.46148500	0.49783600	-0.97614400
C	-5.03547100	0.84469500	2.03249200
H	0.78520800	-4.43760000	4.25174600
C	1.05512800	-4.44976800	2.15660600
O	0.55782200	-2.12335000	4.64917200
C	-2.22263900	-3.49944900	-2.92645200
H	-4.16040700	-3.09845500	-3.66390400
O	-4.72937000	-0.78366000	-3.13746100
C	0.67794000	-4.91176100	0.89069800
C	2.25858700	-3.73906100	2.25724800
C	1.56054100	-1.30279700	4.19587200
C	-3.58853100	2.88653600	3.01912900
C	-1.34356800	-4.35304400	-2.25264700
C	-1.66409000	-2.54205400	-3.78119000
C	-3.78063900	0.11978800	-3.55815400
C	-4.78289600	3.09399300	2.26535100
C	1.42078700	-4.65349600	-0.26604200
H	-0.24370300	-5.47546400	0.79937500
C	3.01570200	-3.42823900	1.13213100
O	2.68829100	-3.31168700	3.50736600
C	2.72337800	-1.94022900	3.65896000
C	0.04932000	-4.26118900	-2.38666000
H	-1.75250900	-5.10975500	-1.59295400
C	-0.29577100	-2.41873300	-3.95951500
O	-2.54416400	-1.71257900	-4.45960300
C	-2.57223700	-0.38297900	-4.14797700
C	1.01691600	-5.18453600	-1.64372700
C	2.57311200	-3.87042100	-0.10447100
H	3.90065300	-2.80912900	1.21703900
C	2.56261900	0.72222100	3.93280800
C	0.54210100	-3.26912200	-3.24711400
H	0.11121300	-1.65026700	-4.60251200
C	-3.00091900	2.24420000	-3.83113900
H	1.93265900	-5.19953100	-2.24033800
O	3.28959800	-3.43179600	-1.23045200
C	3.73170400	0.07971200	3.41336900
O	1.90981700	-3.02678500	-3.34930300
C	-1.73494000	1.73055700	-4.24934900
C	-5.24623800	4.40943400	2.04727100
C	-2.84713900	3.99961100	3.47008900
C	4.80922800	0.85596500	2.93429300
C	2.51913700	2.13327400	3.99314600
C	-0.66619900	2.62063700	-4.50399300
C	-3.19152700	3.64279200	-3.76541500
C	0.50203700	-6.63078600	-1.57377500
H	0.29104200	-7.00421000	-2.57939600
H	-0.41489300	-6.72636900	-0.98602600
C	-0.42621300	-6.03109400	3.50381000
H	0.38323500	-6.76157000	3.58424800
H	-1.04168800	-6.32017400	2.64685800
C	-5.04286400	-4.33006600	2.24291800
H	-5.04259700	-4.69438900	3.27384200
H	-4.43440400	-5.01908400	1.65057800
C	-4.30517400	-4.93719500	-2.58817300
H	-5.39481300	-4.89807700	-2.51020500
H	-3.93055200	-5.45043700	-1.69825900
H	-4.04022000	-5.54731100	-3.45605300
H	-6.06434200	-4.38040100	1.85677500
H	-1.05029200	-6.10486400	4.39869700
H	1.25584100	-7.27376600	-1.11238600
C	-2.13895600	4.49116500	-4.02935600
C	-0.86753400	3.97849900	-4.38176300
H	-0.04757500	4.66670000	-4.55850800
H	-2.27313500	5.56355500	-3.93913100
H	0.29032500	2.20506200	-4.80246700
H	-4.16231000	4.01301700	-3.46140700
C	-3.30616300	5.27557400	3.21830600
H	-1.94058900	3.80723900	4.03167400
C	-4.51870000	5.48093700	2.52056900

H	-6.16703900	4.54059600	1.48920400
C	3.58586300	2.86830000	3.51687300
H	1.62782900	2.60235200	4.39599900
C	4.72601800	2.23058600	2.97304700
H	5.66162500	0.36333000	2.48358400
H	5.53214900	2.81950100	2.55250200
H	3.54734000	3.95242800	3.53458400
H	-4.87027800	6.49319900	2.34855000
H	-2.74483000	6.13448100	3.57246900
P	2.58212200	-2.20870100	-2.05665200
N	3.84192200	-1.34374500	-2.61031500
C	5.20973300	-1.86507200	-2.76106100
C	3.57347800	-0.05842100	-3.26157400
H	5.34288700	-2.73732300	-2.12355100
H	5.90794500	-1.08570200	-2.44820400
H	5.39633600	-2.14508500	-3.80474100
H	2.59646100	0.32438000	-2.95464800
H	3.59032600	-0.16159500	-4.35338000
H	4.33164800	0.65767800	-2.94403300
Au	0.82278200	-1.30127300	-0.81609200
N	-1.56407300	0.38647400	-4.43450300
N	-4.00260900	1.39531300	-3.43917100
N	-5.46117900	2.03193400	1.72461600
N	-3.20444100	1.62179300	3.38275100
N	1.47612400	-0.01139600	4.33054200
N	3.77831400	-1.28001600	3.29523600
C	-0.70385800	-0.01956700	0.69839900
C	-1.17957100	-1.01544200	0.15204100
H	-1.88658800	-1.77373700	-0.12744400
C	-0.24473300	1.19981600	1.36040800
C	0.76234300	2.02242100	0.51732200
H	0.20176500	0.93284600	2.32073600
H	-1.13261600	1.80434100	1.56221200
C	2.05650400	1.19567700	0.37216000
O	2.23274200	0.18910800	1.05070500
O	2.87662100	1.64178900	-0.53896800
H	3.77740500	1.12855800	-0.48058000
C	0.19670000	2.38204300	-0.89916200
H	-0.02112700	1.44286400	-1.41742800
H	0.99900700	2.85983300	-1.45800500
C	1.13547400	3.34487800	1.20343100
O	2.11914900	3.98952500	0.92443800
O	0.20821300	3.75048100	2.09082300
C	0.44222900	5.07114200	2.61833800
H	-0.38376400	5.26599000	3.29728300
H	1.39659100	5.10400500	3.14621900
H	0.45187600	5.79710000	1.80324700
S	6.40479800	1.11202900	-0.53012300
O	5.05907000	0.43104700	-0.42167400
O	7.17219800	0.64936100	-1.69838300
O	7.11118100	1.24029900	0.75258700
C	5.91958700	2.85338700	-0.96553400
F	5.16717600	2.85964800	-2.08706900
F	5.19449300	3.40750200	0.01872000
F	6.99978000	3.60996600	-1.17998600
C	-1.05293700	3.22730900	-0.85296600
C	-0.99641900	4.65973900	-0.89457200
C	-2.28219700	2.60655600	-0.74141600
C	0.21096700	5.38872500	-1.07999200
C	-2.21814300	5.40308100	-0.77505000
C	-3.48200100	3.34115800	-0.63872300
H	-2.33393200	1.52241700	-0.75395000
C	0.21279100	6.76573600	-1.12748100
H	1.15238100	4.86307100	-1.16810700
C	-2.17983500	6.82159000	-0.82065300
C	-3.44798600	4.71256300	-0.64300500
H	-4.42559900	2.81439400	-0.56807500
C	-0.99221900	7.49389800	-0.99234200
H	1.14974900	7.29505300	-1.27015600
H	-3.11502800	7.36671100	-0.72366600
H	-4.36350500	5.28901800	-0.56286900
H	-0.97605200	8.57888300	-1.02947000

(TfO•1c)⊂AuCav-4

C	2.56770100	4.17947900	2.91345800
C	1.42736900	3.24677200	3.31077900
C	2.95403300	3.91635900	1.46215700
H	3.42948900	3.89978500	3.52009100
C	0.08167200	3.61527400	3.31725900
C	1.73859700	1.93905200	3.70251400
C	2.21604100	4.49568800	0.42732300
C	4.03944800	3.10914700	1.09200600
C	-0.93163800	2.74253100	3.72836000
H	-0.18640600	4.62434000	3.02752100
C	0.77323100	1.02527500	4.10732700
O	3.09483700	1.68089900	3.81829000
C	2.52221200	4.34892400	-0.92757600
H	1.37596200	5.12591700	0.69432800
C	4.34141000	2.87573600	-0.25075200
O	4.91911800	2.62165000	2.06429000
C	-2.40173600	3.15350300	3.75726600
C	-0.55615200	1.44769700	4.11843600
H	1.04230900	0.03527400	4.45176300
C	3.81688100	0.75687400	3.14577500
C	1.73572400	5.13039800	-1.97634300
C	3.58804400	3.49291000	-1.24976500
H	5.19921000	2.27117600	-0.52017600
C	4.89612600	1.28038800	2.34501400
H	-2.87951300	2.54653400	4.52651400
C	-3.04708500	2.76536400	2.43194800
O	-1.53831000	0.63441500	4.66133400
C	0.43931700	4.40243100	-2.30611000
H	2.33977200	5.11969300	-2.88385100
O	3.98482700	3.32995300	-2.58207900
C	-2.90947500	3.55916000	1.28929800
C	-3.73855300	1.55292900	2.30194300
C	-1.97961500	-0.49351200	4.01877100
C	4.63601100	-1.34366400	2.86828100
C	-0.77079400	4.60599600	-1.63452800
C	0.46269300	3.43547800	-3.31737200
C	3.62398800	2.15590900	-3.20235600
C	5.80332500	-0.80360500	2.24963900
C	-3.39462300	3.17791700	0.03514400
H	-2.38547200	4.50423800	1.37633900
C	-4.21562000	1.11358600	1.06949100
O	-3.94348600	0.77327300	3.43523000
C	-3.28902800	-0.44048900	3.43919100
C	-1.92730100	3.87000700	-1.92814400
H	-0.81635600	5.35793400	-0.85503100
C	-0.65471700	2.68889600	-3.65250300
O	1.66212800	3.26120600	-3.99206100
C	2.34122400	2.08671600	-3.84428600
C	-3.25560300	4.07118300	-1.19901700
C	-4.01723500	1.92279000	-0.04189500
H	-4.69833800	0.14899000	0.97293600
C	-1.83063800	-2.69257700	3.46109600
C	-1.82968000	2.90837600	-2.94288800
H	-0.60463900	1.93016200	-4.42141700
C	4.01428100	-0.00694000	-3.80825700
H	-4.03180600	3.74573600	-1.89628300
O	-4.40011400	1.40580500	-1.29044800
C	-3.15227400	-2.64534900	2.91570200
O	-2.90202500	2.07617000	-3.23944100
C	2.67229400	-0.12103400	-4.28443600
C	6.87498600	-1.66214500	1.92235100
C	4.53562500	-2.73626300	3.06974800
C	-3.69400700	-3.78629600	2.28507800
C	-1.08979200	-3.89274700	3.37861400
C	2.19563600	-1.36890500	-4.74779500
C	4.87366600	-1.12558300	-3.89143600
C	-3.52906800	5.54831600	-0.87679400
H	-3.49184100	6.14600100	-1.79156600
H	-2.80452900	5.97127900	-0.17568600
C	-2.61983800	4.62800000	4.12163600
H	-3.69034200	4.83704300	4.19636100
H	-2.20362100	5.32019700	3.38388400
C	2.27270400	5.66005500	3.18950600
H	2.04542500	5.80455100	4.24926500
H	1.42556000	6.04577400	2.61557300
C	1.52181800	6.59825300	-1.57901900

H	2.48478100	7.08199000	-1.39554100
H	0.91828700	6.71527500	-0.67475400
H	1.01269200	7.13184700	-2.38638300
H	3.14457300	6.26841000	2.93491400
H	-2.15424500	4.85293700	5.08522100
H	-4.52005200	5.65570300	-0.42848100
C	4.39037400	-2.32909800	-4.35575300
C	3.04237200	-2.45616700	-4.76793500
H	2.67874800	-3.42093400	-5.10613400
H	5.03857500	-3.19827000	-4.38116500
H	1.16771100	-1.43289400	-5.08837800
H	5.89199400	-1.01530100	-3.54072400
C	5.58233600	-3.55869200	2.70947600
H	3.63039300	-3.11102000	3.53096000
C	6.76417700	-3.01741900	2.15329600
H	7.76066900	-1.22526500	1.47367300
C	-1.63710400	-4.98946900	2.74365400
H	-0.09183100	-3.90911300	3.80303100
C	-2.93611600	-4.93265800	2.18582600
H	-4.67698400	-3.72366800	1.83939400
H	-3.33761900	-5.78901700	1.65546200
H	-1.06234000	-5.90513900	2.65192500
H	7.58407300	-3.67994300	1.89482200
H	5.51080800	-4.63010100	2.86774400
P	-3.16718400	0.80360200	-2.19041800
N	-3.84756400	-0.37349000	-3.07802400
C	-5.19283700	-0.24091800	-3.65610200
C	-3.02396300	-1.46352500	-3.60921700
H	-5.76721600	0.49376400	-3.09326900
H	-5.68627100	-1.21084900	-3.57389000
H	-5.12914300	0.07013500	-4.70584100
H	-2.74951700	-1.27291800	-4.65482300
H	-3.59980200	-2.38719700	-3.53235900
H	-2.11557000	-1.57453400	-3.01372600
Au	-1.27168800	0.55895100	-0.85879400
N	1.85911400	0.97755700	-4.32177600
N	4.44923700	1.15117100	-3.21836200
N	5.87556500	0.53026400	1.94053900
N	3.65077600	-0.51691600	3.34333700
N	-1.26647700	-1.58122900	4.02903100
N	-3.86648900	-1.48132800	2.92737500
C	0.70442800	0.01179000	0.68350400
C	0.58119500	1.16974300	0.28483800
H	0.78670800	2.21542100	0.15371000
C	0.94591300	-1.34593800	1.16991200
C	0.48952700	-2.45131700	0.18266900
H	0.43990200	-1.46993200	2.12972700
H	2.02055700	-1.43420900	1.34644500
C	-1.04509900	-2.35197000	0.05682100
O	-1.66334800	-1.56582000	0.76907700
O	-1.57892200	-3.10064400	-0.86941300
H	-2.60348100	-2.94453500	-0.85197300
C	1.15246800	-2.29094100	-1.23049200
H	0.84298100	-1.32199100	-1.63532300
H	0.72050500	-3.04989800	-1.88061100
C	0.84209100	-3.85967800	0.68047100
O	0.37366400	-4.86904500	0.20842800
O	1.78318100	-3.86127700	1.64325200
C	2.24010200	-5.18011400	2.00446800
H	2.94435400	-5.03724000	2.82027800
H	1.39933600	-5.79790500	2.32262900
H	2.72928900	-5.64669700	1.14704000
S	-5.24800200	-3.23884200	-1.11420500
O	-5.37001100	-4.50387700	-0.37581100
O	-5.59536400	-3.25805700	-2.54884800
O	-3.99355500	-2.46128600	-0.80870700
C	-6.50340600	-2.10992900	-0.34604200
F	-7.74288100	-2.56081600	-0.56526500
F	-6.30861000	-2.01490100	0.97915300
F	-6.40518200	-0.86916200	-0.86416400
C	2.66161700	-2.35491100	-1.18568800
C	3.37569500	-3.58521900	-1.37150500
C	3.37312900	-1.20172900	-0.91318900
C	2.74599300	-4.80923700	-1.73015700
C	4.80317900	-3.58480400	-1.21972600
C	4.77634400	-1.20537000	-0.77100400
H	2.84283900	-0.25982200	-0.81750400
C	3.47753200	-5.96301100	-1.90884000
H	1.67158400	-4.84951100	-1.84811700
C	5.52589000	-4.79280500	-1.40413700
C	5.47549000	-2.37688700	-0.91185800
H	5.29425700	-0.27615100	-0.56780200
C	4.88177600	-5.96061100	-1.74028300
H	2.96883800	-6.88218900	-2.18235000
H	6.60526800	-4.77235700	-1.27913200
H	6.55476200	-2.39307300	-0.80731100
H	5.44567200	-6.87759500	-1.88182500

(TfO•1c)⊂AuCav-5

C	-4.33310800	4.17614200	0.90011700
C	-4.26877100	2.88973300	1.71875300
C	-3.36728600	4.15620700	-0.28074600
H	-3.98336800	4.96873700	1.56241000
C	-5.09683100	1.78952400	1.47731700
C	-3.38048000	2.79886600	2.80148300
C	-3.69457800	3.52439000	-1.48392100
C	-2.12310400	4.79926400	-0.21446900
C	-5.09486300	0.64313900	2.28264800
H	-5.79158700	1.83777600	0.64718600
C	-3.34260400	1.68473100	3.63521200
O	-2.58925200	3.90394400	3.07999500
C	-2.85359600	3.51652800	-2.60009000
H	-4.64875800	3.01366500	-1.55428100
C	-1.25595700	4.82709500	-1.30447900
O	-1.74650100	5.43394500	0.97624800
C	-6.01910100	-0.54363300	2.01832000
C	-4.20703500	0.62543800	3.36794700
H	-2.65833400	1.64468300	4.47180500
C	-1.25802600	3.85831200	2.74484100
C	-3.25513900	2.80789100	-3.89206900
C	-1.61888500	4.17175400	-2.47996400
H	-0.29958500	5.32879300	-1.23191300
C	-0.80753100	4.73315700	1.70256100
H	-6.12818800	-1.06504900	2.96964200
C	-5.36044000	-1.53127600	1.05894600
O	-4.23845000	-0.47755300	4.22951900
C	-2.96942600	1.31710700	-3.75935500
H	-2.59055100	3.18377100	-4.67020700
O	-0.75016900	4.19072700	-3.57082900
C	-5.38880100	-1.35310100	-0.32857400
C	-4.66243500	-2.64520700	1.54740800
C	-3.16895200	-1.32735000	4.18419000
C	0.87273100	3.10736300	3.04506900
C	-3.91603400	0.40074800	-3.29594600
C	-1.69433800	0.82864700	-4.08315200
C	0.37434000	3.39491800	-3.46157300
C	1.33494800	4.03015600	2.05402200
C	-4.73846700	-2.21210400	-1.22231800
H	-5.90938800	-0.49065300	-0.72878800
C	-4.03074200	-3.54533300	0.69734300
O	-4.56308300	-2.83135900	2.92608700
C	-3.33167900	-2.56815800	3.47487500
C	-3.63921400	-0.96190000	-3.13178600
H	-4.91640600	0.75512500	-3.07969000
C	-1.35065700	-0.50506100	-3.87898900
O	-0.81076700	1.70496200	-4.68579700
C	0.33495200	2.07703400	-4.01758600
C	-4.71636600	-1.96854800	-2.72842700
C	-4.06053400	-3.30542300	-0.67246900
H	-3.47040900	-4.37655000	1.10440200
C	-1.01578600	-1.89050600	4.66726300
C	-2.32705400	-1.37076300	-3.40193700
H	-0.34568600	-0.84711600	-4.09096700
C	2.50763900	3.01214700	-2.74891500
H	-4.43454900	-2.91874800	-3.18919300
O	-3.24233900	-4.10633800	-1.48615300
C	-1.16698500	-3.10942600	3.93552700
O	-1.96376000	-2.72965600	-3.27222000
C	2.45764500	1.68631300	-3.29141800
C	2.70230200	4.04434700	1.69706900

C	1.79642900	2.23153600	3.66089300
C	-0.06617700	-3.98921300	3.81587000
C	0.22916700	-1.58476700	5.26513800
C	3.56492000	0.82203200	-3.13235500
C	3.66993000	3.43620900	-2.06454500
C	-6.10757100	-1.60593500	-3.27075900
H	-6.06727600	-1.47255300	-4.35481400
H	-6.50541700	-0.68467200	-2.83758300
C	-7.42140500	-0.12195000	1.55509400
H	-8.06103600	-1.00239100	1.44986300
H	-7.41666700	0.39754500	0.59293500
C	-5.76485100	4.53129000	0.46920200
H	-6.42039400	4.58783400	1.34274300
H	-6.19635800	3.80319100	-0.22372600
C	-4.69720400	3.12181200	-4.31359100
H	-4.81616700	4.19901100	-4.45576400
H	-5.43930500	2.80781900	-3.57367600
H	-4.93415100	2.61924600	-5.25570600
H	-5.77008500	5.50191900	-0.03337300
H	-7.87253100	0.54967400	2.29014900
H	-6.81509800	-2.40734700	-3.04262200
C	4.73286400	2.57128300	-1.92024400
C	4.68122400	1.25883300	-2.45292000
H	5.52416100	0.59840700	-2.27949900
H	5.61245800	2.87668700	-1.36634900
H	3.49426800	-0.18227800	-3.53194500
H	3.68712400	4.44248900	-1.65979100
C	3.12335500	2.25916500	3.28387800
H	1.42742800	1.54960300	4.42086600
C	3.57933200	3.16602500	2.29498400
H	3.02254900	4.73648200	0.92627100
C	1.28741400	-2.45972400	5.13461600
H	0.31740700	-0.65223500	5.81193200
C	1.13891700	-3.66572900	4.40703700
H	-0.20388700	-4.91225200	3.26252800
H	1.98570400	-4.33781900	4.31402200
H	2.24809700	-2.21971200	5.57721700
H	4.61974700	3.13124600	1.99230100
H	3.82977700	1.55960100	3.71537300
P	-1.76944400	-3.40492900	-1.80731200
N	-0.70505600	-4.60715900	-2.13041600
C	-0.01257100	-5.26228400	-1.01998500
C	-0.40243900	-5.13559300	-3.46269500
H	-0.27436800	-4.78473900	-0.07212500
H	1.06934200	-5.17382900	-1.16472200
H	-0.29285600	-6.31958600	-0.96089200
H	-0.91438800	-4.55039300	-4.22416200
H	-0.71949800	-6.18170200	-3.53700700
H	0.67712400	-5.06851300	-3.63215500
Au	-1.34333300	-1.91347800	-0.09467700
N	1.33636500	1.25259200	-3.94326600
N	1.43103700	3.85108700	-2.85575500
N	0.45022500	4.83315400	1.38870300
N	-0.45194600	3.06006300	3.38250600
N	-2.05312400	-1.00626300	4.76902600
N	-2.36191900	-3.42464700	3.34637900
C	-0.32835000	-0.71982100	1.71310700
C	-1.49923000	-0.39069600	1.51435800
H	-2.45035300	0.09818100	1.59444300
C	1.08994200	-0.93630800	1.96695700
C	1.99779100	-0.86657500	0.68498200
H	1.25120700	-1.88636600	2.47675800
H	1.39282500	-0.14308500	2.65615400
C	3.35443600	-0.31603900	1.17215000
O	3.77923300	-0.63943100	2.27110800
O	3.92330900	0.45375800	0.28965900
H	4.81310800	0.87832000	0.61859200
C	2.16569600	-2.30513400	0.11224400
H	2.68352000	-2.87927000	0.88718000
H	1.15787500	-2.72873300	0.03708500
C	1.28382100	0.06879700	-0.28038800
O	0.72357300	-0.27970500	-1.30288000
O	1.17674100	1.28895200	0.25123700
C	0.23156800	2.18464600	-0.35628600
H	-0.60475200	2.30068600	0.33533000
H	0.73185300	3.13429800	-0.50314600
H	-0.12073100	1.77764800	-1.30003100
S	7.34644400	1.22338000	0.58150600
O	8.37436200	2.26044200	0.69048400
O	7.29589900	0.41924000	-0.65165400
O	5.97461900	1.68906200	1.02788500
C	7.75501100	0.00068300	1.91973400
F	8.82263900	-0.73939000	1.57264200
F	8.02649800	0.62434900	3.07210400
F	6.72316300	-0.83781500	2.13205200
C	2.84431400	-2.48333100	-1.23224600
C	4.26820800	-2.56302300	-1.36702100
C	2.05335000	-2.61413100	-2.36001200
C	5.16783200	-2.49255700	-0.26839400
C	4.82642800	-2.72705700	-2.68250800
C	2.60257900	-2.79419500	-3.64627400
H	0.98066500	-2.53880400	-2.25759700
C	6.52871400	-2.54738000	-0.45902200
H	4.79177400	-2.36433400	0.73874600
C	6.23702100	-2.77367900	-2.84153100
C	3.96787400	-2.84057400	-3.80494600
H	1.94271800	-2.87053500	-4.50552700
C	7.07237700	-2.67905700	-1.75626900
H	7.19334800	-2.45756500	0.38984000
H	6.64048700	-2.87808600	-3.84506900
H	4.41038700	-2.96150900	-4.78990800
H	8.14917600	-2.68972300	-1.88728500

(TfO•1c)⊂AuCav-6

C	-3.70643000	-4.16121900	2.13937300
C	-2.18198300	-4.16261900	2.07151000
C	-4.31545100	-2.96675500	1.41307900
H	-3.95994200	-4.03489600	3.19235600
C	-1.47967300	-4.74977900	1.01408800
C	-1.42241000	-3.60627100	3.10898700
C	-4.61548100	-2.99689300	0.04654900
C	-4.62800600	-1.79331800	2.11133000
C	-0.08514300	-4.84484300	0.98789100
H	-2.04286500	-5.16468100	0.18629200
C	-0.03368500	-3.68301900	3.13746300
O	-2.10082700	-3.03750500	4.19165900
C	-5.23040600	-1.93315600	-0.61714400
H	-4.36586400	-3.88753400	-0.51821400
C	-5.24786000	-0.70716900	1.49950200
O	-4.38439300	-1.75621000	3.48909400
C	0.67692700	-5.44071700	-0.19257900
C	0.61479400	-4.32110400	2.08358700
H	0.52893500	-3.27960600	3.96932800
C	-2.12058500	-1.67720500	4.29172900
C	-5.49509200	-1.93140600	-2.12236500
C	-5.54556600	-0.80061200	0.14404600
H	-5.50325200	0.17934700	2.06480300
C	-3.34025900	-0.99745000	3.93299000
H	1.64867000	-5.75602400	0.18729800
C	0.93631500	-4.32477000	-1.19956700
O	1.98840100	-4.53050600	2.13649900
C	-4.25921400	-1.34677200	-2.79667000
H	-6.31562300	-1.23620000	-2.30084600
O	-6.24747000	0.21276500	-0.50084900
C	-0.01187500	-3.97167000	-2.16228300
C	2.11458600	-3.56481300	-1.14844800
C	2.82654700	-3.45077100	2.06725700
C	-1.18973900	0.31619600	4.87931000
C	-3.15357100	-2.14329300	-3.10588800
C	-4.14508200	0.02418800	-3.06292600
C	-5.68763300	1.44507200	-0.66052200
C	-2.40540200	0.98754300	4.54496300
C	0.16739400	-2.89680400	-3.03487700
H	-0.92429900	-4.55212600	-2.23336300
C	2.32440100	-2.46522900	-1.97656800
O	3.11251900	-3.96494700	-0.26067600
C	3.41988900	-3.14557700	0.79317000
C	-1.94774700	-1.63268700	-3.59176800
H	-3.24099700	-3.21551800	-2.97803900
C	-2.93631100	0.58149200	-3.47310100

O	-5.28184200	0.83962700	-2.96193100
C	-5.27516600	1.80704500	-1.99059200
C	-0.84183300	-2.54107500	-4.11031500
C	1.33129000	-2.13573700	-2.89350000
H	3.21996200	-1.86237300	-1.89834200
C	4.01352800	-1.76494200	3.02384000
C	-1.82413700	-0.23738100	-3.66627900
H	-2.86131000	1.64217400	-3.66514600
C	-5.13307200	3.52304500	0.07912400
H	-0.29935700	-1.95461400	-4.85603300
O	1.50718200	-1.01389300	-3.70929400
C	4.59012800	-1.44671200	1.75346600
O	-0.64457200	0.37321500	-4.06580000
C	-4.83906800	3.92143600	-1.26059300
C	-2.49368100	2.38950800	4.70411600
C	-0.09052900	1.06501500	5.36081400
C	5.50775000	-0.37678000	1.65296900
C	4.39142100	-1.02646200	4.16754700
C	-4.40335400	5.24188100	-1.51290200
C	-4.94964300	4.44104600	1.13729900
C	-1.38954900	-3.78245000	-4.83209700
H	-2.07389700	-3.48385200	-5.63073100
H	-1.93250700	-4.45896300	-4.16682600
C	-0.00882300	-6.66891400	-0.80447100
H	0.60150800	-7.07132900	-1.61743900
H	-0.99852300	-6.44991900	-1.21520600
C	-4.31080800	-5.49753800	1.68024600
H	-3.90059800	-6.31773300	2.27556500
H	-4.10796100	-5.72131700	0.62930200
C	-5.91220400	-3.30041800	-2.67286400
H	-6.81941700	-3.64609600	-2.16978500
H	-5.14832400	-4.07096400	-2.53615500
H	-6.11582200	-3.22693800	-3.74457000
H	-5.39641800	-5.48123600	1.80950900
H	-0.13315400	-7.44631700	-0.04559300
H	-0.56390200	-4.34854900	-5.27141700
C	-4.49871300	5.71513400	0.86620200
C	-4.23254200	6.11920700	-0.46383600
H	-3.87903500	7.12716600	-0.65501500
H	-4.34848000	6.42046600	1.67743600
H	-4.18446000	5.52355600	-2.53616200
H	-5.17069100	4.10784700	2.14546500
C	-0.20233300	2.43306900	5.50530700
H	0.82381300	0.53565800	5.60651400
C	-1.40846700	3.09721400	5.17804900
H	-3.42320400	2.87594700	4.43016500
C	5.30641000	-0.00118200	4.04505300
H	3.94733700	-1.29587500	5.11983100
C	5.85476100	0.33080900	2.78529000
H	5.91237900	-0.12817800	0.68134500
H	6.54818600	1.16082700	2.70381400
H	5.60722500	0.56139700	4.92275300
H	-1.47355400	4.17418700	5.29420000
H	0.64461700	3.00659300	5.86735500
P	0.82326100	0.41662600	-3.25354600
N	1.68286600	1.49860200	-4.11233300
C	2.24221300	1.21990400	-5.43501000
C	1.87091000	2.86307000	-3.61530300
H	2.07811100	0.17682600	-5.70377000
H	3.31830000	1.40725900	-5.39589200
H	1.77219000	1.86263800	-6.18907700
H	1.30632100	3.57257800	-4.23041500
H	2.93711500	3.09514600	-3.63669700
H	1.52854300	2.94898000	-2.58475800
Au	0.95635300	0.57195700	-0.94096000
N	-4.90208100	3.01747200	-2.28816100
N	-5.59716600	2.26428100	0.34429500
N	-3.47563900	0.29069200	4.05207100
N	-1.07677300	-1.04027000	4.73486100
N	3.10731500	-2.78392800	3.14602900
N	4.26599300	-2.17438100	0.63975900
C	1.25756600	0.22120400	1.38017900
C	0.68516100	-0.74177100	0.86424900
H	0.18714500	-1.66200400	0.63746100
C	1.94467200	1.06004600	2.37933400

C	2.22807500	2.55868400	2.08240700
H	2.90293900	0.58042100	2.58458900
H	1.35749200	0.99054200	3.29790900
C	3.02306700	2.60666300	0.76763300
O	2.47603300	2.70675500	-0.32281500
O	4.30664700	2.42960000	0.95806700
H	4.79758600	2.22806500	0.08021200
C	0.96075800	3.47280000	2.08252200
H	1.31049700	4.48906400	1.90196300
H	0.57810800	3.45497800	3.10769800
C	3.08602500	3.16777300	3.19867300
O	3.55633800	4.28027700	3.13859000
O	3.17048700	2.38384900	4.29427900
C	3.91896300	2.94729700	5.38427100
H	3.89300200	2.19882400	6.17537100
H	4.94629400	3.14863100	5.07409700
H	3.46240000	3.88223200	5.71742000
S	4.88581600	0.94599700	-2.13873600
O	4.71865200	1.61561400	-3.43668500
O	3.70321400	0.24757900	-1.58169200
O	5.62767600	1.74913500	-1.10778600
C	6.06259100	-0.44311700	-2.50557900
F	5.44512600	-1.35189800	-3.27945700
F	7.14401500	0.00549200	-3.15033000
F	6.45992200	-1.05462300	-1.37948300
C	-0.16726700	3.08928700	1.16465700
C	-0.35916600	3.70369500	-0.11439700
C	-1.04576100	2.11295800	1.58979100
C	0.41847400	4.79810100	-0.58109500
C	-1.39349300	3.19706300	-0.96553900
C	-2.06622100	1.61604900	0.75571300
H	-0.91655100	1.68929800	2.57608300
C	0.18114300	5.36965900	-1.80944900
H	1.22234400	5.18426200	0.03166200
C	-1.59751900	3.79513200	-2.23880900
C	-2.20709100	2.12599500	-0.51028600
H	-2.72500900	0.83006400	1.11024900
C	-0.83597300	4.86240600	-2.65059900
H	0.78719400	6.20743500	-2.14014800
H	-2.40265600	3.42789200	-2.86231900
H	-2.94535300	1.71558000	-1.17925200
H	-1.01501500	5.31841700	-3.61966400

(TfO•1c)⊂AuCav-7

C	-3.97129400	3.79685600	2.52195000
C	-3.82891200	2.31838900	2.87184000
C	-3.20273100	4.16381300	1.25737200
H	-3.49204800	4.34266700	3.33536900
C	-4.73078300	1.34360600	2.43295500
C	-2.78669000	1.89941100	3.71464300
C	-3.72824900	3.92662600	-0.01634100
C	-1.94170700	4.77208000	1.32828900
C	-4.65704300	0.00387500	2.83467300
H	-5.53974200	1.64489700	1.77786800
C	-2.67945000	0.58275100	4.15428100
O	-1.91026500	2.86308600	4.19044800
C	-3.06034500	4.26993900	-1.19483000
H	-4.69852400	3.44796400	-0.09272800
C	-1.24477400	5.14442400	0.18103000
O	-1.36727500	5.00552300	2.58192400
C	-5.64687600	-1.05723200	2.35738200
C	-3.62472300	-0.34274300	3.71757500
H	-1.87976200	0.28639000	4.81933200
C	-0.65132400	2.95200800	3.64249900
C	-3.65808000	3.97972500	-2.56920200
C	-1.80176400	4.87419800	-1.06618700
H	-0.26871300	5.60542600	0.25804300
C	-0.34511300	4.12775500	2.88431300
H	-5.64111500	-1.84358900	3.11253500
C	-5.15913700	-1.70257000	1.06187700
O	-3.58843200	-1.65616400	4.19244400
C	-3.39010400	2.52707500	-2.94358100
H	-3.10452100	4.59034800	-3.28279200
O	-1.10315700	5.22657700	-2.22222500
C	-5.37458300	-1.10595200	-0.18509800

C	-4.44384300	-2.91048900	1.08174800
C	-2.56261800	-2.44386100	3.74747900
C	1.48005700	2.19596200	3.34066600
C	-4.28128500	1.49462200	-2.64057800
C	-2.19878000	2.18502100	-3.60236600
C	0.00009000	4.45438400	-2.51375500
C	1.80473000	3.40304900	2.64136800
C	-4.89816000	-1.64515600	-1.38576600
H	-5.90741000	-0.16273900	-0.22055100
C	-3.97897900	-3.50230400	-0.08907700
O	-4.16572200	-3.51798900	2.30361600
C	-2.86256900	-3.42608900	2.74272800
C	-4.03299500	0.15391200	-2.95730500
H	-5.22130700	1.74794100	-2.16616800
C	-1.87987700	0.85980100	-3.88660400
O	-1.38232100	3.21618200	-4.04018400
C	-0.14498500	3.38937200	-3.46179500
C	-5.07694900	-0.94178700	-2.72892700
C	-4.19947000	-2.85358700	-1.29929100
H	-3.39641500	-4.41315300	-0.04007300
C	-0.38056700	-3.09358600	3.73451700
C	-2.79783900	-0.13012900	-3.55612300
H	-0.93802700	0.61982400	-4.36269700
C	2.19301100	3.89184900	-2.23675700
H	-4.90165500	-1.69607600	-3.49985200
O	-3.54768800	-3.36112400	-2.44456700
C	-0.67729300	-4.07317100	2.73514200
O	-2.47706300	-1.45423000	-3.92616800
C	2.04821800	2.83972700	-3.19589000
C	3.10280400	3.57446300	2.10772700
C	2.46999800	1.19799200	3.49109800
C	0.36582700	-4.87653000	2.22378200
C	0.95048900	-2.93655500	4.18650700
C	3.15088500	2.00209100	-3.47883300
C	3.43051100	4.06608100	-1.57457400
C	-6.51709600	-0.44065400	-2.92218300
H	-6.62533500	0.02562600	-3.90470100
H	-6.81544200	0.29456600	-2.17056400
C	-7.08512700	-0.53080200	2.24314500
H	-7.76102100	-1.34377500	1.96442800
H	-7.19457100	0.25883700	1.49457700
C	-5.43780600	4.25184900	2.45869000
H	-5.94898400	4.01555400	3.39613900
H	-5.99767700	3.77945800	1.64627700
C	-5.13865300	4.37352300	-2.66453600
H	-5.25250300	5.44025900	-2.45521000
H	-5.77325600	3.83393400	-1.95551500
H	-5.51985200	4.17443700	-3.67015400
H	-5.48342900	5.33242900	2.30020300
H	-7.41071900	-0.12185400	3.20316200
H	-7.21393700	-1.28007900	-2.85420000
C	4.48100100	3.21749600	-1.84853800
C	4.34092100	2.18666200	-2.80955300
H	5.17890500	1.51967300	-2.97879500
H	5.41950300	3.31235400	-1.31466700
H	3.01557200	1.20643500	-4.20309700
H	3.51084900	4.86525900	-0.84637200
C	3.72743400	1.38811200	2.95613700
H	2.20801800	0.29185000	4.02811100
C	4.04307600	2.57827900	2.25482200
H	3.31534500	4.49451800	1.57500600
C	1.95292500	-3.72035500	3.65370200
H	1.14827100	-2.18039100	4.93854900
C	1.65899500	-4.69378700	2.66889400
H	0.12679600	-5.61107400	1.46515800
H	2.46157300	-5.28251100	2.23845500
H	2.97978500	-3.57967100	3.97346600
H	5.03686700	2.67472600	1.83256200
H	4.48943500	0.62220100	3.02471500
P	-2.14787800	-2.56753700	-2.78378600
N	-1.31802600	-3.69406200	-3.66229300
C	-1.39157700	-5.12779900	-3.36583900
C	-0.09104400	-3.27127600	-4.35309000
H	-2.31204800	-5.35658700	-2.83135600
H	-0.52810200	-5.45299700	-2.77374500
H	-1.39459300	-5.67976000	-4.31109200
H	-0.08349500	-2.18950300	-4.49043600
H	-0.06013600	-3.74097700	-5.34079700
H	0.79797700	-3.56857800	-3.78502300
Au	-1.46232900	-1.61505400	-0.79790500
N	0.84441900	2.62163000	-3.80751900
N	1.13367500	4.70079200	-1.92620100
N	0.84808800	4.35393300	2.41961700
N	0.22241400	2.00932500	3.84847000
N	-1.36596400	-2.28756000	4.22993100
N	-1.95228400	-4.21373600	2.25239100
C	-0.19818500	-1.14864900	1.12314000
C	-1.34723900	-0.71508400	1.22909600
H	-2.23234400	-0.23901800	1.60066000
C	1.19899300	-1.53903100	1.03581700
C	1.94753400	-0.96145300	-0.20206200
H	1.29388000	-2.62614200	1.05146300
H	1.69508600	-1.16851800	1.93756800
C	3.35612500	-0.56303400	0.27056900
O	3.86628900	-1.04964800	1.25999100
O	3.88251300	0.29025300	-0.57476600
H	4.90611900	0.42442700	-0.46274300
C	2.06423800	-1.95383700	-1.39972300
H	1.08052200	-1.98552400	-1.87208700
H	2.72837100	-1.48239400	-2.12826700
C	1.20377600	0.27126900	-0.71294100
O	0.67032300	0.35480900	-1.80323600
O	1.10707200	1.18945900	0.25050900
C	0.16980100	2.26106000	0.05208300
H	-0.55038400	2.21017000	0.86887500
H	0.71551200	3.19732100	0.09231600
H	-0.33480300	2.14731200	-0.90483800
S	7.27732800	0.54001300	0.46932100
O	8.31196500	-0.49597300	0.37220600
O	6.60910000	0.73612000	1.76639100
O	6.32672400	0.54498200	-0.70914400
C	8.15771800	2.14780100	0.18684300
F	9.09229400	2.35103300	1.12079800
F	8.73668900	2.18295000	-1.01907900
F	7.27298800	3.17312000	0.25326800
C	2.48859100	-3.36752500	-1.09137600
C	3.86046200	-3.73859700	-0.89875800
C	1.50614500	-4.33874100	-1.00570300
C	4.94066400	-2.82326200	-1.02081800
C	4.16398300	-5.10035000	-0.55658900
C	1.81333900	-5.68998700	-0.72631800
H	0.46772300	-4.04684900	-1.14672000
C	6.23553300	-3.19963100	-0.74361100
H	4.75891800	-1.80101500	-1.32095100
C	5.51133200	-5.46471900	-0.29650000
C	3.11664900	-6.05833800	-0.49101300
H	1.01691700	-6.42794100	-0.68642400
C	6.52158300	-4.53318500	-0.36930900
H	7.02947700	-2.46045500	-0.78720500
H	5.72744800	-6.49637700	-0.03153700
H	3.36740300	-7.08980800	-0.25806000
H	7.54504800	-4.81882700	-0.14710100

(TfO•1c)⊂AuCav-8

C	-2.91206900	4.94740100	1.79874500
C	-2.92309700	3.60501700	2.52077000
C	-2.27975300	4.79669200	0.41926200
H	-2.25274500	5.60099200	2.37020300
C	-4.02080000	2.74264000	2.52671900
C	-1.78091400	3.20820300	3.23142400
C	-3.01458500	4.27293600	-0.64673500
C	-0.95140400	5.17018500	0.16316200
C	-4.01831600	1.53479600	3.23108200
H	-4.91863700	3.03699000	1.99605200
C	-1.71810900	2.00304900	3.92664300
O	-0.77472900	4.15158000	3.33414400
C	-2.49691300	4.13667300	-1.93507900
H	-4.03637900	3.95870400	-0.46271200
C	-0.39356300	5.04758300	-1.11063300
O	-0.18307700	5.72057200	1.18874800

C	-5.22275100	0.59935000	3.27208500
C	-2.84809900	1.18967100	3.91937400
H	-0.83260900	1.72909400	4.48494600
C	0.46052500	3.99822000	2.76751900
C	-3.33527700	3.59230300	-3.08069200
C	-1.16913100	4.52366500	-2.14286100
H	0.62830400	5.35743400	-1.29110800
C	0.80212300	4.90449000	1.70981500
H	-5.15920800	0.04808300	4.21029200
C	-5.10083200	-0.42938900	2.15462200
O	-2.91323500	0.05025600	4.72255800
C	-3.43211900	2.07652600	-3.00360900
H	-2.78838100	3.81652300	-3.99688100
O	-0.63733700	4.40252400	-3.43018200
C	-5.47123100	-0.12098800	0.84243200
C	-4.57966400	-1.70865300	2.39177600
C	-2.18428300	-1.06453200	4.44755900
C	2.57809500	3.17147700	2.75314800
C	-4.49162000	1.43221700	-2.36194600
C	-2.45902700	1.27677400	-3.62533500
C	0.28947600	3.39486600	-3.57424400
C	2.94773900	4.14373200	1.77015500
C	-5.34893600	-1.01709300	-0.22202500
H	-5.87093700	0.86580800	0.64217300
C	-4.41729000	-2.63039200	1.35753400
O	-4.24429500	-2.08184200	3.69181300
C	-2.90168000	-2.21475500	3.96627400
C	-4.65507900	0.04515400	-2.37406700
H	-5.23563300	2.03749500	-1.85941900
C	-2.60163100	-0.10986400	-3.69436800
O	-1.44705600	1.91141100	-4.32535800
C	-0.15263200	2.06331100	-3.88159900
C	-5.79249100	-0.65261600	-1.63786000
C	-4.78873300	-2.26699000	0.06873600
H	-4.00422000	-3.60988200	1.56625300
C	-0.26595400	-2.28871700	4.48886700
C	-3.70834000	-0.69762200	-3.08510100
H	-1.88638600	-0.70536600	-4.24773100
C	2.44686800	2.66067200	-3.48493400
H	-5.98153100	-1.59227400	-2.16169500
O	-4.63056700	-3.21656700	-0.95510700
C	-0.99009600	-3.44656800	4.06788900
O	-3.91199000	-2.08490100	-3.22616300
C	2.00130300	1.31210700	-3.66454200
C	4.27915700	4.18114200	1.29632800
C	3.54273900	2.24972400	3.21645400
C	-0.30122000	-4.66646800	3.87104700
C	1.13171500	-2.37501900	4.68441300
C	2.94731300	0.26384600	-3.60919000
C	3.82087200	2.92815400	-3.29735600
C	-7.10758000	0.13943400	-1.66033900
H	-7.40849200	0.33378600	-2.69315100
H	-7.03703700	1.10326600	-1.15017800
C	-6.57045100	1.33404700	3.25534700
H	-7.38975100	0.61636700	3.35225700
H	-6.73961700	1.90269400	2.33665800
C	-4.29143400	5.61872000	1.73558000
H	-4.69937300	5.73945600	2.74317100
H	-5.01903900	5.05040200	1.14869800
C	-4.70483600	4.28185000	-3.17605900
H	-4.56432700	5.35410300	-3.33471300
H	-5.30684300	4.16478800	-2.27043100
H	-5.27907300	3.87861700	-4.01521900
H	-4.20322600	6.60656800	1.27571400
H	-6.62310600	2.03662000	4.09124600
H	-7.89710800	-0.43477100	-1.16879900
C	4.71977700	1.88575600	-3.24156200
C	4.27662200	0.54904600	-3.37952400
H	4.98578600	-0.26139900	-3.26229600
H	5.76353400	2.07413900	-3.02648100
H	2.62454400	-0.76259500	-3.71732200
H	4.12497700	3.95979000	-3.15818100
C	4.82877900	2.29875700	2.72592200
H	3.23263300	1.51084600	3.94746300
C	5.20031300	3.27031100	1.76556300
H	4.53327200	4.91516700	0.53939600
C	1.78170100	-3.57151600	4.46992400
H	1.66104800	-1.47764700	4.98490800
C	1.06359100	-4.72248200	4.06263100
H	-0.87332300	-5.53146700	3.55222700
H	1.60425700	-5.64492900	3.88036800
H	2.85737500	-3.63586100	4.59463200
H	6.20562800	3.25552000	1.36315900
H	5.56295700	1.56622900	3.03592900
P	-3.40328200	-3.07332200	-2.02680000
N	-3.54657200	-4.55744400	-2.79055900
C	-3.50272200	-5.74341000	-1.92546300
C	-2.76014900	-4.70723000	-4.02329700
H	-4.15313300	-5.60085400	-1.06328300
H	-2.48246700	-5.97141900	-1.58041200
H	-3.87161700	-6.59820600	-2.49834500
H	-2.88856500	-3.82920500	-4.65569000
H	-3.13709300	-5.57919800	-4.56401200
H	-1.68891200	-4.85353500	-3.82031300
Au	-1.36124500	-2.30785300	-1.37699800
N	0.67153500	1.05232800	-3.88960600
N	1.54576800	3.68263500	-3.41399700
N	2.01178700	4.99313900	1.24444000
N	1.30747100	3.14311700	3.25742500
N	-0.90662500	-1.09808800	4.68771600
N	-2.32889700	-3.36866800	3.79095700
C	1.18235400	-2.64622400	-1.71226200
C	0.61553800	-1.71345500	-2.27964600
H	0.49394700	-0.84039900	-2.90551400
C	1.78251500	-3.79545300	-1.03794700
C	2.97071900	-3.43479800	-0.10833200
H	2.14105400	-4.48801700	-1.80459900
H	1.01071000	-4.30943900	-0.45608400
C	4.02296700	-2.68967500	-0.95368300
O	3.79033500	-2.31693700	-2.09129600
O	5.14046400	-2.52588800	-0.28622400
H	5.76524200	-1.85377300	-0.74007600
C	2.56805700	-2.62642100	1.16423800
H	3.46178000	-2.11126600	1.52133100
H	2.31365000	-3.36383900	1.92373000
C	3.59404900	-4.76417500	0.35745900
O	3.56200900	-5.20724000	1.48432700
O	4.15899000	-5.40549000	-0.68055300
C	4.82700600	-6.63904200	-0.36143900
H	5.22775100	-7.00647300	-1.30496200
H	5.63125400	-6.45477900	0.35376100
H	4.12252200	-7.35544400	0.06767100
S	6.65086000	0.42479000	-0.19720200
O	5.90878200	0.07642800	1.03055900
O	6.63335400	-0.67337900	-1.22714400
O	6.42415000	1.77307100	-0.75065400
C	8.41755900	0.47551200	0.35554200
F	8.79116000	-0.70519600	0.86666200
F	9.23156800	0.77828100	-0.66290800
F	8.55688700	1.41449700	1.30832800
C	1.39748600	-1.68748900	1.01197200
C	1.54460600	-0.34296000	0.54989200
C	0.13393000	-2.16683900	1.30584200
C	2.81023600	0.21938200	0.24704300
C	0.37019800	0.46390000	0.35424500
C	-1.02502900	-1.36546800	1.14205500
H	0.02595400	-3.18013300	1.67383700
C	2.91265600	1.49878100	-0.24424800
H	3.72071700	-0.33736400	0.42370000
C	0.50908000	1.78346600	-0.14665300
C	-0.90643000	-0.07074900	0.66139700
H	-1.99258100	-1.74861800	1.43611600
C	1.75419200	2.28434500	-0.44521000
H	3.89135400	1.90632100	-0.46257900
H	-0.38244000	2.38726700	-0.28462000
H	-1.78693400	0.55569100	0.54568300
H	1.85659000	3.29387300	-0.81768500

(TfO•1c)⊂AuCav-9

C	-3.70758700	-1.83111800	4.24788000

C	-3.35770500	-2.79790100	3.12143100
C	-3.28958500	-0.40418400	3.90470300
H	-3.09776300	-2.12762600	5.10216300
C	-4.17261100	-2.95004600	1.99460900
C	-2.18970800	-3.56903900	3.17127800
C	-4.16278200	0.52084700	3.32601100
C	-1.99406400	0.03989100	4.20503200
C	-3.87319300	-3.83059500	0.95073000
H	-5.06983100	-2.34464800	1.92177200
C	-1.85197600	-4.46142200	2.16146100
O	-1.33530600	-3.45386400	4.26921600
C	-3.80433900	1.85099400	3.08559100
H	-5.17236600	0.20446400	3.09138000
C	-1.58206700	1.34686500	3.96979700
O	-1.15857500	-0.84208800	4.88297400
C	-4.74055300	-3.94051000	-0.29993100
C	-2.69628200	-4.57641200	1.06636400
H	-0.93908500	-5.03912300	2.22190300
C	-0.15272700	-2.81047700	3.99373300
C	-4.77328500	2.86139100	2.47701700
C	-2.50049000	2.24031300	3.42691300
H	-0.58400800	1.67145200	4.23390100
C	-0.08038600	-1.40100700	4.26049400
H	-4.46078400	-4.87147200	-0.79430700
C	-4.36837600	-2.81037800	-1.25129500
O	-2.36715900	-5.47520700	0.04553100
C	-4.58948400	2.88566600	0.96376100
H	-4.46813600	3.84236400	2.84254800
O	-2.18149100	3.59036100	3.33119300
C	-5.03469700	-1.58225500	-1.24537400
C	-3.30619200	-2.95788900	-2.15417000
C	-1.25540200	-5.15188800	-0.67929500
C	1.99290200	-2.77594400	3.23981800
C	-5.16115500	1.91779900	0.13128600
C	-3.76661500	3.84652000	0.36084400
C	-1.27184400	4.02404600	2.40362000
C	2.05483300	-1.36552500	3.47292700
C	-4.68319600	-0.51332300	-2.07664400
H	-5.88505100	-1.46277900	-0.58574000
C	-2.88760300	-1.90805200	-2.96079100
O	-2.73466000	-4.21811800	-2.31039400
C	-1.44521700	-4.46262200	-1.92681400
C	-4.91440800	1.85779700	-1.24420700
H	-5.79465900	1.15916700	0.57814600
C	-3.50021500	3.82966600	-1.00389200
O	-3.16497900	4.81885300	1.14655000
C	-1.79391700	4.69964700	1.25478600
C	-5.50103800	0.77286400	-2.13762700
C	-3.56443100	-0.69622800	-2.89761100
H	-2.07130500	-2.04595200	-3.65346100
C	0.99222100	-5.16854400	-1.03349900
C	-4.05809000	2.82429400	-1.77877900
H	-2.82243400	4.55686900	-1.43205600
C	0.84463700	4.43704000	1.67838200
H	-5.41348300	1.13888700	-3.16416400
O	-3.14633200	0.30015600	-3.78606600
C	0.80573900	-4.50369500	-2.28387100
O	-3.63476400	2.72644600	-3.11454100
C	0.31986900	5.09337000	0.52010900
C	3.24812800	-0.66354000	3.19217200
C	3.12968100	-3.45181900	2.74066000
C	1.93614500	-4.20707300	-3.08172100
C	2.29578700	-5.51794400	-0.61064400
C	1.20295400	5.66190800	-0.42471800
C	2.24322200	4.39222500	1.87417600
C	-6.99641300	0.54912900	-1.86705700
H	-7.54397100	1.48099500	-2.03000200
H	-7.19873200	0.22788300	-0.84199000
C	-6.23940700	-4.01167000	0.01993100
H	-6.82236800	-4.09574300	-0.90171300
H	-6.60296000	-3.13607800	0.56518000
C	-5.18030500	-1.94288300	4.66921200
H	-5.39891400	-2.96503600	4.98988100
H	-5.87628000	-1.69925700	3.86118600
C	-6.23423900	2.63946000	2.89141900

H	-6.32231600	2.64616800	3.98119500
H	-6.64229100	1.68983600	2.53317200
H	-6.86244500	3.43792500	2.48700900
H	-5.38832500	-1.26439800	5.50118800
H	-6.44343400	-4.88756600	0.64122000
H	-7.39561500	-0.21421300	-2.54038700
C	3.08207100	4.99400100	0.96029600
C	2.56133800	5.61609000	-0.19910600
H	3.24560800	6.06804800	-0.91040500
H	4.15290000	4.98313000	1.12036000
H	0.77721600	6.15005500	-1.29517200
H	2.62536700	3.88571500	2.75354400
C	4.29137900	-2.74716000	2.50179800
H	3.05586800	-4.52127100	2.57553100
C	4.34829000	-1.35052600	2.72293300
H	3.27962200	0.40637800	3.34862000
C	3.37696400	-5.21928400	-1.40995800
H	2.40179700	-6.02505500	0.34197200
C	3.19708900	-4.55535100	-2.64731300
H	1.78442000	-3.70846500	-4.03101400
H	4.06661300	-4.31187500	-3.24821000
H	4.37951300	-5.48432300	-1.09413300
H	5.26083000	-0.80970700	2.51095000
H	5.17271000	-3.25965200	2.13106100
P	-2.37322300	1.67284000	-3.32748700
N	-1.67415500	2.08085900	-4.76850300
C	-2.47317500	2.11247400	-5.99764100
C	-0.50178900	2.96381600	-4.76760000
H	-3.27386000	1.37524100	-5.94142700
H	-1.82713700	1.85948000	-6.84377600
H	-2.90693500	3.10589400	-6.16759600
H	0.10079700	2.78992500	-3.87480000
H	-0.78921300	4.02194500	-4.81763800
H	0.11173000	2.72209000	-5.64018100
Au	-0.98570300	1.60203600	-1.56110000
N	-1.03016600	5.20189500	0.33189100
N	0.00466500	3.89226900	2.61000500
N	0.97691800	-0.69616500	3.99190700
N	0.84468000	-3.47763700	3.49679200
N	-0.07765000	-5.48523100	-0.24319800
N	-0.45118400	-4.15497300	-2.70822300
C	-0.77186900	-0.91265000	0.16133900
C	-1.91126800	-0.58076000	0.39763300
H	-2.90809100	-0.28222200	0.62638000
C	0.60029500	-1.36272000	-0.04702400
C	1.58656100	-0.40191900	-0.80112200
H	0.58825100	-2.31314000	-0.58582600
H	1.04843700	-1.55909900	0.93208500
C	1.59687600	0.96063900	-0.05093900
O	0.61393000	1.76784000	-0.19386700
O	2.53418100	1.17011900	0.74311000
H	3.78780600	1.89321800	0.63298100
C	2.95811000	-1.12800500	-0.71474200
H	3.17282800	-1.22180100	0.35310900
H	2.78752100	-2.14184100	-1.09069600
C	1.22569400	-0.22325500	-2.27847700
O	1.48856300	0.75628300	-2.95204300
O	0.64590600	-1.31618400	-2.79905100
C	0.39643300	-1.26351900	-4.21621800
H	-0.29457500	-0.45086900	-4.45021500
H	1.33257500	-1.10148200	-4.75523700
H	-0.03533700	-2.23241600	-4.45822800
S	5.63595100	2.53169100	1.67255300
O	5.07707300	1.89292300	2.86187400
O	6.18801200	3.87825700	1.73347000
O	4.66614200	2.41581000	0.44405900
C	7.02547300	1.44837900	1.08312500
F	7.97096300	1.41267400	2.02483000
F	6.57786400	0.20559600	0.86774500
F	7.54070300	1.93263800	-0.04491000
C	4.19383000	-0.57314200	-1.39558000
C	5.36640500	-1.40425200	-1.40149300
C	4.27317900	0.68097500	-1.96138000
C	5.40702600	-2.67599900	-0.76969900
C	6.56400600	-0.93358600	-2.03310900

C	5.45944700	1.14581600	-2.57669200
H	3.41775500	1.33960700	-1.95308500
C	6.55177900	-3.44387300	-0.77158800
H	4.52828300	-3.04498000	-0.26000800
C	7.72283800	-1.75292900	-2.02835300
C	6.57839900	0.35543800	-2.62614200
H	5.47211700	2.14470400	-3.00099600
C	7.72377800	-2.98369200	-1.41279600
H	6.55719800	-4.40722500	-0.26840400
H	8.62025700	-1.37996100	-2.51477400
H	7.49278100	0.70852100	-3.09445500
H	8.62057400	-3.59567300	-1.40993100

(TfO•1c)⊂AuCav-10

C	-1.70064400	-2.18138500	4.86774000
C	-1.08660700	-2.93606200	3.69274800
C	-2.15355600	-0.79897300	4.41142600
H	-0.89490400	-2.02003400	5.58380300
C	-1.86161700	-3.68939500	2.80869300
C	0.29514000	-2.87890700	3.45286900
C	-3.43379800	-0.56564600	3.90163900
C	-1.27536100	0.29217000	4.48779100
C	-1.31525200	-4.40871000	1.74294900
H	-2.93385000	-3.72674900	2.96467500
C	0.88413300	-3.56038300	2.38789800
O	1.08257600	-2.23099600	4.39301800
C	-3.86233400	0.69746200	3.48754700
H	-4.13101200	-1.39405600	3.85670800
C	-1.64078900	1.56176500	4.04198200
O	-0.07204100	0.10620000	5.14518000
C	-2.19456800	-5.22004700	0.79306900
C	0.07452000	-4.33529800	1.55682200
H	1.95690800	-3.52731400	2.24049100
C	1.73498200	-1.06326600	4.07575800
C	-5.28571900	0.96886500	3.00571500
C	-2.92737400	1.74014500	3.54849700
H	-0.94452700	2.38738400	4.11094900
C	1.12574700	0.17404300	4.47315900
H	-1.54449400	-5.95619300	0.31986300
C	-2.68925300	-4.29850000	-0.31649000
O	0.65283600	-5.17265700	0.60974500
C	-5.36005100	0.82151000	1.49139400
H	-5.49359300	2.01639300	3.22516900
O	-3.39195700	3.01838600	3.21243000
C	-3.88582000	-3.58559300	-0.23327700
C	-1.90236400	-4.11128200	-1.46466900
C	1.25983000	-4.66474900	-0.51396600
C	3.54304600	0.07854300	3.30610700
C	-5.48311200	-0.43112100	0.88216400
C	-5.26846900	1.93734900	0.64878300
C	-2.98772800	3.64637300	2.08170000
C	2.93226100	1.31120800	3.69891200
C	-4.31405500	-2.69636300	-1.22615000
H	-4.51995900	-3.73935600	0.63095800
C	-2.25451100	-3.19129500	-2.45111200
O	-0.83392600	-4.95403300	-1.64152700
C	0.46816400	-4.50354600	-1.69366500
C	-5.50440000	-0.60811000	-0.50381300
H	-5.55242200	-1.30737800	1.51591500
C	-5.25475400	1.80983400	-0.73876600
O	-5.19470800	3.21291800	1.20660100
C	-3.98128800	3.83581700	1.05283000
C	-5.65716700	-1.98107800	-1.14759000
C	-3.44838600	-2.49520500	-2.30734200
H	-1.62627700	-3.07007400	-3.32537200
C	3.10871500	-4.05227600	-1.69404500
C	-5.36042800	0.53971600	-1.28736200
H	-5.16338500	2.68880400	-1.36457400
C	-1.50430400	4.84265500	0.83620400
H	-5.98450500	-1.81267200	-2.17648900
O	-3.84402100	-1.66545800	-3.37929500
C	2.29976200	-3.83486800	-2.85773400
O	-5.30791300	0.41559800	-2.69192700
C	-2.51625100	5.10289700	-0.13725700
C	3.61071300	2.52611000	3.45129500
C	4.80921700	0.08950500	2.68076800
C	2.90635800	-3.38479900	-4.05018400
C	4.49998300	-3.83072500	-1.75955100
C	-2.20685900	5.88696500	-1.27382600
C	-0.20012000	5.34185000	0.62788700
C	-6.75120800	-2.82023300	-0.47040800
H	-7.70777400	-2.29410000	-0.52390300
H	-6.54508500	-3.01865700	0.58409900
C	-3.32155400	-5.97816300	1.50543800
H	-3.89347500	-6.57044500	0.78532800
H	-4.02432900	-5.32006800	2.02485200
C	-2.80354400	-2.97656900	5.57967500
H	-2.40416400	-3.92966900	5.93647800
H	-3.65838500	-3.20224300	4.93519300
C	-6.34345800	0.12468000	3.73112100
H	-6.27493500	0.28741800	4.80974500
H	-6.23232900	-0.94854100	3.55332800
H	-7.34504000	0.41120700	3.39861300
H	-3.17878900	-2.41324300	6.43864000
H	-2.89795800	-6.65438600	2.25222700
H	-6.85278900	-3.78376800	-0.97625800
C	0.08975300	6.07965300	-0.50161200
C	-0.92118000	6.36443000	-1.44952800
H	-0.68037500	6.95807800	-2.32557000
H	1.10169400	6.43646400	-0.65889500
H	-2.99738600	6.08628200	-1.98986100
H	0.55308200	5.12207400	1.37246900
C	5.43615900	1.29130300	2.42526700
H	5.26964300	-0.84405200	2.38749200
C	4.83599400	2.51187600	2.81429500
H	3.13495500	3.45052700	3.76124500
C	5.06370600	-3.37474200	-2.93033600
H	5.10716700	-3.98042700	-0.87757100
C	4.26552400	-3.15380800	-4.07702500
H	2.27697200	-3.23025600	-4.92040500
H	4.73288300	-2.79664300	-4.99005900
H	6.12708800	-3.16540600	-2.94717600
H	5.33869800	3.44888800	2.59757200
H	6.39072200	1.28372900	1.91169900
P	-3.88713400	-0.05132600	-3.33908900
N	-4.16679000	0.26834800	-4.95170300
C	-3.25562100	-0.35241500	-5.92493000
C	-4.52163500	1.65615200	-5.28074200
H	-3.09158100	-1.39831500	-5.66965400
H	-2.28852600	0.16846600	-5.97875500
H	-3.72923300	-0.30773900	-6.90884900
H	-5.24923000	2.03653600	-4.56407100
H	-4.98096100	1.66434200	-6.27240400
H	-3.64263500	2.31719300	-5.29639400
Au	-2.13692300	1.06172400	-2.34867000
N	-3.76171700	4.54579500	-0.01207600
N	-1.78001300	4.11250500	1.96094100
N	1.70123300	1.32372000	4.29961400
N	2.90455000	-1.11332000	3.52020800
N	2.54226400	-4.45298000	-0.51605100
N	0.95539900	-4.08367400	-2.82371200
C	0.07224000	1.93937800	-1.88625300
C	-0.80476100	2.80094700	-1.83221100
H	-1.29723400	3.75190500	-1.76547700
C	1.14573600	0.96288900	-1.91678500
C	1.64091100	0.55059900	-0.51228000
H	1.98785000	1.40026000	-2.46402900
H	0.84519000	0.07471700	-2.47628500
C	2.50730100	-0.71288600	-0.71583900
O	2.75979300	-1.12737200	-1.82913400
O	2.93739700	-1.20123500	0.42950500
H	3.85171300	-1.60681100	0.29294200
C	2.45683600	1.66836200	0.19144500
H	1.72443900	2.40004700	0.53357600
H	2.87860300	1.22555400	1.09258600
C	0.48161300	0.18272300	0.41793800
O	0.41796900	0.46265600	1.59174400
O	-0.48953900	-0.50204200	-0.22927000
C	-1.60840400	-0.88013200	0.60830900
H	-2.09241600	0.01100700	1.01389900

H	-2.28260600	-1.42882100	-0.04092700
H	-1.26475500	-1.50484400	1.42964300
S	6.82982000	-1.71374800	0.05808000
O	7.35655000	-1.38312800	1.39244800
O	5.36568900	-1.44513700	-0.10596300
O	7.28464600	-2.97287600	-0.55629500
C	7.52478200	-0.39385700	-1.04440600
F	7.28539900	0.82889800	-0.53421700
F	8.84695900	-0.53332600	-1.19402700
F	6.95576200	-0.44681800	-2.26921700
C	3.54440200	2.36798300	-0.59960700
C	3.77407300	3.76995400	-0.37602900
C	4.36392500	1.70092000	-1.49255100
C	3.11307000	4.51264700	0.64046700
C	4.75512800	4.45778800	-1.16595100
C	5.34696300	2.37999700	-2.25041500
H	4.28742500	0.63097200	-1.62967100
C	3.36587600	5.85286000	0.83858600
H	2.43775500	4.00234700	1.31341400
C	4.97088200	5.84628200	-0.95939400
C	5.51950100	3.73372000	-2.11683800
H	5.97002600	1.80421100	-2.92464600
C	4.29087800	6.53720500	0.01668100
H	2.86165800	6.38442100	1.64089500
H	5.70798600	6.35031400	-1.57867500
H	6.26450000	4.26305300	-2.70428700
H	4.47927500	7.59477800	0.17449500

(TfO•2c)⊂AuCav-1

C	5.91402300	0.73664800	2.04112400
C	5.29270400	1.72043000	1.05467200
C	5.01684700	-0.46758900	2.31426600
H	5.99155300	1.27168600	2.98845500
C	5.43372200	1.58068200	-0.32966900
C	4.57321100	2.83387800	1.50943600
C	5.03555600	-1.62220200	1.52264600
C	4.15521500	-0.46875900	3.41941200
C	4.91827400	2.50208500	-1.24728700
H	5.96733200	0.71683400	-0.70928800
C	4.05768600	3.78673800	0.64156200
O	4.38462500	3.00272900	2.88607100
C	4.26597800	-2.75534300	1.81149800
H	5.67934000	-1.64116300	0.65051300
C	3.39281200	-1.57548200	3.76430700
O	4.08384100	0.67763400	4.21816000
C	5.03606800	2.29495000	-2.75532200
C	4.24200900	3.61180700	-0.72467200
H	3.50929300	4.63930200	1.01857500
C	3.12891200	2.69062100	3.33342500
C	4.25469400	-3.99889600	0.92494400
C	3.46600400	-2.70612800	2.95960100
H	2.74254100	-1.55463300	4.62750600
C	2.96705300	1.44636700	4.03480500
H	4.88002600	3.27092600	-3.21628300
C	3.89154900	1.40248900	-3.22685400
O	3.75820900	4.58591700	-1.60034200
C	3.23429200	-3.83069800	-0.19864100
H	3.89419800	-4.81777300	1.54799100
O	2.72844100	-3.83738600	3.30633600
C	3.98941900	0.00742900	-3.26750900
C	2.66596600	1.97071800	-3.60233700
C	2.39477700	4.68326700	-1.69108500
C	0.90024400	3.10337100	3.55526500
C	3.56798100	-3.21245800	-1.40587700
C	1.91019500	-4.27311300	-0.04627200
C	1.37075900	-3.70602900	3.17297700
C	0.73844300	1.86276200	4.24533800
C	2.92725700	-0.81915000	-3.65015400
H	4.93327400	-0.45271100	-3.00006100
C	1.57464000	1.19007900	-3.96091300
O	2.57153300	3.35721700	-3.67136800
C	1.75681800	4.00900800	-2.78571700
C	2.65183000	-3.01934800	-2.44334400
H	4.58393200	-2.86232400	-1.54397700
C	0.95248000	-4.08855500	-1.04249100
O	1.56930000	-4.94469700	1.12503000
C	0.75444200	-4.29099900	2.01760900
C	3.04765600	-2.33806300	-3.74951800
C	1.71644000	-0.19267300	-3.96928500
H	0.63805500	1.65462500	-4.24161600
C	0.37231900	5.45128200	-0.98794400
C	1.34014900	-3.46084800	-2.22494100
H	-0.07260800	-4.40700400	-0.88854600
C	-0.65884400	-2.95295500	3.87589800
H	2.31335500	-2.65234600	-4.49537400
O	0.60982900	-0.94140400	-4.35500200
C	-0.26277000	4.78273300	-2.07931300
O	0.35701100	-3.23764500	-3.20438500
C	-1.27956800	-3.56738200	2.74433000
C	-0.54596800	1.47695800	4.69109400
C	-0.22657500	3.92352600	3.32203600
C	-1.66027600	4.89878000	-2.24508700
C	-0.40936100	6.20920200	-0.08660400
C	-2.68023500	-3.47083600	2.57416500
C	-1.45103400	-2.22972000	4.79866800
C	4.42457400	-2.79299100	-4.25575200
H	4.43713400	-3.87910700	-4.37874200
H	5.23795500	-2.53040300	-3.57419800
C	6.41960200	1.79037700	-3.18635200
H	6.46289200	1.69276100	-4.27468300
H	6.67451900	0.81700400	-2.75777000
C	7.33537700	0.32024500	1.63016600
H	7.96678200	1.20545000	1.51518000
H	7.36260300	-0.22831700	0.68452500
C	5.65156600	-4.38065200	0.41489300
H	6.32236300	-4.55457300	1.26054900
H	6.10685700	-3.60699800	-0.20996500
H	5.59800300	-5.29611200	-0.18064100
H	7.77355800	-0.32433000	2.39698700
H	7.19029300	2.49825100	-2.86985800
H	4.64110800	-2.32891000	-5.22165900
C	-2.81026300	-2.11927900	4.59158400
C	-3.42388200	-2.74467900	3.47959800
H	-4.49240700	-2.63735500	3.33167000
H	-3.42014900	-1.54460700	5.28046400
H	-3.11531700	-3.97005400	1.71735200
H	-0.95669300	-1.78640200	5.65670300
C	-1.46843300	3.52870800	3.77506600
H	-0.08636700	4.84088400	2.76200800
C	-1.62859200	2.30281300	4.46392900
H	-0.64747100	0.52706300	5.20270600
C	-1.77205300	6.30224100	-0.27142700
H	0.09868300	6.71081900	0.73023100
C	-2.40030200	5.64508100	-1.35437400
H	-2.11438300	4.39145300	-3.08868000
H	-3.47635600	5.71341500	-1.47218200
H	-2.37400000	6.88139100	0.42149900
H	-2.61245000	1.99907100	4.80429600
H	-2.33655200	4.14625000	3.57368600
P	-0.30852400	-1.73414000	-3.21929200
N	-1.74400300	-1.94726100	-3.94641800
C	-1.98425300	-2.96802000	-4.96993500
C	-2.83640000	-1.00609700	-3.70630900
H	-1.14208800	-3.65769000	-5.01508300
H	-2.11729100	-2.49523200	-5.95038900
H	-2.87467100	-3.52960100	-4.68394500
H	-2.57203000	-0.31756400	-2.90295400
H	-3.72354900	-1.56319000	-3.40184100
H	-3.04320500	-0.42067100	-4.61049700
Au	-0.01327500	-0.87626400	-1.08503800
N	-0.52539300	-4.22893700	1.81271900
N	0.69152100	-3.04983300	4.06738200
N	1.81030000	1.04102700	4.46837700
N	2.13107000	3.49010300	3.09866900
N	1.72762500	5.37573600	-0.81644500
N	0.47031100	4.04696800	-2.97285900
C	-0.43726400	0.32999300	1.16603500
C	0.72989300	-0.37725800	0.98348500
H	0.79418300	-1.36582700	1.42631000
C	-1.75804200	-0.14558600	1.69167600

C	-2.73207900	0.95087000	1.22702200
H	-1.70796600	-0.19430400	2.78311600
H	-2.01819000	-1.13806900	1.32104800
C	-1.82005300	2.12757100	0.89785700
O	-0.48229900	1.65164900	0.88391200
O	-2.04412500	3.26051300	0.61625600
C	-3.43185400	0.46229300	-0.09858300
H	-2.62514900	0.20841200	-0.79091500
H	-3.89643800	-0.48681500	0.14955400
C	-3.78107600	1.26770100	2.28595700
O	-4.13329600	0.46783200	3.12830600
O	-4.29104700	2.49256700	2.14420200
C	-5.39702300	2.81504100	3.00762700
H	-5.65338300	3.84633200	2.77124800
H	-6.23608300	2.15096600	2.79278900
H	-5.10592100	2.71532200	4.05564000
H	1.65857700	0.16126200	0.81902500
C	-4.39308000	1.40740300	-0.76141600
C	-5.78330400	1.42307500	-0.41372800
C	-3.92807600	2.26844700	-1.73499700
C	-6.35738000	0.53145800	0.53348700
C	-6.64526300	2.37335200	-1.05450000
C	-4.78530700	3.18530100	-2.38315500
H	-2.87549800	2.25064000	-2.00015500
C	-7.69659400	0.59614600	0.85051100
H	-5.74541900	-0.22177200	1.01028400
C	-8.01986400	2.41582400	-0.70105400
C	-6.11397800	3.24634700	-2.03984500
H	-4.38937400	3.83972600	-3.15328100
C	-8.53806600	1.55179100	0.23504200
H	-8.11095900	-0.10224900	1.57120000
H	-8.65858000	3.14436200	-1.19305100
H	-6.78104700	3.95273800	-2.52614300
H	-9.59176200	1.59104600	0.49343400
S	-3.32559500	-3.59190100	-1.09150200
O	-3.16937400	-4.78234700	-0.23860300
O	-2.42397400	-2.45037100	-0.74260900
O	-3.49007300	-3.84201700	-2.53431900
C	-4.99225100	-2.91410200	-0.61427200
F	-5.96151900	-3.81381700	-0.78136200
F	-5.29369800	-1.82334300	-1.35104200
F	-5.00235600	-2.52625300	0.68870600

(TfO•2c)⊂AuCav-2

C	1.68561300	-5.30895800	1.87691000
C	1.82657000	-4.83361600	0.43349000
C	0.99332900	-4.25166900	2.73136000
H	2.69998600	-5.40073100	2.26651300
C	0.84497300	-5.07664500	-0.52835100
C	2.97238600	-4.14165800	0.01365800
C	-0.39644500	-4.16291200	2.83909500
C	1.74823400	-3.31288900	3.44475800
C	0.96677400	-4.69372300	-1.86840200
H	-0.05392500	-5.59958400	-0.22441800
C	3.13783700	-3.72837000	-1.30538200
O	4.01570600	-3.97879900	0.91963100
C	-1.04049100	-3.19299800	3.61562400
H	-1.00555400	-4.87777800	2.29715400
C	1.15820300	-2.31786800	4.21310400
O	3.13863000	-3.43433700	3.41952600
C	-0.12817900	-5.00807700	-2.88559200
C	2.13431400	-4.01001700	-2.23028900
H	4.04871300	-3.23051600	-1.61432900
C	4.30590500	-2.74599400	1.44007500
C	-2.56013500	-3.14434200	3.73552700
C	-0.23001600	-2.26710600	4.28741700
H	1.76391700	-1.58937900	4.73640400
C	3.85276500	-2.46449500	2.77486200
H	0.33626400	-4.93999700	-3.86984900
C	-1.20678300	-3.93027800	-2.83910400
O	2.34786200	-3.67710900	-3.57726700
C	-3.19465200	-2.41740800	2.55324100
H	-2.77489300	-2.53588300	4.61459600
O	-0.82394100	-1.28390500	5.08458500
C	-2.32661400	-3.99901500	-2.00409400
C	-1.07476400	-2.78380700	-3.63049700
C	2.26969000	-2.35506600	-3.91227000
C	5.39024000	-0.74291200	1.39590800
C	-3.57345000	-3.06865500	1.37593800
C	-3.42192700	-1.03473000	2.62717300
C	-0.78434000	-0.02109400	4.53770600
C	4.91840500	-0.45499700	2.71289000
C	-3.29355300	-2.98922400	-1.93877900
H	-2.45218500	-4.87513900	-1.37969500
C	-2.00021400	-1.74860800	-3.59745100
O	-0.00553000	-2.74179600	-4.52105500
C	0.99462100	-1.83771500	-4.33920900
C	-4.19308100	-2.40238200	0.31267800
H	-3.37492700	-4.12988400	1.28086400
C	-4.07217500	-0.33341000	1.61835700
O	-3.02306700	-0.38068300	3.78296700
C	-1.89912800	0.40504700	3.74185200
C	-4.50708400	-3.08627100	-1.01463300
C	-3.09903800	-1.86127300	-2.75204300
H	-1.88035400	-0.88406300	-4.23679800
C	3.18530700	-0.28948700	-4.21001900
C	-4.46199900	-1.04174200	0.48779900
H	-4.25522000	0.73287100	1.68835700
C	0.26916500	1.94504300	4.09613200
H	-5.30399200	-2.50734200	-1.48761700
O	-4.05777400	-0.85785500	-2.82066200
C	1.89919800	0.24300400	-4.53257900
O	-5.13508600	-0.34393700	-0.52945000
C	-0.77932800	2.30828400	3.19553800
C	5.25286900	0.77611100	3.32043100
C	6.22371700	0.19152400	0.73921200
C	1.76471100	1.62118900	-4.81361800
C	4.31185500	0.56301300	-4.23009400
C	-0.69695800	3.52280500	2.47572300
C	1.34937800	2.83211200	4.30235900
C	-5.02870400	-4.51926600	-0.84602900
H	-5.93666200	-4.51350600	-0.23747900
H	-4.31102600	-5.18179600	-0.35484600
C	-0.68952900	-6.42884900	-2.73527500
H	-1.43064600	-6.62405800	-3.51530700
H	-1.17413100	-6.59914200	-1.77001700
C	1.02151600	-6.68905900	1.98686800
H	1.57603100	-7.42357300	1.39619400
H	-0.01395000	-6.69666700	1.63473800
C	-3.17202400	-4.53380100	3.97090200
H	-2.74321700	-4.98204400	4.87116000
H	-2.99192600	-5.22612900	3.14343900
H	-4.25444800	-4.45188900	4.10377600
H	1.01341400	-7.01514600	3.03031300
H	0.11685700	-7.16077900	-2.82993200
H	-5.26516700	-4.95302900	-1.82141100
C	1.38960700	4.02888600	3.62220300
C	0.36963900	4.36804900	2.70006900
H	0.42421400	5.31076800	2.16335200
H	2.22066000	4.70960900	3.77727600
H	-1.48128800	3.75764700	1.76775000
H	2.13487700	2.52877000	4.98496900
C	6.54311000	1.38222700	1.35731200
H	6.59023000	-0.05344400	-0.25136900
C	6.04569000	1.67962300	2.64762900
H	4.85154600	0.97999300	4.30644100
C	4.15785500	1.90247800	-4.52488900
H	5.28020200	0.13241700	-3.99904900
C	2.87806300	2.43514500	-4.80473500
H	0.77617500	2.00179500	-5.04675400
H	2.77774900	3.49388200	-5.02033900
H	5.02030900	2.55932400	-4.51589700
H	6.28998300	2.63302100	3.10449400
H	7.16690900	2.10461400	0.84316500
P	-4.21992700	0.33748900	-1.68575400
N	-5.26249400	1.34536600	-2.44445600
C	-6.55589800	1.77742100	-1.90302800
C	-4.73173900	2.16836900	-3.53599800
H	-6.79380700	1.21068100	-1.00648400
H	-7.33062800	1.60420800	-2.65912500

H	-6.50431900	2.83787100	-1.64716400
H	-3.74616000	1.80640600	-3.84229300
H	-4.64752100	3.20880600	-3.20795200
H	-5.39687800	2.09534100	-4.40312000
Au	-2.25289000	1.07376600	-0.86220300
N	-1.88091700	1.50656100	3.05549800
N	0.25453300	0.73324700	4.73134800
N	4.13994000	-1.35423000	3.38591100
N	5.04290900	-1.91089300	0.76646100
N	3.33240700	-1.60678200	-3.86290000
N	0.80435600	-0.57723700	-4.60090900
C	0.75999200	-1.08851400	0.13484800
C	-0.28409100	-1.90001400	0.06284300
H	-0.60143100	-2.28311900	-0.89496200
C	1.62261600	-0.49698000	-0.93855500
C	1.97061100	0.90506800	-0.34430700
H	1.12388200	-0.45489300	-1.90612800
H	2.55446400	-1.06132000	-1.04671700
C	1.92544800	0.56829600	1.16649800
O	1.19783700	-0.57307700	1.35813100
O	2.45981500	1.14728400	2.06820100
C	3.31961900	1.43542300	-0.83011100
H	4.07368600	0.70684900	-0.51782200
H	3.29870500	1.40088200	-1.92218600
C	0.77823500	1.76656200	-0.70195600
O	-0.28635000	1.64997700	-0.08452900
O	0.96207900	2.53369700	-1.75037800
C	-0.14495400	3.35587500	-2.20791200
H	0.31200700	4.09748200	-2.85973700
H	-0.84411600	2.72924000	-2.76829600
H	-0.66832400	3.81018600	-1.36715200
H	-0.81779400	-2.20905400	0.95074400
C	3.76108600	2.81764300	-0.39706300
C	5.02703200	3.29382000	-0.87355800
C	3.01244300	3.63019200	0.42706000
C	5.87638900	2.51472200	-1.70602600
C	5.48587500	4.59270600	-0.47828100
C	3.45864000	4.91892600	0.79949200
H	2.07485800	3.28778800	0.84466000
C	7.10351500	2.98207400	-2.12149000
H	5.56002300	1.52589900	-2.00695400
C	6.75529700	5.04817300	-0.92447000
C	4.66874100	5.39451300	0.35969500
H	2.83219900	5.52491700	1.44504600
C	7.55167700	4.26471600	-1.72732400
H	7.73538500	2.35968100	-2.74851500
H	7.08851300	6.03459300	-0.61340600
H	5.01948200	6.38129500	0.64849000
H	8.52127100	4.62479900	-2.05707100
S	-4.05695000	3.81823600	-0.08205800
O	-4.68851400	2.56859400	0.41074300
O	-2.56908100	3.72568500	-0.20860300
O	-4.72935600	4.45987200	-1.22993700
C	-4.28359600	5.00781700	1.32421300
F	-5.58464300	5.24689000	1.52619200
F	-3.66757700	6.16831600	1.05771700
F	-3.76198300	4.50031700	2.45181300

(TfO•2c)⊂AuCav-3

C	0.59771900	5.40563300	2.09327600
C	-0.70385800	4.84212700	1.53893800
C	1.77712300	4.47535300	1.83178300
H	0.47483600	5.44127800	3.17591400
C	-1.09212600	5.06122600	0.21235700
C	-1.57480400	4.10614400	2.35400700
C	2.52325100	4.51202700	0.65008900
C	2.17895900	3.56188200	2.81345700
C	-2.30811100	4.61354400	-0.30324800
H	-0.42531400	5.61788800	-0.43509300
C	-2.80277800	3.63789900	1.88721300
O	-1.23784900	3.91254800	3.69502800
C	3.66620700	3.73192800	0.45148400
H	2.21100100	5.18462400	-0.14059200
C	3.32019700	2.77956500	2.67664600
O	1.44067600	3.51353600	3.99782500
C	-2.72453600	4.81718400	-1.75899300
C	-3.14634200	3.90402400	0.56663700
H	-3.47535000	3.10174500	2.54425900
C	-0.80521500	2.66344800	4.06126900
C	4.43069500	3.72359100	-0.87038900
C	4.06480800	2.89768500	1.50594200
H	3.63937500	2.11638300	3.47027200
C	0.61127800	2.44345600	4.17944800
H	-3.81447700	4.81079400	-1.78323700
C	-2.25152800	3.59636100	-2.53869200
O	-4.40345700	3.58074600	0.07149000
C	3.78348400	2.67790400	-1.77452200
H	5.43826400	3.37124900	-0.64941100
O	5.28645000	2.24585200	1.40102400
C	-0.95003900	3.51952400	-3.04042900
C	-3.07043600	2.47196100	-2.71182300
C	-4.86811000	2.31824500	-0.09218300
C	-1.18413500	0.50707600	4.68849300
C	2.71169000	2.98599500	-2.61340500
C	4.20794900	1.34040100	-1.73385000
C	5.31683400	0.87694600	1.36130300
C	0.22354200	0.27170900	4.74436400
C	-0.43625600	2.37983700	-3.66040800
H	-0.31254500	4.39150400	-2.95696400
C	-2.56977700	1.28139500	-3.23488900
O	-4.44375500	2.58406200	-2.45522200
C	-5.01167900	1.85703400	-1.45273900
C	2.05020800	2.01888300	-3.37358200
H	2.38302000	4.01618100	-2.68298000
C	3.54621800	0.32978100	-2.42913400
O	5.37379400	1.06523600	-1.02888700
C	5.35684100	0.24798900	0.06989900
C	0.92806000	2.37097000	-4.33745800
C	-1.24766800	1.23732200	-3.67775800
H	-3.20166000	0.40683900	-3.33128300
C	-6.01295500	0.49546200	0.64458100
C	2.46133100	0.68875400	-3.22662100
H	3.85045900	-0.70544300	-2.33432300
C	5.53454200	-1.15703200	2.35661800
H	0.89363300	1.56521000	-5.07435500
O	-0.77169100	0.04104700	-4.19496500
C	-6.30966300	0.14088700	-0.70722600
O	1.78170400	-0.31463800	-3.93321000
C	5.57290600	-1.78324400	1.07109600
C	0.70595900	-1.01049100	5.09036300
C	-2.07603500	-0.54017100	5.01141800
C	-7.14877700	-0.96649700	-0.96741600
C	-6.49245900	-0.31377200	1.69854900
C	5.72542800	-3.18598800	0.97529000
C	5.65631200	-1.94866600	3.52134900
C	1.20059700	3.67131600	-5.11020200
H	2.15273000	3.59504900	-5.64176300
H	1.25547600	4.54984100	-4.46195100
C	-2.25350800	6.15099400	-2.35079000
H	-2.61134100	6.25019200	-3.37903500
H	-1.16515600	6.25371400	-2.37113600
C	0.86950900	6.84031800	1.61397200
H	0.02729200	7.48876700	1.87082900
H	1.02264200	6.90975100	0.53348300
C	4.54911300	5.10900900	-1.51700800
H	5.05495700	5.79862500	-0.83571600
H	3.58123500	5.55082500	-1.77099800
H	5.13158000	5.04301500	-2.43998400
H	1.76942600	7.22949100	2.09757800
H	-2.65041700	6.98314100	-1.76298700
H	0.40538300	3.84878400	-5.83931000
C	5.82003800	-3.31387100	3.40329100
C	5.85005300	-3.93176100	2.12953700
H	5.95961500	-5.00941800	2.06177700
H	5.91937700	-3.92426600	4.29582300
H	5.69798200	-3.62580000	-0.01447500
H	5.62525800	-1.45116200	4.48472900
C	-1.58095200	-1.77770500	5.36542200
H	-3.14070500	-0.33733000	4.97072700
C	-0.18639600	-2.01575500	5.39502900

H	1.77790900	-1.16966400	5.09684300
C	-7.28452900	-1.40913000	1.41758200
H	-6.23397500	-0.04048800	2.71575500
C	-7.62899900	-1.72454900	0.08217500
H	-7.37018300	-1.20964000	-2.00073800
H	-8.26557000	-2.58007500	-0.11864300
H	-7.64141600	-2.03497000	2.22799100
H	0.18098800	-3.00328600	5.65224600
H	-2.26613700	-2.58219800	5.61350600
P	0.35114900	-0.86963000	-3.34489400
N	0.20117000	-2.34831300	-3.99261600
C	0.73069400	-2.68089100	-5.31961300
C	-0.35035500	-3.46360300	-3.22242400
H	1.12205500	-1.78798200	-5.80734100
H	-0.07291600	-3.09715700	-5.93757500
H	1.53927500	-3.40485800	-5.19717500
H	-0.59014800	-3.14471900	-2.20984800
H	0.39533200	-4.25595800	-3.16824500
H	-1.26443500	-3.83060000	-3.69964600
Au	0.24377800	-0.39248600	-1.10715200
N	5.47903700	-1.03456200	-0.07027000
N	5.39349900	0.20149700	2.46954400
N	1.10802500	1.28094200	4.48264600
N	-1.67348100	1.72832500	4.31184400
N	-5.29675400	1.62961800	0.92461700
N	-5.73612000	0.81870700	-1.75041800
C	-3.46239400	-2.75257100	-1.44630600
C	-3.74141700	-2.50303800	-2.72039500
H	-3.34224000	-3.10478100	-3.52534400
C	-3.93623000	-2.01966400	-0.22297300
C	-3.53341600	-2.91908000	0.95754800
H	-5.00622300	-1.85916000	-0.27116100
H	-3.44297700	-1.04907700	-0.15725100
C	-2.63107600	-3.97623100	0.29014900
O	-2.63868700	-3.80592100	-1.06625000
O	-2.02741100	-4.86434600	0.82594500
H	-4.40059800	-1.67721500	-2.96159700
C	-2.87167000	-2.19015200	2.15074200
H	-2.62449300	-2.93233800	2.90807700
H	-3.64745800	-1.55890000	2.59023100
C	-4.75797900	-3.67765600	1.48795000
O	-5.16855900	-3.64096300	2.62510500
O	-5.32563000	-4.39172400	0.49978500
C	-6.46393400	-5.18630600	0.87522100
H	-6.76596700	-5.70909100	-0.03086300
H	-6.18798500	-5.89525800	1.65842600
H	-7.26995300	-4.54602100	1.23942300
C	-1.67713100	-1.33408100	1.80711900
C	-0.33904500	-1.84133900	1.89919900
C	-1.89334100	-0.02548100	1.39917700
C	-0.03390100	-3.15927800	2.33258000
C	0.76057300	-0.96815700	1.58993800
C	-0.82988400	0.81205900	0.99907300
H	-2.89802700	0.38315200	1.40803000
C	1.27079900	-3.57130200	2.46827900
H	-0.83233900	-3.85953400	2.52895000
C	2.08993300	-1.41225200	1.76942900
C	0.49227700	0.34596600	1.07041800
H	-1.02787100	1.83176000	0.68923700
C	2.34177400	-2.68989800	2.20753200
H	1.48310500	-4.59041700	2.77265100
H	2.89952500	-0.73348800	1.53255300
H	1.32112400	1.04014700	0.94950200
H	3.35869200	-3.03344200	2.32659700
S	3.08271600	-3.59268600	-2.01348300
O	2.60326800	-4.09004400	-3.32072100
O	4.54679000	-3.53949700	-1.85820200
O	2.35161400	-2.40164100	-1.48728100
C	2.54382400	-4.93569800	-0.84136800
F	1.21435900	-4.88260000	-0.63094000
F	3.15509400	-4.81499600	0.35579400
F	2.84141600	-6.14717000	-1.32904500

(TfO•3c)⊂AuCav-1

C	-0.77133700	-5.19437700	2.40254000
C	-0.60221300	-3.84541400	3.09515900
C	-0.60221000	-5.02112800	0.89847300
H	-1.80591100	-5.49710500	2.56526100
C	0.62692500	-3.38695700	3.57399200
C	-1.71670700	-3.01464300	3.28011700
C	0.66019200	-5.04138600	0.30199300
C	-1.70628100	-4.81682100	0.06068300
C	0.76979100	-2.17238100	4.25175500
H	1.50249000	-4.01083800	3.43682700
C	-1.62471100	-1.78850100	3.93313800
O	-2.95410500	-3.53199200	2.92340300
C	0.85613500	-4.86759400	-1.06910000
H	1.52487900	-5.22294400	0.92974900
C	-1.55791500	-4.58380800	-1.30416200
O	-2.98613500	-4.93793800	0.60344500
C	2.11185200	-1.68995600	4.79491000
C	-0.38005800	-1.39162400	4.41832900
H	-2.50574400	-1.18241100	4.10240900
C	-3.70527600	-3.03517700	1.89561800
C	2.23620600	-5.00454800	-1.70314000
C	-0.27810800	-4.59662100	-1.84677000
H	-2.42187900	-4.39161200	-1.92680100
C	-3.77159900	-3.81830100	0.69069500
H	1.88711500	-0.97107400	5.58270000
C	2.85780000	-0.92448600	3.70842300
O	-0.27140600	-0.23353200	5.19018100
C	3.00649300	-3.69250500	-1.64730700
H	2.06630000	-5.21362300	-2.75949000
O	-0.10914300	-4.41451400	-3.22466000
C	3.65371900	-1.57823100	2.76485600
C	2.73409300	0.46793900	3.59272300
C	-0.40618500	0.97689400	4.57121700
C	-5.30355900	-1.64016600	1.07303700
C	3.74920000	-3.29499600	-0.53041400
C	2.98293600	-2.82279700	-2.74363300
C	-0.16347600	-3.13961600	-3.71681800
C	-5.38660800	-2.43368900	-0.11235200
C	4.32142300	-0.90923600	1.73566800
H	3.76820500	-2.65252600	2.84264900
C	3.35639400	1.18054500	2.56892900
O	1.99571200	1.14327600	4.56277200
C	0.78850500	1.69833800	4.22102600
C	4.45338400	-2.08733500	-0.48060400
H	3.77556200	-3.94852100	0.33406300
C	3.67899000	-1.61952200	-2.74656500
O	2.24183300	-3.19212000	-3.86352800
C	1.07669600	-2.49672000	-4.06346800
C	5.23089600	-1.64169800	0.75270700
C	4.13063600	0.47685700	1.65036700
H	3.21462600	2.24834000	2.45675000
C	-1.67261600	2.69631700	3.77974700
C	4.40426400	-1.26647500	-1.61411700
H	3.63532000	-0.96558600	-3.60738700
C	-1.29805800	-1.31534200	-4.47483100
H	5.96502400	-0.90991400	0.40784200
O	4.79461900	1.20331500	0.65518900
C	-0.48747600	3.40679800	3.41737200
O	5.06347600	-0.03761300	-1.61132400
C	-0.06548300	-0.67483200	-4.80589200
C	-6.30229900	-2.07238800	-1.12501100
C	-6.15834000	-0.52728400	1.22768600
C	-0.59513100	4.67394100	2.80278300
C	-2.93724600	3.27370200	3.52645900
C	-0.07678500	0.60557000	-5.40242300
C	-2.51722400	-0.65665000	-4.75580100
C	6.01451000	-2.79059100	1.40417900
H	6.70737000	-3.22524800	0.67884500
H	5.37099700	-3.59676300	1.76610600
C	2.94742200	-2.81678800	5.41850300
H	3.87474300	-2.41153300	5.83279500
H	3.22030300	-3.59734900	4.70275000
C	0.12927100	-6.29045800	2.98846800
H	-0.06426500	-6.40362000	4.05880900
H	1.19624500	-6.08228200	2.86803700
C	3.03812900	-6.17954200	-1.12202600

H	2.47186000	-7.10850800	-1.22924800
H	3.26908200	-6.05643200	-0.06021900
H	3.98714000	-6.28570600	-1.65512600
H	-0.07528500	-7.24438100	2.49489000
H	2.38547300	-3.29499100	6.22527700
H	6.58878700	-2.41864700	2.25681500
C	-2.50264600	0.58696600	-5.35296400
C	-1.27970300	1.21985600	-5.67874900
H	-1.29376400	2.19591400	-6.15383000
H	-3.43569800	1.09293500	-5.57586500
H	0.87457100	1.06945900	-5.63883900
H	-3.44038900	-1.16113900	-4.49217600
C	-7.05417100	-0.20529700	0.23092200
H	-6.08158600	0.05945500	2.13609400
C	-7.11909700	-0.97471600	-0.95320600
H	-6.33276200	-2.67925300	-2.02323600
C	-3.01531500	4.50979100	2.91835400
H	-3.82010900	2.71791600	3.82437800
C	-1.84058400	5.20886800	2.55412100
H	0.31437000	5.18531400	2.52427300
H	-1.92278100	6.17479900	2.06670200
H	-3.98512500	4.94699700	2.70455100
H	-7.81293100	-0.68731600	-1.73599800
H	-7.69771000	0.65999800	0.34432300
P	4.27540300	1.23665100	-0.88780400
N	5.12744200	2.50505600	-1.50068500
C	5.64538700	3.61572100	-0.69828700
C	4.86408800	2.83608100	-2.90628100
H	5.74203300	3.31395500	0.34127300
H	4.97643000	4.47831700	-0.74996000
H	6.63261100	3.88659900	-1.08896200
H	4.53084700	1.94861200	-3.44925300
H	5.79653300	3.17809100	-3.36609600
H	4.09414900	3.61030700	-2.97989500
Au	2.05510500	0.91069400	-1.24108800
N	1.12594300	-1.30326800	-4.57508200
N	-1.31200200	-2.56320000	-3.91437000
N	-4.59161300	-3.53424300	-0.27487500
N	-4.43085500	-1.97103400	2.07614900
N	-1.59568700	1.45825700	4.35678600
N	0.74808200	2.86881800	3.65837000
C	0.46083500	-0.13300900	0.97655300
C	0.46451800	-1.25343300	0.25341200
H	1.27879500	-1.96036000	0.17189000
C	-0.74187100	0.75127800	1.02573500
C	-1.66963300	0.59129600	-0.22430800
H	-0.44712300	1.80031400	1.11247400
H	-1.34483600	0.50225200	1.90642000
C	-1.74240400	-0.89687700	-0.62537600
O	-0.62462600	-1.66867000	-0.48455500
O	-2.73018000	-1.42014500	-1.07045000
H	1.32962100	0.11051300	1.56840000
C	-3.05124600	1.16196200	0.17582300
H	-3.45947600	0.47948300	0.93030300
H	-2.82408100	2.09207100	0.70346400
C	-1.01068400	1.35683100	-1.36662800
O	-0.04473700	0.90413800	-2.00583000
O	-1.54909400	2.51751000	-1.60260300
C	-0.96617700	3.34458100	-2.64554900
H	-1.54421000	4.26543900	-2.61388700
H	0.08476200	3.53118200	-2.42886300
H	-1.08827100	2.83325600	-3.60037400
C	-4.12867400	1.45964400	-0.85043000
C	-5.22621300	2.28895500	-0.44134200
C	-4.12264100	0.96322900	-2.13527800
C	-5.35596200	2.79268400	0.88107500
C	-6.27238200	2.58949800	-1.37451800
C	-5.15736400	1.25673800	-3.05139700
H	-3.32321700	0.31592000	-2.46146000
C	-6.44553400	3.54309600	1.26297000
H	-4.59317300	2.56851400	1.61244600
C	-7.37626400	3.37888300	-0.95552700
C	-6.20900100	2.05977400	-2.68793500
H	-5.11832000	0.82708200	-4.04693200
C	-7.46973900	3.84496300	0.33547400
H	-6.52282300	3.90092100	2.28581600
H	-8.15788500	3.59805000	-1.67817900
H	-7.00920200	2.28649300	-3.38711100
H	-8.32409700	4.43934900	0.64443200
S	1.87391500	4.05129500	-0.26224200
O	0.50796900	3.66501600	0.16005300
O	2.97132000	3.48169500	0.55469900
O	2.10174900	3.99249000	-1.73000900
C	1.99174600	5.87182100	0.09015800
F	0.95225200	6.52742500	-0.44292200
F	3.12485600	6.36588400	-0.43277300
F	2.00876300	6.11152100	1.41417200

(TfO•3c)⊂AuCav-2

C	5.99959700	-0.39416800	1.60208100
C	5.29971500	0.89277400	1.17711800
C	5.11377600	-1.62811600	1.45816100
H	6.19442400	-0.28865400	2.67006800
C	5.35802600	1.38962400	-0.12902400
C	4.60436200	1.66125400	2.12084700
C	4.94966900	-2.29264200	0.23946800
C	4.44759300	-2.16759900	2.56679900
C	4.79602300	2.61477500	-0.50139900
H	5.87889100	0.80865600	-0.88088500
C	4.03577900	2.89044500	1.80551200
O	4.55289600	1.19892800	3.43712400
C	4.19000200	-3.46047800	0.09803200
H	5.41954300	-1.87429300	-0.64405600
C	3.72138900	-3.34677500	2.48780700
O	4.49327300	-1.48522400	3.78972900
C	4.83189400	3.13480700	-1.93624400
C	4.15622200	3.35712000	0.50016200
H	3.53248900	3.48025800	2.56008300
C	3.37271900	0.64382500	3.84677200
C	3.96348500	-4.10079200	-1.26709100
C	3.60627800	-3.98580100	1.25707200
H	3.22039100	-3.74340300	3.36061900
C	3.34776200	-0.77772200	4.05552700
H	4.73147600	4.21903000	-1.87764700
C	3.60004000	2.62088600	-2.67547800
O	3.70772800	4.63543900	0.18002800
C	2.87871100	-3.33500000	-2.02140700
H	3.56376000	-5.09814800	-1.07901600
O	2.84818000	-5.16306100	1.19226200
C	3.57882900	1.36933700	-3.29799300
C	2.41714100	3.37264800	-2.70973400
C	2.36549000	4.89109900	0.20275700
C	1.17933200	0.77038300	4.44912600
C	3.16435900	-2.32425700	-2.94302300
C	1.52661100	-3.63832400	-1.79371500
C	1.51496800	-4.91557000	1.42382400
C	1.15187000	-0.64354400	4.65134300
C	2.44536700	0.84465200	-3.92707600
H	4.48411400	0.77389400	-3.28934800
C	1.25647500	2.88161300	-3.29646800
O	2.42138200	4.66190400	-2.18387300
C	1.67954600	4.91084700	-1.05904200
C	2.16477600	-1.64076000	-3.64458700
H	4.19994500	-2.06785300	-3.13094300
C	0.49501800	-3.00497200	-2.47583300
O	1.25936200	-4.68544600	-0.92095500
C	0.70836500	-4.49040800	0.31044700
C	2.46527100	-0.51390900	-4.62756200
C	1.27932100	1.62170200	-3.88502900
H	0.35128100	3.47612700	-3.29571800
C	0.44905400	5.51487600	1.25681900
C	0.84213100	-2.02046900	-3.39506900
H	-0.54629100	-3.27110600	-2.33173000
C	-0.24835800	-4.58836000	2.81949800
H	1.64129300	-0.50164100	-5.34509200
O	0.08203500	1.14802800	-4.42293900
C	-0.23252400	5.54585100	0.00032400
O	-0.19289700	-1.39250700	-4.10740900
C	-1.00214600	-4.03116000	1.74031800
C	-0.05175400	-1.26703000	5.05291000

C	0.00718200	1.52838500	4.66824400
C	-1.58383100	5.95653700	-0.04569200
C	-0.24046100	5.87627200	2.43485600
C	-2.29825500	-3.52509800	1.99117700
C	-0.82538700	-4.68008100	4.10612400
C	3.75532500	-0.74190600	-5.42853800
H	3.70217900	-1.69628600	-5.95907300
H	4.65008100	-0.76307400	-4.80077500
C	6.14719200	2.82610500	-2.66334800
H	6.13187300	3.26152400	-3.66614800
H	6.33670800	1.75473800	-2.77394100
C	7.35552800	-0.58464600	0.90302100
H	7.99088300	0.29043000	1.06539000
H	7.26094900	-0.73109400	-0.17661400
C	5.26442100	-4.26162200	-2.06567700
H	5.96598300	-4.88844700	-1.50894400
H	5.76508200	-3.30949800	-2.26380600
H	5.06217900	-4.73761000	-3.02926900
H	7.86241400	-1.46428400	1.30849500
H	6.98925300	3.25301000	-2.11191200
H	3.88751500	0.05845400	-6.16131900
C	-2.10221100	-4.20246600	4.31893600
C	-2.83057700	-3.61137700	3.26122900
H	-3.83037800	-3.23648200	3.44791900
H	-2.55029200	-4.27404500	5.30535600
H	-2.84951800	-3.08179400	1.17509700
H	-0.23340300	-5.12343700	4.89973100
C	-1.15404600	0.89964000	5.07017400
H	0.04540100	2.59774600	4.49621700
C	-1.18313600	-0.50360000	5.25647600
H	-0.05660900	-2.34332100	5.17347500
C	-1.55594900	6.27881300	2.36226400
H	0.29669900	5.82938500	3.37549800
C	-2.22582400	6.32687400	1.11810700
H	-2.08148600	5.98203300	-1.00910200
H	-3.25989200	6.65386500	1.08271400
H	-2.08924000	6.55409100	3.26583700
H	-2.11210100	-0.98307800	5.54787200
H	-2.06189300	1.47578000	5.20083100
P	-0.76078000	0.01866400	-3.53502300
N	-2.29884700	0.05521800	-4.06731900
C	-2.80058900	-0.72942900	-5.20241300
C	-3.20340400	1.08532200	-3.54664500
H	-2.16414500	-1.59763500	-5.35918900
H	-2.83729700	-0.11518800	-6.11019200
H	-3.79775000	-1.08514100	-4.93866600
H	-2.75815500	1.57667300	-2.67821900
H	-4.13037100	0.60247200	-3.23044900
H	-3.40231900	1.84801800	-4.30857800
Au	-0.33182700	0.22726300	-1.27538400
N	-0.49065100	-4.01734600	0.46600100
N	1.04723500	-4.99169700	2.63123200
N	2.27142200	-1.40030400	4.43523700
N	2.32372800	1.39150300	4.02603100
N	1.76987400	5.16541800	1.32498000
N	0.41857700	5.21301300	-1.15844800
C	-0.05644500	0.02418400	0.96811400
C	0.41415000	1.31312800	1.01397300
H	1.42590700	1.60567900	0.74848200
C	-1.39337300	-0.29356800	1.58128700
C	-2.33791100	0.93915000	1.63511700
H	-1.22283300	-0.67240800	2.59463800
H	-1.89242900	-1.08166400	1.02139000
C	-1.60003000	2.18897200	2.06627600
O	-0.29918800	2.35144600	1.48069700
O	-1.98620800	3.11076400	2.71366500
H	0.67886500	-0.77459400	0.87669000
C	-2.94511100	1.27603100	0.20892200
H	-2.19120000	1.84855800	-0.34377600
H	-3.06924500	0.32620600	-0.30840500
C	-3.49880400	0.59986700	2.58077600
O	-3.88376600	1.22570900	3.53886400
O	-4.05673100	-0.55003100	2.16260800
C	-5.27109500	-0.93706600	2.82759700
H	-5.60382200	-1.83438100	2.30935600
H	-5.07803600	-1.13421200	3.88469400
H	-6.01623700	-0.14577300	2.73255000
C	-4.23039700	2.07212200	0.23681200
C	-5.50240000	1.43936500	0.03731900
C	-4.17822300	3.43405700	0.46149800
C	-5.64533200	0.06070800	-0.27376400
C	-6.69211200	2.23890200	0.12828500
C	-5.35049900	4.21535500	0.54515300
H	-3.21869600	3.91884300	0.58872600
C	-6.88572000	-0.50090100	-0.47182300
H	-4.77304100	-0.56624300	-0.36116500
C	-7.95800000	1.62407300	-0.06443400
C	-6.58346100	3.62829000	0.39333700
H	-5.26965100	5.28136200	0.73465900
C	-8.05648200	0.28342400	-0.35735500
H	-6.95063900	-1.55238500	-0.72887900
H	-8.84960200	2.24033900	0.01499700
H	-7.49260200	4.21961800	0.46127800
H	-9.03021500	-0.17192500	-0.51171700
S	-3.40434900	-2.40369800	-2.09789200
O	-2.63051500	-1.67520400	-1.04264700
O	-4.51933200	-1.62814100	-2.67827800
O	-2.56746300	-3.16226100	-3.04683000
C	-4.29064600	-3.71995300	-1.12732100
F	-3.43923800	-4.64059700	-0.65830100
F	-5.20650400	-4.32999100	-1.88479200
F	-4.93110600	-3.16750200	-0.06486100

1c⊂AuCav

C	1.30321300	1.93795800	4.73861500
C	-0.02374700	2.06546300	3.99746400
C	2.37980600	1.30367200	3.86649600
H	1.12479400	1.24046800	5.55707200
C	-0.33633100	3.19508100	3.23717200
C	-1.01478400	1.07949700	4.10930400
C	3.18845200	2.05153300	3.00512500
C	2.61757500	-0.07399800	3.93317100
C	-1.59062000	3.40879000	2.65949700
H	0.41791800	3.96454100	3.12745500
C	-2.28186900	1.24224100	3.55217200
O	-0.74988500	-0.02682900	4.91873200
C	4.21816300	1.48372700	2.25043700
H	3.02074900	3.11988300	2.93434900
C	3.62883700	-0.69135700	3.20187800
O	1.86044400	-0.81786900	4.84263700
C	-1.87969300	4.66553800	1.84249300
C	-2.56037000	2.41285600	2.84648100
H	-3.05120400	0.49594100	3.70731100
C	-0.46235600	-1.24134800	4.37085200
C	5.07116000	2.30468500	1.28416000
C	4.41573300	0.10128900	2.37020000
H	3.81843700	-1.75258900	3.29878900
C	0.91704800	-1.66972300	4.35485600
H	-2.96061300	4.80483700	1.85006600
C	-1.47540400	4.38456300	0.39978300
O	-3.86810800	2.66659500	2.42835200
C	4.34542100	2.32011900	-0.05535300
H	6.00161700	1.75636000	1.13700600
O	5.49615400	-0.45282400	1.68780300
C	-0.16989100	4.55293800	-0.06813100
C	-2.41472100	3.84936500	-0.48991700
C	-4.42852800	1.98029400	1.39097600
C	-1.08222000	-3.26320700	3.51979100
C	3.35803400	3.26376500	-0.34891800
C	4.59188300	1.33087200	-1.01718000
C	5.28188100	-1.33538500	0.67010600
C	0.28081300	-3.68748400	3.51030500
C	0.22110100	4.17169800	-1.35560800
H	0.57170600	4.98257000	0.59534000
C	-2.07175100	3.43496400	-1.77121800
O	-3.72929600	3.76044500	-0.05151200
C	-4.31706500	2.53658700	0.06545800
C	2.57867700	3.22639000	-1.50979200
H	3.19849000	4.07554200	0.35012600
C	3.79321400	1.20917100	-2.15077000

O	5.68950500	0.48846200	-0.84866500
C	5.44849500	-0.84891000	-0.67366600
C	1.63569300	4.36775700	-1.88331800
C	-0.74958800	3.58451100	-2.17292800
H	-2.80994800	2.98470800	-2.42286800
C	-5.72961000	0.30580200	0.55102100
C	2.76797700	2.12562900	-2.35535700
H	3.98706000	0.42973900	-2.87414600
C	4.88077200	-3.42535500	-0.14279600
H	1.55935200	4.35016700	-2.97338700
O	-0.37833500	3.06096700	-3.41699100
C	-5.53564600	0.80750300	-0.77196600
O	1.97401200	1.94621800	-3.50070700
C	5.10660200	-2.95532900	-1.47397000
C	0.60816100	-4.97920200	3.03820200
C	-2.08686600	-4.14829000	3.06744600
C	-6.12054300	0.13525000	-1.86917000
C	-6.55421200	-0.82573700	0.74984900
C	4.98257500	-3.85090800	-2.55996000
C	4.52642600	-4.77659000	0.06876800
C	2.20781100	5.74355100	-1.50373600
H	3.20069900	5.87345700	-1.94147100
H	2.30045400	5.88361000	-0.42382400
C	-1.24179600	5.93532500	2.42058000
H	-1.53488300	6.80277600	1.82355800
H	-0.14886000	5.90242400	2.43570800
C	1.75860300	3.26681400	5.36204600
H	0.98022000	3.65828600	6.02208600
H	1.98045000	4.03806800	4.61938000
C	5.42702400	3.69911300	1.81399400
H	5.97307500	3.61171500	2.75667100
H	4.55178300	4.32788200	2.00018700
H	6.06160400	4.22270900	1.09419400
H	2.66568900	3.10806100	5.95073600
H	-1.58058200	6.09207400	3.44781200
H	1.55315500	6.53272500	-1.88187800
C	4.40324000	-5.62754100	-1.00874400
C	4.63404300	-5.16416700	-2.32654400
H	4.53459800	-5.85309200	-3.15894900
H	4.13140700	-6.66610000	-0.84994200
H	5.16003300	-3.47336600	-3.56080500
H	4.36043700	-5.10764300	1.08805200
C	-1.74237000	-5.40347500	2.60737800
H	-3.11393800	-3.80760800	3.09955200
C	-0.38958600	-5.81967700	2.58999000
H	1.65208700	-5.27229800	3.03608900
C	-7.14396500	-1.44180100	-0.33498100
H	-6.71111400	-1.16974800	1.76625500
C	-6.90992500	-0.97362700	-1.64990400
H	-5.93838200	0.52391300	-2.86441000
H	-7.36802400	-1.48696900	-2.48853400
H	-7.79926200	-2.29409300	-0.18731100
H	-0.14013000	-6.81066000	2.22560600
H	-2.51423200	-6.08668100	2.26646200
P	0.38397800	1.58636400	-3.39015700
N	0.09462100	0.93429600	-4.87087700
C	0.88236600	1.26432600	-6.06141200
C	-1.29084300	0.52379400	-5.13286400
H	1.90920100	1.49176100	-5.78066700
H	0.45593200	2.11924000	-6.60041300
H	0.88271200	0.39379000	-6.72404500
H	-1.27957500	-0.33878200	-5.80344500
H	-1.86335200	1.33576800	-5.59783600
H	-1.78861800	0.22295500	-4.20802600
Au	-0.34103800	0.51684300	-1.46885100
N	5.37833500	-1.63363400	-1.70964400
N	4.98864400	-2.57677200	0.92471500
N	1.27372400	-2.84448100	3.92584100
N	-1.42545000	-2.00772400	3.94710300
N	-5.11970500	0.90305200	1.62546200
N	-4.81737000	1.95270600	-0.98091600
C	-1.46388200	-0.45414300	0.44725900
C	-0.61094000	0.37814600	0.75427700
H	0.05920600	1.02883800	1.28086600
C	-2.62780500	-1.35163200	0.50972100
C	-2.97801200	-2.32584000	-0.63855100
H	-3.49578800	-0.71656100	0.69333700
H	-2.48675900	-1.93340900	1.42468400
C	-3.20568900	-1.57618900	-1.96892500
O	-2.76875100	-0.45692200	-2.16126500
O	-3.84885700	-2.23402300	-2.92520700
H	-4.31698900	-3.00909400	-2.51735600
C	-1.89986900	-3.47217200	-0.84911300
H	-2.20678300	-4.05936000	-1.71650200
H	-1.97818300	-4.12582300	0.02398900
C	-4.22453900	-3.14581900	-0.27932100
O	-4.87790000	-3.75339100	-1.11812200
O	-4.44766100	-3.24209200	1.02252400
C	-5.50626600	-4.14505100	1.42528000
H	-5.55483800	-4.06350200	2.50921100
H	-6.44636600	-3.84166000	0.96737700
H	-5.25890900	-5.16224400	1.11711100
C	-0.48223200	-2.99092600	-0.94629400
C	0.12830500	-2.62350400	-2.18768100
C	0.23585700	-2.89079500	0.22812500
C	-0.49217100	-2.79785600	-3.45483300
C	1.46045300	-2.08992900	-2.15215400
C	1.52110600	-2.31930400	0.26897100
H	-0.22649500	-3.22374600	1.14537000
C	0.18290100	-2.50589900	-4.61896500
H	-1.50456300	-3.17822000	-3.51601900
C	2.12619100	-1.79885800	-3.37179800
C	2.09922500	-1.88979500	-0.89940200
H	2.03928400	-2.21243600	1.21562100
C	1.50551200	-2.00877000	-4.58045400
H	-0.30137300	-2.67264800	-5.57595900
H	3.15000400	-1.44674100	-3.32861000
H	3.06145400	-1.40446700	-0.88902600
H	2.03046200	-1.79922500	-5.50688200

1c-TS1$_{5anti}$H^{+}⊂AuCav

C	-4.36782200	1.45112500	3.22048800
C	-2.98590000	0.95295900	3.63074900
C	-4.48795000	1.44857800	1.70070300
H	-4.42873300	2.49314600	3.53469300
C	-2.71750200	-0.37245500	3.97669800
C	-1.92238400	1.86484900	3.68461600
C	-4.78768300	0.26938700	1.01472000
C	-4.31230200	2.60844400	0.93329100
C	-1.45299800	-0.79194200	4.39934900
H	-3.52642000	-1.09284200	3.95245300
C	-0.64018300	1.49669100	4.08280400
O	-2.26283300	3.19240700	3.48134900
C	-4.93329500	0.20177500	-0.37142100
H	-4.93140000	-0.63626800	1.59251900
C	-4.42900500	2.58633500	-0.45770200
O	-4.08527100	3.82880200	1.57230800
C	-1.14665500	-2.23298300	4.79580000
C	-0.43473900	0.16708400	4.44065600
H	0.15359500	2.22874900	4.15729300
C	-1.83055100	3.96101000	2.44748700
C	-5.34773000	-1.09261300	-1.05932200
C	-4.71607200	1.38121900	-1.09578100
H	-4.29707300	3.49492000	-1.03239100
C	-2.82857200	4.37510600	1.49354300
H	-0.30895700	-2.19859400	5.49200300
C	-0.66267400	-2.98337800	3.56130800
O	0.77192300	-0.24401600	5.00704500
C	-4.14983500	-2.01157400	-1.25310600
H	-5.68769400	-0.81599700	-2.05728100
O	-4.84416000	1.34958700	-2.49069400
C	-1.57216000	-3.51870700	2.64600500
C	0.70053400	-3.13074700	3.27096700
C	1.93950700	-0.29231900	4.32346900
C	-0.31729600	5.34775300	1.47159200
C	-3.75017300	-2.94718000	-0.29592800
C	-3.40944000	-1.94240600	-2.44087300
C	-3.68149300	1.35827800	-3.21625600
C	-1.32959500	5.82722400	0.58613100
C	-1.19214700	-4.18905000	1.48206300

H	-2.62898500	-3.42011700	2.85979000
C	1.13226400	-3.76556600	2.10568800
O	1.64459400	-2.69062100	4.19671300
C	2.41893000	-1.59580700	3.92182200
C	-2.67962900	-3.82322700	-0.49890100
H	-4.30385700	-3.00884800	0.63298500
C	-2.34715200	-2.80567700	-2.69877500
O	-3.83451100	-1.03601400	-3.40385700
C	-3.11558800	0.10238400	-3.64228800
C	-2.22539700	-4.82568300	0.55489400
C	0.18227200	-4.26572200	1.21991000
H	2.19182500	-3.87179600	1.90614200
C	3.88823600	0.64088600	3.60664300
C	-2.01063700	-3.73899300	-1.72299300
H	-1.81737900	-2.75723700	-3.64146000
C	-1.96970700	2.45911500	-4.24846100
H	-1.71640300	-5.63400400	0.02522400
O	0.64155700	-4.94338900	0.08089700
C	4.37374500	-0.64892600	3.23233000
O	-0.98072900	-4.66051700	-1.99072700
C	-1.38005200	1.21271600	-4.61732800
C	-1.01815100	6.85024600	-0.33851400
C	0.98539400	5.89002900	1.39894800
C	5.68184100	-0.77538900	2.71188100
C	4.72097100	1.77291800	3.45096500
C	-0.15900800	1.20363900	-5.33225200
C	-1.32994700	3.66570800	-4.61332400
C	-3.39429600	-5.46701000	1.31740400
H	-4.06324700	-5.96938900	0.61424000
H	-3.99075000	-4.74360800	1.87873600
C	-2.31096800	-2.93516400	5.50768900
H	-2.01385300	-3.94349800	5.80768400
H	-3.20615400	-3.02727600	4.88655900
C	-5.51674400	0.69569400	3.90341700
H	-5.41231100	0.75631300	4.99003100
H	-5.55813800	-0.36338300	3.63331500
C	-6.52100500	-1.78280900	-0.34620600
H	-7.37654400	-1.10452300	-0.29942800
H	-6.28498400	-2.08193000	0.67874500
H	-6.81954900	-2.68043000	-0.89450400
H	-6.47405500	1.14166000	3.62203600
H	-2.58997500	-2.37531500	6.40394100
H	-3.01745700	-6.20645700	2.02835800
C	-0.13115200	3.63049000	-5.29307400
C	0.45920200	2.39529800	-5.64966200
H	1.40128800	2.38893000	-6.18745200
H	0.36496700	4.55650600	-5.56440300
H	0.25847400	0.24655000	-5.62706800
H	-1.80531900	4.60060900	-4.33764700
C	1.26446600	6.88714200	0.48817000
H	1.73467700	5.50869200	2.08409400
C	0.25838900	7.37174100	-0.38173600
H	-1.80663800	7.20338500	-0.99438000
C	6.00069300	1.62237000	2.95623100
H	4.32788900	2.73885100	3.74852500
C	6.48269100	0.34332000	2.59128000
H	6.03510200	-1.76663000	2.44855300
H	7.50191900	0.23915700	2.23564700
H	6.64778700	2.48740600	2.85340400
H	0.49604300	8.16395900	-1.08455300
H	2.26132500	7.31401900	0.43999700
P	0.51779400	-4.33709800	-1.43041000
N	1.38814200	-5.50414800	-2.24879200
C	2.73517400	-5.81689700	-1.75618200
C	1.29035500	-5.47527900	-3.71537300
H	2.72742400	-5.90095500	-0.67029600
H	3.47561300	-5.06238200	-2.06237400
H	3.03508900	-6.78197400	-2.17259500
H	0.25690300	-5.31046100	-4.01861300
H	1.60709700	-6.44922700	-4.09707000
H	1.92938600	-4.69828600	-4.15934600
Au	0.99066600	-2.12508900	-1.84546400
N	-1.99241800	0.03205800	-4.29498800
N	-3.12623900	2.49465900	-3.51980000
N	-2.58874000	5.28959000	0.60316000
N	-0.60800200	4.40159700	2.41613300
N	2.64146500	0.78787700	4.14917900
N	3.59052600	-1.76510400	3.38444300
C	2.69845500	0.01074700	-2.48069900
C	1.48610400	-0.28082700	-2.71559800
H	0.74617900	0.28199400	-3.26649700
C	4.01273700	-0.19129400	-1.84889500
C	4.46791200	1.15478100	-1.26358600
H	4.74179700	-0.55407400	-2.58018800
H	3.91158200	-0.94676900	-1.06744000
C	4.04609200	2.17012400	-2.33106700
O	3.18846000	1.82463200	-3.14518400
O	4.58684500	3.36219300	-2.35256900
H	5.37007500	3.35178900	-1.73750000
C	3.79399600	1.46446600	0.14064600
H	3.99408800	2.51047500	0.38420600
H	4.35178800	0.86595900	0.86061800
C	5.96430600	1.26837100	-1.02233900
O	6.52384000	2.35605400	-0.94013800
O	6.57072700	0.10351900	-0.87242100
C	8.00463100	0.14865900	-0.65115900
H	8.28352300	-0.86503600	-0.37201700
H	8.50352400	0.44964400	-1.57389900
H	8.23865800	0.85936800	0.14046400
C	2.33148800	1.11569200	0.25813400
C	1.30620400	1.97487400	-0.24736800
C	1.98070300	-0.09562600	0.82490300
C	1.56707400	3.23578100	-0.84342700
C	-0.05343100	1.52194300	-0.20044900
C	0.63333100	-0.51342400	0.91666200
H	2.75682200	-0.75207300	1.19997700
C	0.56101000	3.97097700	-1.42573600
H	2.57136000	3.63996400	-0.84478800
C	-1.06986000	2.31672200	-0.78061900
C	-0.36674800	0.27923200	0.40476500
H	0.39247700	-1.45564500	1.38596900
C	-0.77183400	3.50761500	-1.39666200
H	0.78521800	4.92928400	-1.87841900
H	-2.09064000	1.96208600	-0.73329900
H	-1.40581200	-0.03341300	0.46031700
H	-1.56489900	4.09983700	-1.83351600

1c-Int$_{5anti}$H^{+}⊂AuCav

C	-3.90327500	2.41159300	3.22739000
C	-2.62697600	1.69077900	3.64817800
C	-4.06334400	2.33561100	1.71338400
H	-3.76109100	3.46436400	3.47167600
C	-2.59850300	0.35822000	4.06241300
C	-1.41297100	2.39144900	3.63737200
C	-4.61114900	1.19994100	1.11217400
C	-3.68321800	3.38699300	0.86808900
C	-1.42348700	-0.26569500	4.49068800
H	-3.52560600	-0.20199100	4.08457700
C	-0.21219200	1.81453500	4.04057500
O	-1.50431900	3.74614600	3.35912600
C	-4.80844700	1.07738300	-0.26378300
H	-4.91816600	0.38020900	1.75104600
C	-3.83911800	3.30151800	-0.51648000
O	-3.21613200	4.58068600	1.42250500
C	-1.37812200	-1.71908300	4.95150400
C	-0.24650400	0.49032700	4.46795400
H	0.70422300	2.39002200	4.06407200
C	-0.96045900	4.35668900	2.27412100
C	-5.49886800	-0.14379700	-0.85800300
C	-4.37926100	2.14139100	-1.06839300
H	-3.55262600	4.13071800	-1.15188000
C	-1.88417600	4.88284400	1.30016600
H	-0.52560500	-1.81205200	5.62395500
C	-1.08428300	-2.59872300	3.74278800
O	0.87981700	-0.10596900	5.03645400
C	-4.51496000	-1.29204200	-1.03190500
H	-5.81959700	0.14151900	-1.85983600
O	-4.56320500	2.05581400	-2.45384600
C	-2.10933300	-3.01150400	2.88839000
C	0.21932600	-2.99486200	3.41565700

C	1.99841400	-0.39748200	4.33246000
C	0.75889500	5.39229900	1.20749600
C	-4.25272400	-2.22382800	-0.02506200
C	-3.83588700	-1.44440800	-2.24773400
C	-3.45937500	1.78109700	-3.21781600
C	-0.16651300	5.99233400	0.30097800
C	-1.89903600	-3.79695400	1.75398000
H	-3.12399400	-2.72280700	3.13173600
C	0.48761400	-3.74712600	2.27169900
O	1.25835600	-2.69956700	4.29917300
C	2.21448100	-1.78110000	3.96959800
C	-3.37629000	-3.29822700	-0.20347400
H	-4.75933000	-2.11779800	0.92661100
C	-2.96678500	-2.50692800	-2.48035300
O	-4.12366700	-0.53226500	-3.25720500
C	-3.19819400	0.41456700	-3.59668800
C	-3.06475200	-4.29687900	0.90392900
C	-0.57033600	-4.12387500	1.44785100
H	1.50271400	-4.05224100	2.04748100
C	4.06754000	0.12925800	3.54339400
C	-2.76062200	-3.42155800	-1.45228800
H	-2.47747100	-2.61566400	-3.43958700
C	-1.61456100	2.43955700	-4.38831700
H	-2.72684700	-5.21453500	0.41743400
O	-0.28629500	-4.94345800	0.35001200
C	4.29506100	-1.24079900	3.21145900
O	-1.92752200	-4.52965300	-1.68343300
C	-1.32654600	1.07997700	-4.71347900
C	0.30498800	6.89733500	-0.67736500
C	2.13479100	5.69559900	1.10052700
C	5.54515200	-1.62832400	2.67721600
C	5.09229000	1.07966000	3.32926900
C	-0.18573400	0.77478200	-5.49254900
C	-0.76479400	3.46316200	-4.86692600
C	-4.29799400	-4.66922200	1.74013600
H	-5.06962900	-5.09179900	1.09178500
H	-4.73897600	-3.81579900	2.26126700
C	-2.62814800	-2.15615000	5.72704200
H	-2.51722900	-3.18962800	6.06560400
H	-3.54622100	-2.10050700	5.13580000
C	-5.15186000	1.92887400	3.97913100
H	-5.00722400	2.04102100	5.05699600
H	-5.39452700	0.88018800	3.78545800
C	-6.75406300	-0.54809400	-0.06907100
H	-7.45489100	0.28962300	-0.03092500
H	-6.53803900	-0.84133000	0.96197900
H	-7.24840800	-1.39226700	-0.55729500
H	-6.01858900	2.52469300	3.68193600
H	-2.76364500	-1.51713900	6.60335200
H	-4.03019600	-5.41409000	2.49371400
C	0.34484500	3.14052600	-5.61839700
C	0.63847900	1.79119600	-5.92804900
H	1.51305000	1.55880500	-6.52703100
H	0.99710400	3.92645500	-5.98516500
H	0.00479000	-0.26450200	-5.73804700
H	-1.01653200	4.49075800	-4.62933700
C	2.56993100	6.58241200	0.13814700
H	2.81525400	5.22824900	1.80404800
C	1.65146100	7.18867400	-0.75200000
H	-0.41928700	7.34786900	-1.34745400
C	6.31068800	0.67453200	2.82237000
H	4.89338200	2.11166200	3.59679500
C	6.53805700	-0.68442600	2.50150500
H	5.70093600	-2.67683900	2.44711800
H	7.51256100	-0.99060300	2.13677200
H	7.10661900	1.39896600	2.68164600
H	2.01255700	7.89274200	-1.49498800
H	3.62449900	6.82967100	0.06582800
P	-0.36463700	-4.43273000	-1.20553900
N	0.23150200	-5.81269000	-1.94379200
C	1.53193800	-6.31115300	-1.48160800
C	0.06198600	-5.88889900	-3.40180600
H	1.57202000	-6.30035200	-0.39306100
H	2.37145600	-5.72531200	-1.88631600
H	1.64108500	-7.34572100	-1.81770800
H	-0.94000100	-5.56307800	-3.67931300
H	0.17766200	-6.93290800	-3.70476600
H	0.80450000	-5.28212600	-3.94027800
Au	0.51347500	-2.38940400	-1.83196500
N	-2.15515800	0.07512100	-4.29548900
N	-2.69171400	2.75685100	-3.60700700
N	-1.49978700	5.68611100	0.35440300
N	0.32049000	4.56876300	2.20826700
N	2.88471000	0.52566400	4.10370000
N	3.32147600	-2.18481000	3.41914900
C	2.61636900	-0.45319700	-2.56293900
C	1.34182000	-0.73440900	-2.71488100
H	0.74476600	-0.04225800	-3.30050000
C	3.77667900	-1.03233000	-1.80070400
C	4.53557000	0.22611500	-1.34434000
H	4.41185400	-1.65100700	-2.44546300
H	3.43047100	-1.63988100	-0.96716200
C	4.19240700	1.16890400	-2.47641300
O	3.16198900	0.83301000	-3.14081600
O	4.89000500	2.19235700	-2.81038500
H	5.74930000	2.12404800	-2.27508400
C	4.00894400	0.76514500	0.06389200
H	4.44539100	1.75243800	0.23274500
H	4.45790400	0.08834800	0.79032500
C	6.04447000	0.14995700	-1.22942200
O	6.75080700	1.13768900	-1.43474200
O	6.49646900	-1.02749500	-0.86201000
C	7.93822500	-1.15308400	-0.72237900
H	8.09756700	-2.14804100	-0.31358000
H	8.40726200	-1.05230200	-1.70214000
H	8.31394100	-0.38110900	-0.05129300
C	2.51233700	0.75090900	0.22302800
C	1.69516200	1.77347200	-0.35105800
C	1.91229300	-0.31799700	0.86259200
C	2.22155800	2.91135600	-1.01599700
C	0.27123200	1.61714400	-0.30829600
C	0.50782300	-0.43039500	0.96498600
H	2.53087200	-1.10317400	1.27968600
C	1.40057200	3.79101100	-1.68481900
H	3.28640700	3.11040400	-0.99124600
C	-0.54671500	2.55660300	-0.97855700
C	-0.29922900	0.51470700	0.37767500
H	0.07195800	-1.27006900	1.48807800
C	0.00179400	3.60839700	-1.67263600
H	1.82498800	4.65242700	-2.18656700
H	-1.61954900	2.42567800	-0.94072700
H	-1.38107100	0.42771900	0.42734500
H	-0.64509400	4.31305100	-2.17837300

1c-TS1$_{5syn}$H^{+}⊂AuCav

C	4.82924600	1.68825400	2.73903500
C	4.43269700	2.28927900	1.39191000
C	3.87513200	0.55940100	3.12313900
H	4.68710500	2.47603600	3.47986200
C	4.83752900	1.71376000	0.18176200
C	3.64231500	3.44304400	1.30988600
C	4.12313200	-0.78519500	2.82679600
C	2.69165400	0.85064400	3.81409700
C	4.49391400	2.23592600	-1.06919100
H	5.44727700	0.81827400	0.21755300
C	3.27023400	3.99988800	0.09260800
O	3.21928600	4.07922600	2.48536100
C	3.25150500	-1.81471200	3.19734700
H	5.04043600	-1.04132700	2.31048900
C	1.78022400	-0.12959600	4.18823100
O	2.48986700	2.17202000	4.21087200
C	4.95675800	1.59610000	-2.37603200
C	3.69286900	3.38405900	-1.07727200
H	2.65527800	4.88907100	0.05822800
C	1.88702400	3.95399400	2.75546800
C	3.52959600	-3.28833900	2.90423500
C	2.07702100	-1.44894600	3.86822700
H	0.88338900	0.12189200	4.73746800
C	1.48915300	2.89305000	3.64581000
H	4.85583900	2.36227000	-3.14560600

C	4.01992200	0.46037000	-2.77497000
O	3.31705900	3.93832300	-2.30700500
C	2.87784600	-3.67863200	1.58165000
H	3.02053200	-3.85980700	3.68131700
O	1.24690900	-2.47380600	4.30927600
C	4.21413500	-0.85692000	-2.34535300
C	2.90617200	0.71189100	-3.58928300
C	2.00173100	3.78359100	-2.63295000
C	-0.29416100	4.55393100	2.51643100
C	3.49351900	-3.39860700	0.35517800
C	1.61369500	-4.28405200	1.54285300
C	0.01525200	-2.65153400	3.76044300
C	-0.68953500	3.48120900	3.37182300
C	3.35198000	-1.90718800	-2.68222600
H	5.07430500	-1.07405300	-1.72390100
C	2.02312500	-0.29497300	-3.95522800
O	2.70427200	2.00560800	-4.06820700
C	1.66607000	2.73244600	-3.55999000
C	2.89678500	-3.65951600	-0.88269800
H	4.47280000	-2.93472100	0.36751200
C	0.97696700	-4.55762900	0.33796200
O	0.94901300	-4.60783300	2.72602200
C	-0.16587500	-3.84717100	2.98302700
C	3.58187700	-3.34710800	-2.21593600
C	2.25509900	-1.58403000	-3.49175700
H	1.16148400	-0.07224600	-4.57101800
C	-0.19839700	4.34333000	-2.48138500
C	1.61614300	-4.22348900	-0.84451400
H	-0.01728500	-4.98630500	0.33123900
C	-2.19091400	-2.15653500	3.50533600
H	3.10059700	-3.98589800	-2.96109400
O	1.29225500	-2.55427900	-3.80813500
C	-0.53205200	3.30380200	-3.40080600
O	0.86721500	-4.36392900	-2.02848900
C	-2.38789500	-3.38543300	2.80265900
C	-2.06066400	3.29344300	3.66699500
C	-1.27841800	5.41681500	1.98185900
C	-1.88378900	3.11030700	-3.76618700
C	-1.22270900	5.15854900	-1.94415300
C	-3.69036000	-3.75548800	2.39645800
C	-3.29955100	-1.31381700	3.75305900
C	5.07110400	-3.72648100	-2.19711900
H	5.18380600	-4.78383200	-1.94512800
H	5.64651000	-3.15167000	-1.46692400
C	6.43232900	1.17230200	-2.33463800
H	6.73325700	0.75854300	-3.30082000
H	6.64085100	0.41764100	-1.57119500
C	6.30661300	1.27269400	2.78485700
H	6.94266200	2.13597800	2.57364600
H	6.55725000	0.49621400	2.05668000
C	5.02042900	-3.64925900	2.96244200
H	5.43375500	-3.38175600	3.93834400
H	5.61654300	-3.13933800	2.20047500
H	5.15008400	-4.72445600	2.81452300
H	6.55942000	0.88989400	3.77717500
H	7.06176900	2.03887900	-2.11741300
H	5.51557000	-3.55239100	-3.18026000
C	-4.55997000	-1.70054700	3.34589300
C	-4.75772400	-2.92875000	2.67310700
H	-5.75662500	-3.20875800	2.35778400
H	-5.41516200	-1.06217900	3.54401100
H	-3.81524400	-4.69431600	1.86936700
H	-3.12610200	-0.38898600	4.29347000
C	-2.60691800	5.22277300	2.29702200
H	-0.94952900	6.23131300	1.34586900
C	-2.99988500	4.15787500	3.14169400
H	-2.33288500	2.47794800	4.32866900
C	-2.53636700	4.94345200	-2.31173300
H	-0.93753100	5.94377700	-1.25300500
C	-2.86789300	3.91526200	-3.22710500
H	-2.11728300	2.31495900	-4.46474200
H	-3.90460600	3.76456500	-3.50930800
H	-3.32100700	5.57638100	-1.90857000
H	-4.04930700	4.02393200	3.38299900
H	-3.35751200	5.89886700	1.90083400
P	0.26656700	-2.94011800	-2.56812800
N	-1.16377100	-3.40239200	-3.25642900
C	-1.99674800	-2.31759500	-3.79639500
C	-1.30127700	-4.68821800	-3.95374200
H	-1.82431600	-1.39436300	-3.23988000
H	-3.04957600	-2.58790500	-3.68300700
H	-1.77831900	-2.13682700	-4.85609500
H	-0.70337700	-5.44845700	-3.45248000
H	-0.98948800	-4.61238500	-5.00256000
H	-2.35274300	-4.98751600	-3.91893400
Au	0.29172900	-1.21196700	-1.01287300
N	-1.32507600	-4.20378000	2.51759900
N	-0.95262200	-1.81094700	3.97987500
N	0.24323900	2.64453400	3.92435100
N	1.02441000	4.75440100	2.20433600
N	1.09982000	4.55587500	-2.10485900
N	0.44131200	2.49303000	-3.92473400
C	-0.83746700	1.28999900	-0.05765200
C	0.08800700	0.45946900	0.24320400
H	0.65853800	0.55779400	1.16646000
C	-1.90710600	2.27805300	0.15077800
C	-3.21968000	1.58226500	-0.28750100
H	-1.73821600	3.19519000	-0.41720000
H	-1.93882200	2.52586900	1.21538400
C	-2.83804000	0.95848800	-1.62851500
O	-1.63680200	0.71648800	-1.78829900
O	-3.72496400	0.67661600	-2.53658300
H	-4.58167200	1.11116200	-2.25791800
C	-3.56350200	0.42834600	0.74839800
H	-2.61841800	0.15125400	1.21592500
H	-4.17907200	0.86652400	1.53505600
C	-4.42764400	2.48308400	-0.44824400
O	-5.26423000	2.32189600	-1.33091400
O	-4.52487700	3.40453700	0.49280900
C	-5.69476300	4.25887100	0.43174400
H	-5.67155300	4.85057300	-0.48462900
H	-6.60077900	3.65191200	0.45192800
H	-5.62856700	4.89710400	1.30984800
C	-4.17107800	-0.81439500	0.15113300
C	-5.58441600	-0.99307900	0.03490200
C	-3.31693000	-1.80164700	-0.29969500
C	-6.53319100	-0.05610200	0.52673000
C	-6.07516600	-2.19627300	-0.57540900
C	-3.80029400	-2.97984000	-0.90767700
H	-2.24465600	-1.67821500	-0.17537700
C	-7.88594500	-0.28761000	0.41972800
H	-6.19195500	0.85186100	1.01127900
C	-7.47675500	-2.39919100	-0.67866000
C	-5.15443200	-3.16839300	-1.04948700
H	-3.09141300	-3.72456200	-1.25449100
C	-8.36565600	-1.46829500	-0.19362000
H	-8.59219700	0.43834700	0.81014600
H	-7.83582100	-3.31228200	-1.14456100
H	-5.54377200	-4.07095200	-1.51188300
H	-9.43431700	-1.63793900	-0.27405800

1c-Int$_{5syn}$H^{+}⊂AuCav

C	2.92242400	4.13714500	-2.61661400
C	2.53305700	2.74857200	-3.11717500
C	2.62040900	4.24739100	-1.12513900
H	2.26211500	4.84360000	-3.12063200
C	3.44382200	1.68954900	-3.14577200
C	1.23665400	2.48571600	-3.58225500
C	3.50915300	3.80164500	-0.14060300
C	1.40126400	4.77593000	-0.68798400
C	3.12496700	0.41852200	-3.63102500
H	4.45663600	1.87361300	-2.80972200
C	0.85513300	1.22840500	-4.04215400
O	0.35267000	3.55940800	-3.70552800
C	3.21677900	3.85057900	1.22684400
H	4.46430400	3.39403600	-0.45178800
C	1.05632100	4.83877800	0.65477300
O	0.50929500	5.27565900	-1.64276000
C	4.16600900	-0.69324800	-3.73030100
C	1.80651300	0.21266200	-4.05881500

H	-0.14097000	1.06088600	-4.43130000
C	-0.73946100	3.66581100	-2.90320100
C	4.20466200	3.38512800	2.29499400
C	1.96199900	4.35608100	1.58961400
H	0.09938000	5.23550700	0.96477700
C	-0.65734500	4.59277000	-1.80032200
H	3.83036900	-1.35702600	-4.52771800
C	4.17905200	-1.52450300	-2.45291900
O	1.48936000	-1.01432600	-4.64825300
C	4.10758200	1.87894300	2.51735000
H	3.88591900	3.85141900	3.22799000
O	1.61214300	4.38132400	2.94280700
C	4.89013200	-1.13823400	-1.31198700
C	3.43561300	-2.70721400	-2.37235500
C	0.69913400	-1.91599500	-4.01006900
C	-2.91380400	3.25189100	-2.37359300
C	4.85612100	0.96266500	1.77064300
C	3.25113200	1.35674000	3.49698700
C	0.69106000	3.44290400	3.31503100
C	-2.81760700	4.13423500	-1.25537900
C	4.86446400	-1.86430400	-0.11603300
H	5.46970700	-0.22318500	-1.34969100
C	3.38985000	-3.47188600	-1.21502900
O	2.71042600	-3.12195600	-3.49095800
C	1.35084700	-3.05048800	-3.40080100
C	4.77910100	-0.42229300	1.95648100
H	5.53389500	1.34476400	1.01703600
C	3.13561300	-0.00990200	3.71799600
O	2.51471400	2.23596300	4.29029700
C	1.16987300	2.30175400	4.04574200
C	5.61058600	-1.40884700	1.13735900
C	4.08863700	-3.02981900	-0.09880200
H	2.78571000	-4.36890600	-1.17395600
C	-1.32447400	-2.80787300	-3.46658200
C	3.89343200	-0.87503700	2.94145600
H	2.45917700	-0.38721900	4.47365600
C	-1.42707200	2.61654500	3.42170500
H	5.73377600	-2.29780900	1.76113600
O	3.90168100	-3.74145900	1.09346800
C	-0.67806000	-3.91191800	-2.83644100
O	3.72745800	-2.25035100	3.17489400
C	-0.95689100	1.50118200	4.17985700
C	-3.94854200	4.35014900	-0.43449100
C	-4.15403800	2.64553900	-2.67253200
C	-1.45602000	-4.94843100	-2.27041600
C	-2.73828200	-2.78458900	-3.54096500
C	-1.88291200	0.54092700	4.65033700
C	-2.80524100	2.72531700	3.11964500
C	7.01840200	-0.88249100	0.82045000
H	7.53679700	-0.61392800	1.74442600
H	7.00809800	0.00181000	0.17802700
C	5.56179100	-0.18031300	-4.11420800
H	6.24234100	-1.02446000	-4.25249800
H	6.00246000	0.47445900	-3.35742700
C	4.36636900	4.52595200	-2.96285900
H	4.52839900	4.45634300	-4.04177300
H	5.11184500	3.89224100	-2.47443000
C	5.63897300	3.85495000	2.00863500
H	5.66574700	4.94513200	1.93591800
H	6.04246300	3.45505300	1.07415500
H	6.30603600	3.54446100	2.81727400
H	4.55787000	5.55520800	-2.64897200
H	5.51237900	0.38238700	-5.05000400
H	7.59771700	-1.65397400	0.30700700
C	-3.68822300	1.77909600	3.59559800
C	-3.22597900	0.69098000	4.37518800
H	-3.94218100	-0.02878100	4.75474400
H	-4.74798500	1.86379400	3.37771500
H	-1.50629100	-0.28483100	5.24458700
H	-3.13200500	3.57337900	2.52781900
C	-5.25347300	2.90438400	-1.88018500
H	-4.20729200	1.99886700	-3.54043700
C	-5.14918900	3.74490800	-0.74647600
H	-3.84509300	5.02504500	0.40831800
C	-3.47445100	-3.82647600	-3.01146400
H	-3.20826700	-1.95658400	-4.06200900
C	-2.83125200	-4.90750700	-2.36369900
H	-0.93773200	-5.77569200	-1.79796600
H	-3.42724200	-5.71328300	-1.94849100
H	-4.55627400	-3.82264200	-3.09165600
H	-6.02466400	3.92410500	-0.13078500
H	-6.21481500	2.46859500	-2.13282500
P	2.77863000	-3.06278300	2.11523700
N	2.22486800	-4.30127600	3.06144600
C	1.15194800	-5.12868400	2.50153100
C	3.12529800	-5.00266400	3.98624000
H	0.51025800	-4.52210700	1.85637200
H	0.54411400	-5.52288900	3.32106400
H	1.54671300	-5.97016300	1.91702200
H	3.84458600	-4.29964300	4.40514700
H	3.66206600	-5.81724800	3.48429900
H	2.52935500	-5.41994000	4.80279600
Au	1.31857800	-1.71486200	0.88621200
N	0.37591900	1.35949300	4.46130600
N	-0.56088700	3.58471600	2.99399600
N	-1.65096500	4.79766400	-0.98817500
N	-1.82786800	3.00691900	-3.17521300
N	-0.59689400	-1.79054400	-4.02549000
N	0.68722900	-3.99785600	-2.80846200
C	-1.16080000	-0.83865600	-0.55747800
C	0.08532800	-0.53239600	-0.25213100
H	0.42363900	0.42329400	-0.65255900
C	-2.32537000	-0.21172700	-1.28849100
C	-3.54499000	-0.86859100	-0.60616300
H	-2.29038000	-0.43851700	-2.35689600
H	-2.33080500	0.87147400	-1.17574000
C	-2.94812300	-2.16720600	-0.13725800
O	-1.67878400	-2.14303200	-0.04048000
O	-3.61236400	-3.20362000	0.20102100
H	-4.56340100	-3.02228400	-0.11274500
C	-3.93200800	-0.03633700	0.69938200
H	-4.17481900	0.96568700	0.34160500
H	-3.01238000	0.05856100	1.28348700
C	-4.79466900	-1.17897400	-1.40227800
O	-5.49938300	-2.14658200	-1.11723600
O	-5.06474500	-0.32156300	-2.35976000
C	-6.32229600	-0.51583000	-3.06222600
H	-6.35253100	0.27370800	-3.80969900
H	-6.33678400	-1.50005700	-3.53120300
H	-7.14507100	-0.42527400	-2.35129200
C	-5.02488700	-0.63269900	1.54329100
C	-6.39932300	-0.29303600	1.33219800
C	-4.69414200	-1.54396500	2.52948900
C	-6.82298800	0.64799300	0.35627700
C	-7.40038200	-0.92424300	2.14203000
C	-5.68105100	-2.16369300	3.32683400
H	-3.64851100	-1.77525700	2.71080300
C	-8.15736800	0.94196200	0.18075100
H	-6.08806500	1.15251300	-0.25579900
C	-8.76748900	-0.59924100	1.93506400
C	-7.00848900	-1.86092700	3.13328400
H	-5.38426000	-2.87443100	4.09107200
C	-9.14340100	0.31057200	0.97457800
H	-8.45653800	1.67374500	-0.56436400
H	-9.51517000	-1.08485500	2.55517900
H	-7.77638200	-2.33220700	3.73947700
H	-10.19109900	0.55161400	0.82842600

1c-TS1$_{6}$H^{+}$\subset$AuCav

C	3.39153300	-3.29485300	3.02480600
C	3.68302800	-3.12896700	1.53800800
C	2.12862800	-2.52943400	3.40478700
H	4.21487600	-2.81851300	3.55725200
C	2.97966300	-3.87813400	0.59396300
C	4.65529200	-2.24250900	1.05377900
C	0.87059800	-3.12347800	3.51195700
C	2.22785500	-1.16318700	3.70339400
C	3.21012600	-3.80111900	-0.78068800
H	2.21266400	-4.55759800	0.94851600
C	4.92535300	-2.13418700	-0.31096600

O	5.40176300	-1.47930600	1.95415100
C	-0.25563900	-2.41503200	3.94052500
H	0.77375100	-4.18200400	3.30259300
C	1.13400400	-0.40648300	4.11690400
O	3.51981100	-0.66146300	3.74325700
C	2.42023700	-4.67918500	-1.74328100
C	4.18369800	-2.89293900	-1.21653300
H	5.68419900	-1.44629100	-0.66364900
C	5.10251000	-0.14084900	2.04130600
C	-1.62611100	-3.06450800	4.10091300
C	-0.09325400	-1.05630000	4.23732900
H	1.24624600	0.63480500	4.39040700
C	4.03469500	0.28253100	2.91480900
H	2.91812400	-4.61147100	-2.71067300
C	1.02203400	-4.10701100	-1.93261900
O	4.42565200	-2.74739300	-2.59074400
C	-2.41154300	-2.90058400	2.80588000
H	-2.15652400	-2.49491900	4.86359600
O	-1.18699300	-0.41752300	4.82387800
C	-0.08978900	-4.53783000	-1.20826900
C	0.82111000	-3.10489800	-2.89078400
C	3.92570500	-1.60830700	-3.17127700
C	5.49671400	2.03558200	1.50197400
C	-2.19160700	-3.75056900	1.71936700
C	-3.34521000	-1.86829700	2.63734900
C	-1.86852900	0.59171700	4.22529000
C	4.38718500	2.44337400	2.30211100
C	-1.37858100	-4.04462300	-1.43607300
H	0.04169700	-5.32085300	-0.47246200
C	-0.43733500	-2.56372200	-3.14357100
O	1.91487100	-2.79734000	-3.68125800
C	2.56435500	-1.60068000	-3.65241000
C	-2.86956500	-3.62993300	0.50538900
H	-1.45324600	-4.53638400	1.82298600
C	-4.05062600	-1.70699300	1.44351600
O	-3.60321100	-1.01297900	3.70322700
C	-3.14974300	0.28005900	3.63769200
C	-2.58879400	-4.56107700	-0.66796100
C	-1.51803200	-3.05238200	-2.41399500
H	-0.57024600	-1.82761700	-3.92619300
C	4.15414300	0.54950400	-3.87283000
C	-3.79361100	-2.58622400	0.39715900
H	-4.77995400	-0.91234300	1.34565900
C	-2.20657000	2.79834900	3.76958300
H	-3.44624100	-4.49979500	-1.34208800
O	-2.80691200	-2.66416700	-2.81875200
C	2.78188500	0.57911100	-4.26466700
O	-4.52017700	-2.45276900	-0.80022000
C	-3.46186000	2.48742900	3.16168300
C	4.04885400	3.81333700	2.37745600
C	6.24966700	3.00962300	0.80693100
C	2.24699700	1.75380200	-4.83948100
C	4.95889300	1.69597600	-4.06083600
C	-4.24587800	3.52870900	2.61385200
C	-1.78606000	4.14637300	3.84796900
C	-2.47022700	-6.03113300	-0.24177600
H	-3.40084300	-6.35406100	0.23111500
H	-1.66253600	-6.20820200	0.47257000
C	2.42582600	-6.15524100	-1.31947900
H	1.86858000	-6.75938600	-2.04081500
H	1.98530200	-6.31979300	-0.33216700
C	3.36199200	-4.77100900	3.44727400
H	4.32159700	-5.24165300	3.21908000
H	2.58600600	-5.34917200	2.93736900
C	-1.56420500	-4.52443000	4.57187100
H	-1.01740500	-4.58829400	5.51604100
H	-1.06843300	-5.18800200	3.85792500
H	-2.57487700	-4.90964700	4.73031200
H	3.18397100	-4.85126000	4.52302200
H	3.45383100	-6.52356400	-1.28126600
H	-2.28683700	-6.66232000	-1.11497100
C	-2.57568800	5.14451000	3.31586600
C	-3.80563100	4.83480100	2.68775700
H	-4.39865800	5.63253900	2.25524600
H	-2.25438000	6.17906500	3.36803400
H	-5.19861300	3.27092700	2.16339400
H	-0.83776600	4.35648000	4.33062100
C	5.90264900	4.34203200	0.90089900
H	7.09242300	2.67489300	0.21173500
C	4.79570400	4.74472800	1.68674600
H	3.20202900	4.09622100	2.99316900
C	4.41863600	2.82614300	-4.63779900
H	5.99770600	1.64752700	-3.75295100
C	3.06045200	2.85087400	-5.03596600
H	1.20779100	1.74637300	-5.15039500
H	2.66332100	3.73589600	-5.52416100
H	5.04071000	3.70005700	-4.80283000
H	4.54196000	5.79814000	1.75155800
H	6.48522400	5.09152500	0.37459100
P	-3.87124700	-1.69198200	-2.08552300
N	-5.14230500	-1.82930300	-3.15989800
C	-4.85253700	-1.54285500	-4.57291100
C	-6.43603400	-1.29500800	-2.70857800
H	-3.92677300	-2.03218000	-4.87261600
H	-4.78052600	-0.46350700	-4.77223900
H	-5.66743000	-1.95263200	-5.17462400
H	-6.63138600	-1.60114600	-1.68138700
H	-7.21716100	-1.71697100	-3.34555500
H	-6.48126600	-0.19835000	-2.78127200
Au	-3.21476300	0.46312000	-1.61179200
N	-3.90814600	1.19231300	3.10505500
N	-1.41406300	1.80899500	4.28153200
N	3.66740200	1.52538200	3.01673100
N	5.81675600	0.71064200	1.37011000
N	4.69322600	-0.56738400	-3.29222700
N	2.00097400	-0.53873000	-4.14578400
C	-2.88837800	2.57735500	-1.48288700
C	-3.98043300	2.90711900	-0.97407800
H	-4.96796200	2.97502000	-0.57095800
C	-1.63384300	3.29646800	-1.84376600
C	-1.22480500	4.27805600	-0.72915900
H	-1.81564100	3.83218800	-2.78022500
H	-0.81191000	2.60469600	-2.01847500
C	-2.35224700	5.28565300	-0.35478800
O	-3.53886600	5.07085000	-0.55777200
O	-1.97937200	6.36833300	0.30420200
H	-0.99001500	6.43751700	0.28977500
C	-0.94121200	3.51414300	0.64051400
H	-0.28108300	4.14360800	1.24073700
H	-1.89116900	3.47132300	1.16832500
C	0.01657600	5.06752600	-1.13960000
O	0.46226500	5.98360100	-0.45441100
O	0.56665600	4.67338800	-2.27416500
C	1.80096200	5.33394400	-2.64682000
H	2.17706300	4.76767700	-3.49368700
H	1.59682300	6.37238700	-2.91225700
H	2.50434700	5.29622800	-1.81476000
C	-0.42322000	2.10308700	0.51438700
C	0.90916100	1.81775000	0.08955100
C	-1.27348300	1.04872200	0.80380700
C	1.87930600	2.82531500	-0.15415900
C	1.29603000	0.44973200	-0.09682800
C	-0.86866800	-0.30156900	0.67053300
H	-2.27522300	1.26196800	1.15372600
C	3.13124900	2.50979800	-0.62718600
H	1.65946200	3.85489200	0.09461300
C	2.59907800	0.16045500	-0.56250000
C	0.38970500	-0.59654100	0.20782900
H	-1.55340200	-1.09424200	0.93315600
C	3.49710500	1.16270400	-0.83749900
H	3.86552000	3.29112900	-0.78469400
H	2.87531000	-0.87670900	-0.67673400
H	0.71238000	-1.62654200	0.08767500
H	4.49733200	0.92043100	-1.17022200

1c-Int$_6$H$^+$$\subset$AuCav

C	3.57618100	-3.44220600	2.74224300
C	3.72382000	-3.27226600	1.23470200
C	2.39515200	-2.61676900	3.24116900
H	4.47049900	-3.01218000	3.19339500

C	2.89906900	-3.98215300	0.36034700
C	4.67145200	-2.41260900	0.66217300
C	1.12108300	-3.14485600	3.45241500
C	2.59005900	-1.25885600	3.52971400
C	2.98485600	-3.88884000	-1.02985000
H	2.15281200	-4.64430500	0.78456600
C	4.79352800	-2.28379000	-0.72182000
O	5.54754200	-1.70197700	1.48589900
C	0.07120700	-2.37816200	3.96578600
H	0.95147000	-4.19612800	3.25306400
C	1.57361100	-0.44471400	4.02228700
O	3.90498100	-0.82444300	3.46358000
C	2.08642000	-4.73867600	-1.91920200
C	3.93315500	-3.00044800	-1.55286900
H	5.53427700	-1.61570300	-1.14468100
C	5.33723700	-0.35193700	1.62212100
C	-1.31291100	-2.95392700	4.24280100
C	0.32741900	-1.02930800	4.23719500
H	1.76057600	0.59000900	4.27915100
C	4.39130600	0.11043000	2.60893700
H	2.49649600	-4.67566000	-2.92729700
C	0.68756400	-4.14095900	-1.98278500
O	4.03767400	-2.84074900	-2.94248200
C	-2.19336800	-2.76763900	3.01377900
H	-1.75139300	-2.34926900	5.03629800
O	-0.68699100	-0.32919600	4.89276300
C	-0.35946000	-4.54707500	-1.15491400
C	0.41188100	-3.14711500	-2.92992700
C	3.50297600	-1.68681700	-3.45730700
C	5.81138300	1.81091000	1.09412900
C	-2.12230400	-3.65573000	1.93772600
C	-3.08085200	-1.68926400	2.89855700
C	-1.35669500	0.70834600	4.33596500
C	4.81900100	2.26247600	2.01534600
C	-1.65725000	-4.04177100	-1.27562800
H	-0.17064100	-5.31765800	-0.41827300
C	-0.86057300	-2.60288800	-3.08628800
O	1.44095600	-2.84588600	-3.80961800
C	2.10402100	-1.65818700	-3.81576800
C	-2.91179000	-3.53407900	0.79398800
H	-1.42138400	-4.47943500	1.99686100
C	-3.88949800	-1.52166600	1.77317500
O	-3.20902900	-0.80477700	3.96915700
O	-2.70118600	0.46081900	3.86399100
C	-2.79186600	-4.50782100	-0.37238000
C	-1.88041200	-3.07101400	-2.26113800
H	-1.05526800	-1.87568000	-3.86419200
C	3.69779500	0.47572700	-4.15209300
C	-3.79242100	-2.44775500	0.73868700
H	-4.58727400	-0.69492200	1.72105000
C	-1.61911800	2.92771400	3.89908400
H	-3.71530900	-4.43442500	-0.95104500
O	-3.19513600	-2.68635800	-2.55397000
C	2.29601900	0.52809900	-4.41515600
O	-4.64910200	-2.33636700	-0.36509600
C	-2.94620900	2.68491000	3.43064800
C	4.58882800	3.64838400	2.16750000
C	6.55428800	2.75729400	0.35181600
C	1.72786300	1.71363700	-4.93343500
C	4.50041200	1.60793800	-4.41899800
C	-3.73708500	3.77062900	2.98856100
C	-1.12260800	4.25165200	3.93135600
C	-2.66698400	-5.96942700	0.07848600
H	-3.54650900	-6.25254400	0.66194900
H	-1.78834700	-6.15537900	0.70121800
C	2.09788200	-6.21806800	-1.50589400
H	1.46699900	-6.80413100	-2.17982300
H	1.73982800	-6.38103100	-0.48555900
C	3.51253000	-4.91829800	3.15981900
H	4.41881500	-5.43555300	2.83494200
H	2.66042100	-5.45160600	2.72881200
C	-1.28396500	-4.40847300	4.73358300
H	-0.66995700	-4.48545400	5.63455800
H	-0.87619300	-5.10526400	3.99612200
H	-2.29664800	-4.74197200	4.97478000
H	3.43893900	-4.99865600	4.24770800
H	3.11715700	-6.60843600	-1.55886900
H	-2.60168700	-6.62671200	-0.79233100
C	-1.91773100	5.29545900	3.50306100
C	-3.22816600	5.05429100	3.02619400
H	-3.83830300	5.88781500	2.69460700
H	-1.54225900	6.31317200	3.53816900
H	-4.74569300	3.56405100	2.64763200
H	-0.11728500	4.40876000	4.30696300
C	6.31559200	4.10567700	0.52287300
H	7.30683900	2.38899800	-0.33711900
C	5.32842000	4.55249800	1.43413100
H	3.83278400	3.96394600	2.87818900
C	3.92787300	2.74630600	-4.94704100
H	5.56347600	1.54065700	-4.21470800
C	2.53735200	2.79629000	-5.20993700
H	0.66361300	1.72331000	-5.14306700
H	2.10929300	3.69029700	-5.65326100
H	4.54796400	3.60682400	-5.17796700
H	5.16241200	5.61780300	1.56090000
H	6.89424000	4.83306700	-0.03772000
P	-4.14900900	-1.63518900	-1.75766800
N	-5.54606300	-1.83539800	-2.66133500
C	-5.42812100	-1.63278000	-4.11234100
C	-6.77109800	-1.25505400	-2.09233200
H	-4.54181800	-2.14071000	-4.49066100
H	-5.38474300	-0.56753200	-4.38337500
H	-6.30621500	-2.07580600	-4.58932200
H	-6.83989800	-1.49104600	-1.03077600
H	-7.62788500	-1.70716000	-2.59861100
H	-6.81994000	-0.16473900	-2.22946800
Au	-3.44627200	0.54182400	-1.45820600
N	-3.46097800	1.41452000	3.41236300
N	-0.83294300	1.89855200	4.33793500
N	4.11604900	1.36976700	2.77732300
N	6.03129500	0.47327600	0.89870300
N	4.27080900	-0.65295200	-3.63060500
N	1.51302400	-0.58007500	-4.23776900
C	-3.06864100	2.55178500	-1.21750900
C	-3.89458700	3.32516100	-0.52473500
H	-4.85873800	3.12281800	-0.08810000
C	-1.78547500	3.16714000	-1.72189600
C	-1.25823200	4.21393400	-0.73102200
H	-1.96169500	3.62347300	-2.70457500
H	-1.00665900	2.41811700	-1.86191700
C	-2.37539300	5.14038300	-0.31616200
O	-3.58219600	4.72648200	-0.23628400
O	-2.16327100	6.35271600	0.03994000
H	-1.16668700	6.53471200	-0.13517800
C	-0.81642300	3.54171800	0.66773300
H	-0.13777600	4.23898500	1.16183400
H	-1.71219400	3.49191300	1.28272300
C	-0.11584900	5.05500000	-1.27918100
O	0.16734600	6.15612100	-0.79288400
O	0.52980400	4.50748000	-2.27579500
C	1.71705300	5.19626800	-2.75396500
H	2.28068500	4.43796400	-3.28918400
H	1.41194600	6.01062200	-3.41320400
H	2.28096200	5.59120200	-1.90983400
C	-0.25664800	2.15155200	0.54602700
C	1.06636000	1.91561500	0.06511000
C	-1.06644000	1.07486200	0.86174700
C	1.99560200	2.95315200	-0.21139800
C	1.47881200	0.56194300	-0.16166000
C	-0.62870800	-0.25958600	0.70275900
H	-2.07070400	1.25604800	1.22287300
C	3.21997700	2.68276200	-0.77663700
H	1.75682200	3.97487800	0.06164400
C	2.75080900	0.31935900	-0.73057900
C	0.61674300	-0.51174800	0.17996900
H	-1.28502100	-1.07509800	0.97526200
C	3.59574100	1.35110600	-1.05865400
H	3.92447300	3.48547600	-0.96172700
H	3.04676700	-0.70541800	-0.89441500
H	0.95912100	-1.53100600	0.02554500

H	4.56446600	1.14024200	-1.49235100

TfO•[Au]

Au	0.10182100	-0.80067000	0.35631500
P	2.16684700	-0.00500100	0.10660800
O	2.81475800	0.51915800	1.48742900
O	3.15237200	-1.06964000	-0.59659400
C	4.46276000	-0.75172600	-1.11238500
H	5.21249000	-0.96740300	-0.34745100
H	4.62342200	-1.40100700	-1.97351700
H	4.52299900	0.29468400	-1.41840100
C	4.08510800	1.19939500	1.56790000
H	4.04349700	1.82628800	2.45884500
H	4.88543900	0.46342900	1.67677100
H	4.25812800	1.81866500	0.68495100
N	2.23248500	1.34721300	-0.91523600
C	1.79444500	1.07160100	-2.30003000
H	0.70721000	0.92872000	-2.36472800
H	2.08081800	1.92086400	-2.92638100
H	2.28864900	0.17812100	-2.68347200
C	1.56744300	2.55010300	-0.37950300
H	1.84956700	3.40493200	-1.00022600
H	0.47312900	2.45377700	-0.38935000
H	1.89266300	2.74244200	0.64334800
S	-2.75443600	-0.43668300	-0.65424400
O	-4.04522900	-1.04496300	-0.94782900
O	-1.93974800	-1.29101200	0.34954000
C	-3.11634100	1.06170500	0.38895100
O	-1.91650700	0.07615000	-1.75078100
F	-3.84930000	1.93417900	-0.30655000
F	-1.96118900	1.66778700	0.74457000
F	-3.77024500	0.72212300	1.50003500

(TfO•1a)[Au]

Au	1.94965100	-0.70481600	-0.26161600
P	2.62128800	1.46724500	-0.55862700
O	3.63978500	1.90232300	0.62291300
O	3.38558100	1.54687500	-1.98433300
C	3.76362100	2.77775400	-2.63578700
H	4.82679900	2.96073400	-2.46033600
H	3.58847100	2.63728800	-3.70325700
H	3.16916700	3.61667600	-2.26933800
C	4.30852900	3.18023200	0.63958300
H	4.55425800	3.38856400	1.68126600
H	5.22868200	3.11759700	0.05258000
H	3.66564500	3.97116300	0.24719100
N	1.50309800	2.71388500	-0.62891400
C	0.50557700	2.57919600	-1.71891300
H	-0.15827400	1.72078000	-1.57492800
H	-0.10936900	3.47919000	-1.72889900
H	1.01619400	2.47546700	-2.67831200
C	0.90710000	3.15698400	0.65001800
H	0.40011800	4.10733900	0.47511100
H	0.15406000	2.45784800	1.02332200
H	1.68909400	3.29540800	1.39748100
C	-1.37750100	-1.35304900	-0.75752500
O	-0.44452800	-0.76637500	-1.30351100
O	-2.57002800	-0.86525300	-0.56285200
H	-2.64278100	0.13967000	-0.83684200
C	-1.26253300	-2.78946200	-0.21701200
C	0.04551900	-3.46303200	-0.71007500
H	-0.00235400	-4.52959500	-0.45636200
H	0.07256400	-3.38808900	-1.80038100
C	1.33394100	-2.98352200	-0.19593900
C	2.49837800	-2.84585300	0.17704800
H	3.48197300	-2.96703100	0.57657700
C	-2.41310900	-3.59391200	-0.85393300
O	-3.21029300	-4.27384800	-0.25215600
O	-2.39012400	-3.46234400	-2.19357800
C	-3.45512100	-4.12599800	-2.89905300
H	-4.42039600	-3.73148300	-2.57517600
H	-3.28503100	-3.91167900	-3.95304900
H	-3.42529700	-5.20148300	-2.71026300
C	-1.46430900	-2.82029200	1.32251900
H	-2.43748800	-2.36290700	1.51603400
H	-1.54570400	-3.86872600	1.62048800
C	-0.38476800	-2.13030000	2.11948700
C	-0.26959400	-0.73154000	2.11474300
C	0.53224800	-2.87997100	2.86401500
C	0.77890100	-0.11033300	2.79687300
H	-1.00181500	-0.11265000	1.60551500
C	1.57599300	-2.25971500	3.55076400
H	0.43528300	-3.96236200	2.89249300
C	1.71238800	-0.87151500	3.50527700
H	0.84983800	0.97208000	2.78581900
H	2.27885100	-2.85869300	4.12276700
H	2.52447900	-0.38363200	4.03586600
S	-2.63424500	2.46763200	0.05759700
O	-1.99579400	1.78560900	1.20537100
O	-2.13506400	3.80775900	-0.28303400
C	-4.38400600	2.73122900	0.60781400
O	-2.79873900	1.56108700	-1.13802800
F	-4.95579500	1.55298700	0.89632000
F	-5.09556000	3.32842000	-0.35549300
F	-4.41268800	3.50114200	1.70326300

1a-TS1$_{5anti}$[Au]

Au	-1.82933000	0.15159800	-0.69115300
P	-4.06917400	-0.11695500	-0.26240400
O	-4.89890000	0.86741800	-1.24260000
O	-4.44496000	-1.65988900	-0.55786200
C	-5.77596300	-2.21161400	-0.49723100
H	-6.24987900	-2.11866800	-1.47717900
H	-5.66353700	-3.26664400	-0.24632800
H	-6.38046600	-1.71209500	0.26317800
C	-6.33729100	0.95969700	-1.29387100
H	-6.57500800	1.98737200	-1.57069300
H	-6.71427600	0.27959400	-2.06125900
H	-6.78555100	0.72305000	-0.32643200
N	-4.80667900	0.16231000	1.23970300
C	-4.58550600	-0.80063100	2.32342600
H	-3.72343500	-0.52885800	2.94422500
H	-5.47396900	-0.83175300	2.96332200
H	-4.41547200	-1.79808700	1.91820000
C	-4.88766600	1.54927700	1.70717100
H	-5.73889400	1.64860400	2.38858400
H	-3.97917000	1.85800700	2.24140800
H	-5.03843000	2.22661500	0.86601700
C	3.21824900	1.26054400	-0.68744700
O	2.89470700	0.96857000	-1.83359300
O	4.11409200	0.65263100	0.03665400
C	2.47758300	2.43714600	0.00126500
C	1.14914100	2.61236100	-0.78949100
H	0.38649100	3.07795800	-0.15650800
H	1.31267700	3.26976200	-1.64500500
C	0.66532700	1.31173900	-1.22422500
C	0.14512400	0.19036800	-1.41486600
H	0.55909000	-0.75761100	-1.75318600
C	3.30354100	3.71790000	-0.16870500
O	2.91838700	4.72030500	-0.73244200
O	4.50374300	3.59687800	0.41439500
C	5.36235100	4.74695300	0.31116300
H	4.90318200	5.60993200	0.79910200
H	6.28669100	4.46451100	0.81257100
H	5.54849400	4.98747000	-0.73769500
C	2.26520800	2.20053900	1.52733400
H	3.23492300	1.92465000	1.94381100
H	1.96969200	3.15483100	1.97750900
C	1.22325600	1.15530800	1.84674700
C	1.51940300	-0.20893900	1.74861400
C	-0.07248300	1.53953100	2.21456300
C	0.53672300	-1.17070800	1.97189100
H	2.51462200	-0.52411100	1.47113600
C	-1.05522400	0.57989700	2.45830400
H	-0.31014800	2.59550200	2.32073900
C	-0.75618300	-0.77979400	2.32530800
H	0.78867000	-2.21800900	1.85246300
H	-2.04672200	0.89647700	2.76579300
H	-1.52006200	-1.52953300	2.50941000

S	3.33722900	-2.59008000	-0.95892800
O	2.02741100	-1.90916700	-0.83127800
O	4.51563400	-1.66222400	-0.81590800
O	3.47775600	-3.58892600	-2.01794200
C	3.42580600	-3.54819300	0.62917400
F	3.49572900	-2.70582200	1.68391400
F	2.32054200	-4.29889800	0.78717000
F	4.49386600	-4.34951300	0.66611400
H	4.33368700	-0.28078300	-0.38599600

1a-Int$_{5anti}$ [Au]

Au	2.44186000	-0.27075700	0.91106200
P	4.65212700	-0.28995200	0.23234800
O	5.40760600	1.11405200	0.53423800
O	5.48480500	-1.50730600	0.90573200
C	6.83932000	-1.86230400	0.57062500
H	7.52746400	-1.35115600	1.24888400
H	6.92651600	-2.94045800	0.71293700
H	7.07262300	-1.60528200	-0.46510200
C	6.76003200	1.41195400	0.14086000
H	6.81775500	2.49342400	0.00974400
H	7.44715900	1.10706400	0.93449700
H	7.02133000	0.91096700	-0.79423600
N	4.96447800	-0.51536100	-1.43635000
C	4.53961600	-1.82486400	-1.95552000
H	3.44600000	-1.91520000	-2.01443800
H	4.95709500	-1.95705000	-2.95838400
H	4.91578800	-2.62576900	-1.31799400
C	4.49555000	0.58450800	-2.29209700
H	4.90442500	0.44614900	-3.29764800
H	3.39911500	0.62017800	-2.36195500
H	4.84694100	1.54211700	-1.90628100
C	-2.53345700	1.30585100	0.68440100
O	-1.79784000	0.46192200	1.38549400
O	-3.75959700	1.29092900	0.69482100
C	-1.65649600	2.25750600	-0.14173800
C	-0.26544000	1.99867600	0.46310000
H	0.53415600	2.00750500	-0.27742800
H	-0.05716000	2.76705400	1.21384400
C	-0.39642000	0.64474200	1.10765100
C	0.46237400	-0.32200100	1.39762100
H	0.03680100	-1.20913700	1.86401800
C	-2.13915400	3.68977900	0.10942000
O	-1.65403300	4.44384300	0.92457100
O	-3.18344200	4.00289000	-0.67134400
C	-3.75353200	5.30378100	-0.44362000
H	-3.00934600	6.08322700	-0.62228300
H	-4.57963500	5.38729500	-1.14835300
H	-4.11311900	5.38327500	0.58455000
C	-1.77559900	1.87840000	-1.65417700
H	-2.83528200	1.88987200	-1.91817700
H	-1.28908600	2.67328900	-2.22763000
C	-1.14747400	0.54544700	-1.97980800
C	-1.81619400	-0.64486300	-1.67198000
C	0.13139600	0.47323500	-2.54393200
C	-1.20462900	-1.87939300	-1.87714000
H	-2.81603400	-0.61575400	-1.25694500
C	0.73576400	-0.76206600	-2.77682300
H	0.65364800	1.39057300	-2.80567100
C	0.07575600	-1.94187600	-2.42921000
H	-1.73482100	-2.78362000	-1.59774400
H	1.71797200	-0.80362900	-3.23752900
H	0.55095400	-2.90356600	-2.59915800
S	-4.90727000	-1.97209700	1.00404500
O	-3.53491800	-2.13902400	0.53496400
O	-5.09346000	-0.60729100	1.78228000
O	-5.60312600	-3.01552100	1.73368100
C	-5.90200100	-1.61109700	-0.52349200
F	-5.40108600	-0.52312000	-1.13023800
F	-5.81951700	-2.65004600	-1.35650200
F	-7.17781600	-1.38829300	-0.21600000
H	-4.50379700	0.13345600	1.39344800

1a-TS2$_{5anti}$ [Au]

Au	-0.85380200	-0.46291700	-0.56378100
P	-2.83430100	-1.01557400	0.43576800
O	-2.80032900	-2.36832800	1.34035600
O	-3.32156900	0.21107100	1.36087300
C	-4.65180000	0.46638800	1.84498400
H	-4.68588000	0.24264500	2.91388200
H	-4.83922000	1.52975300	1.68936200
H	-5.39307900	-0.13145400	1.31084000
C	-3.92345600	-2.78388500	2.13984200
H	-3.74672100	-3.82706500	2.40390500
H	-3.97418300	-2.18196200	3.05109200
H	-4.85888100	-2.69569000	1.58153900
N	-4.17998000	-1.30630500	-0.57596200
C	-4.53887400	-0.17972900	-1.45098500
H	-3.84778200	-0.08320000	-2.30074400
H	-5.54954000	-0.34280600	-1.83750300
H	-4.52309500	0.75797100	-0.89720700
C	-4.12909400	-2.57019900	-1.32327800
H	-5.11462500	-2.75914200	-1.75941500
H	-3.38879900	-2.54143900	-2.13716800
H	-3.88002800	-3.39665500	-0.65737400
C	4.23176400	-0.49039700	-1.27386900
O	3.09098000	-0.31596700	-2.02525400
O	5.24134700	-0.93844300	-1.73915500
C	3.94496900	-0.08227500	0.18330200
C	2.59292200	0.62902700	0.06049200
H	1.90510000	0.46977200	0.88767300
H	2.76262200	1.70825500	-0.03113300
C	2.03993300	0.13559100	-1.24742000
C	0.78098500	0.18636800	-1.73931600
H	0.70667300	-0.11411800	-2.78499300
C	5.05876000	0.87745900	0.61154900
O	5.01481900	2.07817400	0.45994100
O	6.10233300	0.22417900	1.14252900
C	7.22685800	1.05092900	1.49389000
H	6.93207600	1.80095000	2.23108300
H	7.97052900	0.37098500	1.90713100
H	7.61440900	1.55592900	0.60654100
C	3.90682100	-1.36971000	1.06365300
H	4.83262500	-1.92198200	0.88831300
H	3.91026000	-1.05355500	2.11045400
C	2.69416900	-2.22525100	0.79076900
C	2.64590500	-3.07729500	-0.32051300
C	1.56881500	-2.13615300	1.61901800
C	1.48926500	-3.80067400	-0.61142900
H	3.51626100	-3.16987900	-0.96400900
C	0.41310500	-2.86217000	1.33589400
H	1.59432000	-1.48235300	2.48665100
C	0.36793100	-3.68988200	0.21178400
H	1.46609200	-4.45034400	-1.48112800
H	-0.45664000	-2.77337200	1.97574100
H	-0.53783300	-4.24337600	-0.01549100
S	-0.30739000	3.03006600	0.28150100
O	-0.20646500	1.86648600	1.17474700
O	0.19478300	2.70442200	-1.14379300
O	0.16276300	4.32956700	0.74132200
C	-2.13277200	3.25699900	-0.03046200
F	-2.61385100	2.27298900	-0.81604800
F	-2.80170500	3.23001200	1.12969400
F	-2.36006700	4.42200800	-0.63661400
H	0.36240100	1.53406400	-1.38142700

(TfO•2a)[Au]

Au	-1.01241100	0.62825800	-0.21251200
P	-0.10129000	2.59589500	0.29874600
O	1.49529000	2.50520100	0.21569300
O	-0.62665500	3.78159000	-0.66961900
C	-0.06991100	5.10874200	-0.60791100
H	0.88074800	5.13515700	-1.14722900
H	-0.78589000	5.76761800	-1.09958500
H	0.07783100	5.42762600	0.42707900
C	2.45821400	3.40181900	0.80835600
H	3.27705900	2.76714700	1.14446300
H	2.81261800	4.09302900	0.03966100
H	2.01908100	3.95778900	1.63860500

N	-0.40375400	3.19993300	1.85436200
C	-1.78056500	3.66189100	2.09084500
H	-2.49021800	2.82458100	2.15779000
H	-1.80296600	4.21306700	3.03535900
H	-2.10075100	4.32965100	1.29081400
C	0.06510100	2.31969100	2.94066500
H	0.12739600	2.90568700	3.86250000
H	-0.62077900	1.47700900	3.09890600
H	1.05121000	1.91491600	2.71169400
C	2.97361600	-0.30263300	0.65351800
O	2.09236000	-0.18349800	1.68356900
O	3.80617300	0.53526400	0.41657800
C	2.73932200	-1.64500600	-0.06580700
C	1.36872300	-2.08362400	0.46693300
H	0.57700200	-1.83991400	-0.24380100
H	1.32273900	-3.15035300	0.68006500
C	1.19328600	-1.26520300	1.71768700
C	0.36584700	-1.41380000	2.74260600
H	0.36553800	-0.71878100	3.57277500
C	3.83003000	-2.60348100	0.45293000
O	3.63490300	-3.51784500	1.22175300
O	5.03664900	-2.29080800	-0.04782400
C	6.12298200	-3.11507500	0.41015200
H	5.95360400	-4.15781200	0.13218000
H	7.01302100	-2.72322200	-0.08019600
H	6.21786000	-3.04838400	1.49626500
C	2.86435000	-1.49238300	-1.60637100
H	3.88025600	-1.15414700	-1.81556300
H	2.75618400	-2.49136800	-2.04195500
C	1.86851100	-0.54956300	-2.24102800
C	2.17656500	0.80973200	-2.38998400
C	0.63043500	-1.01140000	-2.70719200
C	1.26936900	1.68389500	-2.98641600
H	3.12744200	1.17815200	-2.02089900
C	-0.28485900	-0.13627000	-3.29496700
H	0.37000600	-2.06002500	-2.60014200
C	0.03421300	1.21605700	-3.43815800
H	1.52380600	2.73435500	-3.09189700
H	-1.24608900	-0.51177300	-3.63012600
H	-0.67715900	1.89892700	-3.89192700
H	-0.33957700	-2.23394000	2.74166900
S	-2.53327600	-1.97465400	0.61704500
O	-1.88830100	-3.23111200	1.00890100
O	-2.92236500	-1.01801400	1.66114300
O	-1.80065200	-1.29725900	-0.56162100
C	-4.10617600	-2.44431400	-0.25125600
F	-3.84542900	-3.25871400	-1.27844000
F	-4.71485700	-1.34422200	-0.71442100
F	-4.92471600	-3.06522600	0.60300500

1a-TS1$_{5syn}$[Au]

Au	-2.06708700	-1.43067200	-0.79545000
P	-3.52701100	0.25250800	-0.27528600
O	-3.11631800	1.57117300	-1.07865700
O	-5.05768100	-0.15169900	-0.62994300
C	-6.13320000	0.79823500	-0.49156400
H	-6.07484900	1.54728400	-1.28583400
H	-7.05850700	0.23088700	-0.59304100
H	-6.10044100	1.28821300	0.48484100
C	-3.48914200	2.93871700	-0.76047200
H	-2.55937000	3.49152000	-0.62958800
H	-4.06377000	3.32218100	-1.60598000
H	-4.08375300	2.97786000	0.15392100
N	-3.61382500	0.72500400	1.34573800
C	-4.20921500	-0.26664600	2.25135300
H	-3.51948400	-1.09689400	2.46519200
H	-4.45252500	0.22860000	3.19582000
H	-5.12633600	-0.67476500	1.82475300
C	-2.36706000	1.29767400	1.89607100
H	-2.59926200	1.76161400	2.85840300
H	-1.59521800	0.53411300	2.04726800
H	-1.95958800	2.06467500	1.24194100
C	1.52163900	-0.79375700	0.91397500
O	0.44071200	-0.73142000	0.32743300
O	1.95560100	0.03314600	1.81001600
C	2.44878700	-1.96655500	0.55897400
C	1.98039100	-2.42260700	-0.84050100
H	2.31609800	-1.69024700	-1.58155600
H	2.43275800	-3.38974900	-1.09412800
C	0.53918700	-2.59759600	-0.94352600
C	-0.63574400	-2.94607100	-1.22757800
H	-1.01268300	-3.84360000	-1.70032300
C	2.17381000	-3.07449300	1.58083400
O	1.42718300	-2.98251000	2.52803400
O	2.86813700	-4.19072200	1.27663100
C	2.67203000	-5.29677200	2.17733300
H	1.62152100	-5.59604100	2.18782300
H	3.30007500	-6.10044400	1.79518600
H	2.97176900	-5.01881100	3.18994100
C	3.95770600	-1.58340400	0.57511000
H	4.16262000	-1.14656200	1.55596300
H	4.53563600	-2.50743700	0.49518700
C	4.35445500	-0.62885900	-0.52902700
C	4.08791500	0.74224800	-0.43253000
C	4.96073300	-1.11728600	-1.69513400
C	4.39721700	1.60204600	-1.48494700
H	3.62351300	1.13979700	0.45877900
C	5.28297000	-0.25709000	-2.74495100
H	5.18428400	-2.17869500	-1.77724100
C	4.99527200	1.10533900	-2.64320600
H	4.15199900	2.65450400	-1.39471000
H	5.75588000	-0.65044600	-3.64012400
H	5.23538000	1.77491800	-3.46361500
S	0.67553300	2.86265900	0.41874000
O	1.15044600	2.02417700	-0.69041200
O	0.67317800	2.16368600	1.76177700
O	-0.53825700	3.66928600	0.20414000
C	2.03100100	4.10964100	0.64762500
F	3.16456700	3.48939600	1.03388800
F	2.28015700	4.74227000	-0.50820100
F	1.71126300	5.01629700	1.57578200
H	1.36708800	0.90620100	1.82853300

1a-Int$_{5syn}$[Au]

Au	1.31577400	-1.64147200	-0.35638700
P	3.27476400	-0.44321900	-0.14048700
O	3.12040800	0.79796800	0.88237700
O	4.52974800	-1.34277300	0.37999200
C	5.78768800	-0.73762600	0.72302700
H	5.68197900	-0.13569100	1.63031600
H	6.48423000	-1.55550200	0.90999600
H	6.16084100	-0.11197200	-0.09275400
C	3.84113700	2.04393400	0.84916200
H	3.12765900	2.83923000	0.62432400
H	4.27533100	2.20020400	1.83920200
H	4.62562600	2.03030100	0.09041500
N	3.91021500	0.27587200	-1.55466400
C	4.48769700	-0.67128900	-2.51763000
H	3.71633400	-1.26154800	-3.03531800
H	5.04985600	-0.10601500	-3.26745200
H	5.16924400	-1.35821900	-2.01565900
C	2.99366500	1.21976300	-2.21475000
H	3.55756800	1.79623600	-2.95433600
H	2.16925100	0.70265200	-2.72718600
H	2.56477600	1.91766700	-1.49909400
C	-2.73300400	0.04467900	-0.66069700
O	-1.60537800	-0.52631200	-1.02640000
O	-2.92808600	1.25748900	-0.73657100
C	-3.71527100	-0.97513200	-0.08755600
C	-3.08606200	-2.30282900	-0.59058100
H	-3.22604200	-3.12654700	0.11004400
H	-3.53050800	-2.57534300	-1.55368200
C	-1.62981500	-1.94425700	-0.74401900
C	-0.48869100	-2.59020400	-0.55966000
H	-0.60768900	-3.64857000	-0.33133200
C	-5.11231600	-0.73666800	-0.65151000
O	-6.12627800	-0.66020100	0.00344500
O	-5.07208500	-0.64484800	-1.99500700
C	-6.33741600	-0.40620100	-2.63654900
H	-6.77063800	0.53069500	-2.27980200

H	-6.11900500	-0.34944600	-3.70198500
H	-7.03132700	-1.22318900	-2.42591800
C	-3.74579300	-0.83527900	1.46418400
H	-4.08442500	0.17941500	1.69277300
H	-4.51867900	-1.51747500	1.82631000
C	-2.41391600	-1.12336900	2.11477800
C	-1.40817600	-0.14994500	2.13193100
C	-2.13285900	-2.39073500	2.63967500
C	-0.13088600	-0.45238500	2.59913500
H	-1.60998300	0.84279100	1.74789900
C	-0.86276300	-2.69257400	3.12858200
H	-2.91287900	-3.14804700	2.65510000
C	0.14652800	-1.72864600	3.09273300
H	0.64770300	0.30069900	2.54970500
H	-0.65763800	-3.68188100	3.52693400
H	1.14334200	-1.96922000	3.44952000
S	0.03392000	2.97214400	-0.37994400
O	0.32252000	1.68197700	0.23467700
O	-1.12810300	2.87574700	-1.43488000
O	1.10834100	3.78826900	-0.93163200
C	-0.77910200	3.98568100	0.94803800
F	-1.89055500	3.36235300	1.36155600
F	0.06575300	4.10470200	1.97487000
F	-1.09769000	5.19396500	0.49087500
H	-1.83693900	2.15874600	-1.16788100

1a-TS2$_{5syn}$[Au]

Au	0.78350900	-0.38734600	-0.33071700
P	2.55481400	-1.64895100	0.34701300
O	2.27633900	-3.24937300	0.30999500
O	3.89265800	-1.32817300	-0.50060400
C	5.23053900	-1.70576100	-0.11685100
H	5.52650100	-2.59218200	-0.68331400
H	5.88164100	-0.87088700	-0.37951900
H	5.29378400	-1.90304600	0.95501900
C	3.23382800	-4.22601200	0.76422500
H	2.66936700	-5.12973800	0.99669200
H	3.94949900	-4.43996800	-0.03394200
H	3.76017500	-3.87750100	1.65577300
N	3.03216300	-1.38224500	1.95949200
C	3.46739900	0.00456500	2.22409700
H	2.62672100	0.71073400	2.22120500
H	3.95334400	0.03192300	3.20345300
H	4.18386200	0.33008000	1.47067500
C	2.05137300	-1.83114200	2.96224500
H	2.52477400	-1.80267200	3.94773600
H	1.15931900	-1.18860600	2.98547500
H	1.73770800	-2.85555000	2.75817600
C	-3.41277200	-0.30970100	1.26803500
O	-2.14103600	0.17205500	1.04832600
O	-3.73206500	-0.82719700	2.30030900
C	-4.23770300	-0.15068900	-0.01651800
C	-3.38148800	0.85015100	-0.81865500
H	-3.38003300	0.67017000	-1.89353400
H	-3.72113200	1.87523300	-0.63611300
C	-2.00781300	0.68638600	-0.22559000
C	-0.79150600	0.99029800	-0.72601200
H	-0.82317800	1.42317900	-1.72441900
C	-5.62445000	0.38651800	0.32679700
O	-6.66893700	-0.13611300	0.01330300
O	-5.52548200	1.53416700	1.02055100
C	-6.77400500	2.12674900	1.42512100
H	-7.32835300	1.43549700	2.06325700
H	-6.50449800	3.02813500	1.97316600
H	-7.37942300	2.37080400	0.54939200
C	-4.36562900	-1.54284300	-0.69866400
H	-4.89435200	-2.19873500	-0.00208400
H	-5.01214100	-1.41623000	-1.57056700
C	-3.03271300	-2.13325700	-1.09386600
C	-2.24231000	-2.80502900	-0.14986900
C	-2.53484400	-1.97435800	-2.39298900
C	-0.97244900	-3.26764700	-0.48652800
H	-2.61918800	-2.94804500	0.85856800
C	-1.26717000	-2.44402000	-2.73723500
H	-3.14653600	-1.47782800	-3.14178800
C	-0.47650200	-3.08075700	-1.78010100
H	-0.35680800	-3.76157100	0.25722800
H	-0.89491600	-2.30519500	-3.74756400
H	0.51775000	-3.43380800	-2.03243400
S	1.17911700	3.77559200	0.05741700
O	0.32554800	4.14359700	-1.07462500
O	0.61512900	2.57173500	0.84186500
O	1.70342400	4.79993800	0.95475400
C	2.68487500	3.01796500	-0.73083000
F	2.33367400	2.04929800	-1.60044300
F	3.37656800	3.94691900	-1.39377400
F	3.48758700	2.46672000	0.19716500
H	-0.05933400	1.76657500	0.11738200

1a-TS1$_6$[Au]

Au	-1.78749500	0.46676700	-0.57980500
P	-3.93381100	-0.32997300	-0.33848100
O	-4.90938700	0.60814900	-1.22658700
O	-3.98865700	-1.85724800	-0.85785800
C	-5.19353700	-2.64017400	-0.98335800
H	-5.63112600	-2.47364400	-1.97047200
H	-4.89524900	-3.68446200	-0.88890500
H	-5.91584300	-2.38889300	-0.20329900
C	-6.34124000	0.46128100	-1.31622100
H	-6.75110300	1.46267000	-1.45278000
H	-6.58569500	-0.15369600	-2.18547300
H	-6.75122200	0.01273200	-0.40876700
N	-4.75682500	-0.40485900	1.14235700
C	-4.42835000	-1.47437100	2.09020100
H	-3.65868800	-1.16174300	2.80517800
H	-5.32918400	-1.74790200	2.65028000
H	-4.06596800	-2.35580300	1.56163700
C	-5.05541500	0.87216200	1.79804700
H	-5.92049200	0.74364200	2.45649300
H	-4.21057100	1.23156000	2.40142700
H	-5.29823600	1.63411700	1.05630300
C	2.92826000	1.36729400	-0.67054400
O	2.67003500	1.06270300	-1.83933100
O	3.87245700	0.81685300	0.03626600
C	2.10783900	2.45400900	0.06273500
C	0.70328500	2.61618800	-0.58447700
H	0.05749500	3.16547400	0.10076000
H	0.78919900	3.21777300	-1.49146100
C	0.12320800	1.29168800	-0.96905300
C	0.63529400	0.31750200	-1.58067100
H	0.95290600	-0.67695500	-1.85697100
C	2.83684300	3.79937800	-0.09109600
O	2.31851800	4.82198600	-0.48506900
O	4.11268100	3.71169100	0.31174900
C	4.87236400	4.93078900	0.23286800
H	4.42947900	5.69747500	0.87300200
H	5.87315000	4.67137800	0.57466500
H	4.89673700	5.29642800	-0.79594000
C	2.00332600	2.16511400	1.59648400
H	3.01076700	1.96834600	1.96440900
H	1.64312800	3.08067900	2.07855000
C	1.07121100	1.02532700	1.93470800
C	1.46905300	-0.30593300	1.76447700
C	-0.22996800	1.28459400	2.38423200
C	0.58189800	-1.35405900	1.99960000
H	2.47044400	-0.52837500	1.42733200
C	-1.11674600	0.23624100	2.64142500
H	-0.54920200	2.31217500	2.53937000
C	-0.71555100	-1.08679400	2.43911900
H	0.91470100	-2.37188200	1.82976300
H	-2.11183500	0.45854400	3.01540200
H	-1.40270800	-1.90468500	2.63418400
S	3.51459000	-2.47813800	-1.04840100
O	2.12347700	-1.97187300	-0.93508000
O	4.55802900	-1.40464100	-0.89079400
O	3.79008500	-3.44267900	-2.11202900
C	3.70367700	-3.42614000	0.53656300
F	3.67171800	-2.58626900	1.59388100
F	2.69350900	-4.30033800	0.68394500
F	4.85833000	-4.09630100	0.57191500

H	4.20012200	-0.06363700	-0.42296800

1a-Int$_6$[Au]

Au	-2.41306700	0.35076700	-0.61624700
P	-4.61618600	-0.28673300	-0.32207800
O	-5.62271300	0.98186000	-0.22716200
O	-5.10329000	-1.27769600	-1.50870600
C	-6.35958600	-1.98148900	-1.53446600
H	-7.08975700	-1.39804900	-2.10114100
H	-6.17970800	-2.92888400	-2.04473300
H	-6.72991700	-2.16892100	-0.52420600
C	-7.04264700	0.87515600	-0.01391700
H	-7.35858000	1.80458000	0.46169300
H	-7.55045400	0.77046400	-0.97626300
H	-7.28510300	0.02782400	0.63183500
N	-5.01875800	-1.17665300	1.08223300
C	-4.34729500	-2.48315700	1.17857100
H	-3.27759600	-2.38523600	1.40874600
H	-4.82660800	-3.06631100	1.97079600
H	-4.44762000	-3.03038500	0.24038100
C	-4.92188700	-0.42209800	2.33929000
H	-5.39034900	-1.00683600	3.13679100
H	-3.88014100	-0.21715200	2.62369900
H	-5.44811500	0.52908000	2.25423700
C	2.41640700	1.12670300	-0.78012900
O	1.80371700	0.22074800	-1.51384600
O	3.64035700	1.24277500	-0.86682900
C	1.62248300	1.99691900	0.19385800
C	0.14048600	2.11729200	-0.23384600
H	-0.43764300	2.45589800	0.63107100
H	0.06144600	2.90910900	-0.98691900
C	-0.43141100	0.84063200	-0.80271400
C	0.39994100	-0.00448700	-1.40434000
H	0.17661500	-0.95119300	-1.87193100
C	2.27997000	3.38820300	0.10144300
O	2.03024400	4.18523000	-0.77633400
O	3.15944400	3.61759800	1.08724800
C	3.86321700	4.86867000	0.99805800
H	3.16031600	5.70461600	1.01280800
H	4.51905200	4.89723400	1.86727000
H	4.44379000	4.90717800	0.07381500
C	1.78799700	1.39436700	1.62864500
H	2.85596500	1.26830600	1.81893800
H	1.42578000	2.15387900	2.32742300
C	1.05396000	0.09629100	1.85896700
C	1.61417100	-1.11748000	1.44480000
C	-0.20399300	0.08380700	2.47067700
C	0.91433900	-2.31246200	1.59593800
H	2.59631800	-1.13657100	0.98875400
C	-0.89739000	-1.11406300	2.64592700
H	-0.64351400	1.01786100	2.81077700
C	-0.34623300	-2.31511900	2.19701800
H	1.36382700	-3.23757300	1.24900200
H	-1.86202200	-1.11088700	3.14447900
H	-0.88496600	-3.24871800	2.33294900
S	5.02649100	-1.89945000	-1.14297500
O	3.64074000	-2.22129700	-0.81193300
O	5.14099000	-0.52447900	-1.91073800
O	5.90062700	-2.86136800	-1.78882100
C	5.81663300	-1.44393900	0.47712900
F	5.11789800	-0.45032500	1.05100400
F	5.80172500	-2.50573700	1.28489100
F	7.07241600	-1.04177000	0.29494700
H	4.46724700	0.16993000	-1.55969900

1a-TS2$_6$[Au]

Au	0.53107600	-0.33861800	0.07791200
P	2.57008400	-1.19619200	-0.49438100
O	2.49660600	-2.33677300	-1.65066300
O	3.51664600	-0.00096400	-1.00696200
C	4.95157300	0.00045500	-1.11027500
H	5.22681500	-0.05864900	-2.16578500
H	5.28910000	0.95221000	-0.69845100
H	5.39256100	-0.83159400	-0.55762900
C	3.68127200	-2.85381300	-2.28627200
H	3.38195500	-3.76612400	-2.80299700
H	4.05779400	-2.13046600	-3.01398400
H	4.45572800	-3.08237500	-1.54941500
N	3.51090000	-1.95909500	0.70760500
C	3.82656500	-1.10539200	1.86296800
H	2.97232200	-1.01279700	2.54940600
H	4.66704800	-1.54520400	2.40850100
H	4.10552400	-0.10404000	1.53682600
C	3.04012400	-3.28524300	1.12793900
H	3.82613000	-3.76387200	1.71988800
H	2.12773500	-3.22715000	1.73939800
H	2.83287600	-3.90780500	0.25737300
C	-4.03200700	0.00661800	1.34822700
O	-3.00606300	0.00595600	2.26417500
O	-5.14720800	-0.19556300	1.75255600
C	-3.69671200	0.19053600	-0.13799000
C	-2.35882300	0.93341200	-0.36513800
H	-1.99724500	0.71859200	-1.37496500
H	-2.55630100	2.01000900	-0.33459400
C	-1.29329300	0.62803000	0.66692600
C	-1.70460200	0.22043900	1.88650000
H	-1.04987600	0.04977600	2.73334200
C	-4.83698600	1.08649700	-0.66815800
O	-4.88363000	2.27974500	-0.46939200
O	-5.76332400	0.40866200	-1.36155100
C	-6.87687300	1.19724300	-1.81925000
H	-6.53030500	2.00530400	-2.46706300
H	-7.51743500	0.50714400	-2.36689400
H	-7.40777100	1.62614000	-0.96677200
C	-3.73726900	-1.21852100	-0.80705500
H	-4.72271800	-1.64741300	-0.61359400
H	-3.66638300	-1.05690000	-1.88619700
C	-2.66010900	-2.18010600	-0.36761900
C	-2.75623500	-2.85674600	0.85584300
C	-1.55052600	-2.42951600	-1.18410800
C	-1.75710100	-3.74034400	1.26215200
H	-3.62318100	-2.69854800	1.49061100
C	-0.55649000	-3.32889800	-0.79021400
H	-1.46561500	-1.92014800	-2.14010800
C	-0.65511100	-3.98088000	0.43885400
H	-1.85027800	-4.25606200	2.21335600
H	0.29634800	-3.50061900	-1.43700300
H	0.11381700	-4.68245500	0.74821100
S	0.97845100	3.23120400	-0.32914000
O	0.92671500	2.21552900	-1.39115300
O	0.03186800	2.88460700	0.83696300
O	0.93499100	4.64324700	-0.68552400
C	2.63678400	3.00300400	0.49566900
F	2.67684500	1.84821900	1.19566900
F	3.61034100	2.96340800	-0.42450500
F	2.88106000	4.00490500	1.34082900
H	-0.44853300	1.72619200	0.78268600

(TfO•3a)[Au]

Au	-1.16763300	0.28647700	-0.05728100
P	-0.99985900	2.50570500	0.02336800
O	-0.73736800	3.03987900	1.52529000
O	0.19308200	3.01447800	-0.94447900
C	0.52468100	4.40140700	-1.15873400
H	1.22878400	4.73168800	-0.39106200
H	1.00228500	4.45711800	-2.13723700
H	-0.37006000	5.02770400	-1.14453900
C	-0.72948300	4.42913600	1.91338800
H	-1.12559800	4.47193200	2.92860700
H	0.29905700	4.79779200	1.90794200
H	-1.35055900	5.02993100	1.24597400
N	-2.33887900	3.42168400	-0.48487700
C	-2.66099700	3.27181400	-1.91416800
H	-3.07343800	2.27825800	-2.14227100
H	-3.40197300	4.02907100	-2.18443900
H	-1.77002700	3.42600600	-2.52366800
C	-3.53521300	3.23628500	0.35710200
H	-4.27440900	3.99331800	0.08168300
H	-3.98163400	2.24026400	0.22614200

H	-3.28378900	3.36519900	1.41035500
C	3.77088300	-1.84374100	-0.78460600
O	2.56766700	-2.26209900	-1.25589000
O	4.65458600	-2.65341300	-0.64466800
C	3.93214000	-0.36909100	-0.36236000
C	2.99376400	0.59730700	-1.12255000
H	2.78229400	1.46410300	-0.48479800
H	3.52495200	0.97587900	-2.00258700
C	1.71200400	-0.04688800	-1.53937900
C	1.55624000	-1.36617100	-1.56623800
H	0.64487900	-1.89226600	-1.81485000
C	5.38926000	-0.02222300	-0.73193000
O	5.72166600	0.40015000	-1.81905200
O	6.25089700	-0.24754500	0.26880400
C	7.63641400	-0.05480300	-0.06160800
H	7.81238200	0.97081100	-0.39399400
H	8.18702500	-0.26623600	0.85415100
H	7.93055600	-0.74354200	-0.85649800
C	3.73625100	-0.31035700	1.18771400
H	4.46340700	-0.99279200	1.63139200
H	4.01656600	0.70029600	1.50181200
C	2.35381800	-0.63063400	1.70539300
C	1.91264200	-1.95878400	1.80435200
C	1.50616400	0.38893700	2.15066500
C	0.64913500	-2.25658000	2.31025900
H	2.56391300	-2.76601900	1.48326100
C	0.24790300	0.09290800	2.68375300
H	1.83447200	1.42367600	2.09254400
C	-0.18508300	-1.23226100	2.76305900
H	0.30754000	-3.28571600	2.34663600
H	-0.38954200	0.89826100	3.03268400
H	-1.17447200	-1.46074600	3.14192600
H	0.88745100	0.58238700	-1.85029600
S	-2.61246800	-2.43655700	0.38432500
O	-1.42439200	-1.78320200	-0.35308900
O	-2.34605100	-3.83857200	0.69101300
O	-3.17339000	-1.55944700	1.42361700
C	-3.87596400	-2.42208500	-0.97928900
F	-4.11048000	-1.15171300	-1.36517700
F	-5.02354200	-2.95532500	-0.55373900
F	-3.43692100	-3.10933800	-2.03670400

1a[Au]

Au	-0.86659000	-0.96903700	-0.34822900
P	-3.00038600	-0.28623900	0.14021500
O	-3.48636800	0.84564800	-0.88506300
O	-3.93859100	-1.58543500	0.05638900
C	-5.37351000	-1.56375500	0.27322400
H	-5.87338000	-1.28321700	-0.65584700
H	-5.65214400	-2.57995800	0.54862300
H	-5.63768000	-0.87171900	1.07520700
C	-4.73653600	1.57523200	-0.78830800
H	-4.52396300	2.59575600	-1.10693400
H	-5.46008400	1.12001700	-1.46643200
H	-5.11485200	1.57425300	0.23508800
N	-3.30230600	0.35714300	1.66573000
C	-3.20624300	-0.57164100	2.80416600
H	-2.16303200	-0.74049900	3.10592200
H	-3.74611100	-0.14027800	3.65107400
H	-3.65873900	-1.53131700	2.55587300
C	-2.70031800	1.66806800	1.95731700
H	-3.23144900	2.11523500	2.80155400
H	-1.63618800	1.58181400	2.21666700
H	-2.78640400	2.32963300	1.09676500
C	3.91222600	1.18924500	1.19454400
O	3.41972000	1.38469500	2.28300000
O	5.02013200	1.80355700	0.74818200
H	5.34526000	2.37984800	1.46093500
C	3.33349700	0.25545300	0.12868100
C	2.18566900	-0.53966600	0.80795500
H	2.59093600	-1.15522300	1.61693000
H	1.50048700	0.17467800	1.26979500
C	1.47052100	-1.39888000	-0.13382100
C	0.96854000	-2.15560500	-0.96153900
H	0.78743400	-2.92049200	-1.68968700
C	4.41210800	-0.75621000	-0.30816400
O	4.56760000	-1.14635500	-1.44153500
O	5.09694600	-1.19366000	0.75644600
C	6.12833700	-2.17031700	0.48441900
H	6.86935200	-1.74862800	-0.19675200
H	6.57328900	-2.39803900	1.45108700
H	5.69043600	-3.06384000	0.03547700
C	2.86207600	1.05325200	-1.11724200
H	3.64834700	1.77495200	-1.35907100
H	2.83464900	0.34791700	-1.94945800
C	1.52516900	1.75094800	-0.99752600
C	1.25687300	2.70541100	-0.00576400
C	0.52298200	1.47003900	-1.93885200
C	0.02714400	3.36488600	0.03434000
H	2.00955600	2.95274200	0.73469000
C	-0.70558000	2.13389800	-1.90641700
H	0.72253100	0.74640500	-2.72429900
C	-0.95423000	3.08608600	-0.91826400
H	-0.15313200	4.11340000	0.79998700
H	-1.46144900	1.90683600	-2.65021700
H	-1.89897300	3.62093700	-0.90208300

1a-TS1$_{5anti}$H^{+}[Au]

Au	-1.13394500	-0.09105700	-0.80649300
P	-3.19613600	0.57291700	-0.03672500
O	-3.19306700	2.17230900	0.14127500
O	-4.27432300	0.10306800	-1.13161300
C	-5.70814400	0.28874800	-1.02700900
H	-5.98274000	1.21163300	-1.54110000
H	-6.16647900	-0.56228600	-1.53009700
H	-6.02583100	0.32273300	0.01665200
C	-4.36032500	2.95420600	0.49842700
H	-3.98827400	3.85692700	0.98194100
H	-4.90313400	3.22110800	-0.41037500
H	-5.00906200	2.40424600	1.18303700
N	-3.83635200	0.01061900	1.42088400
C	-4.17244800	-1.41790200	1.49724800
H	-3.29788100	-2.03283900	1.74943700
H	-4.93123200	-1.56241900	2.27151000
H	-4.57780000	-1.76700700	0.54739300
C	-3.27310200	0.53586600	2.67079700
H	-4.01622200	0.42759900	3.46599800
H	-2.36492800	-0.00355100	2.97312100
H	-3.02968600	1.59299200	2.56659300
C	4.25247000	-0.61459200	-1.01481000
O	3.55184400	-1.05306400	-1.90962600
O	5.50633700	-0.98991900	-0.84477100
C	3.64190900	0.35689000	0.01662200
C	2.35248400	0.92492200	-0.61388000
H	1.73177400	1.39203100	0.15640700
H	2.60735000	1.70823300	-1.33461100
C	1.55107700	-0.09402100	-1.29035000
C	0.63410200	-0.78549300	-1.78153500
H	0.52505500	-1.60318100	-2.47477400
C	4.64633900	1.44432200	0.38315000
O	5.83003700	1.19912100	0.56650300
O	4.09642800	2.63792200	0.55065400
C	4.99527300	3.70267200	0.96495800
H	5.77633800	3.83784500	0.21580900
H	4.36922400	4.58862000	1.04396200
H	5.44676000	3.45088700	1.92551600
C	3.37982500	-0.41513100	1.36999400
H	4.31389500	-0.91058400	1.64868400
H	3.17671700	0.34147700	2.13428800
C	2.23598600	-1.39346500	1.31403400
C	2.37833600	-2.63731200	0.68494600
C	0.99772800	-1.05143600	1.86952900
C	1.29139800	-3.50139400	0.57316000
H	3.33959700	-2.93011900	0.27429000
C	-0.08825200	-1.92153800	1.77002600
H	0.88490600	-0.10182600	2.38657000
C	0.05142300	-3.14225300	1.10724900
H	1.41414000	-4.45983100	0.07930500
H	-1.03583700	-1.65526200	2.22532000
H	-0.79198200	-3.82113300	1.02922500

H	5.93899800	-0.37489500	-0.20119300

1a-Int$_{5anti}$H^{+}[Au]

Au	1.22678700	-0.02686300	-0.86938500
P	3.29648300	-0.58512100	0.00936200
O	3.35235300	-2.15431300	0.38718800
O	4.44548300	-0.21482500	-1.05866100
C	5.86893200	-0.32285500	-0.82704700
H	6.21800100	-1.28893400	-1.19749800
H	6.33849200	0.47805300	-1.39843200
H	6.10581100	-0.21354700	0.23304000
C	4.52197700	-2.82171900	0.91371200
H	4.16001200	-3.68133100	1.47779100
H	5.14553600	-3.16543000	0.08553900
H	5.09300600	-2.16050100	1.56898900
N	3.83999800	0.16934300	1.43436400
C	4.06117000	1.61873600	1.33688800
H	3.11821400	2.18443300	1.36849300
H	4.68575800	1.93915600	2.17559800
H	4.57790200	1.86474500	0.40882100
C	3.14556900	-0.20458100	2.67179000
H	3.75316400	0.10676100	3.52631800
H	2.15933900	0.27639000	2.75671000
H	3.00760900	-1.28471900	2.72162200
C	-3.98758100	0.34714700	-1.14058000
O	-2.99431900	0.67064500	-1.86242200
O	-5.17923900	0.69036200	-1.46167900
C	-3.62772800	-0.44465600	0.09330600
C	-2.22917000	-0.95942200	-0.29643000
H	-1.55465400	-1.03888400	0.55439900
H	-2.30342700	-1.94569500	-0.76822700
C	-1.73682900	0.07437000	-1.27678900
C	-0.57445700	0.55314500	-1.66529500
H	-0.60142200	1.33611200	-2.42106600
C	-4.73069900	-1.44997700	0.36901700
O	-5.90371800	-1.19125500	0.10252100
O	-4.31605500	-2.54330400	0.96465700
C	-5.33780800	-3.51797600	1.32743200
H	-5.85010000	-3.86112500	0.42806200
H	-4.79480000	-4.32918500	1.80604400
H	-6.05246100	-3.05999200	2.01214900
C	-3.60390200	0.53242600	1.34871500
H	-4.59393700	0.99040400	1.43540900
H	-3.46148800	-0.11712700	2.21689200
C	-2.51555300	1.56952400	1.27694400
C	-2.70115000	2.74618600	0.54003400
C	-1.28320900	1.34264000	1.90074600
C	-1.65845700	3.65815300	0.39142900
H	-3.66445500	2.95312700	0.08016800
C	-0.24198300	2.25738900	1.75848900
H	-1.13829400	0.44629500	2.49788100
C	-0.42248400	3.40922100	0.99172100
H	-1.81196300	4.56489400	-0.18440500
H	0.70762000	2.07489900	2.25036300
H	0.38905000	4.12058600	0.87743900
H	-5.79182300	0.13919300	-0.86896100

1a-TS1$_{5syn}$H^{+}[Au]

Au	-1.16012600	0.34252500	-0.99280800
P	-3.07648100	-0.32098000	0.07777600
O	-3.35885900	0.63630500	1.34882700
O	-4.33926900	-0.32920800	-0.91673000
C	-5.66640400	-0.79100800	-0.56555000
H	-6.25910800	0.05864100	-0.22076800
H	-6.10319900	-1.19573100	-1.47830200
H	-5.62406800	-1.56444300	0.20365400
C	-4.44031200	0.43342500	2.29016900
H	-4.11338100	0.86979700	3.23403700
H	-5.33065400	0.95613800	1.93488800
H	-4.65008200	-0.62960300	2.42318500
N	-3.07115000	-1.87567600	0.75292500
C	-3.04425600	-2.98490800	-0.21543100
H	-2.04769000	-3.11899900	-0.65938700
H	-3.32198800	-3.90686200	0.30250300
H	-3.76178800	-2.80990700	-1.01717200
C	-2.10003700	-2.07619500	1.84194700
H	-2.34487500	-3.00718900	2.36008200
H	-1.06884300	-2.14355600	1.46692000
H	-2.16070600	-1.25816000	2.56061700
C	2.41164900	-1.32402300	0.38341600
O	1.37272200	-1.25592900	-0.26923500
O	2.57259700	-2.17277600	1.36719100
C	3.51658900	-0.31477800	0.05963300
C	3.19233600	0.15934200	-1.37087800
H	3.67889700	1.11796000	-1.58097500
H	3.57013500	-0.57078700	-2.09412800
C	1.74859700	0.35485200	-1.54836100
C	0.64892100	0.86692100	-1.91146300
H	0.59858900	1.70763900	-2.60014000
C	4.88857500	-0.95820000	0.20728100
O	5.12706200	-1.76709300	1.09629500
O	5.77757300	-0.50469500	-0.65551400
C	7.13170200	-1.02229800	-0.52228500
H	7.12562800	-2.10440100	-0.65782600
H	7.70105700	-0.53011900	-1.30720200
H	7.52292500	-0.77461600	0.46520200
C	3.48027400	0.84003300	1.14114400
H	3.71404300	0.38032300	2.10604700
H	4.30377500	1.51733700	0.89575700
C	2.17744400	1.59428400	1.20616400
C	1.07722400	1.05615400	1.88621500
C	2.03913000	2.82627500	0.55686600
C	-0.15241100	1.71040200	1.86866200
H	1.17850600	0.11649300	2.42210500
C	0.81212100	3.48790200	0.54391800
H	2.89582300	3.26742000	0.05386300
C	-0.29131800	2.92192300	1.18500100
H	-1.00654400	1.28149600	2.38006000
H	0.71720900	4.44260800	0.03655900
H	-1.25160300	3.42664900	1.16921100

1a-Int$_{5syn}$H^{+}[Au]

Au	-1.18775600	-0.19277200	-0.83858900
P	-3.26038700	-0.11136200	0.18067500
O	-3.13114300	0.64748000	1.60749800
O	-4.32042200	0.65969200	-0.75982400
C	-5.72864900	0.81533700	-0.47006100
H	-5.88702000	1.77457800	0.02762000
H	-6.24622100	0.81241900	-1.42952300
H	-6.09600100	-0.00125900	0.15431500
C	-4.24011700	0.85858700	2.51105000
H	-3.81720000	0.92080400	3.51400600
H	-4.73434900	1.80097700	2.26396700
H	-4.95341100	0.03362000	2.46012900
N	-4.06870800	-1.54941200	0.58530900
C	-4.50106600	-2.36866100	-0.55816000
H	-3.66340700	-2.91618100	-1.01352700
H	-5.24200800	-3.09382900	-0.21022400
H	-4.96309200	-1.74416100	-1.32314900
C	-3.43475100	-2.36178700	1.63410300
H	-4.16220800	-3.09164400	2.00035100
H	-2.55464600	-2.90579000	1.26106300
H	-3.12661500	-1.73323400	2.47001100
C	2.85006000	-1.08201900	0.79852300
O	1.73694400	-1.20183300	0.19943100
O	3.06303500	-1.65189100	1.92558700
C	3.84603100	-0.21444500	0.07206000
C	3.28168100	-0.28606700	-1.36266500
H	3.45506800	0.63129300	-1.92458400
H	3.74442500	-1.11689800	-1.90805900
C	1.80660400	-0.51352000	-1.13070600
C	0.67170100	-0.17653000	-1.70808700
H	0.81052700	0.32272400	-2.66720900
C	5.24631700	-0.73587100	0.33277700
O	5.52921400	-1.28474100	1.39782500
O	6.09552500	-0.48082900	-0.63349200
C	7.47944100	-0.88504300	-0.41522900
H	7.52622200	-1.96584800	-0.27839800
H	8.00678100	-0.57771300	-1.31488700

H	7.87199200	-0.37926500	0.46758000
C	3.79057800	1.24615800	0.70512300
H	4.13389800	1.16531100	1.74126800
H	4.53318000	1.82797700	0.15219600
C	2.43099500	1.89096600	0.62809000
C	1.44175300	1.57479800	1.56921300
C	2.12177600	2.77059000	-0.41600000
C	0.15053500	2.07976300	1.43459000
H	1.67663800	0.92147400	2.40607100
C	0.83489600	3.28983700	-0.54454400
H	2.89042300	3.04685000	-1.13292900
C	-0.15697400	2.93003800	0.36943800
H	-0.61939500	1.80591600	2.14741400
H	0.60491600	3.96916300	-1.35888200
H	-1.16395900	3.31926500	0.26192300
H	4.06485000	-1.57708800	2.08141500

1a-TS1$_6$H$^+$[Au]

Au	0.96011900	-0.29636700	-0.54860100
P	3.20405100	-0.52468300	-0.06629000
O	3.45593200	-2.03484600	0.42417700
O	4.02123800	-0.19357000	-1.40966100
C	5.46680300	-0.19680900	-1.52708100
H	5.79001000	-1.17906100	-1.87650300
H	5.71681600	0.55879400	-2.27129300
H	5.93920000	0.04520800	-0.57340300
C	4.76029700	-2.58854000	0.73144200
H	4.59180900	-3.38748700	1.45276300
H	5.19515200	-3.00176700	-0.18061500
H	5.41922100	-1.83079800	1.15978000
N	3.96606700	0.41375700	1.11105600
C	4.08420400	1.85366400	0.83911000
H	3.16397900	2.39528600	1.09567100
H	4.90512200	2.25970300	1.43660900
H	4.30570300	2.02706900	-0.21418800
C	3.66838100	0.12041700	2.51905900
H	4.48643900	0.49888600	3.13835500
H	2.73582900	0.59694600	2.85103600
H	3.58317200	-0.95431500	2.67765400
C	-4.00305600	0.56024600	-1.24490700
O	-3.27201900	0.92746800	-2.15030100
O	-5.26121600	0.96414900	-1.20921400
C	-3.52182900	-0.28592300	-0.02681200
C	-2.21515500	-1.08228900	-0.27965100
H	-1.86248000	-1.48091100	0.67161300
H	-2.42438600	-1.94794100	-0.91554800
C	-1.15135500	-0.29227900	-0.95970900
C	-1.06343300	0.57122000	-1.85327900
H	-0.80113600	1.30457200	-2.58538000
C	-4.63879000	-1.24230900	0.41543600
O	-5.81900200	-0.91966800	0.44644000
O	-4.19268000	-2.41423900	0.84185700
C	-5.19603900	-3.33311800	1.35169200
H	-5.91937400	-3.56125100	0.56795600
H	-4.64261900	-4.22186500	1.64671100
H	-5.70777000	-2.88480100	2.20437800
C	-3.36684900	0.69602000	1.20754100
H	-4.29975200	1.25718500	1.30374300
H	-3.27292600	0.06832500	2.09946100
C	-2.18391600	1.62401300	1.11969800
C	-2.22926800	2.77756100	0.32683300
C	-1.00303500	1.32019800	1.80707700
C	-1.10307600	3.58724300	0.19090400
H	-3.14595300	3.04191700	-0.19118100
C	0.12138300	2.13904900	1.68486000
H	-0.96590800	0.44351300	2.44837200
C	0.07864800	3.26678600	0.86473800
H	-1.15237900	4.47862400	-0.42637000
H	1.01982900	1.90620400	2.24659200
H	0.94832500	3.91004300	0.77214800
H	-5.76008700	0.43961800	-0.53003100

1a-Int$_6$H$^+$[Au]

Au	0.98932200	-0.43746100	-0.50801200
P	3.25803400	-0.53732700	-0.05438700
O	3.64119800	-1.94780700	0.62812900
O	4.09219500	-0.31497900	-1.41588300
C	5.53362200	-0.22626100	-1.50020200
H	5.94251700	-1.21991100	-1.69498700
H	5.75490500	0.43064300	-2.34144300
H	5.95746300	0.18625100	-0.58251200
C	4.97831500	-2.32475800	1.03113800
H	4.86632400	-3.02885600	1.85564300
H	5.47991900	-2.81745200	0.19550600
H	5.55186900	-1.45534300	1.35899900
N	3.93874000	0.60396900	1.00652400
C	3.89643500	1.99829900	0.54258100
H	2.89270900	2.43698400	0.64200200
H	4.59590800	2.59012400	1.13966000
H	4.19771700	2.06156100	-0.50341600
C	3.55793900	0.47744200	2.41903500
H	4.25583900	1.06071200	3.02631400
H	2.53975700	0.84841400	2.60976900
H	3.60993700	-0.56361300	2.73789800
C	-3.77791700	0.28333500	-1.31833700
O	-2.88203000	0.74933600	-2.09493600
O	-4.99282400	0.44978400	-1.69018700
C	-3.43253800	-0.37786900	-0.00103600
C	-2.04989400	-1.06826300	-0.08794400
H	-1.70046400	-1.24816300	0.92986100
H	-2.15467900	-2.06054600	-0.54461700
C	-1.03320800	-0.27966000	-0.88125700
C	-1.45857700	0.55914400	-1.81400000
H	-0.91948200	1.19890900	-2.49236700
C	-4.55981300	-1.33189400	0.39100700
O	-5.72398500	-1.18635400	0.01164600
O	-4.17589600	-2.26503000	1.22882300
C	-5.20670700	-3.16897900	1.72352700
H	-5.65125800	-3.70808000	0.88650300
H	-4.68595100	-3.84521300	2.39694700
H	-5.97271800	-2.59701700	2.24829700
C	-3.46997700	0.74904600	1.12842800
H	-4.45999900	1.21516800	1.10157900
H	-3.38801800	0.20028400	2.07104300
C	-2.38230800	1.78456600	1.03151600
C	-2.54015100	2.91008200	0.21421600
C	-1.18562400	1.61587400	1.73660500
C	-1.50137200	3.82784500	0.06799400
H	-3.47790500	3.07247800	-0.31115700
C	-0.15076500	2.53964500	1.60168500
H	-1.06186700	0.75768100	2.39092500
C	-0.30088100	3.63983800	0.75657700
H	-1.63385500	4.69618300	-0.56922300
H	0.76783200	2.40522700	2.16338000
H	0.50314400	4.36147000	0.65207200
H	-5.57650500	-0.11055100	-1.05355400

(TfO•CHCl$_3$)⊂AuCav with C$_{11}$H$_{23}$ feet

C	-0.32789200	2.13542400	-3.60600800
C	-0.80358000	0.69185800	-3.72592000
C	-0.83884800	2.77456500	-2.31485500
H	-0.79341500	2.68537200	-4.42580100
C	-0.12795600	-0.35881200	-3.09703200
C	-1.96297000	0.36623800	-4.44204900
C	-0.12832500	2.70433500	-1.11306100
C	-2.04381800	3.48806000	-2.29985300
C	-0.56117200	-1.68625000	-3.14974300
H	0.76866200	-0.13065000	-2.53241000
C	-2.43850000	-0.93898300	-4.51880400
O	-2.67963700	1.38757000	-5.07428900
C	-0.55075900	3.33931500	0.05845200
H	0.80372700	2.15150800	-1.09585300
C	-2.51442300	4.12583400	-1.15770700
O	-2.76140300	3.59062900	-3.49424900
C	0.20064500	-2.81889200	-2.46954900
C	-1.73918600	-1.94320900	-3.86093000
H	-3.35591800	-1.15921400	-5.04773700
C	-3.87332900	1.70341500	-4.46806600
C	0.26053700	3.29106800	1.34856800

C	-1.75431800	4.05017000	0.00329500
H	-3.45517200	4.65902100	-1.16926400
C	-3.90349100	2.84200100	-3.59473000
H	-0.11381400	-3.74153700	-2.96104100
C	-0.19399100	-2.95609100	-1.00453300
O	-2.20704600	-3.26163000	-3.92387600
C	-0.08657800	2.04923900	2.16300000
H	-0.05713700	4.14800200	1.94508900
O	-2.17076000	4.73846300	1.14780400
C	0.47336000	-2.25498200	0.00024900
C	-1.21281100	-3.83063000	-0.60556600
C	-3.31885600	-3.54070400	-3.17568600
C	-6.04949800	1.26570000	-3.96396900
C	0.56186500	0.82991900	1.95743400
C	-1.08117000	2.08641600	3.14849300
C	-3.26075300	4.22171800	1.79444800
C	-6.06604500	2.37178900	-3.06166500
C	0.19677000	-2.41457300	1.35993900
H	1.26183300	-1.57103900	-0.28480100
C	-1.54971400	-4.00214700	0.73398400
O	-1.85737400	-4.58438300	-1.58001100
C	-3.13481200	-4.22417500	-1.92526200
C	0.27620300	-0.32201000	2.69509500
H	1.32352200	0.77812500	1.18961200
C	-1.40363800	0.96595100	3.90826600
O	-1.73405800	3.28819800	3.39312700
C	-3.03091700	3.41041200	2.95683300
C	0.97477300	-1.64823400	2.42298300
C	-0.83561100	-3.29465300	1.69429900
H	-2.34692500	-4.67859200	1.01403000
C	-5.56851300	-3.53334800	-2.82551200
C	-0.72073800	-0.21952700	3.66970700
H	-2.18479400	1.02461600	4.65492100
C	-5.49732700	3.89161000	2.01382100
H	0.92565700	-2.23634000	3.34408000
O	-1.16649300	-3.51645300	3.04626000
C	-5.38343800	-4.20482400	-1.57836100
O	-1.06447700	-1.34563600	4.44583500
C	-5.26530100	3.03546700	3.13298500
C	-7.22767300	2.63176900	-2.29942500
C	-7.20365000	0.46087700	-4.10083100
C	-6.50985600	-4.54720900	-0.79676500
C	-6.87600100	-3.21581300	-3.25734000
C	-6.36223400	2.39993900	3.75747000
C	-6.81837300	4.11732500	1.56836300
C	2.46409300	-1.47114700	2.07027200
H	2.92987100	-0.87984000	2.86731600
H	2.57457500	-0.87282900	1.15912500
C	1.72910000	-2.70634000	-2.62102800
H	2.17430700	-3.51080800	-2.02660100
H	2.09778100	-1.77136800	-2.18115900
C	1.20023600	2.25840400	-3.76570000
H	1.48735200	1.75312700	-4.69559600
H	1.71668600	1.71647200	-2.96382400
C	1.77180300	3.43934100	1.08878400
H	1.91763000	4.33091000	0.46745800
H	2.15022100	2.60051500	0.49161700
C	-7.87228100	3.49900600	2.20492800
C	-7.64237600	2.63046000	3.29846400
H	-8.48358900	2.13474500	3.77055700
H	-8.88731400	3.66081300	1.85956900
H	-6.16579500	1.73880900	4.59455800
H	-6.96147900	4.77346900	0.71688500
C	-8.32401800	0.73770000	-3.34842700
H	-7.16456400	-0.36923800	-4.79713400
C	-8.33437500	1.82432100	-2.44078100
H	-7.19906100	3.45297500	-1.59316500
C	-7.95939200	-3.57042700	-2.48195700
H	-6.98797000	-2.70090900	-4.20422400
C	-7.77549400	-4.24100700	-1.24875400
H	-6.34450700	-5.05484000	0.14724500
H	-8.64162500	-4.49949100	-0.64884000
H	-8.96449900	-3.32899700	-2.81239100
H	-9.21773800	2.00585300	-1.83818200
H	-9.20522200	0.11016200	-3.43614800

P	-2.11890600	-2.44310000	3.82255000
N	-2.62526600	-3.34518600	5.11104100
C	-1.66490500	-4.16247100	5.86210700
C	-3.74342600	-2.82753800	5.90520500
H	-0.91014200	-4.56202300	5.18536900
H	-2.20198300	-4.99965500	6.31718800
H	-1.17399400	-3.58166600	6.65334900
H	-4.43460700	-2.27535800	5.26373700
H	-3.40091500	-2.16487200	6.71084100
H	-4.28294700	-3.67123300	6.34494200
Au	-3.64113300	-1.43921900	2.52866700
N	-3.99493900	2.82925500	3.60322000
N	-4.45420000	4.47455500	1.35157400
N	-4.96162000	3.16432600	-2.91277600
N	-4.91543200	0.95189000	-4.66226200
N	-4.49727600	-3.20456600	-3.60889300
N	-4.12735900	-4.53724000	-1.14730100
S	-4.47899300	0.67512000	0.42054400
O	-5.57576100	1.54305500	-0.00701600
O	-5.02653900	-0.46720600	1.29696400
O	-3.24543400	1.26557200	0.95044700
C	-3.92207600	-0.24865400	-1.09496800
F	-4.95827200	-0.67861300	-1.81173400
F	-3.17831100	0.57913800	-1.83310800
F	-3.16565400	-1.30756100	-0.75121000
C	-8.14886200	-0.00740300	1.08950700
H	-7.36922600	0.74852700	1.04728700
Cl	-8.03021400	-0.85864600	2.66165800
Cl	-7.91272200	-1.12909800	-0.26387000
Cl	-9.73940100	0.82246300	0.95273900
C	2.60968500	3.55544300	2.36360900
H	2.42646100	2.68412700	3.00456900
H	2.27917900	4.43137200	2.93783300
C	4.10886000	3.66215800	2.07287300
H	4.42515600	2.78623000	1.48803700
H	4.29707200	4.53558000	1.43301000
C	4.96996300	3.76126500	3.33450000
H	4.77365600	2.89090700	3.97654000
H	4.66318700	4.64146400	3.91634000
C	6.46992300	3.84481900	3.03872100
H	6.77395000	2.96435700	2.45493200
H	6.66541800	4.71499500	2.39645900
C	7.33599500	3.94019300	4.29765300
H	7.03462800	4.82226000	4.87986000
H	7.13833700	3.07132700	4.94127500
C	8.83582900	4.01831600	3.99940500
H	9.13669100	3.13580900	3.41726700
H	9.03304400	4.88666700	3.35515900
C	9.70331100	4.11432100	5.25747100
H	9.50517200	3.24647300	5.90232500
H	9.40312000	4.99749700	5.83877000
C	11.20305800	4.19084300	4.95879800
H	11.50392100	3.30738100	4.37788900
H	11.40161500	5.05834000	4.31353000
C	12.07168500	4.28785300	6.21664800
H	11.77045300	5.17114900	6.79569500
H	11.87205500	3.42080400	6.86066700
C	13.56860100	4.36327900	5.90699100
H	14.16635300	4.43190600	6.82154700
H	13.90218700	3.47659600	5.35603500
H	13.79975200	5.23951800	5.29076300
C	3.22753300	-2.78578900	1.88709200
H	2.70150900	-3.42320900	1.16653600
H	3.23622100	-3.33796900	2.83670600
C	4.66487700	-2.55680400	1.40902500
H	4.64413400	-2.05434300	0.43131100
H	5.16783900	-1.86125800	2.09553500
C	5.49396000	-3.84004800	1.30698900
H	5.02724400	-4.52760600	0.58784800
H	5.47220800	-4.35525300	2.27755200
C	6.95273200	-3.58608900	0.91177900
H	6.99599200	-3.18358300	-0.10994900
H	7.36512800	-2.80311700	1.56373100
C	7.83942400	-4.83029600	1.01825300
H	7.48929400	-5.59885700	0.31527100

H	7.72186400	-5.26290000	2.02187000
C	9.32427500	-4.54427400	0.77433600
H	9.46957100	-4.19648600	-0.25722100
H	9.63880400	-3.71349200	1.42154800
C	10.22648800	-5.75207700	1.04304000
H	10.04636500	-6.11266800	2.06563100
H	9.94154300	-6.57877700	0.37824400
C	11.71797200	-5.44992100	0.87505700
H	11.99266900	-4.61515500	1.53536900
H	11.90836300	-5.10052900	-0.14853800
C	12.62198100	-6.64869900	1.17807800
H	12.40436200	-7.01610900	2.19000300
H	12.37288600	-7.47227800	0.49633300
C	14.11230800	-6.32095500	1.06265200
H	14.39586100	-5.52616900	1.76183200
H	14.73415300	-7.19505500	1.28083200
H	14.36423900	-5.97780300	0.05346200
C	2.22404000	-2.78652400	-4.06557300
H	1.73547900	-2.00466500	-4.65787300
H	1.91506600	-3.74541000	-4.50336900
C	3.74766300	-2.63468600	-4.17701100
H	4.02835500	-2.60652600	-5.23764800
H	4.04480100	-1.66210100	-3.75870100
C	4.54145800	-3.74895100	-3.48517800
H	4.30137600	-3.77730200	-2.41438000
H	4.22321100	-4.71919400	-3.89257000
C	6.05722700	-3.60764700	-3.64260000
H	6.38852500	-2.67154500	-3.17098600
H	6.30602300	-3.51407400	-4.70892300
C	6.83467200	-4.78567900	-3.04959400
H	6.58725500	-4.89147100	-1.98392800
H	6.49772000	-5.71381700	-3.53219900
C	8.35145100	-4.65774300	-3.21015400
H	8.58681700	-4.44207300	-4.26183600
H	8.70793800	-3.79025400	-2.63691800
C	9.11621200	-5.90987300	-2.77462100
H	8.76262400	-6.76914400	-3.36144500
H	8.87696800	-6.13933400	-1.72790200
C	10.63281000	-5.77968300	-2.93256100
H	10.86901200	-5.52094400	-3.97445500
H	10.99014000	-4.93682100	-2.32452800
C	11.39816900	-7.04660500	-2.54101700
H	11.15244400	-7.30963600	-1.50477100
H	11.04825300	-7.88501100	-3.15790400
C	12.91396300	-6.89894200	-2.68568400
H	13.43970400	-7.80752300	-2.37532200
H	13.19282500	-6.68870900	-3.72434200
H	13.28771200	-6.07253300	-2.07149300
C	1.70138400	3.70474500	-3.78077900
H	1.31054100	4.23370700	-2.90380700
H	1.29341300	4.22432200	-4.65797600
C	3.22907000	3.80105000	-3.77983800
H	3.63475000	3.29554600	-4.66739200
H	3.62035000	3.24965300	-2.91233500
C	3.74633400	5.24089400	-3.72876700
H	3.31771900	5.74574300	-2.85157200
H	3.37894400	5.79302600	-4.60482200
C	5.27330800	5.33552700	-3.66931500
H	5.70420500	4.83845500	-4.54982400
H	5.63651100	4.77356700	-2.79701500
C	5.79241300	6.77391700	-3.59386000
H	5.35498900	7.27137800	-2.71678200
H	5.43618300	7.33568800	-4.46867500
C	7.31892200	6.86678200	-3.51861700
H	7.75696800	6.37189000	-4.39684700
H	7.67404700	6.30190100	-2.64505200
C	7.83858000	8.30469300	-3.43600000
H	7.39873100	8.79957800	-2.55864100
H	7.48507400	8.86935900	-4.31018100
C	9.36483200	8.39737000	-3.35678800
H	9.80543600	7.90334600	-4.23442200
H	9.71873400	7.83213400	-2.48282500
C	9.88536900	9.83543100	-3.27245100
H	9.44422400	10.32784700	-2.39539700
H	9.53146000	10.39893100	-4.14620400
C	11.41161300	9.91688500	-3.19305800
H	11.87691000	9.45993500	-4.07381000
H	11.75734800	10.95390600	-3.13368900
H	11.78900200	9.38826200	-2.31032100

SolvOpt-TfO•[Au]

Au	-0.09670700	-0.65834100	-0.30259500
P	-2.22642200	-0.00321600	-0.10524100
O	-2.76284900	0.56348400	-1.51242400
O	-3.13379000	-1.23435700	0.39290500
C	-4.53976900	-1.12405500	0.73670300
H	-5.13977100	-1.36392900	-0.14347700
H	-4.72255500	-1.85977900	1.52046100
H	-4.78190800	-0.12469700	1.10181000
C	-4.09079300	1.11775700	-1.70273600
H	-4.00951500	1.83050100	-2.52393900
H	-4.77763100	0.31412200	-1.97656200
H	-4.44002600	1.62664600	-0.80259000
N	-2.54978900	1.23111300	0.99723400
C	-2.31611500	0.88851400	2.40992700
H	-1.24432000	0.86347900	2.65252100
H	-2.79786300	1.64592700	3.03446000
H	-2.75193500	-0.08249000	2.64810600
C	-1.99607700	2.55006000	0.65135800
H	-2.49083400	3.30616000	1.26745500
H	-0.91387400	2.60060800	0.83745700
H	-2.18263700	2.78304400	-0.39764600
S	2.94935800	-0.55056600	0.53079300
O	4.24542400	-1.23010700	0.50064100
O	1.95235000	-1.21904300	-0.42759500
C	3.22891300	1.08051000	-0.31655700
O	2.35958600	-0.20165200	1.83121200
F	4.14789500	1.78548200	0.34974800
F	2.08731600	1.79152000	-0.35151100
F	3.64757500	0.89028100	-1.57133300

SolvOpt-(TfO•1a)[Au]

Au	-2.14895000	0.50230300	-0.27561200
P	-2.46651700	-1.74986200	-0.57133900
O	-3.40663200	-2.30738300	0.61680000
O	-3.19768500	-1.94487800	-1.99841400
C	-3.46332500	-3.23301900	-2.60906600
H	-4.49220300	-3.52451900	-2.38670600
H	-3.34282700	-3.09783600	-3.68479700
H	-2.76758900	-3.99344100	-2.25191000
C	-3.86630900	-3.68145600	0.68688700
H	-4.01181200	-3.90435700	1.74449200
H	-4.81806800	-3.76702800	0.15751000
H	-3.13418700	-4.37050900	0.26254100
N	-1.17556300	-2.82015700	-0.62187300
C	-0.22240700	-2.62214300	-1.73121600
H	0.42995100	-1.75838100	-1.56619500
H	0.40107600	-3.51525100	-1.81110200
H	-0.76005200	-2.48428700	-2.67112100
C	-0.51075600	-3.14139000	0.65283500
H	0.05527500	-4.06666800	0.52051900
H	0.19228200	-2.36150400	0.95941700
H	-1.25045700	-3.29235200	1.43953800
C	1.17040600	1.52410500	-0.73143900
O	0.36509500	0.84448100	-1.35543900
O	2.39971500	1.15770800	-0.43967800
H	2.59145400	0.20022000	-0.73580000
C	0.86937500	2.93347100	-0.20187300
C	-0.49443700	3.44437300	-0.73706200
H	-0.56795200	4.51602000	-0.51364700
H	-0.49999800	3.33912800	-1.82496800
C	-1.72722800	2.84579100	-0.21805200
C	-2.86353700	2.58567300	0.17340700
H	-3.85563300	2.60673200	0.57826800
C	1.93147400	3.86464400	-0.82370100
O	2.60707000	4.66166600	-0.21189300
O	1.98573300	3.68858900	-2.15175900
C	2.96072700	4.48621000	-2.85730100
H	3.96520700	4.26044300	-2.49262500
H	2.86248400	4.20504200	-3.90509800

H	2.75057600	5.54974700	-2.72310900
C	1.02748700	3.00261100	1.34143500
H	2.06492000	2.74057200	1.56245600
H	0.89680400	4.04761100	1.63267500
C	0.08945700	2.12547600	2.13767200
C	0.24283700	0.73072700	2.16127000
C	-0.95310700	2.69851800	2.87573300
C	-0.65732600	-0.06791200	2.86976700
H	1.07354900	0.25733200	1.64806700
C	-1.85175400	1.90284100	3.58673300
H	-1.06899300	3.77906000	2.87945400
C	-1.71403300	0.51319400	3.57523600
H	-0.51996600	-1.14409100	2.88021000
H	-2.65537400	2.36714200	4.15135200
H	-2.40991600	-0.11063800	4.12867000
S	3.06640800	-2.18149200	0.09776000
O	2.44857600	-1.63240400	1.32313600
O	2.76231900	-3.58373000	-0.23145200
C	4.87932500	-2.17774200	0.47946100
O	2.98711900	-1.24974600	-1.08092400
F	5.29436700	-0.93182100	0.75349800
F	5.58440300	-2.64115500	-0.56248600
F	5.13356000	-2.95430200	1.54309000

SolvOpt-1a-TS1$_{5anti}$[Au]

Au	-2.45900300	-0.17315800	-0.78607900
P	-4.65549200	-0.25238800	-0.11952500
O	-5.29392400	1.22221500	-0.28006200
O	-5.41855100	-1.30585000	-1.07288800
C	-6.80493500	-1.70521200	-0.92257900
H	-7.40385300	-1.16709400	-1.66016400
H	-6.84739300	-2.77663300	-1.12412900
H	-7.17110800	-1.50294600	0.08505600
C	-6.69927900	1.51507800	-0.07514000
H	-6.76227900	2.58712200	0.11377500
H	-7.25582200	1.26744600	-0.98190500
H	-7.10076400	0.96526700	0.77815100
N	-5.11851600	-0.72048000	1.43443200
C	-4.86927700	-2.11847900	1.80795200
H	-3.83698700	-2.27642300	2.14663300
H	-5.54699400	-2.39148700	2.62255100
H	-5.06170300	-2.78274400	0.96462200
C	-4.87721000	0.21934300	2.53498600
H	-5.56037600	-0.01879700	3.35614500
H	-3.84823400	0.15709100	2.91374400
H	-5.06843900	1.24393300	2.21488600
C	2.68435400	1.34140300	-0.81180400
O	2.23033800	0.71066900	-1.76338800
O	3.93119000	1.34744600	-0.42965400
C	1.74044100	2.19714900	0.05297100
C	0.37305100	2.16106400	-0.66313400
H	-0.43148700	2.41414200	0.03517000
H	0.37227300	2.91130600	-1.45873700
C	0.07807000	0.85048400	-1.23405500
C	-0.49864800	-0.20221400	-1.60201000
H	-0.20867200	-1.07156500	-2.17372000
C	2.26639800	3.64300600	0.02875400
O	1.92048800	4.47417500	-0.78734900
O	3.15782000	3.86811700	0.99431300
C	3.76334100	5.17861000	0.99830200
H	3.00187800	5.94916800	1.13884800
H	4.46129800	5.17182200	1.83466400
H	4.29248300	5.35225900	0.05874700
C	1.69874500	1.64962000	1.51345900
H	2.72831600	1.58529200	1.86970100
H	1.18602000	2.39416900	2.12872200
C	1.00399600	0.31616200	1.63799400
C	1.64028200	-0.86155700	1.22404000
C	-0.30467300	0.23954800	2.12926300
C	0.96898000	-2.08197100	1.25861600
H	2.66111300	-0.83746700	0.86304600
C	-0.97296400	-0.98520900	2.18332600
H	-0.80236400	1.14399900	2.47063100
C	-0.34327000	-2.14840000	1.73386500
H	1.47978000	-2.97608700	0.91526600
H	-1.98065700	-1.03207500	2.58216800
H	-0.86483300	-3.10051700	1.77014700
S	5.17692300	-1.82478100	-1.01082700
O	3.76770100	-2.20449500	-0.79729200
O	5.36473600	-0.42100100	-1.52739800
O	6.04735300	-2.80846800	-1.67006000
C	5.84788400	-1.68358600	0.71068500
F	5.17572700	-0.73723000	1.39601800
F	5.71824800	-2.84581200	1.36651600
F	7.14448300	-1.34568100	0.69758100
H	4.48621400	0.64297800	-0.94250300

SolvOpt-1a-Int$_{5anti}$[Au]

Au	-2.42406400	-0.30117500	-0.84330700
P	-4.65824300	-0.28505800	-0.23117600
O	-5.34615800	1.13746300	-0.59063700
O	-5.45084100	-1.48087300	-0.98296400
C	-6.84652600	-1.79695500	-0.77179000
H	-7.45806400	-1.22096100	-1.47058300
H	-6.95873600	-2.86196600	-0.98068200
H	-7.15152100	-1.58871600	0.25542100
C	-6.72002700	1.47703100	-0.28825800
H	-6.74552000	2.55431300	-0.11633400
H	-7.34862400	1.22861200	-1.14679300
H	-7.07443400	0.95364100	0.60166500
N	-5.08857700	-0.51024900	1.40087200
C	-4.75643600	-1.83083400	1.95366100
H	-3.67843300	-1.94666500	2.13577100
H	-5.28450000	-1.95649800	2.90384800
H	-5.07840700	-2.62267500	1.27585500
C	-4.70306200	0.57944600	2.30712000
H	-5.23181100	0.45039300	3.25647200
H	-3.62271000	0.58943200	2.51067000
H	-4.98391200	1.54601400	1.88700100
C	2.55354400	1.29499400	-0.66793400
O	1.83126400	0.41556900	-1.33509100
O	3.78404300	1.28312400	-0.68105900
C	1.66817600	2.26558300	0.11590700
C	0.28954300	1.99508500	-0.51008900
H	-0.52924900	2.05936500	0.20557900
H	0.11147200	2.72526700	-1.30565000
C	0.41902300	0.61019600	-1.08060900
C	-0.43447200	-0.37401200	-1.32514400
H	0.00287600	-1.28228200	-1.74106900
C	2.14155100	3.70457000	-0.11240000
O	1.56178700	4.51402600	-0.80668500
O	3.27118200	3.95993900	0.55351700
C	3.81896300	5.28329100	0.37754700
H	3.11280000	6.03770200	0.73204300
H	4.73020200	5.30139900	0.97421700
H	4.04715000	5.46171700	-0.67563600
C	1.74824500	1.91247500	1.64243600
H	2.79872400	1.94265700	1.93929400
H	1.22846000	2.71176300	2.17871500
C	1.12835200	0.57709800	1.97224100
C	1.84409000	-0.60873800	1.76425000
C	-0.19167900	0.49566300	2.43289100
C	1.24232200	-1.84918800	1.96973700
H	2.87464100	-0.56638800	1.43065800
C	-0.79210600	-0.74384400	2.65412700
H	-0.75188100	1.40943700	2.61321100
C	-0.08095400	-1.92067000	2.40970900
H	1.80938300	-2.75515600	1.78047500
H	-1.81445800	-0.79162600	3.01596800
H	-0.55147500	-2.88606100	2.57183300
S	4.92696700	-1.95693800	-1.00980500
O	3.56257900	-2.18193800	-0.54217000
O	5.07437000	-0.58238700	-1.76351700
O	5.64478600	-2.97259900	-1.76564800
C	5.92451000	-1.61130300	0.51899100
F	5.43286600	-0.52641600	1.13274200
F	5.83903900	-2.65754400	1.34114200
F	7.19973400	-1.39478000	0.20321000
H	4.48413000	0.16943100	-1.34643300

SolvOpt-1a-TS2$_{5anti}$[Au]

Au	-0.80670700	-0.41174000	-0.48582800
P	-2.67196100	-1.16383000	0.61339600
O	-2.34723000	-2.28564500	1.73638300
O	-3.40783400	0.09242500	1.30605200
C	-4.72589600	0.08407400	1.89791000
H	-4.62566900	0.00763600	2.98300600
H	-5.18875700	1.03810700	1.64017700
H	-5.33283100	-0.73849600	1.51573100
C	-3.36367200	-2.89643900	2.56641000
H	-2.94178900	-3.83580700	2.92602400
H	-3.58343900	-2.24498000	3.41553000
H	-4.27401600	-3.09368000	1.99694500
N	-3.90090100	-1.88897200	-0.31128700
C	-4.50845300	-1.00643400	-1.31868500
H	-3.84601300	-0.84953700	-2.18286800
H	-5.43716000	-1.46443700	-1.67198000
H	-4.74695100	-0.03470700	-0.88490700
C	-3.55360600	-3.20126600	-0.87462400
H	-4.46522500	-3.67247100	-1.25388200
H	-2.83415000	-3.11897400	-1.70326200
H	-3.12681400	-3.84766100	-0.10645200
C	4.26726600	-0.00637400	-1.18760600
O	3.12860700	0.15291600	-1.94160600
O	5.33550100	-0.21380500	-1.69542100
C	3.89937700	0.07938400	0.30110300
C	2.48802900	0.67382700	0.25298700
H	1.81136000	0.28063200	1.00868200
H	2.54445500	1.76040500	0.37952600
C	2.01696400	0.37072400	-1.14149300
C	0.77119300	0.36180200	-1.66152700
H	0.73264000	0.16998200	-2.73532400
C	4.89527300	1.00704300	1.00186300
O	4.66095200	2.15982000	1.30077400
O	6.05864900	0.39235000	1.23188700
C	7.08349100	1.19932200	1.84950200
H	6.74874100	1.55794600	2.82551500
H	7.94474900	0.54104400	1.95771100
H	7.33010800	2.05085100	1.21138800
C	3.94951900	-1.35843500	0.91834300
H	4.94172500	-1.77120200	0.72419400
H	3.84385000	-1.24559800	2.00099700
C	2.86945200	-2.27361900	0.39192800
C	3.00200600	-2.90183200	-0.85496400
C	1.69435800	-2.48103300	1.12549600
C	1.97509000	-3.69616500	-1.36534100
H	3.91492000	-2.77090900	-1.42929800
C	0.66845100	-3.28325000	0.62309000
H	1.57902300	-2.00530900	2.09568400
C	0.80350300	-3.88597800	-0.62920100
H	2.09468400	-4.17263100	-2.33395800
H	-0.23700000	-3.42180200	1.20320200
H	0.00459800	-4.50627200	-1.02494100
S	-0.70161500	3.25987400	0.11090400
O	-0.44144500	2.28294900	1.17328800
O	-0.08757900	2.82840500	-1.24240700
O	-0.45687800	4.67727300	0.36920700
C	-2.52959100	3.14935600	-0.25809700
F	-2.81995600	2.04782700	-0.96424400
F	-3.21310300	3.12044700	0.88977000
F	-2.91006300	4.21587200	-0.96713200
H	0.22005300	1.67854000	-1.32675500

SolvOpt-(TfO•2a)[Au]

Au	0.99131100	0.60322100	0.13856500
P	0.09362900	2.60509900	-0.27591900
O	-1.49959100	2.50471000	-0.15971200
O	0.66066500	3.71179300	0.75264400
C	0.15779000	5.06995500	0.77427300
H	-0.78962800	5.10059000	1.31783400
H	0.90422400	5.66273300	1.30367400
H	0.02541400	5.45952100	-0.23722200
C	-2.46032700	3.48763500	-0.60985700
H	-3.28388200	2.92396700	-1.04681600
H	-2.81447800	4.04952100	0.25725300
H	-2.02384900	4.16480200	-1.34568200
N	0.36216200	3.28505200	-1.79876600
C	1.73051100	3.75614100	-2.05722500
H	2.43299100	2.92208500	-2.20060800
H	1.72157500	4.36217300	-2.96774200
H	2.08532900	4.37610500	-1.23325100
C	-0.16977300	2.49516900	-2.92194500
H	-0.24782800	3.14356700	-3.79963500
H	0.48648800	1.64857400	-3.16545300
H	-1.16032900	2.10576000	-2.68525700
C	-3.02232700	-0.29317900	-0.64574700
O	-2.14252100	-0.15546700	-1.67709900
O	-3.86091000	0.53731600	-0.40474000
C	-2.77985500	-1.64221500	0.05466900
C	-1.40420100	-2.05723600	-0.47992400
H	-0.62037400	-1.79681800	0.23385500
H	-1.34008800	-3.12345300	-0.69245400
C	-1.23858500	-1.22725800	-1.72340400
C	-0.40780500	-1.35646700	-2.75001400
H	-0.40644900	-0.64949500	-3.57129400
C	-3.86066200	-2.61043900	-0.46902900
O	-3.63978000	-3.56717900	-1.18121300
O	-5.07754900	-2.26220100	-0.03675700
C	-6.16517000	-3.10058700	-0.47999400
H	-6.02688500	-4.12380200	-0.12319800
H	-7.06382000	-2.66287900	-0.04675800
H	-6.22526800	-3.09899600	-1.57069500
C	-2.91237700	-1.52079000	1.59853100
H	-3.92250200	-1.16555100	1.80937100
H	-2.82729800	-2.53184200	2.00945100
C	-1.90026800	-0.61497600	2.26030800
C	-2.16731900	0.75126900	2.42512900
C	-0.68331600	-1.12252100	2.73720300
C	-1.24206100	1.58797500	3.04884000
H	-3.10229100	1.15669700	2.05373100
C	0.24880600	-0.28596200	3.35383400
H	-0.46282600	-2.18016700	2.62443700
C	-0.02957500	1.07390500	3.51295900
H	-1.46634900	2.64368700	3.16877700
H	1.18910000	-0.69694600	3.70842800
H	0.69321900	1.72652600	3.99347000
H	0.29109400	-2.18319600	-2.76883600
S	2.63375300	-1.99434000	-0.66622500
O	2.10326800	-3.30108800	-1.07353700
O	3.04014800	-1.04877500	-1.71627500
O	1.79746600	-1.35038700	0.44325900
C	4.18102800	-2.35310500	0.29317600
F	3.91262200	-3.16144400	1.32432400
F	4.69994000	-1.21037800	0.76490600
F	5.08353700	-2.94192100	-0.49873500

SolvOpt-(1a)$_2$

C	1.87524100	-0.81687900	0.15292200
O	1.33167200	-0.96593600	-0.94166500
O	1.23274900	-0.73890000	1.29767000
H	0.23679600	-0.84405600	1.15648800
C	3.38311900	-0.65001900	0.30414800
C	4.07143500	-1.04662100	-1.02921900
H	3.76925800	-2.06350100	-1.29477000
H	3.69814400	-0.38779100	-1.81797100
C	5.52631900	-0.96305000	-0.94928200
C	6.72877400	-0.88613300	-0.85864700
H	7.79238400	-0.82630300	-0.78483200
C	3.86366300	-1.58904100	1.41914600
O	4.50944100	-1.25545500	2.38726000
O	3.48433100	-2.85029700	1.15941000
C	3.88883000	-3.84018700	2.12785400
H	3.46965100	-3.60412100	3.10864900
H	3.49292700	-4.78644400	1.76063600
H	4.97843500	-3.88094500	2.19440400
C	3.68654500	0.81978500	0.71512400
H	3.22229200	0.99646500	1.68765600
H	4.76719800	0.89306500	0.85344000
C	3.20961500	1.83644900	-0.29425200
C	1.89435700	2.32107900	-0.25586600

C	4.06344600	2.29238800	-1.30745700
C	1.44157000	3.23153200	-1.21085800
H	1.22272300	1.98589400	0.52934500
C	3.61309500	3.20398300	-2.26394600
H	5.08593000	1.92691700	-1.34438900
C	2.29974000	3.67463000	-2.21961300
H	0.42071500	3.59816900	-1.16322100
H	4.28940000	3.54753300	-3.04141200
H	1.94786400	4.38484900	-2.96199200
C	-1.87534600	-0.81656000	-0.15301600
O	-1.33177800	-0.96586500	0.94153400
O	-1.23284800	-0.73838400	-1.29775200
H	-0.23689500	-0.84367200	-1.15662100
C	-3.38323800	-0.64970000	-0.30418600
C	-4.07151200	-1.04658600	1.02912000
H	-3.76929100	-2.06351100	1.29445300
H	-3.69822200	-0.38790900	1.81799700
C	-5.52640000	-0.96305100	0.94924100
C	-6.72885900	-0.88613400	0.85864500
H	-7.79243200	-0.82598800	0.78456700
C	-3.86379300	-1.58853400	-1.41932800
O	-4.50986500	-1.25483800	-2.38720700
O	-3.48467200	-2.84987400	-1.15968100
C	-3.88950900	-3.83965800	-2.12809200
H	-3.47043500	-3.60364400	-3.10894400
H	-3.49373400	-4.78600200	-1.76096100
H	-4.97913200	-3.88020600	-2.19447800
C	-3.68672000	0.82017600	-0.71489900
H	-3.22272500	0.99697300	-1.68753400
H	-4.76740500	0.89349800	-0.85292200
C	-3.20948300	1.83671200	0.29447300
C	-1.89432400	2.32157200	0.25553500
C	-4.06289100	2.29233700	1.30817200
C	-1.44119800	3.23190600	1.21047100
H	-1.22303500	1.98673500	-0.53012300
C	-3.61219600	3.20379500	2.26463200
H	-5.08530700	1.92672100	1.34552500
C	-2.29893400	3.67464500	2.21975500
H	-0.42045100	3.59877300	1.16238300
H	-4.28817000	3.54709500	3.04249700
H	-1.94680600	4.38476800	2.96210800
